# Diversity-oriented synthesis of nanographenes enabled by dearomative annulative π-extension

**DOI:** 10.1038/s41467-021-24261-y

**Published:** 2021-06-24

**Authors:** Wataru Matsuoka, Hideto Ito, David Sarlah, Kenichiro Itami

**Affiliations:** 1 Graduate School of Science, Nagoya University, Nagoya, Japan; 2 JST-ERATO, Itami Molecular Nanocarbon Project, Nagoya, Japan; 3 Department of Chemistry, University of Illinois, Urbana, IL United States; 4 Institute of Transformative Bio-Molecules (WPI-ITbM), Nagoya University, Nagoya, Japan

**Keywords:** Synthetic chemistry methodology, Synthesis and processing

## Abstract

Nanographenes and polycyclic aromatic hydrocarbons (PAHs) are among the most important classes of compounds, with potential applications in nearly all areas of science and technology. While the theoretically possible number of nanographene structures is extraordinary, most of these molecules remain synthetically out of reach due to a lack of programmable and diversity-oriented synthetic methods, and their potentially huge structure-property diversity has not been fully exploited. Herein we report a diversity-oriented, growth-from-template synthesis of nanographenes enabled by iterative annulative π-extension (APEX) reactions from small PAH starting materials. The developed dearomative annulative π-extension (DAPEX) reaction enables π-elongation at the less-reactive *M*-regions of PAHs, and is successfully combined with complementary APEX reactions that occur at *K*- and *bay*-regions to access a variety of previously untapped nanographenes.

## Abstract (web summary)

Nanographenes and polycyclic aromatic hydrocarbons (PAHs) are important classes of compounds with numerous applications, but challenging to access due to a lack of programmable and diversity-oriented methods. Here, the authors report a diversity-oriented, growth-from-template synthesis of nanographenes enabled by iterative annulative π-extension reactions from small PAH starting materials.

## Introduction

**Fig. 1:**
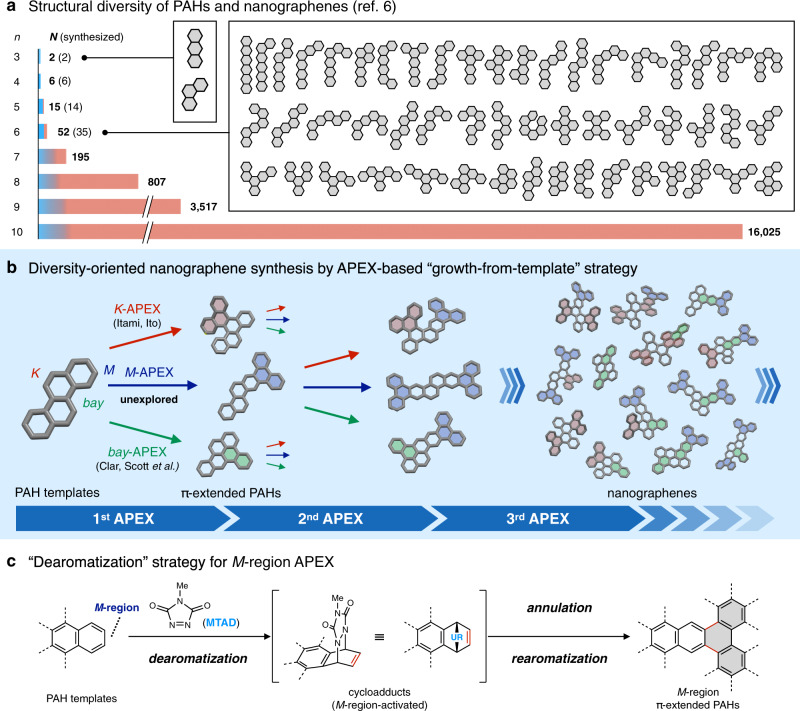
Synthetic strategy toward structurally diverse nanographenes. **a** Structural diversity of PAHs and nanographenes. **b** Diversity-oriented synthesis of nanographenes by APEX-based growth-from-template method. Red, blue, and green hexagons represent newly constructed hexagons created by *K*-APEX, *M*-APEX, and *bay*-APEX reactions, respectively. **c** DAPEX strategy toward achieving the formal *M*-region-selective APEX reaction. Gray hexagons represent newly constructed hexagons created by the present *M*-APEX reaction. *n* number of hexagons contained in a nanographene or a PAH structure, *N* number of possible structures of nanographenes and PAHs.

Nanographenes and polycyclic aromatic hydrocarbons (PAHs) are among the most important classes of compounds, with potential applications in nearly all areas of materials science, and particular promise in organic electronics, biology, and space science^1–4^. Nanographenes and PAHs are structurally simple assemblies of benzene-based hexagons and one can imaginarily build up a range of structures with ease^5^. While seemingly simple, the structural diversity of nanographenes and PAHs is extraordinary (Fig. 1a), and the enumeration of these molecules (also known as Kekuléan fusenes) using graph theory has been of central interest in mathematical chemistry^6^. The number of possible structures of nanographenes and PAHs (*N*) from *n* hexagons rapidly becomes extremely high even in relatively small systems (*N* = 52 when *n* = 6; *N* = 195 when *n* = 7; *N* = 16,025 when *n* = 10) and increases roughly fivefold for every additional hexagon (*N* > 10^15^ when *n* = 25). Since the key characteristics of nanographenes and PAHs—such as their photophysical, electronic, magnetic, and self-assembling properties—are determined by their structures^1–4^, a comprehensive evaluation of nanographenes is required to fully understand the relationship between their molecular structures and properties. However, to the best of our knowledge, most of the theoretically possible structures remain unsynthesized and unexplored in experimental science in reality (Fig. 1a). Moreover, while nature provides a tremendous variety of PAHs as components of fuel, coal, and tar, as well as side-products in combustion, interstellar dust, and meteorites^4,7^, only an extremely limited number of pure PAHs can be reliably accessed from these natural sources due to challenges in isolation.

The only logical way to access and utilize a greater diversity of structurally pure PAHs and nanographenes is to draw inspiration from organic synthesis, where a target molecular entity is built up from a template (seed) molecule with structural precision. While decades of research into the synthetic chemistry of PAHs has uncovered a number of exciting properties and applications^1–4,7–13^, thereby contributing significantly to the emergence of nanographene science, the synthetic bottleneck remains considerable. Owing to the lack of predictable and diversity-oriented synthetic methods for their synthesis^14^, the huge structure-property diversity of nanographenes has not been fully exploited. One intuitive, programmable, and potentially diversity-oriented synthetic strategy would be the “growth-from-template” approach, in which nanographenes are built up from small PAH templates by regioselective, template-elongating annulative π-extension (APEX) reactions (Fig. 1b)^15^. The regioselective and direct construction of fused aromatic rings on a peripheral region of an unfunctionalized aromatic template would result in the formation of another, larger aromatic template, and repeating this protocol would grant access to diverse nanographene structures. This APEX methodology is very different from typical nanographene syntheses, which consist of multiple synthetic steps including prefunctionalization of aromatics, component assembly into polyarylene precursors, and dehydrocyclization^8–13^. While challenges exist in obtaining APEX regioselectivity among the structurally and electronically similar C–H bonds and PAH regions found in unfunctionalized aromatic templates, this diversity-oriented strategy enables access to a range of previously untapped nanographene structures in an intuitive fashion, thereby revolutionizing nanographene synthesis.

To this end, we have established several palladium-catalyzed APEX reactions that occur at the *K*-region (convex armchair edge) of PAHs (Fig. 1b, *K*-APEX), realizing the synthesis of various nanographenes^16,17^. Clar, Scott, and others have achieved Diels–Alder-type APEX reactions at the *bay*-region (concave armchair edge) of PAHs (Fig. 1b, *bay*-APEX)^18–21^. To realize a diversity-oriented nanographene synthesis, APEX reactions that occur at other peripheral regions such as the terminal acene-like *M*-region are essential (Fig. 1b). *M*-region-selective APEX (*M*-APEX) is particularly challenging because the *M*-region is much less reactive compared to the “olefinic” *K*-region and “diene-like” *bay*-region—indeed, *M*-region (C2 and C3)-selective functionalizations of phenanthrene have been scarcely reported^22,23^.

We have previously developed several dearomative functionalizations by exploiting the ability of 4-methyl-1,2,4-triazoline-3,5-dione (MTAD) as an arenophile, which can participate in photo-induced [4 + 2] cycloaddition with aromatic compounds^24–26^. During the study, we have discovered that the [4 + 2] cycloaddition of polyarenes with MTAD preferentially occurs on a terminal acene-like structure, likely to minimize steric repulsion, and the reaction furnishes an activated olefin moiety on the *M*-region of the PAH starting materials (Fig. 1c). Here, we report the long-sought-after, broadly applicable *M*-APEX reaction with a concept of “dearomative APEX” (DAPEX), involving the MTAD-mediated dearomative activation of an aromatic ring, annulation, and finally rearomatization (Fig. 1c). The diversity-oriented synthesis of nanographenes by combining *K*-, *M*- and *bay*-APEX reactions is also demonstrated.

## Results

**Fig. 2:**
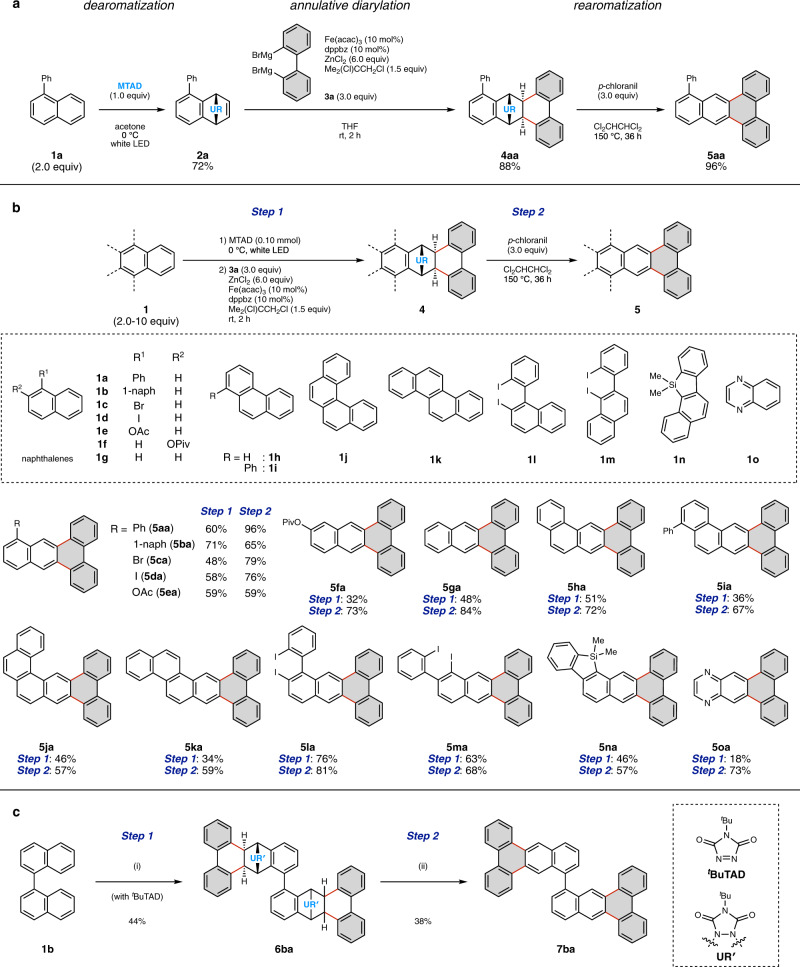
*M*-APEX reactions enabled by dearomative strategy. **a** Optimized reaction conditions. **b** Scope of aromatic templates. **c** Double *M*-APEX reaction of 1,1′-binaphthyl (**1b**). Reaction conditions. (i) **1b** (1.0 equiv), ^*t*^BuTAD (2.2 equiv), AcOMe, 0 °C, white LED, then **3a** (6.0 equiv), Fe(acac)_3_ (20 mol%), dppbz (20 mol%), ZnCl_2_ (12 equiv), 1,2-dichloroisobutane (3.0 equiv), THF, rt, 2 h; (ii) **6ba** (1.0 equiv), *p*-chloranil (6.0 equiv), 1,1,2,2-tetrachloroethane, 150 °C, 36 h. acac, acetylacetate; THF, tetrahydrofuran; Ac, acetyl. Gray hexagons represent newly constructed hexagons created by the present *M*-APEX reaction.

We began our investigations by establishing conditions for the annulation of cycloadduct **2a**, which is readily prepared by the photo-induced [4 + 2] cycloaddition of 1-phenylnaphthalene (**1a**) with MTAD^24–27^, to form a precursor of the desired *M*-APEX products (Fig. 2a). Through extensive screening of various catalysts, π-extending agents, and additives, we found that the treatment of **2a** with bis-Grignard reagent **3a** (3.0 equiv) in the presence of Fe(acac)_3_ (10 mol%), 1,2-bis(diphenylphosphino)benzene (dppbz, 10 mol%), ZnCl_2_ (6.0 equiv), and 1,2-dichloroisobutane (1.5 equiv) in THF at room temperature afforded the *exo*-cycloadduct **4aa** in 88% yield^28^. The relative configuration of **4aa** was determined by X-ray crystallographic analysis (see Supplementary Fig. 2). To our delight, the diarylated cycloadduct **4aa** was successfully converted into the corresponding *M*-APEX product **5aa** in 96% yield simply by treating with *p*-chloranil (3.0 equiv) in 1,1,2,2-tetrachloroethane at 150 °C for 36 h, likely via dehydrogenation followed by retro-[4 + 2] cycloaddition^27,29^.

With the optimal reaction conditions in hand, we investigated the substrate scope of PAHs and naphthalene derivatives in the DAPEX protocol (Fig. 2b). In all cases, annulative diarylations were conducted without isolating the cycloadduct intermediates formed in the dearomatization step. The reactions with naphthalene derivatives such as 1-phenylnaphthalene (**1a**), 1,1′-binaphthyl (**1b**), 1-bromonaphthalene (**1c**), 1-iodonaphthalene (**1d**), 1-acetoxynaphthalene (**1e**) and 2-pivaloxy naphthalene (**1f**), as well as naphthalene itself (**1g**) gave the intermediates of **5aa**–**5ga** in 32–71% yield (Step 1). Rearomatization of these intermediates successfully afforded the desired *M*-region APEX products **5aa**–**5ga** in 59–96% yield (Step 2). In addition to naphthalene derivatives, simple PAHs were found to be amenable to the described DAPEX protocol. For example, phenanthrene (**1h**), 1-phenylphenanthrene (**1i**), [4]helicene (**1j**), and chrysene (**1k**), which all feature both *K*- and *M*-regions, could be selectively transformed to the desired *M-*APEX products (**5ha**–**5ka**) as single constitutional isomers. In addition, previously reported π-extending agents for *K*-APEX^16,17^ including 2-iodo-1-(2-iodophenyl)naphthalene (**1l**), 1-iodo-2-(2-iodophenyl)naphthalene (**1m**) and 11,11-dimethyl-11*H*-benzo[*b*]naphtho[2,1-*d*]silole (**1n**), can be successfully π-extended on the *M*-regions of their naphthalene moieties to give the corresponding products **5la**, **5ma** and **5na**, respectively. Quinoxaline (**1o**) could also participate in the reaction sequence to afford phenanthro[9,10-*g*]quinoxaline (**5oa**), albeit in a low yield. For the reaction of 1,1′-binaphthyl (**1b**), two *M*-regions (C6–C7 and C6′–C7′) could be π-extended in the same reaction step by employing an excessive amount of arenophile (Fig. 2c). Treatment of **1b** with 2.2 equiv of 4-*tert*-butyl-1,2,4-triazoline-3,5-dione (^*t*^BuTAD) under photoirradiation gave the corresponding bis-cycloadduct, and treatment of this reaction mixture with bis-Grignard reagent **3a** under the standard conditions successfully afforded the precursor **6ba**. Rearomatization of **6ba** gave the corresponding *M*-region π-extended product **7ba**.

**Fig. 3:**
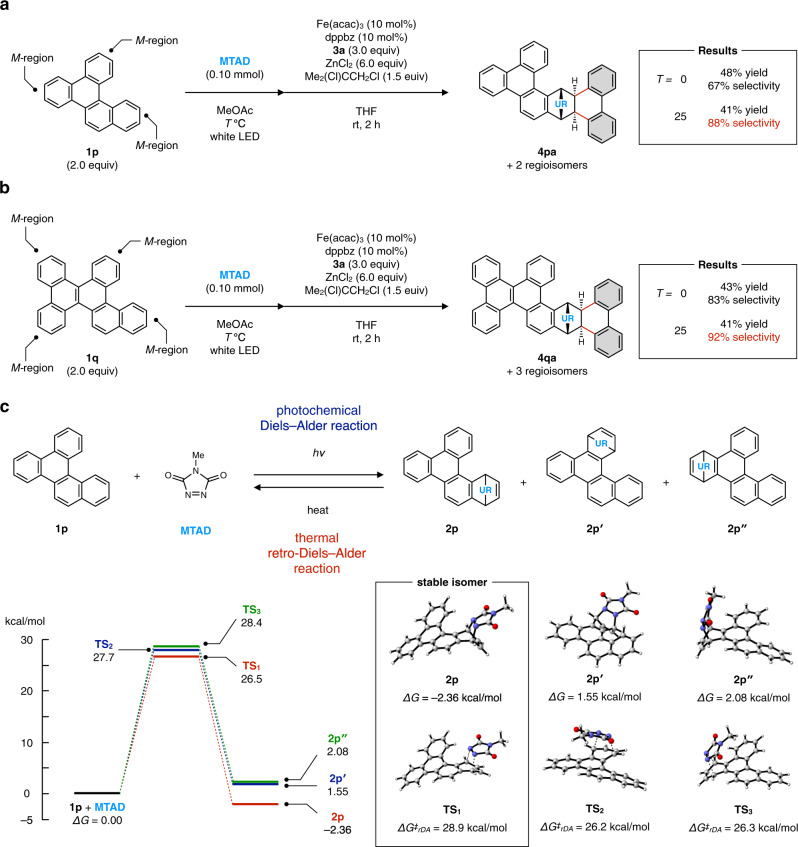
*M*-APEX reactions of PAHs containing multiple *M*-regions. **a** Dearomatization and annulative diarylation of benzo[*g*]chrysene (**1p**). **b** Dearomatization and annulative diarylation of dibenzo[*f,s*]picene (**1q**). **c** Evaluation of thermal stability of cycloaddducts **2p**, **2p′** and **2p″** at M06-2X/6-311++G(d,p)//M06-2X/6-31+G(d) level.

As demonstrated in Fig. 2b, the DAPEX protocol preferentially occurs at *M*-region of PAH templates over other peripheral regions such as *K*-region and *bay*-region. However, when a PAH template has two or more distinguishable *M*-regions, the protocol could potentially give *M*-APEX products as a mixture of regioisomers. To obtain insights into the regioselectivity of DAPEX protocol, dearomatization, and subsequent annulative diarylation of benzo[*g*]chrysene (**1p**) and benzo[*f,s*]picene (**1q**) were conducted (Fig. 3). As expected, the dearomatization and annulative diarylation of **1p** under standard reaction conditions afforded the corresponding diarylated products as a mixture of three regioisomers in 48% yield (Fig. 3a, *T* = 0). Further analysis of the products revealed that **4pa** was generated as a major regioisomer with 67% selectivity (see Supplementary Figs. 1 and 3 for details). To our delight, the regioselectivity could be further improved to 88% by conducting dearomatization at 25 °C (Fig. 3a, *T* = 25). Similarly, the dearomatization and annulative diarylation of **1q** under standard reaction conditions afforded **4qa** as a major product with 83% regioselectivity (Fig. 3b, *T* = 0), and the regioselectivity could be improved to 92% when dearomatization was conducted at 25 °C (Fig. 3b, *T* = 25). To unveil the origin of these high regioselectivities, we have evaluated the thermal stability of cycloadducts **2p**, **2p′**, and **2p″**, which are intermediates corresponding to **4pa** and its regioisomers, using DFT calculation at M06-2X/6-311++G(d,p)//M06-2X/6-31+G(d) level (Fig. 3c, see Supplementary Fig. 10 for details). **2p** was found to be more stable than starting materials (**1p** and MTAD), whereas **2p′** and **2p″** were thermodynamically unstable (*ΔG* = 1.55 and 2.08 kcal/mol, respectively). Furthermore, activation barriers for the thermal retro-Diels–Alder reaction^27,29^ of **2p** were estimated to be 28.9 kcal/mol, which is about 2.5 kcal/mol larger than that of **2p′** (*ΔG*^*‡*^_*rDA*_ = 26.2 kcal/mol) and **2p″** (*ΔG*^*‡*^_*rDA*_ = 26.3 kcal/mol). These results indicate that **2p** is both thermodynamically and kinetically more stable than **2p′** and **2p″**. Although regioselectivity in the photochemical Diels–Alder reaction cannot be experimentally or theoretically evaluated due to the transient nature of cycloadducts and multiple possible reaction pathways^27^, the regioselective formation of **4pa** shown in Fig. 3a can be rationalized as follows. At the dearomatization step, thermal retro-Diels–Alder reaction of cycloadducts took place along with the photochemical Diels–Alder reaction. Because the less stable isomers, **2p′** and **2p″** were preferentially decomposed to regenerate **1p** and MTAD, the most stable isomer **2p** would be accumulated in the reaction mixture, affording **4pa** as the major product of the iron-catalyzed annulative diarylation. When the dearomatization was conducted at 25 °C (*T* = 25), the retro-Diels–Alder reaction was accelerated and hence the regioselectivity was improved. Previously, Breton and Newton investigated the correlation between regioselectivity in Diels–Alder reactions of MTAD with substituted naphthalenes and experimentally determined rate constants for the retro-Diels–Alder reaction^29^. They observed that an initially formed photochemical mixture of constitutional isomers could equilibrate to a single product if this cycloadduct was sufficiently more stable toward retro-Diels–Alder reaction.

**Fig. 4:**
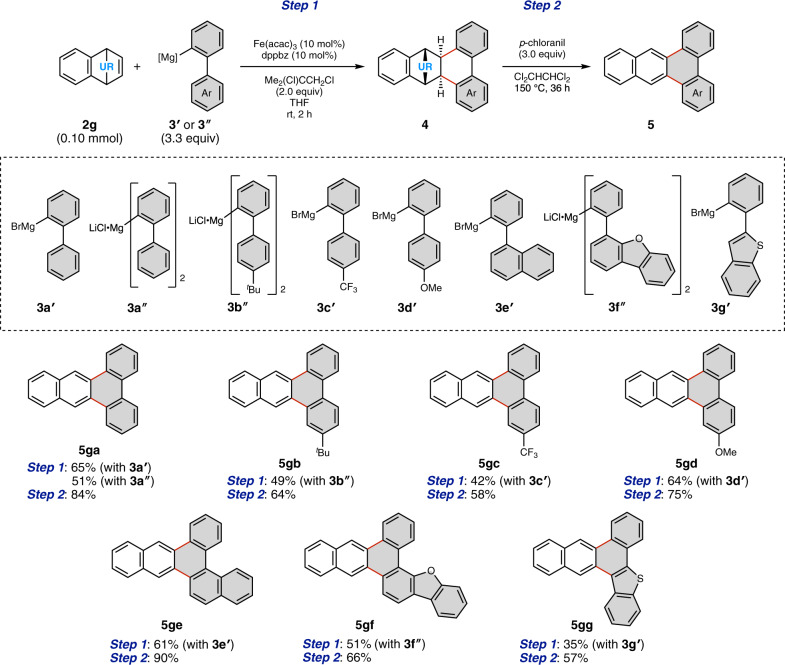
*M*-APEX reactions with Grignard reagents **3′** and **3″**. Reaction conditions for Step 1: **2g** (1.0 equiv), **3′** or **3″** (3.3 equiv), Fe(acac)_3_ (10 mol%), dppbz (10 mol%), 1,2-dichloroisobutane (2.0 equiv), THF, rt, 2 h. Reaction conditions for Step 2: **4** (1.0 equiv), *p*-chloranil (3.0 equiv), 1,1,2,2-tetrachloroethane, 150 °C, 36 h. Gray hexagons represent newly constructed hexagons created by the present *M*-APEX reaction.

We further investigated alternative arylation agents and reaction conditions to expand the scope of π-extending agents. To our delight, we found 2-biphenyl-1-yl magnesium bromide (**3a′**) also worked in the presence of Fe(acac)_3_ (10 mol%), dppbz (10 mol%), and 1,2-dichloroisobutane (2.0 equiv) to afford the precursor of **5ga** in 65% yield (Fig. 4, Step 1)^30^. It was also found that di(1,1′-biphenyl-2-yl) magnesium (**3a″**), which can be readily prepared from corresponding biarylyl bromide and ^*i*^PrMgCl·LiCl^31^, can be used as a π-extending agent (51% yield). As for other Grignard reagents, a series of 4′-substituted-1,1′-biphenyl-2-yl magnesium species (**3b″**, **3c′**, and **3d′**) afforded the corresponding annulation products, and these intermediates could also be rearomatized to give the *M*-region APEX products **5gb**–**5gd**. The reaction with 2-(1-naphthyl)-phenyl Grignard reagent **3e′** gave the π-extended PAH **5ge**, while the reactions with heteroarene-derived Grignard reagents **3f″** and **3g′** successfully generated polycyclic heteroaromatics **5gf** and **5gg**.

**Fig. 5:**
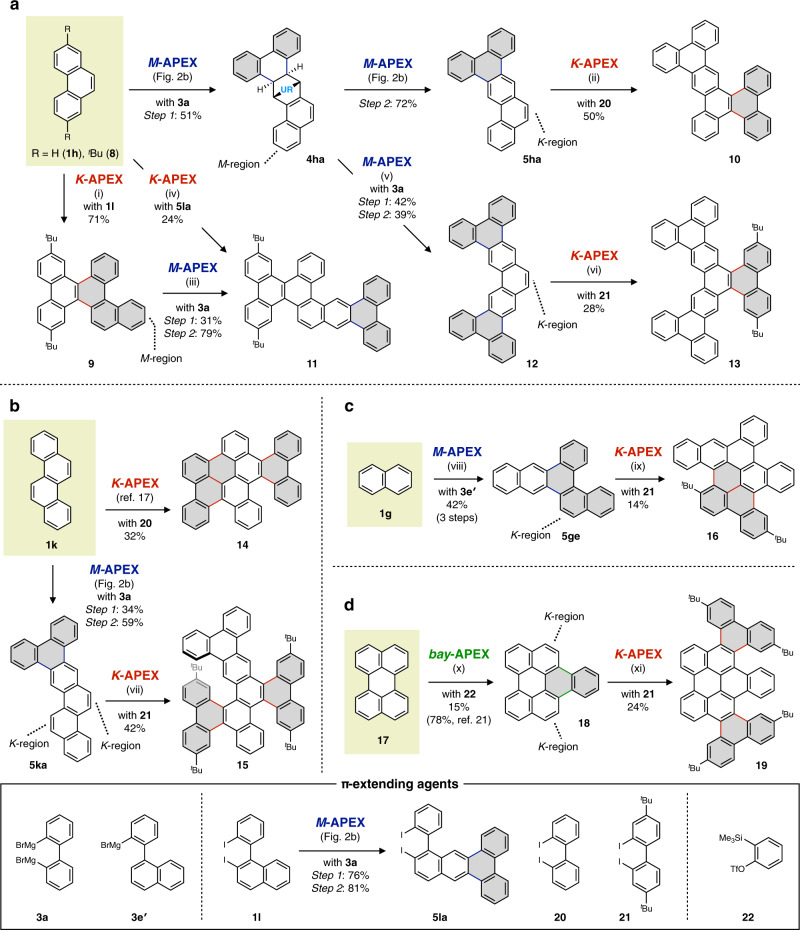
Diversity-oriented nanographene synthesis by growth-from-template method utilizing region-selective APEX reactions. **a** Nanographene synthesis from phenanthrene. **b** Nanographene synthesis from chrysene. **c** Nanographene synthesis from naphthalene. **d** Nanographene synthesis from perylene. Reaction conditions. (i) Pd(MeCN)_4_(BF_4_)_2_, **1l**, AgOPiv, TfOH, 1,2-dichloroethane; (ii) Pd(MeCN)_4_(BF_4_)_2_, **20**, AgBF_4_, PivOH, 1,2-dichloroethane; (iii) Step 1: MTAD, AcOMe, white LED, then **3a**, Fe(acac)_3_, dppbz, ZnCl_2_, 1,2-dichloroisobutane, THF; Step 2: *p*-chloranil, 1,1,2,2-tetrachloroethane. (iv) Pd(MeCN)_4_(BF_4_)_2_, **5la**, AgOPiv, TfOH, 1,2-dichloroethane; (v) Step 1: MTAD, AcOMe, white LED, then **3a**, Fe(acac)_3_, dppbz, ZnCl_2_, 1,2-dichloroisobutane, THF; Step 2: *p*-chloranil, 1,1,2,2-tetrachloroethane; (vi) Pd(MeCN)_4_(BF_4_)_2_, **21**, AgBF_4_; (vii) Pd(MeCN)_4_(BF_4_)_2_, **21**, AgOPiv, TfOH, 1,2-dichloroethane; (viii) Step 1: MTAD, acetone, white LED; Step 2: **3e′**, Fe(acac)_3_, dppbz, 1,2-dichloroisobutane, THF; Step 3: *p*-chloranil, 1,1,2,2-tetrachloroethane; (ix) Pd(MeCN)_4_(BF_4_)_2_, **21**, AgOPiv, TfOH, 1,2-dichloroethane; (x) **22**, CsF, THF/MeCN; (xi) Pd(MeCN)_4_(BF_4_)_2_, **21**, AgOPiv, TfOH, 1,2-dichloroethane. Piv, pivaloyl; Tf, trifluoromethanesulfonyl. Gray hexagons represent newly constructed hexagons created by APEX reactions.

The power of this DAPEX strategy becomes most apparent when strategically combining the present *M*-APEX with previously reported *K*- and *bay*-APEX reactions (Fig. 5). Iterative APEX reactions starting from phenanthrene are shown in Fig. 5a. *M*-APEX reaction of phenanthrene (**1h**) gave **5ha** through rearomatization of the intermediate **4ha**, as described in Fig. 2b. Conversely, palladium-catalyzed *K*-APEX reaction^17^ of phenanthrene **8** with diiodobiaryl **1l** successfully gave the corresponding π-extended PAH **9**. Further APEX reactions at the remaining peripheral regions of **5ha** and **9** are possible. For example, nanographene **10** was obtained by the *K*-APEX reaction of **5ha** with diiodobiphenyl **20**, while *M*-APEX of **9** gave nanographene **11** (see Supplementary Fig. 5 for X-ray crystal structure). Nanographene **11** can also be obtained directly from phenanthrene **8** by employing the pre-π-extended diiodobiaryl **5la** as the π-extending agent for *K*-APEX. In addition to newly formed PAHs, APEX intermediates such as **4ha** can also be employed as templates for further π-extension. Indeed, dearomatization and annulative diarylation of **4ha** gave the corresponding tetra-arylated product **6ha** (see section 1.9 of Supplementary Information and Supplementary Fig. 6). Rearomatization with *p*-chloranil proceeded to remove both of the urazole moieties and afford the double *M*-APEX product **12**. APEX reaction at the remaining *K*-region of **12** also proceeded to give nanographene **13** (see Supplementary Fig. 7 for X-ray crystal structure). It should be noted that for the APEX reactions of less-soluble compounds such as **12**, a mechanochemical reaction setup with ball-milling^32^ is advantageous (see section 1.12 of Supplementary Information).

We further verified the utility of DAPEX strategy in the diversity-oriented synthesis of nanographenes. We previously clarified that the *K*-APEX reaction of chrysene (**1k**) with 2,2′-diiodobiphenyl (**20**) gives the highly fused nanographene **14** in one step^17^, while the present *M*-APEX reaction of **1k** with **3a** gave the elongated product **5ka** (Fig. 5b). The π-extended chrysene **5ka** also worked as a new template for late-stage *K*-APEX, successfully affording nanographene **15** in 42% yield (see Supplementary Fig. 8 for X-ray crystal structure). Another nanographene **16** could be synthesized starting from naphthalene (**1g**) through the sequence of *M*-APEX and *K*-APEX (Fig. 5c). The *M*-APEX of **1g** with 2-(1-naphthyl)-phenyl magnesium bromide (**3e′**) gave the π-extended PAH **5ge**, bearing a newly-formed *K*-region. *K*-APEX reaction on this *K*-region also proceeded successfully to afford the nanographene **16** (see Supplementary Fig. 9 for X-ray crystal structure). As another related example for growth-from-template synthesis, we conducted the *bay*-APEX reaction of perylene with π-extending agent **22** to form naphthoperylele **18**^21^, which was then subjected to *K*-APEX to access nanographene **19** (Fig. 5d).

In conclusion, we have established a concept of DAPEX that involves the dearomative activation of polyaromatic templates, annulative diarylation, and rearomatization, and realized a formal APEX reaction at the less-reactive *M*-region of aromatic templates. In addition, an intuitive, diversity-oriented synthesis of nanographenes has been achieved by combining the developed DAPEX and previous APEX reactions of PAHs. As many properties of nanographenes are not easily predicted, this powerful strategy capable of generating vast structural diversity in a programmable fashion will allow for an increased understanding of nanographene structure-property relationships. This will in turn aid in the discovery of hitherto unknown functional molecules and new guiding principles for the future rational property-driven design of nanographenes. The present study speaks well for the potential of regiodivergent PAH functionalization in the creation of nanographene libraries to confront the most significant challenges in materials science.

## Methods

See Supplementary Information for detailed methods and characterization data.

### The typical procedure for dearomatization of polyaromatics **1** followed by annulative diarylation

To a glass tube containing a magnetic stirring bar were added MTAD (0.10 mmol, 1.0 equiv) and polyaromatics **1** (0.20–1.0 mmol, 2.0–10 equiv). The tube was sealed with a septum, filled with N_2_ gas, and then methyl acetate (20 mL) was added at ambient temperature. The contents were sonicated to dissolve solids as much as possible and then cooled to 0 °C. The resulting pink solution was stirred under irradiation with LED lights at 0 °C until the solution became colorless or brown (approx. for 2 h). After turning off the lights, the mixture was transferred to a 50 mL two-necked round-bottomed flask, and the volatile was removed in vacuo at 0 °C. Then, the flask was filled with N_2_ gas, and THF (2.5 mL) was added to dissolve the residue (solution A).

To another glass tube containing a magnetic stirring bar was added dppbz (0.01 mmol, 10 mol%). The tube was sealed with a septum and filled with N_2_ gas. Then THF (1.0 mL), 1.9 M ZnCl_2_ solution in 2-methyltetrahydrofuran (0.30 mmol, 3.0 equiv) and THF solution of **3a** (0.30 mmol, 3.0 equiv) were added in this order at ambient temperature. Another portion of 1.9 M ZnCl_2_ solution in 2-methyltetrahydrofuran (0.30 mmol, 3.0 equiv) was added and then white precipitation was formed. This mixture was stirred at ambient temperature for 30 min and then cooled to 0 °C. At this temperature, 0.20 M Fe(acac)_3_ solution in THF (0.01 mmol, 10 mol%) was added, and the resulting mixture was stirred for 5 min. Then, 1,2-dichloroisobutane (0.15 mmol, 1.5 equiv) and solution A were added to this tube, and the mixture was stirred at ambient temperature for 2 h. The mixture was quenched with 1 M HCl aq. (approx. 10 mL) and extracted with dichloromethane (3 × 30 mL). The combined organic layers were dried with Na_2_SO_4_, filtered, and concentrated under reduced pressure. The crude product was purified by PTLC to yield diarylated product **4**.

### The typical procedure for rearomatization of diarylated compound **4**

To a screw-capped tube containing a magnetic stirring bar were added diarylated compound **4** (1.0 equiv) and *p*-chloranil (3.0 equiv). Then, 1,1,2,2-tetrachloroethane was added to this tube to prepare a 0.1 M solution of **4** under air. The tube was sealed with a cap, and the resulting mixture was stirred at 150 °C for 36 h. Then, the reaction mixture was cooled to ambient temperature and diluted with chloroform (approx. 3 mL). To this mixture, hydrazine monohydrate (5.0 equiv) was added and the resulting mixture was stirred at ambient temperature for 15 min to quench the remaining *p*-chloranil. The mixture was washed with 1 M NaOH aq. (approx. 10 mL) and extracted with chloroform (3 × 30 mL). The combined organic layers were dried with Na_2_SO_4_, filtered, and concentrated under reduced pressure. The crude product was purified by flash column chromatography on silica gel to yield π-extended product **5**.

## Supplementary information

Supplementary Information

Description of Additional Supplementary Files

Supplementary Dataset 1

## Data Availability

All the characterization data and experimental protocols are provided in this article and its Supplementary Information. Cartesian coordinates for all calculated geometries are provided in Supplementary Data 1. The X-ray crystallographic data for compounds **4aa**, **4pa**, **6ba**, **6ha**, **11**, **13**, **15**, and **16** have been deposited at the Cambridge Crystallographic Data Center (CCDC), under deposition numbers 2012769–2012775 and 2071523. These data can be obtained free of charge from CCDC via www.ccdc.cam.ac.uk/data_request/cif.
