# Supplementary material for: Diversity-oriented synthesis of nanographenes enabled by dearomative annulative π-extension
Source: Nat Commun. 2021 Jun 24;12:3940. doi: 10.1038/s41467-021-24261-y (PMC8225822; doi:10.1038/s41467-021-24261-y)
Supplement: Supplementary file 1 — Supplementary Information [file 41467_2021_24261_MOESM1_ESM.pdf]

Supplementary Information

Diversity-oriented synthesis of nanographenes enabled by dearomative annulative π-extension

Wataru Matsuoka,[1] Hideto Ito,[1,2,*] David Sarlah[3,*] & Kenichiro Itami[1,2,4,*]

[1] Graduate School of Science, Nagoya University, Chikusa, Nagoya 464-8602, Japan.
[2] JST-ERATO, Itami Molecular Nanocarbon Project, Chikusa, Nagoya 464-8602, Japan.
[3] Department of Chemistry,University of Illinois, Urbana, Illinois 61801, United States
[4] Institute of Transformative Bio-Molecules (WPI-ITbM), Nagoya University, Chikusa, Nagoya 464-8602, Japan.

Table of Contents

1. Supplementary Methods
- 1.1 General S2
- 1.2 Synthesis of substrates and reagents S3–S4
- 1.3 Preparation of the Grignard reagents S5
- 1.4 Annulative diarylation of **2a** S6
- 1.5 Dearomatization of polyaromatics followed by annulative diarylation S7–S16
- 1.6 Annulative diarylation of cycloadduct **2g** with mono-Grignard reagents **3′** or **3″** S17–S20
- 1.7 Rearomatization of diarylated compounds S21–S31
- 1.8 Synthesis of 10,10'-bibenzo[*f*]tetraphene (**7ba**) S32–S34
- 1.9 Synthesis of tetrabenzo[*a,c,m,o*]pentaphene (**12**) S35–S36
- 1.10 Synthesis of 2,7-di-tert-butyldibenzo[*j,s*]phenanthro[9,10-*b*]picene (**11**) S37–S38
- 1.11 Regioselectivity and limitation S39–S42
- 1.12 Synthesis of nanographenes by *K*- and *bay*-APEX reactions S43–S48
- 1.13 X-ray crystallographic analysis S49–S55
- 1.14 Computational study S56–S68
- 1.15 Photophysical properties S60
- 1.16 Assignment of ^{1}H NMR signals S61
- 1.17 ^{1}H and ^{13}C NMR spectra S62–S311

2. Supplementary References S312–S313

1. Supplementary Methods

1.1 General

Unless otherwise noted, all reactants or reagents including dry solvents were obtained from commercial suppliers and used as received. $Fe(acac)_3$ was purchased from STREM Chemicals, INC. $ZnCl_2$ (1.9 M, 2-methyltetrahydrofuran solution), dppbz, 1,2-dichloroisobutane, **1a**, **1b**, **1c**, **1e**, **1g**, **1h**, **1k**, **1o**, **1r**, **17**, **22**, 2,2′-dibromobiphenyl, 2-bromobiphenyl, triflic acid and pivalic acid were purchased from Tokyo Chemical Industry Co., Ltd. *p*-Chloranil, **1d**, cesium fluoride, methyl acetate and 1,1,2,2-tetrachloroethane, *N*-bromosuccinimide were purchased from Wako Pure Chemical Industries, Ltd. **1f**, tetrahydrofuran and acetonitrile were purchased from Kanto Chemical Co., Inc. $Pd(MeCN)_4(BF_4)_2$, silver tetrafluoroborate and 1,2-dichloroethane were purchased from Sigma-Aldrich. Benzoyl peroxide was purchased from Nacalai tesque. MTAD[1,2], ^{t}BuTAD[1,2], **1f**[3], **1i**[4], **1j**[5], **1l**[6], **1m**[7], **1n**[8], **1p**[9], aryl bromides[10–12] for Grignard reagents (**3b″**, **3c′**, **3d′**, **3e′**, **3f″** and **3g′**), **20**[13], **21**[14] and silver pivalate[15] were synthesized according to the reported procedure. Unless otherwise noted, all reactions were performed with dry solvents under an atmosphere of N_2 gas in dried glassware using standard vacuum-line techniques. Eleven white light LED corn bulbs (4W) were used for the photochemical experiments. All work-up and purification procedures were carried out with reagent-grade solvents in air. Analytical thin-layer chromatography (TLC) was performed using E. Merck silica gel 60 F254 precoated plates (0.25 mm). The developed chromatogram was analyzed by UV lamp (254 nm or 365 nm). Flash column chromatography was performed with E. Merck silica gel 60 (230–400 mesh). Silica-gel column chromatography was performed on an Isolera Spektra instrument equipped with a Biotage SNAP Ultra 50 g. Preparative recycling gel permeation chromatography (GPC) was performed with a JAI LC-9260 II NEXT instrument equipped with JAIGEL-2HR columns using chloroform as an eluent. Nuclear magnetic resonance (NMR) spectra were recorded on a JEOL JNM-ECA-600 (^{1}H 600 MHz, ^{13}C 150 MHz) spectrometer. Chemical shifts for ^{1}H NMR are expressed in parts per million (ppm) relative to tetramethylsilane (δ 0.0 ppm), CD_2Cl_2 (δ 5.32 ppm), $Cl_2CDCDCl_2$ (δ 5.94 ppm) or acetone-d_6 (δ 2.04 ppm). Chemical shifts for ^{13}C NMR are expressed in ppm relative to $CDCl_3$ (δ 77.0 ppm), CD_2Cl_2 (δ 53.8 ppm), $Cl_2CDCDCl_2$ (δ 73.78 ppm) or acetone-d_6 (δ 29.8 ppm). Data are reported as follows: chemical shift, multiplicity (s = singlet, d = doublet, dd = doublet of doublets, t = triplet, dt = doublet of triplets, td = triplet of doublets, q = quartet, m = multiplet, bs = broad singlet), coupling constant (Hz), and integration.

1.2 Synthesis of substrates and reagents

Synthesis of **2a**

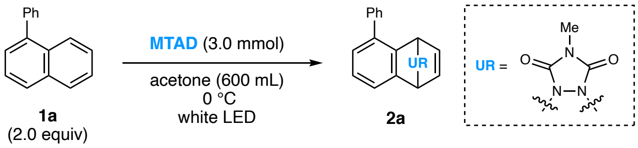

MTAD (339 mg, 3.0 mmol, 1.0 equiv) and 1-phenylnaphthalene (1.22 g, 6.0 mmol, 2.0 equiv) were placed in a 1-L round-bottomed flask. Acetone (600 mL) was added to the flask at ambient temperature, and the mixture was stirred until MTAD was completely dissolved. Then the resulting pink solution was stirred under irradiation with LED lights at 0 °C until the solution became colourless. The reaction mixture was concentrated under reduced pressure with ice bath, when most of the solvents were removed, the crude product was dissolved into small amount of ethyl acetate and diethyl ether (about 1:1 ratio). Then, excess amount of pentane was added and the precipitate was collected by filtration and washed with pentane for 3 times. The product was dried under high vacuum. The cycloadduct **2a** was obtained as a white solid (686.5 mg, 72%).

^{1}H NMR (600 MHz, acetone-d_6) δ 7.50–7.55 (m, 3H), 7.44–7.48 (m, 3H), 7.35 (t, J = 7.5 Hz, 1H), 7.29 (d, J = 7.5 Hz, 1H), 6.92–6.98 (m, 2H), 6.01 (dd, J = 5.1, 1.5 Hz, 1H), 5.98 (dd, J = 4.8, 2.4 Hz, 1H), 2.86 (s, 3H)

^{13}C NMR (150 MHz, acetone-d_6) δ 159.10, 158.96, 139.47, 139.26, 138.53, 135.98, 134.69, 134.27, 129.97, 129.66, 129.23, 128.62, 128.20, 123.96, 57.85, 55.32, 25.57

HRMS (ESI, positive): Calcd for $C_{19}H_{15}N_3O_2Na$ ([M+Na]$^+$): 340.1056. Found: 340.1055.

Synthesis of **2g**

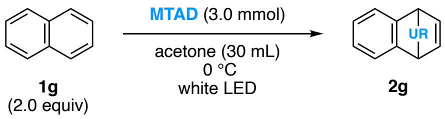

MTAD (339 mg, 3.0 mmol, 1.0 equiv) and naphthalene (768 mg, 6.0 mmol, 2.0 equiv) were placed in a 100-mL round-bottomed flask. Acetone (30 mL) was added to the flask at ambient temperature, and the mixture was stirred until MTAD was completely dissolved. Then the resulting pink solution was stirred under irradiation with LED lights at 0 °C until the solution became colourless. The reaction mixture was concentrated under reduced pressure with ice bath. When most of the solvents were removed, excess amount of pentane was added and the precipitate was collected by filtration and washed with pentane for 3 times. The product was dried under high vacuum. The cycloadduct **2g** was obtained as a white solid (551.5 mg, 76%).

^{1}H NMR (600 MHz, acetone-d_6) δ 7.46 (dd, J = 5.1, 3.3 Hz, 2H), 7.26 (dd, J = 5.4, 3.6 Hz, 2H), 6.89 (t, J = 3.3 Hz, 2H), 5.89 (t, J = 3.3 Hz, 2H), 2.84 (s, 3H)

^{13}C NMR (600 MHz, acetone-d_6) δ 159.16, 138.63, 134.38, 128.21, 124.52, 57.82, 25.49

HRMS (ESI, positive): Calcd for $C_{13}H_{12}N_3O_2$ ([M+H]$^+$): 242.9024. Found: 242.9025.

Synthesis of **1q**

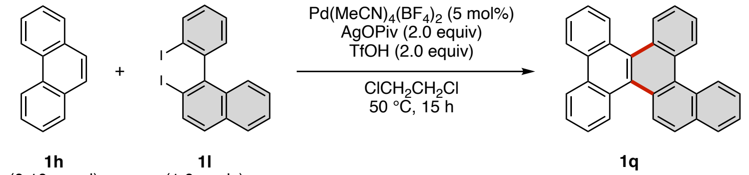

To a Schlenk tube containing a magnetic stirring bar were added phenanthrene (**1h**) (17.8 mg, 0.10 mmol, 1.0 equiv), diiodobiaryl **1l** (45.6 mg, 0.10 mmol, 1.0 equiv) and AgOPiv (41.8 mg, 0.20 mmol, 2.0 equiv). The tube was taken into a glove box. Then, $Pd(MeCN)_4(BF_4)_2$ (2.2 mg, 0.005 mmol, 5 mol%) was added, and the tube was sealed with a septum. The tube was taken out from the glove box, and then 1,2-dichloroethane (1.0 mL) and TfOH (17.8 µL, 0.20 mmol, 2.0 equiv) were added by a syringe. After stirring at 50 °C for 15 h, the reaction mixture was cooled to ambient temperature, and then passed through a short pad of silica gel (eluent: $CHCl_3$). After the organic solvent was removed under reduced pressure, the residue was purified by flash column chromatography on silica gel (eluent: hexane/$CHCl_3$ = 10:1 to 5:1) to yield desired product **1q** (24.5 mg, 65%) as a white solid.

^{1}H NMR (600 MHz, $CDCl_3$) δ 9.14 (d, J = 7.8 Hz, 1H), 8.96 (dd, J = 7.2, 1.8 Hz, 1H), 8.68–8.79 (m, 6H), 8.02 (d, J = 9.0 Hz, 2H), 7.59–7.74 (m, 8H)

^{13}C NMR (150 MHz, $CDCl_3$) δ 132.97, 131.16, 131.01, 130.35, 130.00, 129.69, 129.24, 129.12, 129.01, 128.83, 128.78, 128.43, 128.05, 127.92, 127.90, 127.85, 127.64, 127.42, 127.37, 126.80, 126.74, 126.62, 126.45, 126.39, 126.32, 126.06, 125.66, 125.63, 123.73, 123.55

HRMS (APCI, positive): Calcd for $C_{30}H_{19}$ ([M+H]$^+$): 379.1481. Found: 379.1476.

1.3 Preparation of the Grignard reagents

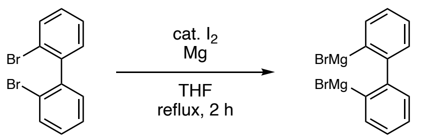

3a

A 50-mL Schlenk tube containing Mg powder (365 mg, 15 mmol, 3.0 equiv) and magnetic stirring bar was heated by heat gun for 5 min, and stirred for about 20 min. A small piece of I_2 was dissolved into THF (4.0 mL) and this solution was added to the Schlenk tube. The mixture was stirred at 65 °C until the mixture became colourless suspension. Then, to the resulting suspension, 2,2′-dibromobiphenyl (1.56 g, 5.0 mmol, 1.0 equiv) dissolved in THF (8.0 mL) was slowly added at this temperature. After addition, the reaction mixture was stirred at this temperature for 2 h to give the Grignard reagent as a white suspension (about 0.30 M, which was determined by titration with THF solution of I_2).

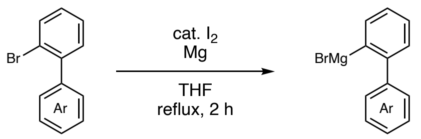

3′

A 5-mL Schlenk tube containing Mg powder (36.5 mg, 1.5 mmol, 1.5 equiv) and magnetic stirring bar was heated by heat gun for 10 min, and stirred for about 20 min. A small piece of I_2 was dissolved into THF (0.80 mL) and this solution was added to the Schlenk tube. The mixture was stirred at 65 °C until the mixture became colourless suspension. Then, to the resulting suspension, aryl bromide (1.0 mmol, 1.0 equiv) dissolved (or suspended) in THF (1.6 mL) was slowly added at this temperature. After addition, the reaction mixture was stirred at this temperature for 2 h to give the Grignard reagent (about 0.30 M, which was determined by titration with THF solution of I_2).

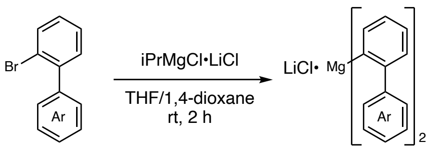

3″

To a 5 mL Schlenk tube containing a magnetic stirring bar was added aryl bromide (1.25 mmol, 1.0 equiv) under a stream of nitrogen. The tube was sealed with septum and THF (1 mL), ^{i}PrMgCl·LiCl (1 M THF solution, 1.25 mL), 1,4-dioxane (130 µL) was added in this order at ambient temperature. The resulting solution were stirred at ambient temperature for 2 h to give the Grignard reagent. The generation of Grignard reagent was monitored by GC-MS, and the Grignard reagents was used as 0.5 M solution.

1.4 Annulative diarylation of 2a

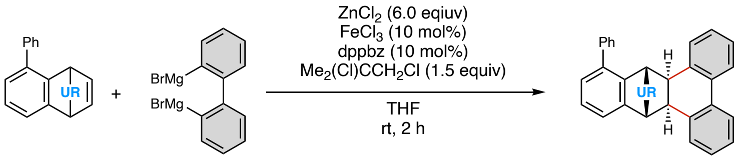

To a test tube containing a magnetic stirring bar was added 1,2-bis(diphenylphosphino)benzene (4.5 mg, dppbz, 0.01 mmol, 10 mol%). The tube was sealed with a septum and filled with N_2 gas. Then THF (1.0 mL), 1.9 M $ZnCl_2$ solution in 2-methyltetrahydrofuran (158 μL, 0.30 mmol, 3.0 equiv) and THF solution of **3a** (0.30 mmol, 3.0 equiv) were added in this order at ambient temperature. Another portion of 1.9 M $ZnCl_2$ solution in 2-methyltetrahydrofuran (158 μL, 0.30 mmol, 3.0 equiv) was added and then white precipitation formed. This mixture was stirred at ambient temperature for 30 min, and then cooled to 0 °C. At this temperature, 0.20 M $Fe(acac)_3$ solution in THF (50 μL, 0.01 mmol, 10 mol%) was added and resulting mixture was stirred for 5 min. Then, 1,2-dichloroisobutane (17.5 μL, 0.15 mmol, 1.5 equiv) and **2a** (31.7 mg, 0.10 mmol, 1.0 equiv, dissolved in 2.5 mL THF) was added to this tube, and the mixture was stirred at ambient temperature for 2 h. The mixture was quenched with 1 M HCl aq. (approx. 10 mL) and extracted with dichloromethane (3×30 mL). The combined organic layers were dried with Na_2SO_4, filtered and concentrated under reduced pressure. The crude product was purified by PTLC (eluent: $CHCl_3$) to yield **4aa** as a white solid (41.3 mg, 88%).

^{1}H NMR (600 MHz, CD_2Cl_2) δ 7.98 (dd, J = 11.1, 7.5 Hz, 2H), 7.46–7.63 (m, 9H), 7.41 (td, J = 7.5, 1.2 Hz, 1H), 7.37 (td, J = 7.2, 1.2 Hz, 1H), 7.26–7.34 (m, 2H), 7.20 (d, J = 7.2 Hz, 1H), 5.64 (s, 1H), 5.49 (s, 1H), 3.73 (s, 2H), 2.64 (s, 3H)

^{13}C NMR (150 MHz, CD_2Cl_2) δ 157.63, 157.20, 138.71, 138.50, 135.78, 133.12, 133.06, 132.92, 132.35, 132.26, 130.50, 129.68, 129.49, 129.30, 129.21, 129.07, 128.90, 128.34, 128.19, 128.13, 123.20, 123.15, 62.34, 59.18, 38.04, 37.88, 25.30 (two sp^2 carbon signals were overlapping with others)

HRMS (ESI, positive): Calcd for $C_{31}H_{23}N_3O_2Na$ ($[M+Na]^+$): 492.1682. Found: 492.1687.

1.5 Dearomatization of polyaromatics followed by annulative diarylation

Procedure A

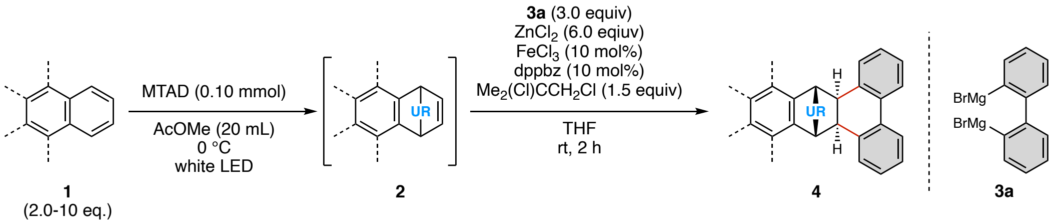

To a test tube containing a magnetic stirring bar were added MTAD (0.10 mmol, 1.0 equiv) and polyaromatics **1** (0.20–1.0 mmol, 2.0–10 equiv). The tube was sealed with a septum, filled with N_2 gas, and then methyl acetate (20 mL) was added at ambient temperature. The contents were sonicated to dissolve solids as much as possible, and then cooled to 0 °C. The resulting pink solution was stirred under irradiation with LED lights at 0 °C until the solution became colourless or brown (approx. for 2 h). After turning off the lights, the mixture was transferred to a 50-mL two-necked round-bottomed flask, and the volatile was removed in vacuo at 0 °C. Then, the flask was filled with N_2 gas, and THF (2.5 mL) were added to dissolve the residue (**solution A**).

To another test tube containing a magnetic stirring bar was added 1,2-bis(diphenylphosphino)benzene (dppbz, 0.01 mmol, 10 mol%). The tube was sealed with a septum and filled with N_2 gas. Then THF (1.0 mL), 1.9 M $ZnCl_2$ solution in 2-methyltetrahydrofuran (0.30 mmol, 3.0 equiv) and THF solution of **3a** (0.30 mmol, 3.0 equiv) were added in this order at ambient temperature. Another portion of 1.9 M $ZnCl_2$ solution in 2-methyltetrahydrofuran (0.30 mmol, 3.0 equiv) was added and then white precipitation was formed. This mixture was stirred at ambient temperature for 30 min, and then cooled to 0 °C. At this temperature, 0.20 M $Fe(acac)_3$ solution in THF (0.01 mmol, 10 mol%) was added and resulting mixture was stirred for 5 min. Then, 1,2-dichloroisobutane (0.15 mmol, 1.5 equiv) and **solution A** was added to this tube, and the mixture was stirred at ambient temperature for 2 h. The mixture was quenched with 1 M HCl aq. (approx. 10 mL) and extracted with dichloromethane (3×30 mL). The combined organic layers were dried with Na_2SO_4, filtered and concentrated under reduced pressure. The crude product was purified by PTLC to yield diarylated product **4**.

Procedure B

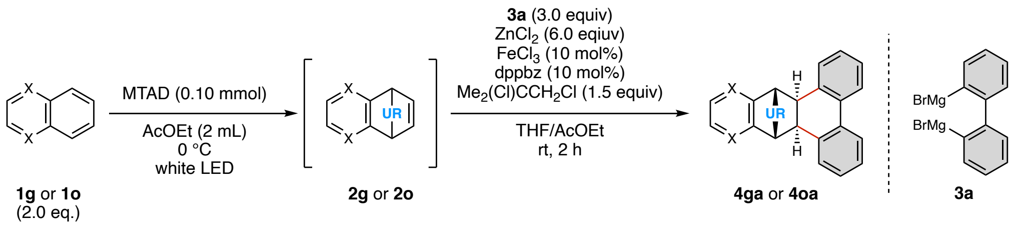

To a test tube containing a magnetic stirring bar were added MTAD (0.10 mmol, 1.0 equiv) and polyaromatics **1** (0.20 mmol, 2.0 equiv). The tube was sealed with a septum, filled with N_2 gas, and then ethyl acetate (2 mL) was added at ambient temperature. The contents were sonicated to dissolve solids, and then cooled to 0 °C. The resulting pink solution was stirred under irradiation with LED lights at 0 °C until the solution became colourless or brown (approx. for 2 h). (**solution A**).

To another test tube containing a magnetic stirring bar was added dppbz (0.01 mmol, 10 mol%). The tube was sealed with a septum and filled with N_2 gas. Then, THF (1.0 mL), 1.9 M $ZnCl_2$ solution in 2-methyltetrahydrofuran (0.30 mmol, 3.0 equiv) and THF solution of **3a** (0.30 mmol, 3.0 equiv) were added in this order at ambient temperature. Another portion of 1.9 M $ZnCl_2$ solution in 2-methyltetrahydrofuran (0.30 mmol, 3.0 equiv) was added and then white precipitation was immediately formed. This mixture was stirred at ambient temperature for 30 min, and then cooled to 0 °C. At this temperature, 0.20 M $Fe(acac)_3$ in THF (0.01 mmol, 10 mol%) was added and resulting mixture was stirred for 5 min. Then, 1,2-dichloroisobutane (0.15 mmol, 1.5 equiv) was added to this tube, and the mixture was added to **solution A** by a syringe. The mixture was stirred at ambient temperature for 2 h. After completion of the reaction, the mixture was quenched with 1 M HCl aq. (approx. 10 mL) and extracted with dichloromethane (3×30 mL). The combined organic layers were dried with Na_2SO_4, filtered and concentrated under reduced pressure. The crude product was purified by PTLC to yield diarylated product **4**.

18-Methyl-10-phenyl-8b,9,14,14a-tetrahydro-9,14-[1,2]epitriazolobenzo[*f*]tetraphene-17,19-dione (**4aa**)

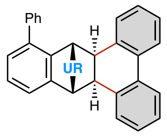

Method: Procedure A, 2.0 eq. of 1-phenylnaphthalene (**1a**) was used. Relative configuration was confirmed by X-ray crystallographic analysis (Supplementary Fig. 1)

Purification: PTLC ($CHCl_3$)

Yield: 28.0 mg, 60%, white solid

The NMR spectra of product were identical to those described above.

18-Methyl-10-(naphthalen-1-yl)-8b,9,14,14a-tetrahydro-9,14-[1,2]epitriazolobenzo[*f*]tetraphene-17,19-dione (**4ba**)

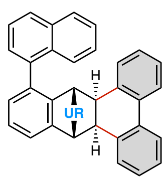

Method: Procedure A, 5.0 eq. of 1,1′-binaphthyl (**1b**) was used.

Purification: PTLC ($CHCl_3$), The product was obtained as a mixture of rotational isomers (major/minor = 78/22).

Yield: 36.9 mg, 71%, white solid

^{1}H NMR (600 MHz, $Cl_2CDCDCl_2$) δ 7.98–8.04 (m, 0.78×2H, 0.22×1H), 7.95 (d, *J* = 7.8 Hz, 0.22×1H), 7.89 (d, *J* = 7.8 Hz, 0.22×1H), 7.83–7.87 (m, 1H), 7.79 (d, *J* = 7.2 Hz, 0.78×1H), 7.61–7.67 (m, 1H), 7.41–7.58 (m, 0.78×8H, 0.22×6H), 7.27–7.38 (m, 0.78×2H, 0.22×4H), 7.23 (t, *J* = 7.8 Hz, 0.22×1H), 7.11–7.18 (m, 1H), 7.05 (d, *J* = 7.8 Hz, 0.22×1H), 6.92 (t, *J* = 7.2 Hz, 0.78×1H), 6.17 (d, *J* = 7.2 Hz, 0.78×1H), 5.50 (d, *J* = 1.8 Hz, 1H), 5.19 (d, *J* = 1.2 Hz, 0.22×1H), 4.85 (d, *J* = 2.4 Hz, 0.78×1H), 3.74–3.80 (m, 0.78×1H, 0.22×2H), 3.56 (dd, *J* = 12.0, 1.8 Hz, 0.78×1H), 2.62 (s, 0.78×3H), 2.56 (s, 0.22×3H)

^{13}C NMR (150 MHz, $Cl_2CDCDCl_2$) δ 156.76, 156.53, 156.19, 155.57, 136.09, 136.05, 135.56, 135.31, 135.17, 134.77, 134.11, 134.02, 133.77, 133.28, 132.22, 132.12, 132.01, 131.99, 131.77, 131.49, 131.44, 131.16, 130.71, 129.28, 129.13, 129.06, 128.85, 128.74, 128.60, 128.53, 128.48, 128.34, 128.21, 128.18, 127.99, 127.83, 127.76, 127.72, 127.49, 126.55, 126.51, 126.31, 126.28, 125.97, 125.93, 125.61, 125.08, 124.70, 122.99, 122.82, 122.72, 122.57, 122.46, 61.43, 61.36, 59.02, 58.79, 38.02, 37.64, 37.53, 37.20, 25.12, 24.92 (seven sp^2 carbon signals were overlapping with others)

HRMS (ESI, positive): Calcd for $C_{35}H_{25}N_3O_2Na$ ($[M+Na]^+$): 542.1839. Found: 542.1846.

10-Bromo-18-methyl-8b,9,14,14a-tetrahydro-9,14-[1,2]epitriazolobenzo[*f*]tetraphene-17,19-dione (**4ca**)

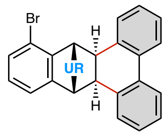

Method: Procedure A, 2.0 eq. of 1-bromonaphthalene (**1c**) was used.

Purification: PTLC ($CHCl_3$)

Yield: 22.8 mg, 48%, white solid

^{1}H NMR (600 MHz, CD_2Cl_2) δ 8.00 (dd, *J* = 7.2, 2.4 Hz, 2H), 7.66 (d, *J* = 8.4 Hz, 1H), 7.55 (d, *J* = 7.8 Hz, 1H), 7.52 (dd, *J* = 7.8, 1.2 Hz, 1H), 7.32–7.47 (m, 6H), 5.84 (s, 1H), 5.44 (s, 1H), 3.67 (t, *J* = 13.5 Hz, 2H), 2.64 (s, 3H)

^{13}C NMR (150 MHz, CD_2Cl_2) δ 157.57, 157.50, 137.26, 135.28, 132.91, 132.62, 132.510, 132.36, 132.30, 130.84, 129.78, 129.53, 129.04, 128.93, 128.33, 128.29, 123.38, 123.29, 123.23, 119.20, 61.91, 61.33, 37.54,

37.40, 25.35

HRMS (ESI, positive): Calcd for $C_{25}H_{18}N_3O_2BrNa$ ($[M+Na]^+$): 474.1424. Found: 474.1426.

10-Iodo-18-methyl-8b,9,14,14a-tetrahydro-9,14-[1,2]epitriazolobenzo[*f*]tetraphene-17,19-dione (**4da**)

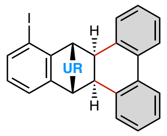

Method: Procedure A, 2.0 eq. of 1-iodonaphthalene (**1d**) was used.

Purification: PTLC ($CHCl_3$)

Yield: 30.1 mg, 58%, white solid

^{1}H NMR (600 MHz, $CDCl_3$) δ 7.94–7.99 (m, 2H), 7.87 (d, J = 8.4 Hz, 1H), 7.54 (d, J = 7.8 Hz, 1H), 7.31–7.49 (m, 6H), 7.17 (t, J = 7.5 Hz, 1H), 5.78 (s, 1H), 5.43 (s, 1H), 3.63 (s, 2H), 2.69 (s, 3H)

^{13}C NMR (150 MHz, $CDCl_3$) δ 157.10, 156.94, 139.04, 138.82, 136.47, 132.03, 131.86, 131.77, 131.65, 130.74, 129.46, 129.17, 128.82, 128.63, 128.03, 128.01, 123.75, 122.98, 122.88, 93.67, 65.33, 61.77, 37.29, 37.24, 25.22

HRMS (ESI, positive): Calcd for $C_{25}H_{18}N_3O_2INa$ ($[M+Na]^+$): 534.0336. Found: 534.0336.

18-Methyl-17,19-dioxo-8b,9,14,14a-tetrahydro-9,14-[1,2]epitriazolobenzo[*f*]tetraphen-10-yl acetate (**4ea**)

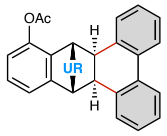

Method: Procedure A, 2.0 eq. of naphthalen-1-yl acetate (**1e**) was used.

Purification: PTLC ($CHCl_3$/Ethyl acetate = 4:1)

Yield: 26.8 mg, 59%, white solid

^{1}H NMR (600 MHz, $CDCl_3$) δ 7.93–7.97 (m, 2H), 7.49 (dd, J = 7.2, 1.2 Hz, 1H), 7.45 (t, J = 8.1 Hz, 1H), 7.30–7.39 (m, 6H), 7.17 (d, J = 7.8 Hz, 1H), 5.54 (d, J = 1.8 Hz, 1H), 5.37 (d, J = 2.4 Hz, 1H), 3.75 (dd, J = 12.0, 1.8 Hz, 1H), 3.72 (dd, J = 12.0, 2.4 Hz, 1H), 2.67 (s, 3H), 2.51 (s, 3H)

^{13}C NMR (150 MHz, $CDCl_3$) δ 169.63, 157.06, 156.87, 144.55, 136.43, 132.18, 132.15, 132.02, 131.88, 129.88, 129.44, 129.09, 128.66, 128.56, 127.88, 127.64, 122.88 (2C), 122.78, 122.29, 121.51, 60.93, 57.29, 37.27, 36.83, 25.12, 21.05

HRMS (ESI, positive): Calcd for $C_{27}H_{21}N_3O_4Na$ ($[M+Na]^+$): 474.1424. Found: 474.1426.

18-Methyl-17,19-dioxo-8b,9,14,14a-tetrahydro-9,14-[1,2]epitriazolobenzo[*f*]tetraphen-11-yl pivalate (**4fa**)

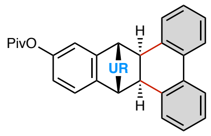

Method: Procedure A, 2.0 eq. of naphthalen-2-yl pivalate (**1f**) was used.

Purification: PTLC ($CHCl_3$/Ethyl acetate = 4:1)

Yield: 16.0 mg, 32%, white solid

^{1}H NMR (600 MHz, $CDCl_3$) δ 7.96 (d, J = 6.6 Hz, 2H), 7.48 (d, J = 7.8 Hz, 2H), 7.45 (dd, J = 6.6, 1.8 Hz, 1H), 7.31–7.38 (m, 4H), 7.24 (d, J = 2.4 Hz, 1H), 7.14 (dd, J = 8.1, 2.1 Hz, 1H), 5.49 (d, J = 3.0 Hz, 1H), 5.45 (d, J = 2.4 Hz, 1H), 3.69 (dd, J = 12.0, 1.8 Hz, 1H), 3.65 (dd, J = 11.4, 1.8 Hz, 1H), 2.68 (s, 3H), 1.38 (s, 9H)

^{13}C NMR (150 MHz, $CDCl_3$) δ 176.97, 157.13, 157.09, 151.44, 136.01, 132.18, 132.11, 132.04, 131.95, 131.90, 129.19, 128.63, 128.61, 127.91, 124.89, 122.89, 122.18, 117.46, 61.53, 61.21, 39.16, 37.74, 37.50, 27.09, 25.12 (three sp^2 carbon signals were overlapping with others)

HRMS (ESI, positive): Calcd for $C_{30}H_{27}N_3O_4Na$ ($[M+Na]^+$): 516.1894. Found: 516.1901.

18-Methyl-8b,9,14,14a-tetrahydro-9,14-[1,2]epitriazolobenzo[*f*]tetraphene-17,19-dione (**4ga**)

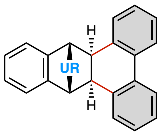

Method: Procedure B, 2.0 eq. of naphthalene (**1g**) was used.

Purification: PTLC ($CHCl_3$)

Yield: 18.9 mg, 48%, white solid

^{1}H NMR (600 MHz, $CDCl_3$) δ 7.96 (dd, J = 7.2, 2.4 Hz, 2H), 7.42–7.51 (m, 6H), 7.31–7.38 (m, 4H), 5.49 (s, 2H), 3.66 (s, 2H), 2.66 (s, 3H)

^{13}C NMR (150 MHz, $CDCl_3$) δ 157.09, 135.00, 132.29, 131.94, 129.28, 129.20, 128.59, 127.89, 123.72, 122.87, 61.69, 37.78, 25.08

HRMS (ESI, positive): Calcd for $C_{25}H_{19}N_3O_2Na$ ($[M+Na]^+$): 416.1369. Found: 416.1368.

20-Methyl-8b,9,16,16a-tetrahydro-9,16-[1,2]epitriazolodibenzo[*f*,*k*]tetraphene-19,21-dione (**4ha**)

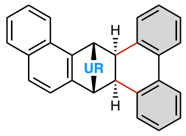

Method: Procedure A, 10 eq. of phenanthrene (**1h**) was used.

Purification: PTLC ($CHCl_3$)

Yield: 22.6 mg, 51%, white solid

^{1}H NMR (600 MHz, $CDCl_3$) δ 8.33 (d, J = 8.4 Hz, 1H), 8.00 (d, J = 7.8 Hz, 2H), 7.95 (d, J = 7.8 Hz, 2H), 7.71 (t, J = 7.8 Hz, 1H), 7.53–7.63 (m, 4H), 7.33–7.44 (m, 4H), 6.21 (d, J = 1.8 Hz, 1H), 5.67 (d, J = 3.0 Hz, 1H), 3.71 (d, J = 12.0 Hz, 1H), 3.63 (d, J = 12.6 Hz, 1H), 2.60 (s, 3H)

^{13}C NMR (150 MHz, $CDCl_3$) δ 157.61, 157.55, 133.57, 132.55, 132.49, 132.45, 132.08, 132.02, 131.30, 129.50, 129.25, 129.21, 128.94, 128.66, 128.63, 127.96, 127.88, 127.79, 127.76, 126.59, 122.95, 122.08, 121.58, 61.99, 57.80, 37.69 (2C), 25.11 (one sp^2 carbon signal was overlapping with others)

HRMS (ESI, positive): Calcd for $C_{29}H_{21}N_3O_2Na$ ([M+Na]$^+$): 466.1526. Found: 433.1527.

20-Methyl-13-phenyl-8b,9,16,16a-tetrahydro-9,16-[1,2]epitriazolodibenzo[*f*,*k*]tetraphene-19,21-dione (**4ia**)

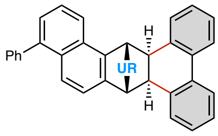

Method: Procedure A, 2.0 eq. of 1-phenylphenanthrene (**1i**) was used.

Purification: PTLC ($CHCl_3$)

Yield: 18.7 mg, 36%, white solid

^{1}H NMR (600 MHz, $CDCl_3$) δ 8.36 (d, J = 9.0 Hz, 1H), 7.98–8.03 (m, 3H), 7.75 (dd, J = 9.0, 7.2 Hz, 1H), 7.61 (d, J = 7.8 Hz, 1H), 7.45–7.58 (m, 8H), 7.43 (td, J = 7.8, 1.2 Hz, 1H), 7.33–7.40 (m, 3H), 6.27 (d, J = 2.4 Hz, 1H), 5.66 (d, J = 2.4 Hz, 1H), 3.71 (dd, J = 12.0, 2.4 Hz, 1H), 3.66 (dd, J = 12.0, 2.4 Hz, 1H), 2.62 (s, 3H)

^{13}C NMR (150 MHz, $CDCl_3$) δ 157.60 (2C), 141.41, 140.22, 132.50, 132.45, 132.43, 132.10, 132.03, 131.91, 131.48, 130.01, 129.53, 129.22, 128.68, 128.63, 128.42, 128.09, 127.99, 127.89, 127.61, 127.51, 127.22, 122.97, 122.94, 121.53, 121.50, 61.89, 58.01, 37.77, 37.68, 25.15 (one sp^2 carbon signal was overlapping with others)

HRMS (ESI, positive): Calcd for $C_{35}H_{25}N_3O_2Na$ ([M+Na]$^+$): 542.1839. Found: 542.1852.

22-Methyl-9,9a,17b,18-tetrahydro-9,18-[1,2]epitriazolotribenzo[*a*,*k*,*m*]tetraphene-21,23-dione (**4ja**)

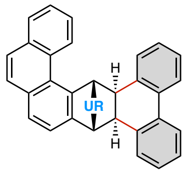

Method: Procedure A, 2.0 eq. of [4]helicene (**1j**) was used.

Purification: PTLC ($CHCl_3$)

Yield: 22.6 mg, 46%, white solid

^{1}H NMR (600 MHz, $CDCl_3$) δ 9.02 (d, J = 8.4 Hz, 1H), 7.96–8.03 (m, 3H), 7.94 (d, J = 7.2 Hz, 1H), 7.85 (td, J = 7.8, 1.4 Hz, 1H), 7.78 (d, J = 7.8 Hz, 1H), 7.70–7.74 (m, 2H), 7.67 (d, J = 7.8 Hz, 1H), 7.61 (d, J = 7.2 Hz, 1H), 7.56 (d, J = 7.8 Hz, 1H), 7.34–7.45 (m, 4H), 7.05 (d, J = 2.4 Hz, 1H), 5.65 (d, J = 3.0 Hz, 1H), 3.89 (dd, J = 12.0, 2.4 Hz, 1H), 3.73 (dd, J = 12.0, 2.4 Hz, 1H), 2.62 (s, 3H)

^{13}C NMR (150 MHz, $CDCl_3$) δ 157.31, 156.96, 134.72, 133.66, 133.60, 132.57, 132.30, 132.28, 132.15, 132.03, 129.49, 129.43, 129.36, 129.05, 128.95, 128.84, 128.67, 127.97, 127.93, 127.31, 127.11, 126.99, 126.60, 126.46, 123.05, 122.94, 122.38, 62.36, 60.23, 37.76, 37.37, 25.10 (one sp^2 carbon signal was overlapping with others)

HRMS (ESI, positive): Calcd for $C_{33}H_{23}N_3O_2Na$ ([M+Na]$^+$): 516.1682. Found: 516.1683.

22-Methyl-7,7a,15b,16-tetrahydro-7,16-[1,2]epitriazolotribenzo[*c*,*k*,*m*]tetraphene-21,23-dione (**4ka**)

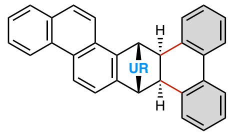

Method: Procedure A, 5.0 eq. of chrysene (**1k**) was used.

Purification: flash column chromatography ($CHCl_3$), then PTLC ($CHCl_3$)

Yield: 17.0 mg, 34%, white solid

^{1}H NMR (600 MHz, $CDCl_3$) δ 8.82 (d, J = 8.4 Hz, 1H), 8.72 (d, J = 8.4 Hz, 1H), 8.24 (d, J = 8.4 Hz, 1H), 8.01 (d, J = 7.8 Hz, 2H), 7.94–7.98 (m, 2H), 7.77 (d, J = 8.4 Hz, 1H), 7.72 (t, J = 7.2 Hz, 1H), 7.67 (t, J = 7.2 Hz, 1H), 7.62 (d, J = 7.2 Hz, 1H), 7.58 (d, J = 7.2 Hz, 1H), 7.34–7.46 (m, 4H), 6.28 (d, J = 3.0 Hz, 1H), 5.71 (d, J = 3.0 Hz, 1H), 3.76 (dd, J = 12.3, 2.7 Hz, 1H), 3.69 (dd, J = 12.3, 2.7 Hz, 1H), 2.59 (s, 3H)

^{13}C NMR (150 MHz, $CDCl_3$) δ 157.61, 157.59, 133.08, 132.48, 132.41, 132.10, 132.02, 131.80, 130.78, 130.24, 129.54, 129.21, 129.19, 128.86, 128.68, 128.66, 128.00, 127.90, 127.38, 127.27, 126.36, 123.55, 122.97, 122.95, 122.75, 121.87, 120.20, 61.91, 58.11, 37.82, 37.58, 25.10 (one sp^2 carbon signal was overlapping with others)

HRMS (ESI, positive): Calcd for $C_{33}H_{23}N_3O_2Na$ ([M+Na]$^+$): 516.1682. Found: 516.1688.

11-Iodo-10-(2-iodophenyl)-18-methyl-8b,9,14,14a-tetrahydro-9,14-[1,2]epitriazolobenzo[*f*]tetraphene-17,19-dione (**4la**)

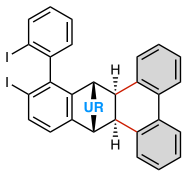

Method: Procedure A, 2.0 eq. of 2-iodo-1-(2-iodophenyl)naphthalene (**1l**) was used.

Purification: PTLC ($CHCl_3$), The product was obtained as a mixture of rotational isomers (major/minor = 62:38).

Yield: 55.1 mg, 76%, white solid

^{1}H NMR (600 MHz, CD_2Cl_2) δ 8.11 (d, *J* = 8.4 Hz, 0.62×1H), 8.04–8.08 (m, 0.62×1H, 0.38×2H), 7.95 (d, *J* = 7.8 Hz, 1H), 7.92 (d, *J* = 7.2 Hz, 1H), 7.66 (td, *J* = 7.5, 1.4 Hz, 0.38×1H), 7.52–7.60 (m, 0.62×2H, 0.38×1H), 7.19–7.40 (m, 0.62×6H, 0.38×7H), 7.15 (dd, *J* = 7.5, 1.5 Hz, 0.62×1H), 7.03 (d, *J* = 7.2 Hz, 0.62×1H), 6.98 (d, *J* = 7.2 Hz, 0.38×1H), 5.55 (d, *J* = 2.4 Hz, 0.38×1H), 5.49 (d, *J* = 3.0 Hz, 0.62×1H), 4.83–4.85 (m, 1H), 3.96 (dd, *J* = 12.3, 2.1 Hz, 0.62×1H), 3.71–3.78 (m, 1H), 3.53 (dd, *J* = 12.3, 1.5 Hz, 0.38×1H), 2.66 (s, 0.62×3H), 2.65 (s, 0.38×3H)

^{13}C NMR (150 MHz, CD_2Cl_2) δ 157.70, 157.39, 157.18, 156.53, 145.41, 145.07, 144.69, 144.20, 140.28, 139.59, 139.35, 139.29, 135.66, 135.26, 134.84, 134.81, 132.78, 132.72, 132.60, 132.51, 132.37, 132.28, 131.48, 130.95, 130.89, 129.60, 129.45, 129.136, 129.32, 129.20, 129.13, 128.91, 128.89, 128.30, 128.25, 128.22, 128.14, 125.53, 125.36, 123.31, 123.28, 123.15, 123.12, 101.36, 101.32, 99.43 (2C), 61.37 (2C), 60.85, 60.41, 38.84, 37.36 (2C), 37.26, 25.44, 25.41 (five sp^2 carbon signals were overlapping with others)

HRMS (ESI, positive): Calcd for $C_{31}H_{21}N_3O_2I_2Na$ ($[M+Na]^+$): 743.9615. Found: 743.9616.

10-Iodo-11-(2-iodophenyl)-18-methyl-8b,9,14,14a-tetrahydro-9,14-[1,2]epitriazolobenzo[*f*]tetraphene-17,19-dione (**4ma**)

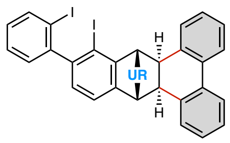

Method: Procedure A, 2.0 eq. of 1-iodo-2-(2-iodophenyl)naphthalene (**1m**) was used.

Purification: PTLC ($CHCl_3$), The product was obtained as a mixture of rotational isomers (major/minor = 63:37).

Yield: 45.7 mg, 63%, white solid

^{1}H NMR (600 MHz, $Cl_2CDCDCl_2$) δ 7.91–7.96 (m, 3H), 7.40–7.53 (m, 4H), 7.31–7.39 (m, 4H), 7.22–7.29 (m, 0.63×1H, 0.37×2H), 7.20 (dd, J = 7.2, 1.8 Hz, 0.63×1H), 7.08–7.13 (m, 1H), 5.86 (d, J = 1.8 Hz, 0.63×1H), 5.85 (d, J = 1.8 Hz, 0.37×1H), 5.45 (d, J = 3.0 Hz, 0.63×1H), 5.43 (d, J = 2.4 Hz, 0.37×1H), 3.63–3.73 (m, 2H), 2.68 (s, 0.63×3H), 2.62 (s, 0.37×3H)

^{13}C NMR (150 MHz, $Cl_2CDCDCl_2$) δ 157.44, 157.14, 156.51, 156.42, 149.93, 149.86, 148.10, 148.05, 139.49, 139.11, 138.91, 138.88, 134.98, 134.74, 131.83, 131.81, 131.74, 131.65, 131.54, 130.44, 130.21, 129.82, 129.74, 129.65, 129.44, 129.40, 129.06, 128.77, 128.61, 128.21, 127.95, 127.91, 123.54, 122.87, 122.76, 99.87, 99.76, 99.49, 99.28, 66.38, 66.07, 61.67, 61.29, 37.33, 37.14, 36.87, 36.74, 25.27, 25.06 (thirteen sp^2 carbon signals were overlapping with others).

HRMS (ESI, positive): Calcd for $C_{31}H_{21}N_3O_2I_2Na$ ([M+Na]$^+$): 743.9615. Found: 743.9616.

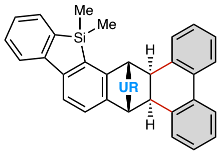

16,16,21-Trimethyl-8b,16,17,17a-tetrahydro-9*H*-9,17-[1,2]epitriazolobenzo[*b*]benzo[5,6]tetrapheno[9,8-*d*]silole-20,22-dione (**4na**)

Method: Procedure A, 2.0 eq. of 11,11-dimethyl-11*H*-benzo[*b*]naphtho[2,1-*d*]silole (**1n**) was used.

Purification: PTLC ($CHCl_3$)

Yield: 24.3 mg, 46%, white solid

^{1}H NMR (600 MHz, $CDCl_3$) δ 7.98 (d, J = 7.2 Hz, 2H), 7.88 (d, J = 7.2 Hz, 1H), 7.84 (d, J = 7.2 Hz, 1H), 7.68 (d, J = 6.6 Hz, 1H), 7.52 (d, J = 7.2 Hz, 2H), 7.46 (td, J = 7.8, 1.2 Hz, 1H), 7.31–7.43 (m, 6H), 5.52 (d, J = 3.0 Hz, 1H), 5.41 (d, J = 1.8 Hz, 1H), 3.72 (dd, J = 11.7, 2.7 Hz, 1H), 3.65 (dd, J = 11.7, 2.1 Hz, 1H), 2.67 (s, 3H), 0.71 (s, 3H), 0.65 (s, 3H)

^{13}C NMR (150 MHz, $CDCl_3$) δ 157.10, 157.04, 149.14, 146.90, 140.18, 138.57, 134.44, 133.50, 132.89, 132.47, 132.41, 132.11, 131.89, 130.48, 129.08 (2C), 128.73, 128.61, 128.06, 127.94, 127.80, 125.78, 123.02, 122.90, 121.11, 121.09, 63.16, 61.26, 38.42, 37.72, 25.12, –2.55, –2.97

HRMS (ESI, positive): Calcd for $C_{33}H_{27}N_3O_2SiNa$ ([M+Na]$^+$): 548.1765. Found: 548.1766.

18-Methyl-8b,9,14,14a-tetrahydro-9,14-[1,2]epitriazolophenanthro[9,10-*g*]quinoxaline-17,19-dione (**4oa**)

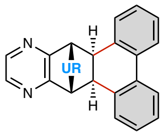

Method: Procedure B, 2.0 eq. of quinoxaline (**1o**) was used.

Purification: PTLC ($CHCl_3$/Ethyl acetate = 2:1)

Yield: 7.3 mg, 18%, white solid

^{1}H NMR (600 MHz, $CDCl_3$) δ 8.63 (s, 2H), 7.98 (dd, *J* = 6.3, 2.1 Hz, 2H), 7.53 (dd, *J* = 6.6, 1.8 Hz, 2H), 7.34–7.42 (m, 4H), 5.74 (s, 2H), 3.78 (s, 2H), 2.70 (s, 3H)

^{13}C NMR (150 MHz, $CDCl_3$) δ 155.86, 149.95, 145.19, 131.95, 130.76, 129.37, 128.89, 128.32, 123.00, 62.63, 37.21, 25.29

HRMS (ESI, positive): Calcd for $C_{23}H_{17}N_5O_2Na$ ($[M+Na]^+$): 418.1275. Found: 418.1274.

1.6 Annulative diarylation of cycloadduct 2g with mono-Grignard reagents 3′ or 3″

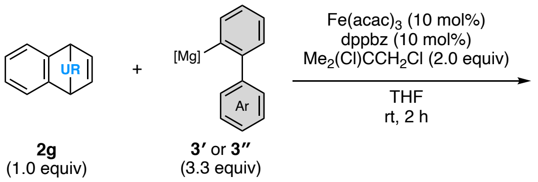

To a test tube containing a magnetic stirring bar was added cycloadduct **2g** (0.10 mmol, 1.0 equiv). The tube was sealed with a septum and filled with N_2 gas. THF (200 µL), 1,2-dichloroisobutane (0.20 mmol, 2.0 equiv) and THF solution (200 µL) of $Fe(acac)_3$ (0.01 mmol, 10 mol%) and dppbz (0.01 mmol, 10 mol%) were added in this order, and resulting mixture was stirred. Then, Grignard reagent in THF (0.33 mmol, 3.3 equiv) was added dropwise over 5 min, and resulting mixture was stirred at ambient temperature for 2 h. The mixture was quenched with 1 M HCl aq. (approx. 10 mL) and then extracted with dichloromethane (3×30 mL). The combined organic layers were dried with Na_2SO_4, filtered and concentrated under reduced pressure. The crude product was purified by PTLC to yield diarylated product **4**.

18-Methyl-8b,9,14,14a-tetrahydro-9,14-[1,2]epitriazolobenzo[*f*]tetraphene-17,19-dione (**4ga**)

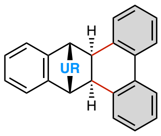

Purification: PTLC ($CHCl_3$)

Yield: 25.6 mg, 65%, white solid (with **3a′**); 20.0 mg, 51%, white solid (with **3a″**)

The NMR spectra of product were identical to those described above.

2-(*tert*-Butyl)-18-methyl-8b,9,14,14a-tetrahydro-9,14-[1,2]epitriazolobenzo[*f*]tetraphene-17,19-dione (**4gb**)

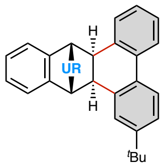

Purification: PTLC ($CHCl_3$)

Yield: 22.0 mg, 49%, white solid

^{1}H NMR (600 MHz, $CDCl_3$) δ 7.91–7.96 (m, 1H), 7.89 (d, J = 9.0 Hz, 1H), 7.50–7.55 (m, 1H), 7.41–7.49 (m, 5H), 7.37 (dd, J = 8.1, 2.1 Hz, 1H), 7.29–7.35 (m, 2H), 5.48 (d, J = 6.0 Hz, 2H), 3.65 (t, J = 12.9 Hz, 2H), 2.66 (s, 3H), 1.40 (s, 9H)

^{13}C NMR (150 MHz, $CDCl_3$) δ 157.12, 157.07, 151.58, 135.14, 135.04, 132.08, 132.05, 131.82, 129.39,

129.14 (2C), 129.11, 128.17, 127.79, 125.82, 125.14, 123.73, 123.71, 122.73, 122.64, 61.68, 61.60, 38.07, 37.88, 34.65, 31.26, 25.07

HRMS (ESI, positive): Calcd for $C_{29}H_{27}N_3O_2Na$ ($[M+Na]^+$): 472.1995. Found: 472.1996.

18-Methyl-2-(trifluoromethyl)-8b,9,14,14a-tetrahydro-9,14-[1,2]epitriazolobenzo[*f*]tetraphene-17,19-dione (**4gc**)

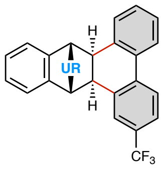

Purification: PTLC ($CHCl_3$)

Yield: 19.3 mg, 42%, white solid

^{1}H NMR (600 MHz, $CDCl_3$) δ 8.07 (d, J = 8.4 Hz, 1H), 7.98 (d, J = 7.2 Hz, 1H), 7.72 (s, 1H), 7.58 (d, J = 7.8 Hz, 1H), 7.54 (d, J = 7.2 Hz, 1H), 7.49–7.52 (m, 1H), 7.46–7.49 (m , 3H), 7.43 (t, J = 7.2 Hz, 1H), 7.38 (t, J = 7.5 Hz, 1H), 5.50 (d, J = 1.8 Hz, 1H), 5.46 (d, J = 2.4 Hz, 1H), 3.71 (dd, J = 12.3, 2.1 Hz, 1H), 3.68 (dd, J = 12.3, 2.1 Hz, 1H), 2.67 (s, 3H)

^{13}CNMR (150 MHz, $CDCl_3$) δ 157.23, 157.21, 135.57, 134.69, 134.62, 133.18, 133.01, 130.64, 130.26 ($^3J_{C\text{-}F}$ = 32.6 Hz), 129.73, 129.41 (2C), 129.36, 128.14, 126.14 ($^4J_{C\text{-}F}$ = 3.9 Hz), 124.73 ($^4J_{C\text{-}F}$ = 3.9 Hz), 123.96 ($^2J_{C\text{-}F}$ = 270 Hz), 123.81 (2C), 123.51, 123.45, 61.69, 61.51, 37.94, 37.62, 25.13

HRMS (ESI, positive): Calcd for $C_{26}H_{18}N_3O_2F_3Na$ ($[M+Na]^+$): 484.1243. Found: 484.1246.

2-Methoxy-18-methyl-8b,9,14,14a-tetrahydro-9,14-[1,2]epitriazolobenzo[*f*]tetraphene-17,19-dione (**4gd**)

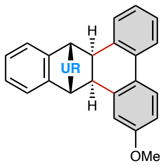

Purification: PTLC ($CHCl_3$)

Yield: 27.2 mg, 64%, white solid

^{1}H NMR (600 MHz, $CDCl_3$) δ 7.83–7.91 (m, 2H), 7.42–7.50 (m, 5H), 7.28–7.33 (m, 2H), 6.98 (d, J = 2.4 Hz, 1H), 6.89 (dd, J = 8.7, 2.1 Hz, 1H), 5.49 (d, J = 2.4 Hz, 1H), 5.48 (d, J = 2.4 Hz, 1H), 3.90 (s, 3H), 3.64 (dd, J = 12.3, 2.1 Hz, 1H), 3.61 (dd, J = 12.3, 2.1 Hz, 1H), 2.67 (s, 3H)

^{13}C NMR (150 MHz, $CDCl_3$) δ 159.74, 157.18, 157.15, 134.98, 134.94, 133.93, 132.06, 131.33, 129.21, 129.18, 129.15, 127.85, 127.62, 125.04, 124.42, 123.73, 123.71, 122.23, 114.45, 113.48, 61.68 (2C), 55.40,

38.05, 37.77, 25.09

HRMS (ESI, positive): Calcd for $C_{26}H_{21}N_3O_3Na$ ($[M+Na]^+$): 446.1475. Found: 446.1476.

20-Methyl-8b,9,14,14a-tetrahydro-9,14-[1,2]epitriazolodibenzo[*c*,*f*]tetraphene-19,21-dione (**4ge**)

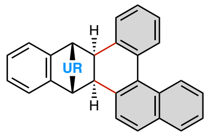

Purification: PTLC ($CHCl_3$)

Yield: 27.2 mg, 61%, white solid

^{1}**H NMR** (600 MHz, $CDCl_3$) δ 8.62 (d, J = 9.0 Hz, 1H), 8.10 (dd, J = 7.8, 1.2 Hz, 1H), 7.85 (dd, J = 8.4, 1.2 Hz, 1H), 7.79 (d, J = 9.0 Hz, 1H), 7.55 (d, J = 7.8 Hz, 1H), 7.43–7.53 (m, 7H), 7.31–7.39 (m, 2H), 5.53 (d, J = 2.4 Hz, 1H), 5.52 (d, J = 3.6 Hz, 1H), 3.90 (dd, J = 11.7, 2.7 Hz, 1H), 3.59 (dd, J = 11.7, 1.5 Hz, 1H), 2.57 (s, 3H)

^{13}**C NMR** (150 MHz, $CDCl_3$) δ 157.10, 156.16, 136.01, 134.66, 134.21, 133.47, 133.08, 132.17, 130.73, 130.18, 129.96, 129.31, 129.17 (2C), 128.47, 128.45, 127.99, 127.07, 126.75, 125.86, 125.58, 125.53, 124.01, 123.39, 61.49, 60.06, 40.41, 38.26, 25.00

HRMS (ESI, positive): Calcd for $C_{29}H_{21}N_3O_2Na$ ($[M+Na]^+$): 466.1526. Found: 466.1525.

21-Methyl-9b,10,15,15a-tetrahydro-10,15-[1,2]epitriazolobenzo[5,6]tetrapheno[4,3-*b*]benzofuran-20,22-dione (**4gf**)

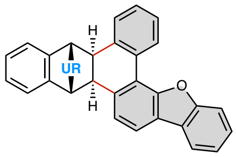

Purification: PTLC ($CHCl_3$)

Yield: 24.7 mg, 51%, white solid

^{1}**H NMR** (600 MHz, $CDCl_3$) δ 9.20 (d, J = 7.8 Hz, 1H), 7.96 (d, J = 7.2 Hz, 1H), 7.94 (d, J = 7.8 Hz, 1H), 7.69 (d, J = 7.2 Hz, 1H), 7.55 (d, J = 7.8 Hz, 1H), 7.41–7.52 (m, 8H), 7.38 (t, J = 7.5 Hz, 1H), 5.54 (d, J = 2.4 Hz, 1H), 5.51 (d, J = 1.8 Hz, 1H), 3.85 (dd, J = 12.6, 1.8 Hz, 1H), 3.75 (dd, J = 12.3, 2.1 Hz, 1H), 2.63 (s, 3H)

^{13}**C NMR** (150 MHz, $CDCl_3$) δ 157.00, 156.02, 153.46, 135.05, 134.99, 132.63, 132.42, 130.63, 129.26, 129.22, 128.99, 128.69, 128.24, 127.98, 127.15, 124.61, 124.09, 123.77, 123.73, 123.51, 123.03, 120.41 (2C), 119.08, 111.75, 62.30, 61.90, 38.56, 38.30, 25.06 (one sp^2 carbon signal was overlapping with others)

HRMS (ESI, positive): Calcd for $C_{31}H_{21}N_3O_3Na$ ($[M+Na]^+$): 506.1477. Found: 506.1483.

19-Methyl-9b,10,15,15a-tetrahydro-10,15-[1,2]epitriazolobenzo[*b*]tetrapheno[6,5-*d*]thiophene-18,20-dione (**4gg**)

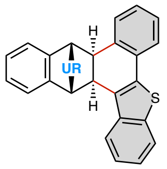

Purification: PTLC ($CHCl_3$)

Yield: 15.6 mg, 35%, white solid

^{1}H NMR (600 MHz, $CDCl_3$) δ 7.87 (d, *J* = 8.4 Hz, 1H), 7.72 (d, *J* = 7.8 Hz, 1H), 7.43–7.56 (m, 7H), 7.33–7.41 (m, 2H), 7.31 (t, *J* = 7.5 Hz, 1H), 5.72 (bs, 1H), 5.63 (d, *J* = 2.4 Hz, 1H), 3.83–3.91 (m, 2H), 2.68 (s, 3H)

^{13}C NMR (150 MHz, $CDCl_3$) δ 157.15, 156.73, 139.32, 138.63, 137.82, 135.36, 134.43, 132.39, 130.17, 129.36, 129.30, 129.05, 128.26, 128.00, 125.40, 124.98, 124.96, 124.52, 124.10, 123.60, 123.24, 120.74, 60.57, 57.28, 38.23, 36.42, 25.11

HRMS (ESI, positive): Calcd for $C_{27}H_{19}N_3O_2SNa$ ($[M+Na]^+$):472.1090. Found: 472.1091.

1.7 Rearomatization of diarylated compounds

Procedure C

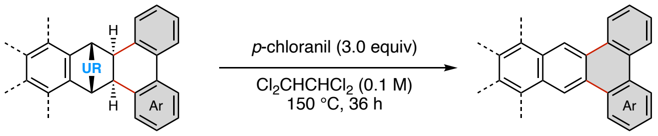

To a screw-capped tube containing a magnetic stirring bar were added diarylated compound **4** (1.0 equiv) and *p*-chloranil (3.0 equiv). Then, 1,1,2,2-tetrachloroethane was added to this tube to prepare 0.1 M solution of **4** under air. The tube was sealed with a cap, and resulting mixture was stirred at 150 °C for 36 h. Then, the reaction mixture was cooled to ambient temperature and diluted with chloroform (approx. 3 mL). To this mixture, hydrazine monohydrate (5.0 equiv) was added and the resulting mixture was stirred at ambient temperature for 15 min to quench remaining *p*-chloranil. The mixture was washed with 1 M NaOH aq. (approx. 10 mL) and extracted with chloroform (3×30 mL). The combined organic layers were dried with Na_2SO_4, filtered and concentrated under reduced pressure. The crude product was purified by flash column chromatography on silica gel to yield π-extended product **5**.

Procedure D

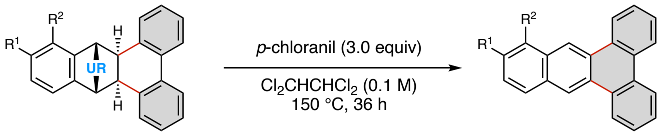

To a screw-capped tube containing a magnetic stirring bar were added diarylated compound **4ea** or **4fa** (1.0 equiv) and *p*-chloranil (3.0 equiv). Then, 1,1,2,2-tetrachloroethane was added to this tube to prepare 0.1 M solution of **4** under air. The tube was sealed with a cap, and resulting mixture was stirred at 150 °C for 36 h. The reaction mixture was cooled to ambient temperature, and then passed through a short pad of silica gel (eluent: $CHCl_3$). After the organic solvent was removed under reduced pressure, the crude product was purified by flash column chromatography to yield π-extended product **5ea** or **5fa**.

10-Phenylbenzo[*f*]tetraphene (**5aa**)

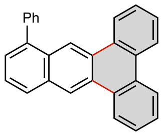

Method: Procedure C (0.060 mmol scale)

Purification: flash column chromatography (Hexane/$CHCl_3$ = 5:1)

Yield: 20.4 mg, 96%, white solid

^{1}H NMR (600 MHz, $CDCl_3$) δ 9.20 (s, 1H), 9.13 (s, 1H), 8.79 (dd, *J* = 7.8, 1.2 Hz, 1H), 8.52–8.59 (m, 2H), 8.39 (d, *J* = 7.8 Hz, 1H), 8.10 (d, *J* = 8.4 Hz, 1H), 7.57–7.70 (m, 8H), 7.54 (t, *J* = 7.2 Hz, 2H), 7.51 (d, *J* = 6.0 Hz, 1H)

^{13}C NMR (150 MHz, $CDCl_3$) δ 140.72, 140.33, 132.65, 130.48, 130.28, 130.24, 130.14, 130.04, 129.97, 128.50, 128.44, 128.27, 127.82, 127.69, 127.54, 127.49, 127.46, 127.03, 125.68, 123.70, 123.42, 123.37, 122.39, 120.42 (two sp^2 carbon signals were overlapping with others)

HRMS (APCI, positive): Calcd for $C_{28}H_{19}$ ([M+H]$^+$): 355.1481. Found: 355.1475.

10-(Naphthalen-1-yl)benzo[*f*]tetraphene (**5ba**)

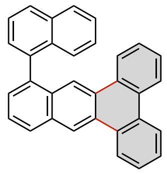

Method: Procedure C (0.071 mmol scale)

Purification: flash column chromatography (Hexane/$CHCl_3$ = 5:1)

Yield: 18.6 mg, 65%, white solid

^{1}H NMR (600 MHz, $CDCl_3$) δ 9.18 (s, 1H), 8.81 (d, *J* = 7.8 Hz, 1H), 8.70 (s, 1H), 8.53 (d, *J* = 7.8 Hz, 1H), 8.49 (d, *J* = 8.4 Hz, 1H), 8.19 (d, *J* = 8.4 Hz, 1H), 8.06 (d, *J* = 7.8 Hz, 1H), 8.02 (d, *J* = 7.8 Hz, 1H), 7.97 (d, *J* = 8.4 Hz, 1H), 7.60–7.73 (m, 5H), 7.58 (d, *J* = 7.2 Hz, 1H), 7.54 (d, *J* = 9.0 Hz, 1H), 7.47–7.52 (m, 2H), 7.35 (t, *J* = 7.5 Hz, 1H), 7.28 (t, *J* = 7.8 Hz, 1H)

^{13}C NMR (150 MHz, $CDCl_3$) δ 138.58, 138.25, 133.69, 132.97, 132.45, 131.72, 130.12, 130.10, 129.97, 129.95, 128.42, 128.35, 128.23, 128.17, 128.14, 128.11 (2C), 127.68, 127.47 (2C), 127.32, 126.56, 126.13, 125.93, 125.64, 125.46, 123.70, 123.61, 123.41, 123.23, 122.33, 120.92

HRMS (APCI, positive): Calcd for $C_{32}H_{21}$ ([M+H]$^+$): 405.1638. Found: 405.1638

10-Bromobenzo[*f*]tetraphene (**5ca**)

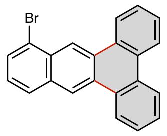

Method: Procedure C (0.048 mmol scale)

Purification: flash column chromatography (Hexane/$CHCl_3$ = 5:1)

Yield: 13.6 mg, 79%, white solid

^{1}H NMR (600 MHz, $CDCl_3$) δ 9.46 (s, 1H), 9.03 (s, 1H), 8.84 (d, *J* = 7.2 Hz, 1H), 8.74 (dd, *J* = 5.7, 3.3 Hz, 1H), 8.53–8.61 (m, 2H), 8.03 (d, *J* = 8.4 Hz, 1H), 7.85 (d, *J* = 6.6 Hz, 1H), 7.63–7.72 (m, 4H), 7.38 (t, *J* = 8.1 Hz, 1H)

^{13}C NMR (150 MHz, $CDCl_3$) δ 133.11, 130.60, 130.29, 130.20, 129.89, 129.61, 129.51, 129.14, 128.15, 128.02 (2C), 127.68, 127.56, 126.18, 124.04, 123.81, 123.49, 123.43, 123.07, 122.60, 121.71 (one sp^2 carbon signal was overlapping with others)

HRMS (APCI, positive): Calcd for $C_{22}H_{14}Br$ ($[M+H]^+$): 357.0273. Found: 357.0273.

10-Iodobenzo[*f*]tetraphene (**5da**)

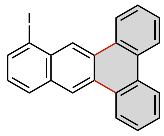

Method: Procedure C (0.058 mmol scale)

Purification: flash column chromatography (Hexane/$CHCl_3$ = 5:1)

Yield: 17.7 mg, 76%, white solid

^{1}H NMR (600 MHz, CD_2Cl_2) δ 9.37 (s, 1H), 9.07 (s, 1H), 8.89 (dd, *J* = 7.5, 1.5 Hz, 1H), 8.78–8.82 (m, 1H), 8.61–8.66 (m, 2H), 8.20 (d, *J* = 7.2 Hz, 1H), 8.13 (d, *J* = 7.8 Hz, 1H), 7.68–7.77 (m, 4H), 7.29 (t, *J* = 8.1 Hz, 1H)

^{13}C NMR (150 MHz, CD_2Cl_2) δ 138.09, 133.17, 132.94, 130.59 (2C), 130.26, 130.05, 129.81, 129.64, 129.50, 128.53, 128.48, 128.13, 128.07, 127.35, 127.13, 124.37, 124.26, 123.86 (2C), 123.30, 100.05

HRMS (DART, positive): Calcd for $C_{22}H_{14}I$ ($[M+H]^+$): 405.0140. Found: 405.0140.

Benzo[*f*]tetraphen-10-yl acetate (**5ea**)

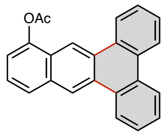

Method: Procedure D (0.059 mmol scale)

Purification: flash column chromatography (Hexane/$CHCl_3$ = 1:1)

Yield: 11.7 mg, 59%, white solid

^{1}H NMR (600 MHz, $CDCl_3$) δ 9.07 (s, 1H), 9.06 (s, 1H), 8.70–8.75 (m, 1H), 8.65–8.70 (m, 1H), 8.52–8.58 (m, 2H), 7.95 (d, J = 8.4 Hz, 1H), 7.60–7.69 (m, 4H), 7.52 (t, J = 8.1 Hz, 1H), 7.33 (d, J = 7.8 Hz, 1H), 2.61 (s, 3H)

^{13}C NMR (150 MHz, $CDCl_3$) δ 169.52, 146.65, 133.29, 130.19, 130.13, 129.89, 129.76, 128.95, 128.78, 127.87 (2C), 127.54, 127.48, 126.16, 125.70, 125.42, 123.71, 123.62, 123.48, 123.43, 122.30, 117.88, 115.29, 21.28

HRMS (APCI, positive): Calcd for $C_{24}H_{17}O_2$ ([M+H]$^+$): 337.1223. Found: 337.1222.

Benzo[*f*]tetraphen-11-yl pivalate (**5fa**)

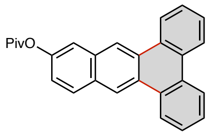

Method: Procedure D (0.032 mmol scale)

Purification: flash column chromatography (Hexane/$CHCl_3$ = 1:1)

Yield: 8.8 mg, 73%, white solid

^{1}H NMR (600 MHz, $CDCl_3$) δ 9.04 (s, 1H), 8.98 (s, 1H), 8.66–8.74 (m, 2H), 8.53–8.58 (m, 2H), 8.06 (d, J = 9.0 Hz, 1H), 7.76 (s, 1H), 7.59–7.68 (m, 4H), 7.27 (dd, J = 8.7, 2.1 Hz, 1H), 1.45 (s, 9H)

^{13}C NMR (150 MHz, $CDCl_3$) δ 177.30, 148.98, 132.39, 130.16 (2C), 129.99, 129.96, 129.84, 129.60, 128.96, 128.24, 127.78, 127.62, 127.51 (2C), 123.65, 123.55, 123.43 (2C), 122.00, 121.81, 121.67, 118.13, 39.22, 27.22

HRMS (APCI, positive): Calcd for $C_{27}H_{23}O_2$ ([M+H]$^+$): 379.1693. Found: 379.1697.

Benzo[*f*]tetraphene (**5ga**)

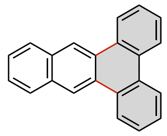

Method: Procedure C (0.065 mmol scale)

Purification: flash column chromatography (Hexane/$CHCl_3$ = 5:1)

Yield: 15.2 mg, 84%, white solid

^{1}H NMR (600 MHz, $CDCl_3$) δ 9.07 (s, 2H), 8.77 (dd, J = 7.2, 2.4 Hz, 2H), 8.57 (dd, J = 6.6, 2.4 Hz, 2H), 8.05–8.10 (m, 2H), 7.61–7.68 (m, 4H), 7.54–7.58 (m, J = 6.3, 3.2 Hz, 2H)

^{13}C NMR (150 MHz, $CDCl_3$) δ 132.23, 130.15, 130.05, 128.46, 128.09, 127.61, 127.49, 126.09, 123.68, 123.43, 122.06

HRMS (APCI, positive): Calcd for $C_{22}H_{15}$ ($[M+H]^+$): 279.1168. Found: 279.1167.

Dibenzo[*f*,*k*]tetraphene (**5ha**)

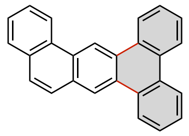

Method: Procedure C (0.056 mmol scale)

Purification: flash column chromatography (Hexane/$CHCl_3$ = 5:1)

Yield: 13.2 mg, 72%, white solid

^{1}H NMR (600 MHz, $CDCl_3$) δ 9.94 (s, 1H), 9.07 (s, 1H), 8.98 (d, *J* = 8.4 Hz, 1H), 8.95 (d, *J* = 7.8 Hz, 1H), 8.81 (d, *J* = 7.8 Hz, 1H), 8.61–8.67 (m, 2H), 7.93 (d, *J* = 8.4 Hz, 2H), 7.64–7.81 (m, 7H)

^{13}C NMR (150 MHz, $CDCl_3$) δ 132.25, 131.11, 130.42, 130.22, 130.11, 130.02, 129.81, 129.17, 128.79, 128.72, 128.45, 127.57, 127.53, 127.49, 127.46 (2C), 127.11, 126.97, 126.87, 123.59, 123.56, 123.52, 123.43, 122.73 (2C), 117.03

HRMS (APCI, positive): Calcd for $C_{26}H_{17}$ ($[M+H]^+$): 326.1325. Found: 329.1325.

13-Phenyldibenzo[*f*,*k*]tetraphene (**5ia**)

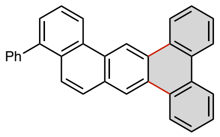

Method: Procedure C (0.036 mmol scale)

Purification: flash column chromatography (Hexane/$CHCl_3$ = 5:1)

Yield: 9.7 mg, 67%, white solid

^{1}H NMR (600 MHz, $CDCl_3$) δ 9.99 (s, 1H), 9.04 (s, 1H), 9.01 (d, *J* = 8.4 Hz, 1H), 8.96 (d, *J* = 7.8 Hz, 1H), 8.77–8.81 (m, 1H), 8.61–8.67 (m, 2H), 7.85 (s, 2H), 7.78 (t, *J* = 7.5 Hz, 1H), 7.74 (t, *J* = 7.2 Hz, 1H), 7.65–7.71 (m, 3H), 7.61 (d, *J* = 6.6 Hz, 1H), 7.52–7.57 (m, 4H), 7.45–7.50 (m, 1H)

^{13}C NMR (150 MHz, $CDCl_3$) δ 141.23, 141.04, 130.75 (2C), 130.24, 130.11, 130.02 (2C), 129.78, 129.25, 128.83, 128.51, 128.31, 127.59, 127.53, 127.46, 127.43, 127.29, 127.01, 126.22, 125.11, 123.62, 123.56, 123.53, 123.41, 122.58, 122.18, 117.34 (two sp^2 carbon signals were overlapping with others)

HRMS (APCI, positive): Calcd for $C_{32}H_{21}$ ($[M+H]^+$): 405.1638. Found: 405.1638.

Tribenzo[*a*,*k*,*m*]tetraphene (**5ja**)

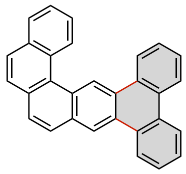

Method: Procedure C (0.046 mmol scale)

Purification: flash column chromatography (Hexane/$CHCl_3$ = 5:1)

Yield: 9.9 mg, 57%, white solid

^{1}H NMR (600 MHz, $CDCl_3$) δ 10.37 (s, 1H), 9.31 (d, *J* = 8.4 Hz, 1H), 9.16 (s, 1H), 8.83 (d, *J* = 7.8 Hz, 1H), 8.80 (d, *J* = 7.8 Hz, 1H), 8.63–8.67 (m, 2H), 8.07–8.12 (m, 2H), 7.98 (d, *J* = 8.4 Hz, 1H), 7.88 (t, *J* = 8.7 Hz, 2H), 7.80–7.85 (m, 1H), 7.66–7.74 (m, 5H)

^{13}C NMR (150 MHz, $CDCl_3$) δ 133.73, 132.36, 131.14, 130.47, 130.41, 130.28, 130.14, 129.94, 129.17, 128.82, 128.19, 128.12, 127.76, 127.67 (2C), 127.56, 127.50, 127.47, 127.40, 126.89, 126.66, 125.90, 123.85, 123.63, 123.57, 123.45, 122.65, 122.45 (two sp^2 carbon signals were overlapping with others)

HRMS (APCI, positive): Calcd for $C_{30}H_{19}$ ($[M+H]^+$): 379.1481. Found: 379.1480.

Tribenzo[*c*,*k*,*m*]tetraphene (**5ka**)

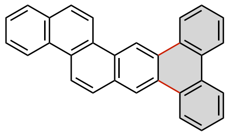

Method: Procedure C (0.034 mmol scale)

Purification: flash column chromatography (Hexane/$CHCl_3$ = 5:1)

Yield: 7.6 mg, 59%, white solid

^{1}H NMR (600 MHz, $CDCl_3$) δ 10.06 (s, 1H), 9.19 (s, 1H), 9.03 (d, *J* = 9.0 Hz, 1H), 9.00 (d, *J* = 7.2 Hz, 1H), 8.85 (d, *J* = 7.8 Hz, 1H), 8.82 (d, *J* = 8.4 Hz, 1H), 8.78 (d, *J* = 9.6 Hz, 1H), 8.65 (t, *J* = 6.9 Hz, 2H), 8.22 (d, *J* = 9.6 Hz, 1H), 8.13 (d, *J* = 8.4 Hz, 1H), 8.05 (d, *J* = 7.8 Hz, 1H), 7.65–7.79 (m, 6H)

^{13}C NMR (150 MHz, $CDCl_3$) δ 132.41, 131.13, 130.70, 130.33, 130.22, 130.12, 129.87, 129.43, 128.66 (3C), 128.36, 128.30, 127.70, 127.65, 127.61, 127.55 (2C), 127.53, 126.81, 126.44, 123.69, 123.65, 123.58, 123.48, 123.23, 122.71, 121.75, 121.22, 117.57

HRMS (APCI, positive): Calcd for $C_{30}H_{19}$ ($[M+H]^+$): 379.1481. Found: 379.1479.

11-Iodo-10-(2-iodophenyl)benzo[*f*]tetraphene (**5la**)

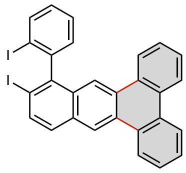

Method: Procedure C (0.076 mmol scale)

Purification: flash column chromatography (Hexane/$CHCl_3$ = 5:1)

Yield: 37.4 mg, 81%, white solid

^{1}H NMR (600 MHz, $CDCl_3$) δ 9.11 (s, 1H), 8.75–8.80 (m, 1H), 8.52–8.59 (m, 2H), 8.46 (s, 1H), 8.16 (t, *J* = 8.4 Hz, 2H), 8.03 (d, *J* = 9.0 Hz, 1H), 7.87 (d, *J* = 7.8 Hz, 1H), 7.65–7.70 (m, 2H), 7.63 (t, *J* = 7.5 Hz, 1H), 7.60 (t, *J* = 7.5 Hz, 1H), 7.51 (t, *J* = 7.5 Hz, 1H), 7.37 (dd, *J* = 7.8, 1.2 Hz, 1H), 7.31 (td, *J* = 7.7, 1.6 Hz, 1H)

^{13}C NMR (150 MHz, $CDCl_3$) δ 147.55, 146.26, 139.49, 135.47, 131.46, 131.43, 131.03, 130.23, 130.21, 129.81, 129.63, 129.52, 129.26, 128.84, 128.69, 127.97, 127.87, 127.60, 127.52, 123.73, 123.66, 123.47, 123.41, 122.36, 120.90, 100.44, 98.99 (one sp^2 carbon signal was overlapping with others)

HRMS (DART, positive): Calcd for $C_{28}H_{17}I_2$ ($[M+H]^+$): 606.9420. Found: 606.9430.

10-Iodo-11-(2-iodophenyl)benzo[*f*]tetraphene (**5ma**)

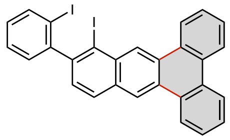

Method: Procedure C (0.063 mmol scale)

Purification: flash column chromatography (Hexane/$CHCl_3$ = 5:1)

Yield: 26.0 mg, 68%, white solid

^{1}H NMR (600 MHz, $CDCl_3$) δ 9.56–9.59 (m, 1H), 9.05–9.08 (m, 1H), 8.84–8.90 (m, 1H), 8.76–8.81 (m, 1H), 8.57–8.64 (m, 2H), 8.11 (d, *J* = 7.8 Hz, 1H), 8.03 (dd, *J* = 8.1, 1.5 Hz, 1H), 7.64–7.74 (m, 4H), 7.50 (td, *J* = 7.5, 1.4 Hz, 1H), 7.35 (d, *J* = 9.0 Hz, 2H), 7.16 (td, *J* = 7.7, 1.6 Hz, 1H)

^{13}C NMR (150 MHz, $CDCl_3$) δ 150.45, 148.36, 138.97 (2C), 133.18, 131.56, 130.37 (2C), 130.27, 130.14, 129.86, 129.48, 129.41, 129.32, 128.80, 128.16, 128.07 (2C), 127.76, 127.64, 127.34, 124.12, 123.94, 123.54, 123.49, 122.64, 105.40, 99.29

HRMS (DART, positive): Calcd for $C_{28}H_{17}I_2$ ($[M+H]^+$): 606.9420. Found: 606.9443.

16,16-Dimethyl-16*H*-benzo[*b*]benzo[5,6]tetrapheno[9,8-*d*]silole (**5na**)

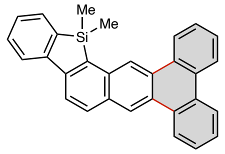

Method: Procedure C (0.046 mmol scale)

Purification: flash column chromatography (Hexane/$CHCl_3$ = 5:1)

Yield: 10.8 mg, 57%, white solid

^{1}H NMR (600 MHz, $CDCl_3$) δ 9.10 (s, 1H), 9.07 (s, 1H), 8.77 (d, J = 7.8 Hz, 1H), 8.72 (d, J = 7.8 Hz, 1H), 8.60 (t, J = 8.4 Hz, 2H), 8.19 (d, J = 8.4 Hz, 1H), 8.06 (d, J = 9.0 Hz, 1H), 7.94 (d, J = 7.8 Hz, 1H), 7.74 (d, J = 7.2 Hz, 1H), 7.62–7.73 (m, 4H), 7.51 (td, J = 7.5, 1.2 Hz, 1H), 7.35 (t, J = 6.9 Hz, 1H), 0.75 (s, 6H)

^{13}C NMR (150 MHz, $CDCl_3$) δ 148.12, 147.17, 139.12, 137.10, 135.33, 132.73, 131.93, 131.16, 130.33, 130.26, 130.18, 130.05, 129.93, 128.99, 128.16, 127.73, 127.59, 127.56 (2C), 127.51, 123.59, 123.57, 123.46, 123.34, 123.06, 122.31, 121.12, 120.28, -2.38

HRMS (APCI, positive): Calcd for $C_{30}H_{23}Si$ ($[M+H]^+$): 411.1564. Found: 411.1567.

Phenanthro[9,10-*g*]quinoxaline (**5oa**)

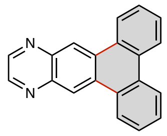

Method: Procedure C (0.018 mmol scale)

Purification: flash column chromatography ($CHCl_3$)

Yield: 3.7 mg, 73%, white solid

^{1}H NMR (600 MHz, $CDCl_3$) δ 9.34 (s, 2H), 8.95 (s, 2H), 8.77–8.83 (m, 2H), 8.58–8.64 (m, 2H), 7.68–7.75 (m, 4H)

^{13}C NMR (150 MHz, $CDCl_3$) δ 145.61, 141.10, 132.24, 130.52, 129.18, 128.79, 127.94, 124.44, 123.58, 123.51

HRMS (ESI, positive): Calcd for $C_{20}H_{13}N_2$ ($[M+H]^+$): 281.1073. Found: 281.1074.

2-(*tert*-Butyl)benzo[*f*]tetraphene (**5gb**)

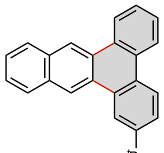

Method: Procedure C (0.047 mmol scale)

Purification: flash column chromatography (Hexane/$CHCl_3$ = 5:1)

Yield: 10.0 mg, 64%, white solid

^{1}H NMR (600 MHz, $CDCl_3$) δ 9.08 (s, 2H), 8.73–8.79 (m, 2H), 8.53–8.57 (m, 1H), 8.51 (d, J = 8.4 Hz, 1H), 8.12 (d, J = 7.2 Hz, 1H), 8.08 (d, J = 8.4 Hz, 1H), 7.71 (dd, J = 9.0, 1.8 Hz, 1H), 7.60–7.66 (m, 2H), 7.53–7.59 (m, 2H), 1.55 (s, 9H)

^{13}C NMR (150 MHz, $CDCl_3$) δ 150.26, 132.19, 132.13, 130.09, 129.86, 129.64, 128.82, 128.68, 128.09, 128.07, 127.70, 127.56, 127.09, 126.01, 125.98, 125.53, 123.61, 123.26 (2C), 122.12, 121.75, 119.75, 35.10, 31.50

HRMS (APCI, positive): Calcd for $C_{26}H_{23}$ ([M+H]$^+$): 355.1794. Found: 355.1793.

2-(Trifluoromethyl)benzo[*f*]tetraphene (**5gc**)

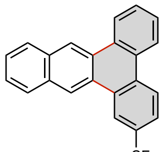

Method: Procedure C (0.042 mmol scale)

Purification: flash column chromatography (Hexane/$CHCl_3$ = 5:1)

Yield: 8.4 mg, 58%, white solid

^{1}H NMR (600 MHz, $CDCl_3$) δ 9.06 (s, 1H), 9.04 (s, 1H), 8.97 (s, 1H), 8.76 (d, J = 7.8 Hz, 1H), 8.64 (d, J = 8.4 Hz, 1H), 8.56 (d, J = 7.8 Hz, 1H), 8.05–8.15 (m, 2H), 7.83 (d, J = 8.4 Hz, 1H), 7.72 (t, J = 7.2 Hz, 1H), 7.67 (t, J = 7.8 Hz, 1H), 7.57–7.63 (m, 2H)

^{13}C NMR (150 MHz, $CDCl_3$) δ 132.67, 132.56, 132.24, 130.82, 130.22, 129.17 ($^3J_{C\text{-}F}$ = 33 Hz), 128.86, 128.57, 128.27, 128.20, 128.10, 127.85, 127.40, 126.63, 126.49, 124.58 ($^2J_{C\text{-}F}$ = 254 Hz), 124.14, 123.89, 123.79, 123.61 ($^4J_{C\text{-}F}$ = 4.4 Hz), 122.38, 122.33, 120.87 ($^4J_{C\text{-}F}$ = 4.4 Hz)

HRMS (APCI, positive): Calcd for $C_{23}H_{14}F_3$ ([M+H]$^+$): 347.1042. Found: 347.1044.

2-(Methoxy)benzo[*f*]tetraphene (**5gd**)

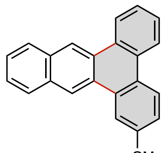

Method: Procedure C (0.064 mmol scale)

Purification: flash column chromatography (Hexane/$CHCl_3$ = 5:1)

Yield: 14.9 mg, 75%, white solid

^{1}H NMR (600 MHz, $CDCl_3$) δ 9.05 (s, 1H), 8.97 (s, 1H), 8.70–8.75 (m, 1H), 8.44–8.49 (m, 2H), 8.16 (d, J = 1.8 Hz, 1H), 8.05–8.09 (m, 2H), 7.53-7.63 (m, 4H), 7.24 (dd, J = 9.0, 3.0 Hz, 1H), 4.06 (s, 3H)

^{13}C NMR (150 MHz, $CDCl_3$) δ 159.10, 132.29, 132.09, 131.59, 130.16, 129.11, 128.76, 128.22, 128.08, 128.05, 127.62, 126.51, 126.12, 126.03, 125.04, 123.88, 123.61, 122.87, 122.09, 122.05, 115.60, 106.72, 55.54

HRMS (APCI, positive): Calcd for $C_{23}H_{17}O$ ($[M+H]^+$): 309.1274. Found: 309.1272.

Dibenzo[*c*,*f*]tetraphene (**5ge**)

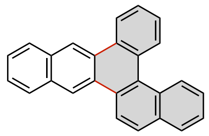

Method: Procedure C (0.061 mmol scale)

Purification: flash column chromatography (Hexane/$CHCl_3$ = 5:1)

Yield: 18.1 mg, 90%, white solid

^{1}H NMR (600 MHz, $CDCl_3$) δ 9.15 (s, 1H), 9.10 (s, 1H), 8.91 (d, J = 8.4 Hz, 1H), 8.84 (d, J = 8.4 Hz, 1H), 8.81 (d, J = 8.4 Hz, 1H), 8.76 (d, J = 9.0 Hz, 1H), 8.10–8.16 (m, 2H), 8.04 (d, J = 9.0 Hz, 1H), 8.02 (d, J = 7.2 Hz, 1H), 7.71 (t, J = 7.2 Hz, 1H), 7.56–7.67 (m, 5H)

^{13}C NMR (150 MHz, $CDCl_3$) δ 133.87, 132.42, 132.18, 131.38, 130.33, 129.87, 129.66, 128.75, 128.70, 128.49, 128.24 (2C), 128.19, 128.10, 127.87, 127.68, 126.93, 126.40, 126.10, 126.03 (2C), 125.85, 124.13, 122.58, 121.79, 120.92

HRMS (APCI, positive): Calcd for $C_{26}H_{17}$ ($[M+H]^+$): 329.1325. Found: 329.1320.

Benzo[5,6]tetrapheno[4,3-*b*]benzofuran (**5gf**)

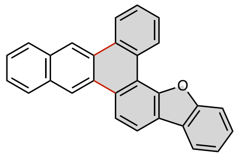

Method: Procedure C (0.051 mmol scale)

Purification: flash column chromatography (Hexane/$CHCl_3$ = 5:1)

Yield: 12.4 mg, 66%, white solid

^{1}**H NMR** (600 MHz, $CDCl_3$) δ 9.82 (d, *J* = 7.2 Hz, 1H), 9.14 (s, 1H), 9.12 (s, 1H), 8.85 (d, *J* = 8.4 Hz, 1H), 8.80 (d, *J* = 8.4 Hz, 1H), 8.19 (d, *J* = 9.0 Hz, 1H), 8.04–8.14 (m, 3H), 7.77–7.86 (m, 2H), 7.74 (t, *J* = 7.5 Hz, 1H), 7.53–7.60 (m, 3H), 7.44 (t, *J* = 7.5 Hz, 1H)

^{13}**C NMR** (150 MHz, $CDCl_3$) δ 156.17, 154.07, 132.33, 132.24, 130.44, 130.29, 128.74, 128.66, 128.54 (2C), 128.14, 128.08, 127.82, 127.55, 127.04, 126.18, 126.16, 123.83, 123.65, 123.28, 123.13, 122.83, 122.15, 120.47, 119.36, 118.92, 117.57, 111.87

HRMS (APCI, positive): Calcd for $C_{28}H_{17}O$ ($[M+H]^+$): 369.1274. Found: 369.1275.

Benzo[*b*]tetrapheno[6,5-*d*]thiophene (**5gg**)

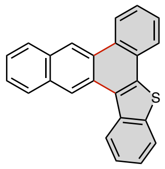

Method: Procedure C (0.035 mmol scale)

Purification: flash column chromatography (Hexane/$CHCl_3$ = 5:1)

Yield: 6.7 mg, 57%, white solid

^{1}**H NMR** (600 MHz, $CDCl_3$) δ 9.45 (s, 1H), 9.26 (s, 1H), 8.99 (d, *J* = 8.4 Hz, 1H), 8.86 (d, *J* = 7.8 Hz, 1H), 8.20 (d, *J* = 8.4 Hz, 1H), 8.15 (t, *J* = 7.8 Hz, 2H), 8.05 (d, *J* = 7.8 Hz, 1H), 7.72 (td, *J* = 7.5, 1.4 Hz, 1H), 7.58–7.69 (m, 4H), 7.53 (t, *J* = 7.5 Hz, 1H)

^{13}**C NMR** (150 MHz, $CDCl_3$) δ 139.27, 138.74, 137.87, 132.24, 130.88, 129.85, 128.54, 128.38, 128.27, 128.23, 128.10, 127.73, 127.69, 127.64, 126.35, 125.91, 125.29, 125.17, 125.08, 124.58, 123.58, 123.29, 122.83, 122.37

HRMS (APCI, positive): Calcd for $C_{24}H_{15}S$ ($[M+H]^+$): 355.0889. Found: 335.0888.

1.8 Synthesis of 10,10'-bibenzo[*f*]tetraphene (7ba)

Synthesis of **6ba**

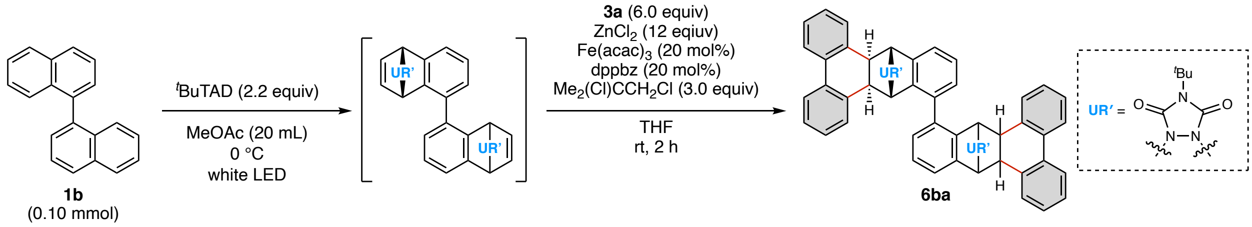

To a test tube containing a magnetic stirring bar were added ^{t}BuTAD (34.1 mg, 0.22 mmol, 2.2 equiv) and 1,1'-binaphtyl (**1b**, 25.4 mg, 0.10 mmol, 1.0 equiv). The tube was sealed with a septum and filled with N_2 gas. Then, methyl acetate (20 mL) was added at ambient temperature. The contents were sonicated to dissolve solids and then cooled to 0 °C. The resulting pink solution was stirred under irradiation with LED lights at 0 °C until the solution became colourless (approx. 2 h). After turning off the lights, the reaction mixture was transferred to a 50-mL two-necked round-bottomed flask, and the volatile was removed in vacuo at 0 °C. Then, the flask was filled with N_2 gas, and THF (3.0 mL) was added to dissolve the residue (**solution A**).

To another test tube containing a magnetic stirring bar were added dppbz (8.92 mg, 0.02 mmol, 20 mol%). The tube was sealed with a septum and filled with N_2 gas. Then, THF (2.0 mL), 1.9 M $ZnCl_2$ solution in 2-methyltetrahydrofuran (316 μL, 0.60 mmol, 6.0 equiv) and THF solution of **3a** (0.60 mmol, 6.0 equiv) were added in this order at ambient temperature. Another portion of 1.9 M $ZnCl_2$ solution in 2-methyltetrahydrofuran (316 μL, 0.60 mmol, 6.0 equiv) was added and then white precipitation formed. This mixture was stirred at ambient temperature for 30 min, and then cooled to 0 °C. At this temperature, 0.20 M $Fe(acac)_3$ solution in THF (100 μL, 0.02 mmol, 20 mol%) was added and resulting mixture was stirred for 5 min. Then, 1,2-dichloroisobutane (35 μL, 0.30 mmol, 3.0 equiv) and **solution A** was added to the tube and the mixture was stirred at ambient temperature for 2 h. The mixture was quenched with 1 M HCl aq. (ca. 10 mL) and extracted with dichloromethane (3×30 mL). The combined organic layers were dried with Na_2SO_4 , filtered and concentrated under reduced pressure. The crude product was purified by PTLC to yield tetraarylated products **6ba-1** and **6ba-2** (total 38.2 mg, 44%, **6ba-1/6ba-2** = 3:2). Each diastereomer was obtained as a mixture of rotational isomers (**6ba-1**: major/minor = 92:8, **6ba-2**: major/minor = 50:50). The relative configuration of **6ba-1** was determined by X-ray crystallographic analysis (Supplementary Fig. 2)

6ba-1

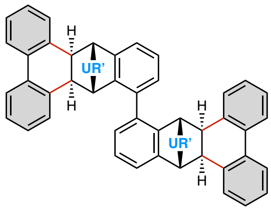

^{1}H NMR (600 MHz, $CDCl_3$) δ 7.91–7.95 (m, 0.08×4H), 7.92 (d, J = 7.8 Hz, 0.92×2H) 7.89 (d, J = 7.2 Hz, 0.92×2H), 7.62–7.73 (m, 0.08×6H), 7.72 (d, J = 6.6 Hz, 0.92×2H), 7.68 (t, J = 7.2 Hz, 0.92×2H), 7.64 (d, J = 6.6 Hz, 0.92×2H), 7.52–7.57 (m, 0.08×2H), 7.56 (d, J = 7.8 Hz, 0.92×2H), 7.31–7.40 (m, 0.08×6H), 7.38 (t, J = 6.9 Hz, 0.920×2H), 7.33 (t, J = 7.5 Hz, 0.92×2H), 7.27–7.31 (m, 0.08×4H), 7.21 (t, J = 7.5 Hz, 0.92×2H), 7.10 (t, J = 7.5 Hz, 0.92×2H), 6.63 (d, J = 7.2 Hz, 0.92×2H), 5.60 (dd, J = 4.5, 2.1 Hz, 0.08×4H), 5.57 (d, J = 1.8 Hz, 0.92×2H), 5.30 (d, J = 2.4 Hz, 0.92×2H), 3.96 (d, J = 10.8 Hz, 0.08×2H), 3.80 (d, J = 12.0 Hz, 0.08×2H), 3.72 (dd, J = 11.7, 2.1 Hz, 0.92×2H), 3.37 (dd, J = 12.3, 2.1 Hz, 0.92×2H), 1.33 (s, 0.92×18H), 1.16 (s, 0.08×18H)

^{13}C NMR (150 MHz, $CDCl_3$) δ 158.64, 158.26, 135.25, 133.56, 133.08, 132.06, 131.91, 131.73, 131.12, 129.25, 128.71 (2C), 128.62, 128.31, 127.88, 127.79, 123.94, 122.99, 122.89, 61.71, 58.68, 57.53, 38.28, 37.65, 27.81 (one sp^2 carbon signal was overlapping with others, and only signals of major rotational isomer were shown)

HRMS (ESI, positive): Calcd for $C_{56}H_{48}N_6O_4Na$ ($[M+Na]^+$): 891.3629. Found: 891.3608.

6ba-2

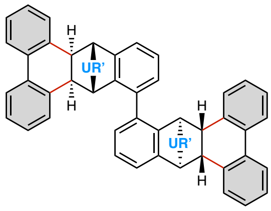

^{1}H NMR (600 MHz, $Cl_2CDCDCl_2$) δ 7.89–7.95 (m, 4H), 7.87 (d, J = 8.4 Hz, 0.50×2H), 7.66 (d, J = 7.2 Hz, 0.50×2H), 7.64 (d, J = 7.2 Hz, 0.50×2H), 7.56–7.61 (m, 2H), 7.50 (d, J = 7.8 Hz, 0.50×2H), 7.47 (d, J = 6.6 Hz, 0.50×2H), 7.25–7.40 (m, 6H + 0.50×2H), 7.19–7.24 (m, 0.50×4H), 7.06 (t, J = 7.5 Hz, 0.50×2H), 6.79 (d, J = 7.8 Hz, 0.50×2H), 5.52 (d, J = 2.4 Hz, 0.50×2H), 5.43 (d, J = 1.8 Hz, 0.50×2H), 5.42 (d, J = 1.8 Hz, 0.50×2H), 5.30 (d, J = 3.0 Hz, 0.50×2H), 3.90 (dd, J = 12.0, 2.4 Hz, 0.50×2H), 3.67–3.75 (m, 3H), 1.26 (s, 0.50×18H), 1.09 (s, 0.50×18H)

^{13}C NMR (150 MHz, $CDCl_3$) δ 158.71, 158.28, 158.19, 157.77, 136.80, 135.71, 134.14, 133.91, 133.77, 133.60, 132.57, 132.55, 132.35, 132.29, 132.00, 131.91, 131.83, 131.77, 130.54, 130.21, 129.59, 129.57, 128.97, 128.77, 128.69, 128.67, 128.60, 128.51, 128.14, 128.01, 127.88, 127.77, 127.67, 127.34, 123.95, 123.78, 122.93, 122.85, 122.69, 122.66, 62.57, 62.47, 58.09, 58.06, 57.46, 57.29, 38.02, 37.48, 37.20, 35.54,

27.82, 27.72

HRMS (ESI, positive): Calcd for $C_{56}H_{48}N_6O_4Na$ ($[M+Na]^+$): 891.3629. Found: 891.3614.

Synthesis of 10,10'-bibenzo[*f*]tetraphene (**7ba**)

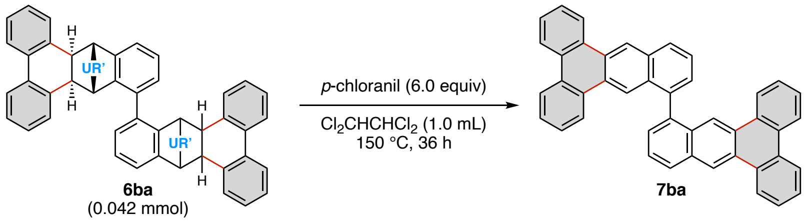

To a screw-capped tube containing a magnetic stirring bar were added **6ba** (36.5 mg, 0.042 mmol, 1.0 equiv), *p*-chloranil (62 mg, 0.25 mmol, 6.0 equiv) and 1,1,2,2-tetrachloroethane (1.0 mL) under air. The tube was sealed with a cap, and the resulting mixture was stirred at 150 °C for 36 h. Then, the reaction mixture was cooled to ambient temperature and diluted with chloroform (approx. 3 mL). To this mixture, hydrazine monohydrate (10 equiv) was added and the resulting mixture was stirred at ambient temperature for 15 min to quench remaining *p*-chloranil. The mixture was washed with 1 M NaOH aq. (approx. 10 mL) and extracted with chloroform (3×30 mL). The combined organic layers were dried with Na_2SO_4, filtered and concentrated under reduced pressure. The crude product was purified by flash column chromatography on silica gel (eluent: hexane/$CHCl_3$ = 5:1) to yield π-extended product **7ba** (8.7 mg, 38%) as a white solid.

^{1}H NMR (600 MHz, $CDCl_3$) δ 9.27 (s, 2H), 8.84–8.88 (m, 4H), 8.53 (d, *J* = 7.2 Hz, 2H), 8.46 (d, *J* = 8.4 Hz, 2H), 8.31 (d, *J* = 8.4 Hz, 2H), 7.98 (d, *J* = 8.4 Hz, 2H), 7.79 (t, *J* = 7.5 Hz, 2H), 7.72 (d, *J* = 6.0 Hz, 2H), 7.69 (t, *J* = 7.5 Hz, 2H), 7.64 (t, *J* = 7.5 Hz, 2H), 7.45 (t, *J* = 7.8 Hz, 2H), 7.28 (t, *J* = 7.8 Hz, 2H)

^{13}C NMR (150 MHz, $CDCl_3$) δ 138.40, 132.65, 131.97, 130.22, 130.07, 129.99, 129.93, 128.64, 128.51, 128.47, 128.36, 127.72, 127.48 (2C), 127.36, 125.71, 123.72, 123.64, 123.45, 123.19, 122.47, 120.88

HRMS (DART, positive): Calcd for $C_{44}H_{27}$ ($[M+H]^+$): 555.2113. Found: 555.2121.

1.9 Synthesis of tetrabenzo[*a,c,m,o*]pentaphene (12)

Synthesis of **6ha**

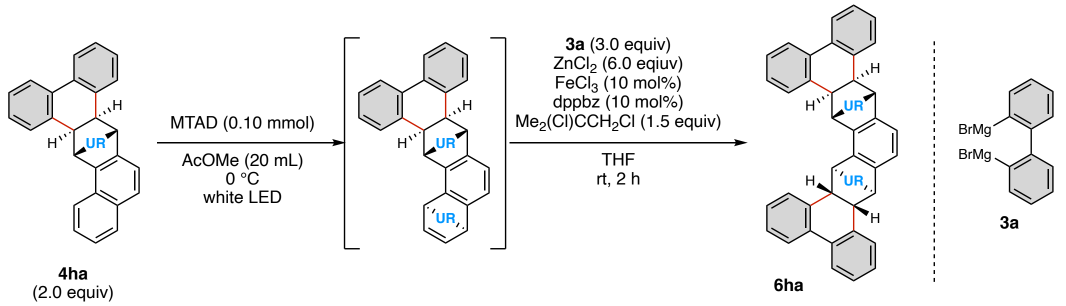

To a test tube containing a magnetic stirring bar were added MTAD (11.3 mg, 0.10 mmol, 1.0 equiv) and **4ha** (88.6 mg, 0.20 mmol, 2.0 equiv). The tube was sealed with a septum and filled with N_2 gas. Then, methyl acetate (20 mL) was added at ambient temperature. The contents were sonicated to dissolve solids as much as possible, and then cooled to 0 °C. The resulting pink mixture was stirred under irradiation with LED lights at 0 °C until the mixture became white (approx. 2 h). After turning off the lights, the mixture was transferred to a 50-mL two-necked round-bottomed flask, and the volatile was removed in vacuo at 0 °C. Then, the flask was filled with N_2 gas, and THF (2.5 mL) were added to dissolve the residue (**solution A**).

To another test tube containing a magnetic stirring bar was added dppbz (4.5 mg, 0.01 mmol, 10 mol%). The tube was sealed with a septum and filled with N_2 gas. Then, THF (1.0 mL), 1.9 M $ZnCl_2$ solution in 2-methyltetrahydrofuran (158 μL, 0.30 mmol, 3.0 equiv) and THF solution of **3a** (0.30 mmol, 3.0 equiv) were added in this order at ambient temperature. Another portion of 1.9 M $ZnCl_2$ solution in 2-methyltetrahydrofuran (158 μL, 0.30 mmol, 3.0 equiv) was added and then white precipitation formed. This mixture was stirred at ambient temperature for 30 min, and then cooled to 0 °C. At this temperature, 0.20 M $Fe(acac)_3$ solution in THF (50 μL, 0.01 mmol, 10 mol%) was added and the resulting mixture was stirred for 5 min. Then, 1,2-dichloroisobutane (17.5 μL, 0.15 mmol, 1.5 equiv) and **solution A** was added to the tube and the mixture was stirred at ambient temperature for 2 h. The mixture was quenched with 1 M HCl aq. (ca. 10 mL) and extracted with dichloromethane (3×30 mL). The combined organic layers were dried with Na_2SO_4, filtered and concentrated under reduced pressure. The crude product was purified by PTLC (eluent: $CHCl_3$) to yield tetraarylated product **6ha** (29.8 mg, 42%) as a white solid. The relative configuration of **6ha** was determined by X-ray crystallographic analysis (Supplementary Fig. 3)

^{1}H NMR (600 MHz, $CDCl_3$) δ 7.99–8.02 (m, 2H), 7.97 (d, *J* = 7.2 Hz, 2H), 7.70 (d, *J* = 7.8 Hz, 2H), 7.58 (s, 2H), 7.49–7.53 (m, 2H), 7.46 (t, *J* = 7.2 Hz, 2H), 7.33–7.41 (m, 6H), 5.79 (d, *J* = 2.4 Hz, 2H), 5.61 (d, *J* = 3.0 Hz, 2H), 3.71 (dd, *J* = 12.0, 2.4 Hz, 2H), 3.46 (dd, *J* = 12.0, 1.8 Hz, 2H), 2.69 (s, 6H)

^{13}C NMR (150 MHz, $CDCl_3$) δ 158.59, 157.51, 135.47, 132.11, 131.92, 131.76, 131.70, 130.19, 129.77, 129.15, 128.80, 128.61, 128.14, 127.97, 124.30, 123.04, 122.77, 61.47, 58.24, 38.25, 36.66, 25.28

HRMS (ESI, positive): Calcd for $C_{44}H_{33}O_4N_6$ ($[M+H]^+$): 709.2558. Found: 709.2553.

Synthesis of tetrabenzo[*a*,*c*,*m*,*o*]pentaphene (**12**)

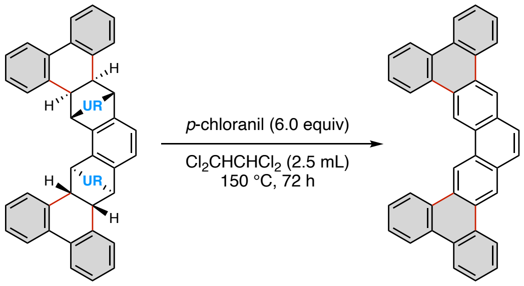

6ha
(0.25 mmol)

12

To a screw-capped tube containing a magnetic stirring bar were added compound **6ha** (177 mg, 0.25 mmol, 1.0 equiv), *p*-chloranil (369 mg, 1.5 mmol, 6.0 equiv) and 1,1,2,2-tetrachloroethane (2.5 mL) under air. The tube was sealed with a cap, and the resulting mixture was stirred at 150 °C for 72 h. Then, the reaction mixture was cooled to ambient temperature and poured into a 50-mL round-bottomed flask. After removing solvent under reduced pressure, ethyl acetate (15 mL) and hydrazine monohydrate (150 mg, 3.0 mmol, 12.0 equiv) was added. Then, the resulting mixture was stirred at ambient temperature for 15 min to quench remaining *p*-chloranil. 1 M NaOH aq. (approx. 15 mL) was added to the mixture and the precipitates were filtrated and washed with 1 M NaOH aq., water, acetone and ethyl acetate. The crude product was extracted from the solid with boiling 1,1,2,2-tetrachloroethane. The crude product was further purified by flash column chromatography on silica gel (eluent: $CHCl_3$) and then washed with small amount of methanol to yield product **12** as a pale brown solid (46.7 mg, 39%).

^{1}H NMR (600 MHz, $Cl_2CDCDCl_2$) δ 10.12 (s, 2H), 9.02–9.08 (m, 4H), 8.80 (d, *J* = 7.2 Hz, 2H), 8.66 (d, *J* = 7.8 Hz, 2H), 8.64 (d, *J* = 7.2 Hz, 2H), 7.96 (s, 2H), 7.83 (t, *J* = 7.2 Hz, 2H), 7.66–7.76 (m, 6H)

^{13}C NMR (150 MHz, $Cl_2CDCDCl_2$) δ 131.31, 130.19, 130.09, 130.04, 129.70, 129.26, 129.02, 128.64, 127.53 (2C), 127.51, 127.45, 127.38, 123.49 (2C), 123.46, 123.34, 122.84, 116.95 (two sp^2 carbon signals were overlapping with others)

HRMS (APCI, positive): Calcd for $C_{38}H_{23}$ ($[M+H]^+$): 479.1794. Found: 479.1797.

1.10 Synthesis of 2,7-di-tert-butyldibenzo[*j*,*s*]phenanthro[9,10-*b*]picene (11)

Synthesis of **S1**

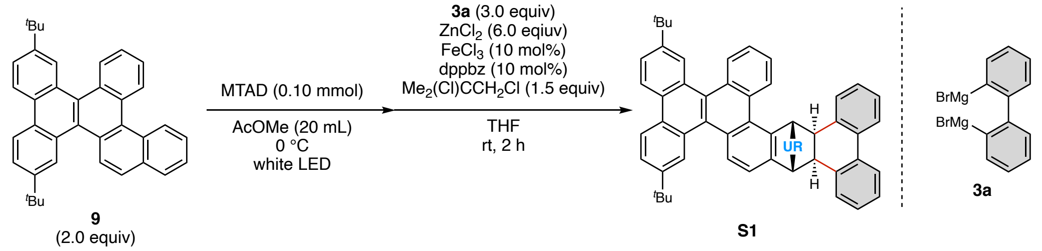

To a test tube containing a magnetic stirring bar were added MTAD (11.3 mg, 0.10 mmol, 1.0 equiv) and **9** (98 mg, 0.20 mmol, 2.0 equiv). The tube was sealed with a septum and filled with N_2 gas. Then, methyl acetate (20 mL) was added at ambient temperature. The contents were sonicated to dissolve solids, and then cooled to 0 °C. The resulting pink solution was stirred under irradiation with LED lights at 0 °C until the solution became colourless (approx. 2 h). After turning off the lights, the mixture was transferred to a 50-mL two necked round-bottomed flask, and the volatile was removed in vacuo at 0 °C. Then, the flask was filled with N_2 gas, and THF (2.5 mL) were added to dissolve the residue (**solution A**).

To another test tube containing a magnetic stirring bar were added dppbz (4.5 mg, 0.01 mmol, 10 mol%). The tube was sealed with a septum and filled with N_2 gas. Then, THF (1.0 mL), 1.9 M $ZnCl_2$ solution in 2-methyltetrahydrofuran (158 μL, 0.30 mmol, 3.0 equiv) and THF solution of **3a** (0.30 mmol, 3.0 equiv) were added in this order at ambient temperature. Another portion of 1.9 M 2-methyltetrahydrofuran solution of $ZnCl_2$ (158 μL, 0.30 mmol, 3.0 equiv) was added and then white precipitation formed. This mixture was stirred at ambient temperature for 30 min, and then cooled to 0 °C. At this temperature, 0.20 M $Fe(acac)_3$ solution in THF (50 μL, 0.01 mmol, 10 mol%) was added and the resulting mixture was stirred for 5 min. Then, 1,2-dichloroisobutane (17.5 μL, 0.15 mmol, 1.5 equiv) and **solution A** was added to the tube and the mixture was stirred at ambient temperature for 2 h. The mixture was quenched with 1 M HCl aq. (approx. 10 mL) and extracted with dichloromethane (3×30 mL). The combined organic layers were dried with Na_2SO_4, filtered and concentrated under reduced pressure. The crude product was purified by PTLC (eluent: $CHCl_3$) followed by GPC to yield diarylated product **S1** (23.5 mg, 31%) as a white solid.

^{1}H NMR (600 MHz, $CDCl_3$) δ 8.87 (bs, 1H), 8.71–8.75 (m, 2H), 8.69 (d, J = 8.4 Hz, 1H), 8.58–8.62 (m, 3H), 8.01 (d, J = 7.8 Hz, 1H), 8.01 (d, 7.2 Hz, 1H), 7.81 (t, J = 7.5 Hz, 1H), 7.70–7.78 (m, 4H), 7.60 (bs, 1H), 7.57 (d, J = 7.8 Hz, 1H), 7.35–7.46 (m, 4H), 6.99 (d, J = 1.8 Hz, 1H), 5.67 (d, J = 2.4 Hz, 1H), 3.92 (bs, 1H), 3.76 (bs, 1H), 2.68 (s, 3H), 1.48 (s, 9H), 1.47 (s, 9H)

^{13}C NMR (150 MHz, $CDCl_3$) δ 157.06, 156.61, 149.36, 149.10, 134.31, 132.64, 132.31, 132.16, 132.11, 132.06, 131.33, 131.23, 129.38, 129.04, 128.83, 128.78, 128.69, 128.52, 128.06, 127.98, 127.94, 127.86, 127.81, 127.75, 127.05, 126.38, 125.05, 125.02, 124.94, 124.65, 123.31, 123.18, 123.01, 122.95, 122.60,

61.91, 60.08, 38.10, 37.78, 35.10, 35.05, 31.44 (2C), 25.20 (five sp^2 carbon signals were overlapping with others)

HRMS (ESI, positive): Calcd for $C_{53}H_{46}N_3O_2$ ($[M+H]^+$): 756.3585. Found: 756.3584.

Synthesis of 2,7-di-*tert*-butyldibenzo[*j*,*s*]phenanthro[9,10-*b*]picene (**11**)

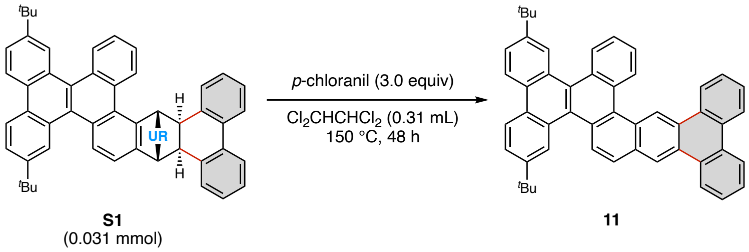

To a screw-capped tube containing a magnetic stirring bar were added diarylated compound **S1** (23.5 mg, 0.031 mmol, 1.0 equiv) and *p*-chloranil (22.9 mg, 0.093 mmol, 3.0 equiv). Then, 1,1,2,2-tetrachloroethane (0.31 mL) was added under air. The tube was sealed with a cap, and the resulting mixture was stirred at 150 °C for 48 h. Then, the reaction mixture was cooled to ambient temperature and diluted with chloroform (ca 3 mL). To this mixture, hydrazine monohydrate (7.8 mg, 0.155 mmol, 5.0 equiv) was added and the resulting mixture was stirred at ambient temperature for 15 min to quench remaining *p*-chloranil. The mixture was washed with 1 M NaOH aq. (approx. 10 mL) and extracted with chloroform (3×30 mL). The combined organic layers were dried with Na_2SO_4, filtered and concentrated under reduced pressure. The crude product was purified by flash column chromatography on silica gel (eluent: hexane/$CHCl_3$ = 5:1) to yield **11** (15.7 mg, 79% yield) as a yellow solid.

^{1}H NMR (600 MHz, $CDCl_3$) δ 10.45 (s, 1H), 9.20 (s, 1H), 9.18 (d, J = 7.8 Hz, 1H), 8.82–8.88 (m, 4H), 8.81 (s, 1H), 8.76 (s, 1H), 8.61–8.68 (m, 4H), 8.26 (d, J = 9.0 Hz, 1H), 7.82 (t, J = 7.5 Hz, 1H), 7.66–7.78 (m, 7H), 1.52 (s, 9H), 1.50 (s, 9H)

^{13}C NMR (150 MHz, $CDCl_3$) δ149.26, 148.77, 131.78, 130.85, 130.55, 130.29, 130.23, 130.05, 129.93, 129.16, 129.00, 128.98, 128.81, 128.70, 128.54, 128.46, 128.39, 128.15, 128.10, 127.87, 127.82, 127.73, 127.67, 127.56, 127.52, 127.13, 126.66, 126.16, 126.01, 125.69, 125.22, 124.76, 124.45, 123.87, 123.81, 123.58, 123.49, 123.30, 123.15, 122.71, 122.37, 35.10 (2C), 31.50, 31.48 (one sp^2 carbon signal was overlapping with others)

HRMS (DART, positive): Calcd for $C_{50}H_{41}$ ($[M+H]^+$): 641.3208. Found: 641.3208.

1.11 Regioselectivity and limitation

When a PAH template has two or more distinguishable *M*-regions, the DAPEX protocol potentially gives *M*-APEX products as a mixture of regioisomers. To obtain insights into the regioselectivity and limitation of the protocol, dearomatization and subsequent annulative diarylation of several PAHs containing multiple *M*-regions were conducted following the **procedure A** described above, using 2.0 equivalents of PAH templates. The results are summarized in Supplementary Fig. 1. As also described in the main text, when benzo[*g*]chrysene (**1p**) was employed as a starting material under the standard conditions (T = 0 °C), the desired product was obtained as a mixture of regioisomers (**4pa**, **4pa′** and **4pa″**) in 48% combined yield (Supplementary Fig. 1a). The ratio of regioisomers was determined by ^{1}H NMR analysis, and calculated to be 67:20:13. When the dearomatization of **1p** was conducted at 25 °C (T = 25 °C), the regioselectivity was improved to 88% whereas the combined yield did not significantly change (43%). The reaction of dibenzo[*f,s*]picene (**1q**) also afforded the mixture of diarylated products **4qa**, **4qa′**, **4qa″** and **4qa‴** in 43% yield with 83% selectivity of **4qa**. The regioselectivity could also be improved up to 92% when the dearomatization was conducted at 25 °C, whereas the combined yield was almost the same (41%). Furthermore, as showcased by the reaction of **9**, introduction of *tert*-butyl groups on C2 and C7 position of **1q** completely suppressed the Diels–Alder reaction not only at C1–C4 and C5–C8 positions but also C15–C18 position, and compound **S1** was obtained as an exclusive product.

On the other hand, when tetraphene (**1r**) was used as a starting template, the dearomative activation of **1r** was not very successful owing to the Diels–Alder reaction at the internal benzene ring (C7–C12 position), and the cycloadduct **2r″** was obtained as a major product (Supplementary Fig. 1b). The desired diarylated products was obtained as a mixture of regioisomers **4ra** and **4ra′** albeit with low yield (9%). Notably, the Diels–Alder reaction at the sterically less hindered C8–C11 position was much more favored than the C1–C4 position (**4ra**/**4ra′** = 96:4).

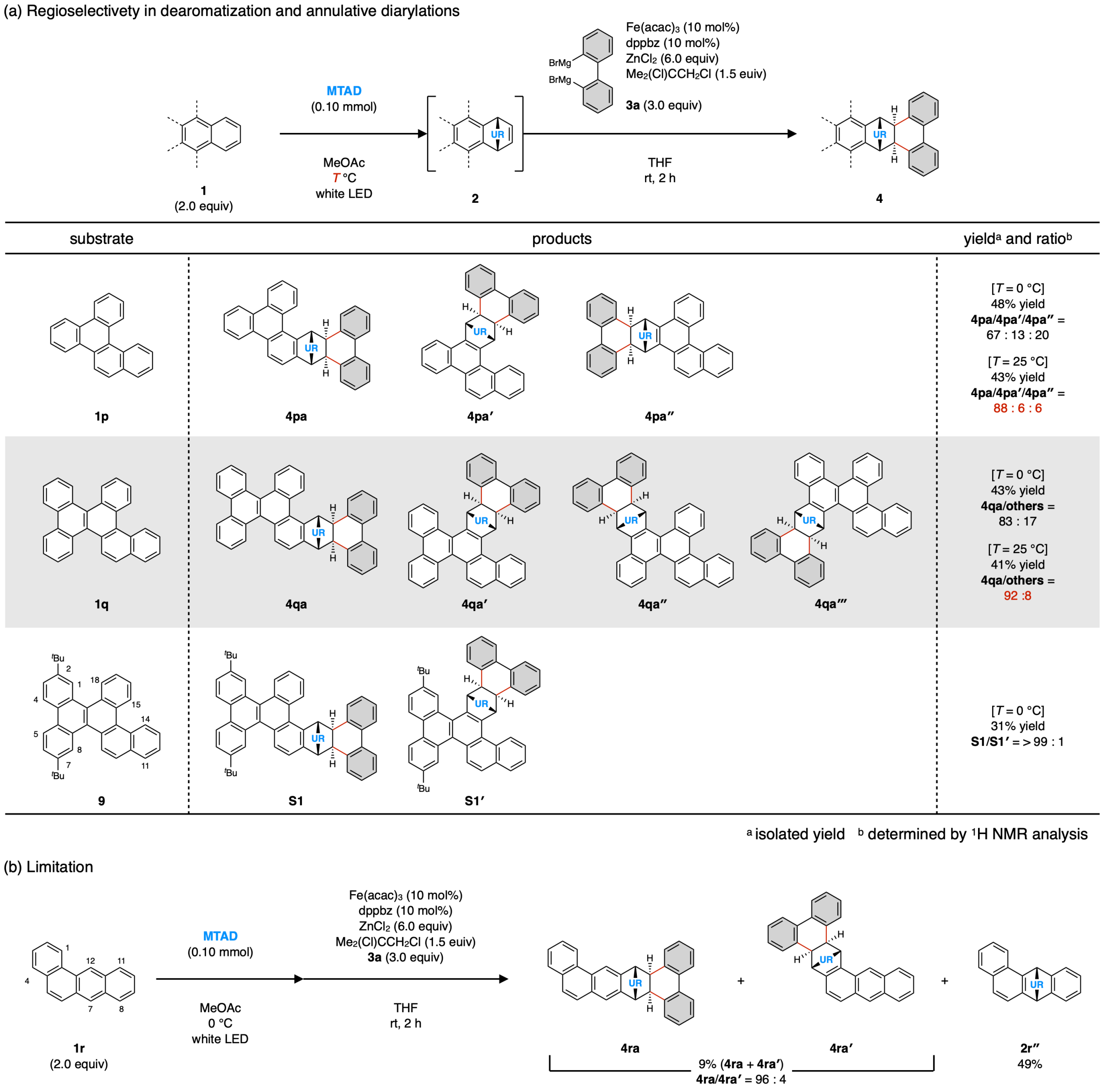

Supplementary Fig. 1. Dearomatization and following annulative diarylation of PAHs containing multiple distinguishable *M*-regions.

Results and characterization data

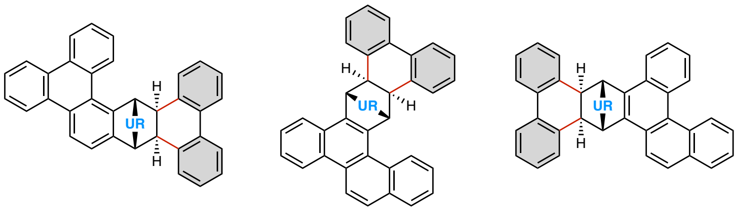

4pa **4pa′** **4pa″**

Purification: PTLC ($CHCl_3$)

Combined yield and ratio: T = 0 °C: 26.2 mg (48%, white solid), **4pa**/**4pa′**/**4pa″** = 67:13:20

T = 25 °C: 23.6 mg (43%, white solid), **4pa**/**4pa′**/**4pa″** = 88:6:6

^{1}H NMR (600 MHz, $CDCl_3$) δ 8.69 (t, J = 7.5 Hz, 2H), 8.62–8.65 (m, 1H), 8.57–8.62 (m, 2H), 8.01(t, J = 7.5 Hz, 2H), 7.83 (td, J = 7.5, 1.2 Hz, 1H), 7.78 (td, J = 7.5, 1.2 Hz, 1H), 7.72 (d, J = 7.8 Hz, 1H), 7.66–7.70 (m, 3H), 7.57 (d, J = 7.2 Hz, 1H), 7.35–7.45 (m, 4H), 6.83 (d, J = 3.0 Hz, 1H), 5.61 (d, J = 2.4 Hz, 1H), 4.14 (dd, J = 11.7, 2.7 Hz, 1H), 3.84 (dd, J = 12.6, 2.4 Hz, 1H), 2.58 (s, 3H) (only signals of **4pa** are shown)

^{13}C NMR (150 MHz, $CDCl_3$) δ 157.28, 156.37, 134.94, 132.62, 132.25, 132.23, 132.05, 132.03, 131.48, 131.43, 130.19, 129.58, 129.49, 128.84, 128.68 (2C), 128.35, 128.07, 127.99 (2C), 127.91, 127.74, 127.08, 126.88, 124.06, 123.39, 123.32, 123.19, 123.06, 122.93, 122.64, 62.28, 59.97, 38.17, 36.90, 25.07 (one sp^2 carbon signal was overlapping with others, and only signals of **4pa** are shown)

HRMS (ESI, positive): Calcd for $C_{37}H_{25}N_3O_2Na$ ($[M+Na]^+$): 566.1839. Found: 566.1842.

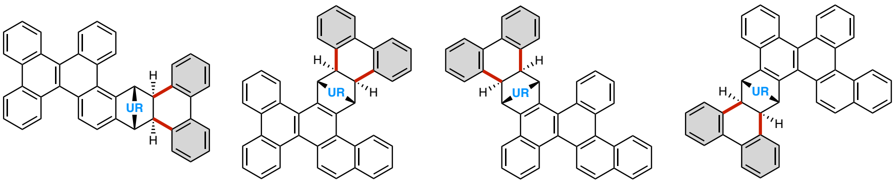

4qa **4qa′** **4qa″** **4qa‴**

Purification: PTLC ($CHCl_3$)

Combined yield and ratio: T = 0 °C: 27.5 mg (43%, white solid), **4qa**/**4qa′**/(**4qa″**+**4qa‴**) = 83:7:10

T = 25 °C: 26.7 mg (41%, white solid), **4qa**/**4qa′**/(**4qa″**+**4qa‴**) = 92:4:4

^{1}H NMR (600 MHz, $CDCl_3$) δ 8.87 (bs, 1H), 8.67–8.75 (m, 5H), 8.59 (dd, J = 8.4, 0.6 Hz, 1H), 7.97–8.02 (m, 2H), 7.82 (t, J = 7.5, 1H), 7.63–7.75 (m, 6H), 7.54–7.62 (m, 2H), 7.34–7.45 (m, 4H), 6.97 (d, J = 2.4 Hz, 1H), 5.66 (d, J = 2.4 Hz, 1H), 3.89 (bs, 1H), 3.73 (d, J = 11.4 Hz, 1H), 2.65 (s, 3H) (only signals of **4qa** are shown)

^{13}C NMR (150 MHz, $CDCl_3$) δ 157.06, 156.64, 134.46, 132.55, 132.24, 132.11, 132.08, 132.02, 131.13, 131.11, 131.01, 130.92, 129.40, 129.02 (2C), 128.92, 128.83, 128.80, 128.65, 128.57 (2C), 128.55, 128.43, 128.38, 127.98, 127.94, 127.90, 127.79, 127.29, 127.05 (2C), 126.91, 126.73 (2C), 126.56, 123.78, 123.61,

123.01, 122.93, 122.69, 61.91, 60.00, 38.01, 37.70, 25.17 (only signals of **4qa** are shown)

HRMS (ESI, positive): Calcd for $C_{45}H_{29}N_3O_2Na$ ($[M+Na]^+$): 666.2152. Found: 666.2150.

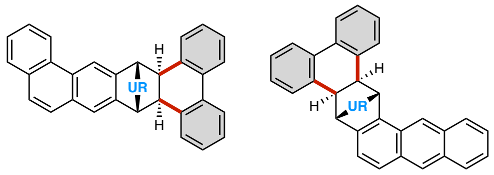

4ra **4ra′**

Purification: PTLC ($CHCl_3$)

Combined yield and ratio: T = 0 °C: 4.6 mg (9%, white solid), **4ra**/**4ra′** = 96:4

^{1}H NMR (600 MHz, $CDCl_3$) δ 8.78 (s, 1H), 8.73 (d, J = 8.4 Hz, 1H), 8.00 (d, J = 7.8 Hz, 2H), 7.99 (s, 1H), 7.94 (d, J = 6.6 Hz, 1H), 7.85 (d, J = 9.0 Hz, 1H), 7.81 (d, J = 9.0 Hz, 1H), 7.73 (ddd, J = 8.1, 6.8, 1.4 Hz, 1H), 7.67 (t, J = 7.2 Hz, 1H), 7.59 (d, J = 7.2 Hz, 1H), 7.56 (d, J = 7.8 Hz, 1H), 7.34–7.44 (m, 4H), 5.74 (d, J = 1.8 Hz, 1H), 5.68 (d, J = 1.8 Hz, 1H), 3.81 (t, J = 14.1 Hz, 2H), 2.63 (s, 3H) (only signals of **4ra** were shown)

^{13}C NMR (150 MHz, $CDCl_3$) δ 157.13 (2C), 133.15, 133.11, 132.27, 132.25, 132.20, 131.98, 130.62, 130.07, 129.31, 129.26, 128.79, 128.66 (2C), 128.10, 127.95 (2C), 127.24, 127.07, 126.62, 123.48, 122.95 (2C), 122.80, 117.99, 62.31, 61.67, 38.33, 38.18, 25.09 (two sp^2 carbon signals were overlapping with others, and only signals of **4ra** isomer are shown)

HRMS (ESI, positive): Calcd for $C_{33}H_{23}N_3O_2Na$ ($[M+Na]^+$): 516.1682. Found: 516.1684.

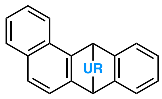

2r″

Purification: PTLC ($CHCl_3$)

Yield: T = 0 °C: 16.8 mg (49%, white solid)

^{1}H NMR (600 MHz, acetone-d_6) δ 8.51 (d, J = 7.8 Hz, 1H), 7.95 (d, J = 8.4 Hz, 1H), 7.90 (d, J = 8.4 Hz, 1H), 7.71–7.76 (m, 2H), 7.67 (ddd, J = 8.6, 7.1, 1.4 Hz, 1H), 7.61–7.64 (m, 1H), 7.55 (ddd, J = 8.3, 7.1, 1.1 Hz, 1H), 7.27–7.33 (m, 2H), 7.25 (s, 1H), 6.57 (s, 1H), 2.66 (s, 3H)

^{13}C NMR (150 MHz, acetone-d_6) δ 158.70, 158.58, 139.10, 138.57, 136.17, 134.78, 133.88, 129.56, 129.04, 128.96, 128.77, 128.68, 128.10, 127.24, 124.95, 124.87, 123.45, 122.93, 61.30, 56.78, 25.44

HRMS (ESI, positive): Calcd for $C_{21}H_{15}N_3O_2Na$ ($[M+Na]^+$): 364.1056. Found: 364.1056.

1.12 Synthesis of nanographenes by *K*- and *bay*-APEX reactions

2,7-Di-*tert*-butyldibenzo[*f*,*s*]picene (**9**)

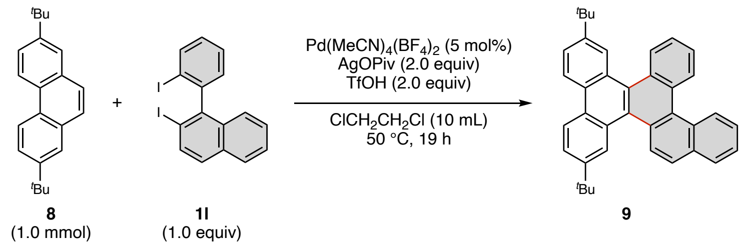

To a Schlenk tube containing a magnetic stirring bar were added 2,7-di-*tert*-butylphenanthrene (**8**) (290 mg, 1.0 mmol, 1.0 equiv), diiodobiaryl **1l** (456 mg, 1.0 mmol, 1.0 equiv) and AgOPiv (418 mg, 2.0 mmol, 2.0 equiv). The tube was taken into a glove box. Then, $Pd(MeCN)_4(BF_4)_2$ (22.2 mg, 0.05 mmol, 5 mol%) was added, and the tube was sealed with a septum. The tube was taken out from the glove box, and then 1,2-dichloroethane (10 mL) and TfOH (178 μL, 2.0 mmol, 2.0 equiv) were added by a syringe. After stirring at 50 °C for 19 h, the reaction mixture was cooled to ambient temperature, and then passed through a short pad of silica gel (eluent: $CHCl_3$). After the organic solvent was removed under reduced pressure, the residue was purified by flash column chromatography on silica gel (eluent: hexane/$CHCl_3$ = 10:1) to yield desired product **9** (346.2 mg, 71%) as a white solid.

^{1}H NMR (600 MHz, $CDCl_3$) δ 9.16 (d, J = 7.8 Hz, 1H), 8.95–8.99 (m, 1H), 8.73–8.78 (m, 3H), 8.72 (d, J = 1.8 Hz, 1H), 8.61 (d, J = 8.4 Hz, 2H), 8.05 (d, J = 9.0 Hz, 1H), 8.03 (d, J = 7.2 Hz, 1H), 7.74 (dt, J = 8.6, 2.6 Hz, 2H), 7.59–7.71 (m, 4H), 1.483 (s, 9H), 1.475 (s, 9H)

^{13}C NMR (150 MHz, $CDCl_3$) δ 149.16, 148.70, 132.91, 130.67, 130.07, 129.61, 129.30, 128.89, 128.72, 128.50, 128.41 (2C), 128.31, 127.93, 127.88 (2C), 127.85, 127.70, 127.19, 126.56, 126.27, 125.97, 125.63, 125.57, 125.48, 125.12, 124.63, 124.32, 123.24, 123.08, 35.05 (2C), 31.46 (2C)

HRMS (DART, positive): Calcd for $C_{38}H_{35}$ ($[M+H]^+$): 491.2739. Found: 491.2738.

2,7-Di-*tert*-butyldibenzo[*j*,*s*]phenanthro[9,10-*b*]picene (**11**)

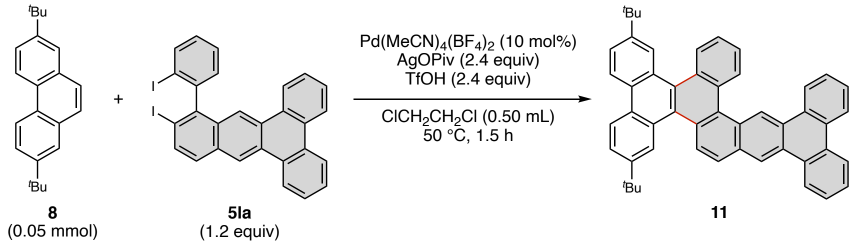

To a screw-capped tube containing a magnetic stirring bar were added 2,7-di-*tert*-butylphenanthrene (**8**) (14.5 mg, 0.05 mmol, 1.0 equiv), diiodobiaryl **5la** (36.4 mg, 0.06 mmol, 1.2 equiv), AgOPiv (25.0 mg, 0.12 mol, 2.4 equiv). The tube was taken into a glove box. Then, $Pd(MeCN)_4(BF_4)_2$ (2.2 mg, 5 μmol, 10 mol%)

was added, and the tube was sealed with a cap. The tube was taken out from the glove box, and then 1,2-dichloroethane (0.5 mL) and TfOH (11 µL, 0.12 mmol, 2.4 equiv) were added by a syringe. After stirring at 50 °C for 1.5 h, the reaction mixture was cooled to ambient temperature, and then passed through a short pad of silica gel (eluent: $CHCl_3$). After the organic solvent was removed under reduced pressure, the residue was purified by GPC to yield **11** (7.7 mg, 24%) as a yellow solid. The NMR spectra of product were identical to those described above.

Pentabenzo[*a*,*c*,*f*,*k*,*m*]tetraphene (**10**)

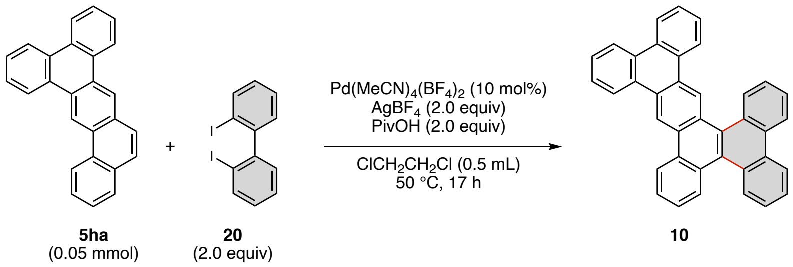

To a screw-capped tube containing a magnetic stirring bar were added **5ha** (16.4 mg, 0.05 mmol, 1.0 equiv), diiodobiphenyl **20** (40.6 mg, 0.10 mmol, 2.0 equiv) and pivalic acid (10.2 mg, 0.10 mmol, 2.0 equiv). The tube was taken into a glove box. Then, $AgBF_4$ (19.5 mg, 0.10 mmol, 2.0 equiv) and $Pd(MeCN)_4(BF_4)_2$ (2.2 mg, 0.005 mmol, 10 mol%) were added. The tube was sealed with a cap and taken out from the glove box. Then, 1,2-dichloroethane (0.50 mL) was added by a syringe. After stirring at 50 °C for 17 h, the reaction mixture was cooled to ambient temperature, and then passed through a short pad of silica gel (eluent: $CHCl_3$). After the organic solvent was removed under reduced pressure, the residue was purified by flash column chromatography on silica gel (eluent: hexane/$CHCl_3$ = 5:1) and then PTLC (eluent: hexane/Et_2O = 10:1) to yield desired product **10** (12.0 mg, 50%) as a white solid.

^{1}H NMR (600 MHz, $CDCl_3$) δ 9.94 (s, 1H), 9.93 (s, 1H), 8.98–9.02 (m, 2H), 8.90–8.95 (m, 1H), 8.71–8.82 (m, 4H), 8.63–8.70 (m, 3H), 7.75–7.81 (m, 4H), 7.65–7.74 (m, 6H)

^{13}C NMR (150 MHz, $CDCl_3$) δ 131.17, 131.09, 130.96, 130.23, 130.14, 130.04, 130.02, 129.97, 129.80, 129.40, 129.22 (2C), 128.82, 128.68, 128.47, 128.43, 128.26, 127.97, 127.75, 127.62, 127.59, 127.52, 127.49, 126.97, 126.91, 126.87, 126.72, 126.66 (2C), 123.87, 123.85, 123.68, 123.65, 123.59, 123.55 (2C), 123.49, 117.89

HRMS (DART, positive): Calcd for $C_{38}H_{23}$ ($[M+H]^+$): 479.1800. Found: 479.1793.

7,12-Di-*tert*-butyltetrabenzo[*a*,*c*,*m*,*o*]phenanthro[9,10-*h*]pentaphene (**13**)

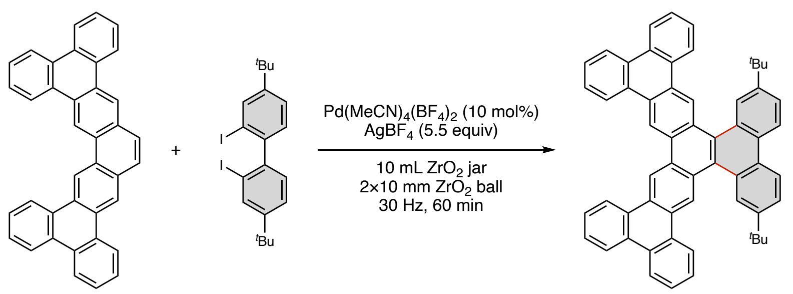

12
(0.05 mmol)

21
(2.0 equiv)

13

To a ball-milling vessel (ZrO_2, 10 mL) loaded with two grinding balls (ZrO_2, diameter 10 mm) were added **12** (23.9 mg, 0.05 mmol, 1.0 equiv) and **21** (77.7 mg, 0.10 mmol, 2.0 equiv). The vessel was taken into a glove box. Then, $AgBF_4$ (53.5 mg, 0.275 mmol, 5.5 equiv) and $Pd(MeCN)_4(BF_4)_2$ (2.2 mg, 0.005 mmol, 10 mol%) were added and the vessel was closed. Then, the vessel was taken out from the glove box, and placed in the ball mill (Retch MM400). The vessel was shaken at the rate of 30 Hz for 60 min at ambient temperature. Then, the contents were dissolved with $CHCl_3$ and transferred to another flask. The sticking residue inside the ball mill vessel was collected by shaking with silica gel at the rate of 30 Hz for 10 min. Then $CHCl_3$ solution and collected silica gel were combined, passed through a short pad of silica gel (eluent: $CHCl_3$). After removing the solvent, the crude mixture was purified by GPC to give **13** (10.5 mg, 28%) as a yellow solid.

^{1}H NMR (600 MHz, $CDCl_3$) δ 10.09 (s, 2H), 9.98 (s, 2H), 9.11 (d, J = 7.8 Hz, 2H), 9.01 (d, J = 1.8 Hz, 2H), 8.75 (d, J = 8.4 Hz, 4H), 8.62–8.68 (m, 4H), 7.82–7.87 (m, 4H), 7.72 (t, J = 7.5 Hz, 2H), 7.59–7.66 (m, 4H), 1.52 (s, 18H)

^{13}C NMR (150 MHz, $CDCl_3$) δ 149.38, 130.23, 130.14 (2C), 130.05, 129.96, 129.21, 129.02, 128.96, 128.69, 128.60, 128.29, 127.62, 127.54, 127.46, 127.38, 125.00, 124.74, 123.85, 123.70, 123.53 (2C), 123.44, 123.40, 118.06, 35.14, 31.62

HRMS (DART, positive): Calcd for $C_{58}H_{45}$ ($[M+H]^+$): 741.3521. Found: 741.3516.

3,10,15,28-Tetra-*tert*-butyltetrabenzo[*a*,*c*,*f*,*j*]triphenyleno[2,3-*s*]picene (**15**)

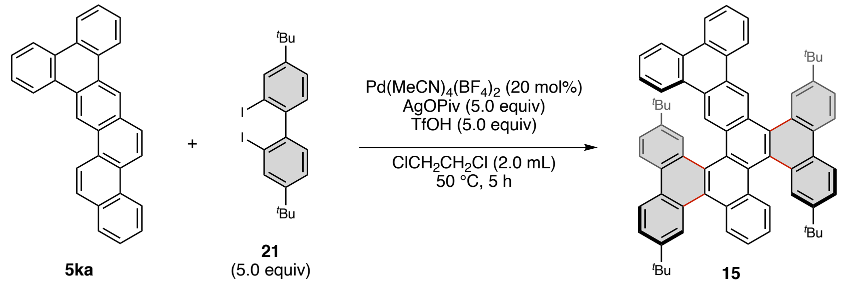

5ka

21
(5.0 equiv)

15

To a screw-capped tube containing a magnetic stirring bar were added **5ka** (37.8 mg, 0.10 mmol, 1.0 equiv), diiodobiphenyl **21** (259 mg, 0.50 mmol, 5.0 equiv) and AgOPiv (104.5 mg, 0.50 mol, 5.0 equiv). The

tube was taken into a glove box. Then, $Pd(MeCN)_4(BF_4)_2$ (8.8 mg, 20 μmol, 20 mol%) was added, and the tube was sealed with a cap. The tube was taken out form the glove box, and then 1,2-dichloroethane (2.0 mL) and TfOH (44.5 μL, 0.50 mmol, 5.0 equiv) were added by a syringe. After stirring at 50 °C for 5 h, the reaction mixture was cooled to ambient temperature, and then passed through a short pad of silica gel (eluent: $CHCl_3$). After the organic solvent was removed under reduced pressure, the residue was purified by GPC to yield **15** (37.8 mg, 42%, yellow solid) as a racemic mixture.

^{1}H NMR (600 MHz, $CDCl_3$) δ 10.03 (s, 1H), 9.53 (s, 1H), 9.24 (d, J = 1.8 Hz, 1H), 8.97 (d, J = 1.8 Hz, 1H), 8.84 (d, J = 8.4 Hz, 1H), 8.77 (d, J = 8.4 Hz, 2H), 8.72–8.76 (m, 1H), 8.67 (d, J = 8.4 Hz, 1H), 8.58 (d, J = 8.4 Hz, 2H), 8.51 (d, J = 7.8 Hz, 1H), 8.24 (d, J = 1.2 Hz, 1H), 8.14 (d, J = 7.8 Hz, 1H), 8.07 (d, J = 2.4 Hz, 1H), 7.95 (dd, J = 8.4, 2.4 Hz, 1H), 7.91 (dd, J = 8.4, 1.8 Hz, 1H), 7.59–7.66 (m, 4H), 7.48–7.55 (m, 3H), 7.34 (t, J = 7.8 Hz, 1H), 7.19 (t, J = 7.2 Hz, 1H), 1.69 (s, 9H), 1.61(s, 9H), 1.18 (s, 9H), 0.80 (s, 9H)

^{13}C NMR (150 MHz, $CDCl_3$) δ 149.35, 149.07, 147.75, 147.60, 132.15, 131.95, 131.41 (2C), 130.79, 130.65, 130.38, 130.32, 130.26, 130.22, 130.07, 129.94, 129.81, 129.57, 129.27, 128.88, 128.72, 128.69, 128.23, 127.70, 127.32, 127.27, 127.20 (2C), 127.11, 126.81, 126.69, 126.49, 126.46 (2C), 126.26, 126.07, 125.99, 125.63, 125.54, 124.75 (2C), 124.56, 124.44, 124.40, 123.70, 123.46, 123.40, 123.28, 123.07, 123.00, 122.97, 122.35, 35.25, 35.15, 34.64, 34.44, 31.80, 31.59, 30.96, 30.73 (two sp^2 carbon signals were overlapping with others)

HRMS (APCI, positive): Calcd for $C_{70}H_{63}$ ($[M+H]^+$): 903.4924. Found: 903.4920.

10,15-Di-*tert*-butylbenzo[*fg*]dinaphtho[1,2,3-*ij*:1',2',3',4'-*rst*]pentaphene (**16**)

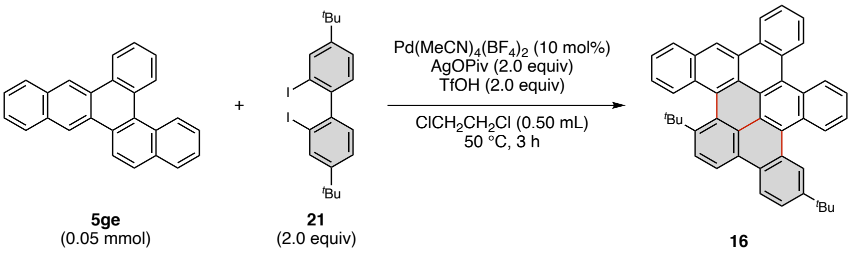

To a screw-capped tube containing a magnetic stirring bar were added **5ge** (16.4 mg, 0.05 mmol, 1.0 equiv), diiodobiphenyl **21** (51.8 mg, 0.10 mmol, 2.0 equiv) and AgOPiv (20.8 mg, 0.10 mol, 2.0 equiv). The tube was taken into a glove box. Then, $Pd(MeCN)_4(BF_4)_2$ (2.2 mg, 5 μmol, 10 mol%) was added, and the tube was sealed with a cap. The tube was taken out from the glove box, and then 1,2-dichloroethane (0.5 mL) and TfOH (9 μL, 0.10 mmol, 2.0 equiv) were added by a syringe. After stirring at 50 °C for 3 h, the reaction mixture was cooled to ambient temperature, and then passed through a short pad of silica gel (eluent: $CHCl_3$). After the organic solvent was removed under reduced pressure, the residue was purified by GPC to yield **16** (4.0 mg, 14%) as a yellow solid.

^{1}H NMR (600 MHz, $CDCl_3$) δ 9.16–9.23 (m, 2H), 9.13 (s, 1H), 9.08 (d, J = 1.8 Hz, 1H), 8.95–9.00 (m, 2H),

8.88 (d, J = 9.0 Hz, 1H), 8.87 (d, J = 9.0 Hz, 1H), 8.46 (d, J = 9.0 Hz, 1H), 8.28 (d, J = 9.0 Hz, 1H), 8.20 (d, J = 7.8 Hz, 1H), 7.88 (dd, J = 8.7, 1.5 Hz, 1H), 7.81 (t, J = 7.2 Hz, 1H), 7.77 (t, J = 7.2 Hz, 1H), 7.67–7.74 (m, 2H), 7.58 (t, J = 7.2 Hz, 1H), 7.51 (td, J = 7.5, 1.2 Hz, 1H), 1.56 (s, 9H), 0.97 (s, 9H)

^{13}C NMR (600 MHz, $CDCl_3$) δ 149.47, 148.54, 132.86, 131.54, 130.94, 129.89, 129.73, 129.68, 129.17, 128.92, 128.68 (2C), 128.43, 128.02, 127.94 (2C), 127.65, 127.31, 127.07, 126.56, 126.51, 126.05 (2C), 125.76, 125.72, 125.57 (2C), 125.24, 124.89 (2C), 124.28, 124.22, 123.66, 123.36, 123.20, 122.77, 120.58, 120.55, 39.42, 35.15, 32.46, 31.51

HRMS (APCI, positive): Calcd for $C_{46}H_{37}$ ($[M+H]^+$): 589.2890. Found: 589.2892.

Naphtho[1,2,3,4-*ghi*]perylene (**18**)

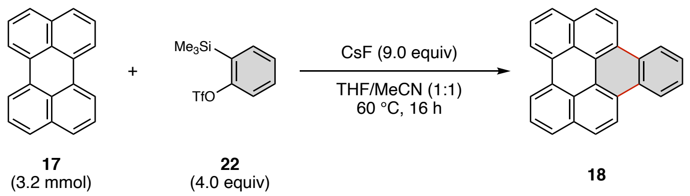

To a 300-mL two-necked round-bottomed flask containing a magnetic stirring bar was added perylene (**17**) (800 mg, 3.2 mmol, 1.0 equiv). The flask was taken into a glove box, and then CsF (4.32 g, 28.8 mmol, 9.0 equiv) was added. The flask was equipped with a reflux condenser, sealed with septa, and taken out from the glove box. Then, acetonitrile (80 mL), 2-(trimethylsilyl)phenyl trifluoromethanesulfonate (**22**) (2.3 mL, 9.6 mmol, 3.0 equiv) and THF (80 mL) were added to the flask. After stirring at 60 °C for 16 h, the reaction mixture was cooled to ambient temperature, and then diluted with $CHCl_3$ (100 mL). The mixture was filtered, and the filtrate was concentrated under reduced pressure. The residue was roughly purified by flash column chromatography on silica gel (eluent: hexane/$CHCl_3$ = 5:1). Then, the product was washed with methanol and further purified by GPC to yield desired product **18** (152 mg, 15%) as a yellow solid.

^{1}H NMR (600 MHz, $CDCl_3$) δ 9.10 (dd, J = 6.6, 3.0 Hz, 2H), 8.98 (d, J = 9.0 Hz, 2H), 8.88 (d, J = 7.2 Hz, 2H), 8.21 (d, J = 9.0 Hz, 2H), 8.15 (d, J = 7.8 Hz, 2H), 7.90–7.96 (m, 4H)

^{13}C NMR (150 MHz, $CDCl_3$) δ 132.59, 130.88, 128.55, 127.82, 127.01, 126.65, 126.61, 126.44, 126.35, 123.60, 122.13, 120.71 (one sp^2 signal was overlapping with others)

HRMS (APCI, positive): Calcd for $C_{26}H_{15}$ ($[M+H]^+$): 327.1168. Found: 327.1168.

3,12,17,24-Tetra-*tert*-butylhexabenzo[*a*,*c*,*fg*,*ij*,*m*,*o*]naphtho[1,2,3,4-*rst*]pentaphene (**19**)

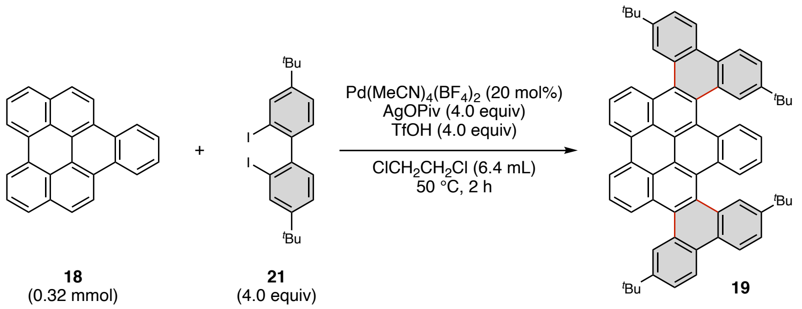

To a screw-capped tube containing a magnetic stirring bar were added **18** (105 mg, 0.32 mmol, 1.0 equiv), diiodobiphenyl **21** (668 mg, 1.28 mmol, 4 equiv) and AgOPiv (269 mg, 1,28 mmol, 5.0 equiv). The tube was taken into a glove box. Then, $Pd(MeCN)_4(BF_4)_2$ (27 mg, 60 µmol, 20 mol%) was added and the tube was sealed with a cap. The tube was taken out from the glove box, and then 1,2-dichloroethane (6.4 mL) and TfOH (115 µL, 1.28 mmol, 4.0 equiv) were added by a syringe. After stirring at 50 °C for 2 h, the reaction mixture was cooled to ambient temperature, and then passed through a short pad of silica gel (eluent: $CHCl_3$). After the organic solvent was removed under reduced pressure, the residue was purified by GPC to yield nanographene **19** (64.5 mg, 24%) as a yellow solid.

^{1}H NMR (600 MHz, $CDCl_3$) δ 9.07 (d, J = 7.8 Hz, 2H), 8.94–8.97 (m, 4H), 8.69 (d, J = 8.4 Hz, 2H), 8.57–8.60 (m, 4H), 8.48 (dd, J = 6.0, 3.6 Hz, 2H), 7.99 (t, J = 7.5 Hz, 2H), 7.83 (dd, J = 8.4, 1.8 Hz, 2H), 7.68 (dd, J = 8.1, 2.1 Hz, 2H), 7.13 (dd, J = 7.2, 3.6 Hz, 2H), 1.53 (s, 18H), 1.25 (s, 18H)

^{13}C NMR (150 MHz, $CDCl_3$) δ 148.91, 148.26, 131.10, 130.94, 130.67, 130.32, 130.01, 129.52, 129.11, 128.99, 127.74, 127.35, 127.20, 126.66, 126.30, 125.66, 125.60, 124.76, 124.43, 124.36, 124.25, 123.54, 123.21, 122.90, 120.87, 35.13, 34.75, 31.60, 31.54

HRMS (APCI, positive): Calcd for $C_{66}H_{59}$ ($[M+H]^+$): 851.4611. Found: 851.4602.

1.13 X-ray crystallographic analysis

Details of the crystal data and a summary of the intensity data collection parameters for **4aa**, **4pa**, **6ba-1**, **6ha**, **11**, **13**, **15** and **16** are listed in Table S1–S3. A suitable crystal was mounted with mineral oil on a MiTeGen MicroMounts and transferred to the goniometer of the kappa goniometer of a RIGAKU XtaLAB Synergy-S system with 1.2 kW MicroMax-007HF microfocus rotating anode (Graphite-monochromated Mo Kα radiation (λ = 0.71073 Å)) and PILATUS200K hybrid photon-counting detector. Cell parameters were determined and refined, and raw frame data were integrated using CrysAlis^{Pro} (Agilent Technologies, 2010). The structures were solved by direct methods with SHELXT[16] and refined by full-matrix least-squares techniques against F^2 (SHELXL-2018/3)[17] by using Olex2 software package[18]. The intensities were corrected for Lorentz and polarization effects. The non-hydrogen atoms were refined anisotropically. Hydrogen atoms were placed using AFIX instructions. CCDC 2012769–2012775 and 2071523 contain the supplementary crystallographic data for this paper. These data can be obtained free of charge from The Cambridge Crystallographic Data Centre via www.ccdc.cam.ac.uk/data_request/cif.

Supplementary Table 1 . Crystallographic data and refinement details for **4aa**, **4pa** and **6ba-1**.

	**4aa**	**4pa**	**6ba-1**
CCDC	2012772	2071523	2012774
formula	$C_{31.5}H_{23.5}Cl_{1.5}N_3O_2$	$C_{37}H_{25}N_3O_2$	$C_{57}H_{49}Cl_3N_6O_4$
fw	529.21	543.60	988.37
T (K)	123(2)	123(2)	123(2)
λ (Å)	0.71073	0.71073	0.71073
cryst syst	Triclinic	Monoclinic	Triclinic
space group	P-1	C2/c	P-1
a (Å)	9.3418(4)	18.9129(14)	12.8984(7)
b (Å)	11.4529(6)	13.0709(6)	13.6946(6)
c (Å)	13.1938(7)	24.166(2)	15.6121(9)
α (deg)	97.906(4)	90	75.854(4)
β (deg)	100.525(4)	118.893(11)	81.151(5)
γ (deg)	113.299(5)	90	71.487(5)
V (Å^3)	1239.81(12)	5230.5(8)	2527.0(2)
Z	2	8	2
D_{calc} (g / cm^3)	1.418	1.381	1.299
μ (mm^{-1})	0.245	0.086	0.235
F(000)	550.0	2272.0	1032.0
cryst size (mm)	0.150 × 0.150 × 0.150	0.150 × 0.050 × 0.050	0.100 × 0.100 × 0.050
2θ range, (deg)	3.98 to 55.466	3.85 to 55.966	3.736 to 56.18
reflns collected	14563	18194	30393
indep reflns/R_{int}	5017/0.0789	5262/ 0.1026	10219/0.1034
params	362	380	637
GOF on F^2	1.064	1.021	1.023
R_1, wR_2 [I>2σ(I)]	0.0638, 0.1592	0.0709, 0.1557	0.0897, 0.2068
R_1, wR_2 (all data)	0.1045, 0.1810	0.1364, 0.1819	0.2295, 0.2620

Supplementary Table 2 . Crystallographic data and refinement details for **11**, **6ha**, and **13**.

	**11**	**6ha**	**13**
CCDC	2012771	2012775	2012769
formula	$C_{50}H_{40}$	$C_{45}H_{34}Cl_2N_6O_4$	$C_{58}H_{44}$
fw	640.82	793.68	740.93
T (K)	123(2)	123(2)	123(2)
λ (Å)	0.71073	0.71073	0.71073
cryst syst	Monoclinic	Triclinic	Monoclinic
space group	$P2_1$/c	P-1	$P2_1$/n
a (Å)	22.7513(18)	11.40820(10)	13.5366(6)
b (Å)	8.4732(6)	12.15800(10)	19.4330(7)
c (Å)	18.4080(15)	14.27090(10)	16.1458(7)
α (deg)	90	86.0930(10)	90
β (deg)	107.189(8)	88.0900(10)	110.398(5)
γ (deg)	90	67.2740(10)	90
V (Å^3)	3390.1(5)	1821.42(3)	3980.9(3)
Z	4	2	4
D_{calc} (g / cm^3)	1.256	1.447	1.236
μ (mm^{-1})	0.071	0.235	0.070
F(000)	1360.0	824.0	1568.0
cryst size (mm)	0.150 × 0.150 × 0.150	0.150 × 0.150 × 0.150	0.150 × 0.100 × 0.050
2θ range, (deg)	5.024 to 59.422	4.832 to 59.622	3.99–55.528
reflns collected	36254	35372	23719
indep reflns/R_{int}	8796/0.1036	9371/0.0235	7857/0.0371
params	457	516	529
GOF on F^2	1.100	1.037	1.039
R_1, wR_2 [I>2σ(I)]	0.1101, 0.2921	0.0460, 0.1321	0.0483, 0.1097
R_1, wR_2 (all data)	0.2135, 0.3304	0.0526, 0.1368	0.0871, 0.1237

Supplementary Table 3 . Crystallographic data and refinement details for **15** and **16**.

	**15**	**16**
CCDC	2012773	2012770
formula	$C_{143.25}H_{127.25}Cl_{9.75}$	$C_{92}H_{72}$
fw	2194.33	1177.49
T (K)	123(2)	123(2)
λ (Å)	0.71073	0.71073
cryst syst	Triclinic	Triclinic
space group	P-1	P-1
a (Å)	16.9476(4)	12.1293(3)
b (Å)	18.9439(5)	15.3894(4)
c (Å)	21.4965(4)	17.4280(4)
α (deg)	70.274(2)	83.921(2)
β (deg)	86.020(2)	81.852(2)
γ (deg)	66.288(2)	71.337(2)
V (Å^3)	5930.4(3)	3044.62(14)
Z	2	2
D_{calc} (g / cm^3)	1.229	1.284
μ (mm^{-1})	0.281	0.072
F(000)	2305.0	1248.0
cryst size (mm)	0.150 × 0.150 × 0.150	0.150 × 0.100 × 0.050
2θ range, (deg)	3.438 to 55.746	3.546 to 55.448
reflns collected	78558	38586
indep reflns/R_{int}	23900/0.0692	12162/0.0599
params	1524	841
GOF on F^2	1.034	0.903
R_1, wR_2 [I>2σ(I)]	0.0851, 0.2123	0.0510, 0.1316
R_1, wR_2 (all data)	0.1543, 0.2435	0.0957, 0.1535

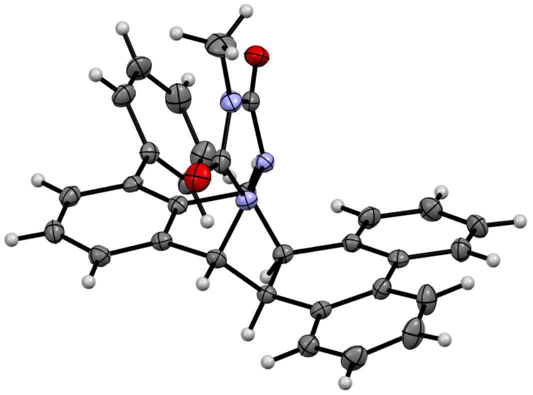

Supplementary Fig. 2. ORTEP drawing of **4aa** with 50% probability. The solvent molecule ($CHCl_3$) is omitted for clarity.

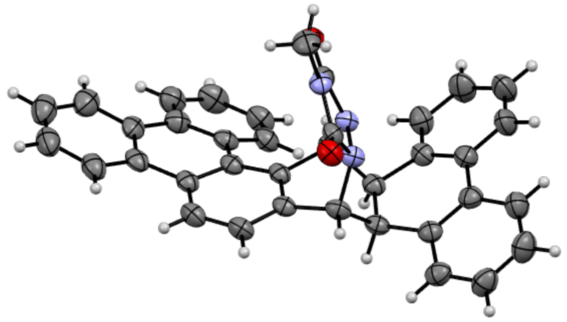

Supplementary Fig. 3. ORTEP drawing of **4pa** with 50% probability.

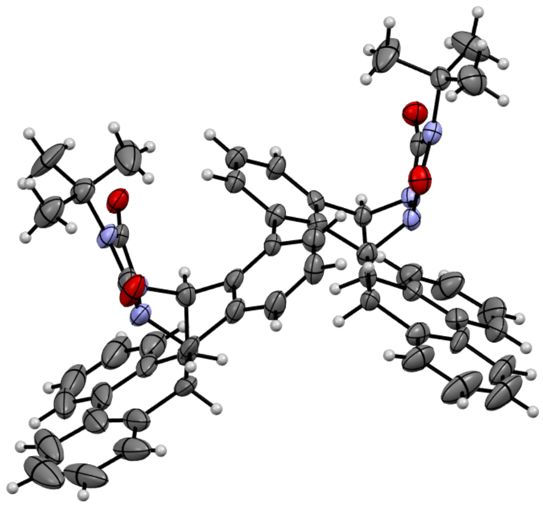

Supplementary Fig. 4. ORTEP drawing of **6ba-1** with 50% probability. The solvent molecule ($CHCl_3$) is omitted for clarity.

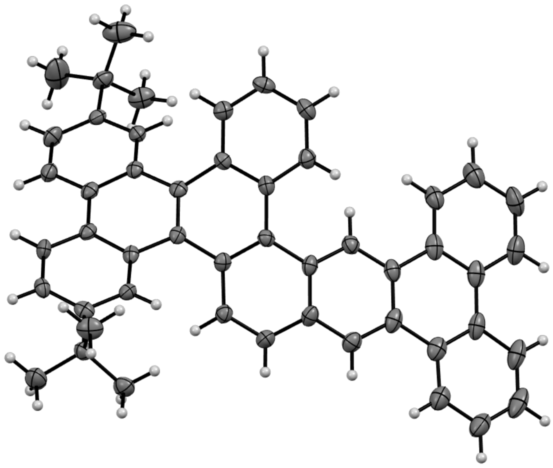

Supplementary Fig. 5. ORTEP drawing of **11** with 50% probability.

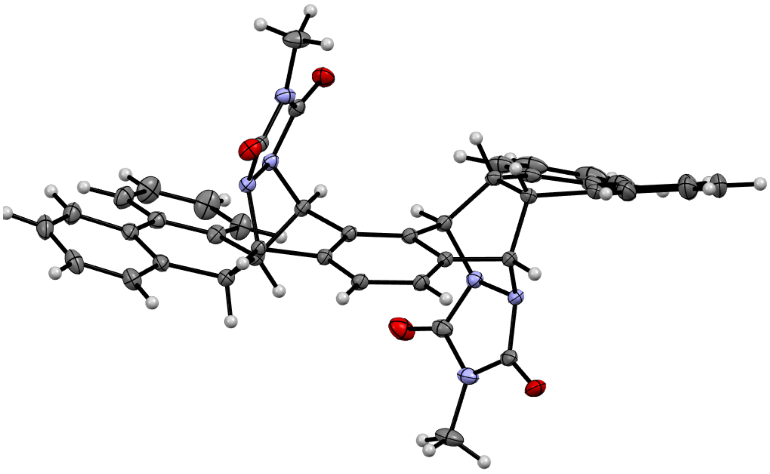

Supplementary Fig. 6. ORTEP drawing of **6ha** with 50% probability. The solvent molecule (CH_2Cl_2) is omitted for clarity.

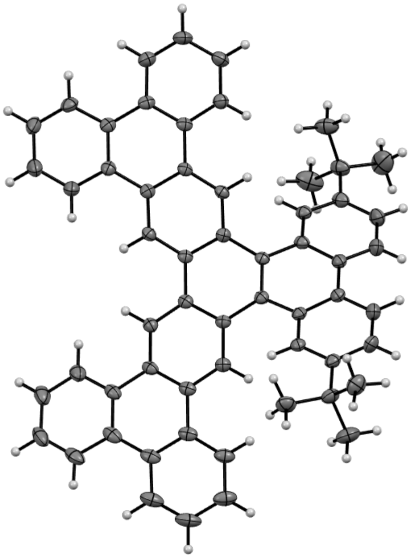

Supplementary Fig. 7. ORTEP drawing of **13** with 50% probability.

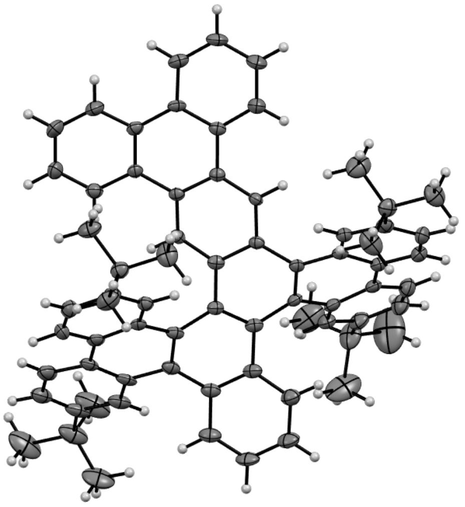

Supplementary Fig. 8. ORTEP drawing of **15** with 50% probability. One of two independent molecules and solvent molecules ($CHCl_3$) are omitted for clarity.

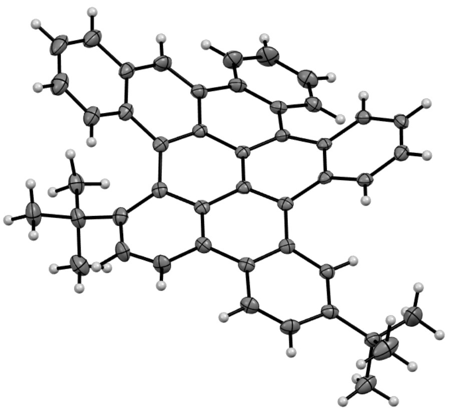

Supplementary Fig. 9. ORTEP drawing of **16** with 50% probability. One of two independent molecules is omitted for clarity.

1.14 Computational study

The Gaussian 16 program[19] running on the NEC LX system was used for optimization, calculations of molecular orbitals and their energies. Structures were optimized without any symmetry assumptions with M06-2X[20] functional and the 6-31+G(d) basis set[21–23]. Zero-point energy, enthalpy, and Gibbs free energy at 298.15 K and 1 atm were estimated from the gas-phase studies. The nature of the stationary points was determined in each case according to the appropriate number of negative eigenvalues of the Hessian matrix. Intrinsic reaction coordinate (IRC)[24] calculations were also carried out for each transition state to ensure the connection of appropriate reactants and products. Single-point energies for all optimized structures were determined at M06-2X/6-311++G(d,p) level. Computed structures are visualized using CYL-view.[25]

Structure sampling

In evaluating the stability of cycloadducts, structure sampling for each cycloadduct was conducted as shown in Supplementary Fig. 10. The cycloadduct **2p** potentially has four conformational isomers, namely (*exo*,*P*)-**2p**, (*exo*,*M*)-**2p**, (*endo*,*P*)-**2p** and (*endo*,*M*)-**2p**, depending on conformations at the bicyclic moiety (*i.e. exo*/*endo*) and [4]helicene-like moiety (*i.e. P*/*M*). To determine the most stable conformations, optimizations and calculations of energies for these conformers were individually conducted. As a result, (*exo*/*M*)-**2p** was found to be the most stable, and relative free energies of the other conformers are shown in the left column of Supplementary Fig. 10. Similarly, transition states (TSs) for retro-Diels–Alder (rDA) reaction were investigated starting from each conformer, and corresponding geometries and energies were obtained ((*exo*,*P*)-$\mathbf{TS_1}$, (*exo*,*M*)-$\mathbf{TS_1}$, (*endo*,*P*)-$\mathbf{TS_1}$ and (*endo*,*M*)-$\mathbf{TS_1}$). As a result, the energy of (*exo*,*M*)-$\mathbf{TS_1}$ was found to be the lowest, and the activation barrier was calculated to be 28.9 kcal/mol. The conformational isomers of **2p′** and **2p″** as well as corresponding TSs for rDA reaction were also evaluated in a same manner, and activation energies were estimated to be 26.2 kcal/mol for **2p′** and 26.3 kcal/mol for **2p″**. Notably, isomerization between conformational isomers seems to be very fast, as exemplified by isomerizations between (*exo*/*M*)-**2p**, (*exo*/*P*)-**2p** and (*endo*/*P*)-**2p** through $\mathbf{TS_{M\text{-}P}}$ and $\mathbf{TS_{exo\text{-}endo}}$ (shown in the bottom of Supplementary Fig. 10).

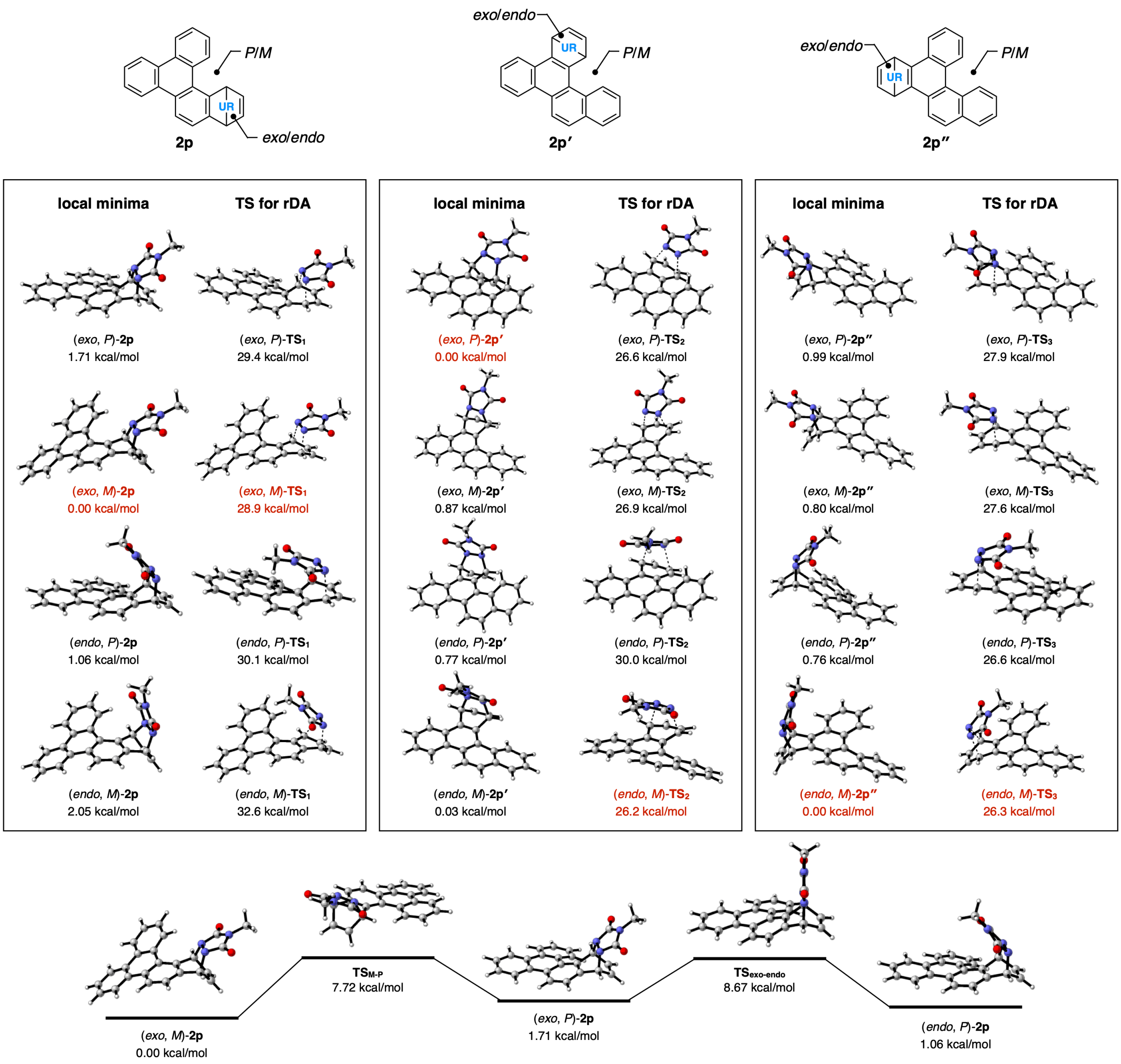

Supplementary Fig. 10. Structure sampling of conformers of **2p**, **2p′** and **2p″** and transition states for retro-Diels–Alder reactions from each cycloadduct (M06-2X/6-311++G(d,p)//M06-2X/6-31+G(d)).

Supplementary Table 4. Uncorrected and thermal-corrected (298 K) energies of stationary points (Hartree) at M06-2X/6-31+G(d).[a]

structure	M06-2X/6-31+G(d)						
	EE	*ZPE*	*ΔH*	*ΔG*	*H*	*G*	IF
**MTAD**	-430.635122	0.073457	0.081388	0.041540	-430.553734	-430.593582	–
**1p**	-846.482720	0.291477	0.307133	0.249931	-846.175587	-846.232789	–
(*exo*,*P*)-**2p**	-1277.147161	0.370450	0.392873	0.319860	-1276.754288	-1276.827302	–
(*exo*,*P*)-**TS$_1$**	-1277.096682	0.366329	0.389333	0.313750	-1276.707349	-1276.782932	-456.2183
(*exo*,*M*)-**2p**	-1277.148462	0.370368	0.392887	0.318553	-1276.755575	-1276.829909	–
(*exo*,*M*)-**TS$_1$**	-1277.097146	0.366309	0.389339	0.313631	-1276.707808	-1276.783515	-457.3250
(*endo*,*P*)-**2p**	-1277.147577	0.370576	0.392977	0.319706	-1276.754600	-1276.827871	–
(*endo*,*P*)-**TS$_1$**	-1277.098113	0.366894	0.389402	0.317187	-1276.708712	-1276.780926	-485.5244
(*endo*,*M*)-**2p**	-1277.146781	0.370776	0.393136	0.320241	-1276.753645	-1276.826541	–
(*endo*,*M*)-**TS$_1$**	-1277.091223	0.366281	0.389350	0.313860	-1276.701873	-1276.777363	-443.7049
(*exo*,*P*)-**2p′**	-1277.143746	0.370455	0.392889	0.320176	-1276.750857	-1276.823570	–
(*exo*,*P*)-**TS$_2$**	-1277.094002	0.366396	0.389452	0.313070	-1276.704550	-1276.780932	-475.3041
(*exo*,*M*)-**2p′**	-1277.142617	0.370490	0.392890	0.320342	-1276.749727	-1276.822274	–
(*exo*,*M*)-**TS$_2$**	-1277.095133	0.366523	0.389449	0.314662	-1276.705684	-1276.780472	-465.1542
(*endo*,*P*)-**2p′**	-1277.143083	0.370818	0.393115	0.320962	-1276.749968	-1276.822121	–
(*endo*,*P*)-**TS$_2$**	-1277.090456	0.366536	0.389458	0.315526	-1276.700998	-1276.774930	-413.7189
(*endo*,*M*)-**2p′**	-1277.143245	0.370512	0.392894	0.320187	-1276.750352	-1276.823058	–
(*endo*,*M*)-**TS$_2$**	-1277.098236	0.366968	0.389445	0.317535	-1276.708791	-1276.780702	-487.4500
(*exo*,*P*)-**2p″**	-1277.141301	0.370527	0.392895	0.320200	-1276.748406	-1276.821101	–
(*exo*,*P*)-**TS$_3$**	-1277.093108	0.366605	0.389451	0.315045	-1276.703657	-1276.778063	-473.6024
(*exo*,*M*)-**2p″**	-1277.141586	0.370485	0.392855	0.320226	-1276.748731	-1276.821360	–
(*exo*,*M*)-**TS$_3$**	-1277.093348	0.366620	0.389510	0.314878	-1276.703838	-1276.778470	-474.1644
(*endo*,*P*)-**2p″**	-1277.141580	0.370712	0.392997	0.320516	-1276.748583	-1276.821063	–
(*endo*,*P*)-**TS$_3$**	-1277.095853	0.366740	0.389301	0.316839	-1276.706552	-1276.779014	-497.7352
(*endo*,*M*)-**2p″**	-1277.142719	0.370641	0.392926	0.320544	-1276.749794	-1276.822175	–
(*endo*,*M*)-**TS$_3$**	-1277.095684	0.366659	0.389336	0.316173	-1276.706348	-1276.779510	-491.6736
**TS$_{M-P}$**	-1277.138595	0.370357	0.392176	0.320549	-1276.746419	-1276.818046	-105.3178
**TS$_{exo-endo}$**	-1277.136127	0.369843	0.391821	0.319986	-1276.744306	-1276.816141	-89.5739

(a) EE: electronic energy; *ZPE*: zero-point energy correction; *ΔH*: thermal correction to enthalpy; *ΔG*: thermal correction to free energy; H (= $EE + \Delta H = EE + ZPE + E_{vib} + E_{rot} + E_{trans} + RT$): sum of electronic energy and thermal correction to enthalpy; G (= $EE + \Delta G = H - TS$): sum of electronic energy and thermal correction to free energy.

Supplementary Table 5. Uncorrected and thermal-corrected (298 K) energies of stationary points (Hartree) at M06-2X/6-311++G(d,p)//M06-2X/6-31+G(d).[a]

structure	6-31+G(d)			6-311++G(d,p)	*H*	*G*
	ZPE	*ΔH*	*ΔG*	*EE*		
**MTAD**	0.073457	0.081388	0.041540	-430.7478657	-430.666478	-430.706326
**1p**	0.291477	0.307133	0.249931	-846.6633168	-846.356184	-846.413386
(*exo*,*P*)-**2p**	0.370450	0.392873	0.319860	-1277.440602	-1277.047729	-1277.120742
(*exo*,*P*)-$\mathbf{TS_1}$	0.366329	0.389333	0.313750	-1277.390446	-1277.001113	-1277.076696
(*exo*,*M*)-**2p**	0.370368	0.392887	0.318553	-1277.442024	-1277.049137	-1277.123471
(*exo*,*M*)-$\mathbf{TS_1}$	0.366309	0.389339	0.313631	-1277.391068	-1277.001729	-1277.077437
(*endo*,*P*)-**2p**	0.370576	0.392977	0.319706	-1277.441481	-1277.048504	-1277.121775
(*endo*,*P*)-$\mathbf{TS_1}$	0.366894	0.389402	0.317187	-1277.392750	-1277.003348	-1277.075563
(*endo*,*M*)-**2p**	0.370776	0.393136	0.320241	-1277.440444	-1277.047308	-1277.120203
(*endo*,*M*)-$\mathbf{TS_1}$	0.366281	0.389350	0.313860	-1277.385348	-1276.995998	-1277.071488
(*exo*,*P*)-**2p′**	0.370455	0.392889	0.320176	-1277.437421	-1277.044532	-1277.117245
(*exo*,*P*)-$\mathbf{TS_2}$	0.366396	0.389452	0.313070	-1277.387946	-1276.998494	-1277.074876
(*exo*,*M*)-**2p′**	0.370490	0.392890	0.320342	-1277.436195	-1277.043305	-1277.115853
(*exo*,*M*)-$\mathbf{TS_2}$	0.366523	0.389449	0.314662	-1277.389016	-1276.999567	-1277.074354
(*endo*,*P*)-**2p′**	0.370818	0.393115	0.320962	-1277.436983	-1277.043868	-1277.116021
(*endo*,*P*)-$\mathbf{TS_2}$	0.366536	0.389458	0.315526	-1277.384752	-1276.995294	-1277.069226
(*endo*,*M*)-**2p′**	0.370512	0.392894	0.320187	-1277.437391	-1277.044497	-1277.117204
(*endo*,*M*)-$\mathbf{TS_2}$	0.366968	0.389445	0.317535	-1277.393079	-1277.003634	-1277.075544
(*exo*,*P*)-**2p″**	0.370527	0.392895	0.320200	-1277.435016	-1277.042121	-1277.114816
(*exo*,*P*)-$\mathbf{TS_3}$	0.366605	0.389451	0.315045	-1277.387030	-1276.997579	-1277.071985
(*exo*,*M*)-**2p″**	0.370485	0.392855	0.320226	-1277.435344	-1277.042489	-1277.115118
(*exo*,*M*)-$\mathbf{TS_3}$	0.366620	0.389510	0.314878	-1277.387240	-1276.997730	-1277.072362
(*endo*,*P*)-**2p″**	0.370712	0.392997	0.320516	-1277.435706	-1277.042709	-1277.115190
(*endo*,*P*)-$\mathbf{TS_3}$	0.366740	0.389301	0.316839	-1277.390867	-1277.001566	-1277.074028
(*endo*,*M*)-**2p″**	0.370641	0.392926	0.320544	-1277.436941	-1277.044015	-1277.116397
(*endo*,*M*)-$\mathbf{TS_3}$	0.366659	0.389336	0.316173	-1277.390603	-1277.001267	-1277.074430
$\mathbf{TS_{M\text{-}P}}$	0.370357	0.392176	0.320549	-1277.431722	-1277.039546	-1277.111173
$\mathbf{TS_{exo\text{-}endo}}$	0.369843	0.391821	0.319986	-1277.429633	-1277.037812	-1277.109647

(a) *EE*: electronic energy; *ZPE*: zero-point energy; *ΔH*: thermal correction to enthalpy; *ΔG*: thermal correction to free energy; H (= $EE + \Delta H = EE + ZPE + E_{vib} + E_{rot} + E_{trans} + RT$): sum of electronic energy and thermal correction to enthalpy; G (= $EE + \Delta G = H - TS$): sum of electronic energy and thermal correction to free energy.

1.15 Photophysical properties

UV/Vis absorption spectra of **10**, **11**, **12** and **13** in CH_2Cl_2 were recorded on a Shimadzu UV-3600 spectrometer with a resolution of 0.5 nm. Emission spectra in CH_2Cl_2 was measured with a Shimadzu RF-6000 Hitachi spectrometer with a resolution of 0.2 nm upon excitation at 370 nm for **10** and **11**, 350 nm for **12** and 400 nm for **13**. Dilute solutions in degassed spectral grade CH_2Cl_2 in a 1 cm square quartz cell were used for measurements.

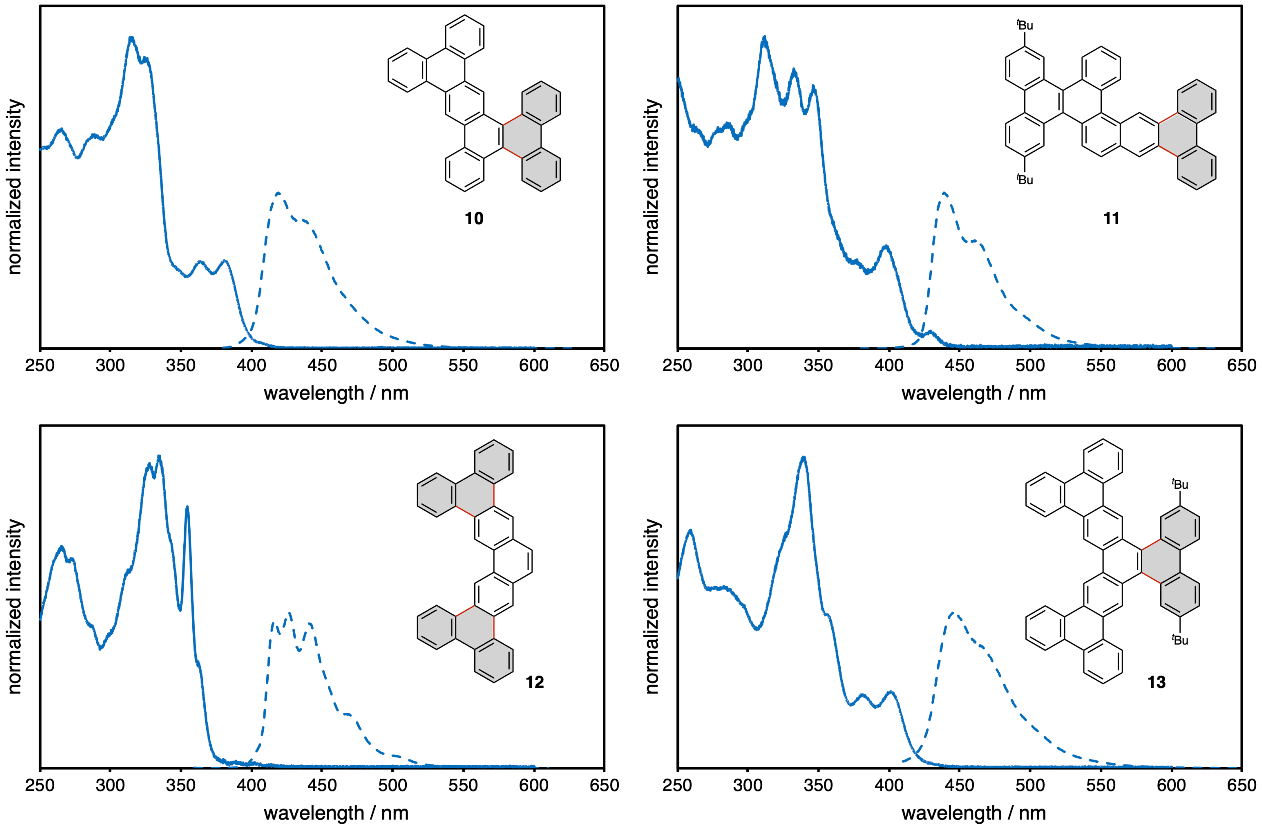

Supplementary Fig. 11. Photophysical properties of nanographenes **10**, **11**, **12** and **13**.

1.16 Assignment of ¹H NMR signals

The assignment of ¹H NMR signals of PAHs and nanographenes have been conducted as follows (Supplementary Fig. 12). As protons located next to a junction of two benzene rings (colored by blue) are deshielded by ring currents on both rings, their signals appear around 8 ppm, whereas ¹H NMR signals of typical aromatic compounds appear at 7-7.5 ppm (*e.g.* benzene: 7.3 ppm, *p*-xylene: 7.0 ppm). Similarly, signals corresponding to protons at *bay*-, *cove*- and *fjord*-regions (colored by green) are always observed at 8-9 ppm because these protons experience deshielding by three or more benzene rings. Moreover, protons located between two junctions of benzene rings (colored by red) are further deshielded, and they are observed as singlet signals at 9-11 ppm. Based on these knowledges, total number of protons and multiplicity of each signal, ¹H NMR signals of PAHs and nanographenes are assigned as exemplified by **5ka**.

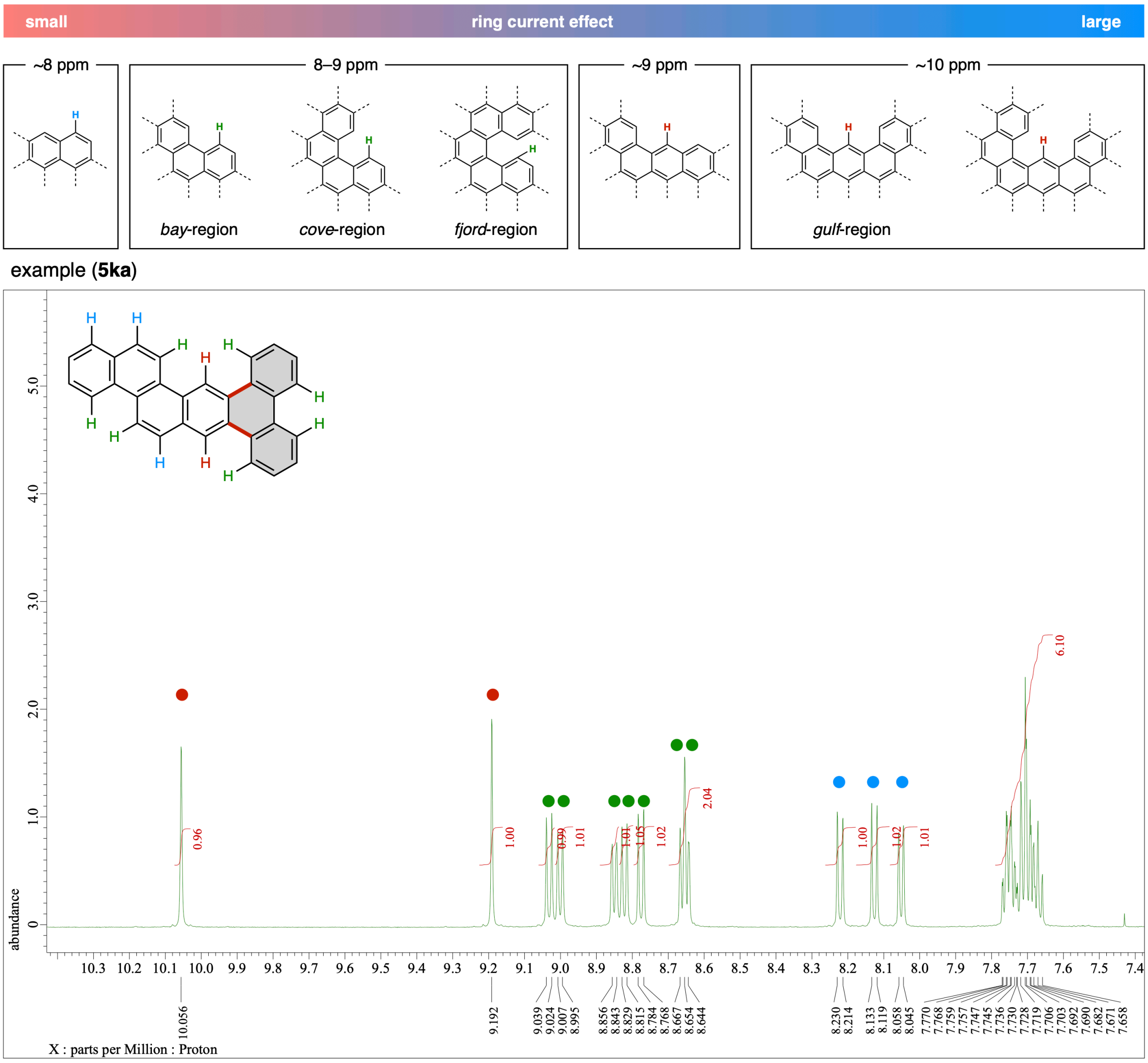

Supplementary Fig. 12. Assignment of ¹H NMR signals of PAHs and nanographenes.

1.17 ^{1}H and ^{13}C NMR spectra

^{1}H NMR spectrum of **1q** (solvent: $CDCl_3$)

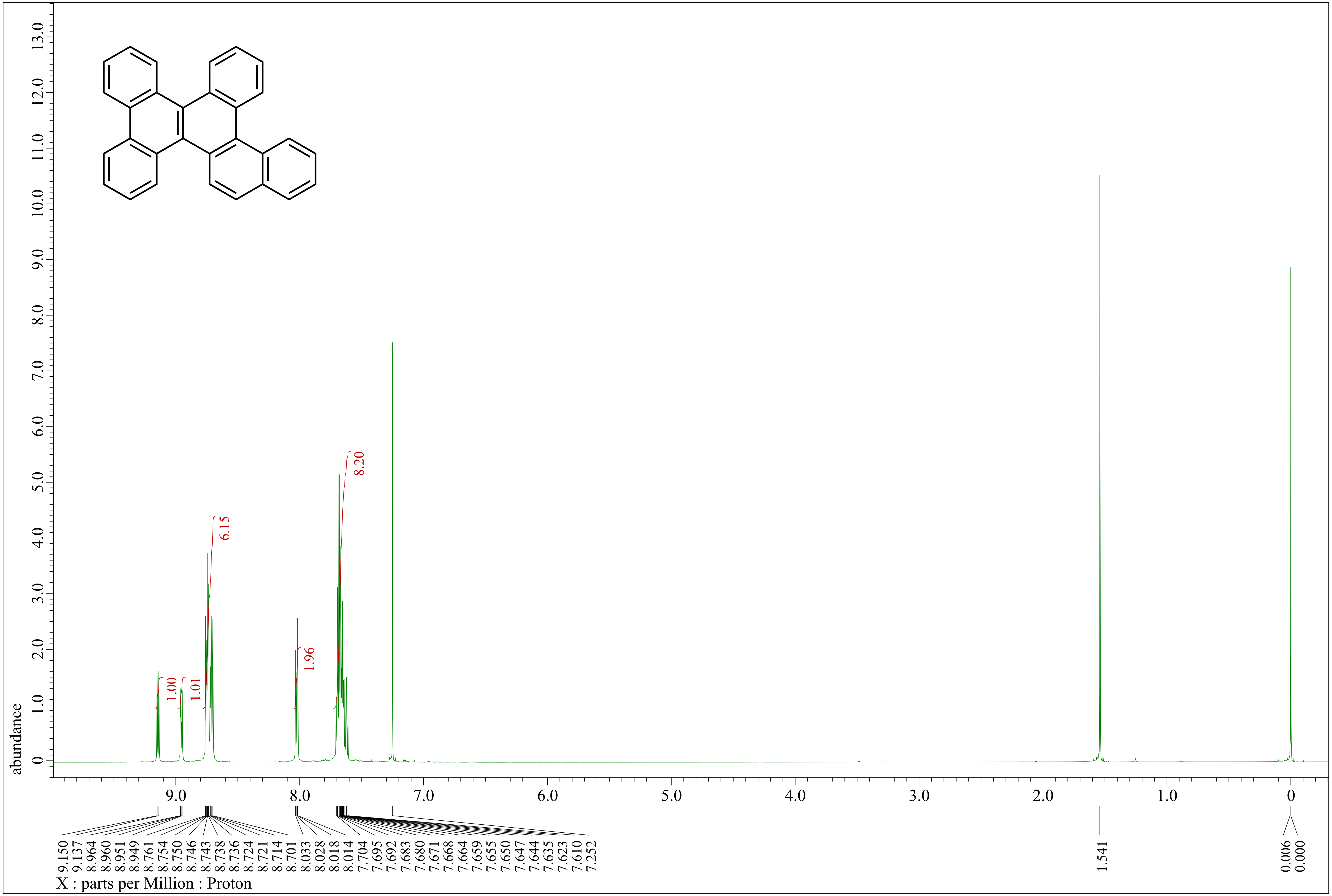

^{1}H NMR spectrum (inset) of **1q** (solvent: $CDCl_3$)

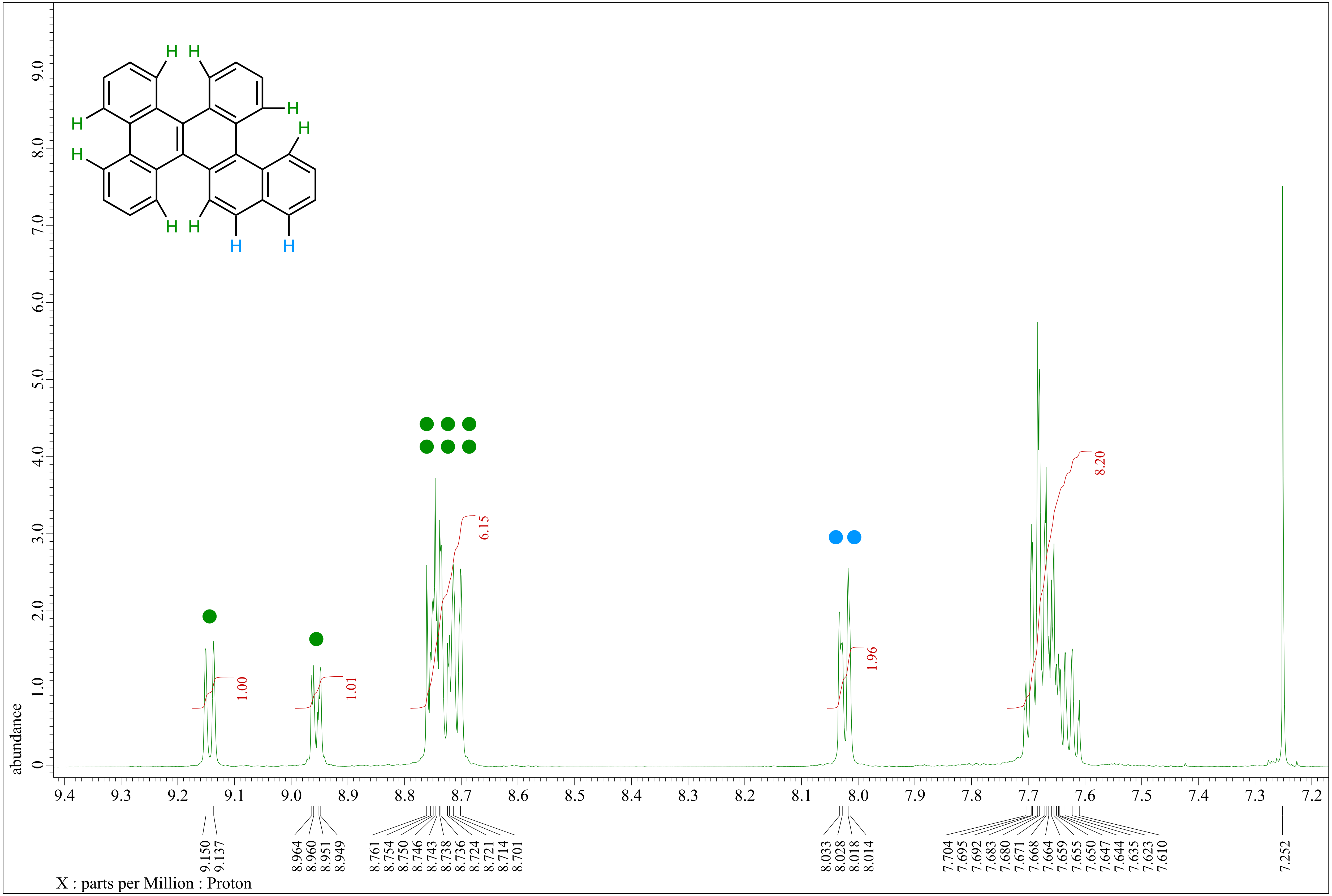

^{13}C NMR spectrum of **1q** (solvent: $CDCl_3$)

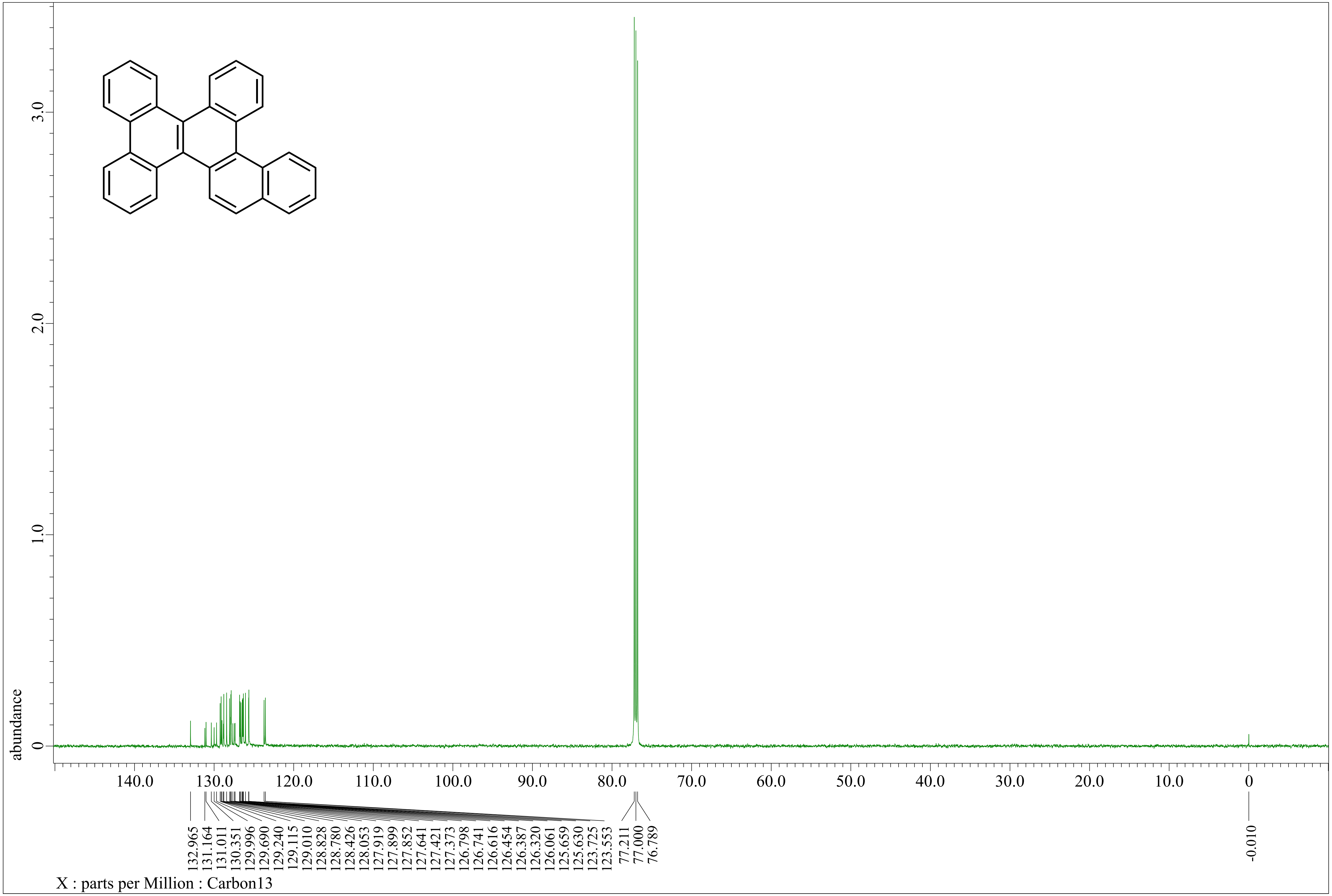

^{13}C NMR spectrum (inset) of **1q** (solvent: $CDCl_3$)

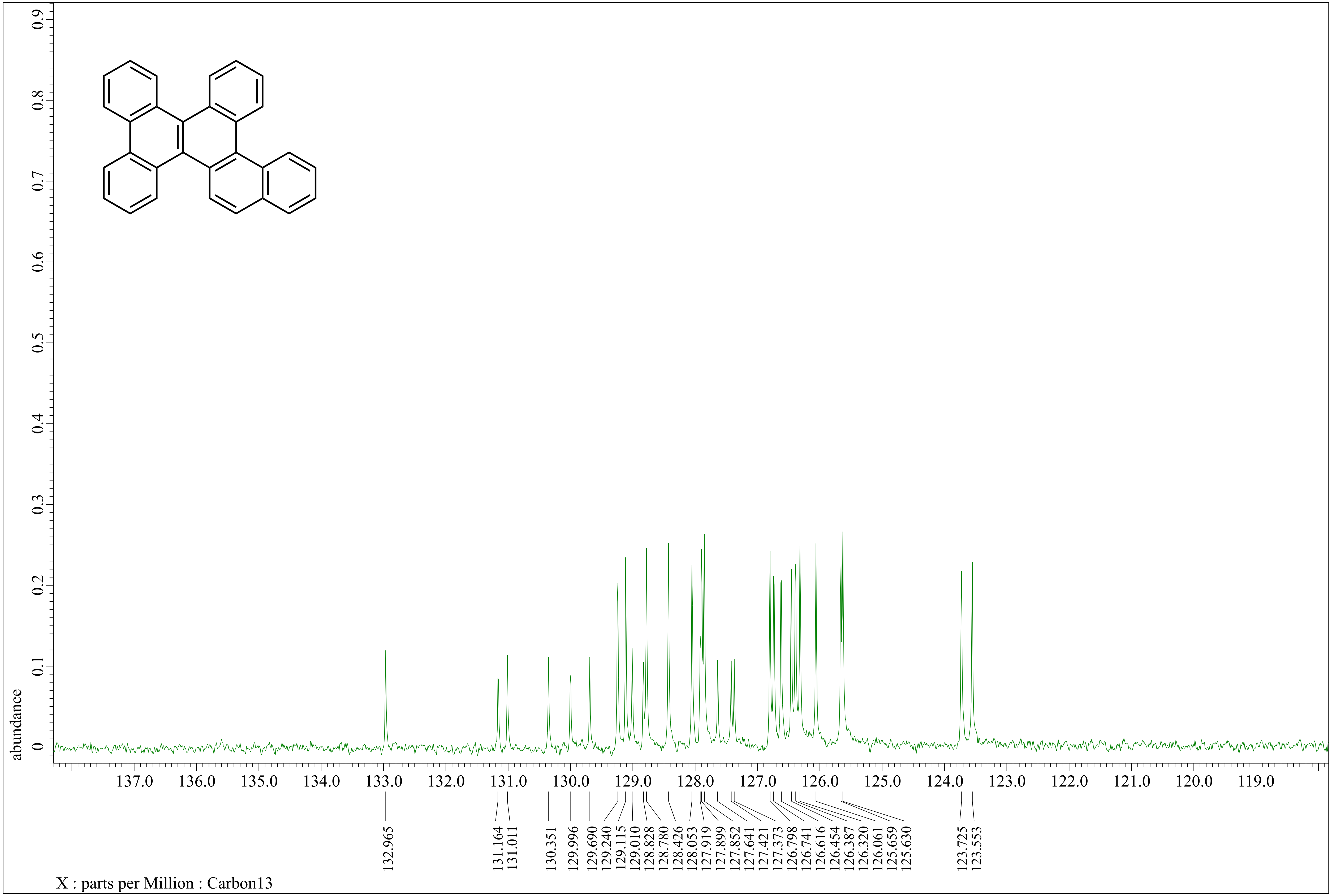

^{1}H NMR spectrum of **2a** (solvent: acetone-d_6)

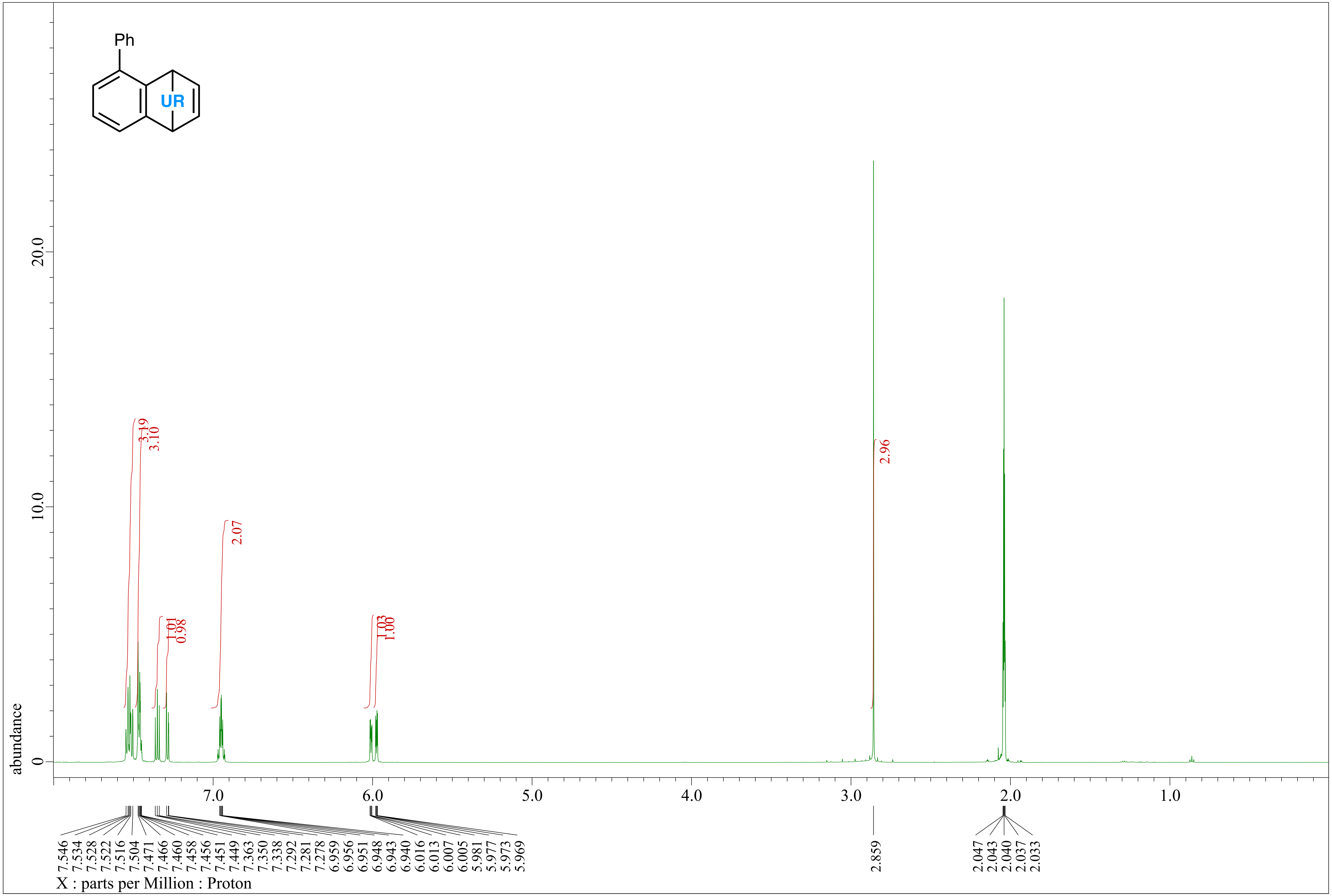

^{1}H NMR spectrum (inset) of **2a** (solvent: acetone-d_6)

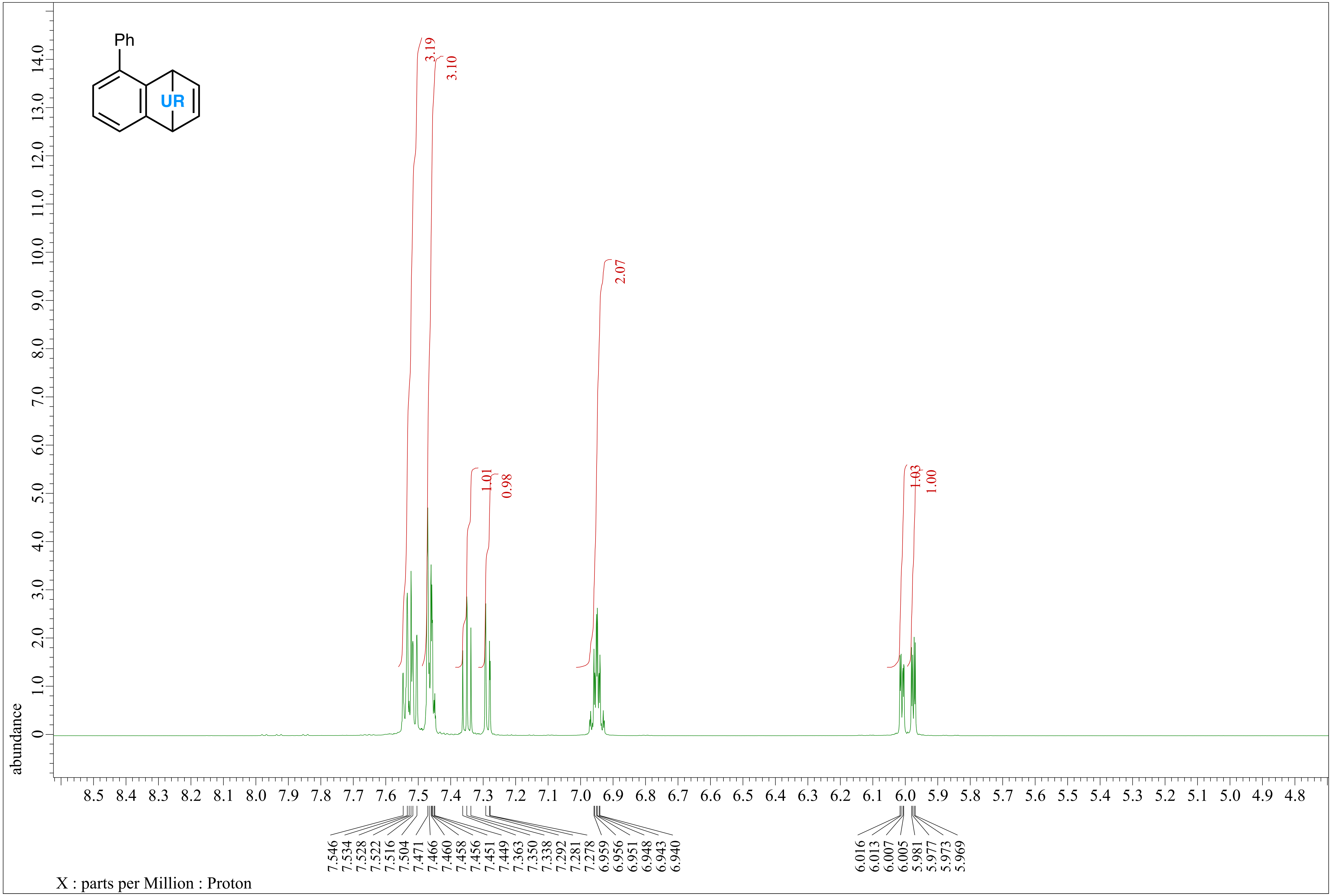

^{13}C NMR spectrum of **2a** (solvent: acetone-d_6)

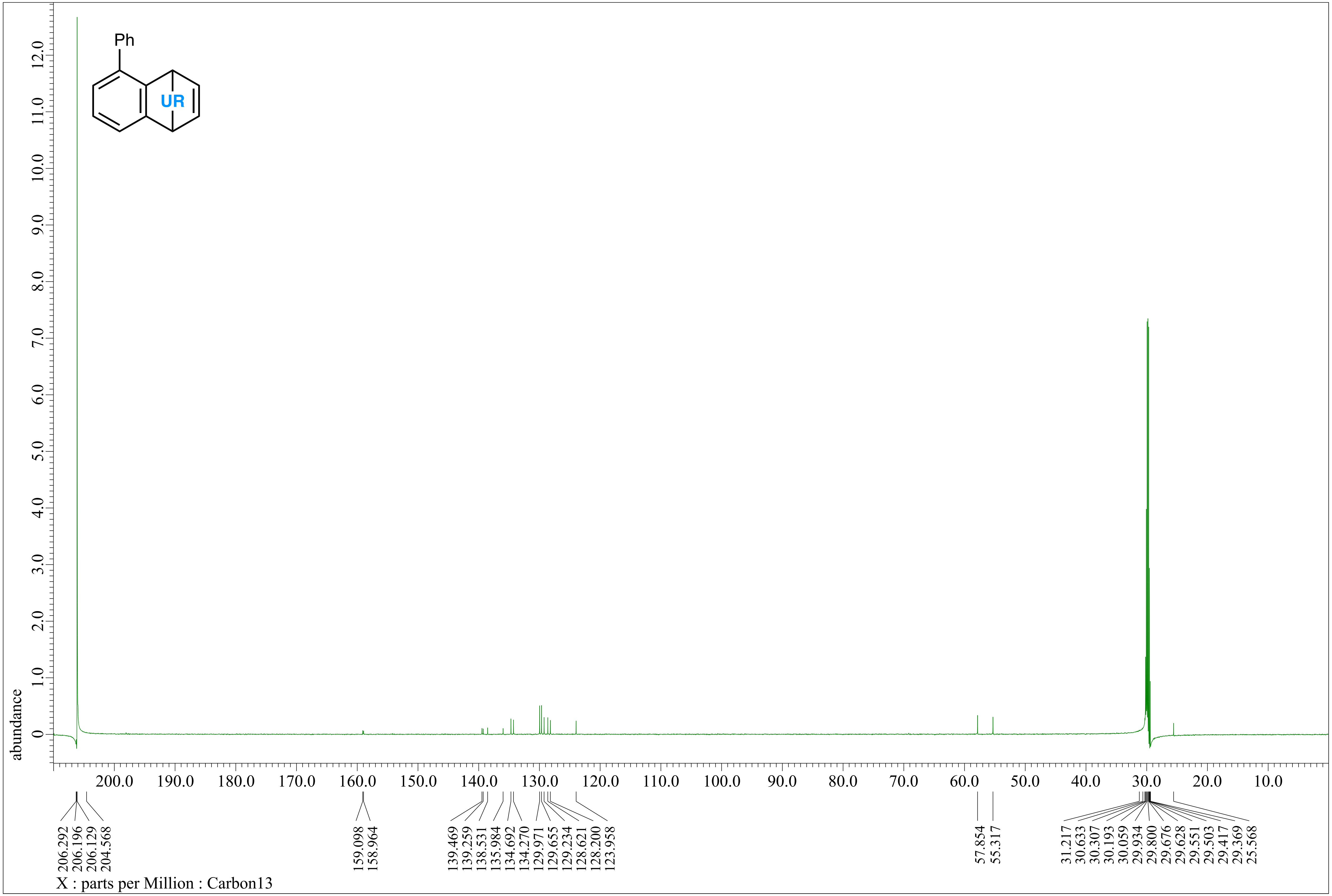

^{13}C NMR spectrum (inset) of **2a** (solvent: acetone-d_6)

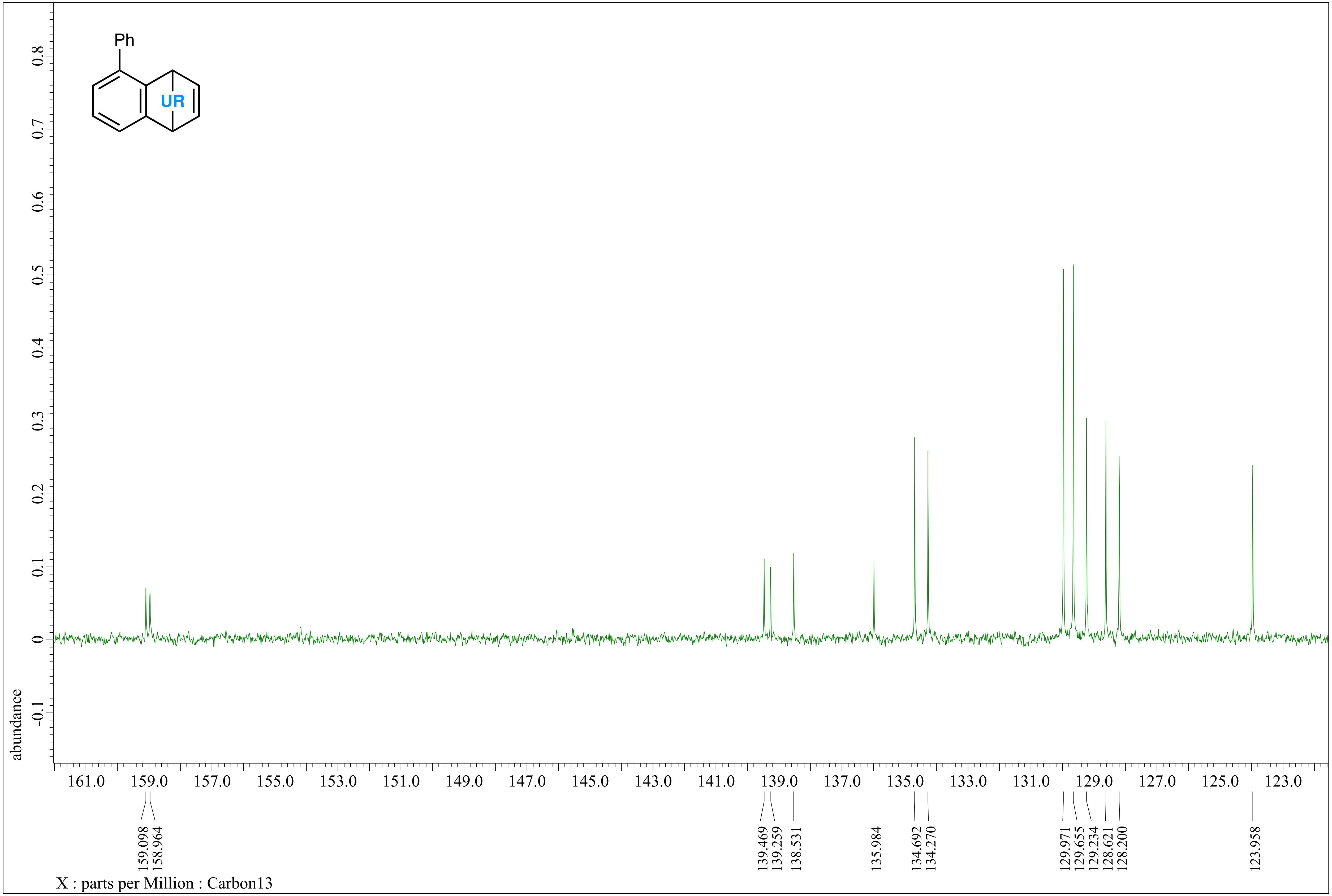

^{1}H NMR spectrum of **2g** (solvent: acetone-d_6)

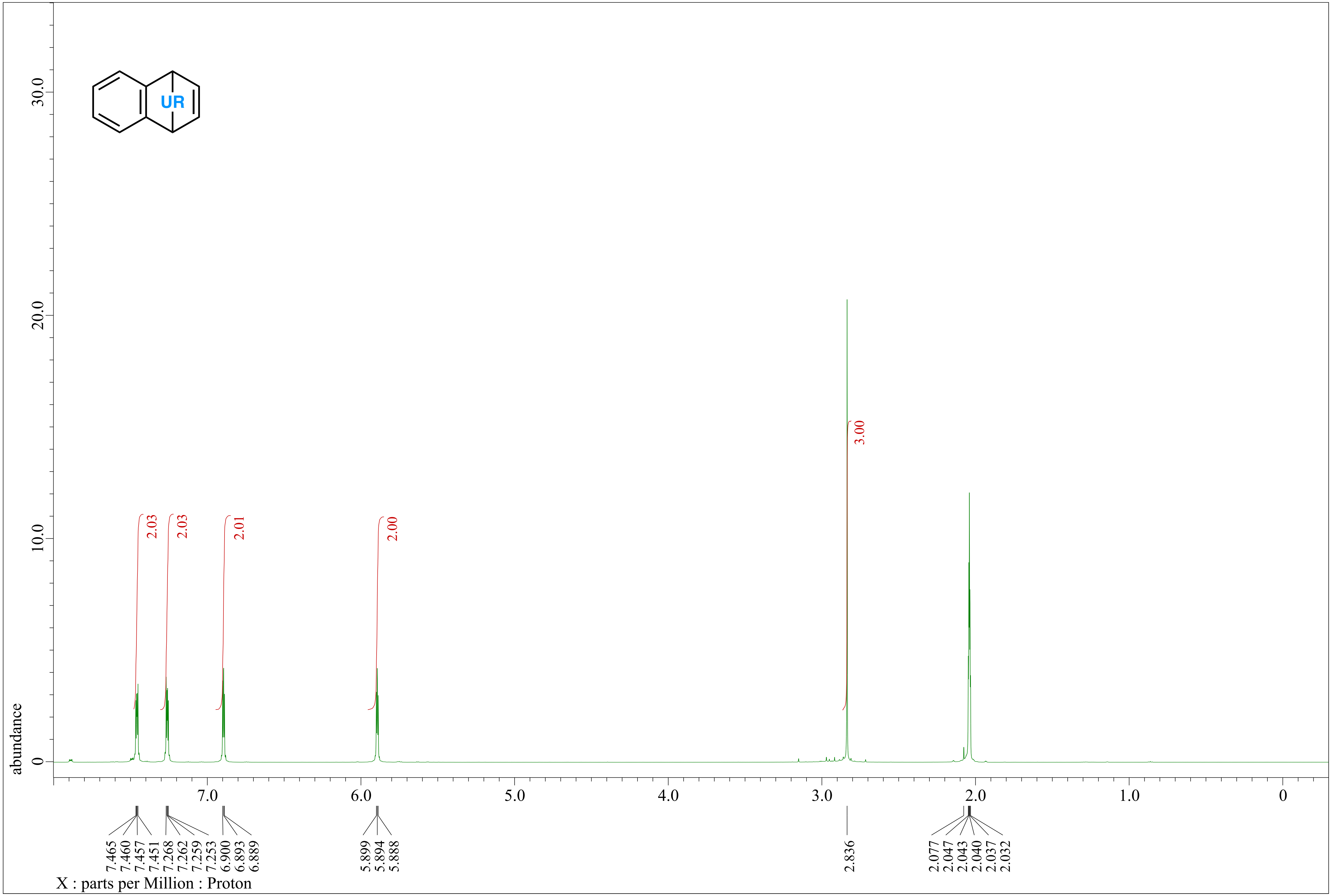

^{13}C NMR spectrum of **2g** (solvent: acetone-d_6)

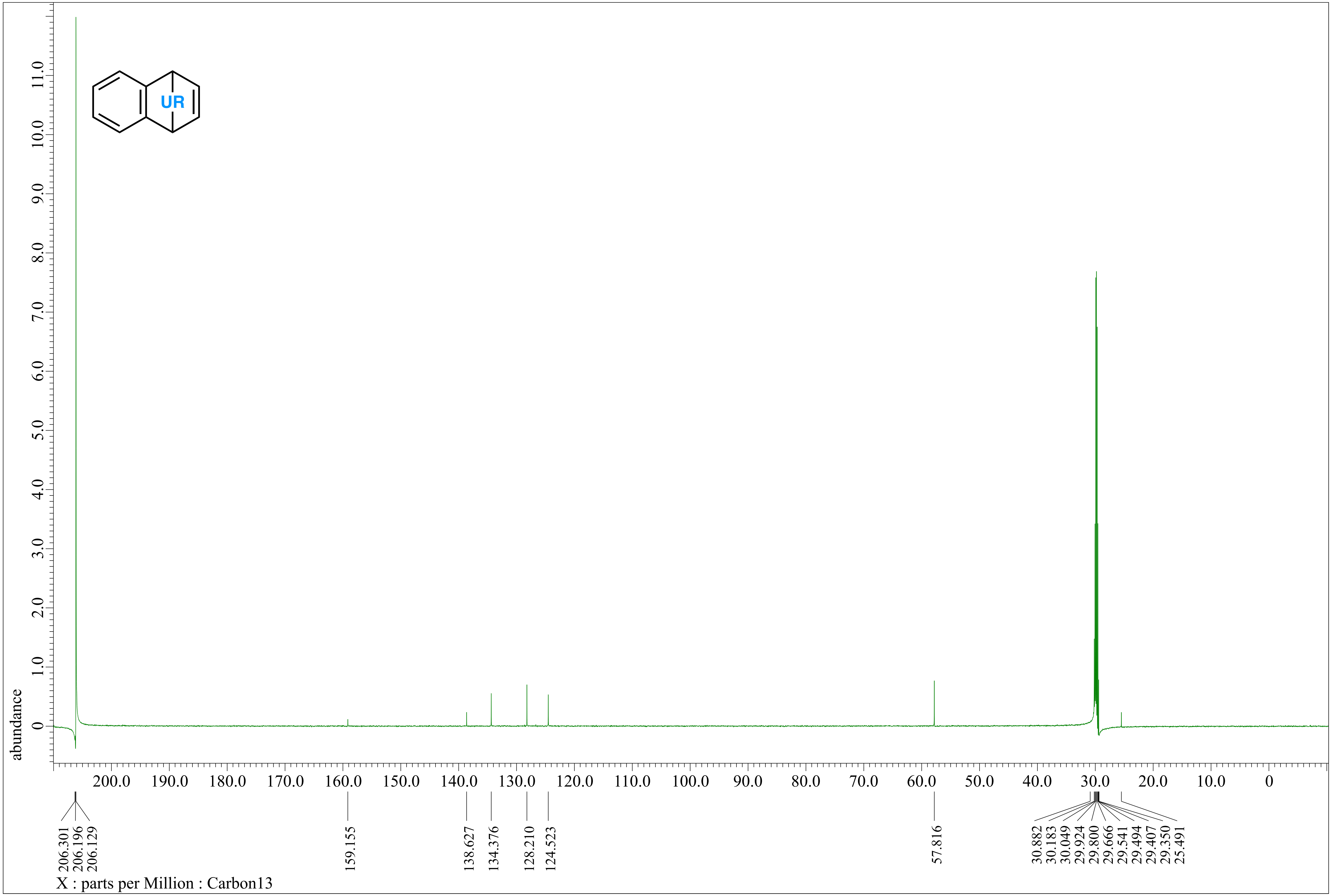

^{1}H NMR spectrum of **4aa** (solvent: CD_2Cl_2)

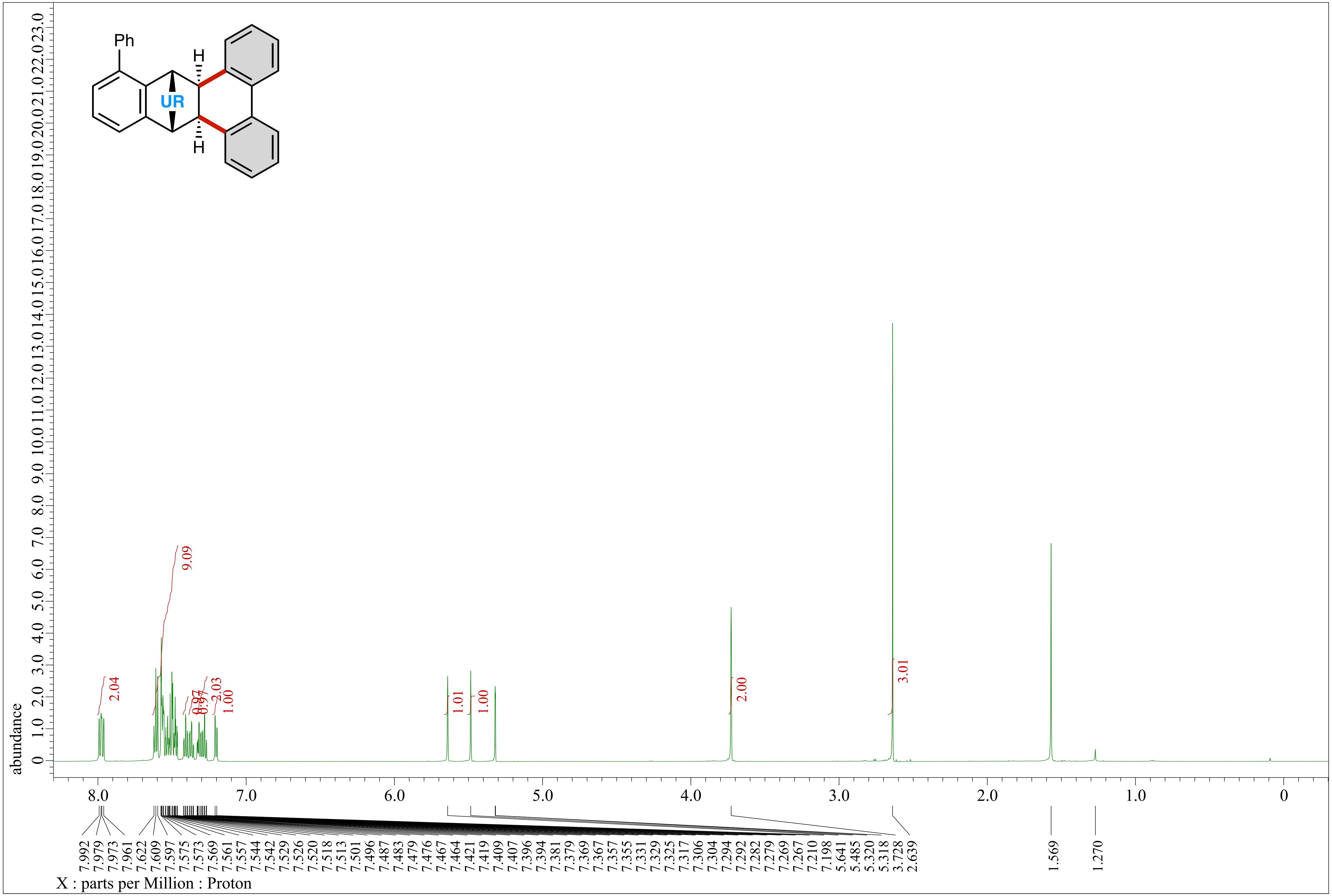

^{1}H NMR spectrum (inset) of **4aa** (solvent: CD_2Cl_2)

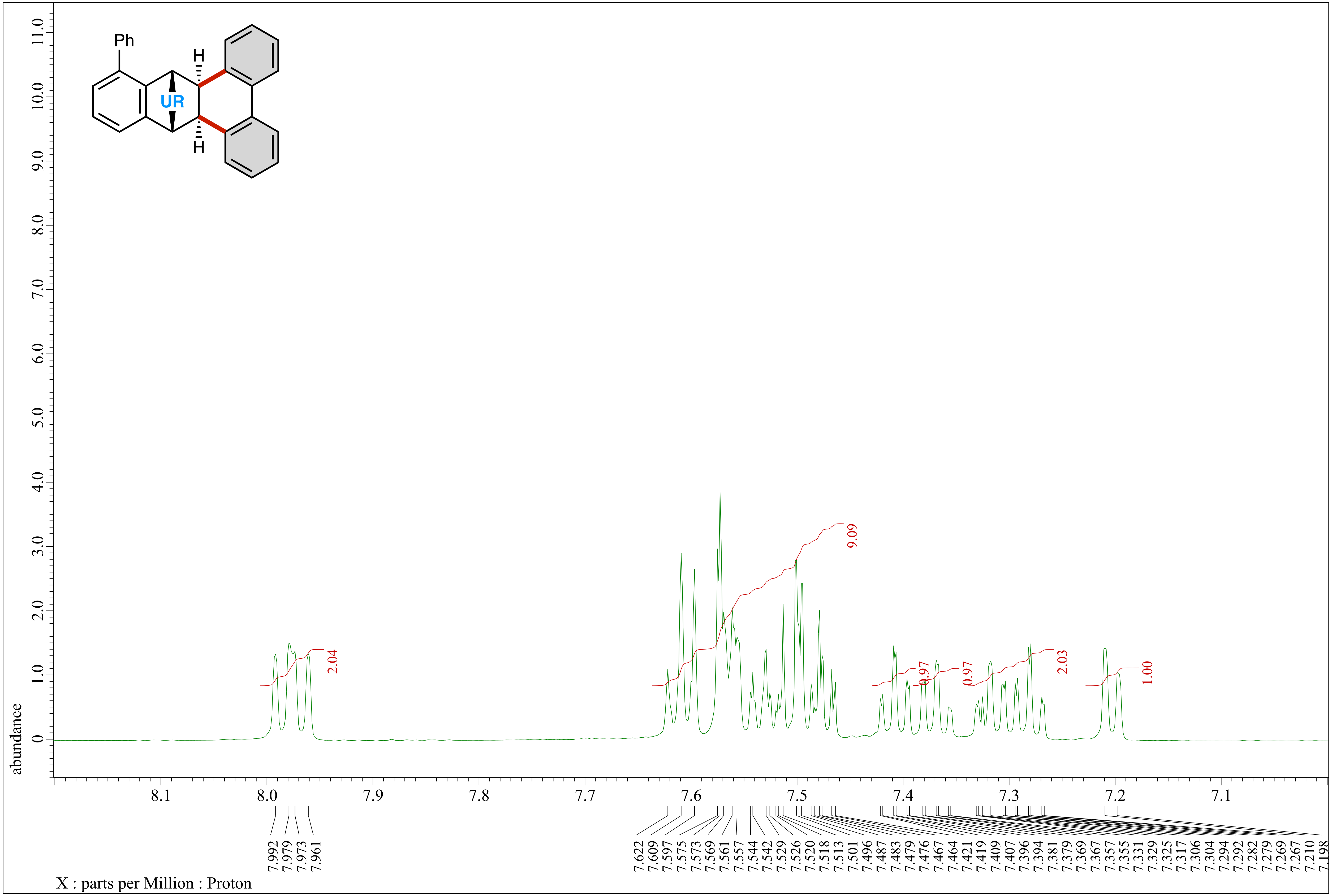

^{13}C NMR spectrum of **4aa** (solvent: CD_2Cl_2)

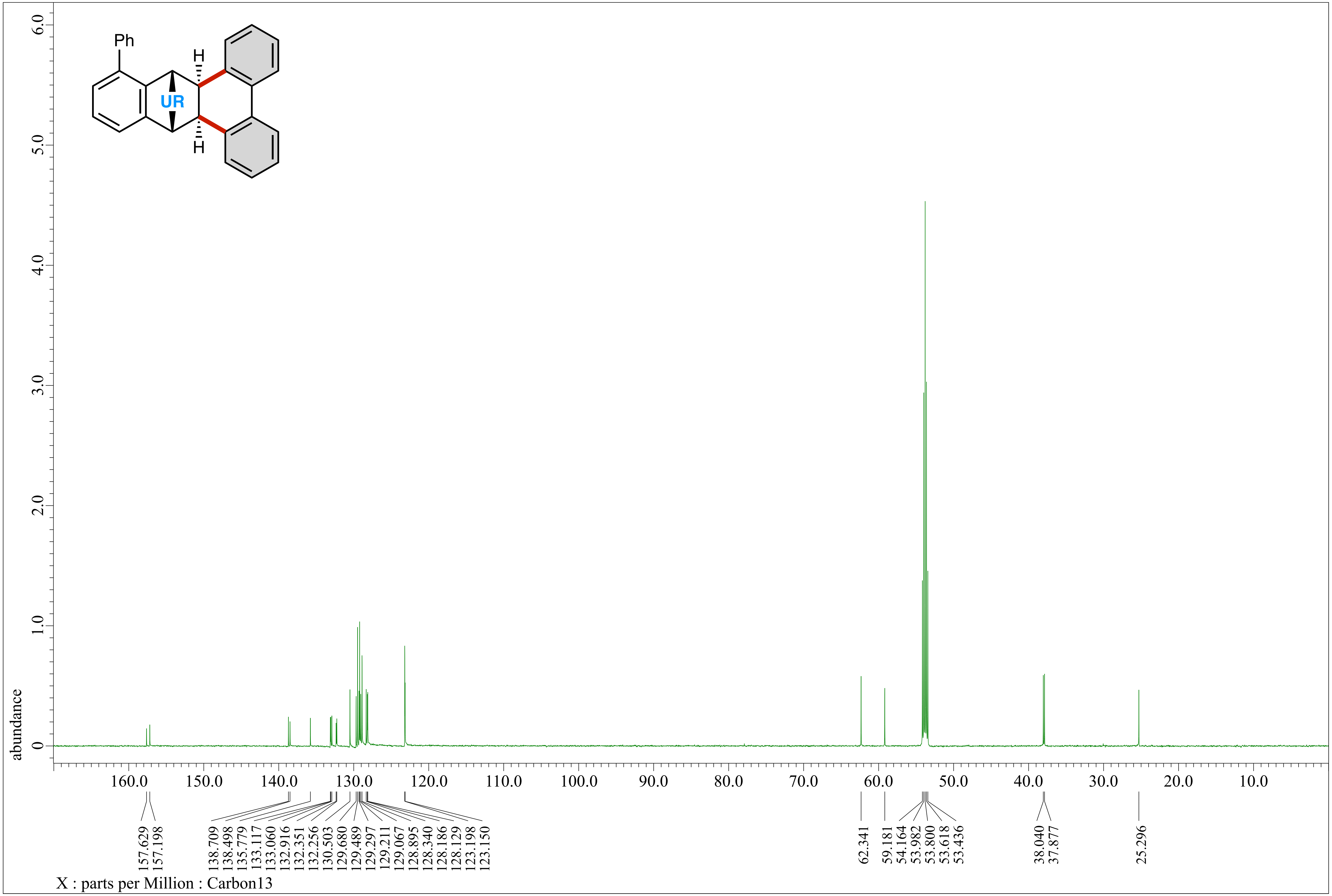

^{13}C NMR spectrum (inset) of **4aa** (solvent: CD_2Cl_2)

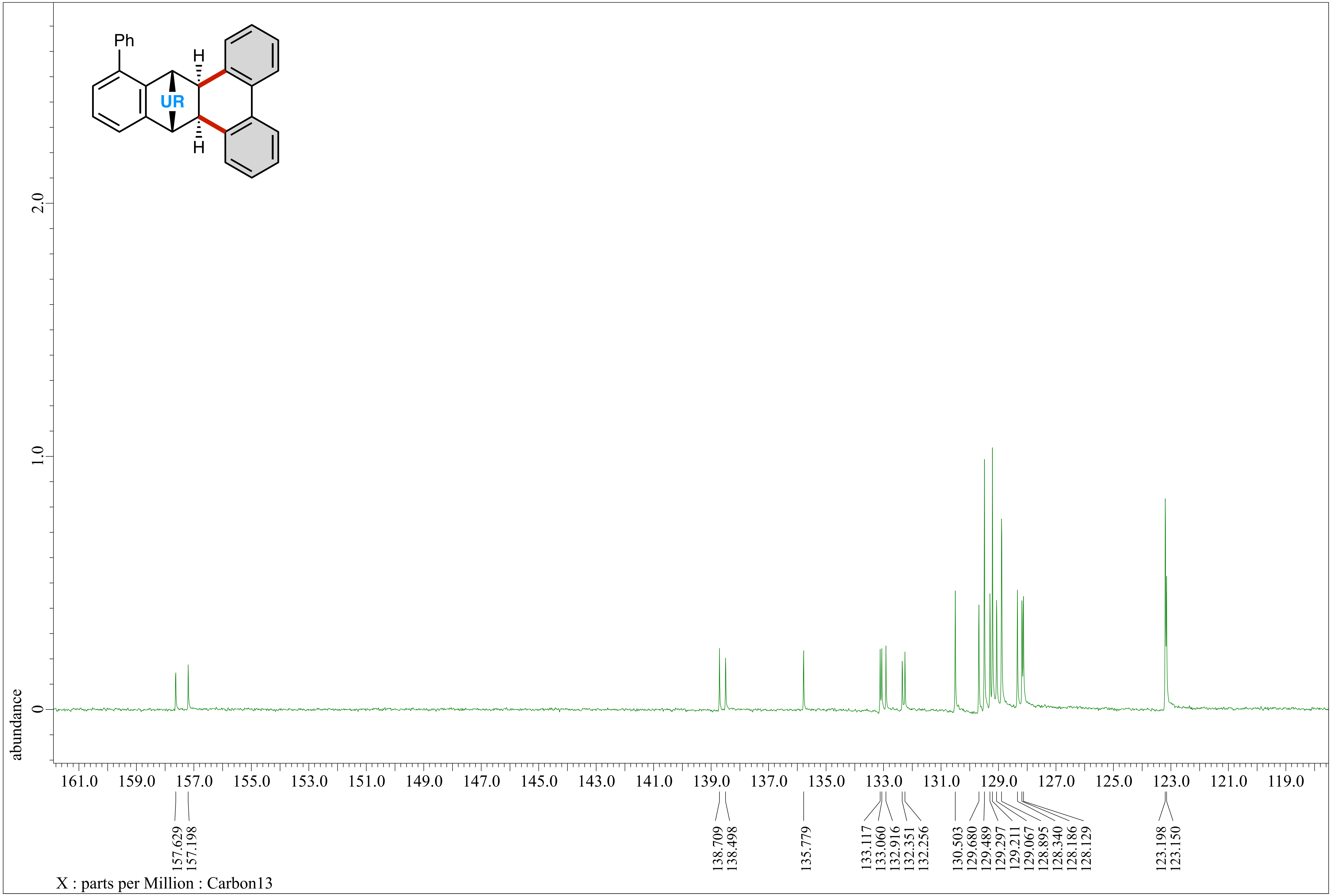

^1H NMR spectrum of **4ba** (solvent: $Cl_2CDCDCl_2$)

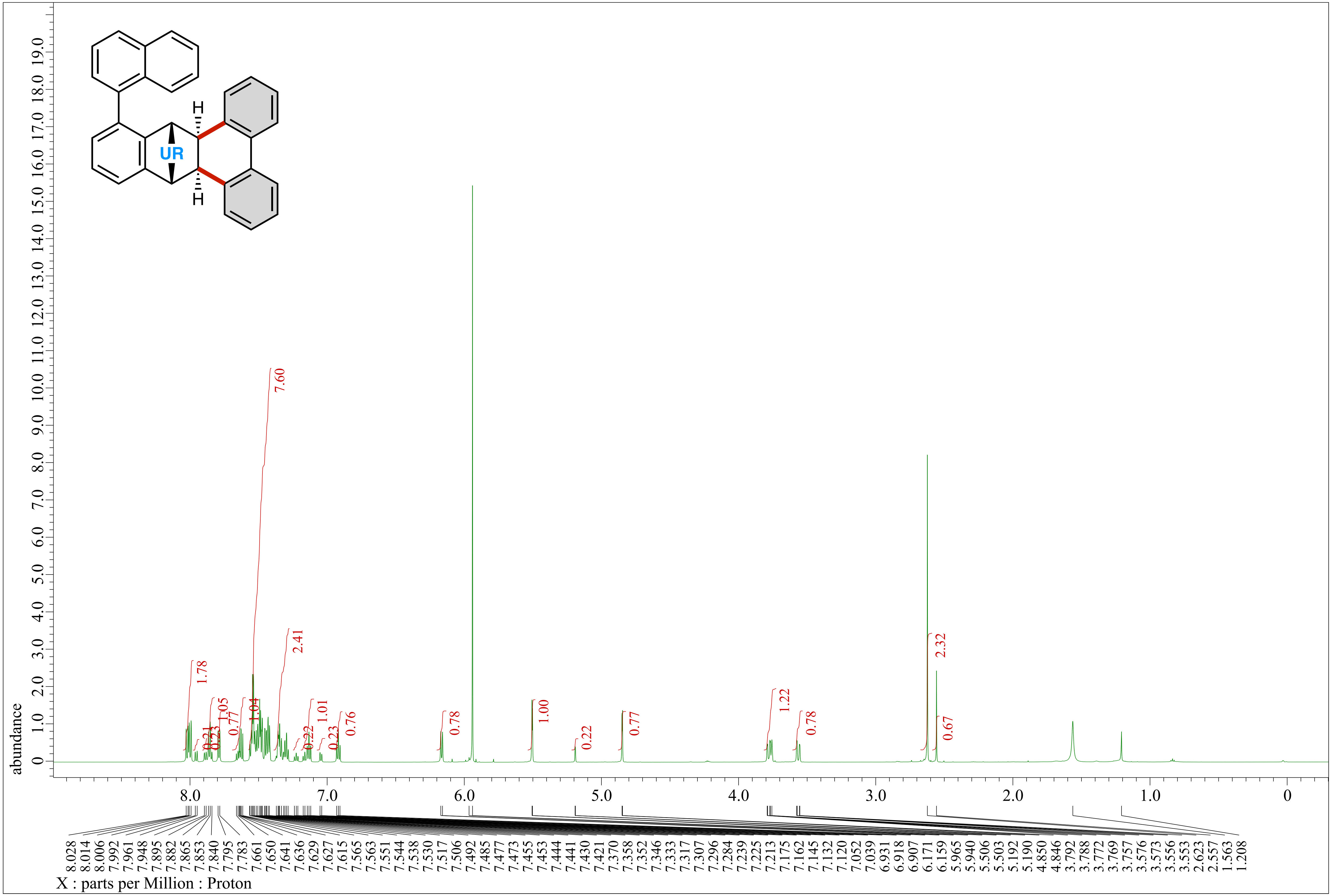

^{1}H NMR spectrum (inset) of **4ba** (solvent: $Cl_2CDCDCl_2$)

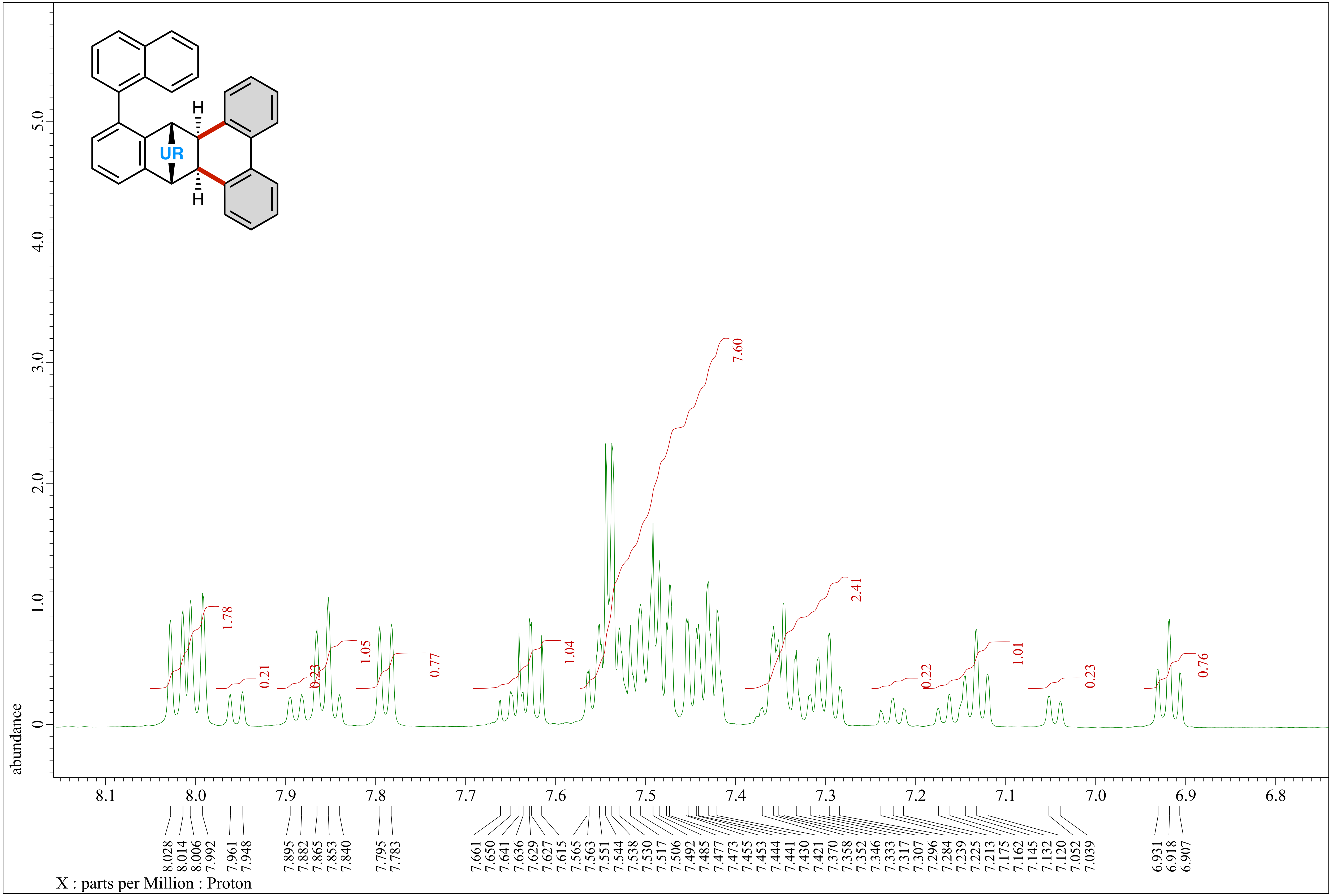

^{13}C NMR spectrum of **4ba** (solvent: $Cl_2CDCDCl_2$)

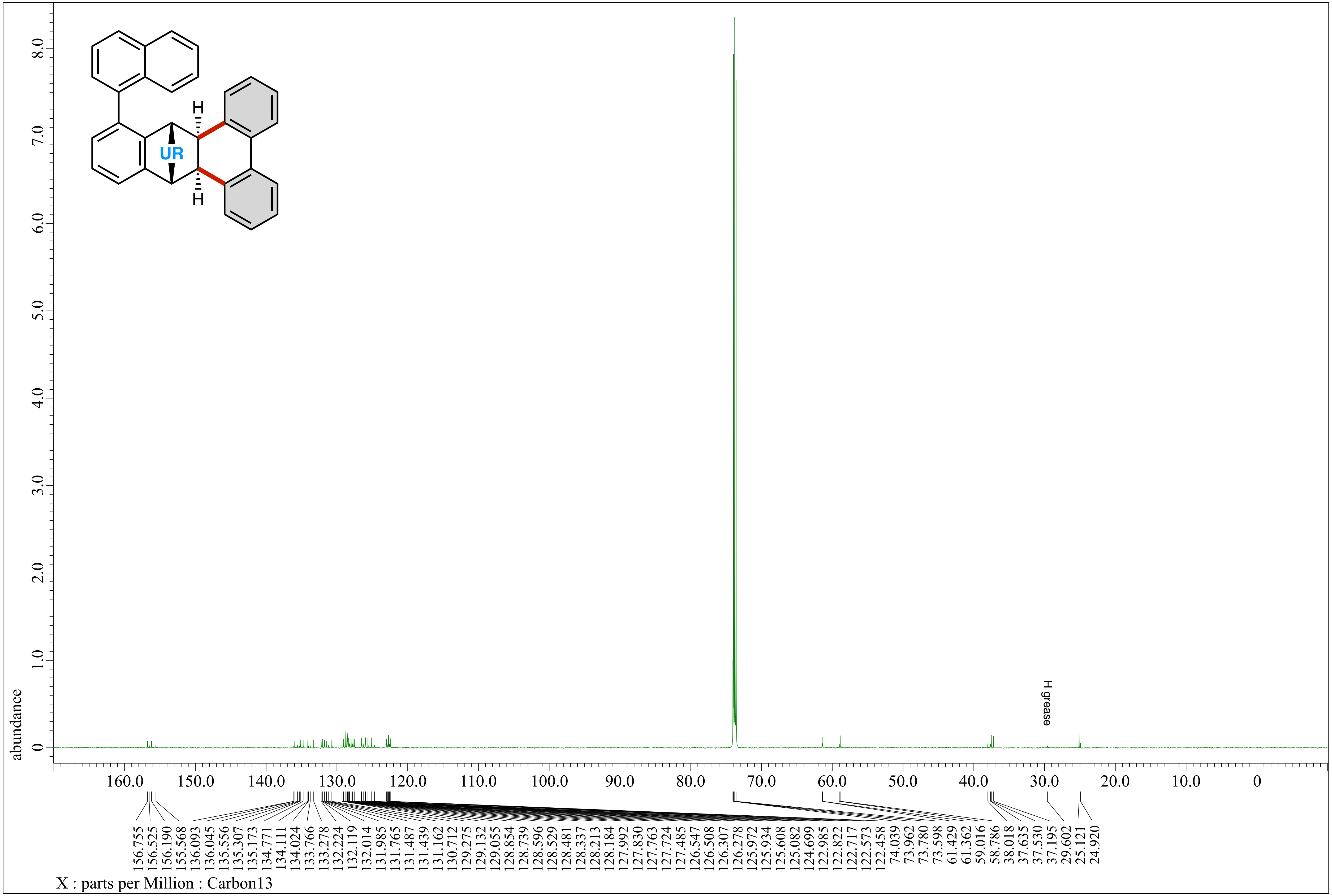

^{13}C NMR spectrum (inset) of **4ba** (solvent: $Cl_2CDCDCl_2$)

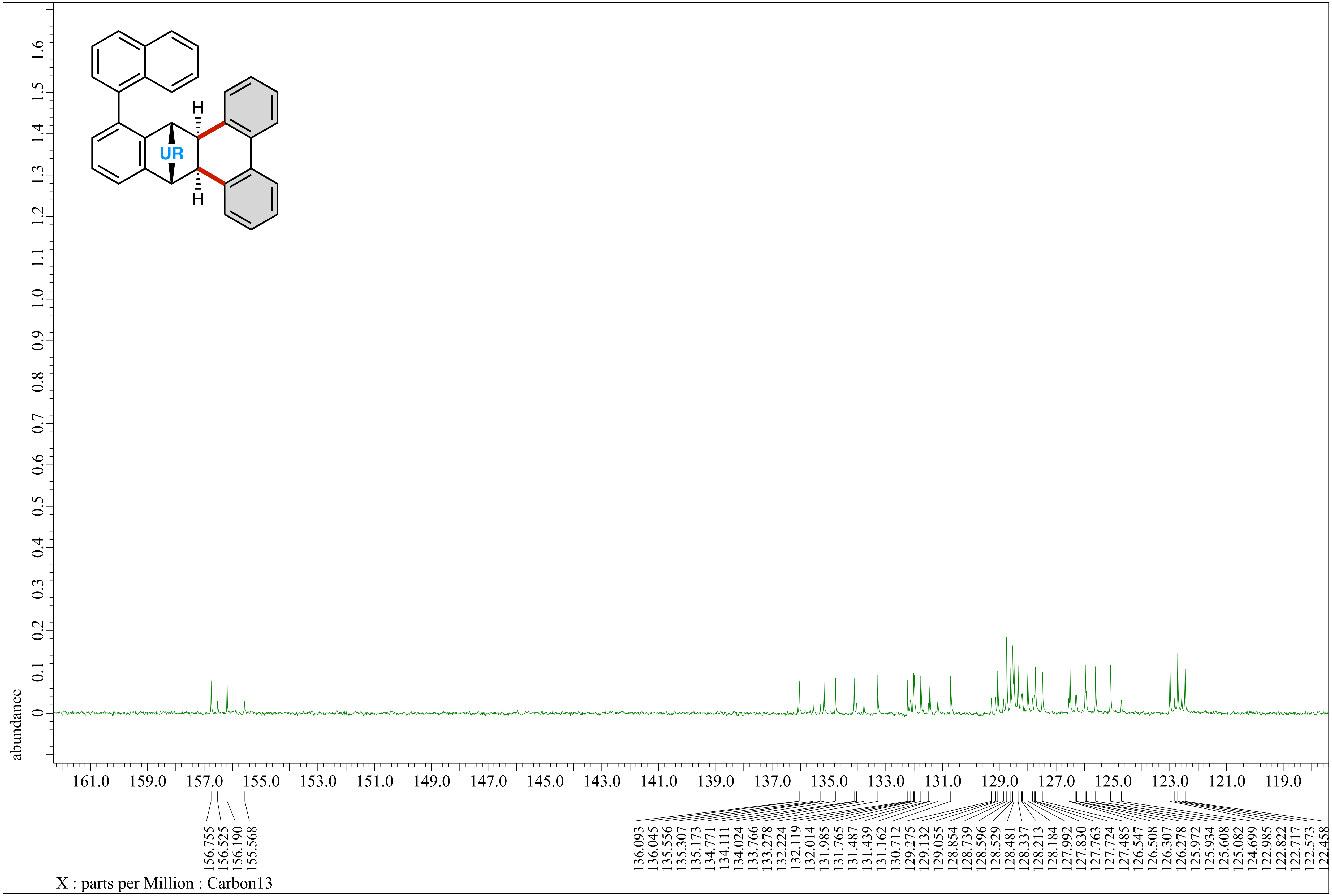

^{1}H NMR spectrum of **4ca** (solvent: CD_2Cl_2)

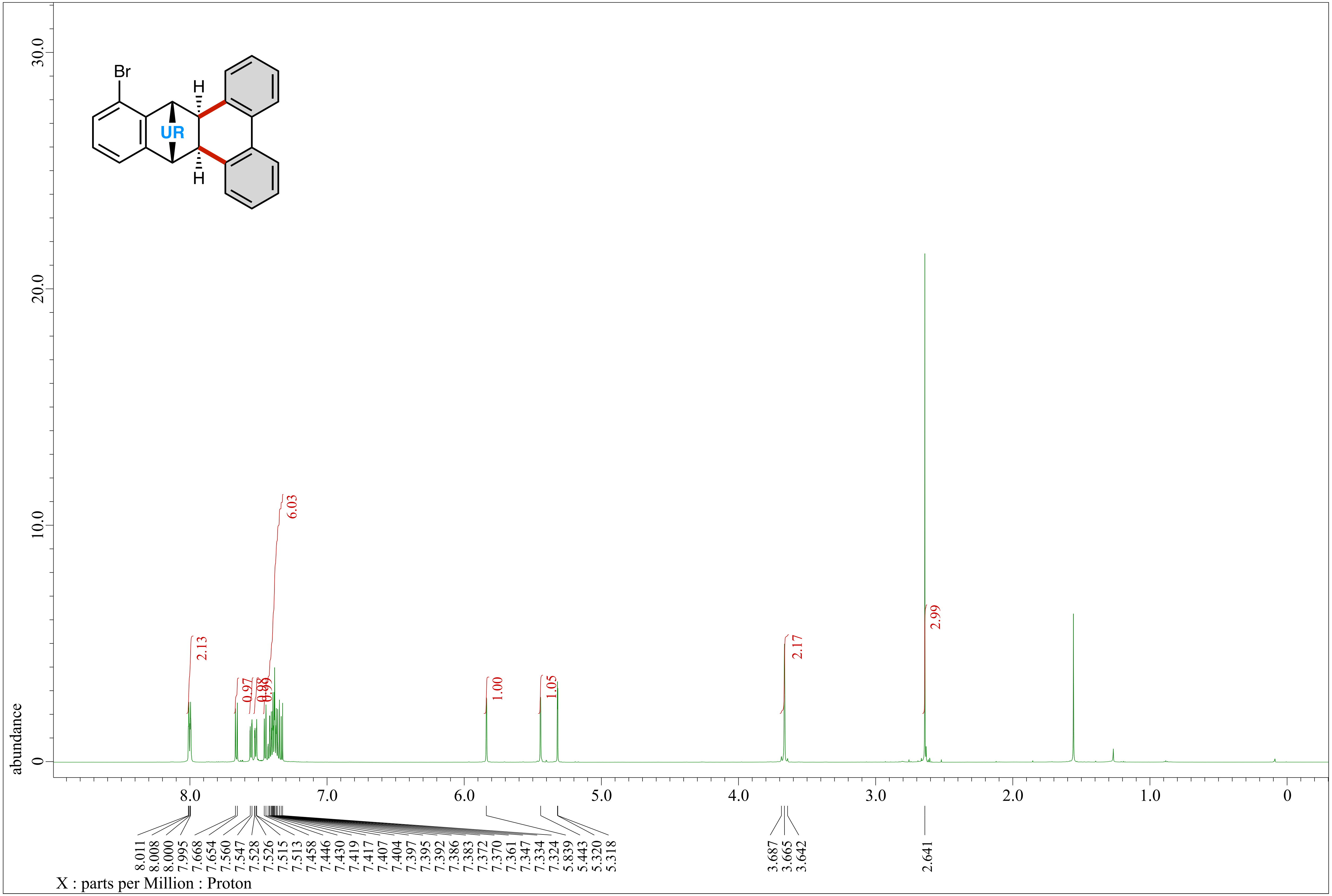

^{1}H NMR spectrum (inset) of **4ca** (solvent: CD_2Cl_2)

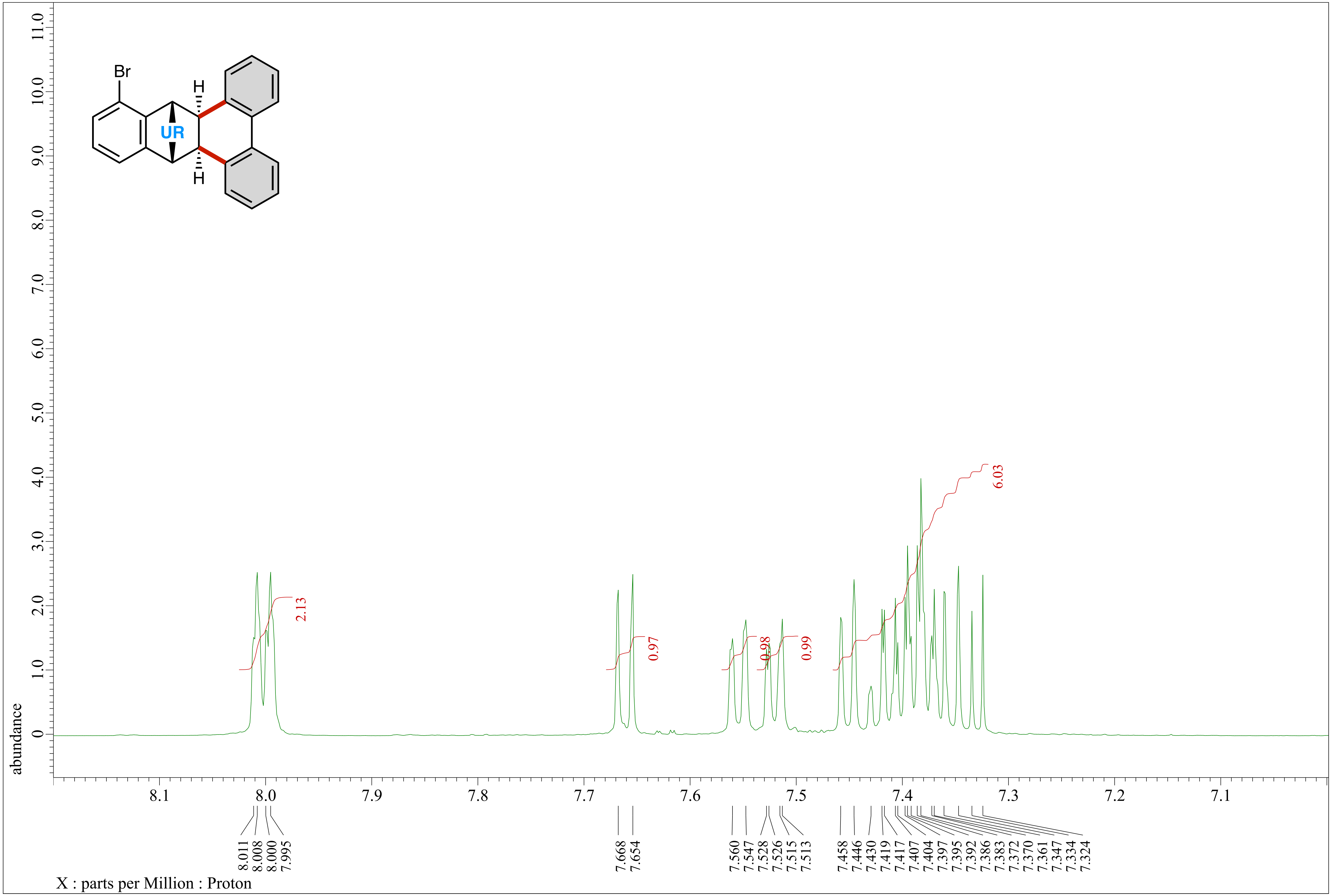

^{13}C NMR spectrum of **4ca** (solvent: CD_2Cl_2)

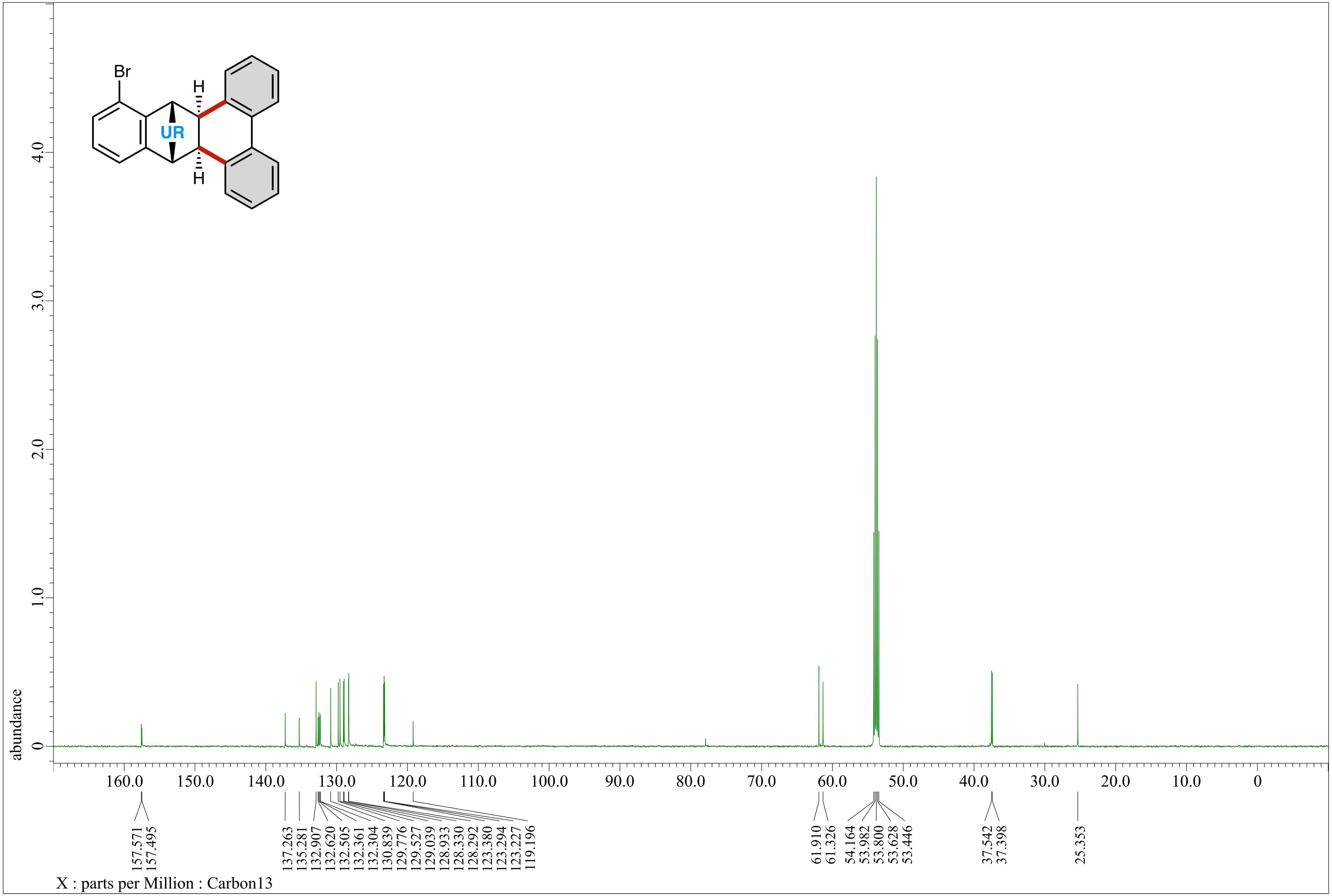

^{13}C NMR spectrum (inset) of **4ca** (solvent: CD_2Cl_2)

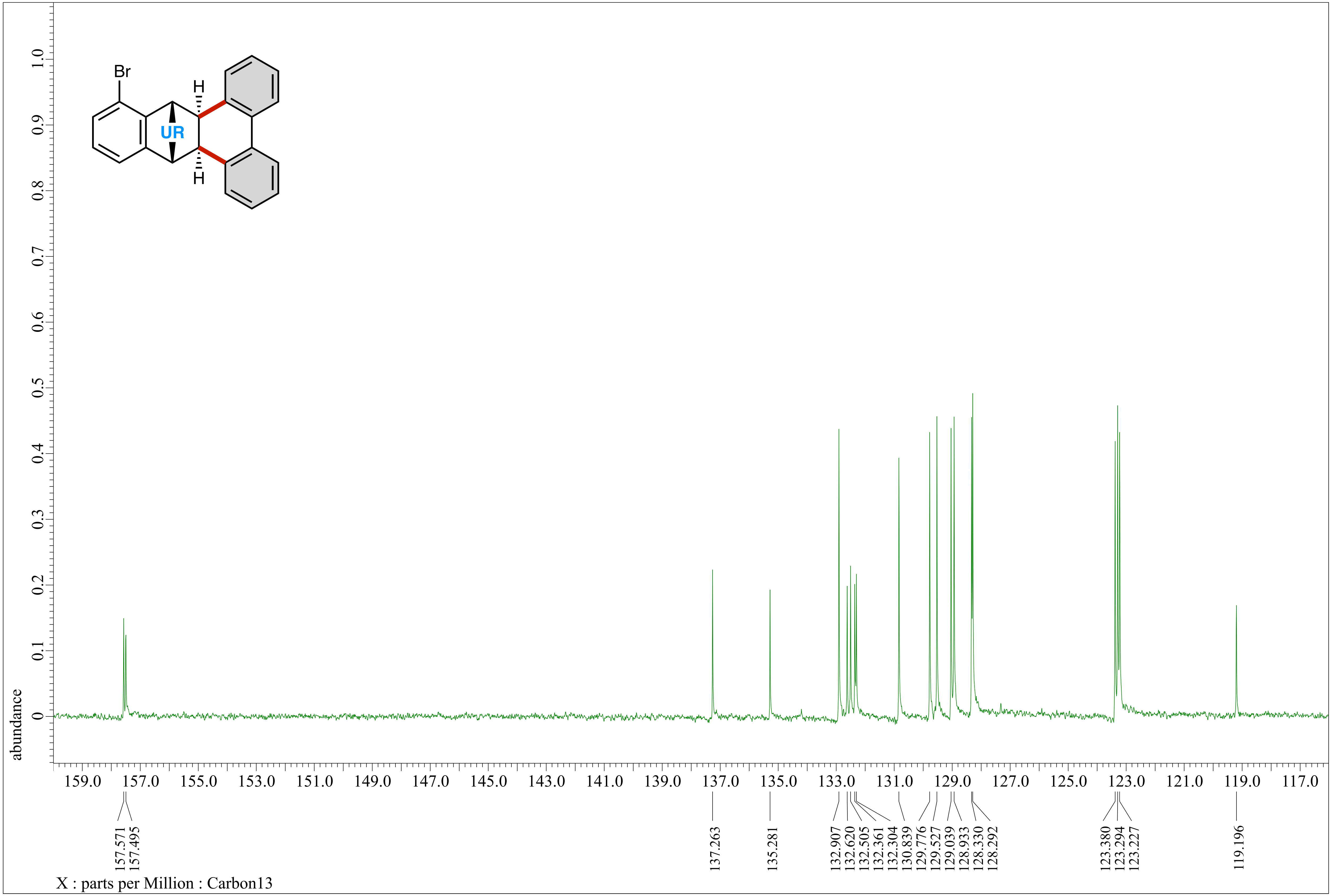

^{1}H NMR spectrum of **4da** (solvent: $CDCl_3$)

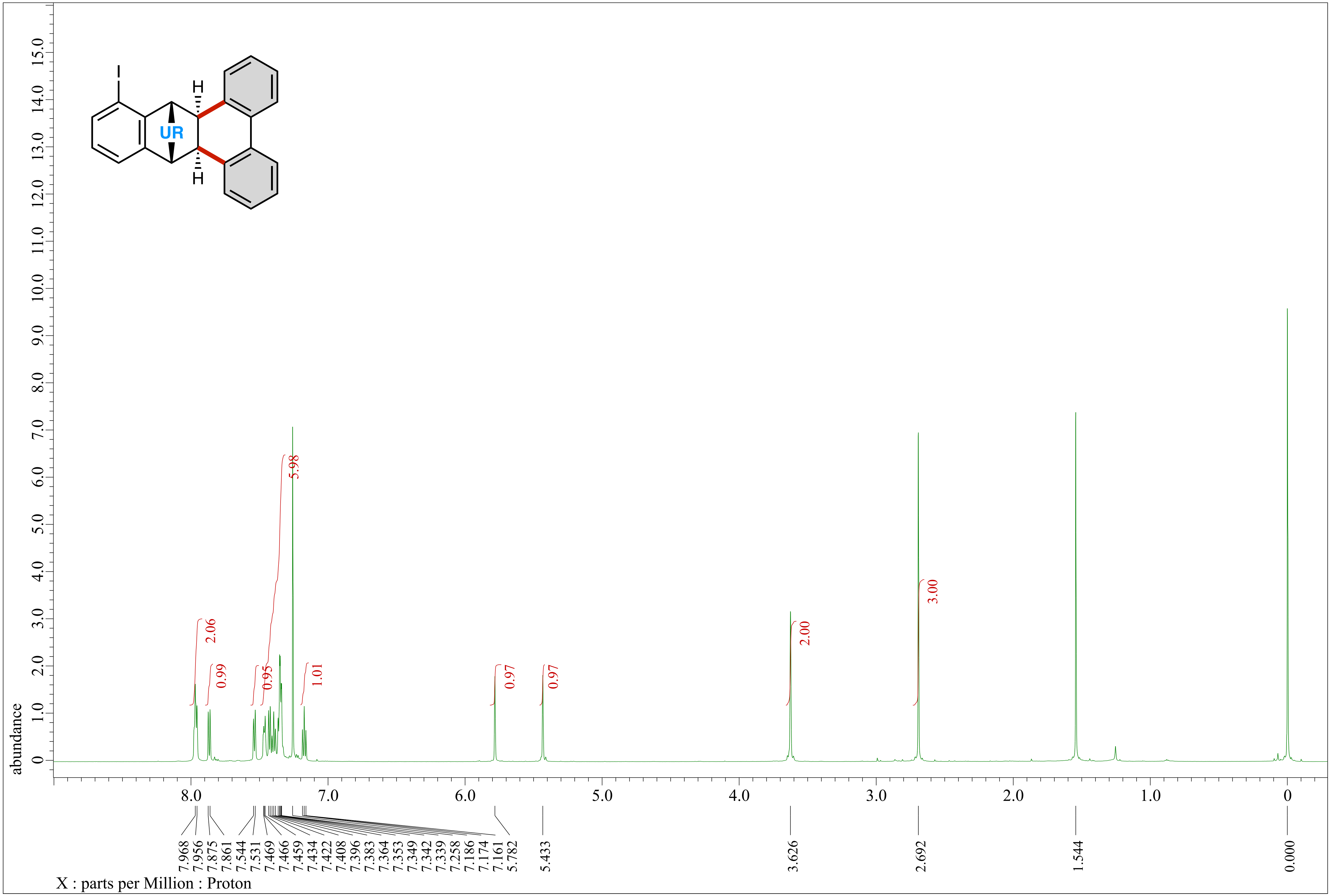

^{1}H NMR spectrum (inset) of **4da** (solvent: $CDCl_3$)

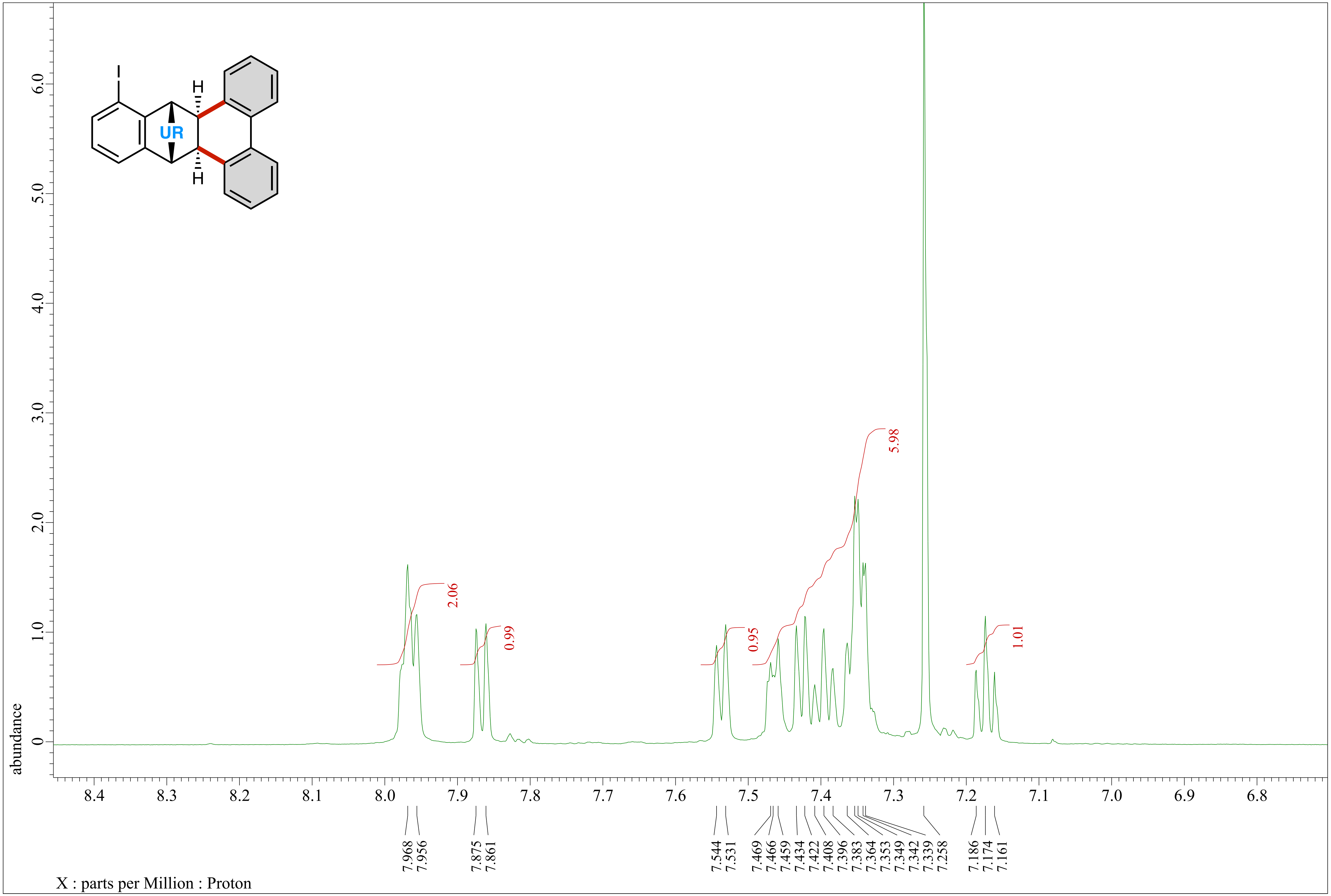

^{13}C NMR spectrum of **4da** (solvent: $CDCl_3$)

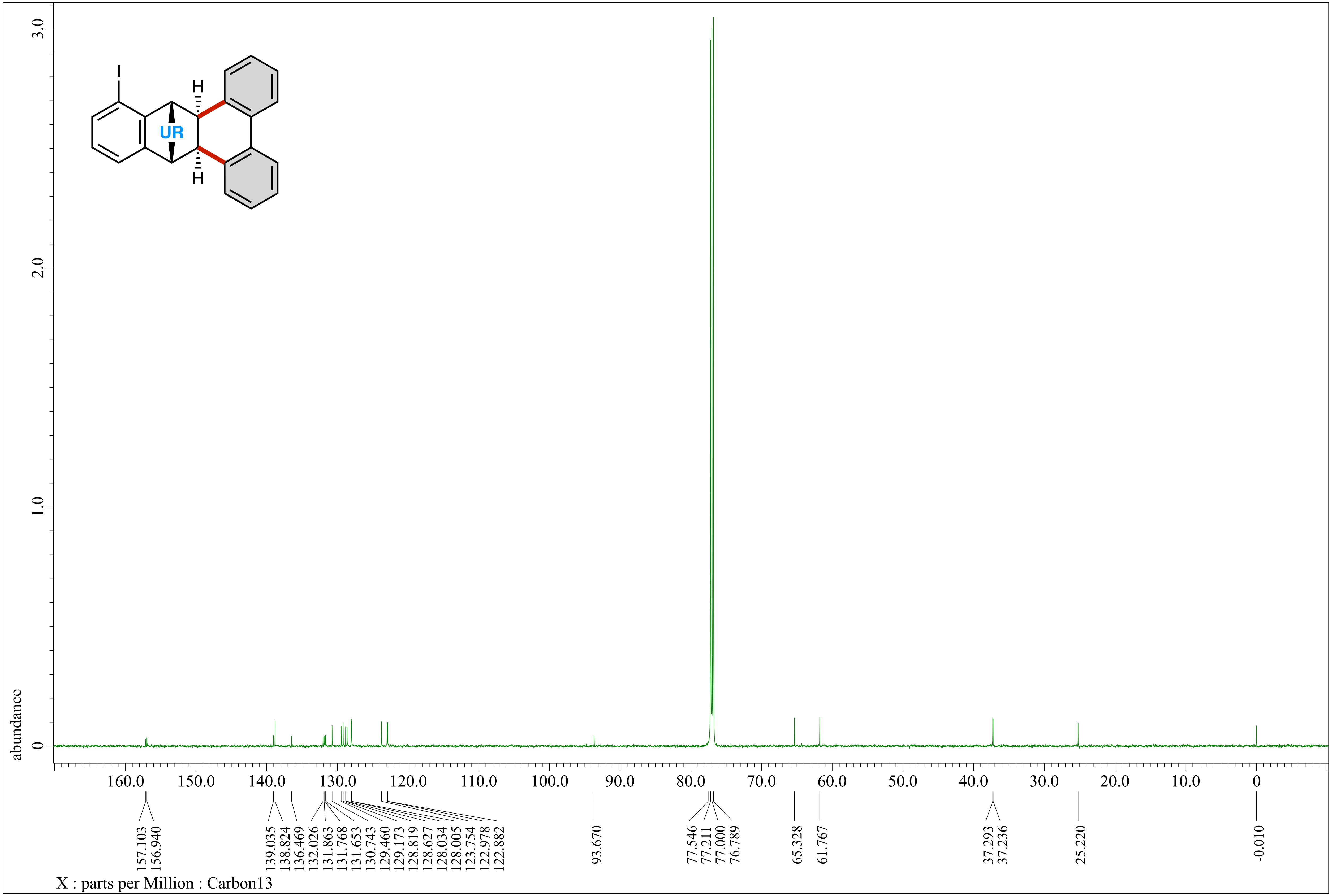

^{13}C NMR spectrum (inset) of **4da** (solvent: $CDCl_3$)

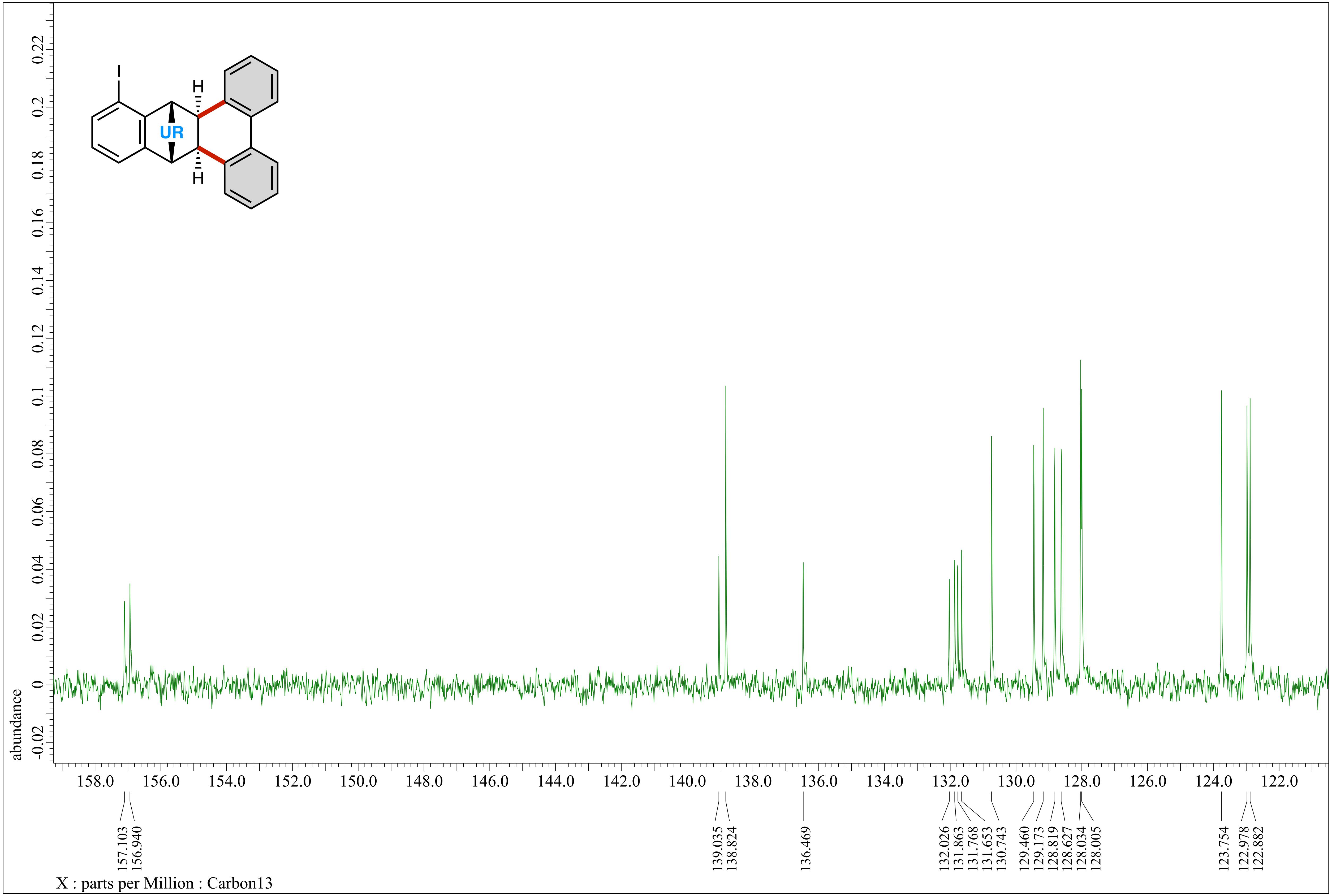

^{1}H NMR spectrum of **4ea** (solvent: $CDCl_3$)

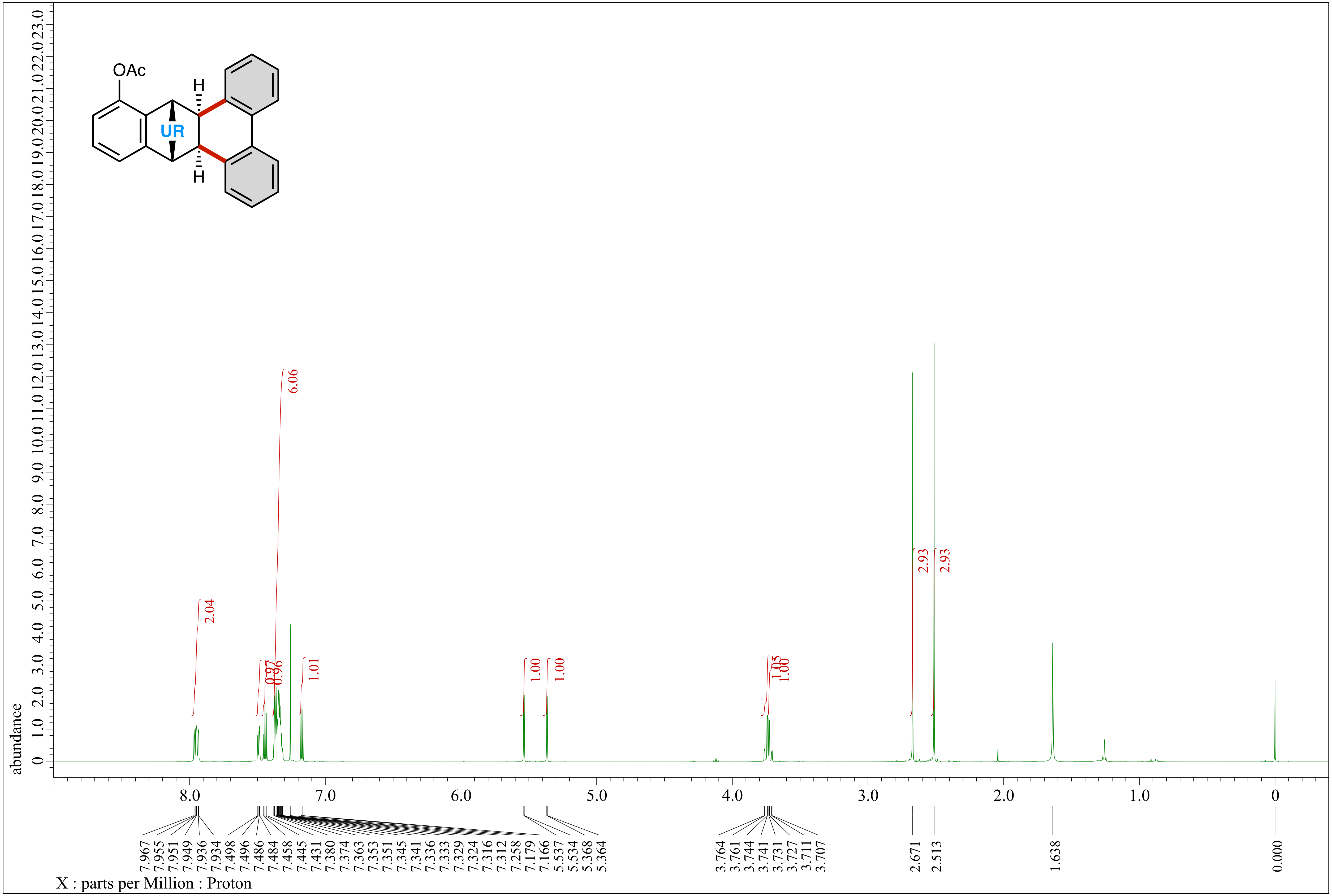

^{1}H NMR spectrum (inset) of **4ea** (solvent: $CDCl_3$)

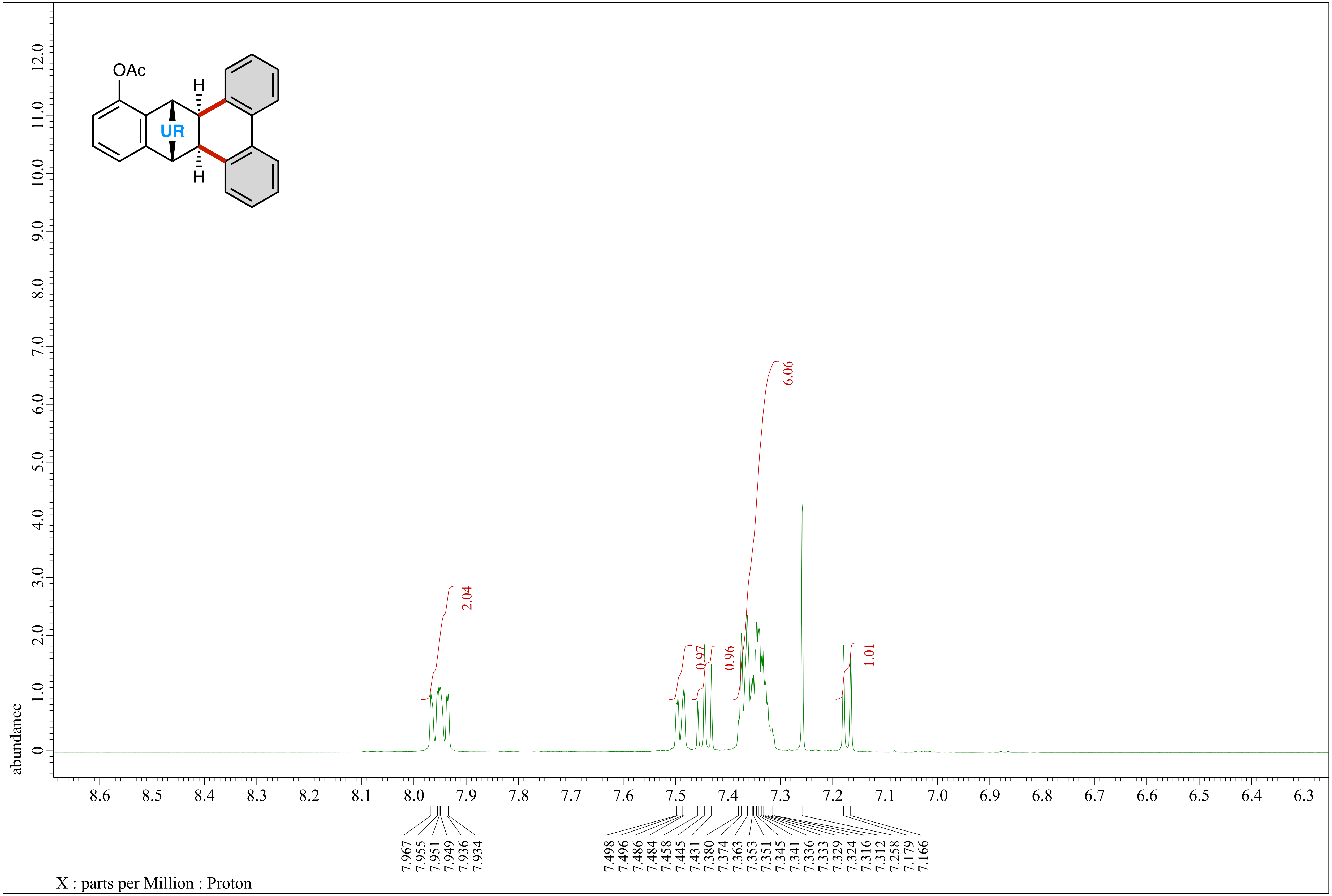

^{13}C NMR spectrum of **4ea** (solvent: $CDCl_3$)

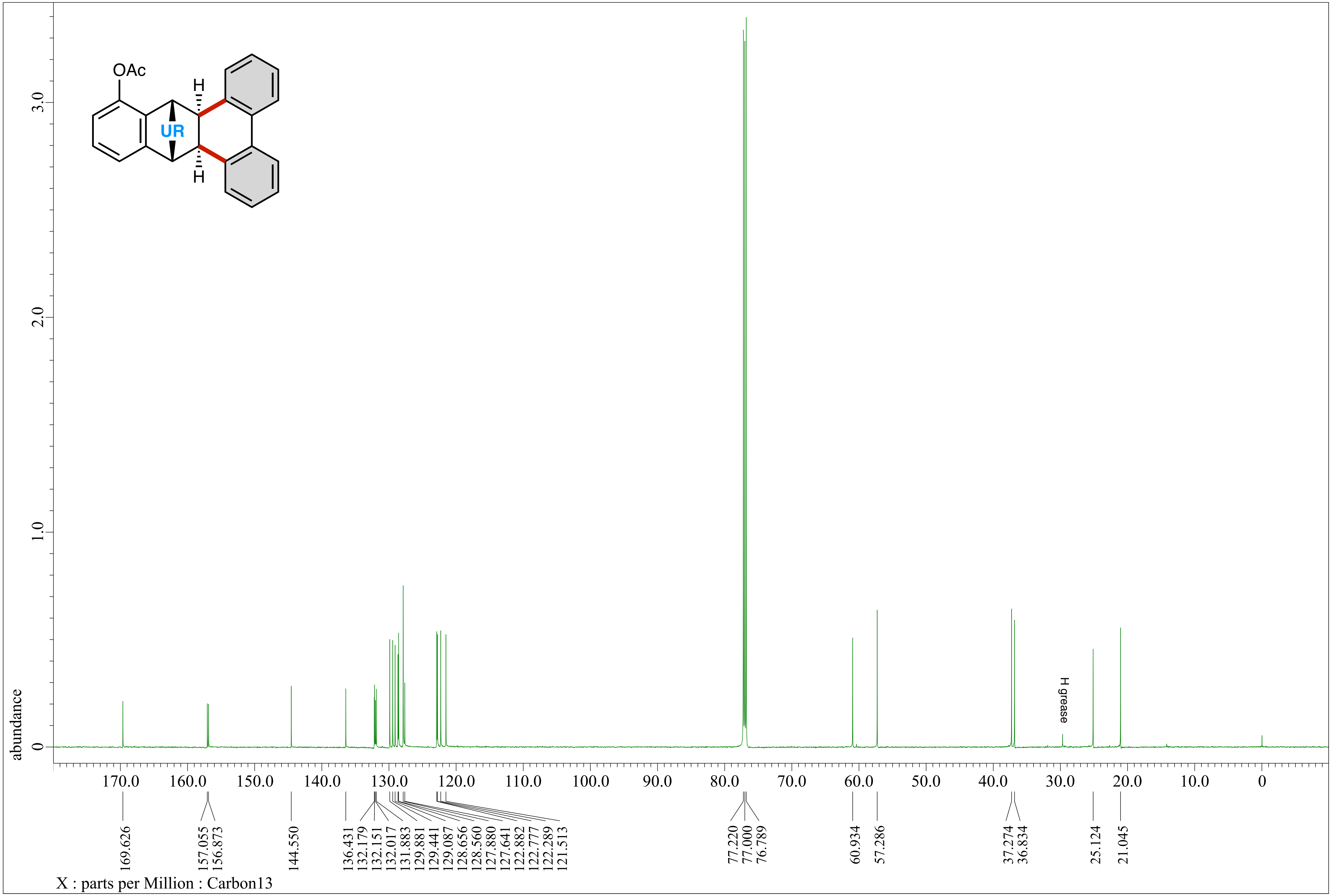

^{13}C NMR spectrum (inset) of **4ea** (solvent: $CDCl_3$)

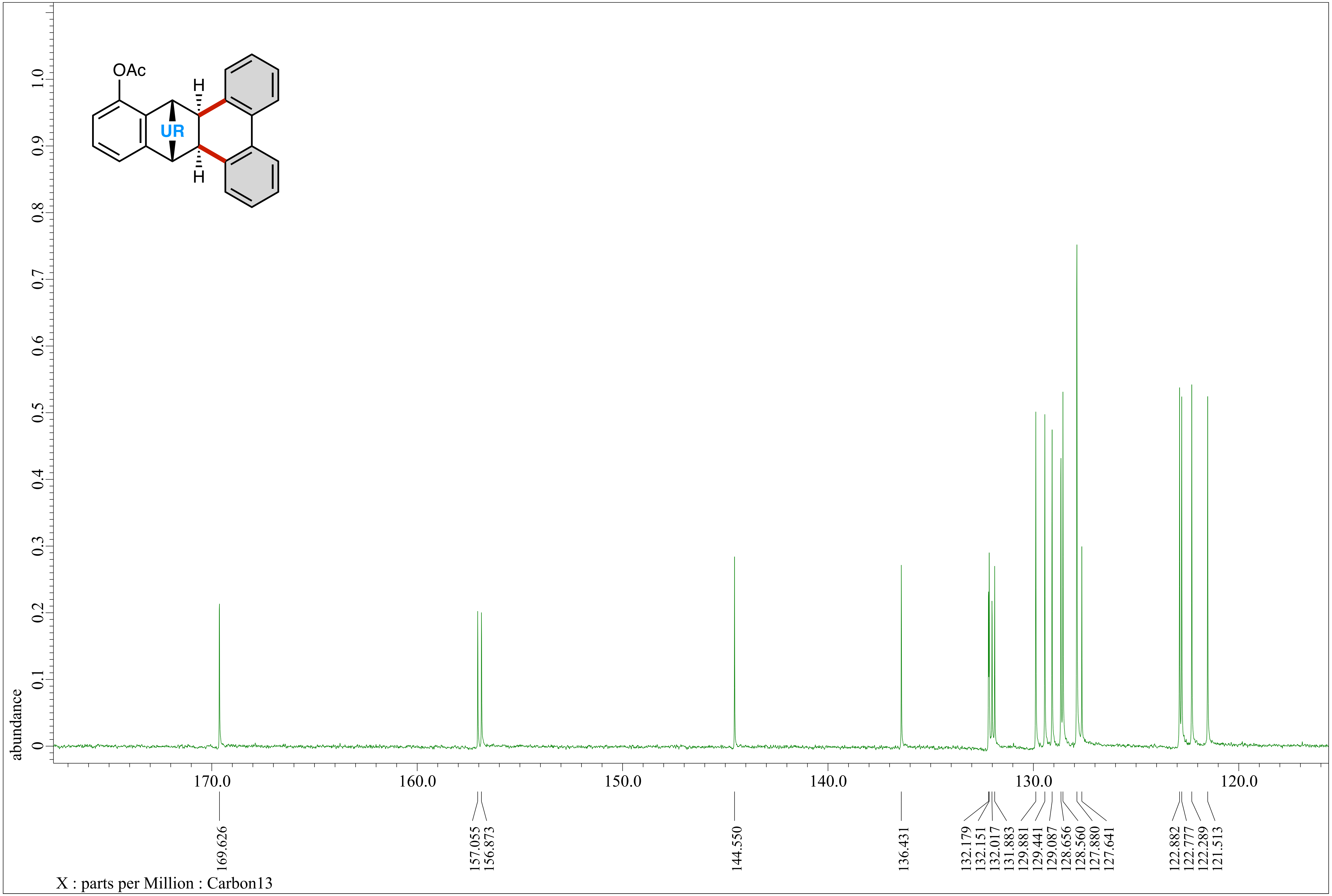

^{1}H NMR spectrum of **4fa** (solvent: $CDCl_3$)

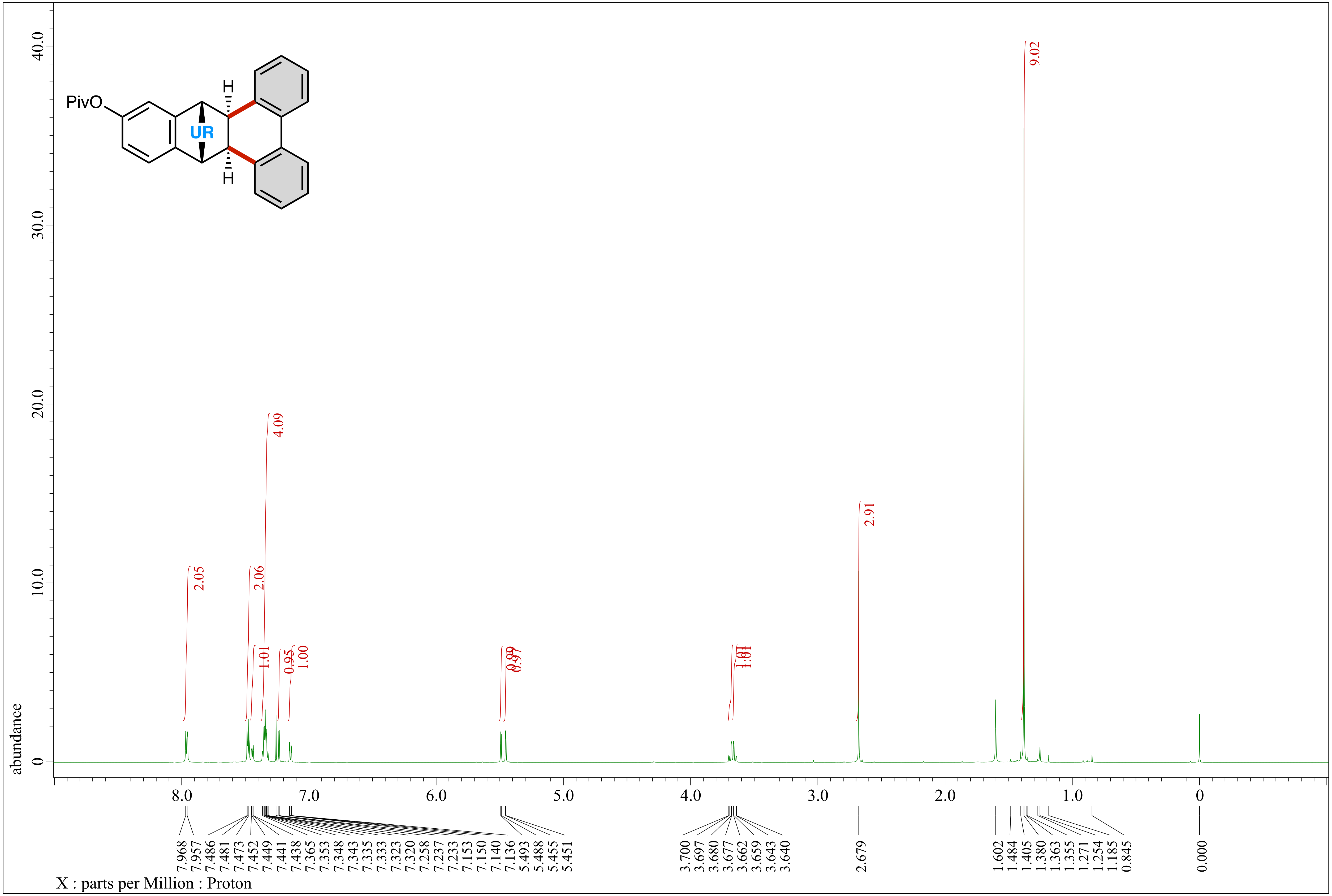

^{1}H NMR spectrum (inset) of **4fa** (solvent: $CDCl_3$)

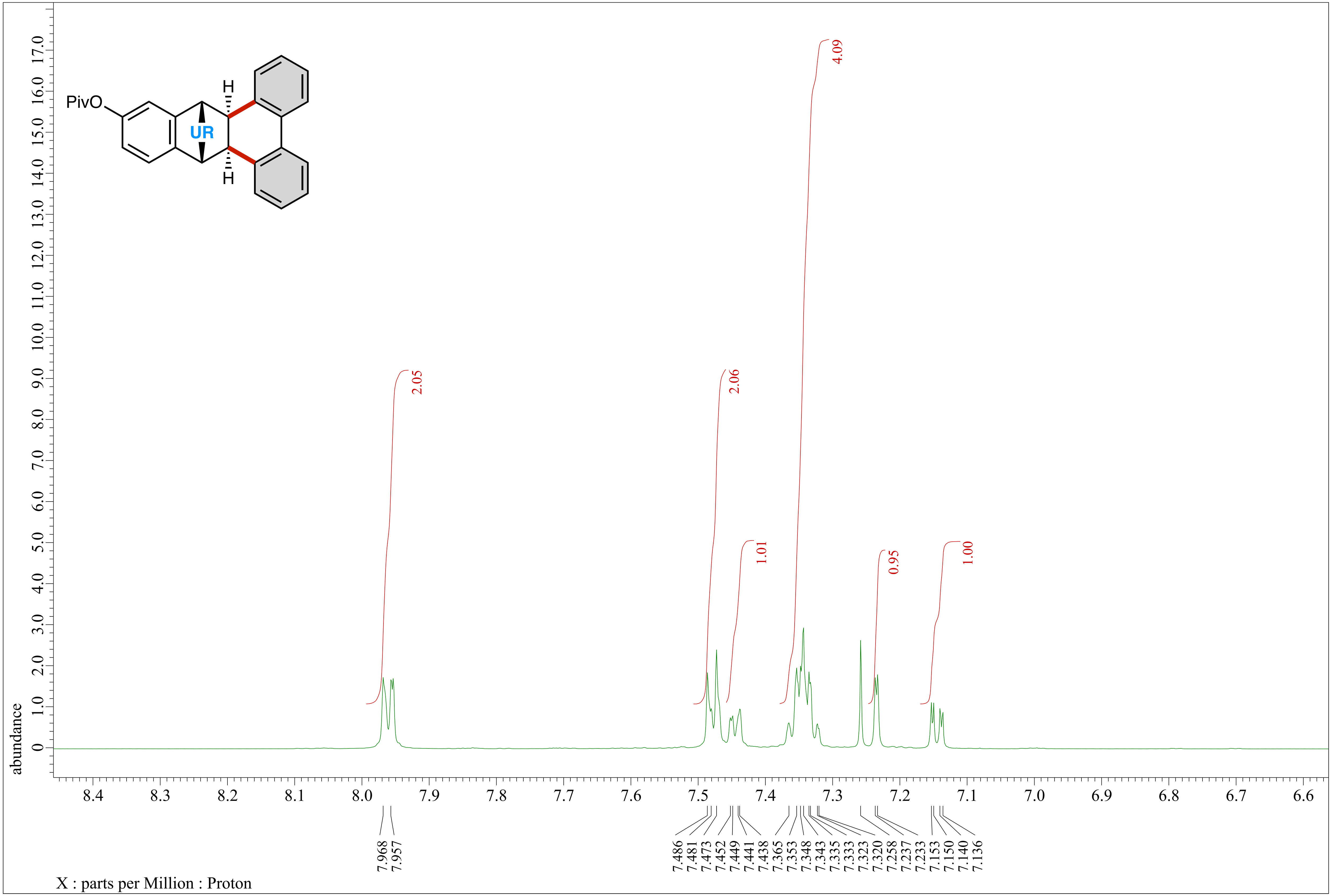

^{13}C NMR spectrum of **4fa** (solvent: $CDCl_3$)

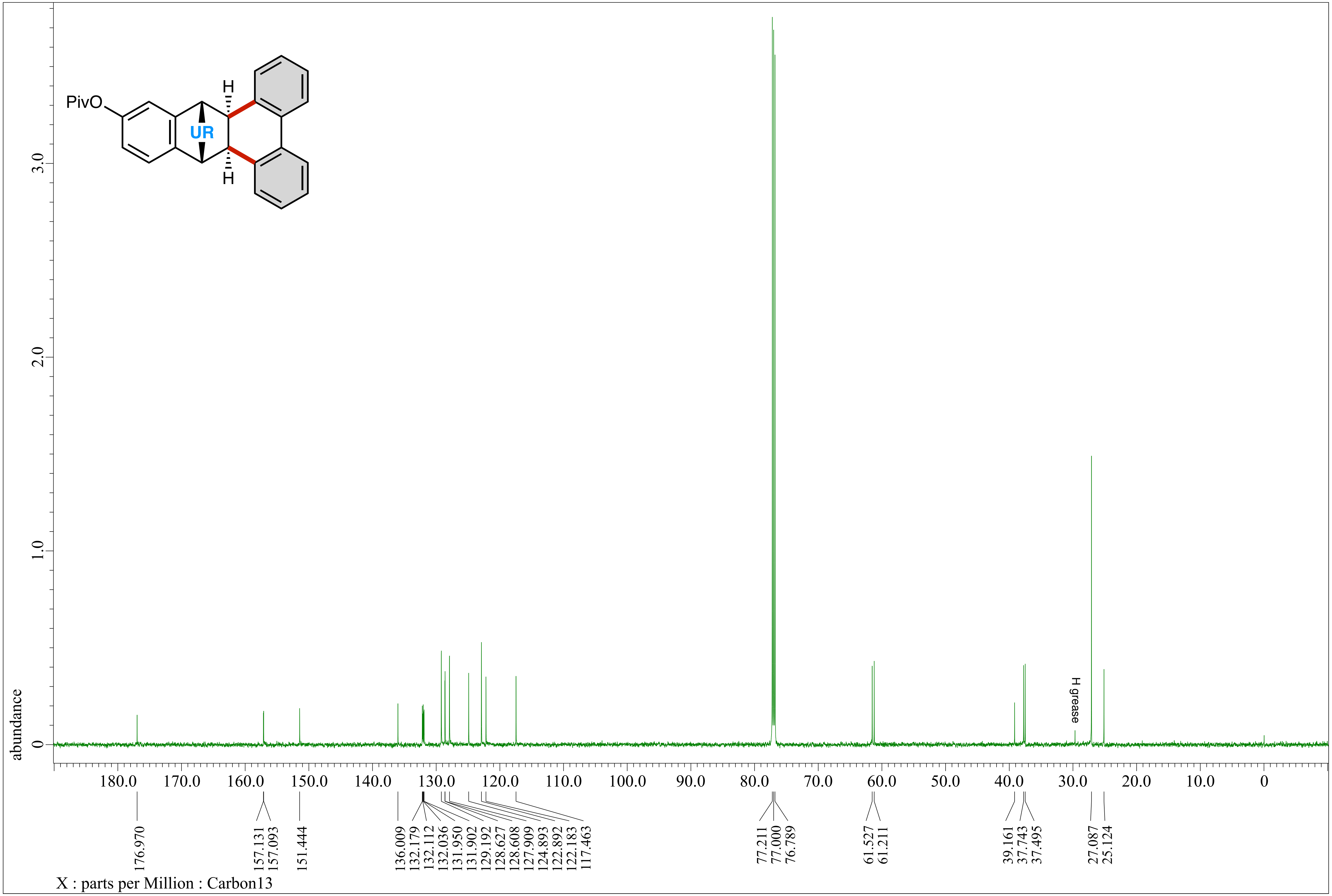

^{13}C NMR spectrum (inset) of **4fa** (solvent: $CDCl_3$)

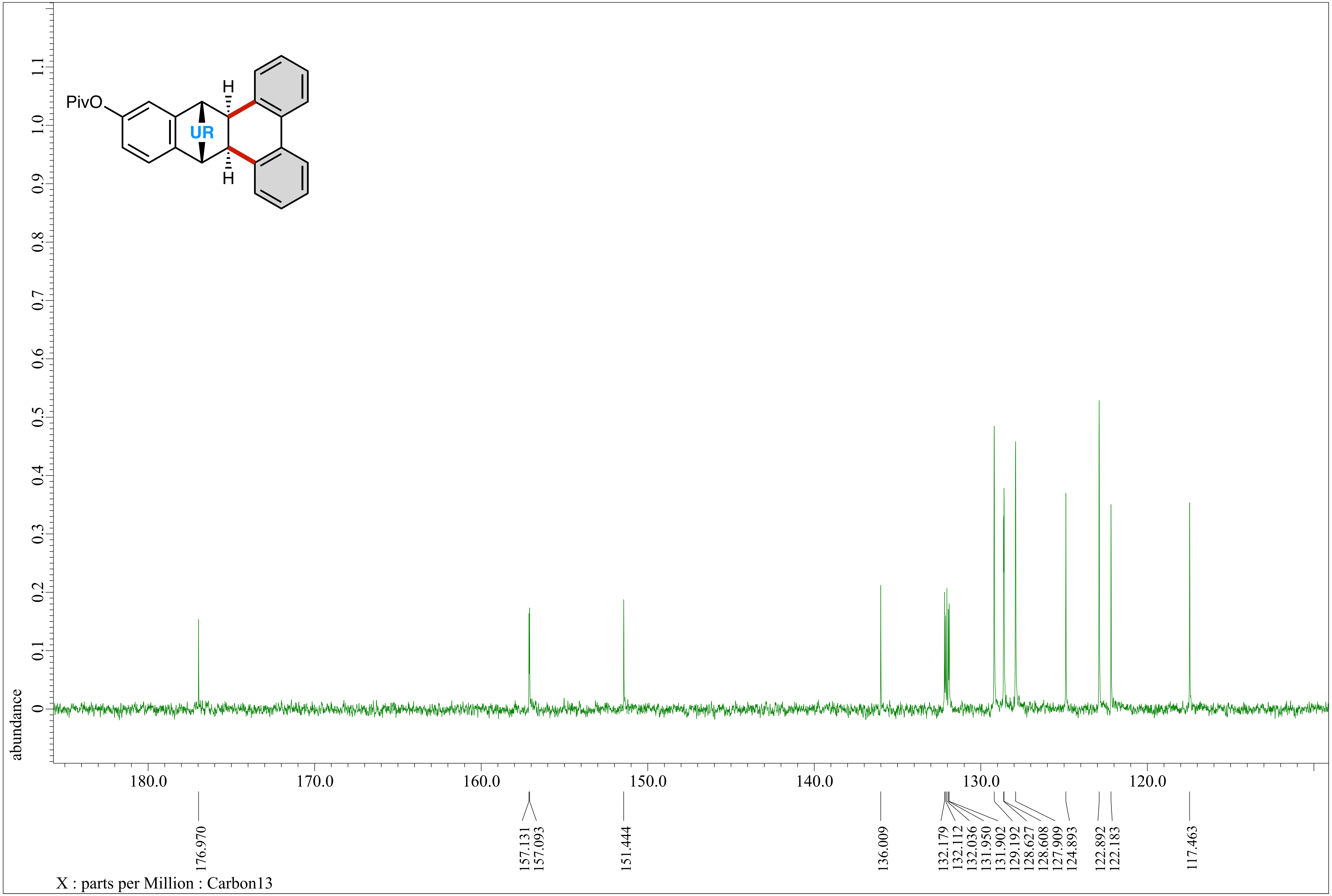

^{1}H NMR spectrum of **4ga** (solvent: $CDCl_3$)

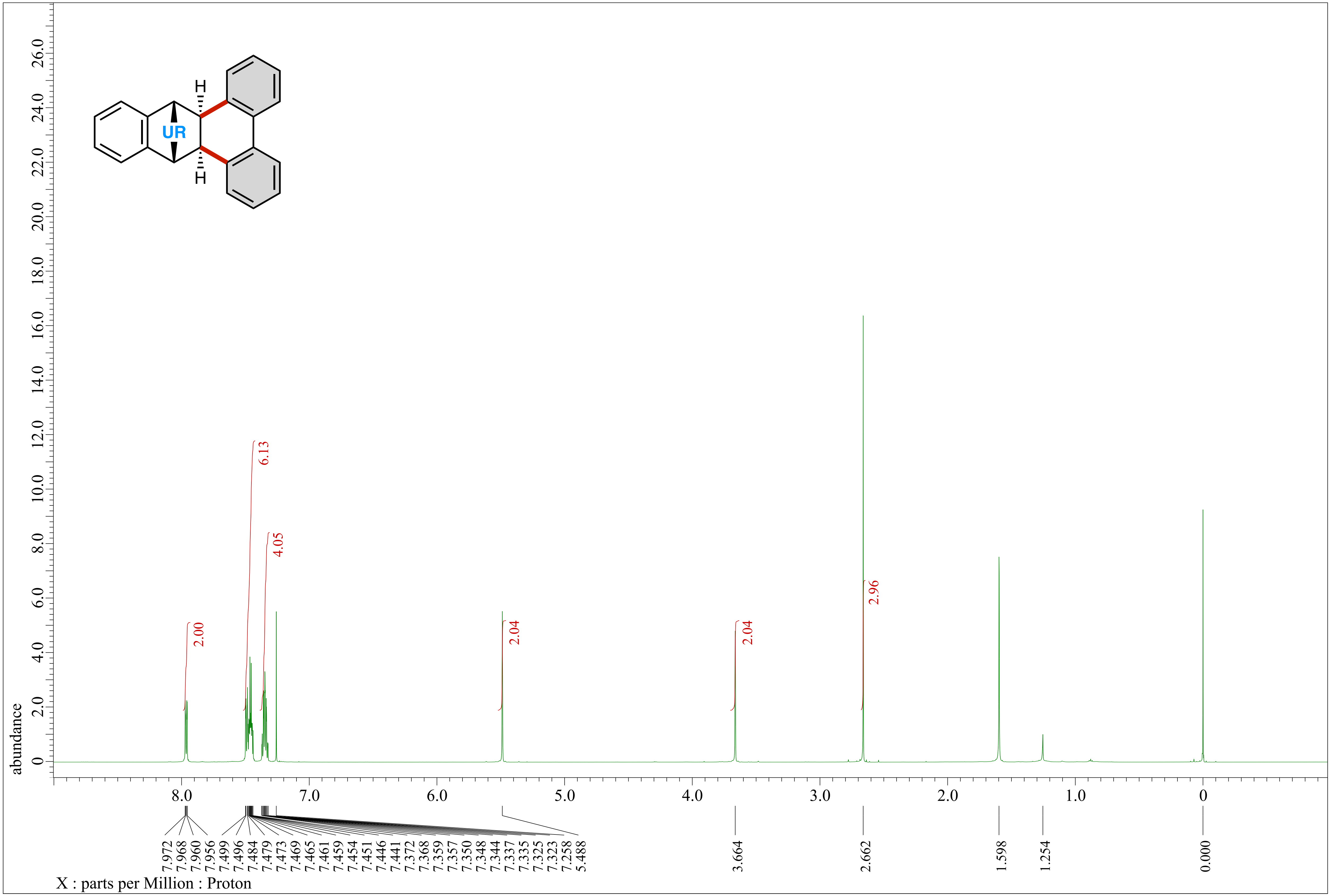

^{1}H NMR spectrum (inset) of **4ga** (solvent: $CDCl_3$)

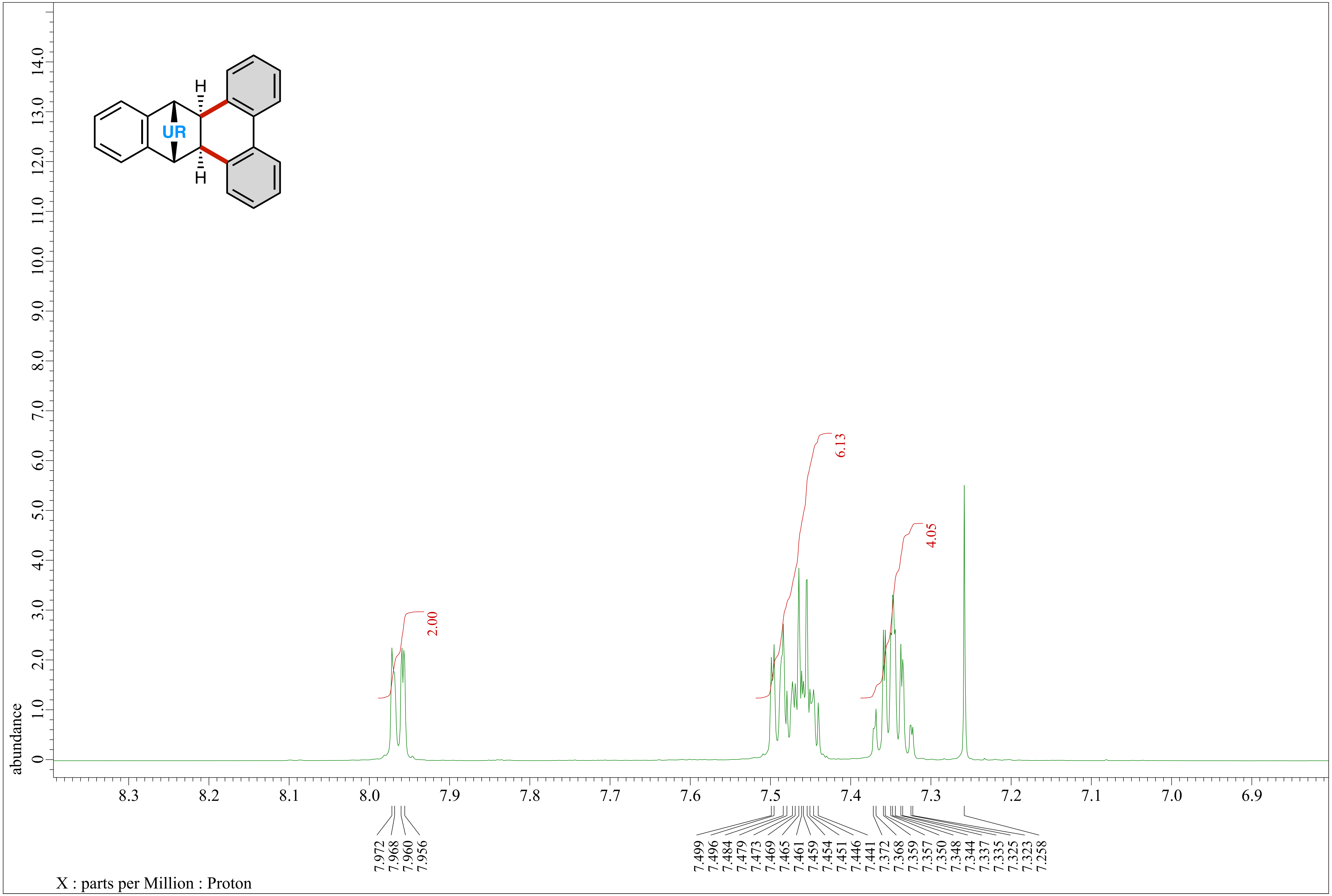

^{13}C NMR spectrum of **4ga** (solvent: $CDCl_3$)

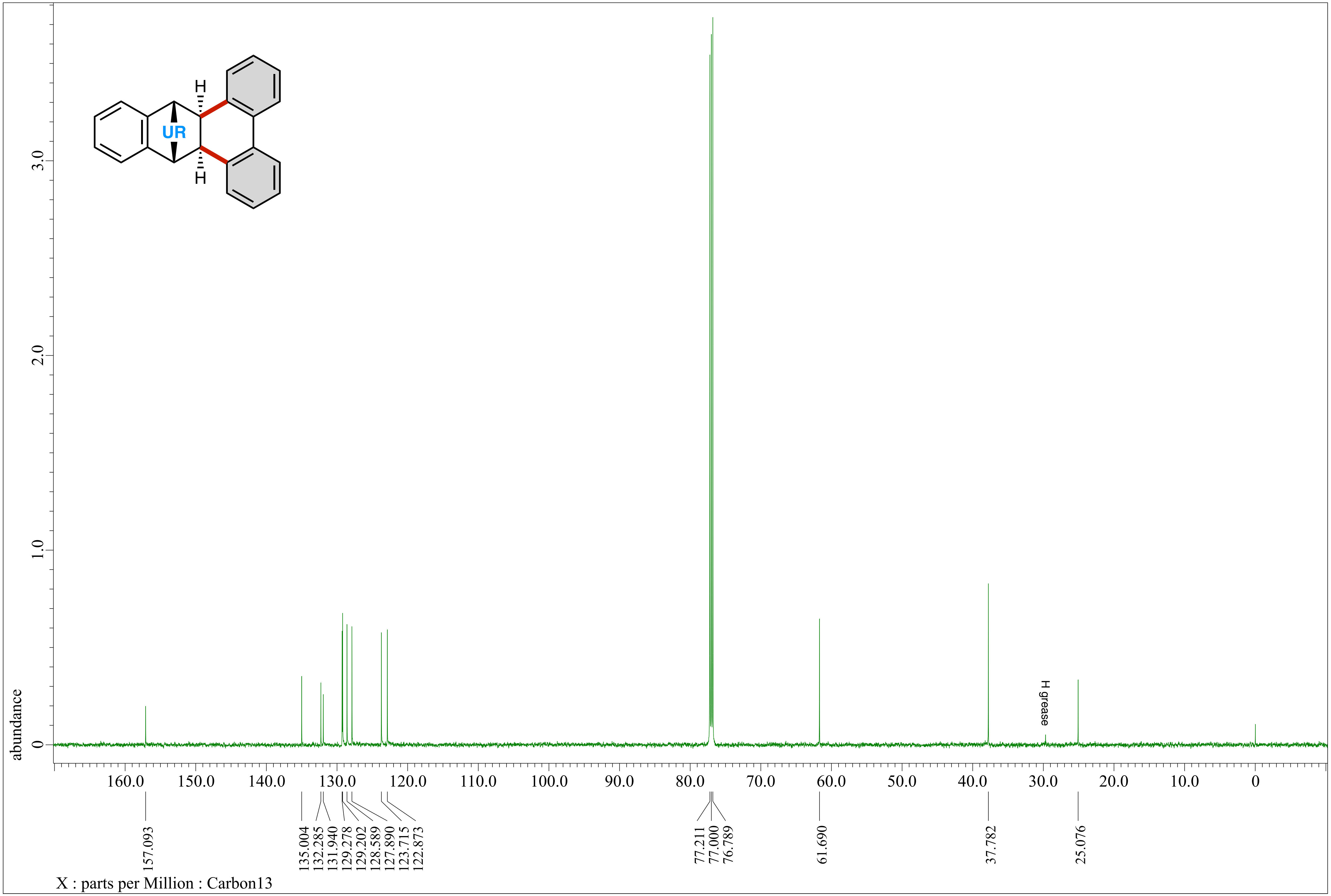

^{13}C NMR spectrum (inset) of **4ga** (solvent: $CDCl_3$)

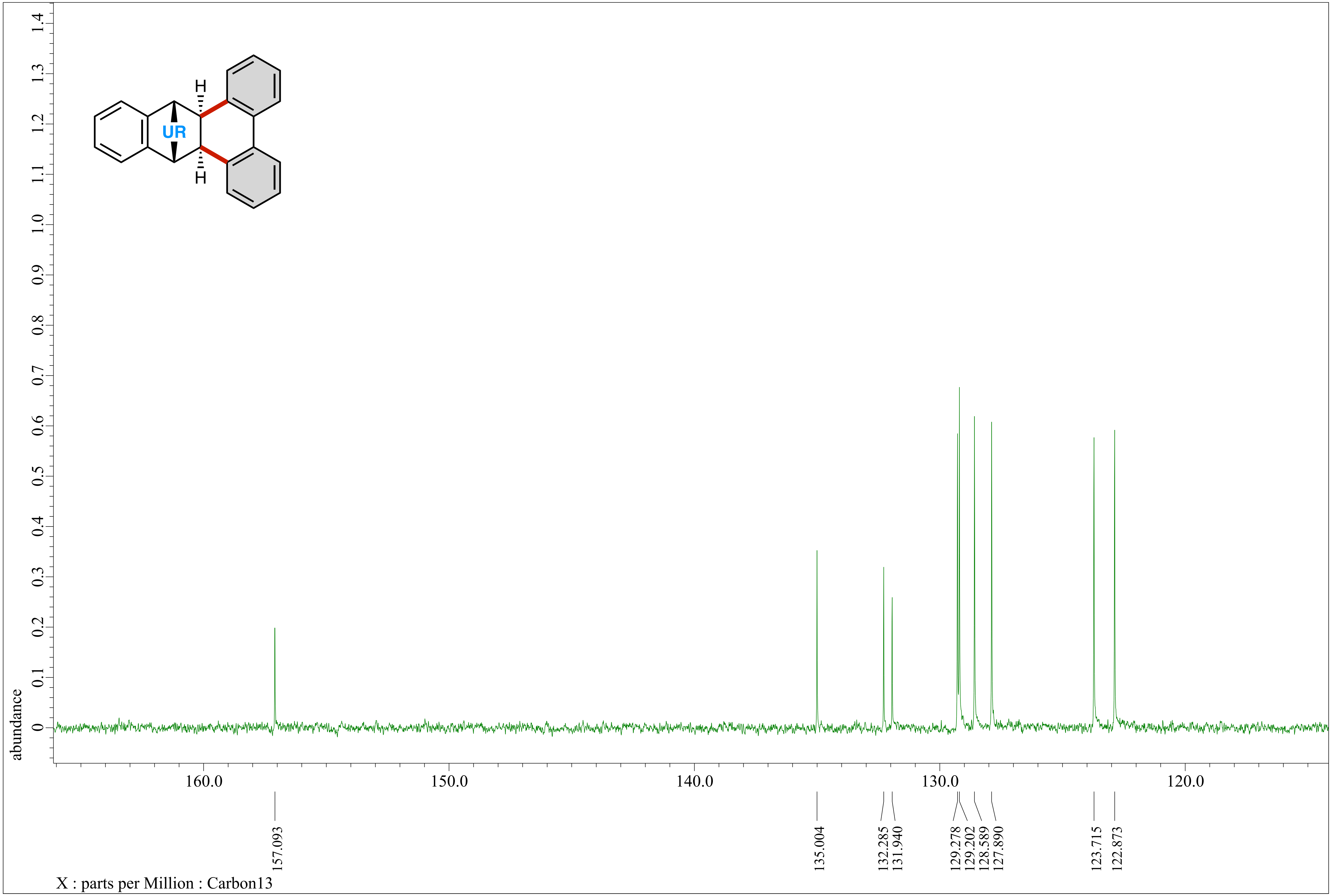

^{1}H NMR spectrum of **4ha** (solvent: $CDCl_3$)

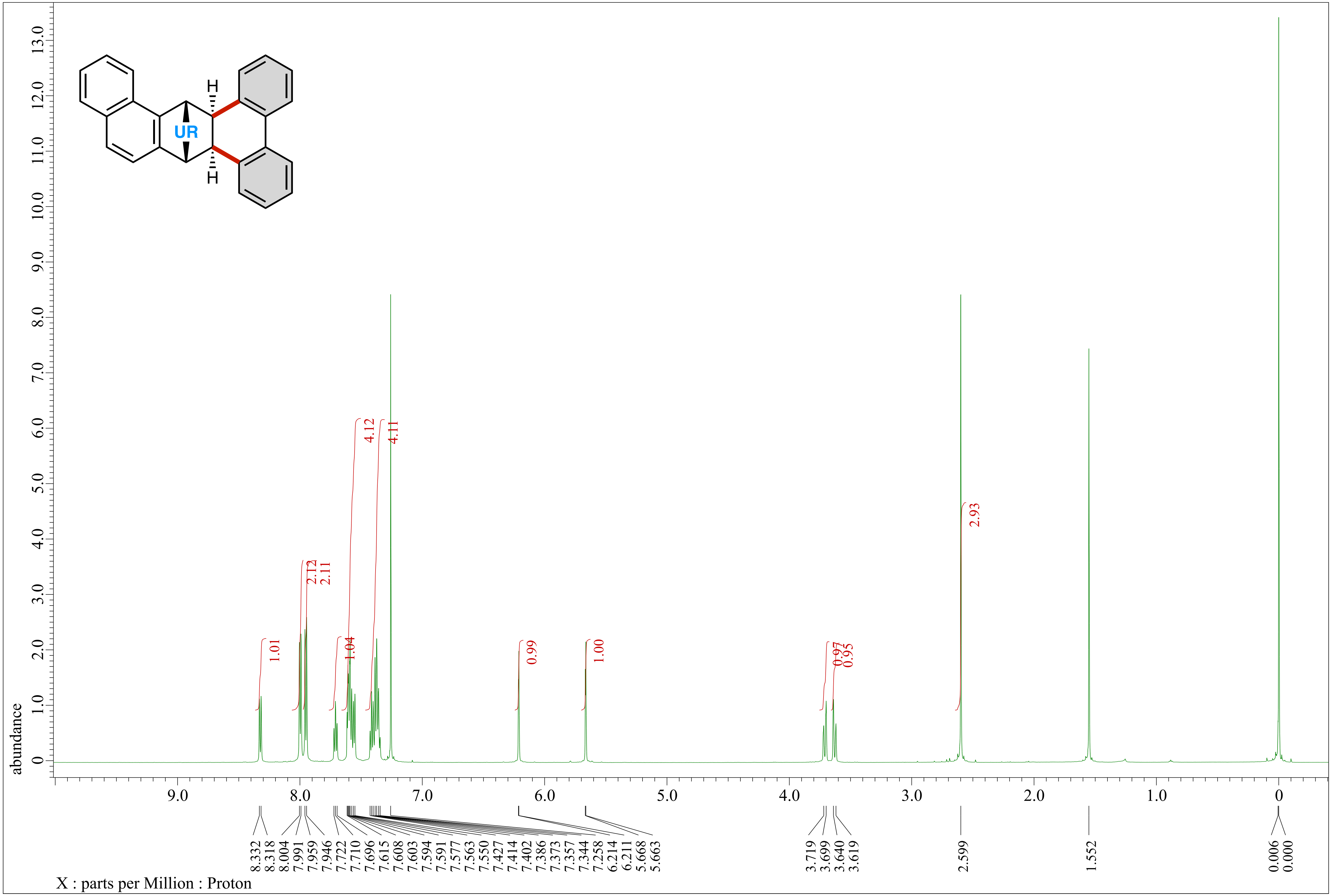

^{1}H NMR spectrum (inset) of **4ha** (solvent: $CDCl_3$)

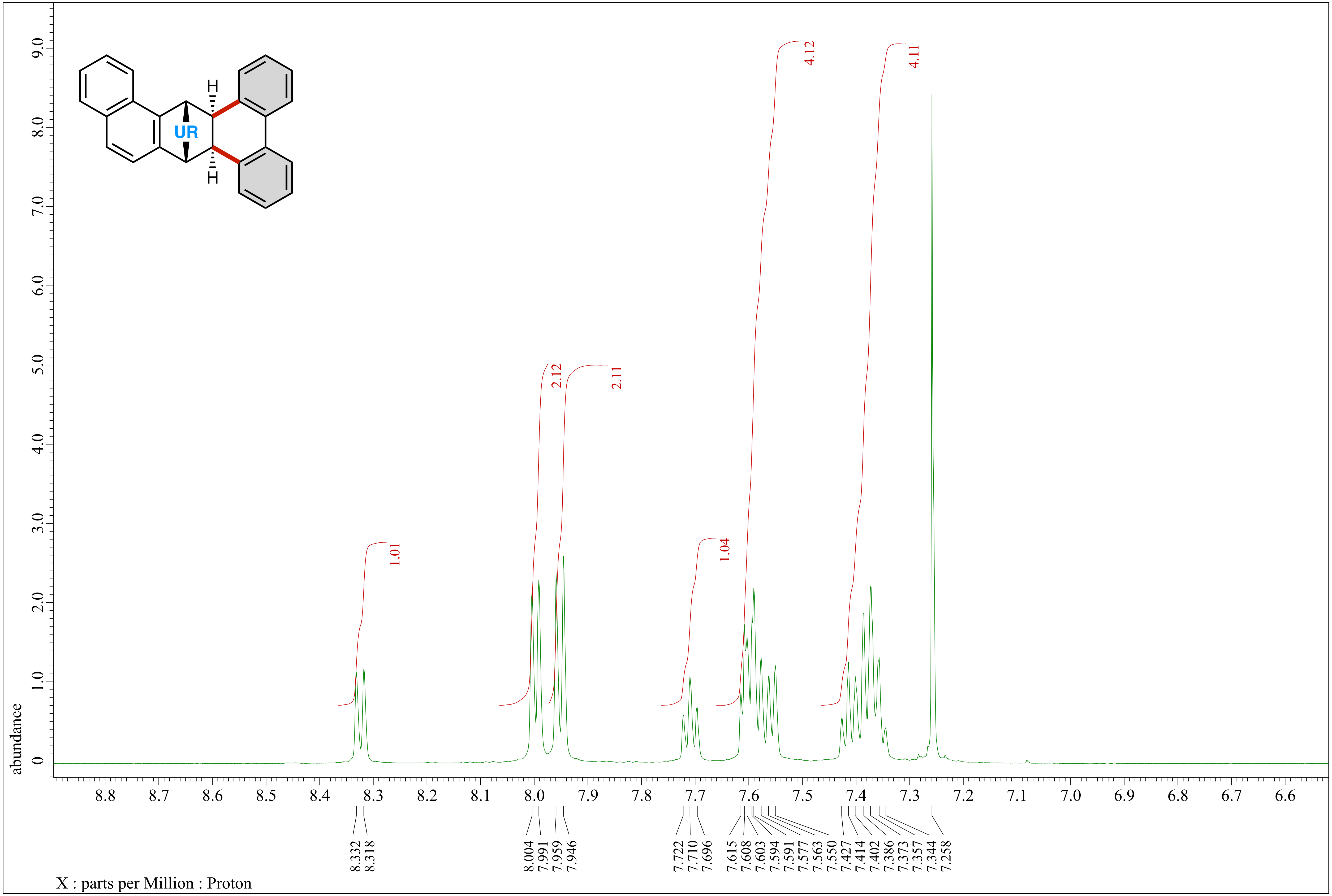

^{13}C NMR spectrum of **4ha** (solvent: $CDCl_3$)

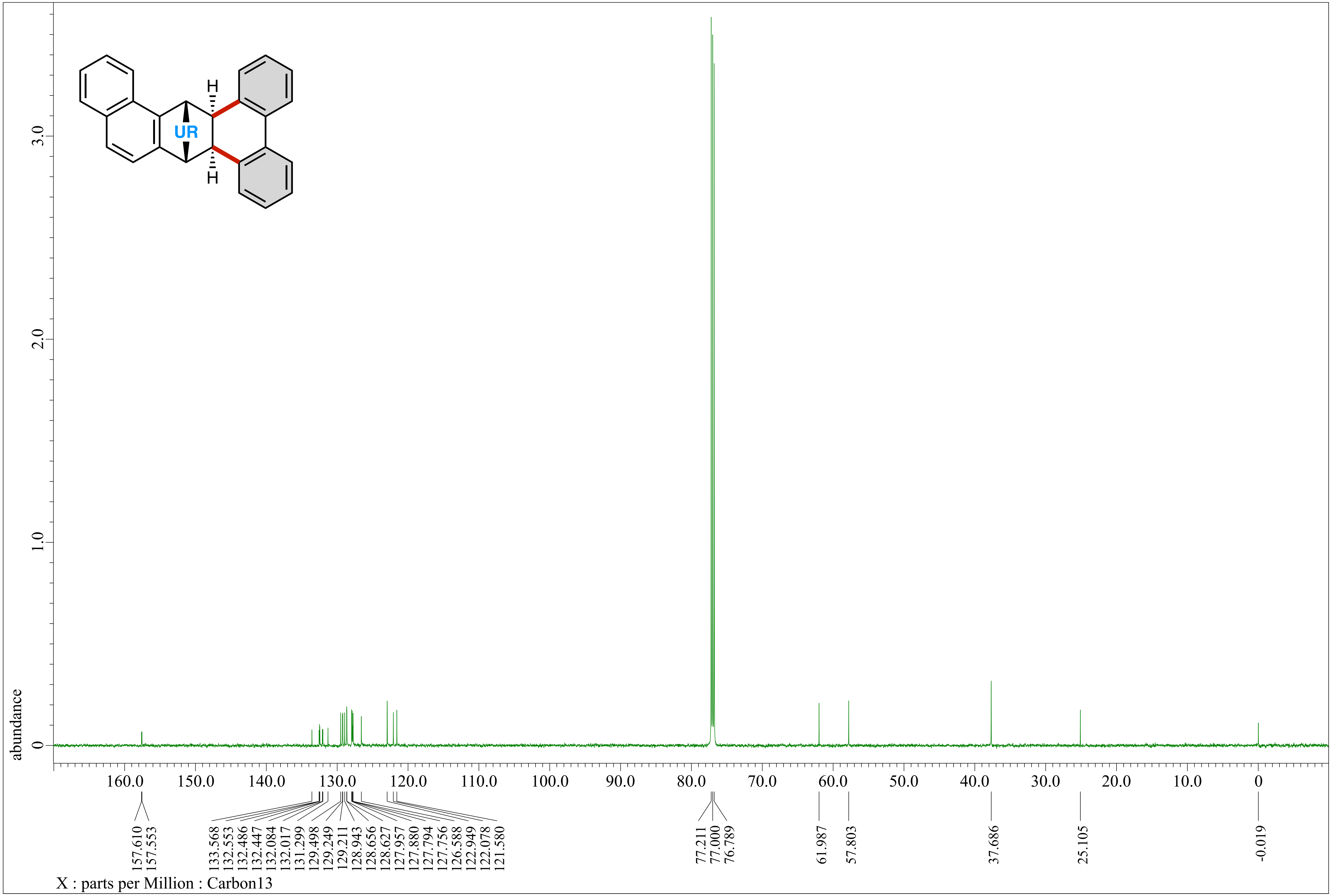

^{13}C NMR spectrum (inset) of **4ha** (solvent: $CDCl_3$)

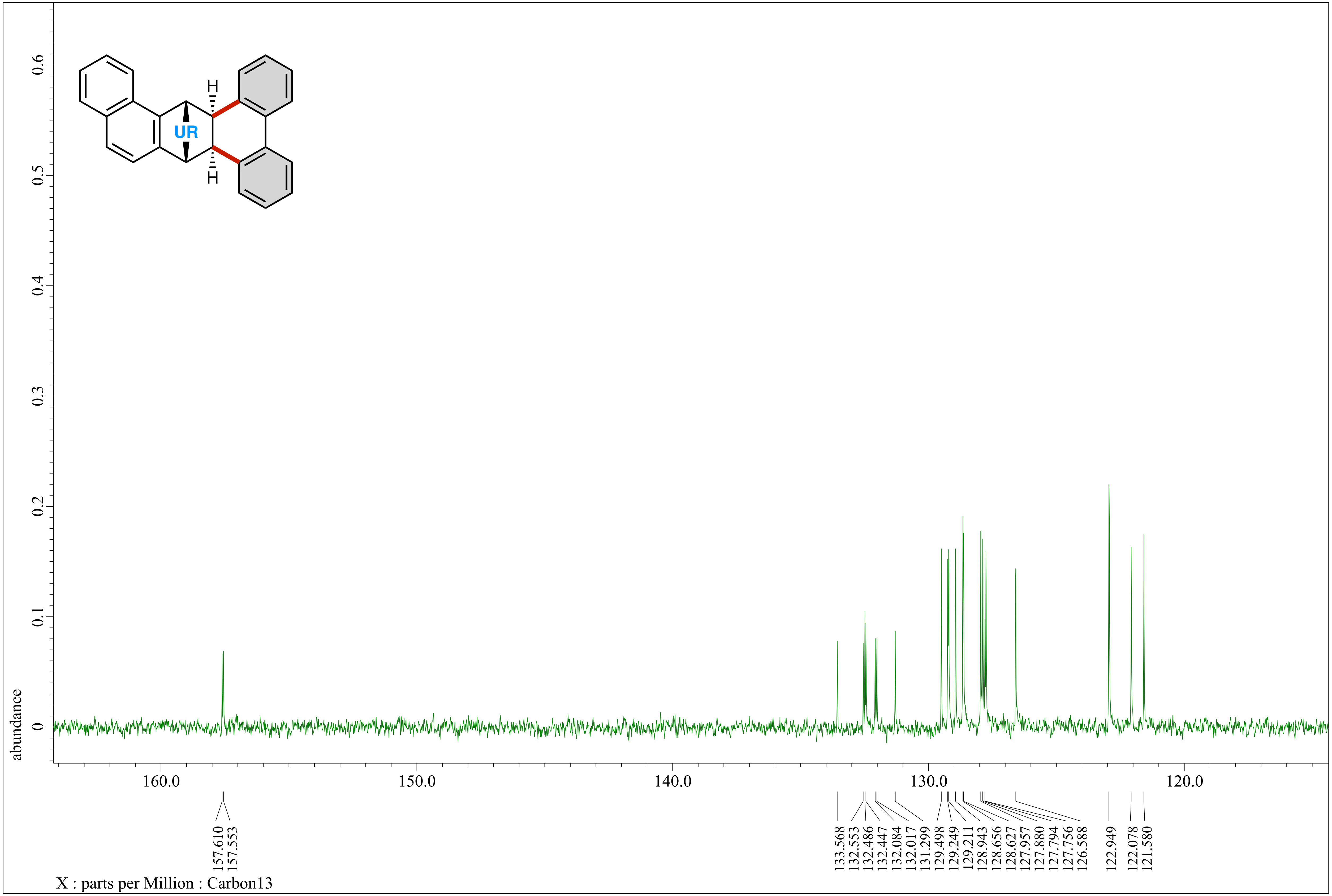

^{1}H NMR spectrum of **4ia** (solvent: $CDCl_3$)

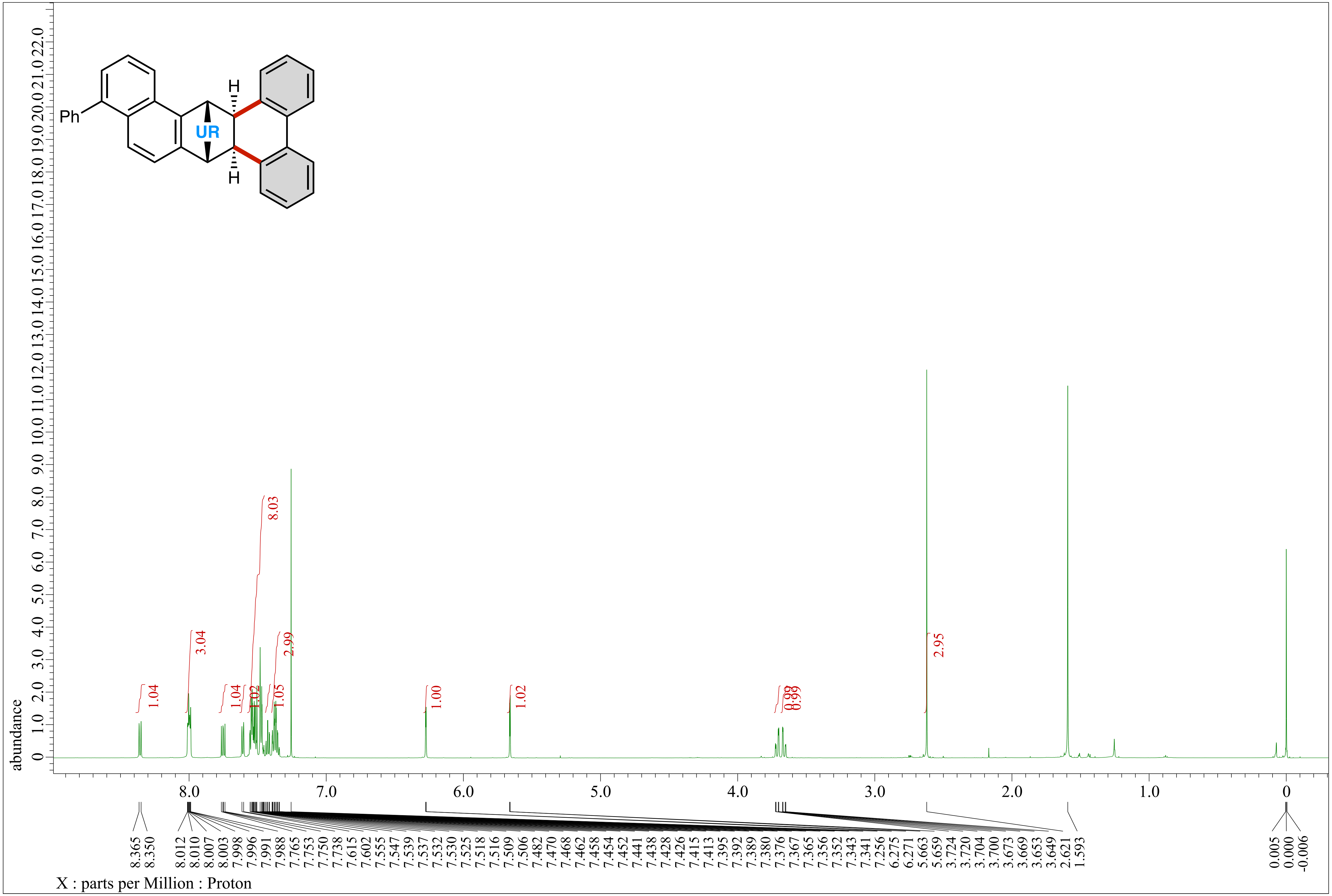

^{1}H NMR spectrum (inset) of **4ia** (solvent: $CDCl_3$)

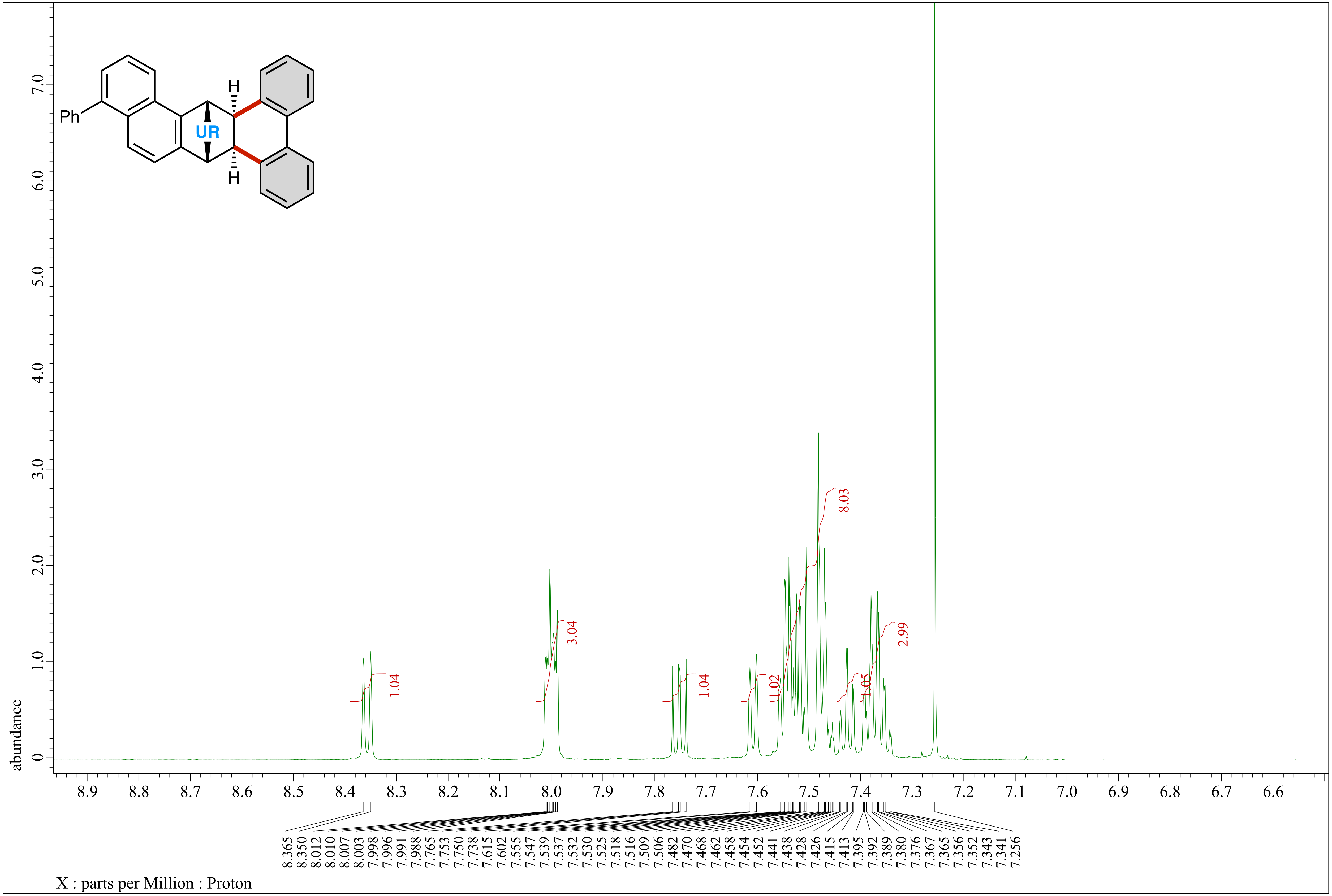

^{13}C NMR spectrum of **4ia** (solvent: $CDCl_3$)

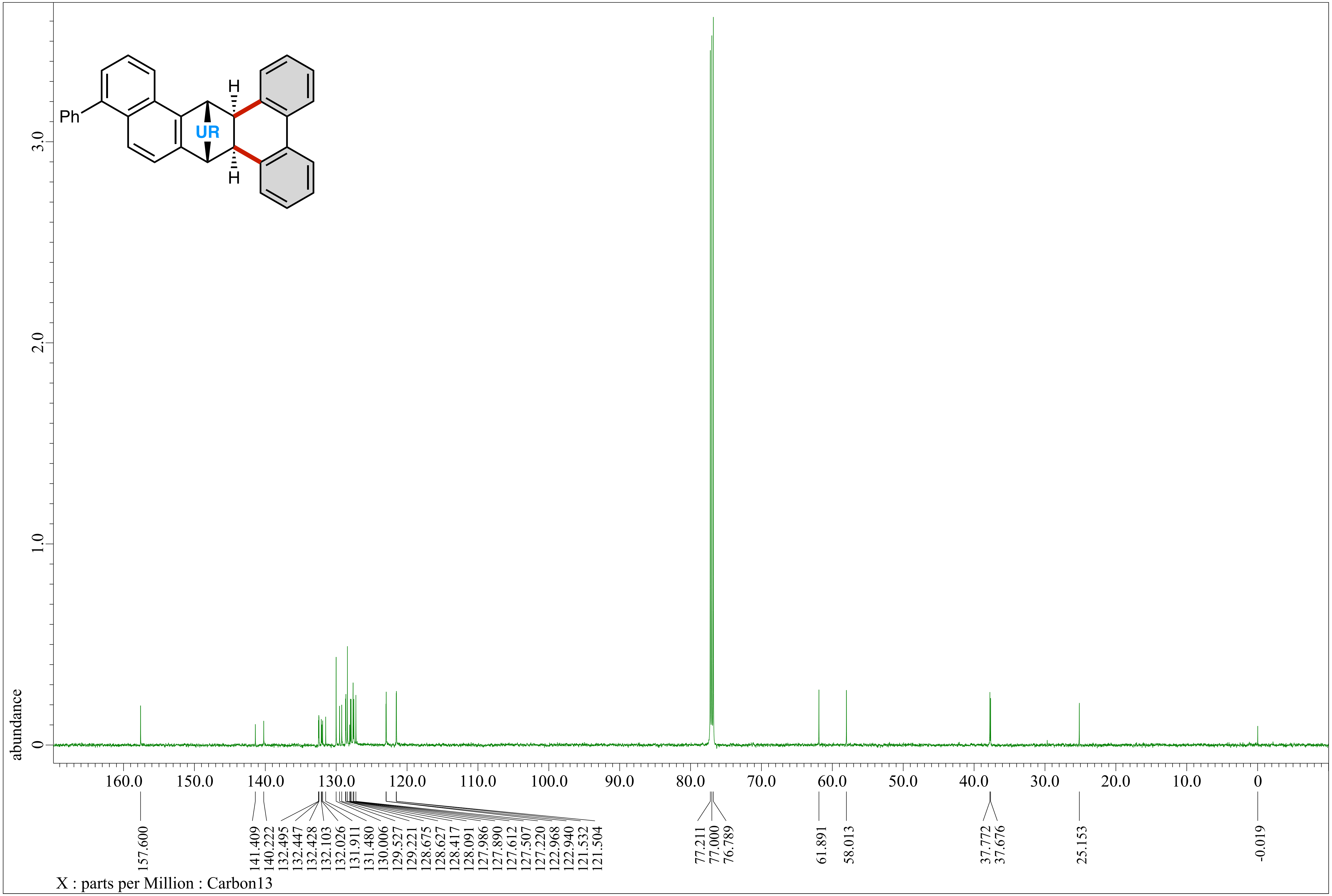

^{13}C NMR spectrum (inset) of **4ia** (solvent: $CDCl_3$)

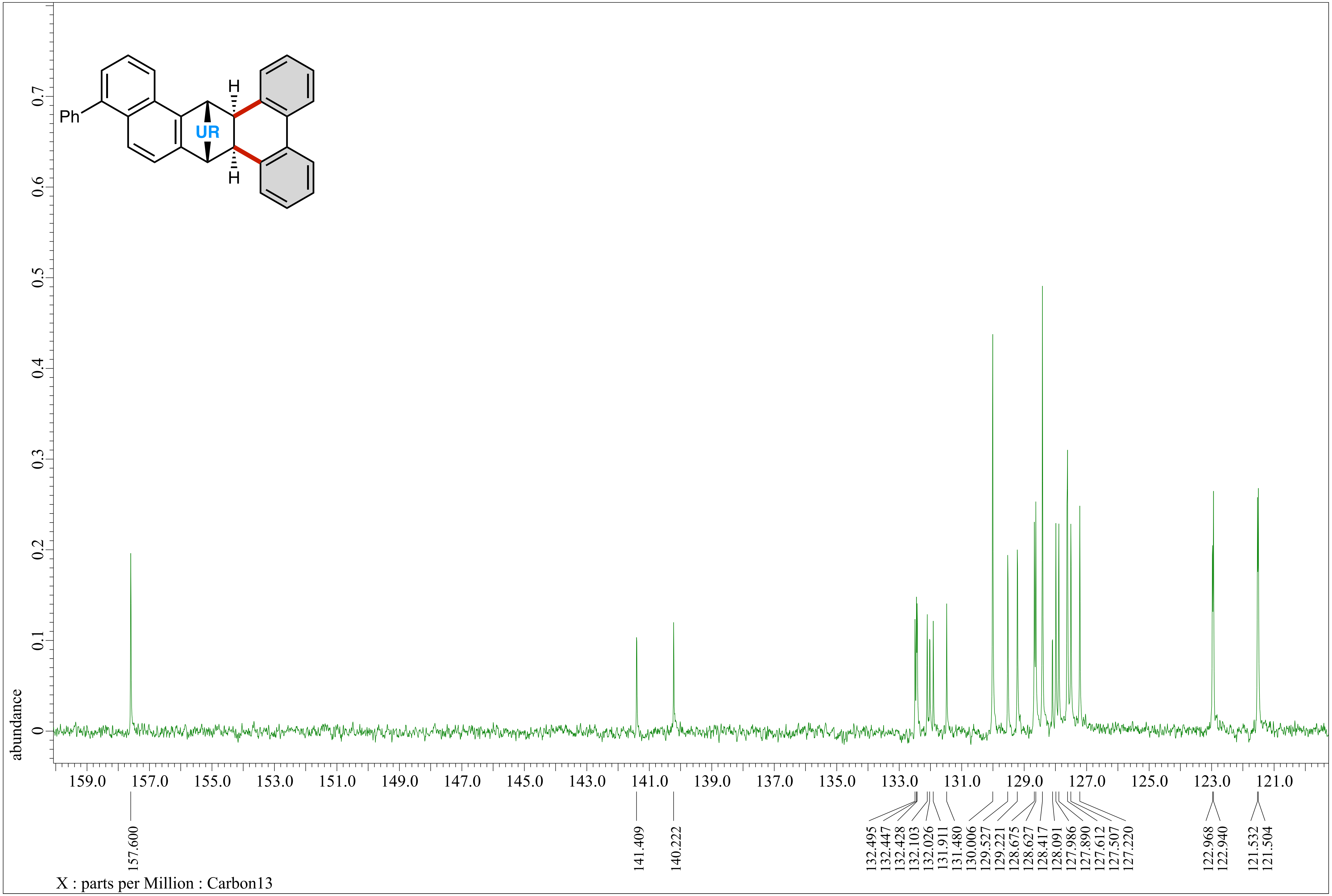

^{1}H NMR spectrum of **4ja** (solvent: $CDCl_3$)

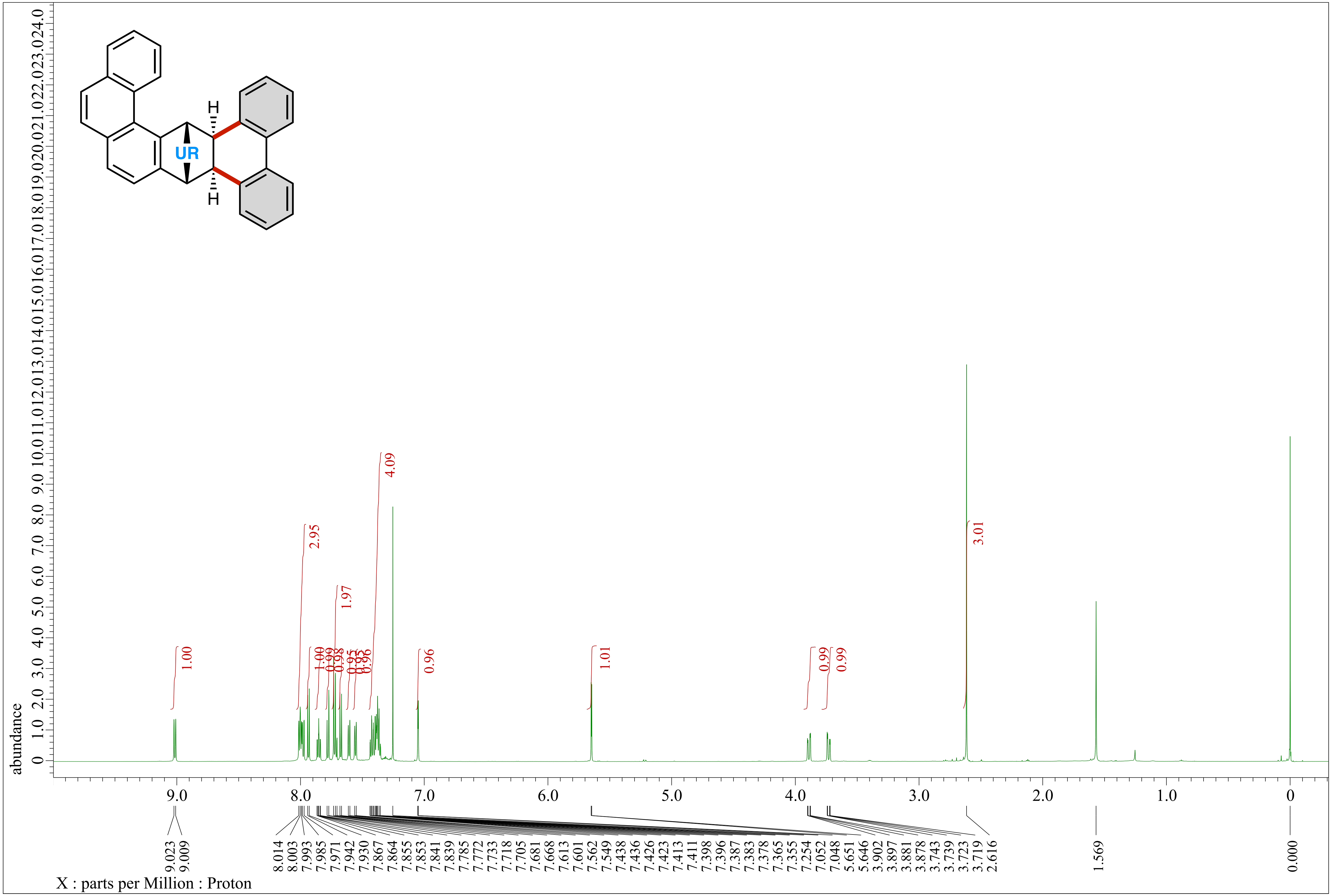

^{1}H NMR spectrum (inset) of **4ja** (solvent: $CDCl_3$)

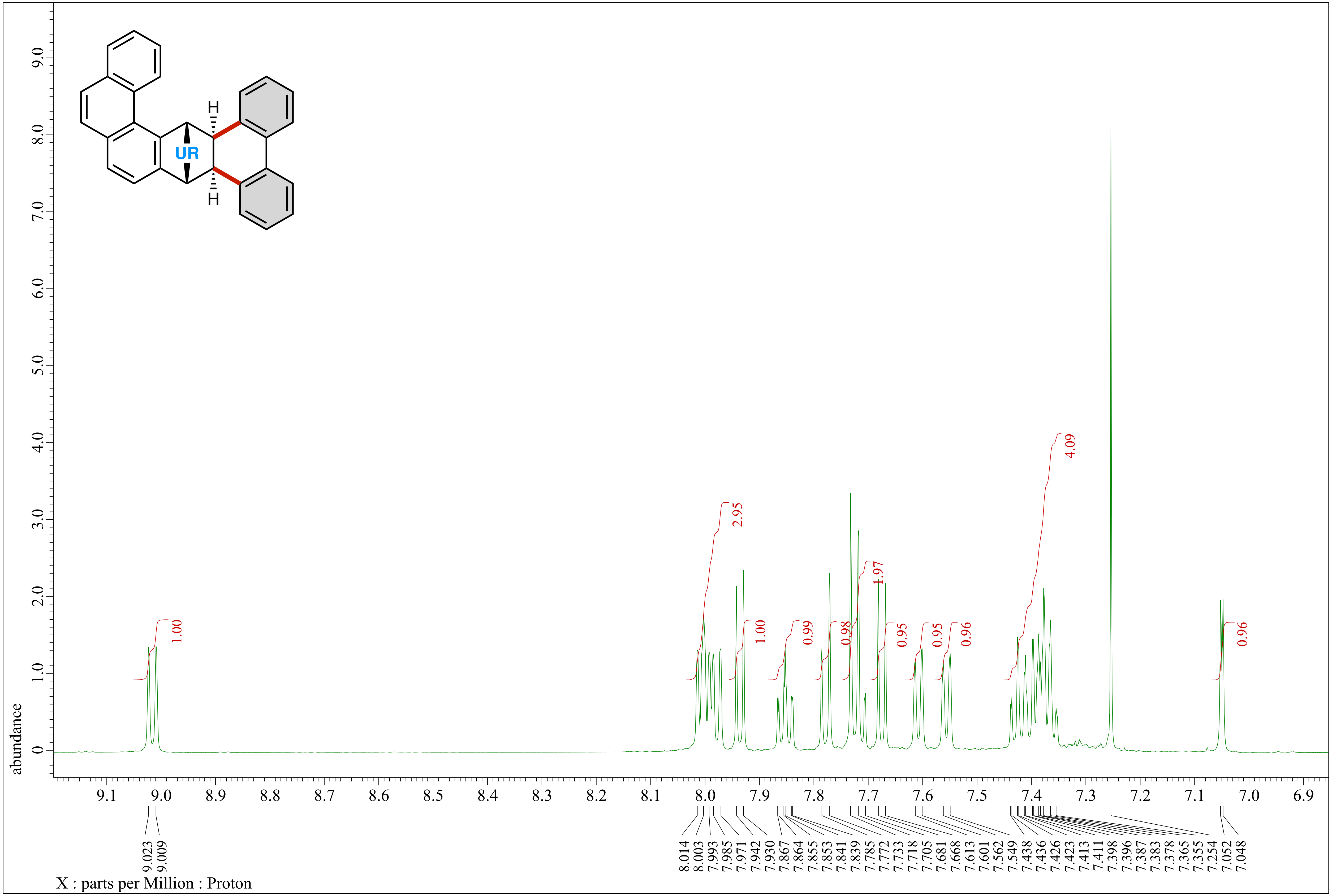

^{13}C NMR spectrum of **4ja** (solvent: $CDCl_3$)

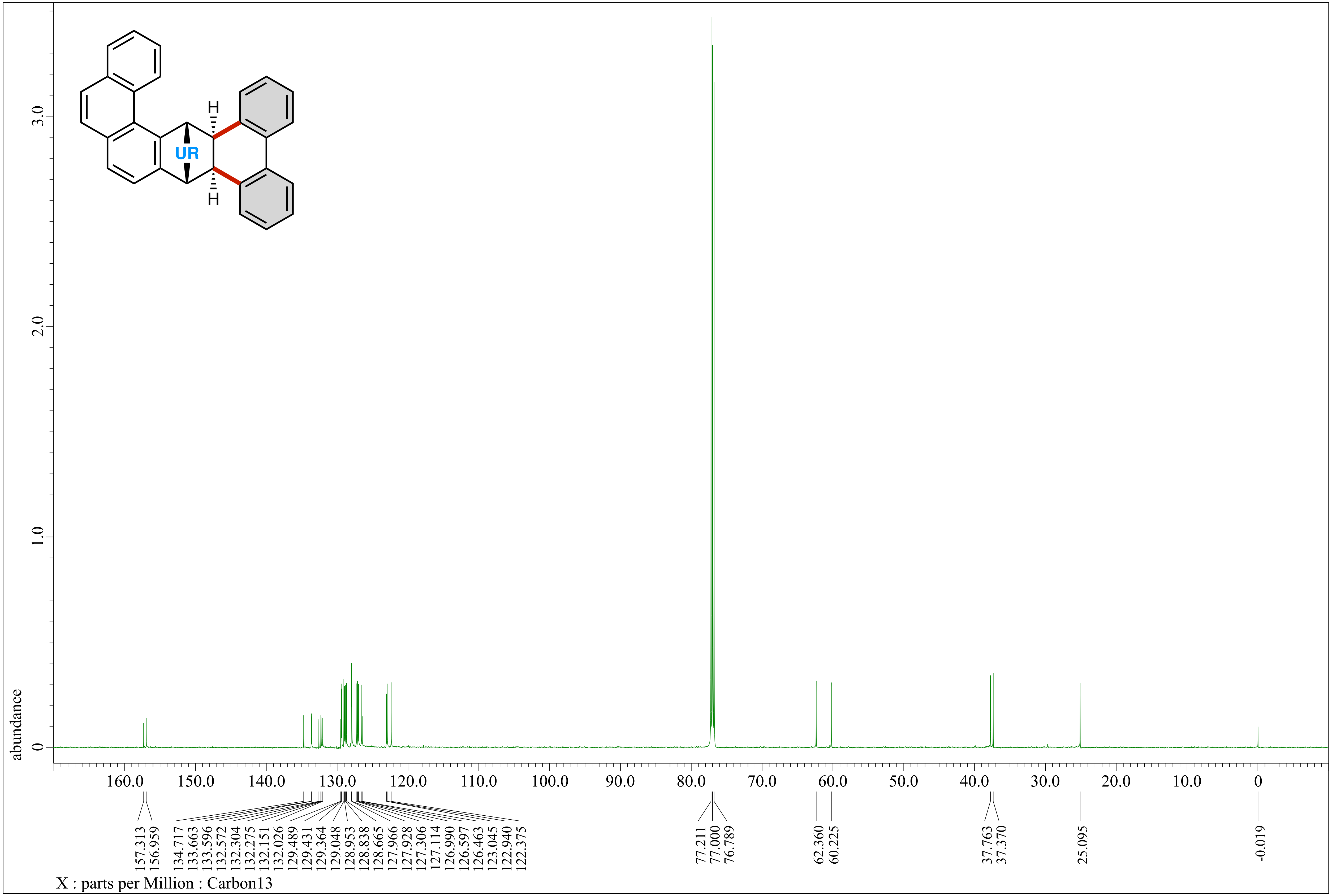

^{13}C NMR spectrum (inset) of **4ja** (solvent: $CDCl_3$)

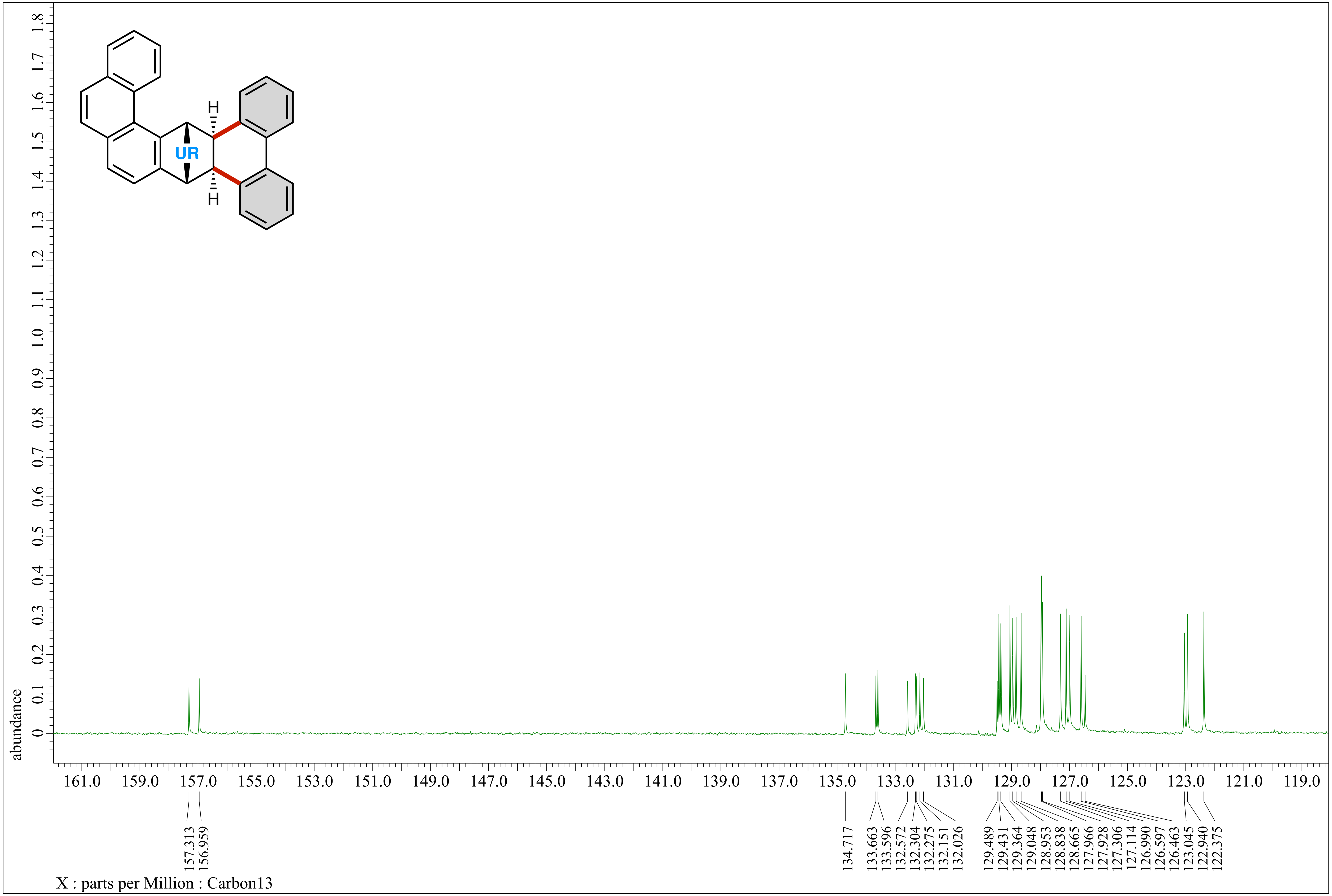

^{1}H NMR spectrum of **4ka** (solvent: $CDCl_3$)

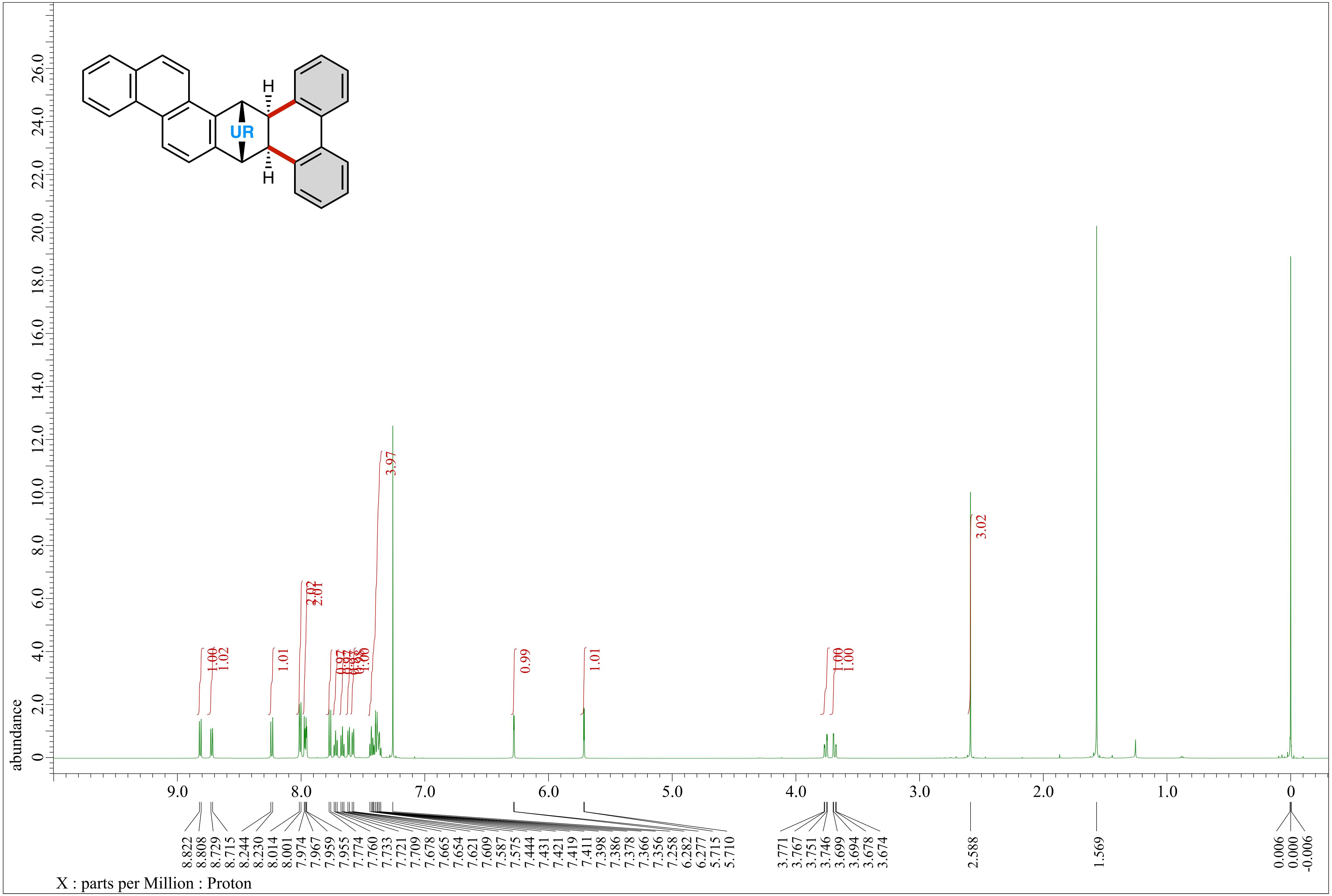

^{1}H NMR spectrum (inset) of **4ka** (solvent: $CDCl_3$)

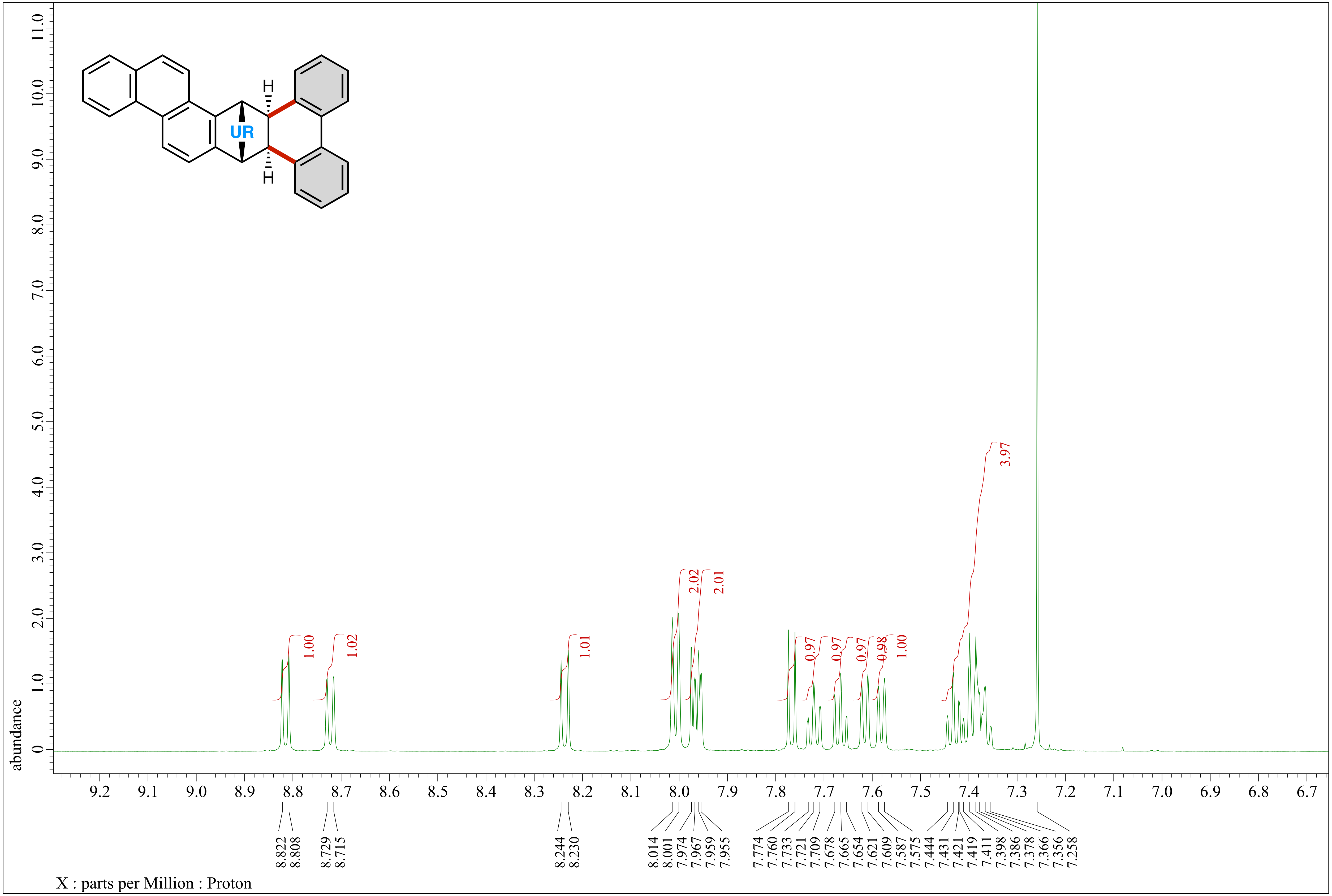

^{13}C NMR spectrum of **4ka** (solvent: $CDCl_3$)

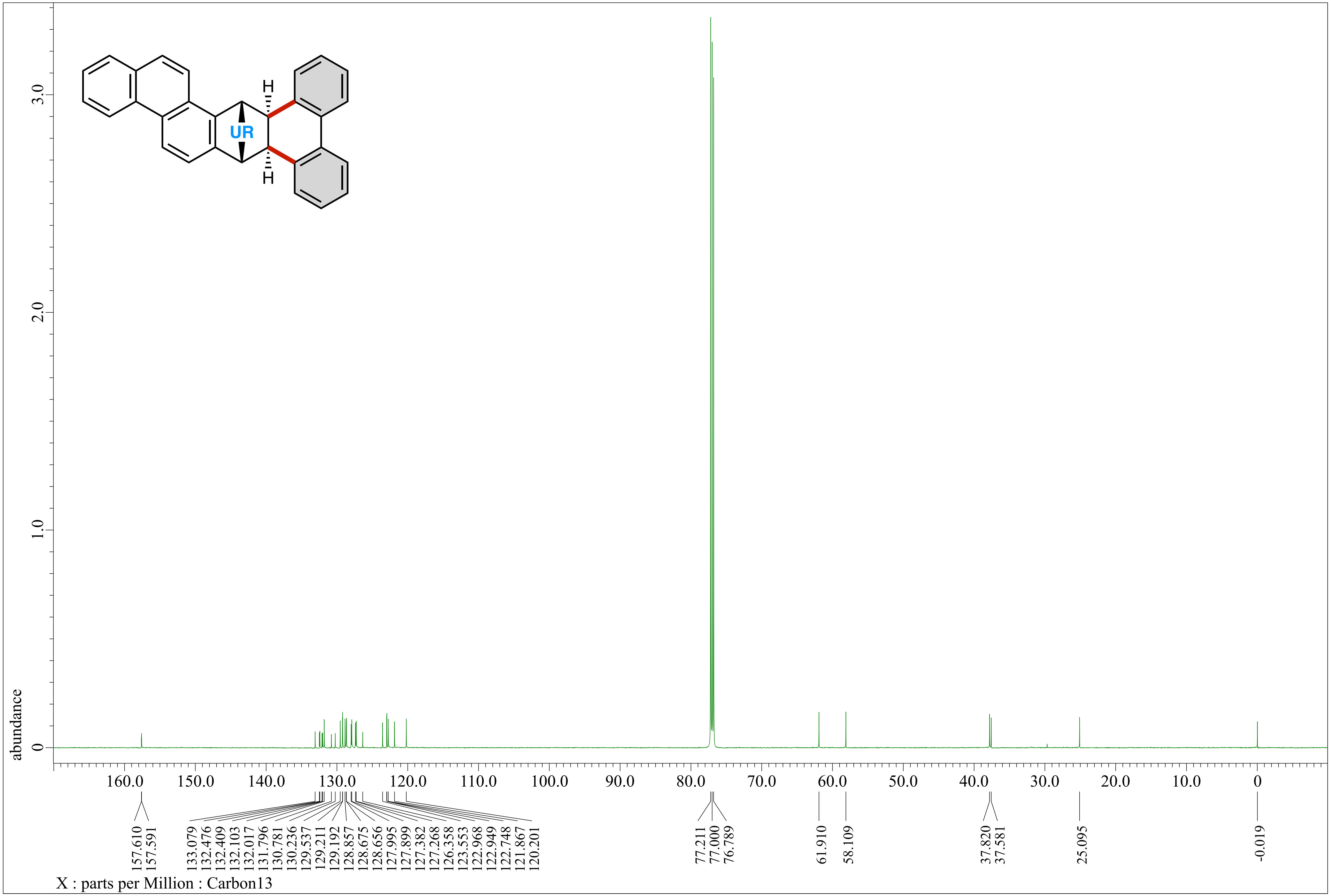

^{13}C NMR spectrum (inset) of **4ka** (solvent: $CDCl_3$)

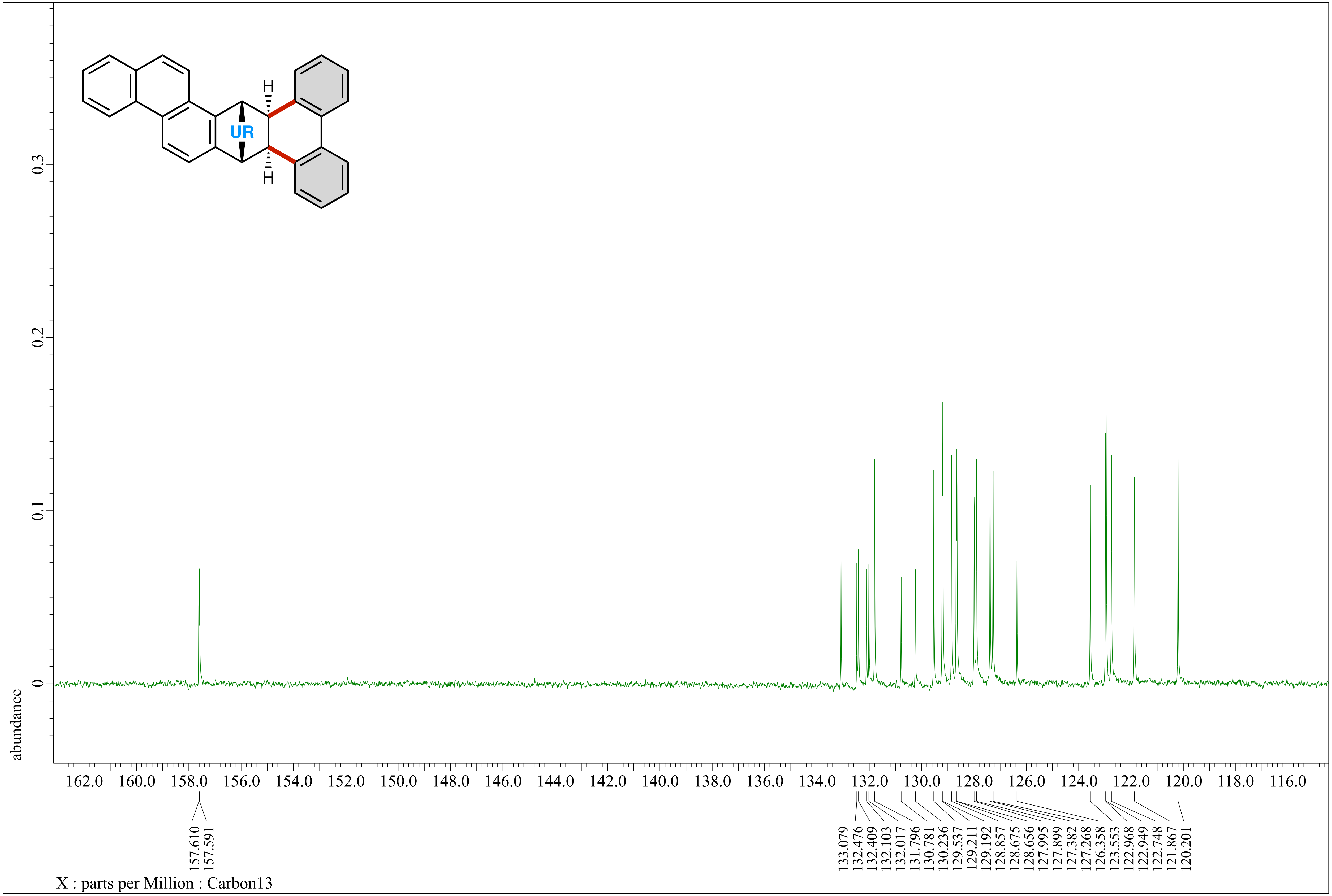

^{1}H NMR spectrum of **4la** (solvent: CD_2Cl_2)

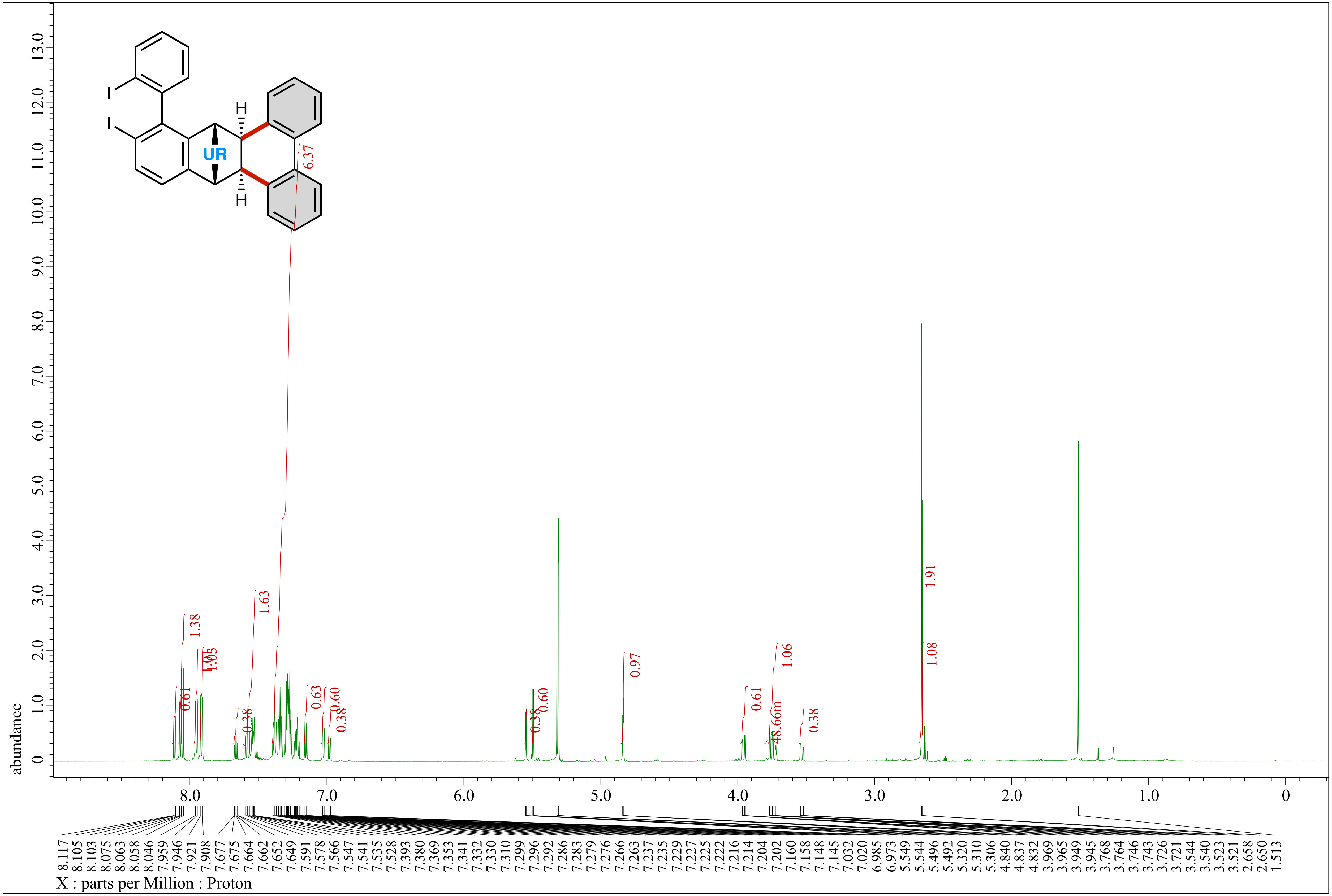

^{1}H NMR spectrum (inset) of **4la** (solvent: CD_2Cl_2)

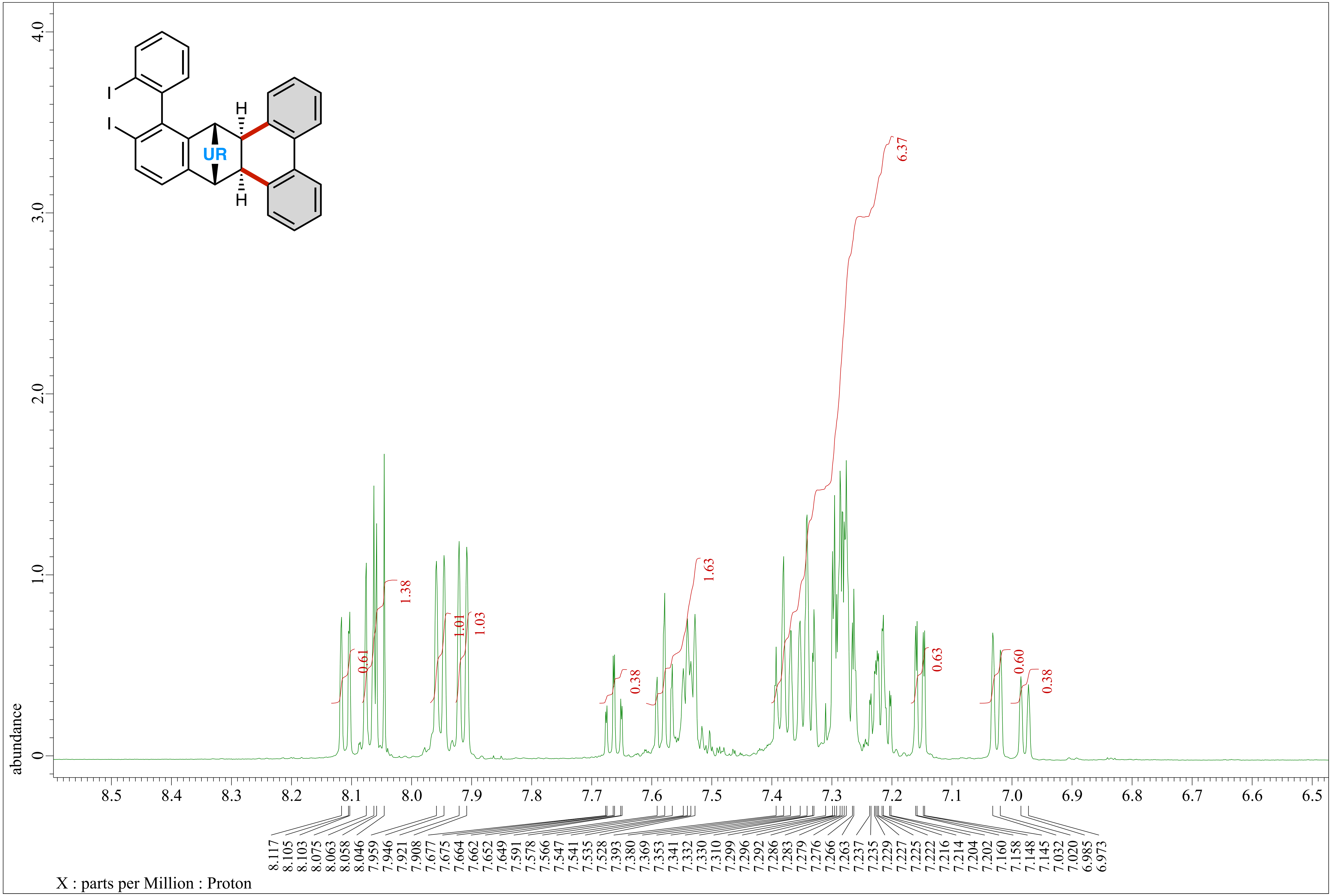

^{13}C NMR spectrum of **4la** (solvent: CD_2Cl_2)

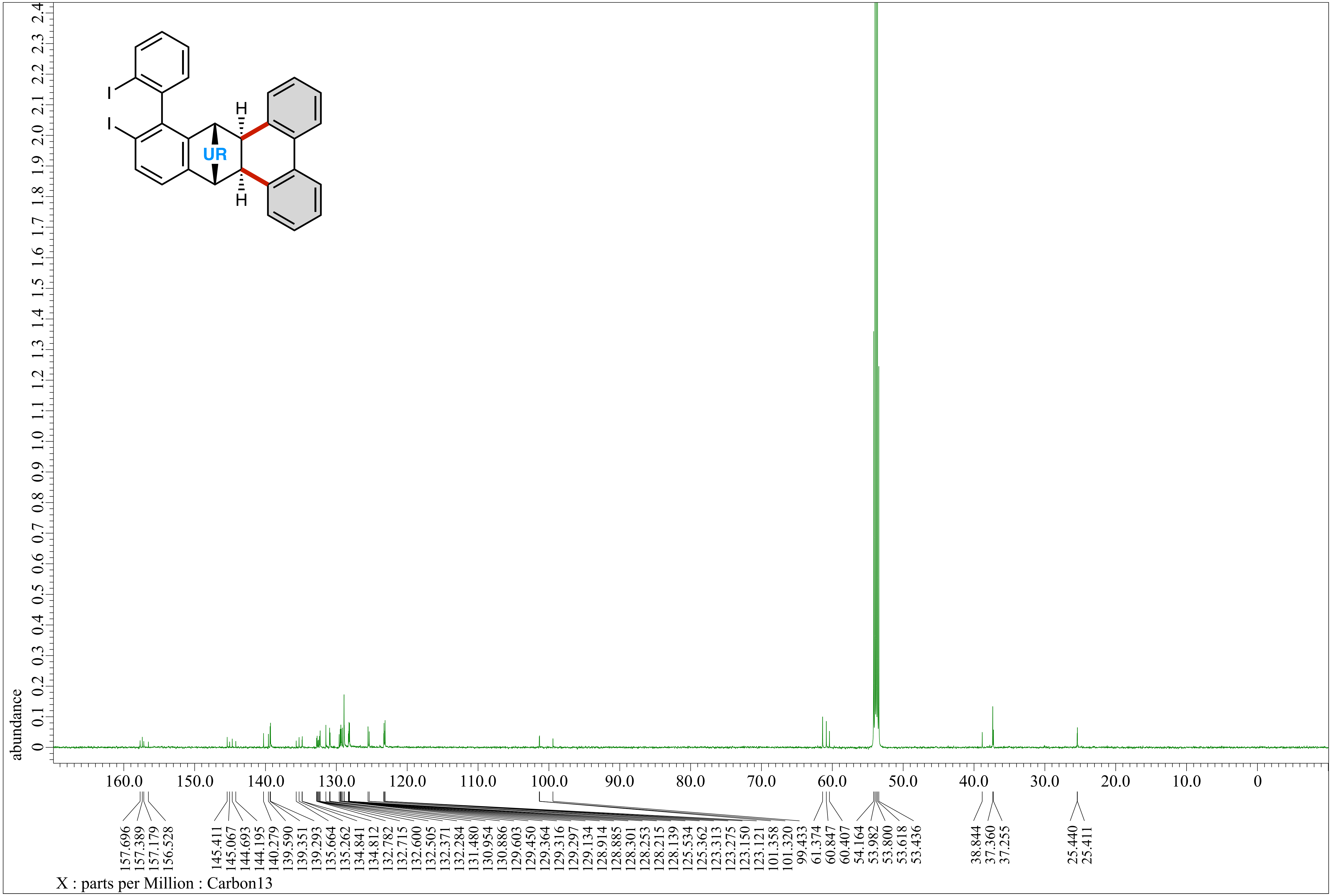

^{13}C NMR spectrum (inset) of **4la** (solvent: CD_2Cl_2)

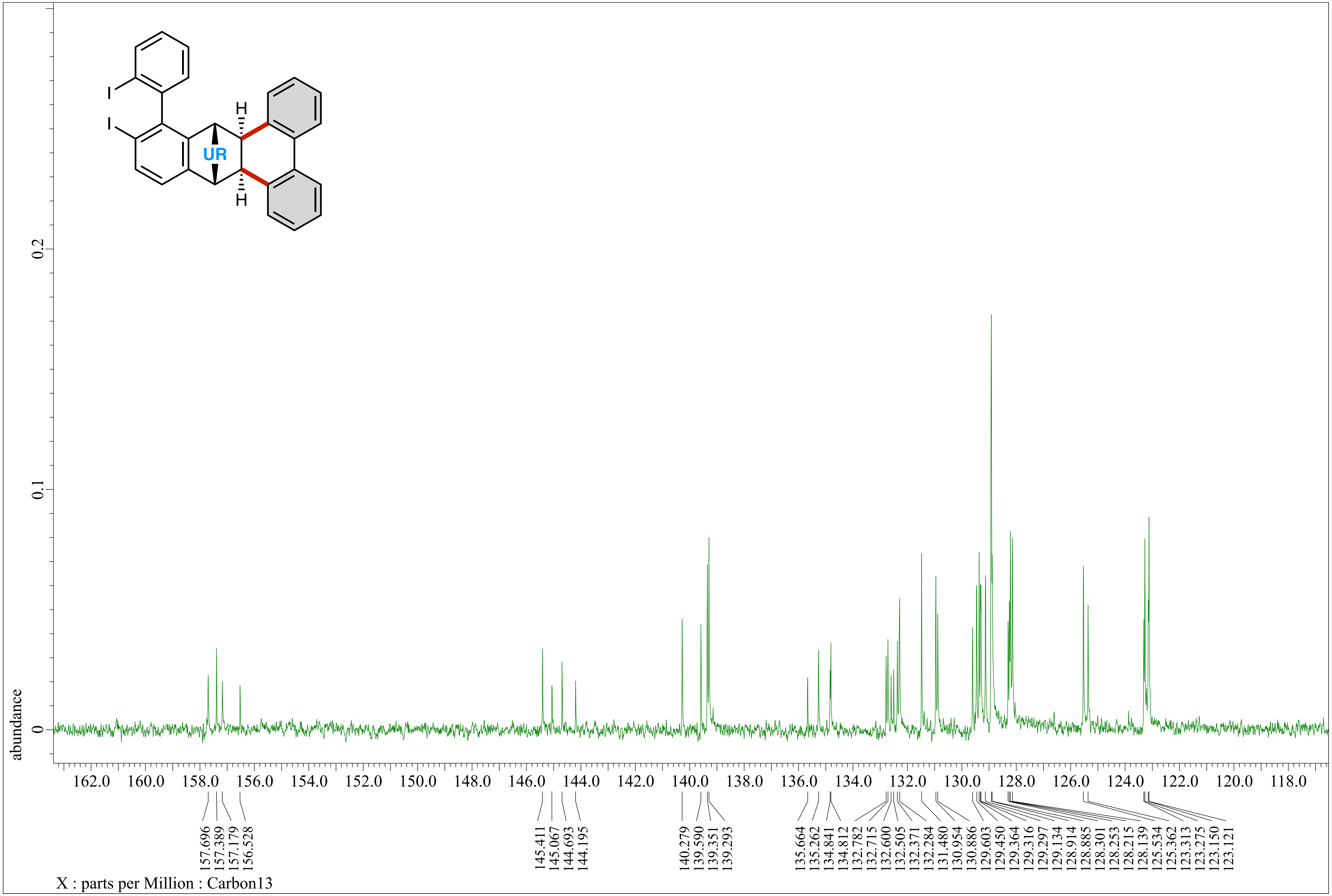

^{1}H NMR spectrum of **4ma** (solvent: $Cl_2CDCDCl_2$)

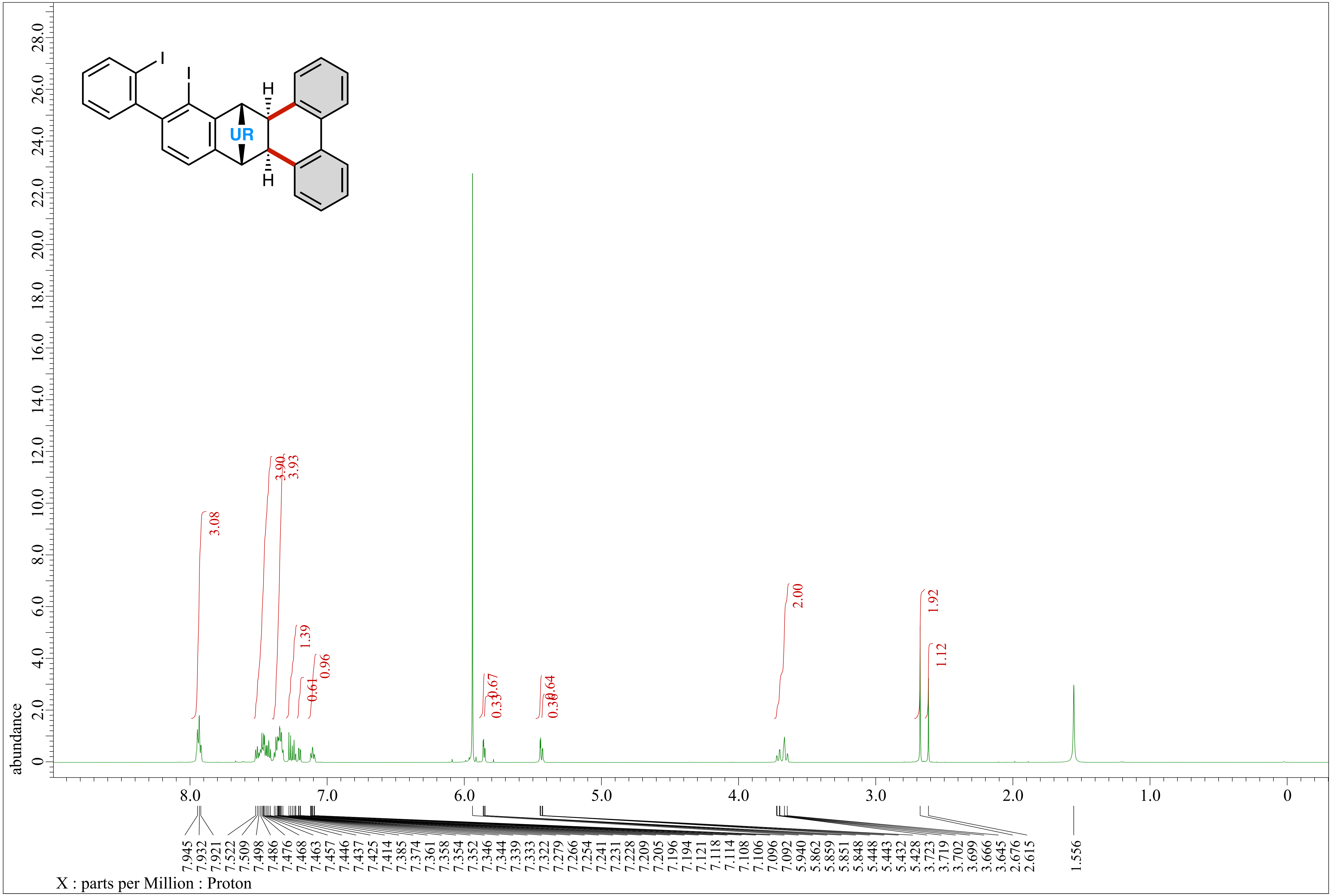

^{1}H NMR spectrum (inset) of **4ma** (solvent: $Cl_2CDCDCl_2$)

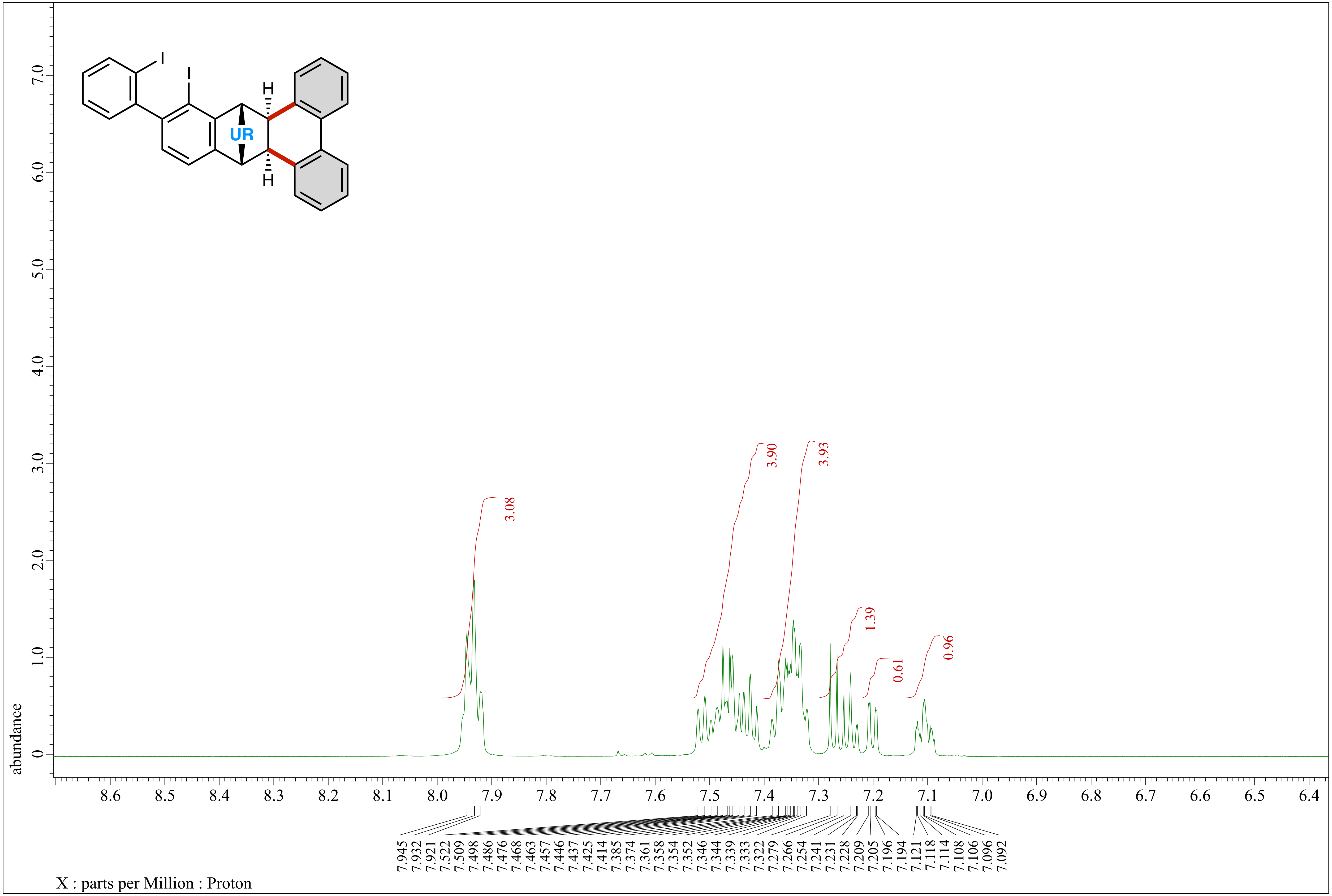

^{13}C NMR spectrum of **4ma** (solvent: $Cl_2CDCDCl_2$)

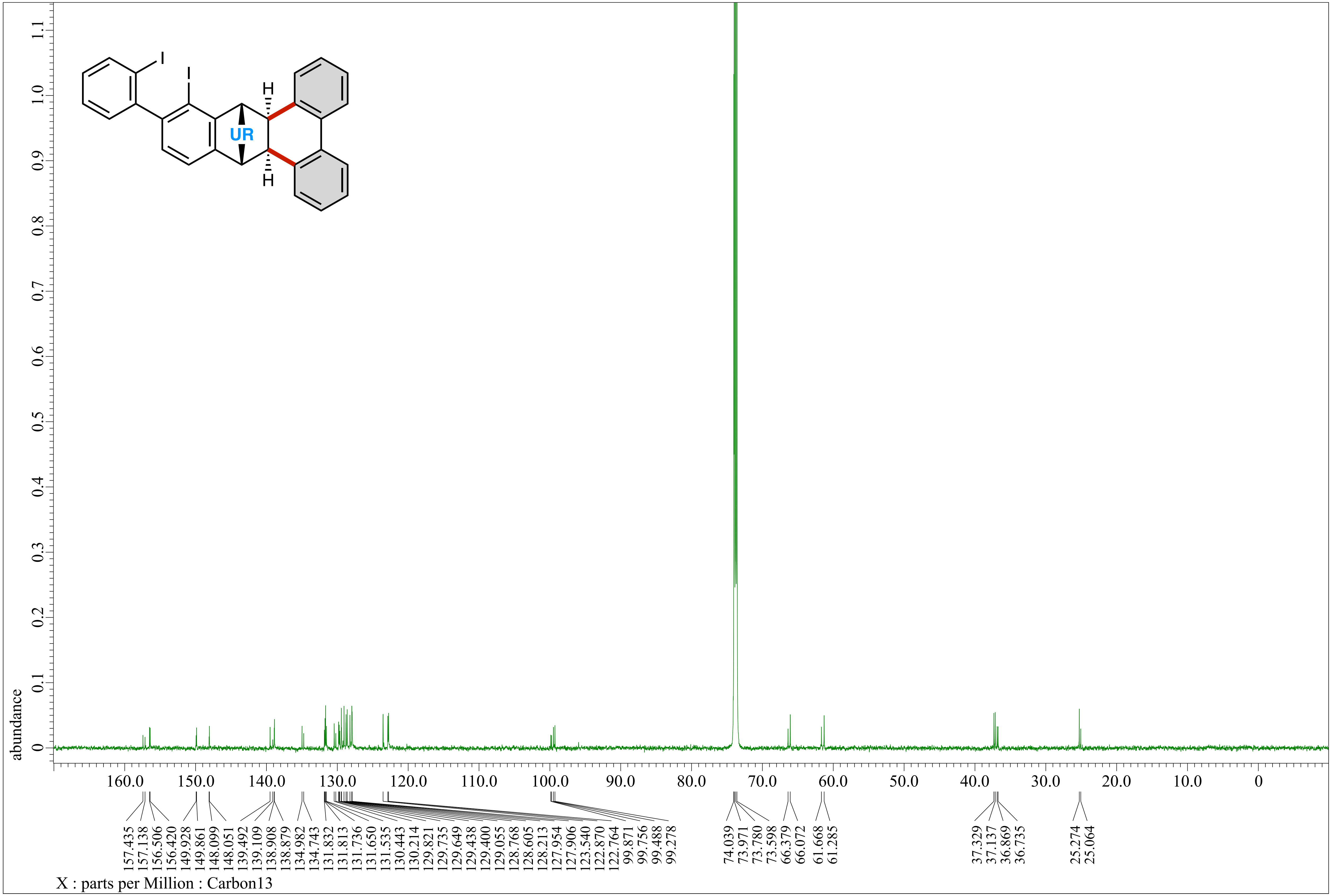

^{13}C NMR spectrum (inset) of **4ma** (solvent: $Cl_2CDCDCl_2$)

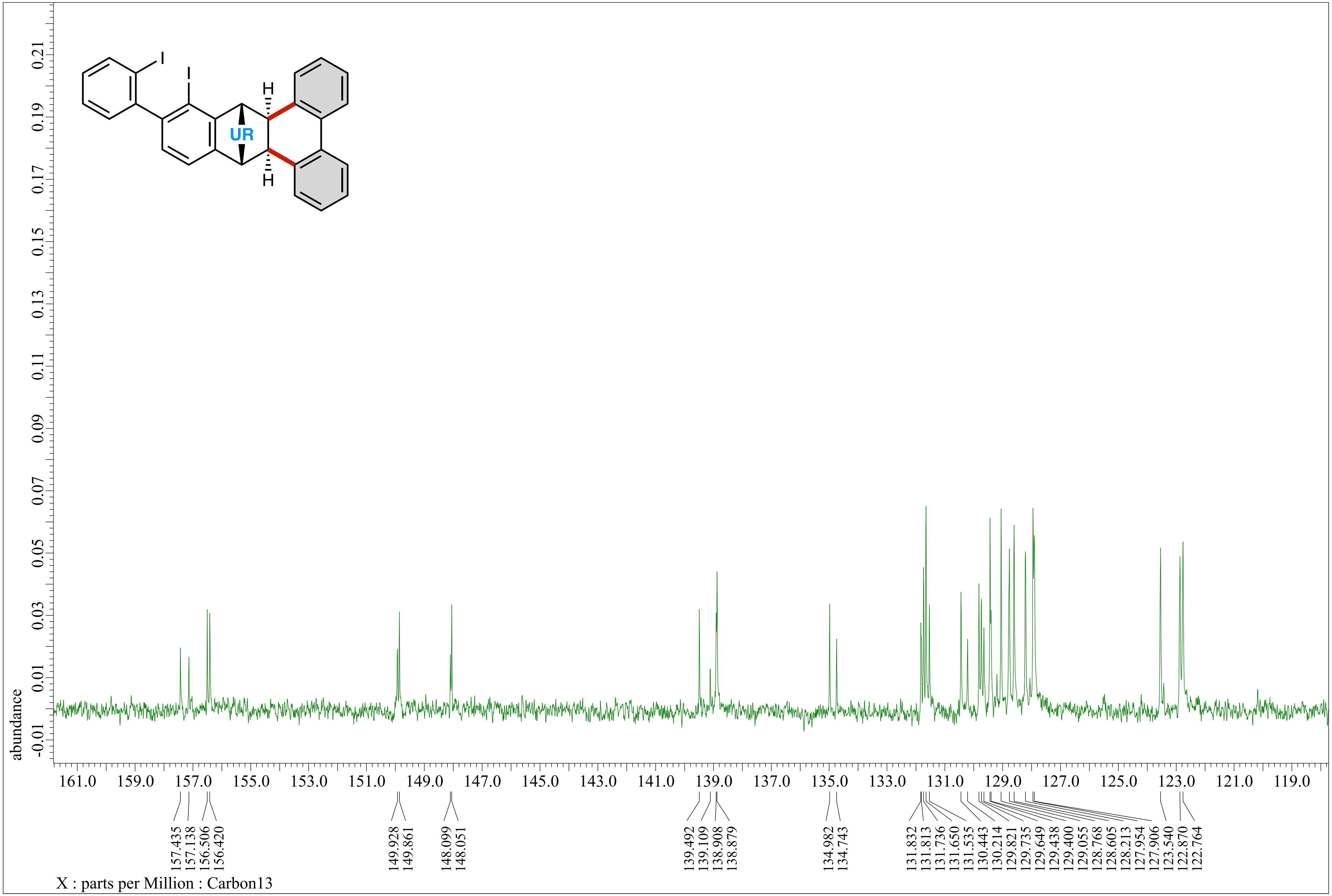

^{1}H NMR spectrum of **4na** (solvent: $CDCl_3$)

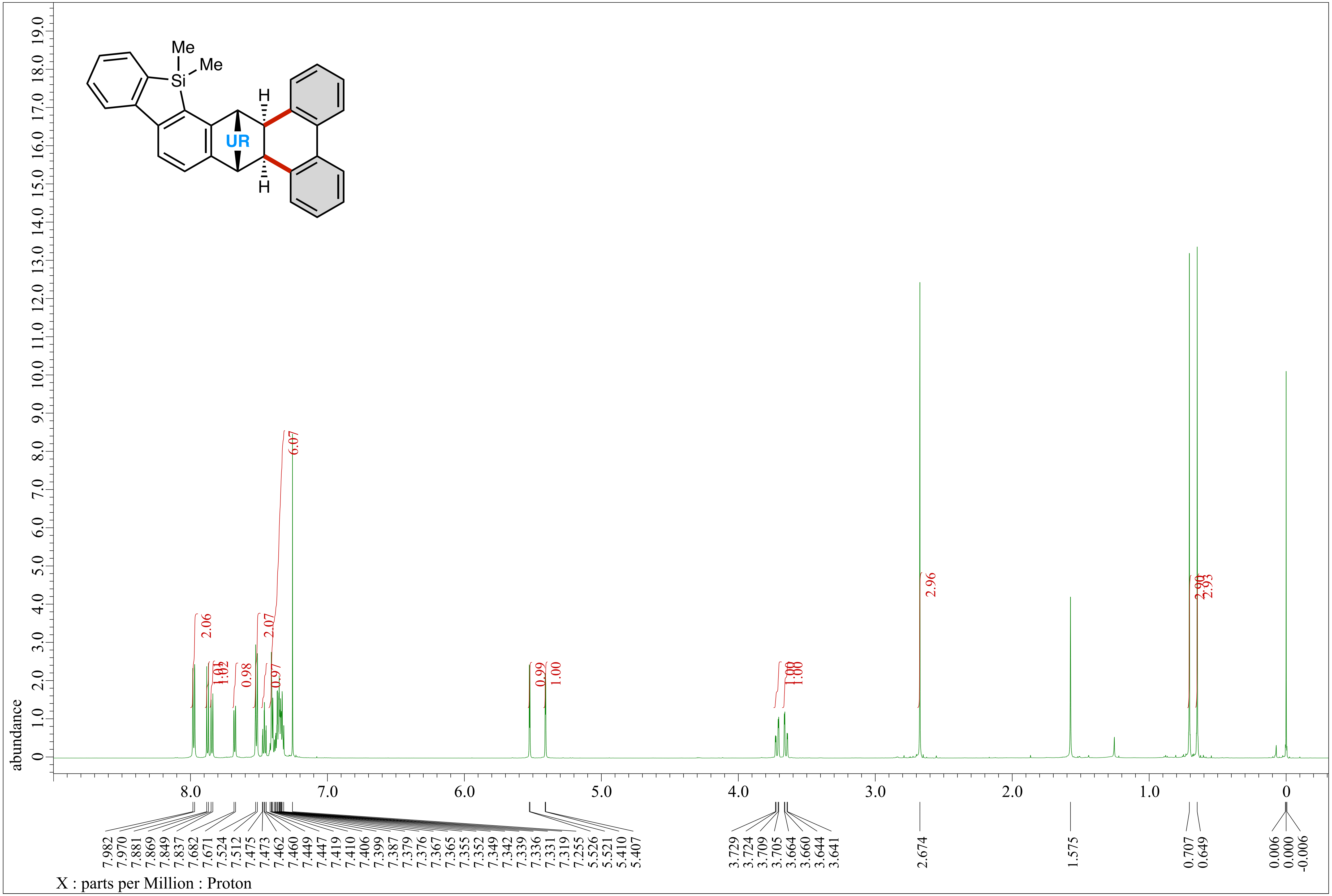

^{1}H NMR spectrum (inset) of **4na** (solvent: $CDCl_3$)

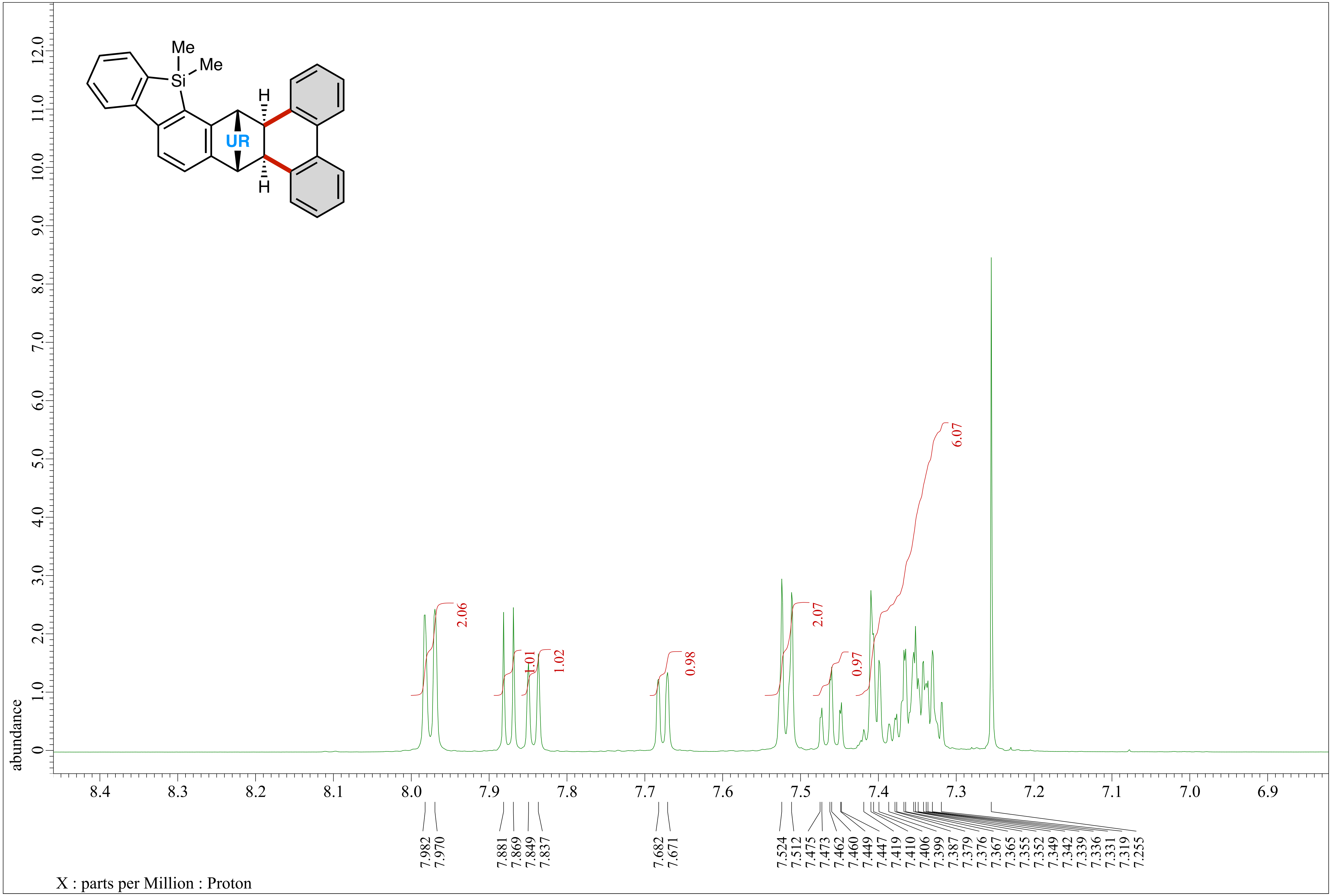

^{13}C NMR spectrum of **4na** (solvent: $CDCl_3$)

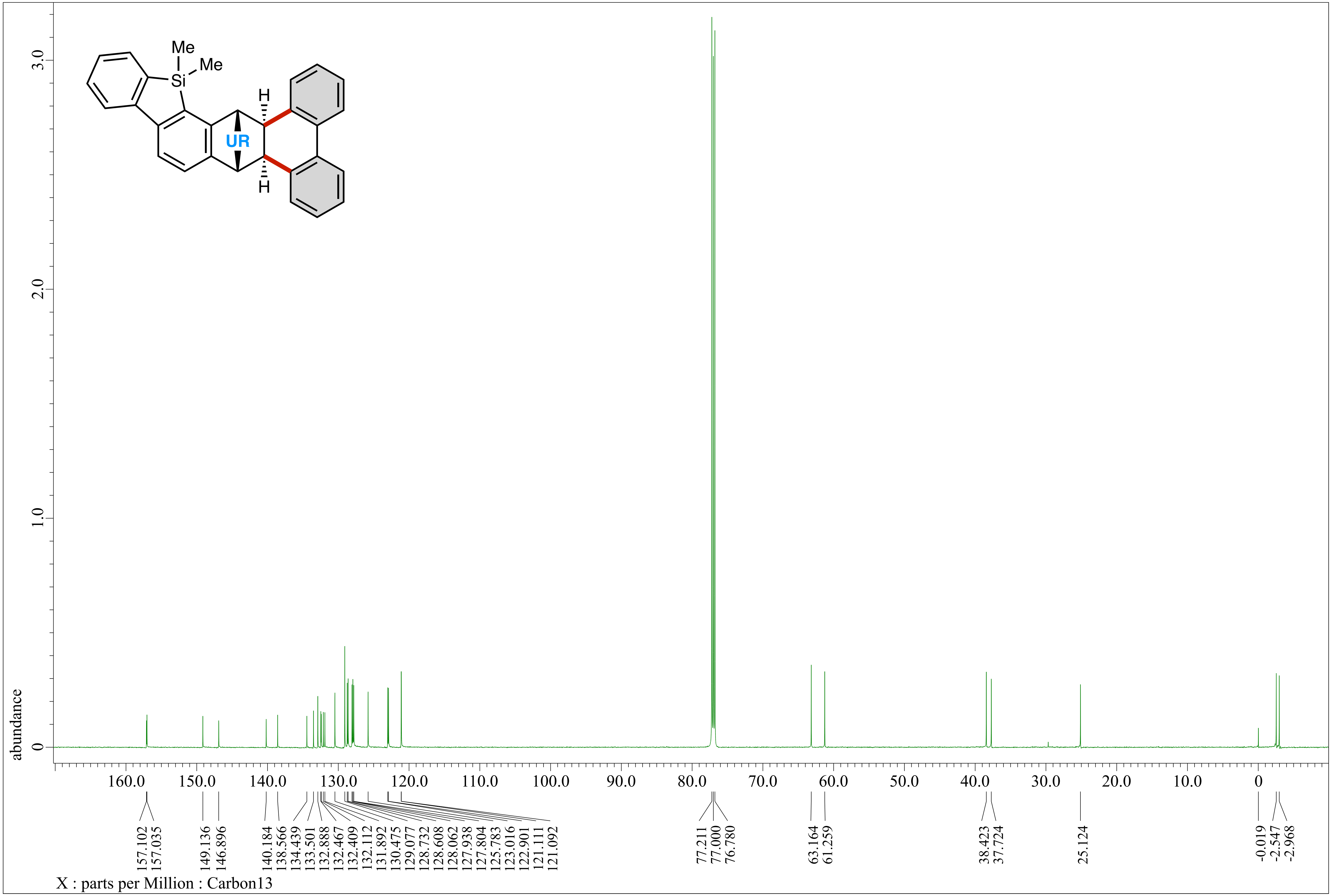

^{13}C NMR spectrum (inset) of **4na** (solvent: $CDCl_3$)

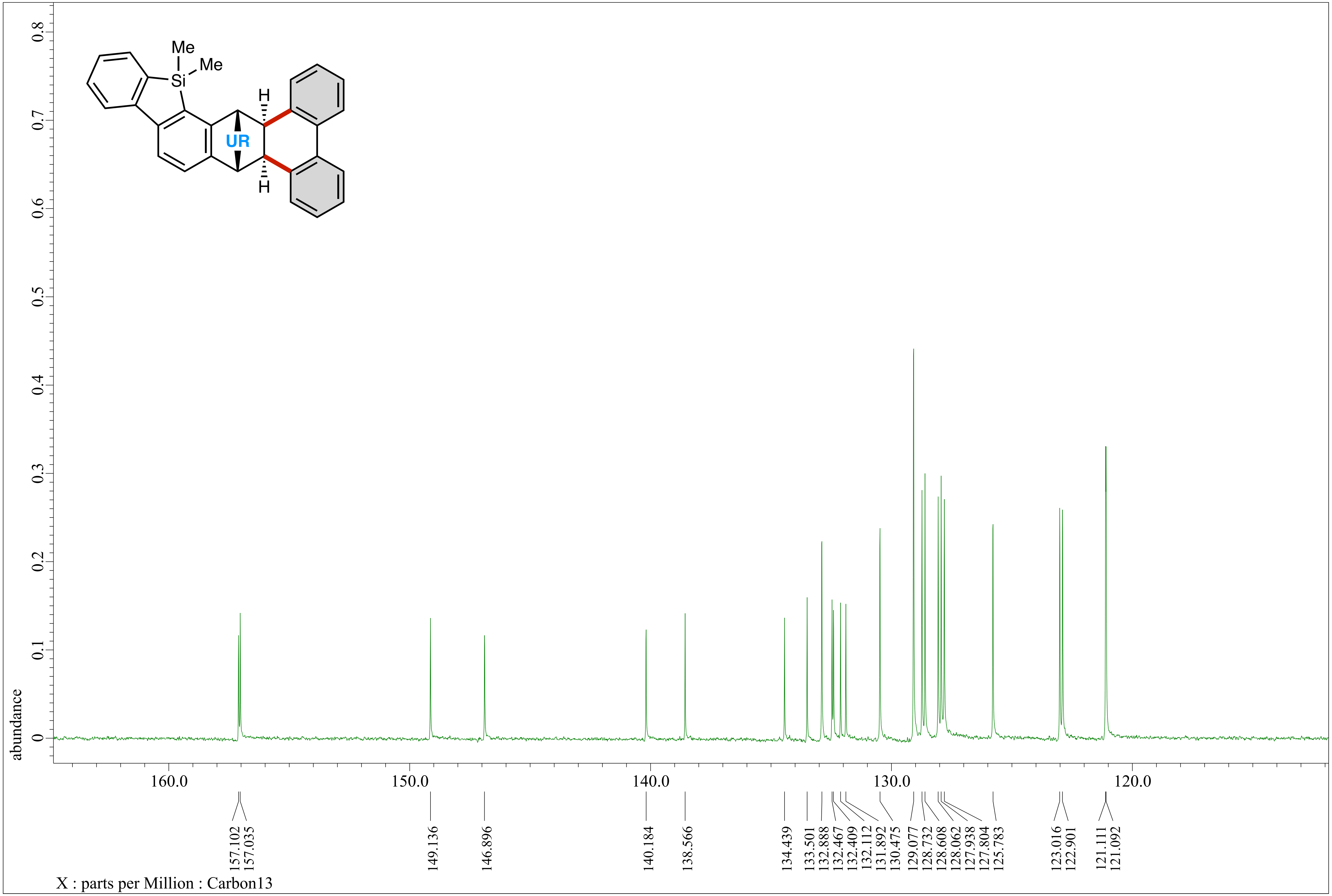

^1H NMR spectrum of **4oa** (solvent: $CDCl_3$)

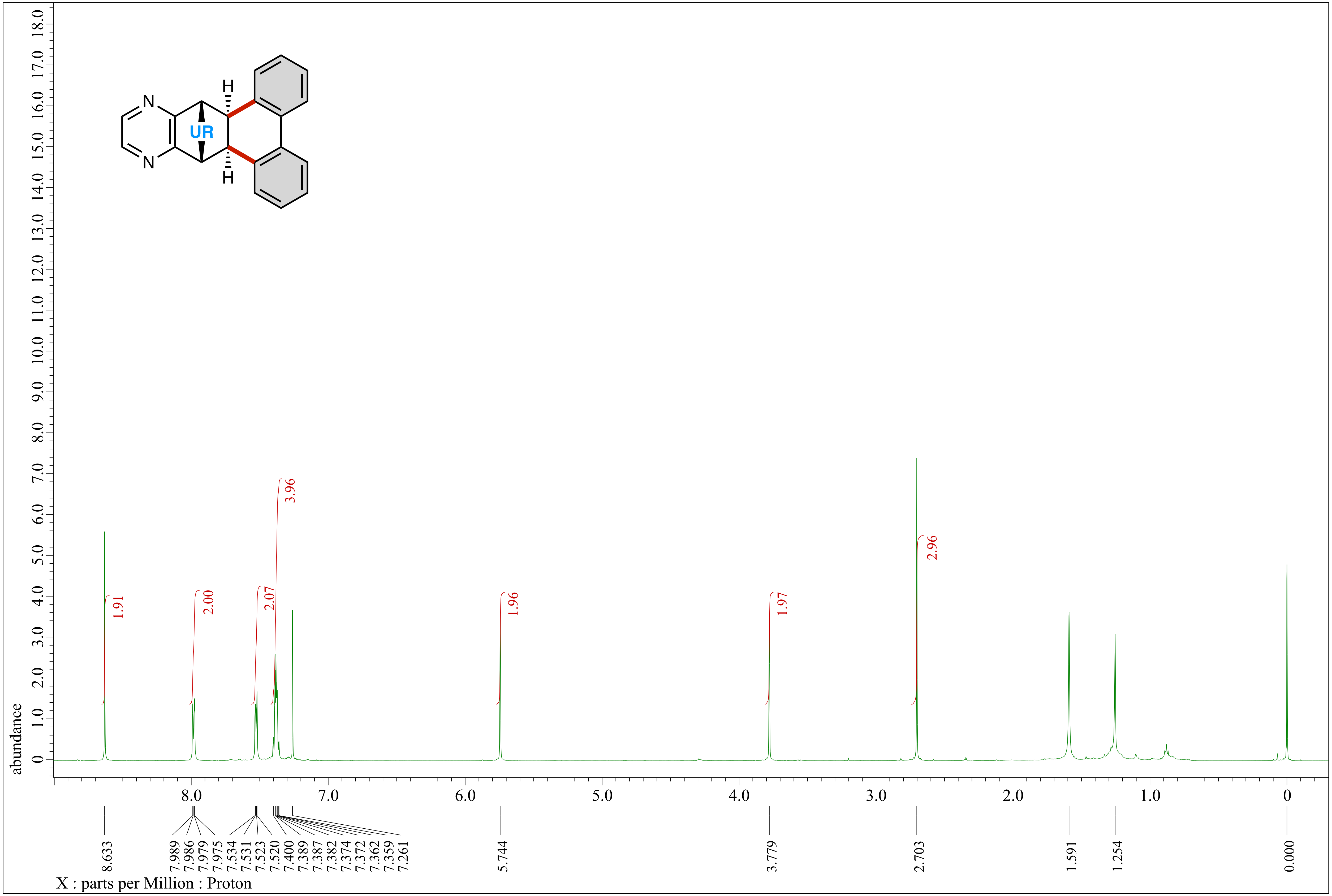

^{1}H NMR spectrum (inset) of **4oa** (solvent: $CDCl_3$)

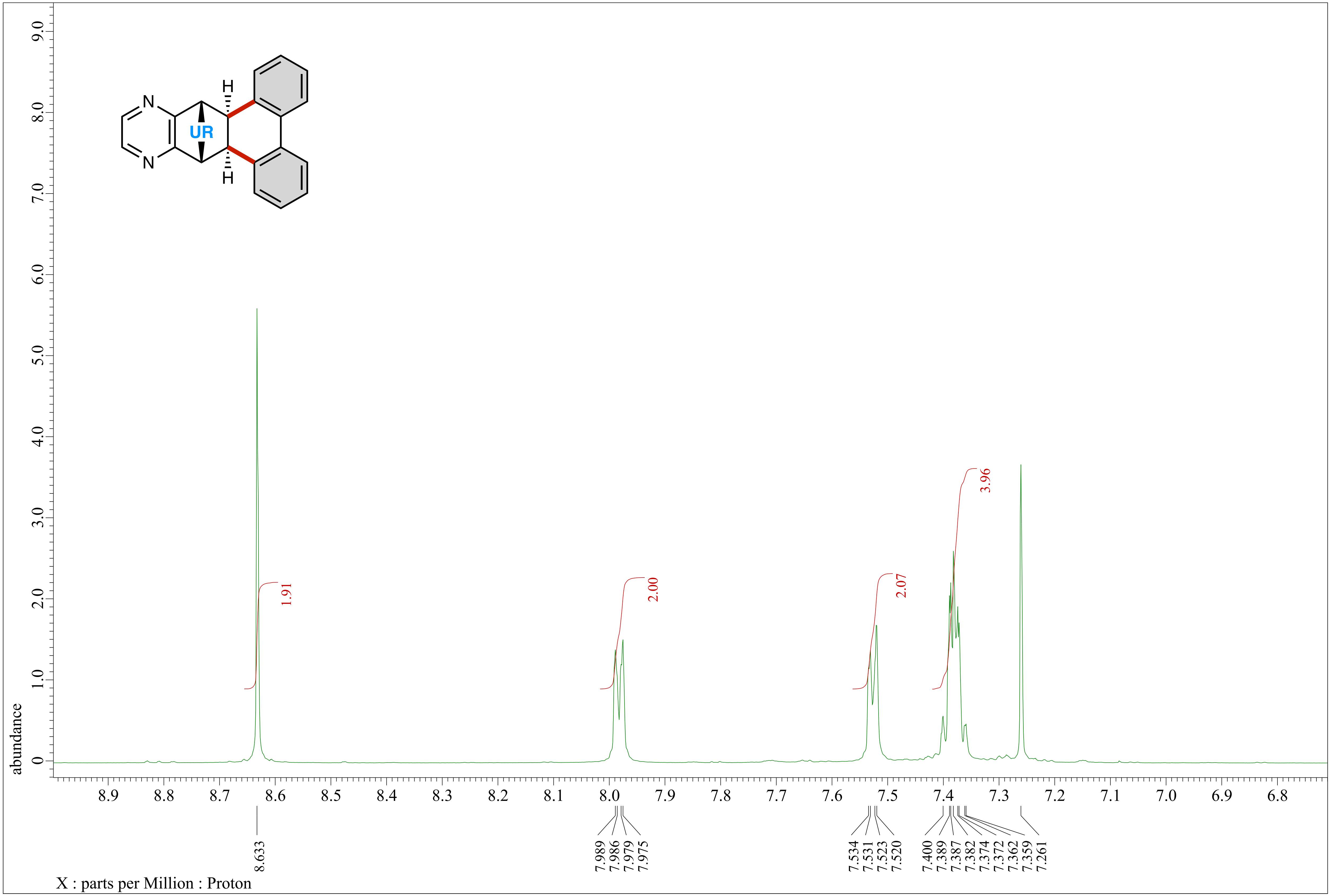

^{13}C NMR spectrum of **4oa** (solvent: $CDCl_3$)

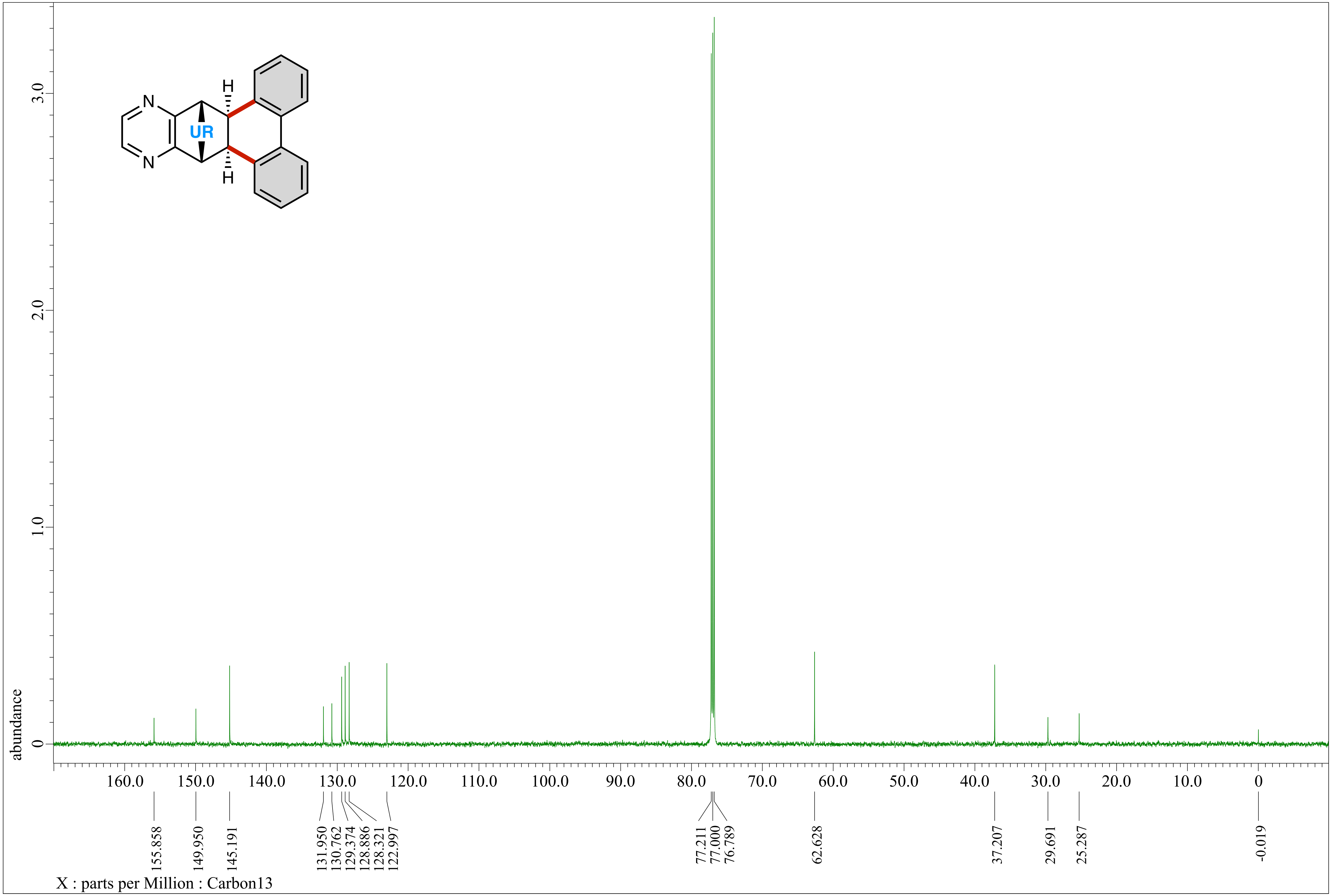

^{13}C NMR spectrum (inset) of **4oa** (solvent: $CDCl_3$)

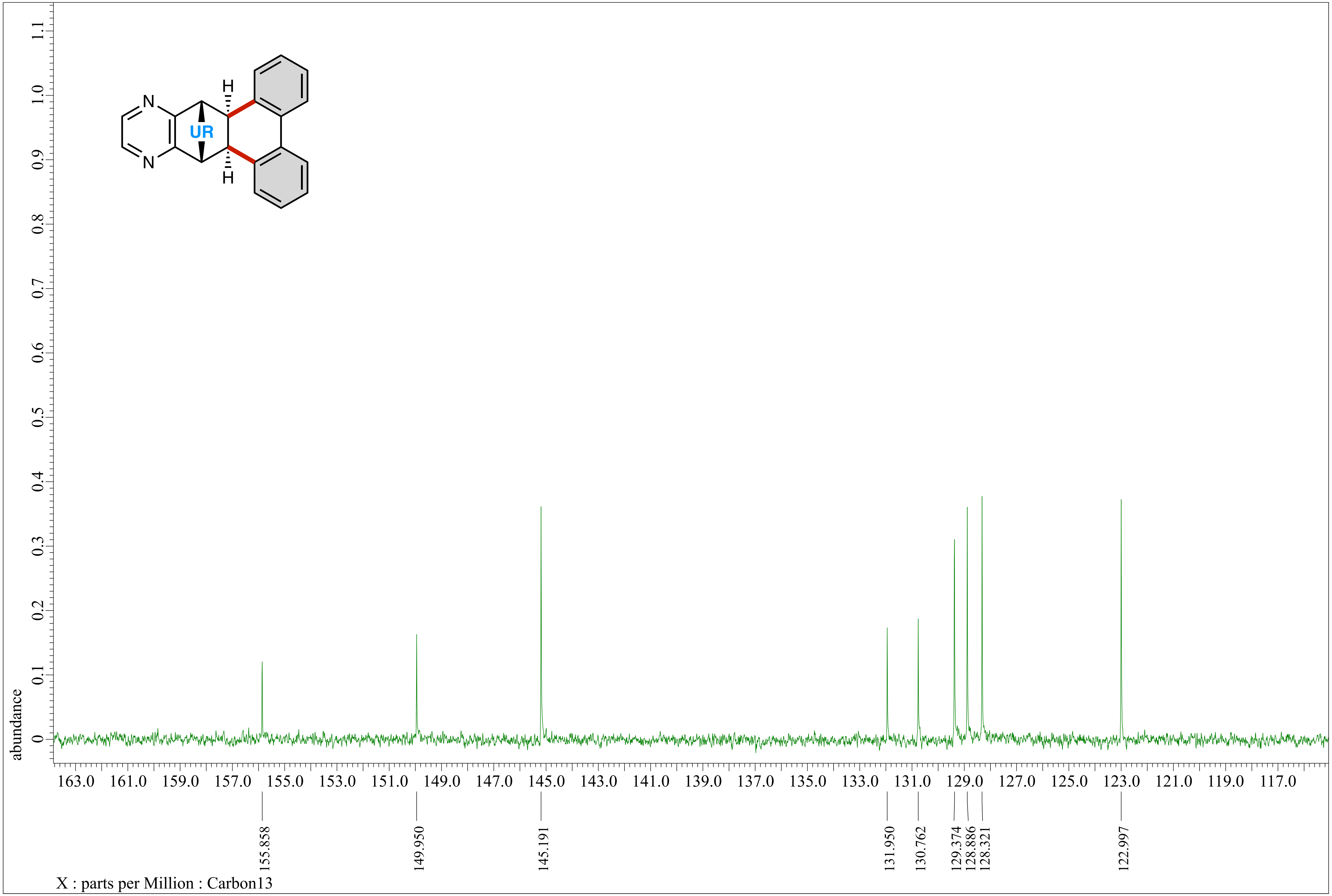

^{1}H NMR spectrum of **4pa**, **4pa′** and **4pa″** (solvent: $CDCl_3$)

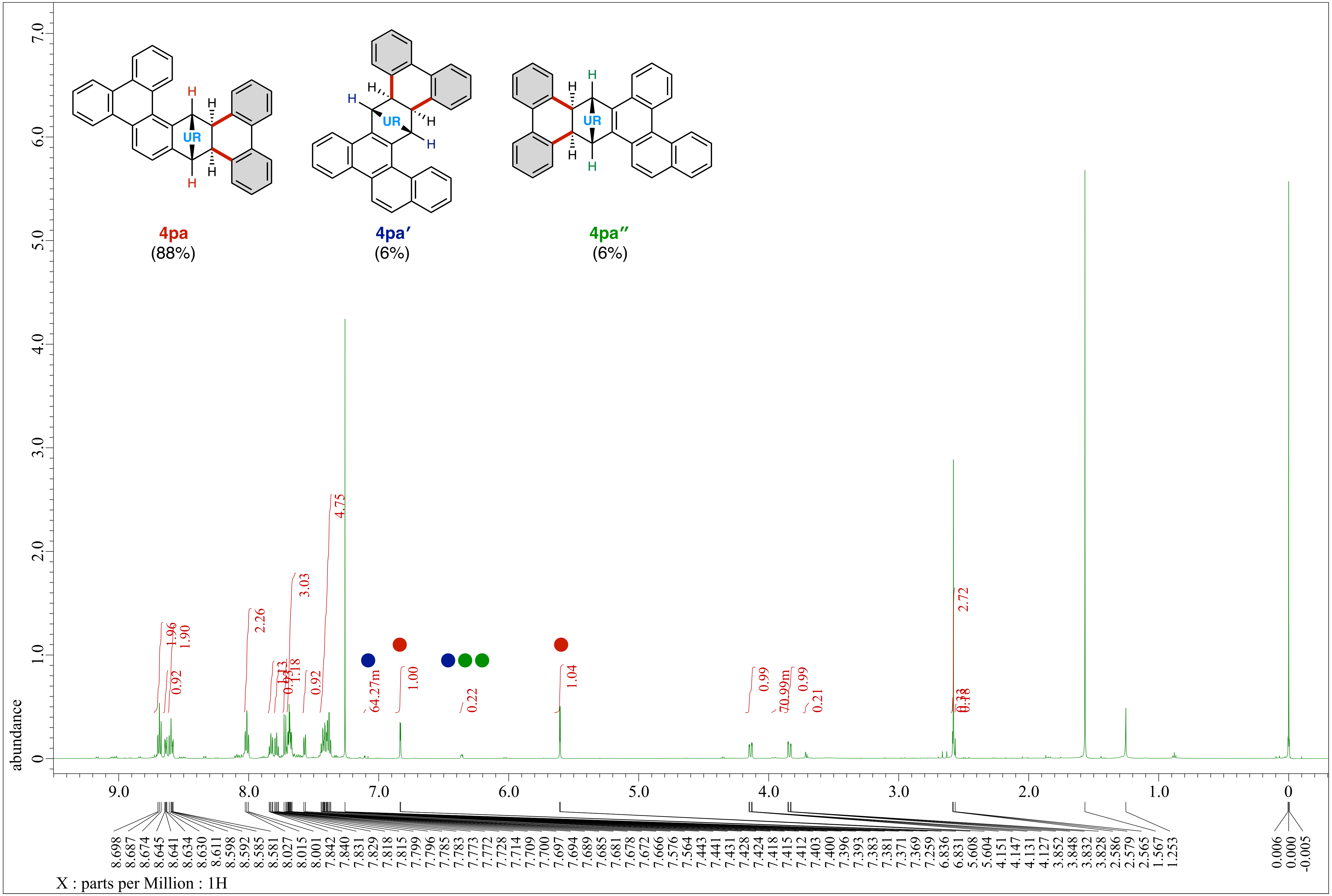

^{1}H NMR spectrum (inset) of **4pa**, **4pa′** and **4pa″** (solvent: $CDCl_3$)

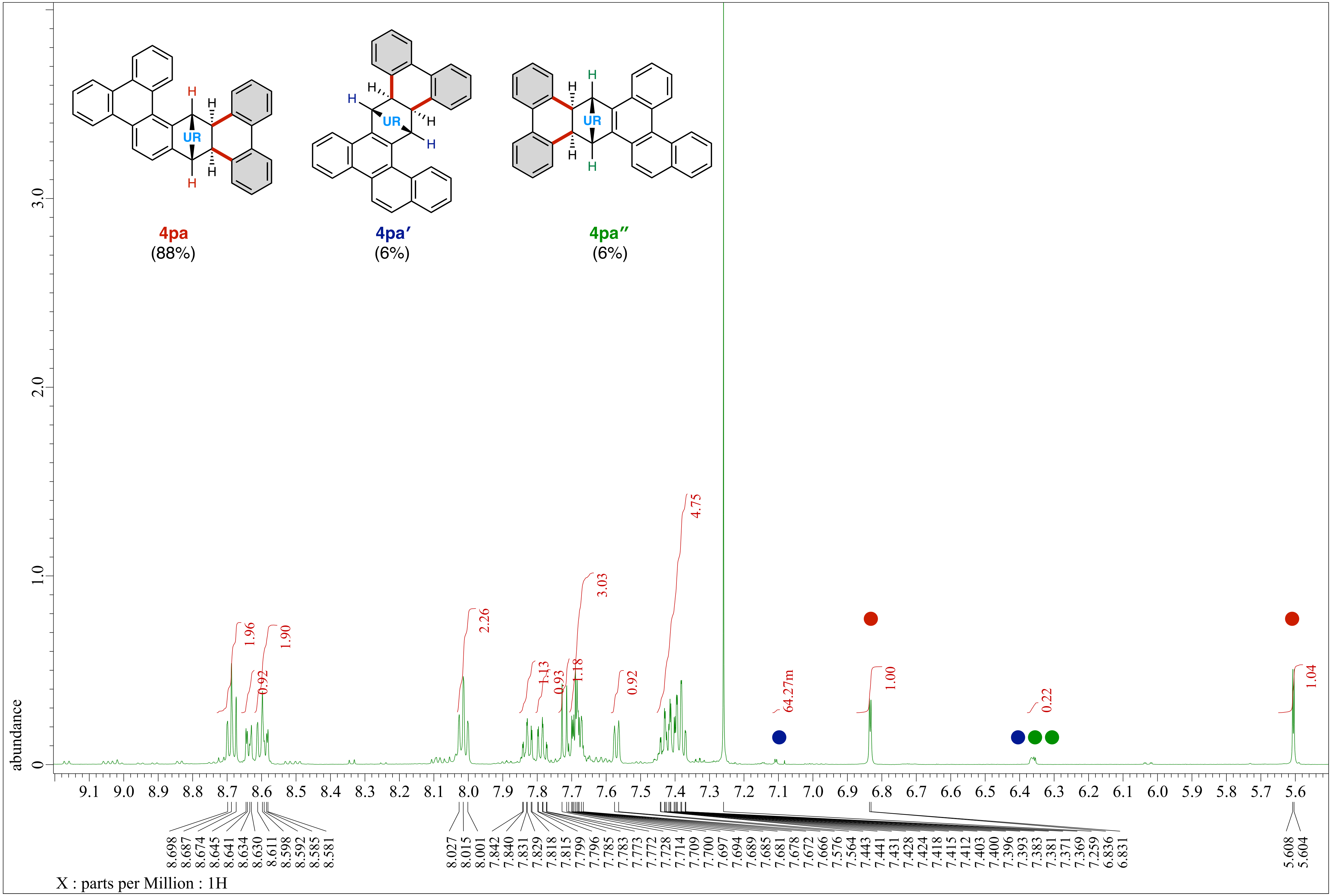

^{13}C NMR spectrum of **4pa** (solvent: $CDCl_3$)

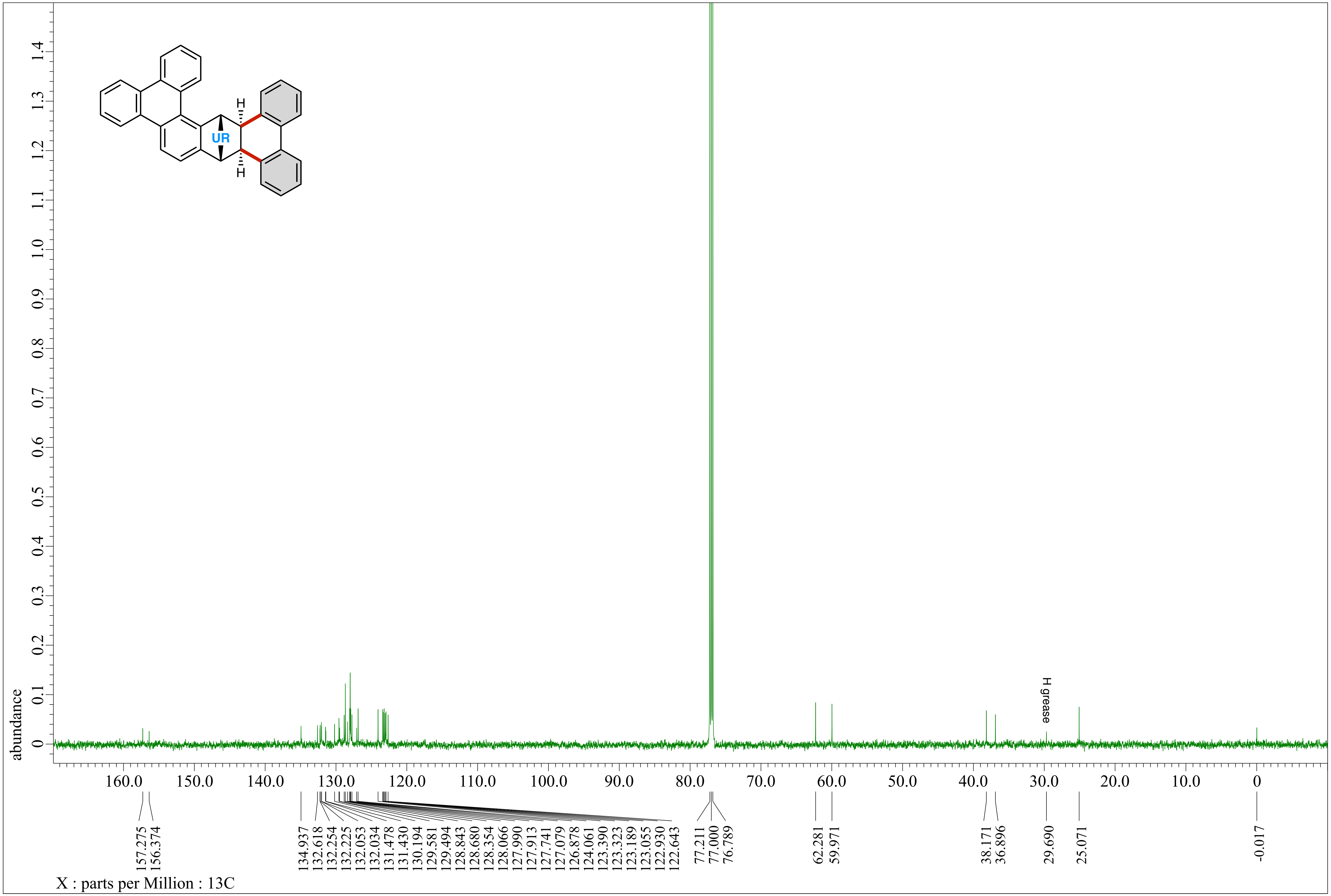

^{13}C NMR spectrum (inset) of **4pa** (solvent: $CDCl_3$)

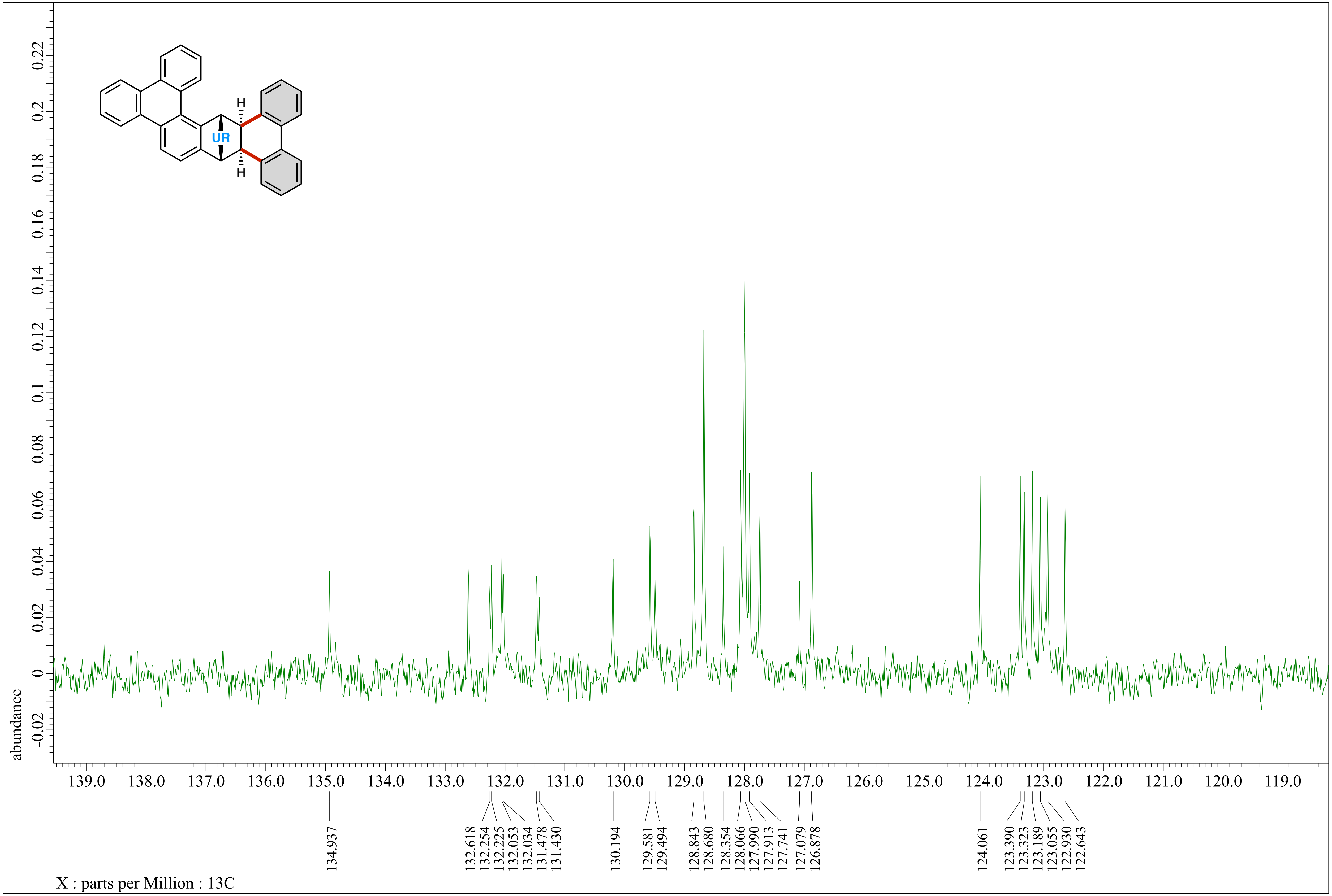

^{1}H NMR spectrum of **4qa**, **4qa′**, **4qa″** and **4qa‴** (solvent: $CDCl_3$)

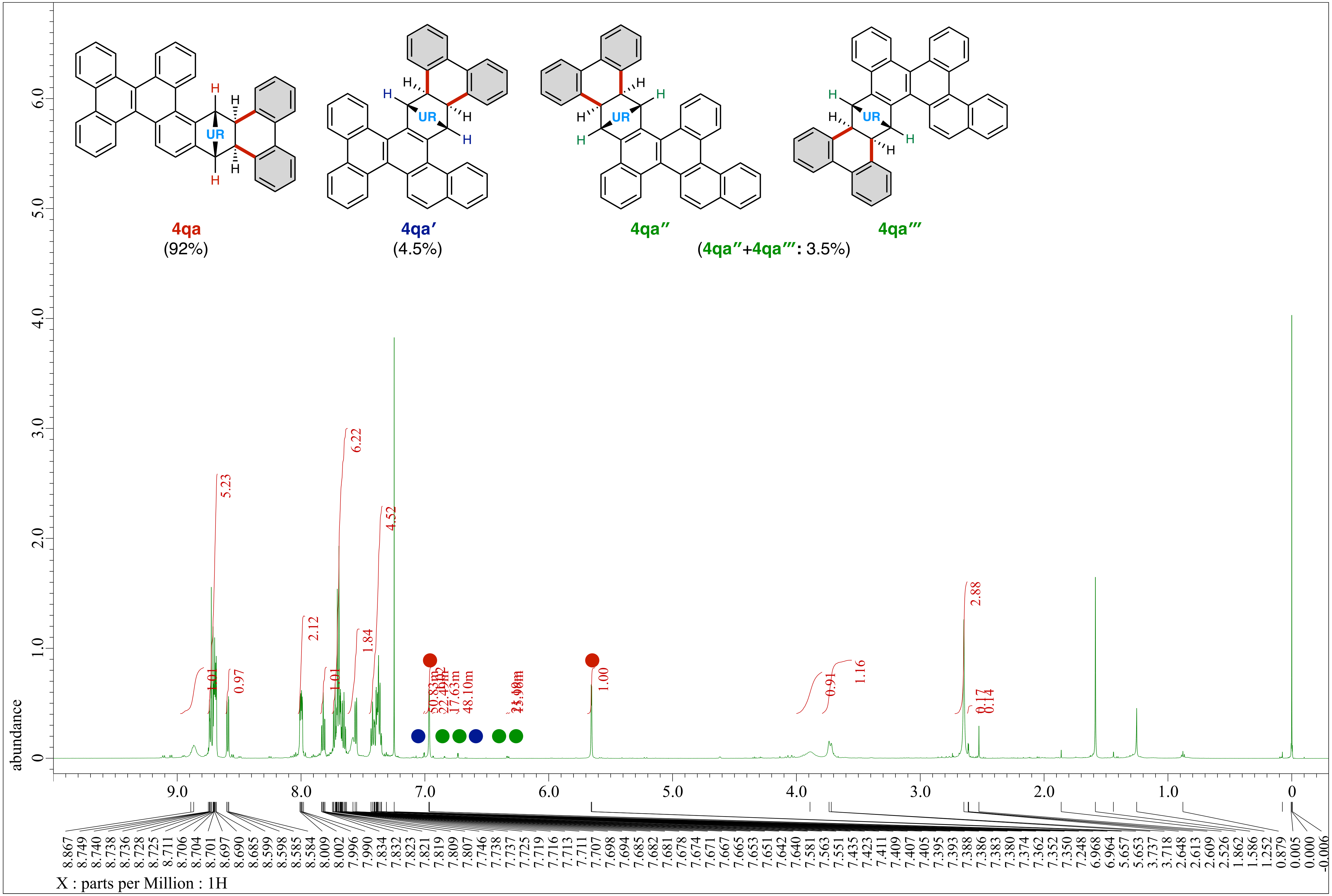

^{1}H NMR spectrum (inset) of **4qa**, **4qa′**, **4qa″** and **4qa‴** (solvent: $CDCl_3$)

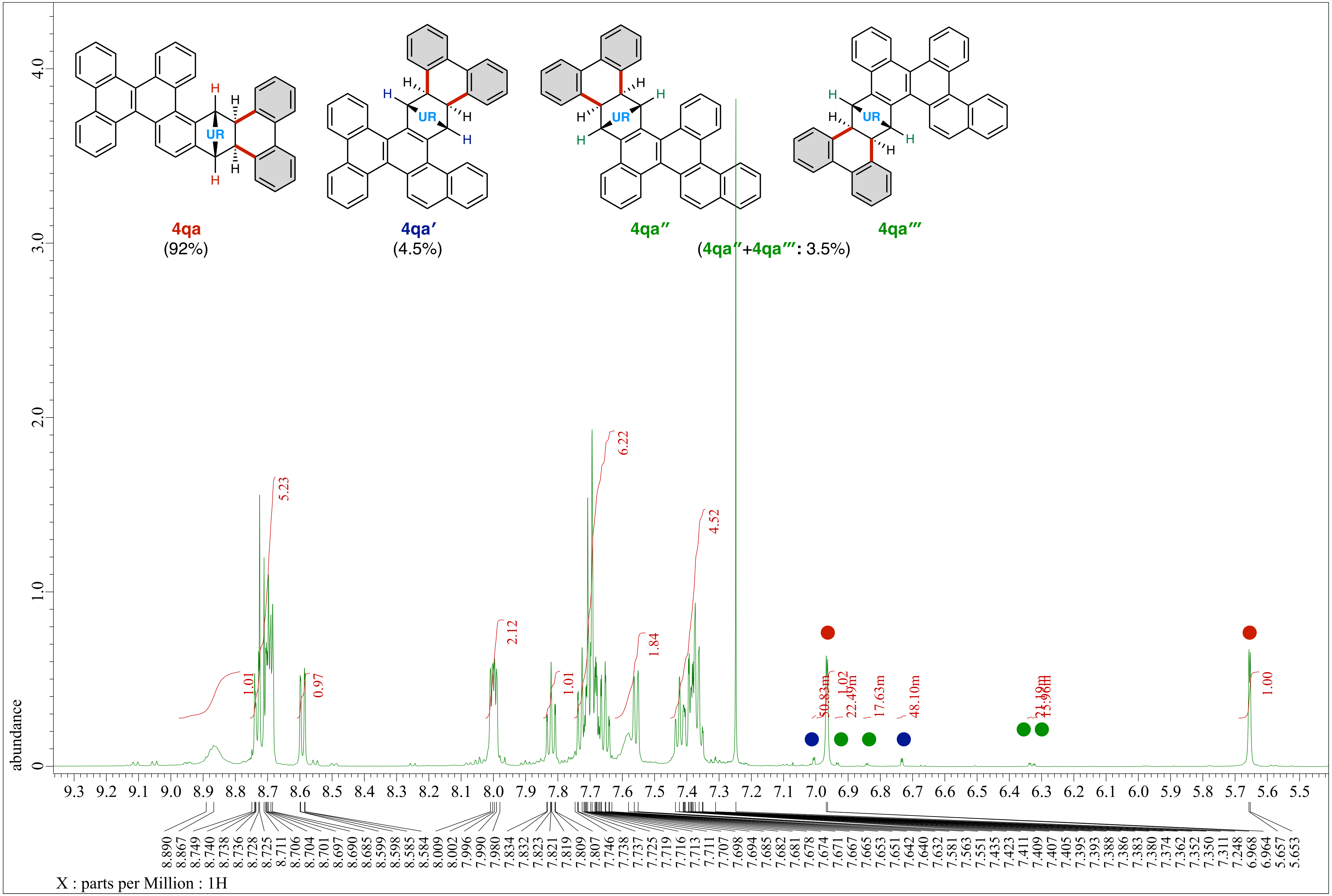

^{13}C NMR spectrum of **4qa** (solvent: $CDCl_3$)

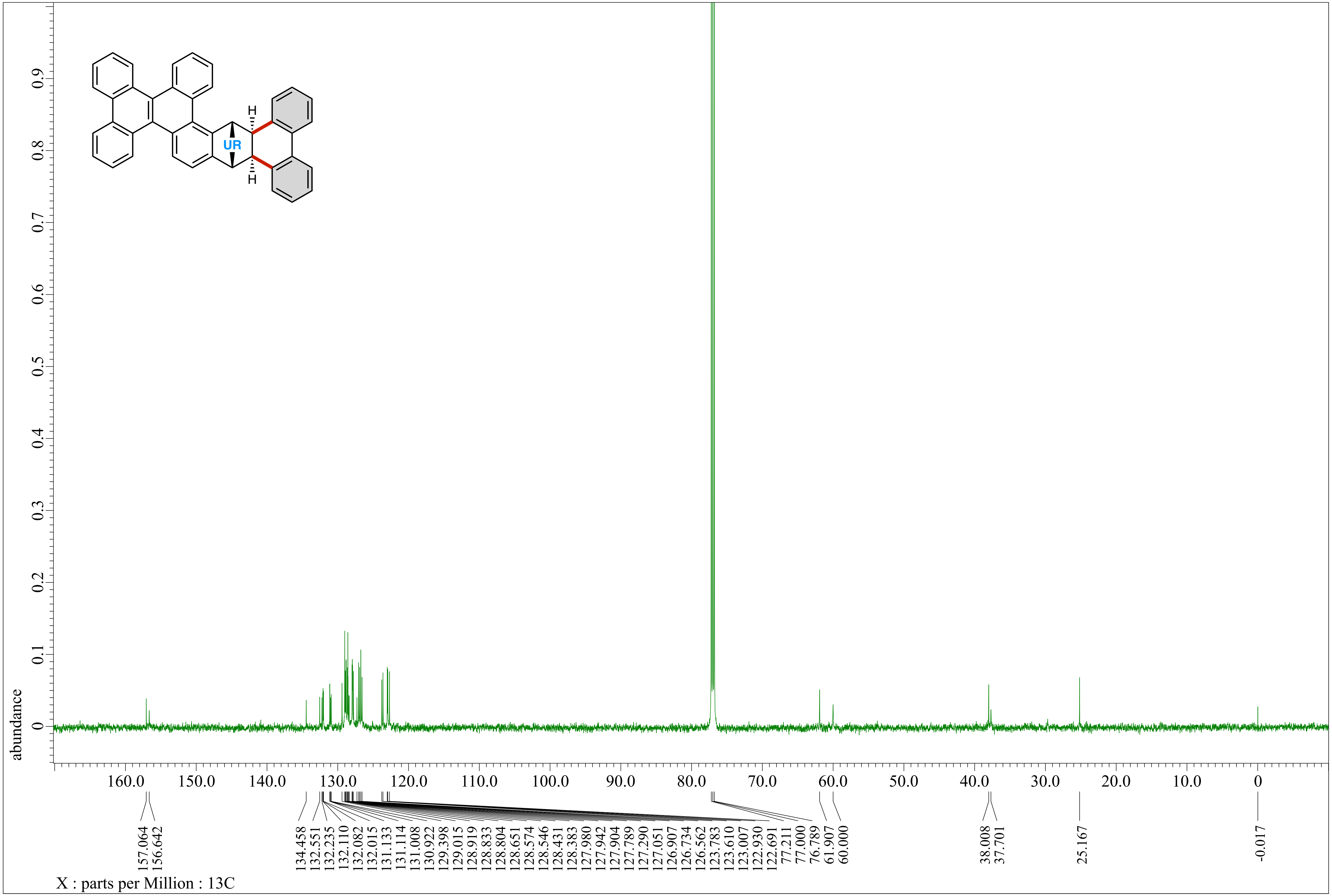

^{13}C NMR spectrum (inset) of **4qa** (solvent: $CDCl_3$)

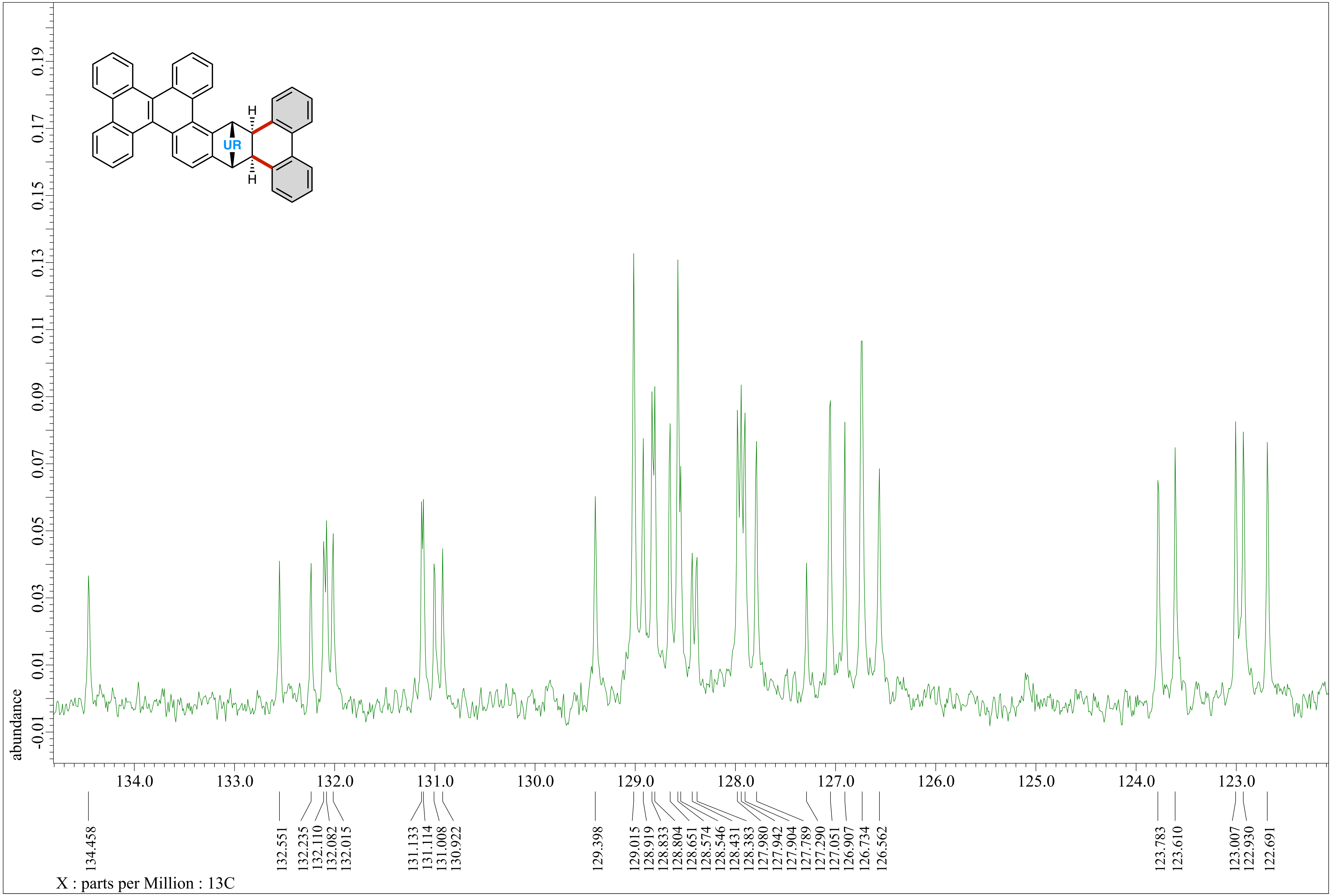

^{1}H NMR spectrum of **2r″** (solvent: acetone-d_6)

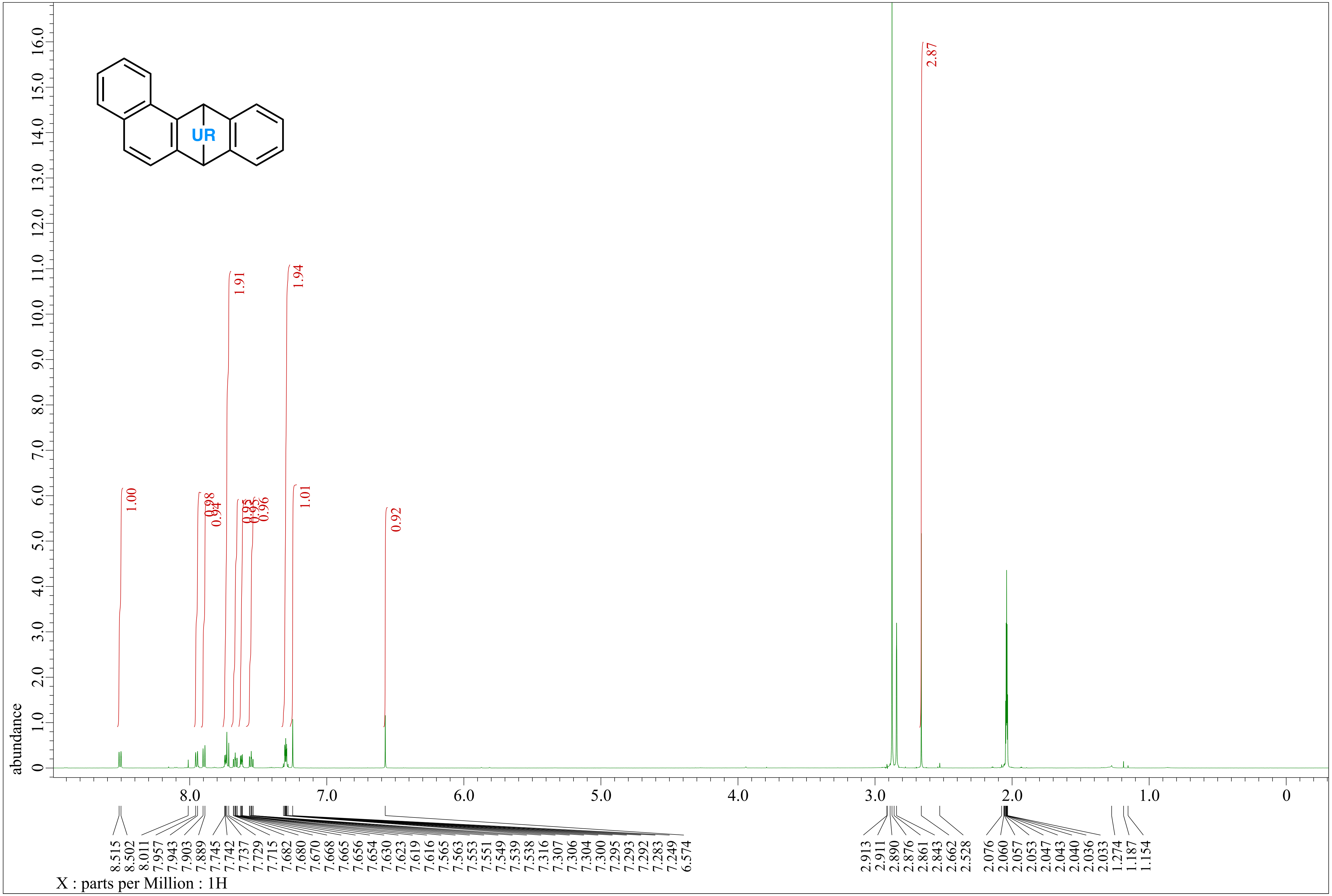

^{1}H NMR spectrum (inset) of **2r″** (solvent: acetone-d_6)

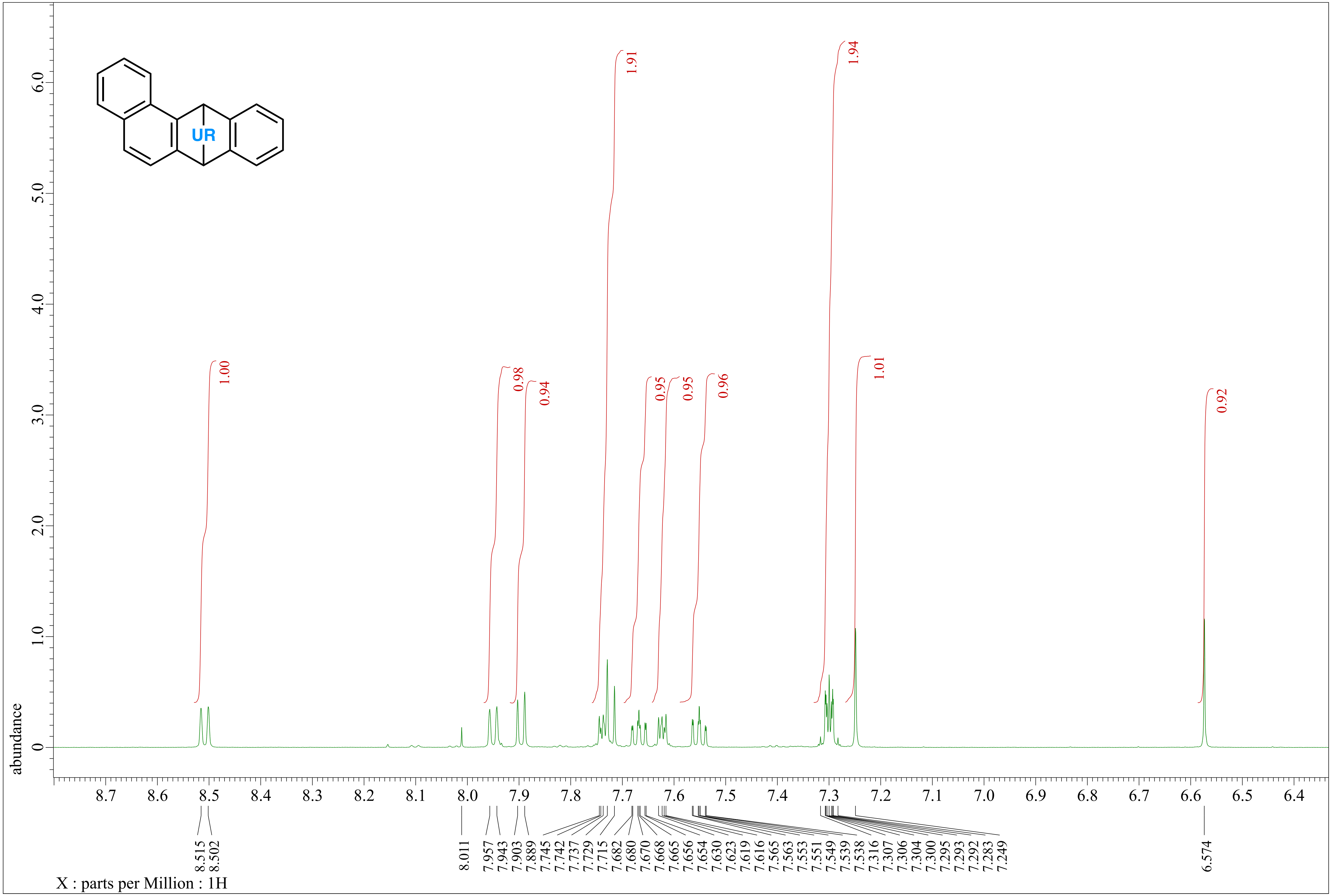

^{13}C NMR spectrum of **2r″** (solvent: acetone-d_6)

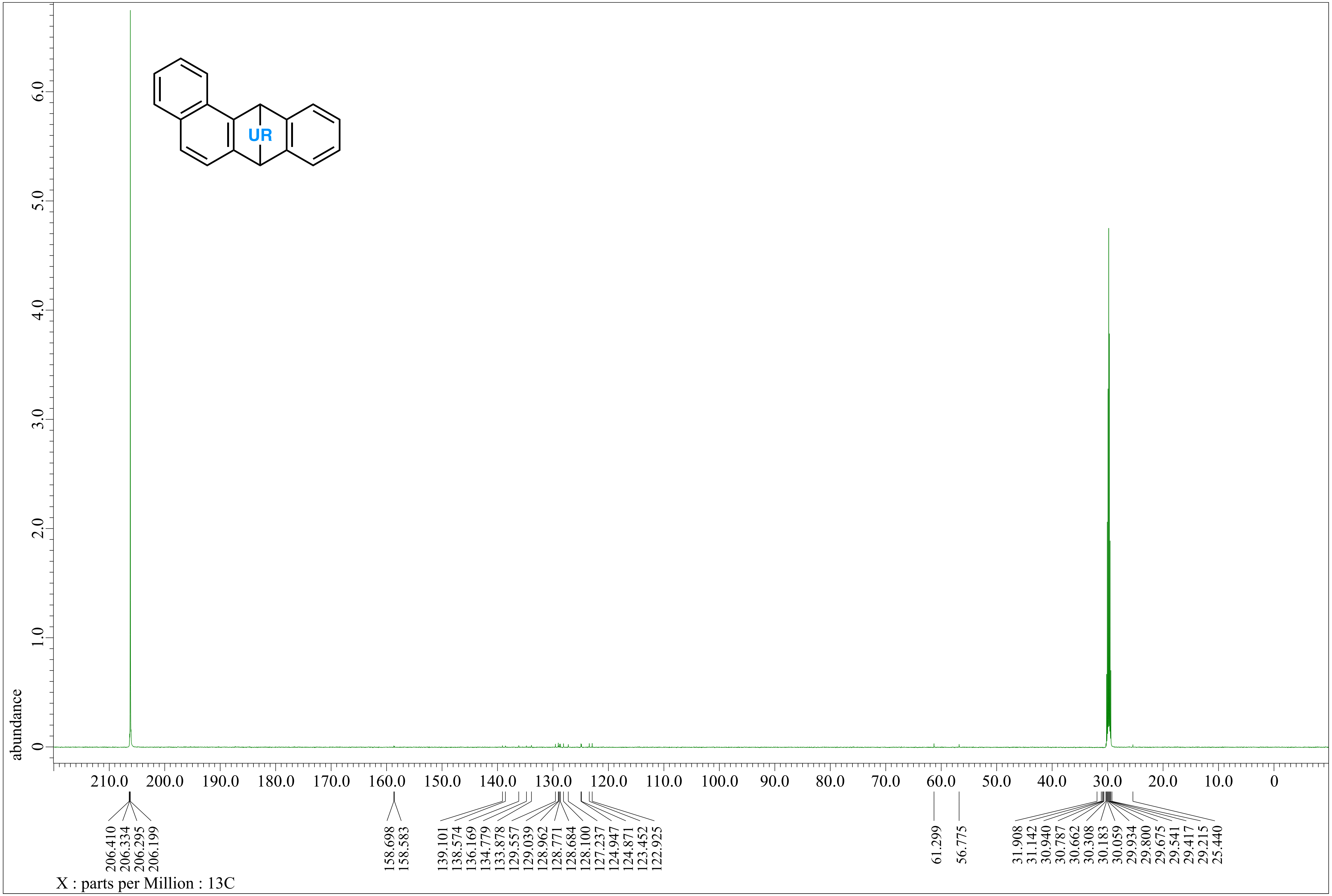

^{13}C NMR spectrum (inset) of **2r″** (solvent: acetone-d_6)

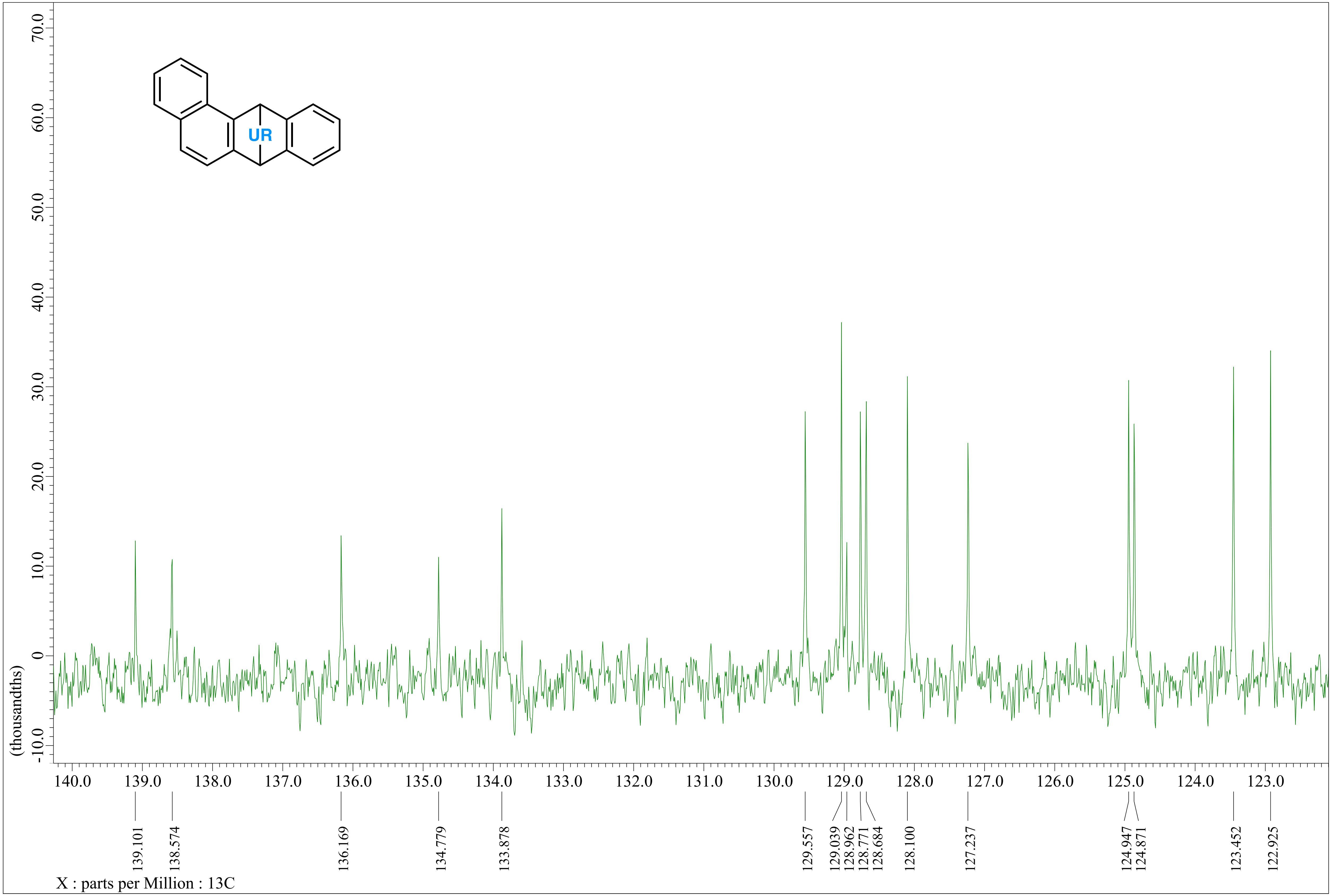

^{1}H NMR spectrum of **4ra** and **4ra′** (solvent: $CDCl_3$)

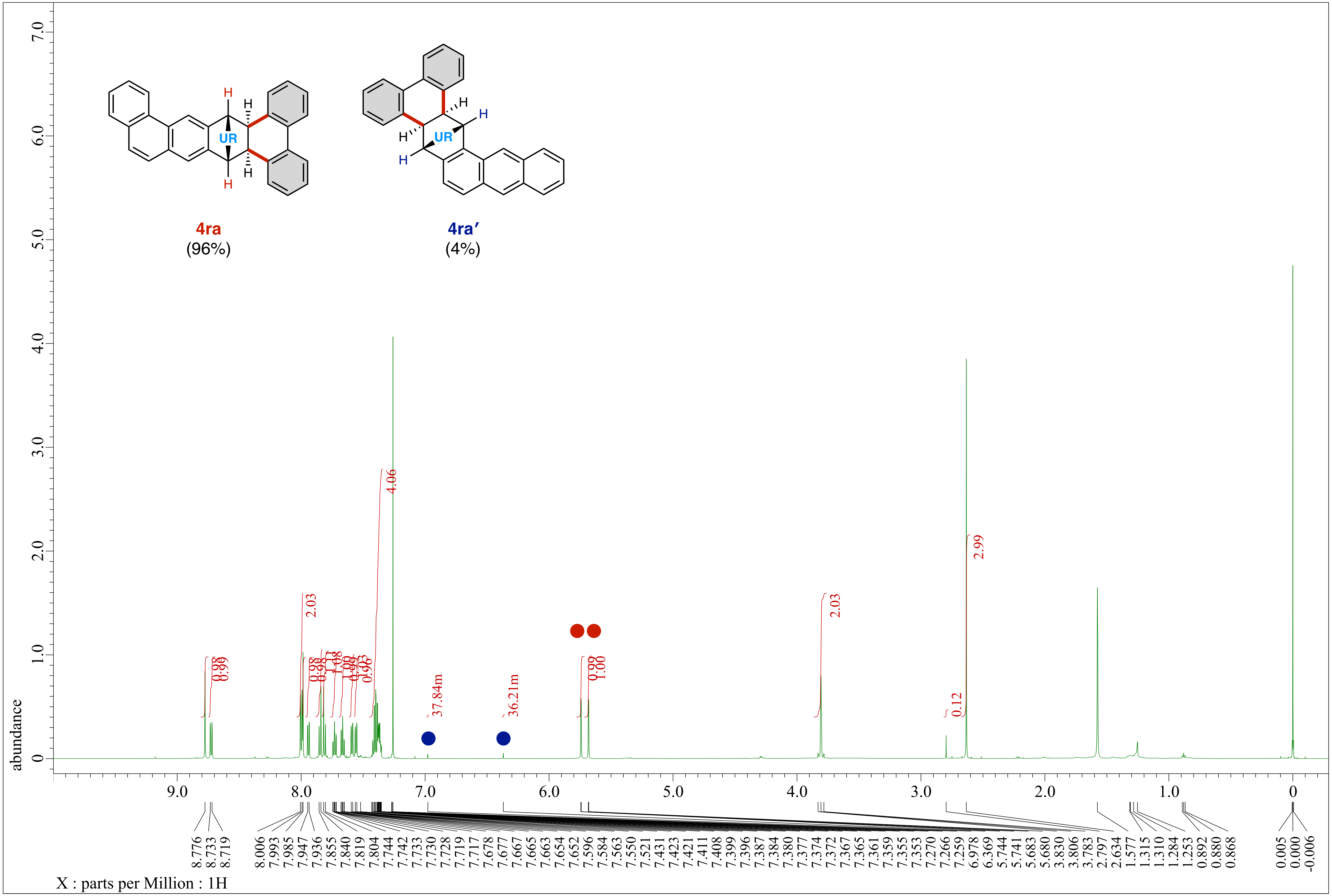

^{1}H NMR spectrum (inset) of **4ra** and **4ra′** (solvent: $CDCl_3$)

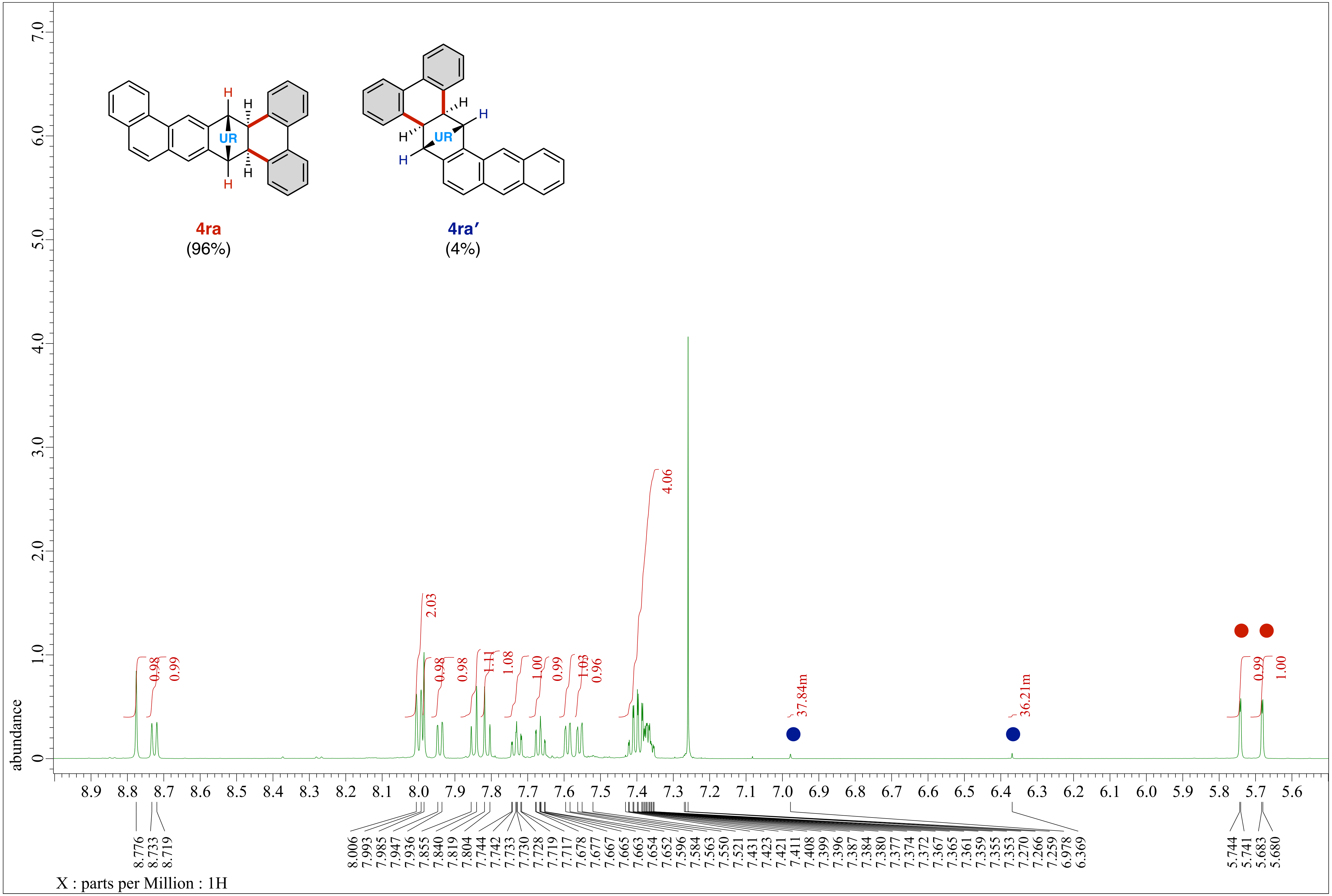

^{13}C NMR spectrum of **4ra** (solvent: $CDCl_3$)

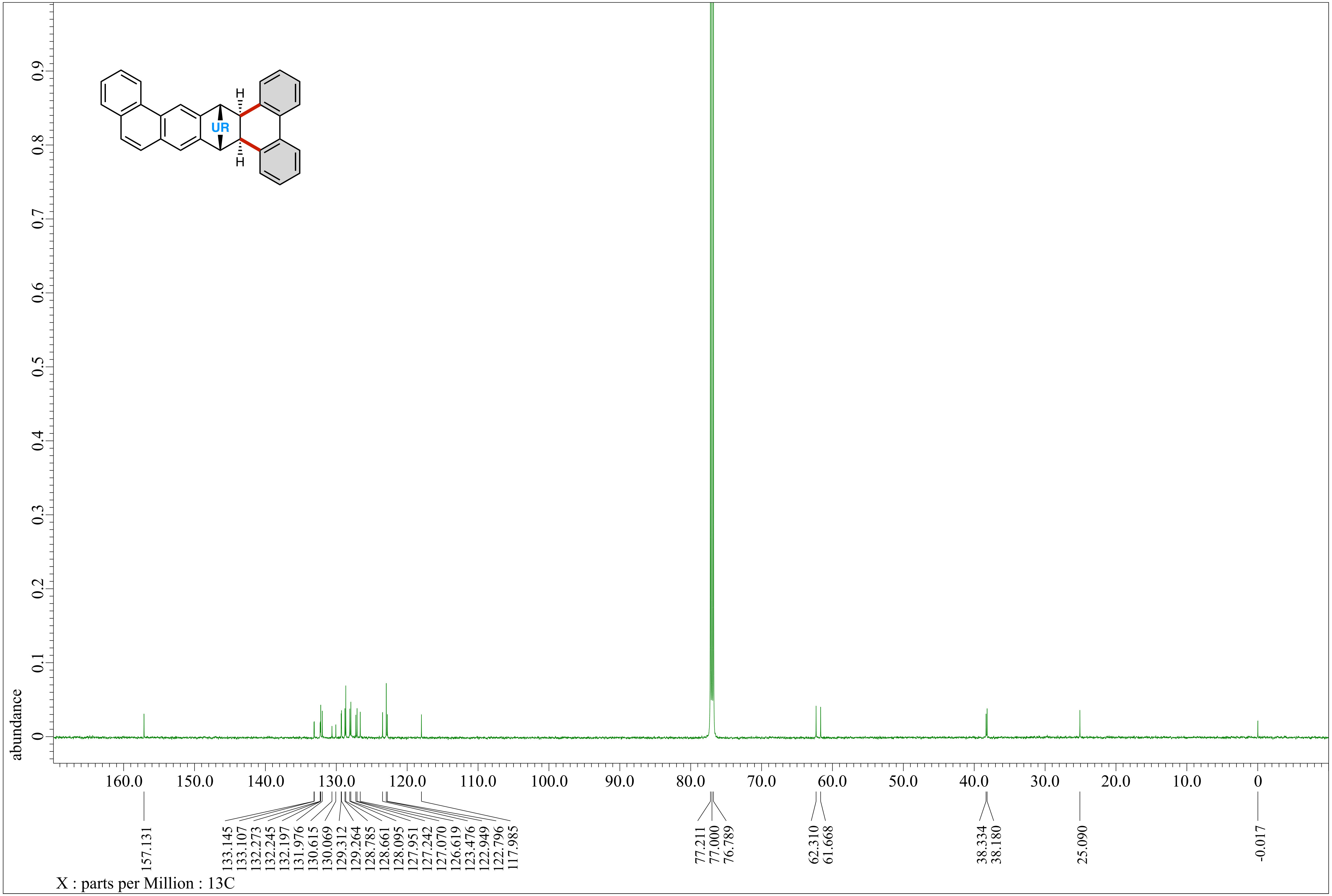

^{13}C NMR spectrum (inset) of **4ra** (solvent: $CDCl_3$)

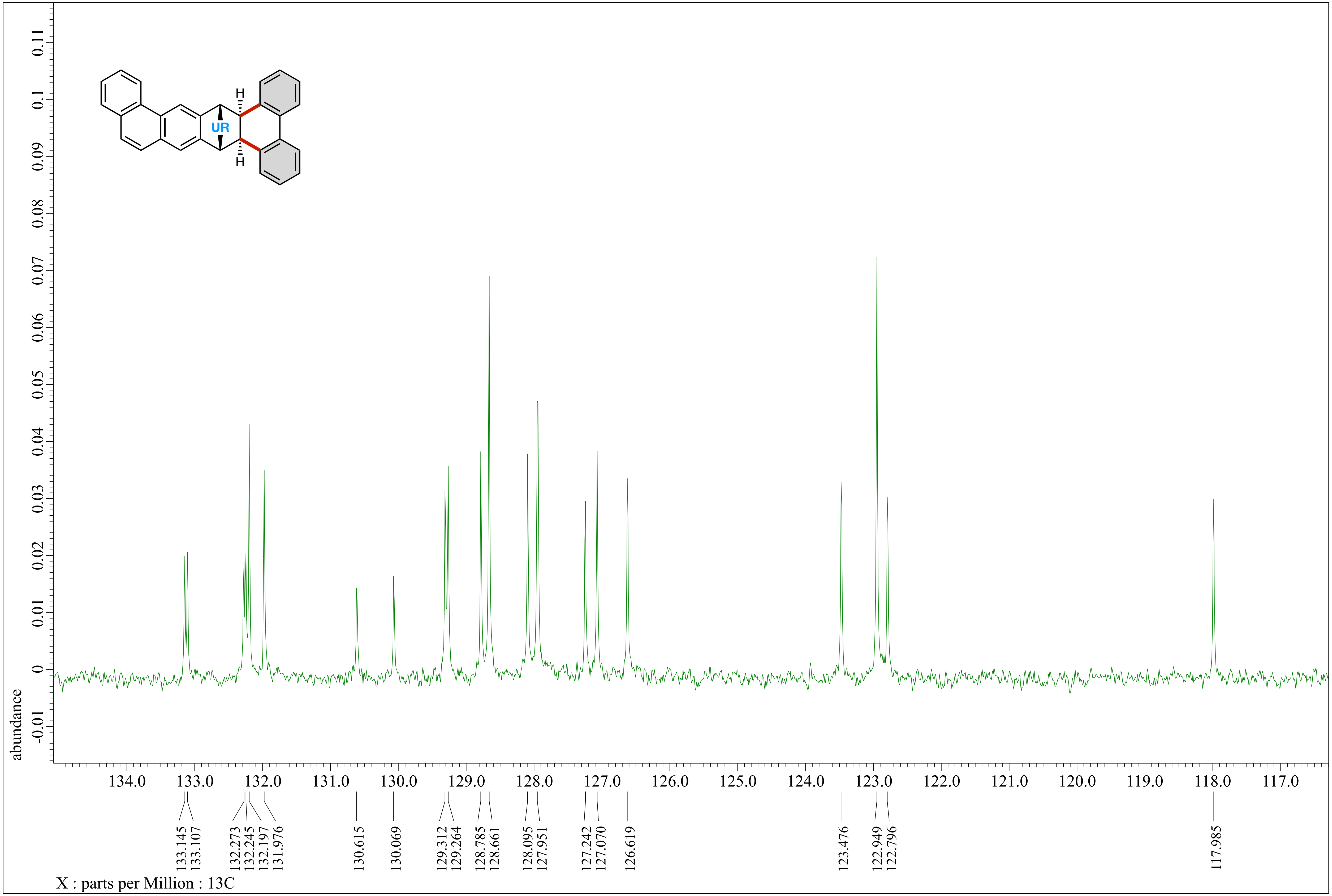

^{1}H NMR spectrum of **4gb** (solvent: $CDCl_3$)

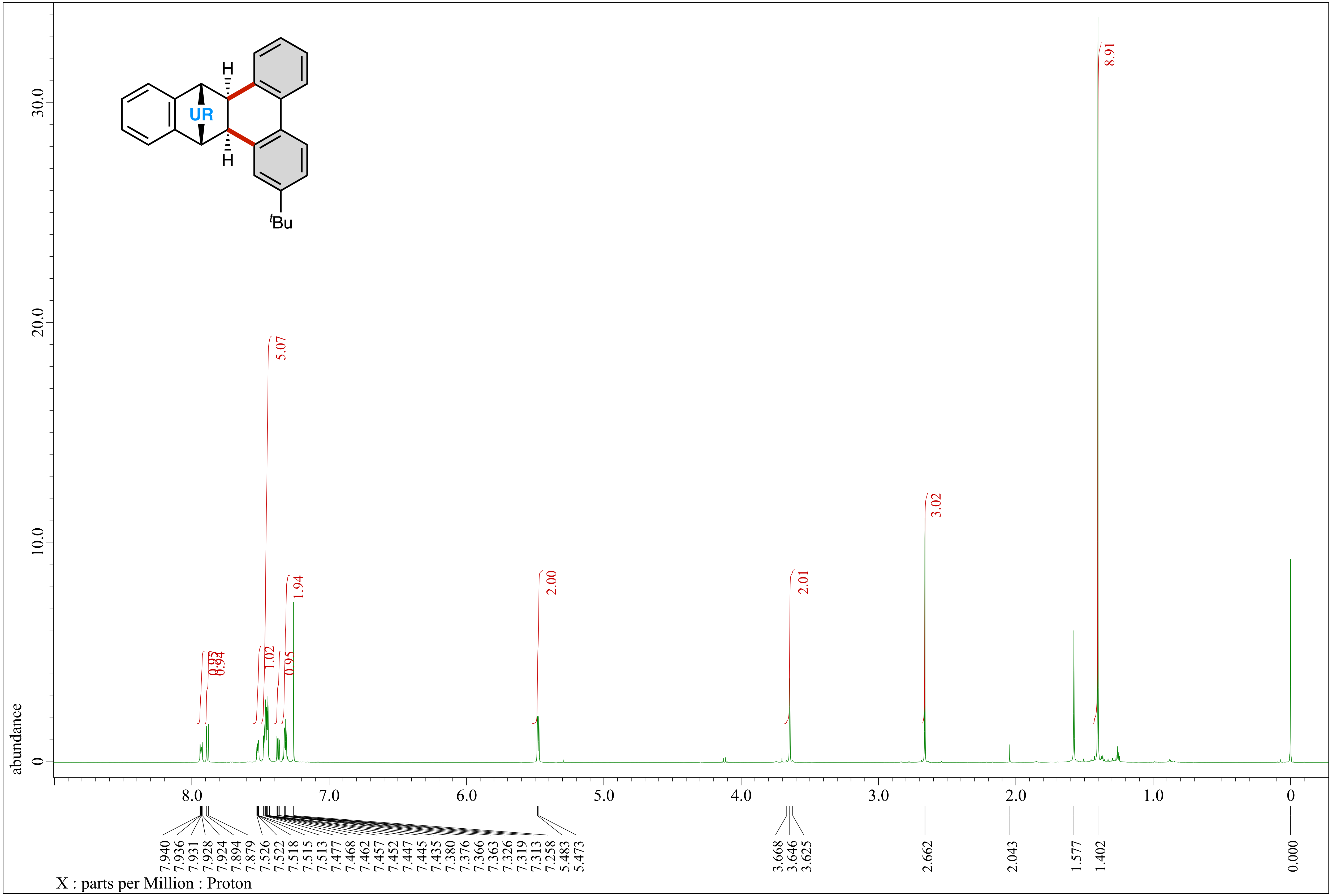

^{1}H NMR spectrum (inset) of **4gb** (solvent: $CDCl_3$)

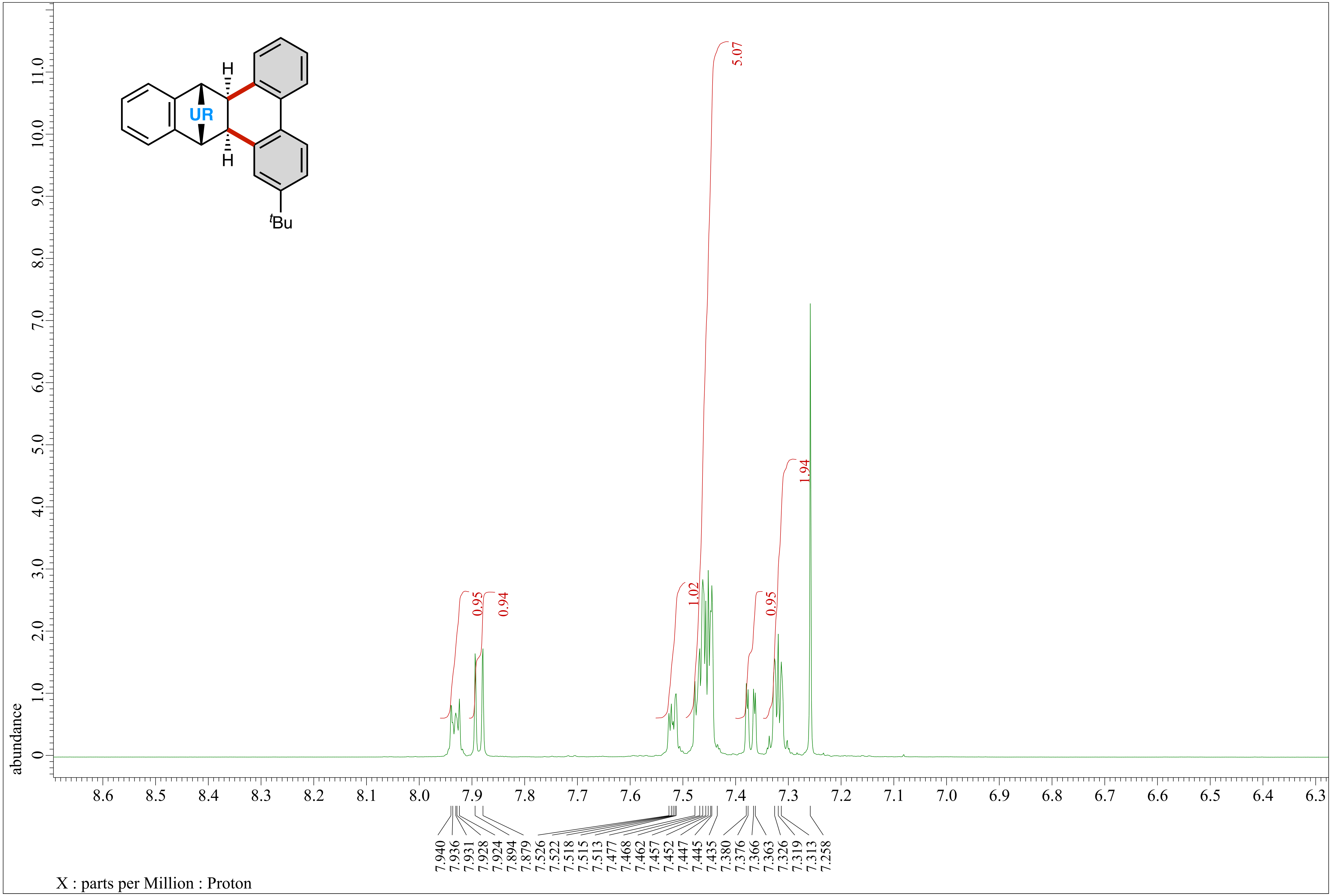

^{13}C NMR spectrum of **4gb** (solvent: $CDCl_3$)

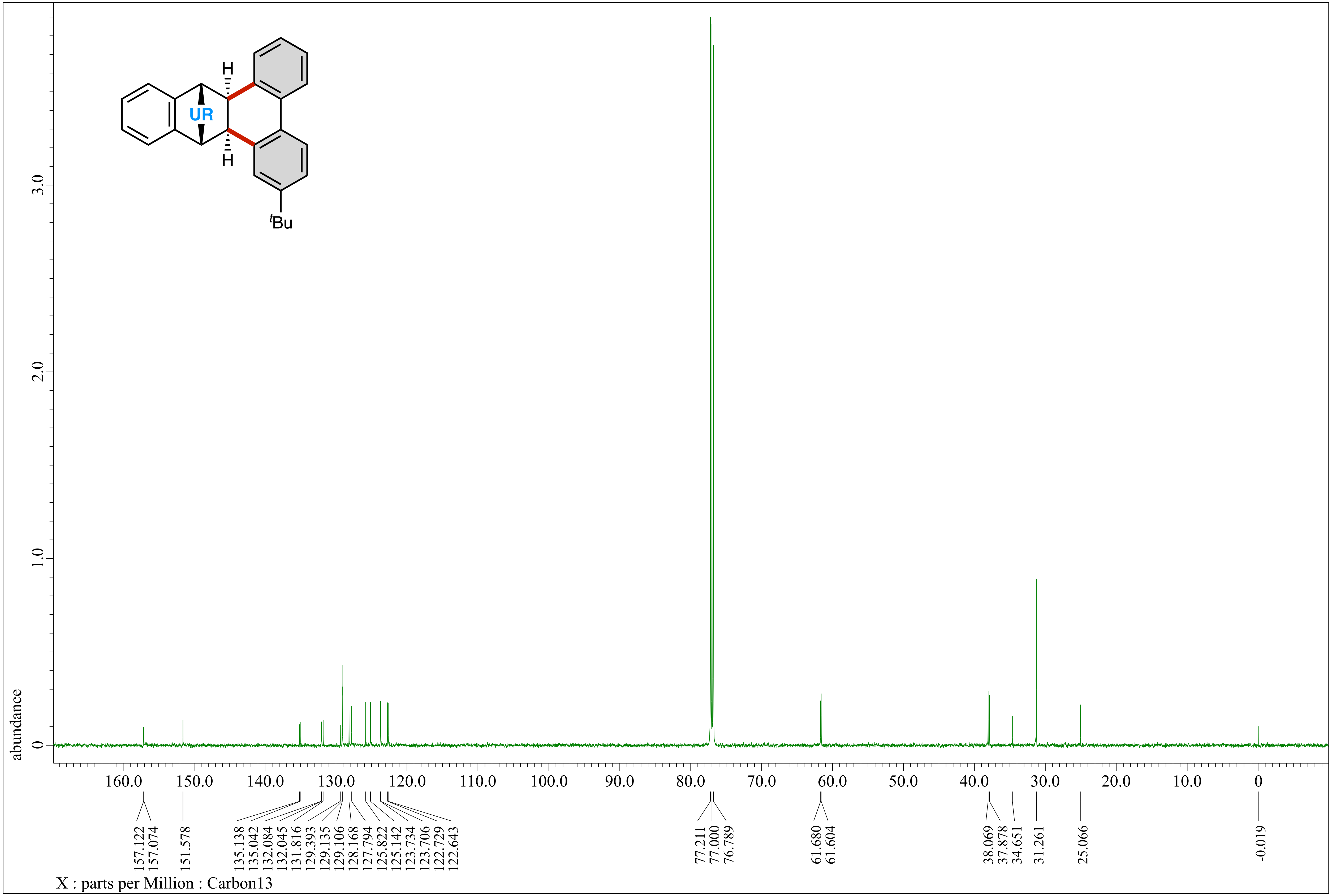

^{13}C NMR spectrum (inset) of **4gb** (solvent: $CDCl_3$)

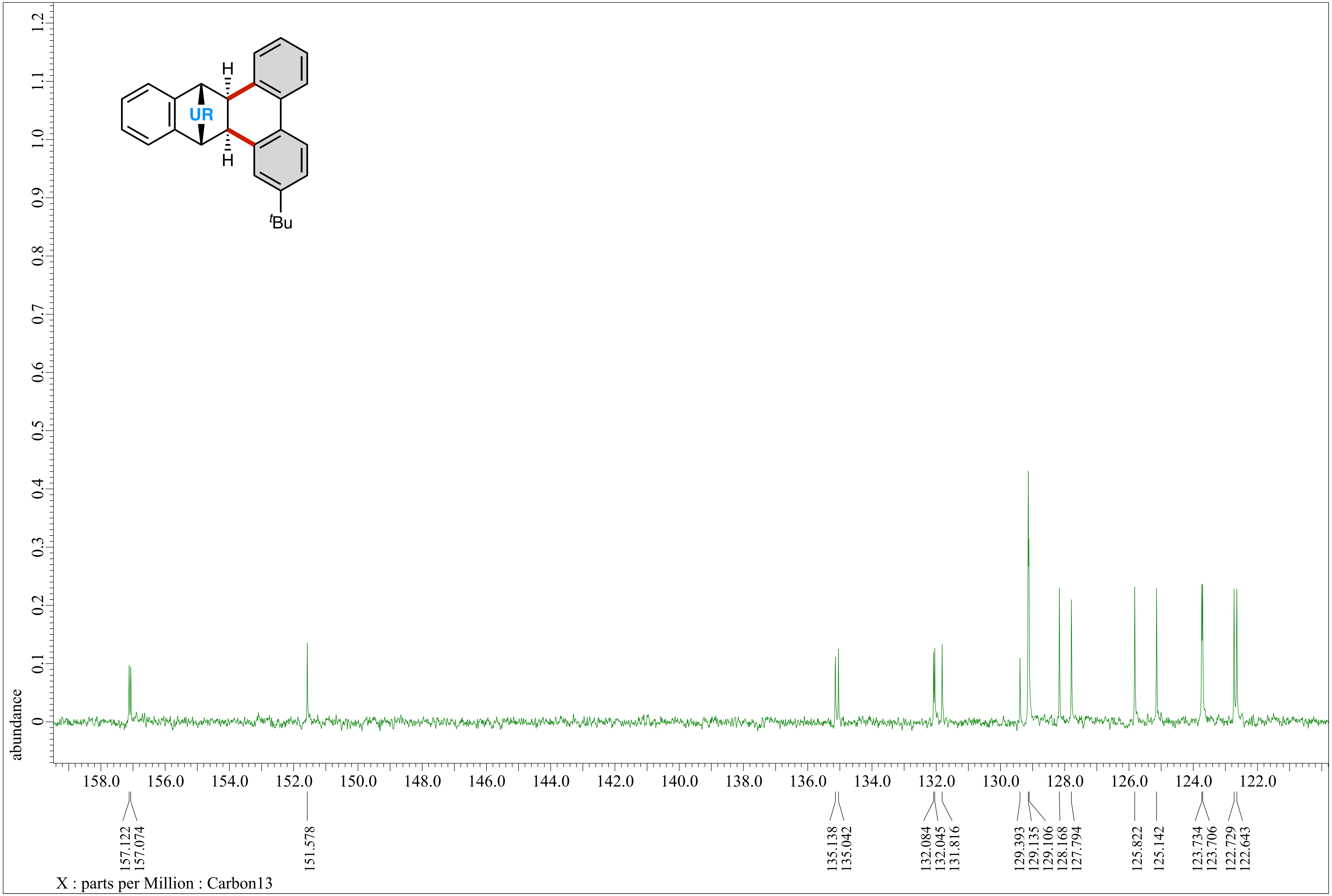

^{1}H NMR spectrum of **4gc** (solvent: $CDCl_3$)

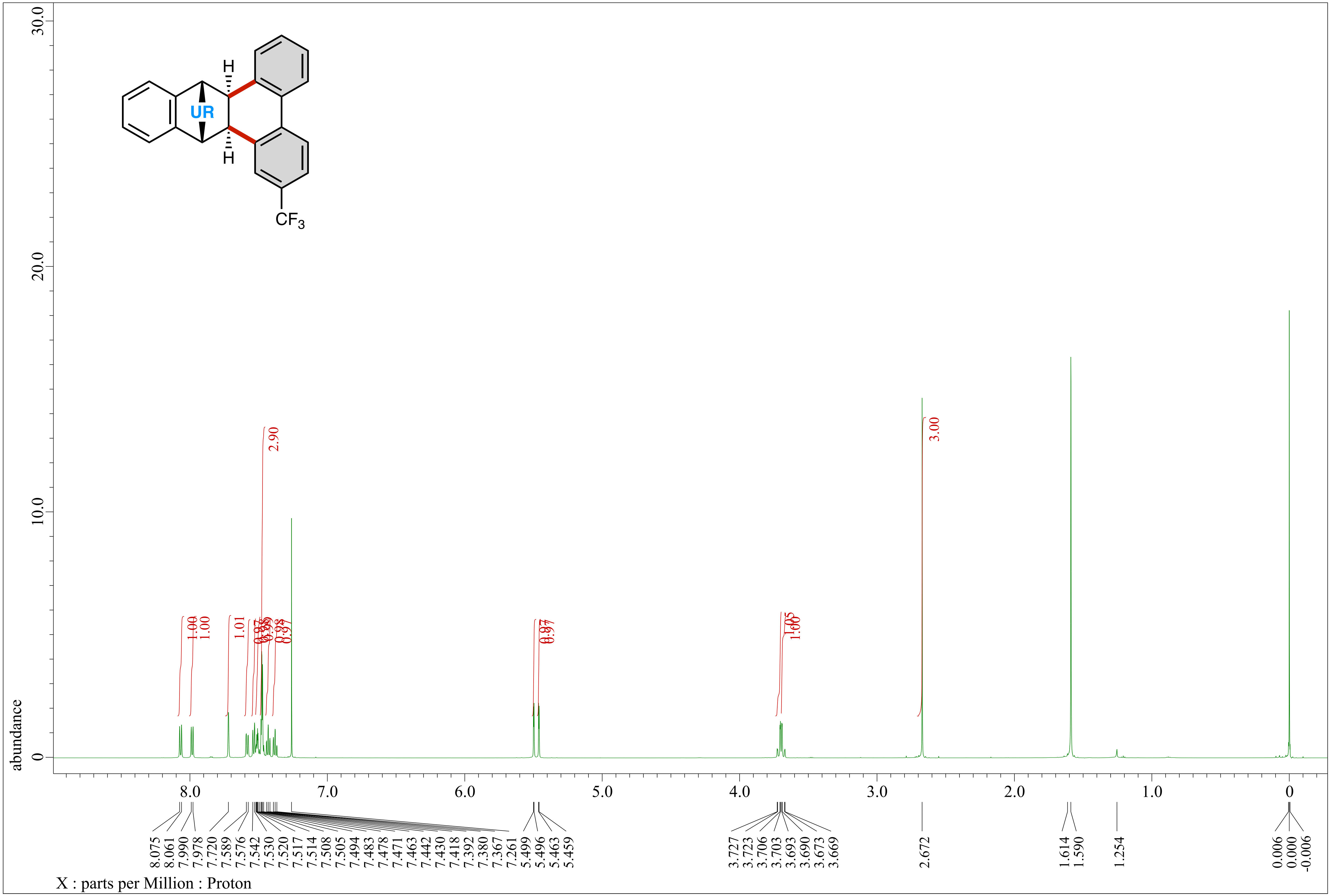

^{1}H NMR spectrum (inset) of **4gc** (solvent: $CDCl_3$)

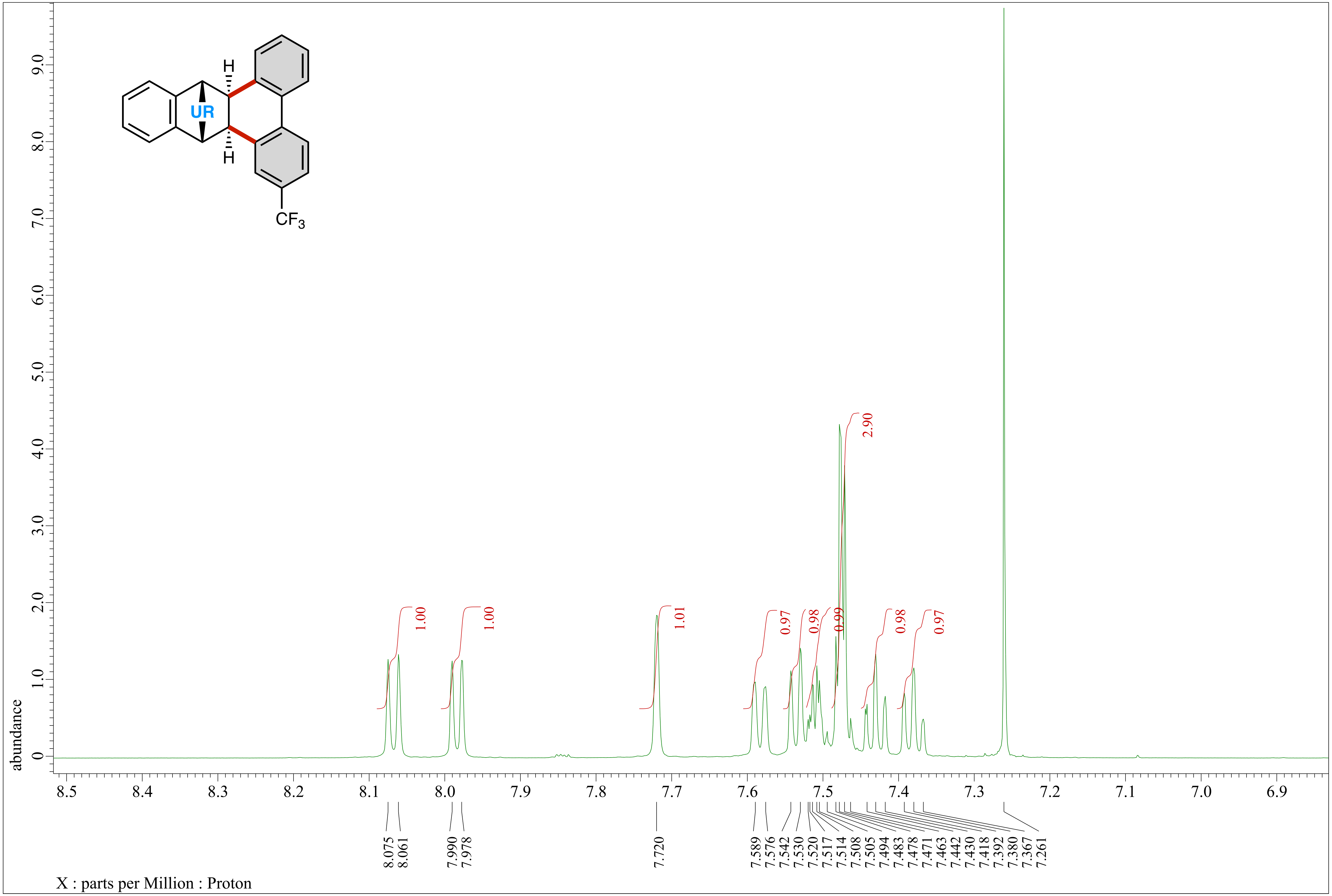

^{13}C NMR spectrum of **4gc** (solvent: $CDCl_3$)

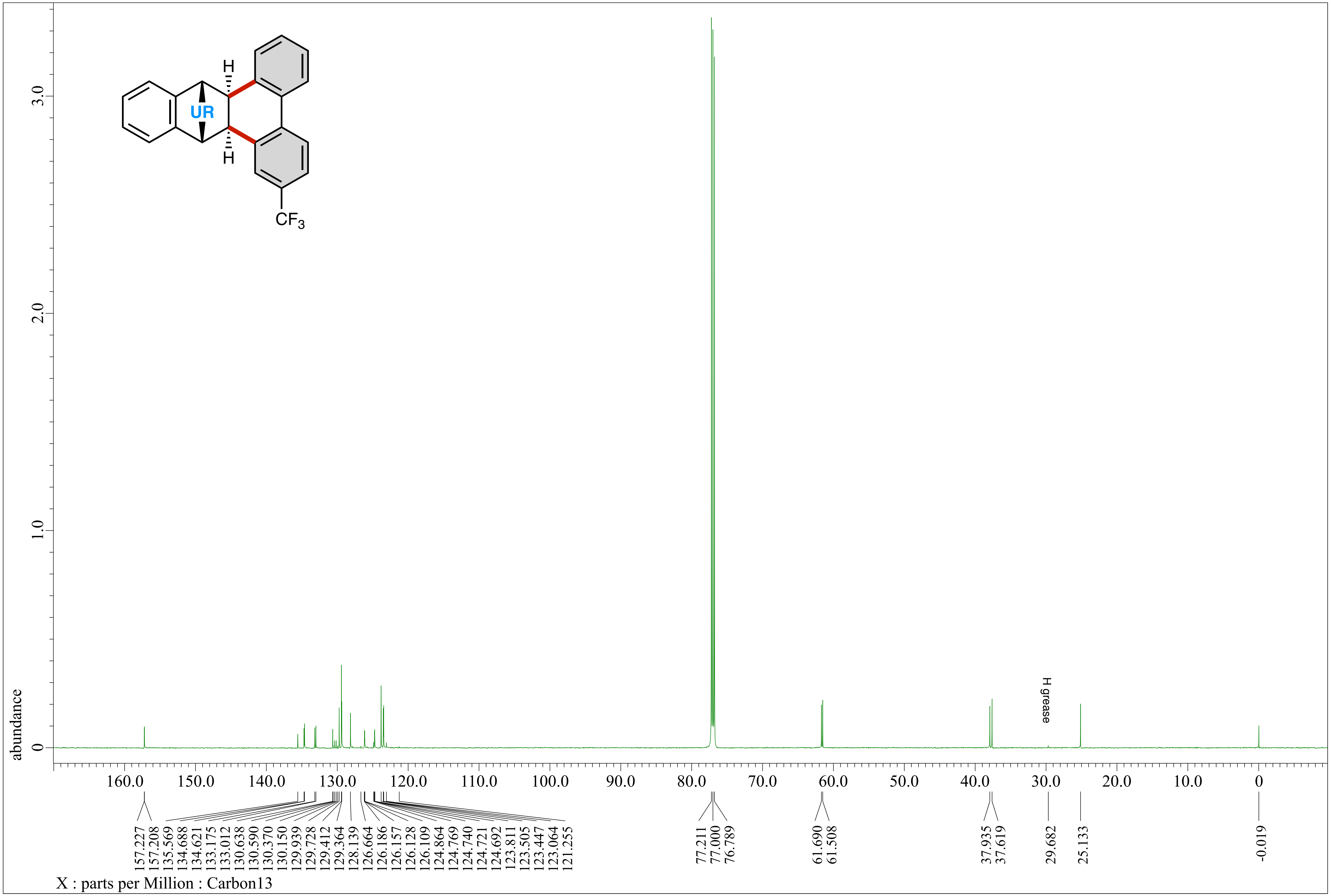

^{13}C NMR spectrum (inset) of **4gc** (solvent: $CDCl_3$)

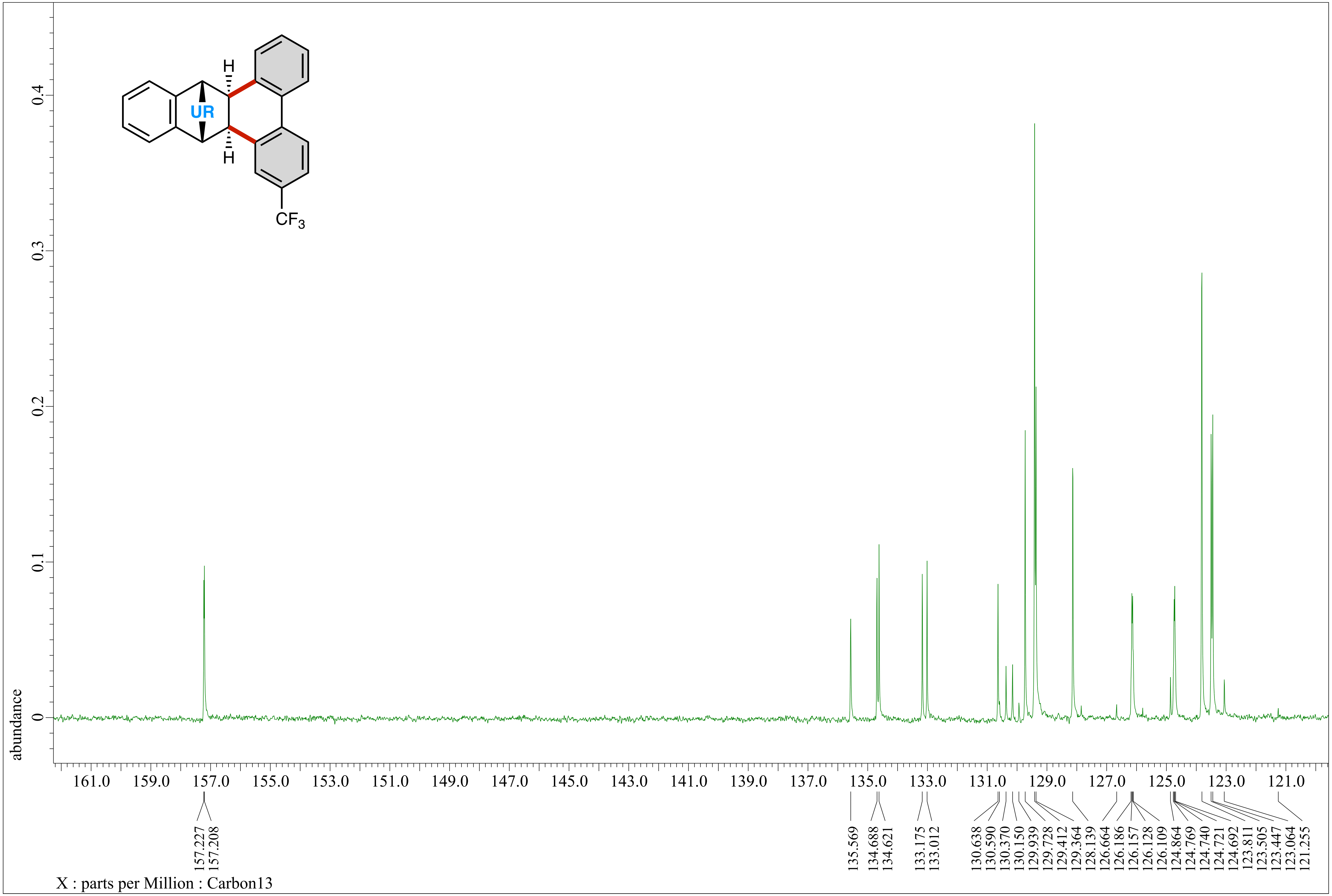

^{1}H NMR spectrum of **4gd** (solvent: $CDCl_3$)

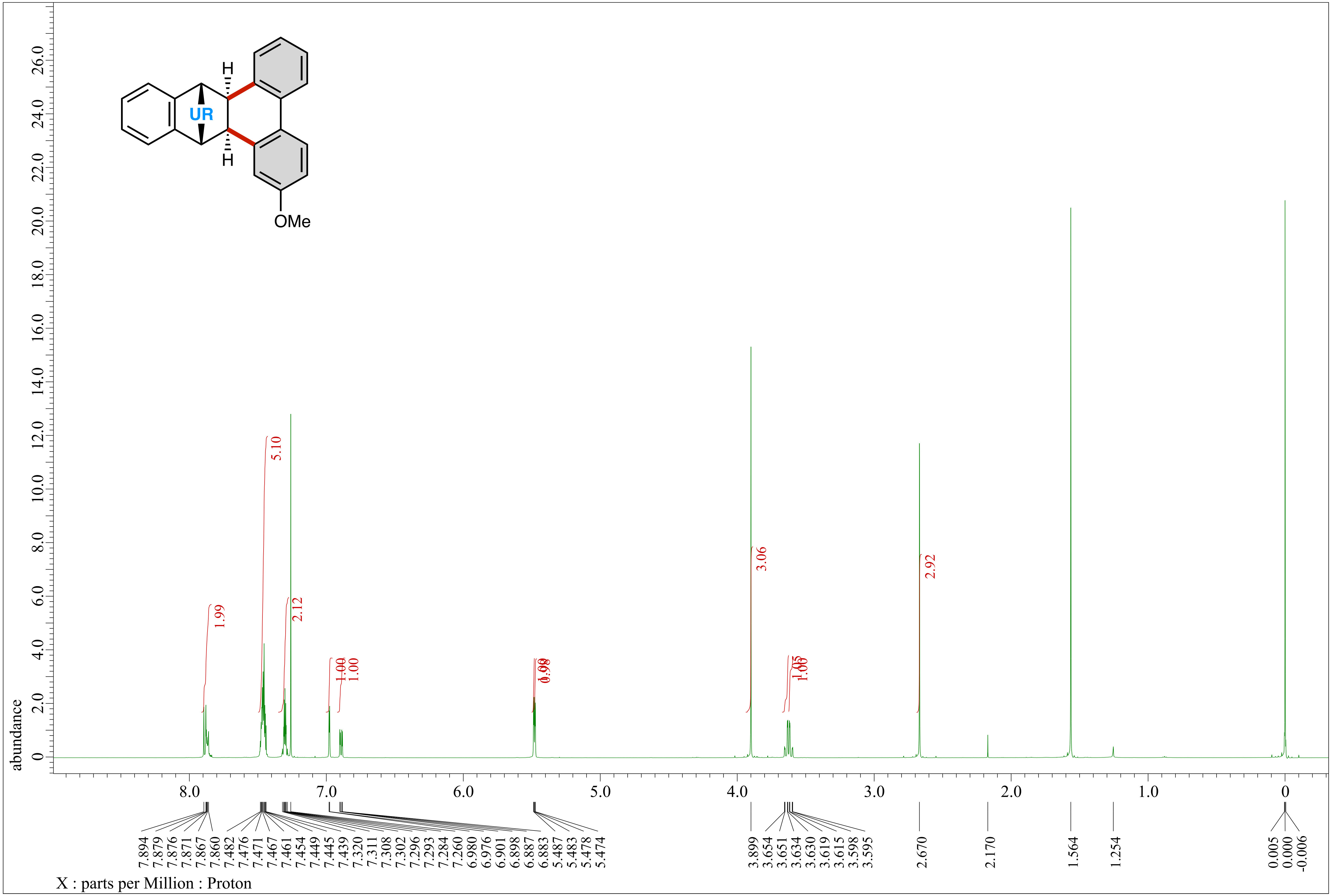

^{1}H NMR spectrum (inset) of **4gd** (solvent: $CDCl_3$)

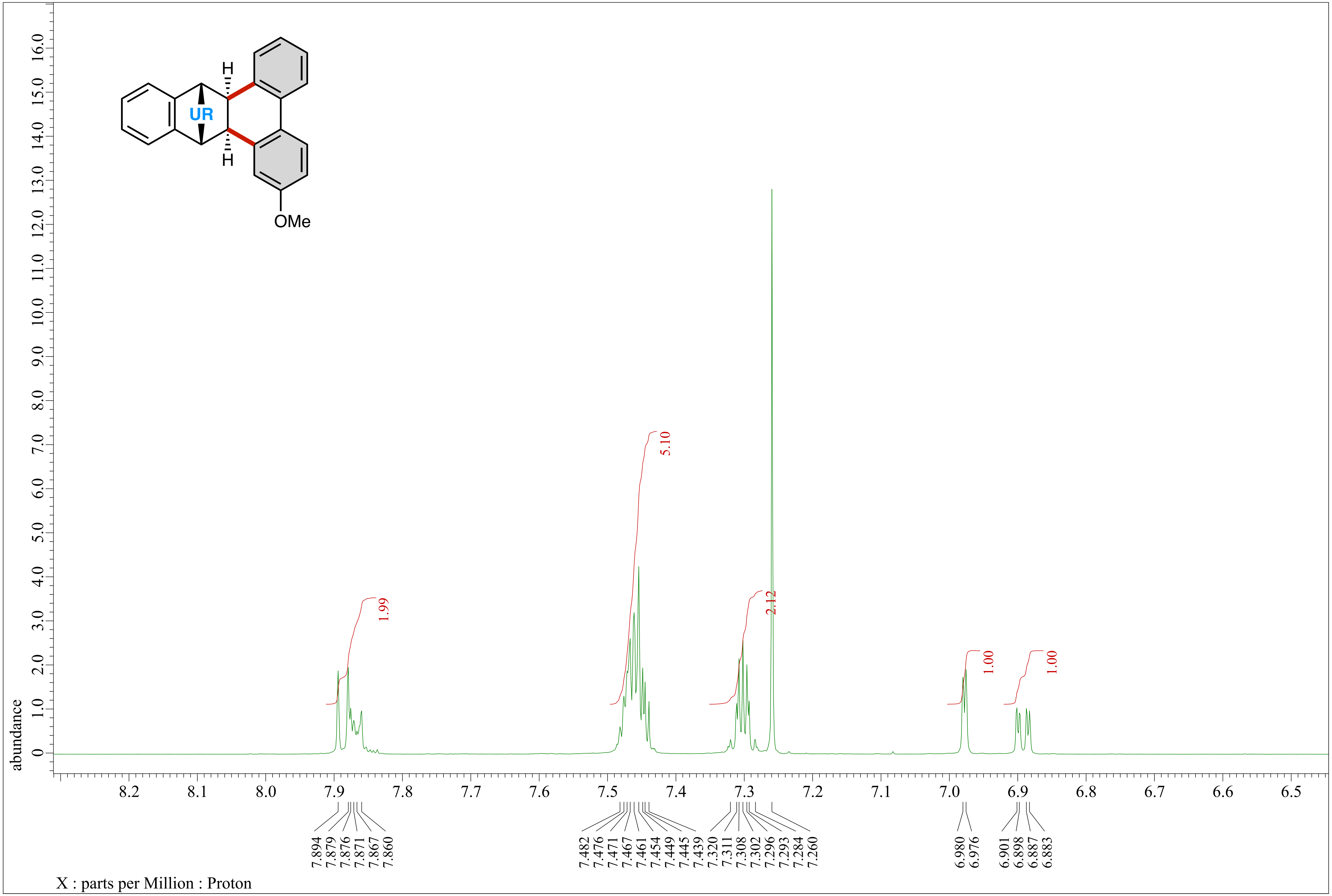

^{13}C NMR spectrum of **4gd** (solvent: $CDCl_3$)

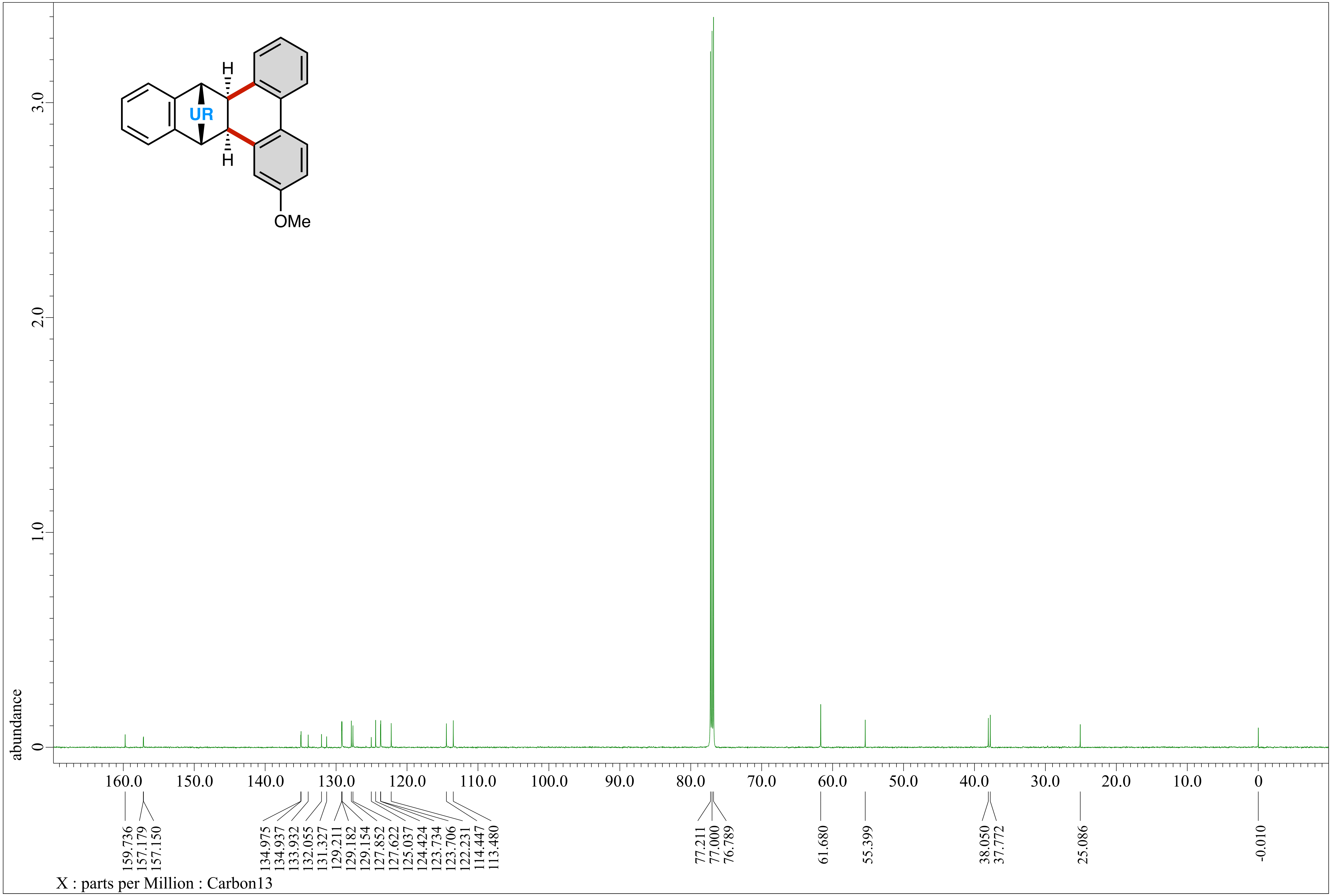

^{13}C NMR spectrum (inset) of **4gd** (solvent: $CDCl_3$)

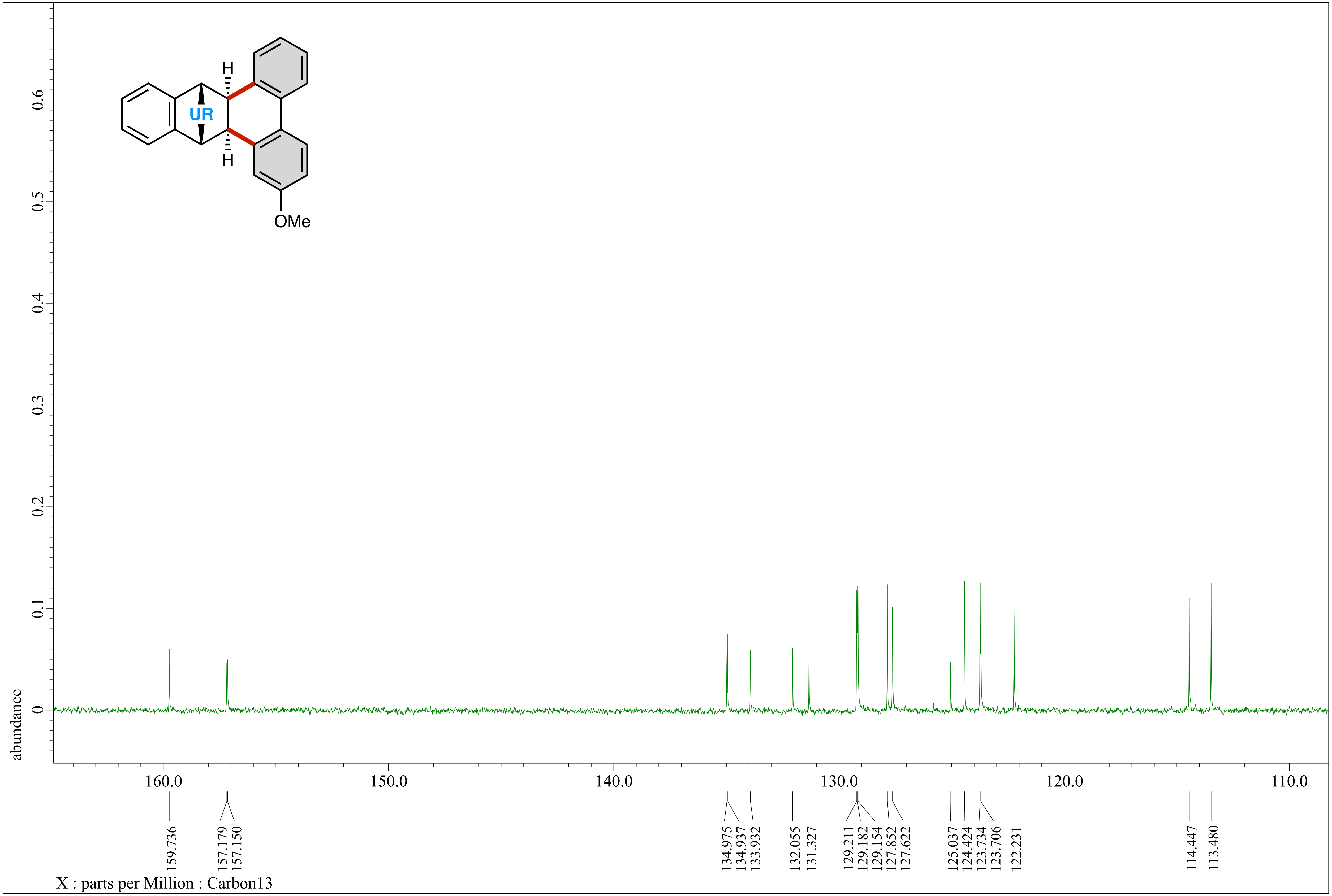

^{1}H NMR spectrum of **4ge** (solvent: $CDCl_3$)

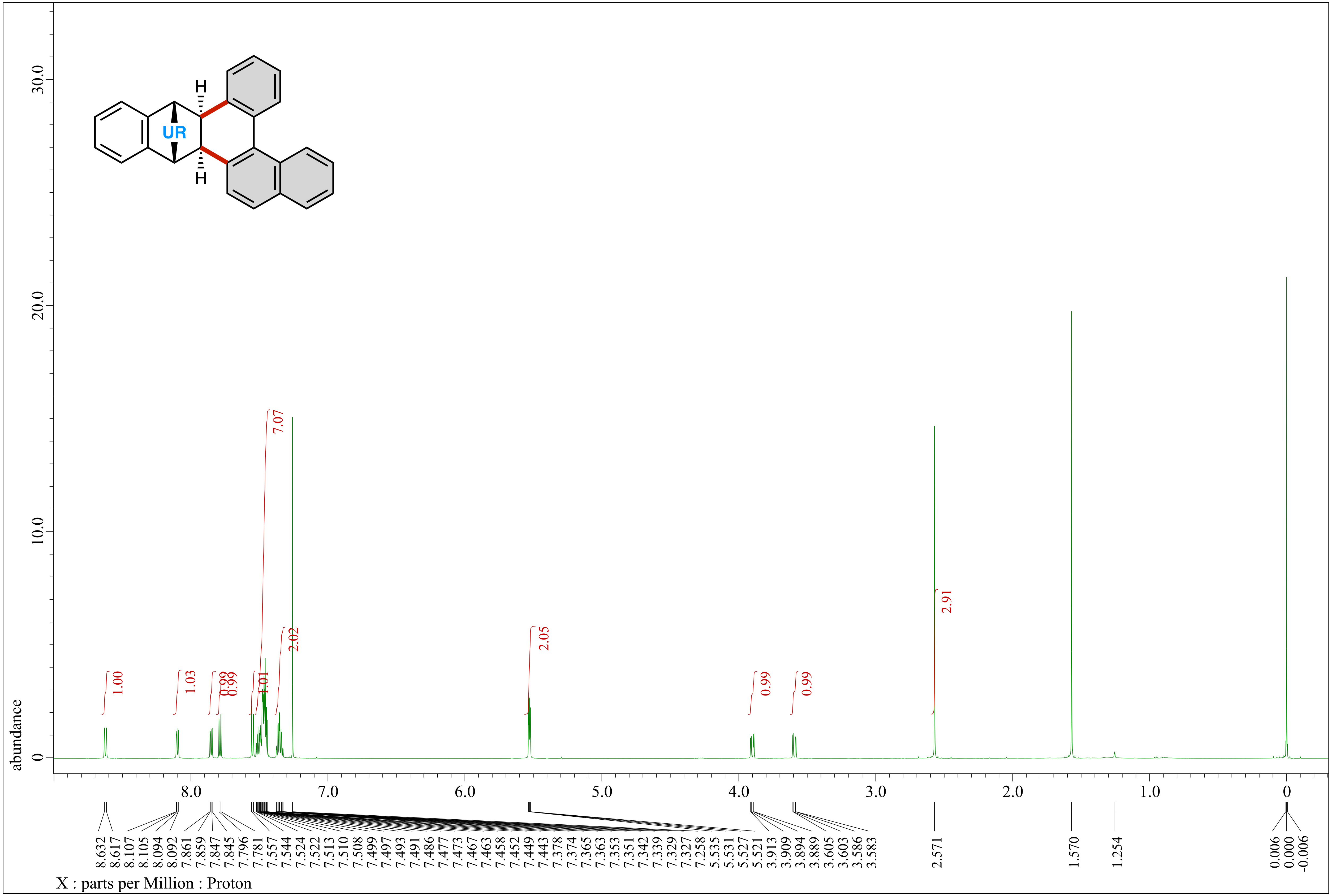

^{1}H NMR spectrum (inset) of **4ge** (solvent: $CDCl_3$)

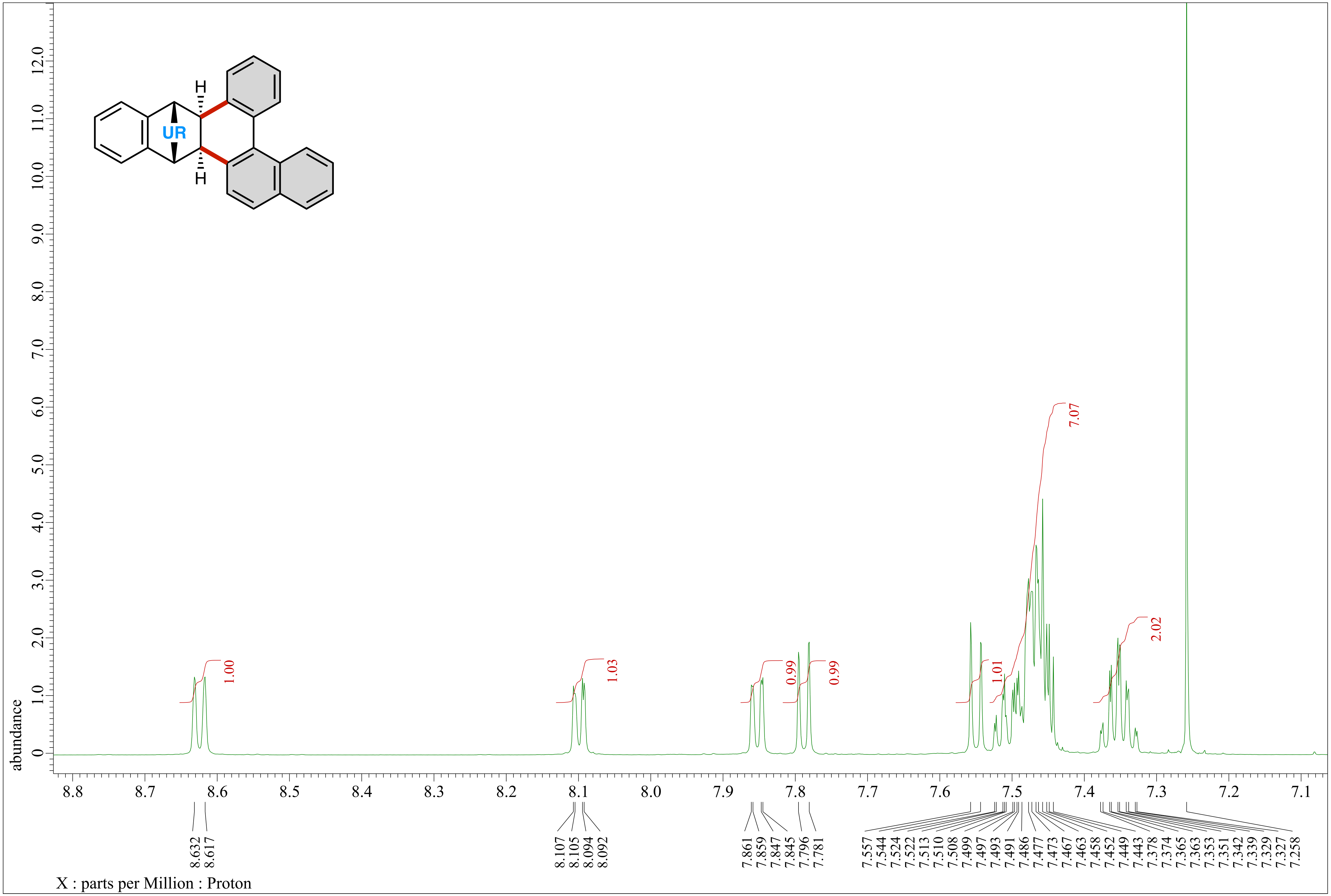

^{13}C NMR spectrum of **4ge** (solvent: $CDCl_3$)

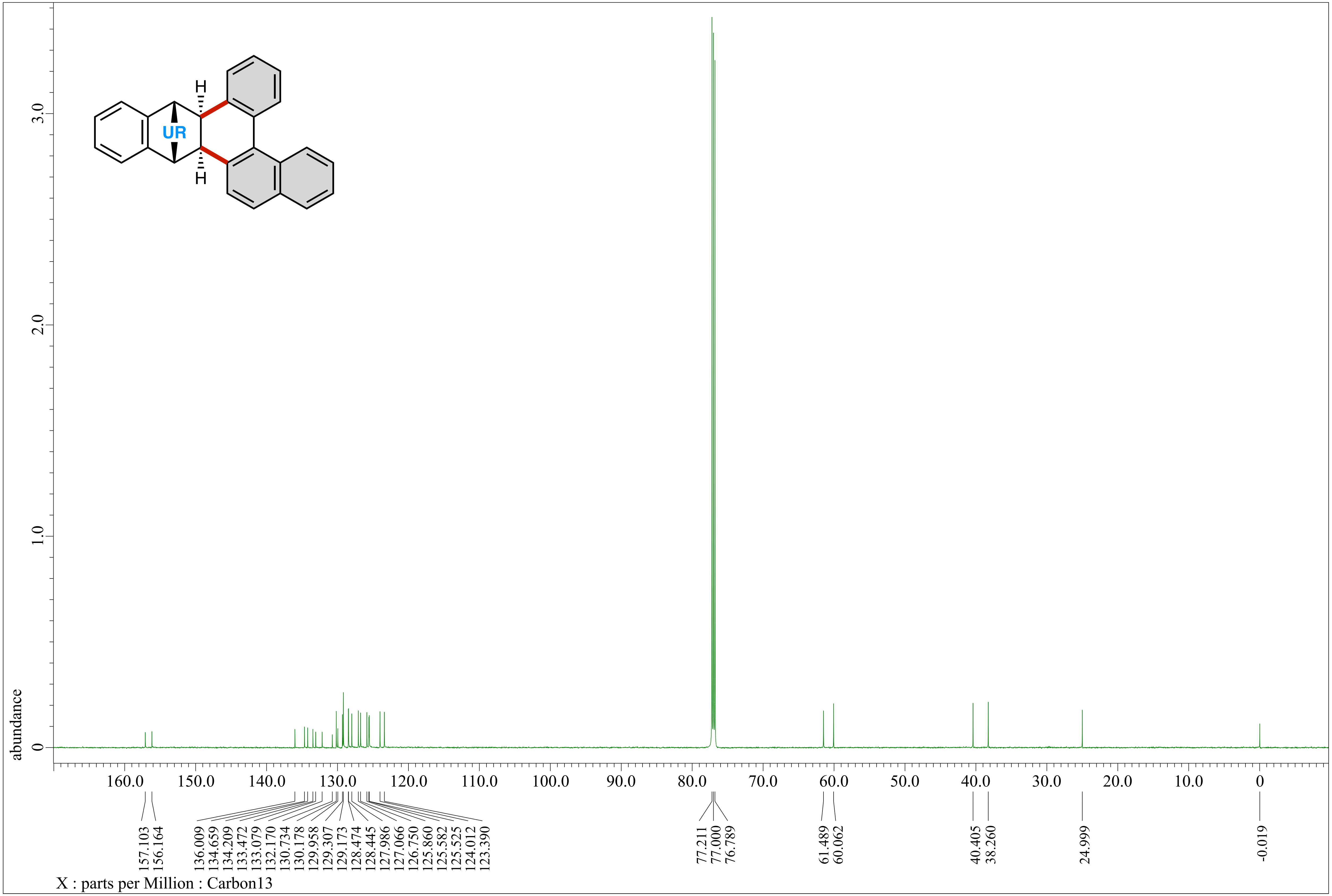

^{13}C NMR spectrum (inset) of **4ge** (solvent: $CDCl_3$)

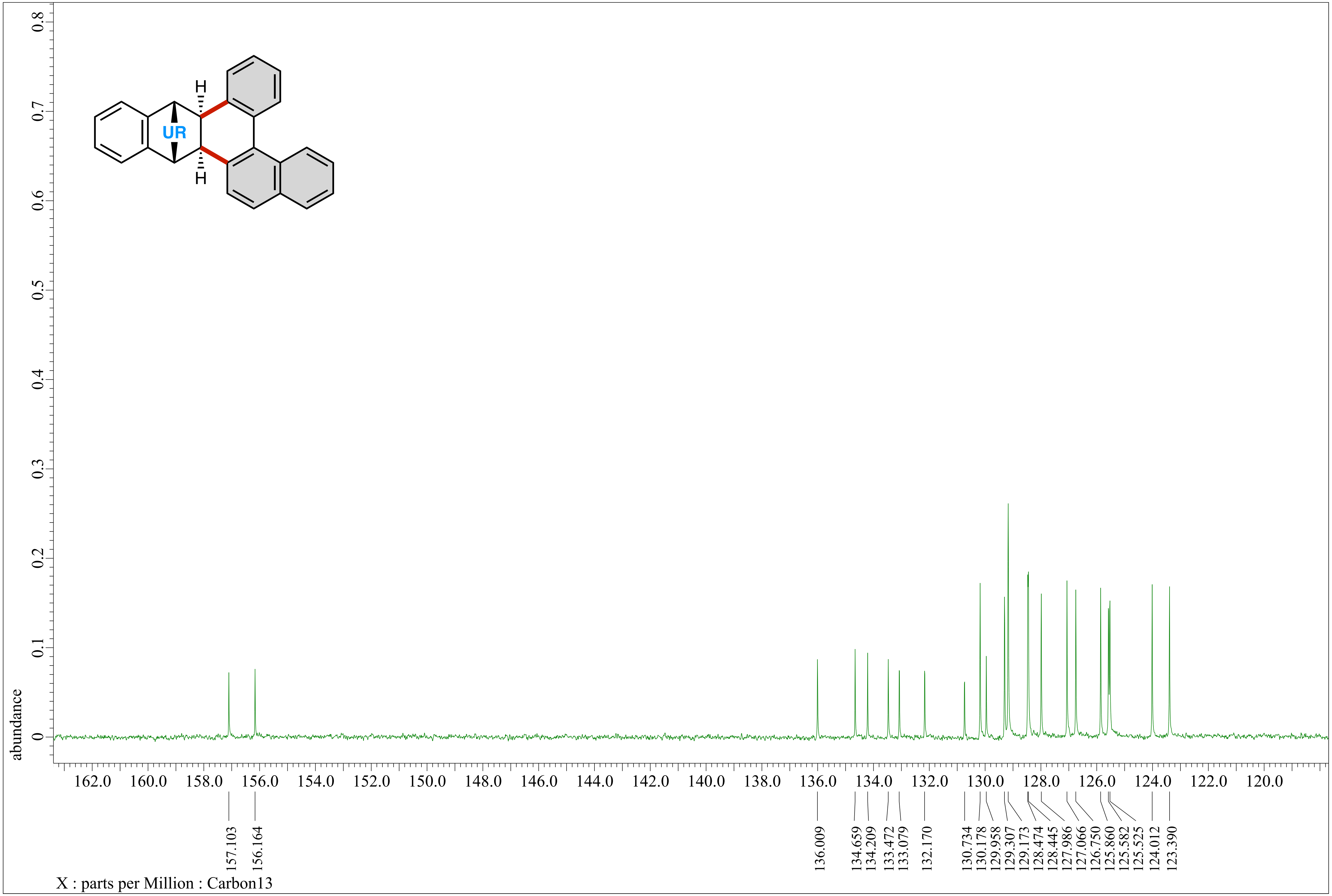

^{1}H NMR spectrum of **4gf** (solvent: $CDCl_3$)

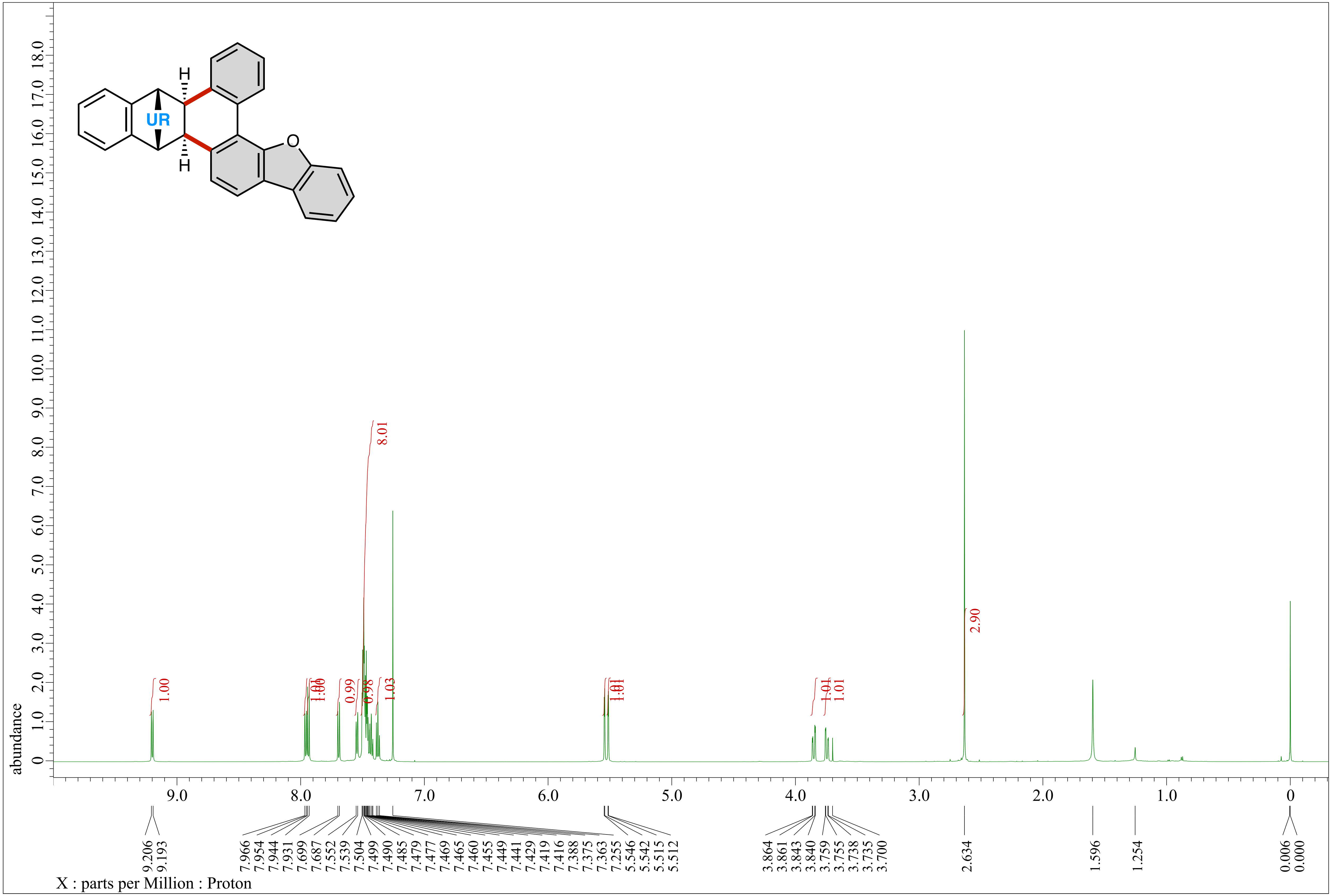

^{1}H NMR spectrum (inset) of **4gf** (solvent: $CDCl_3$)

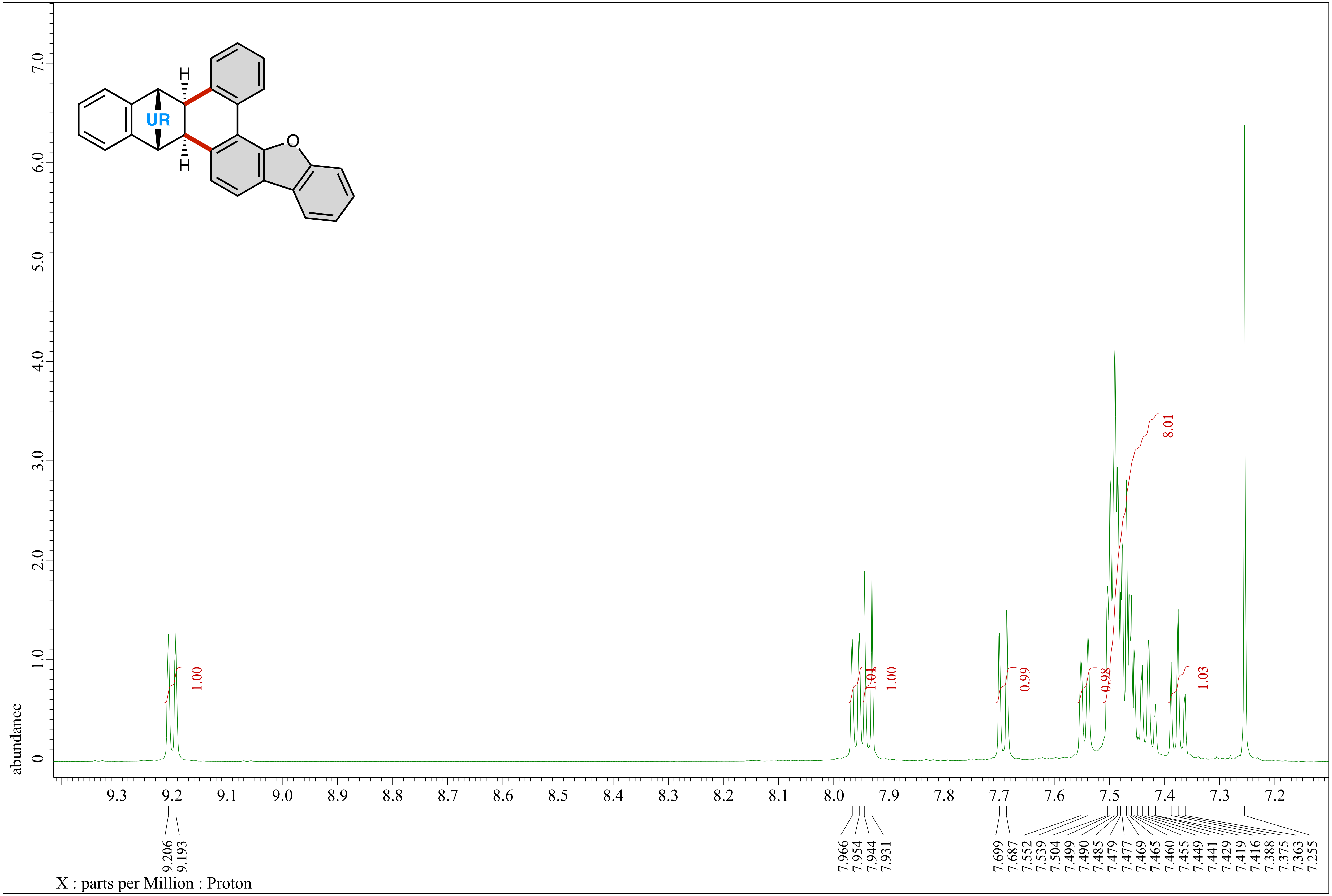

^{13}C NMR spectrum of **4gf** (solvent: $CDCl_3$)

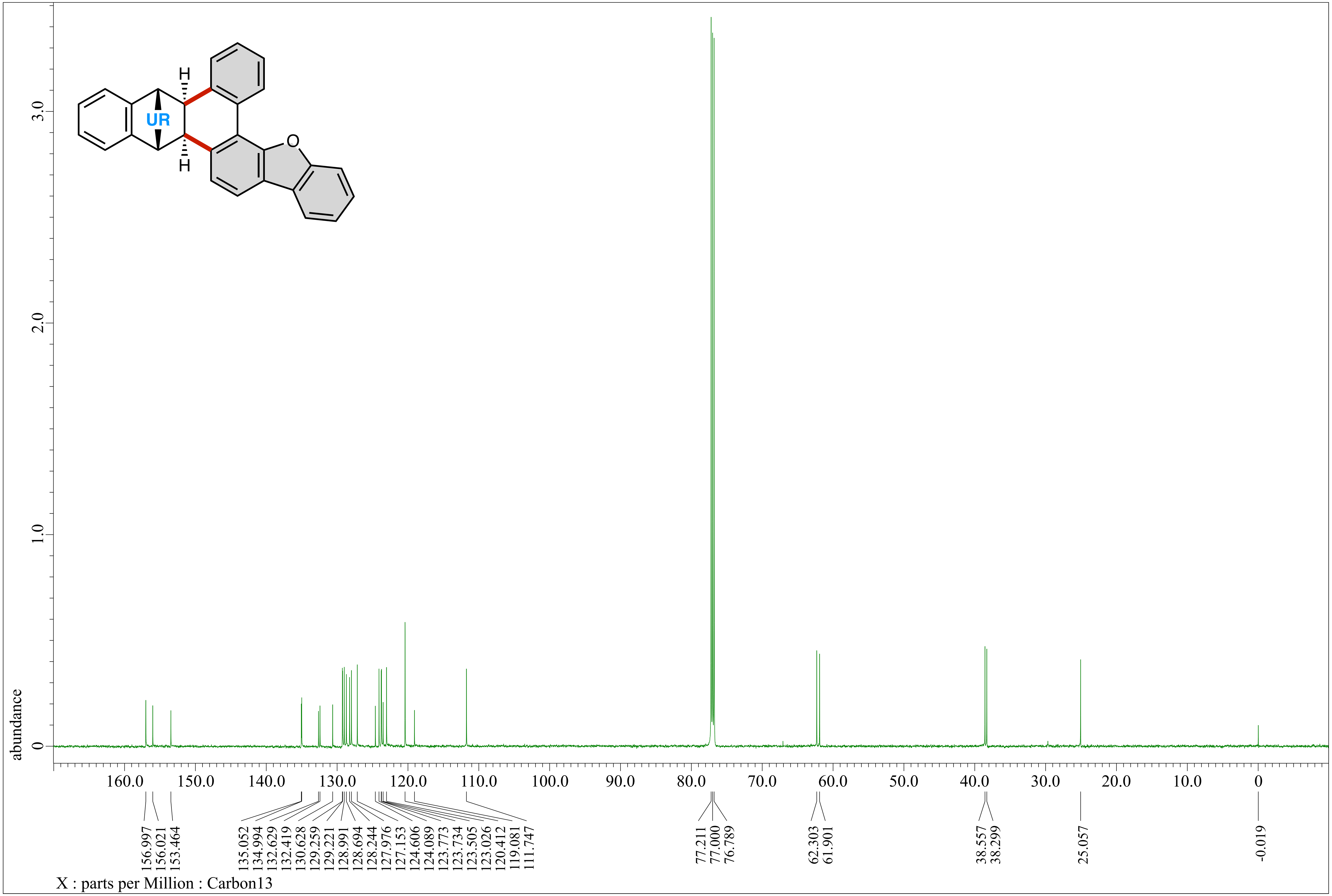

^{13}C NMR spectrum (inset) of **4gf** (solvent: $CDCl_3$)

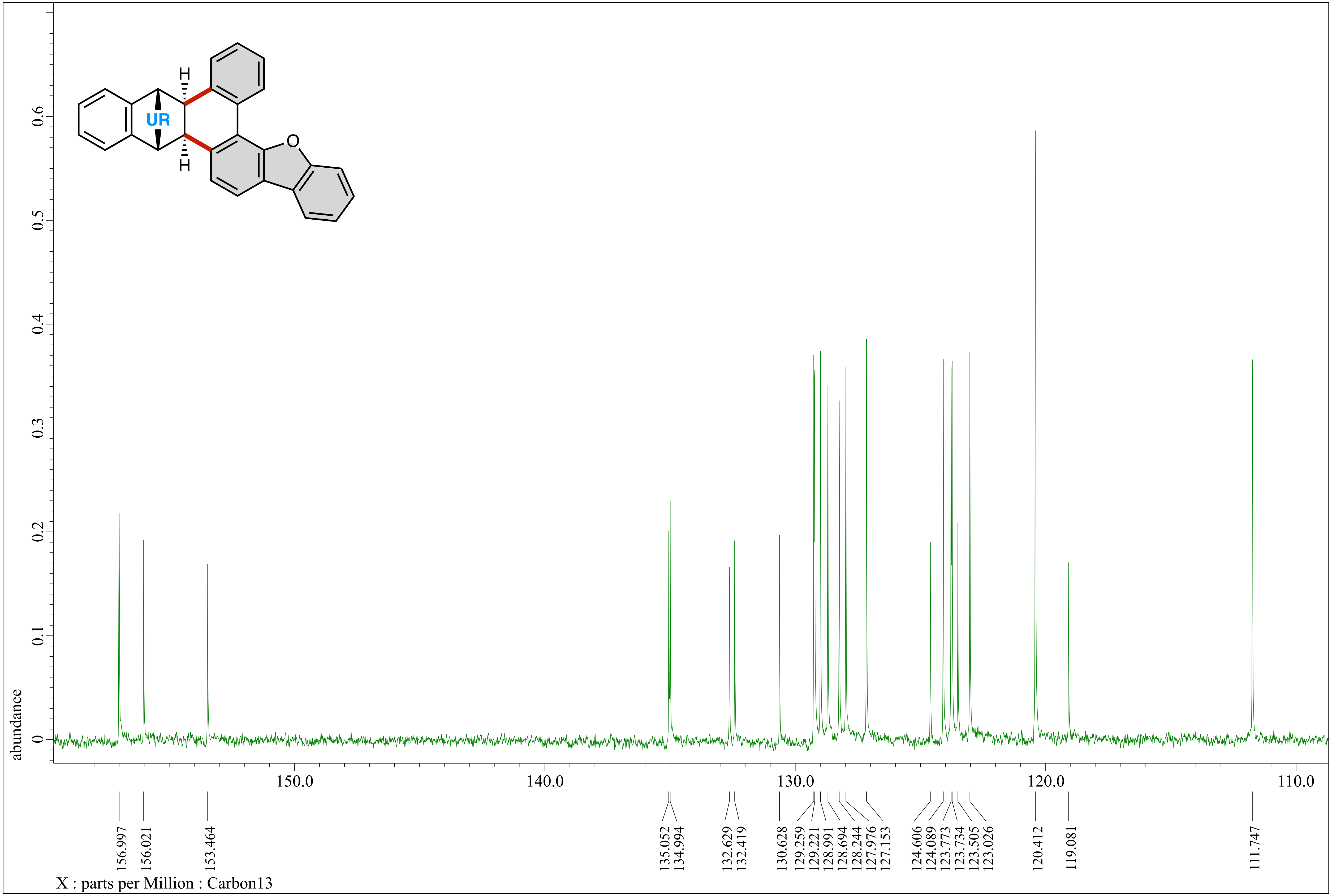

^{1}H NMR spectrum of **4gg** (solvent: $CDCl_3$)

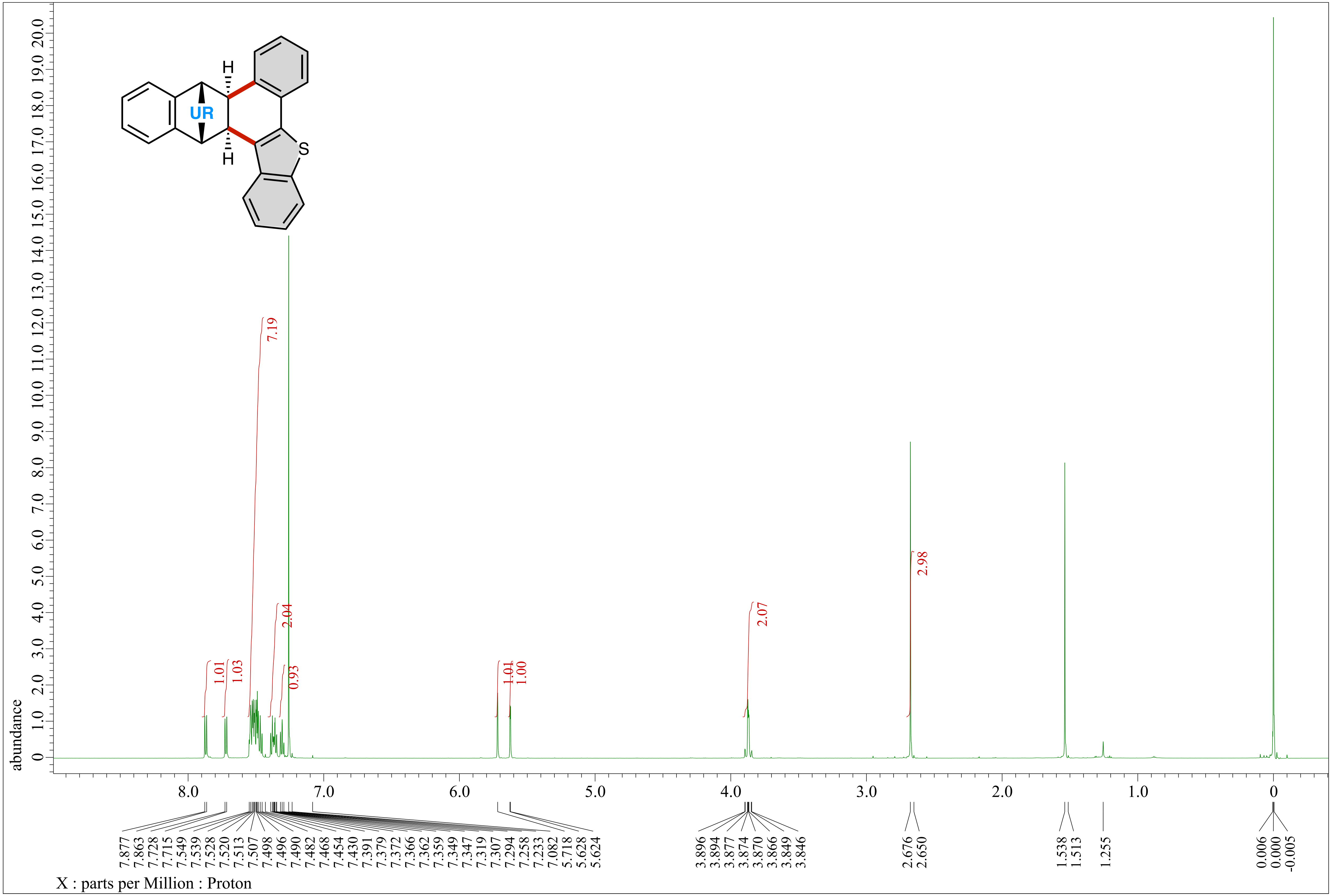

^{1}H NMR spectrum (inset) of **4gg** (solvent: $CDCl_3$)

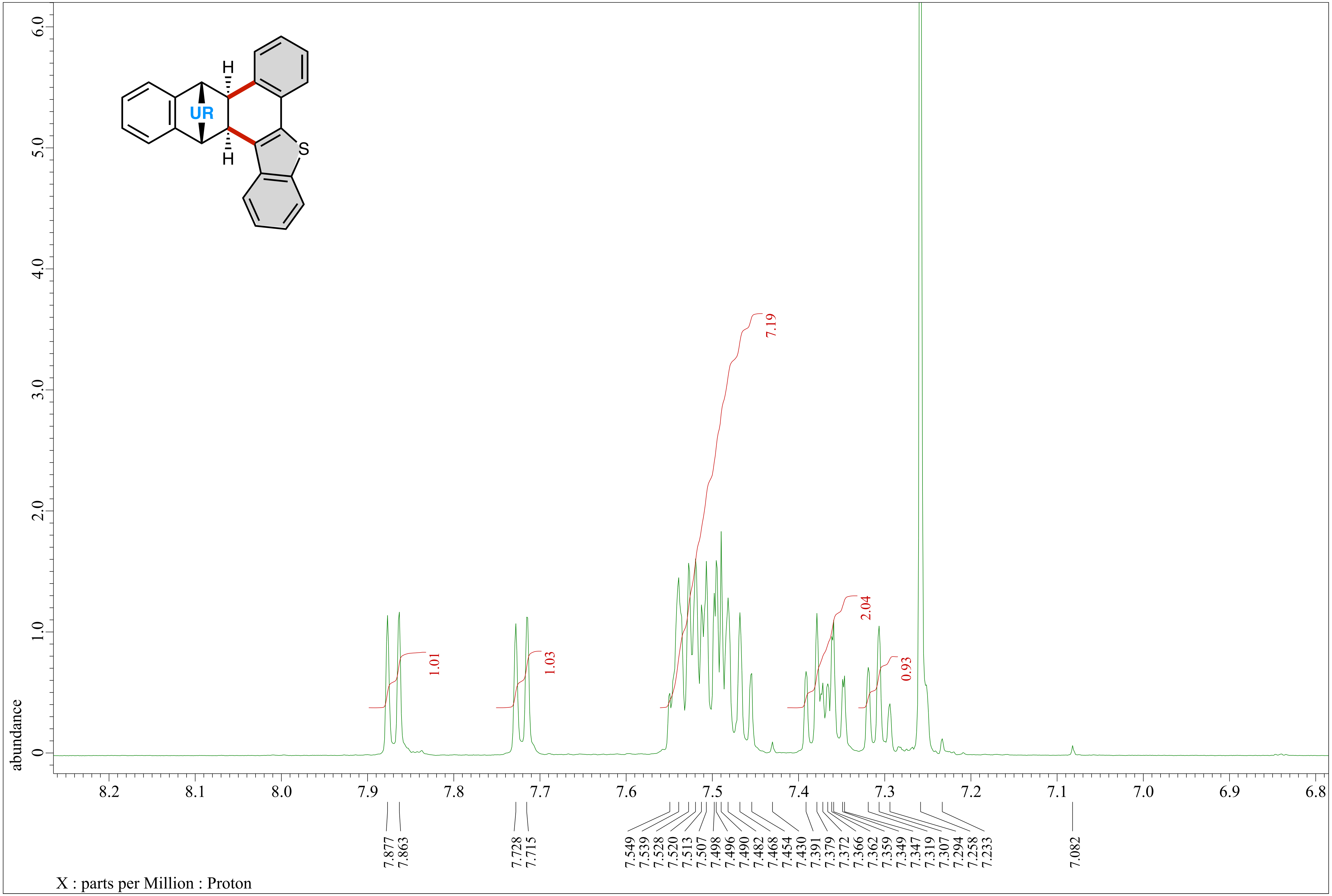

^{13}C NMR spectrum of **4gg** (solvent: $CDCl_3$)

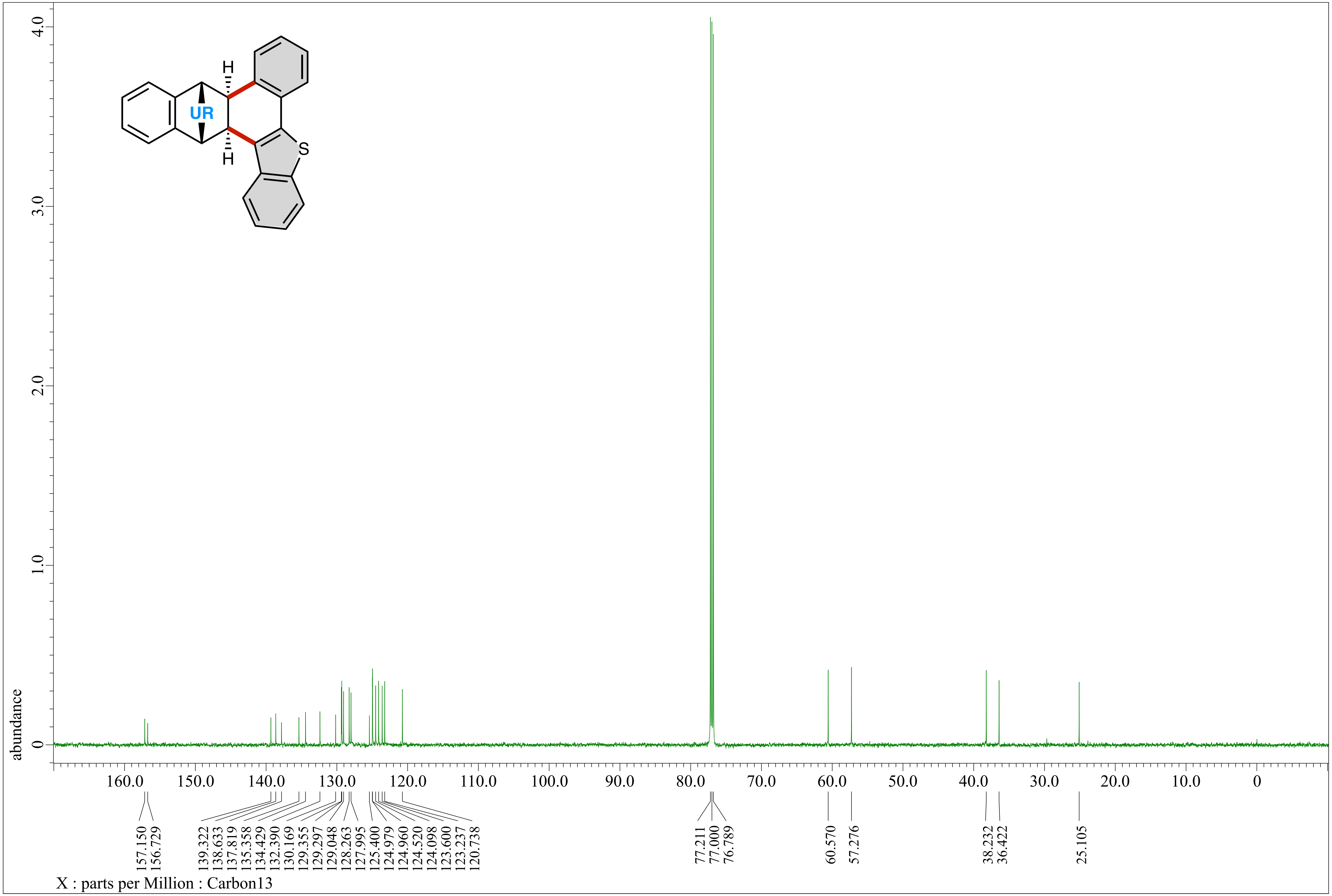

^{13}C NMR spectrum (inset) of **4gg** (solvent: $CDCl_3$)

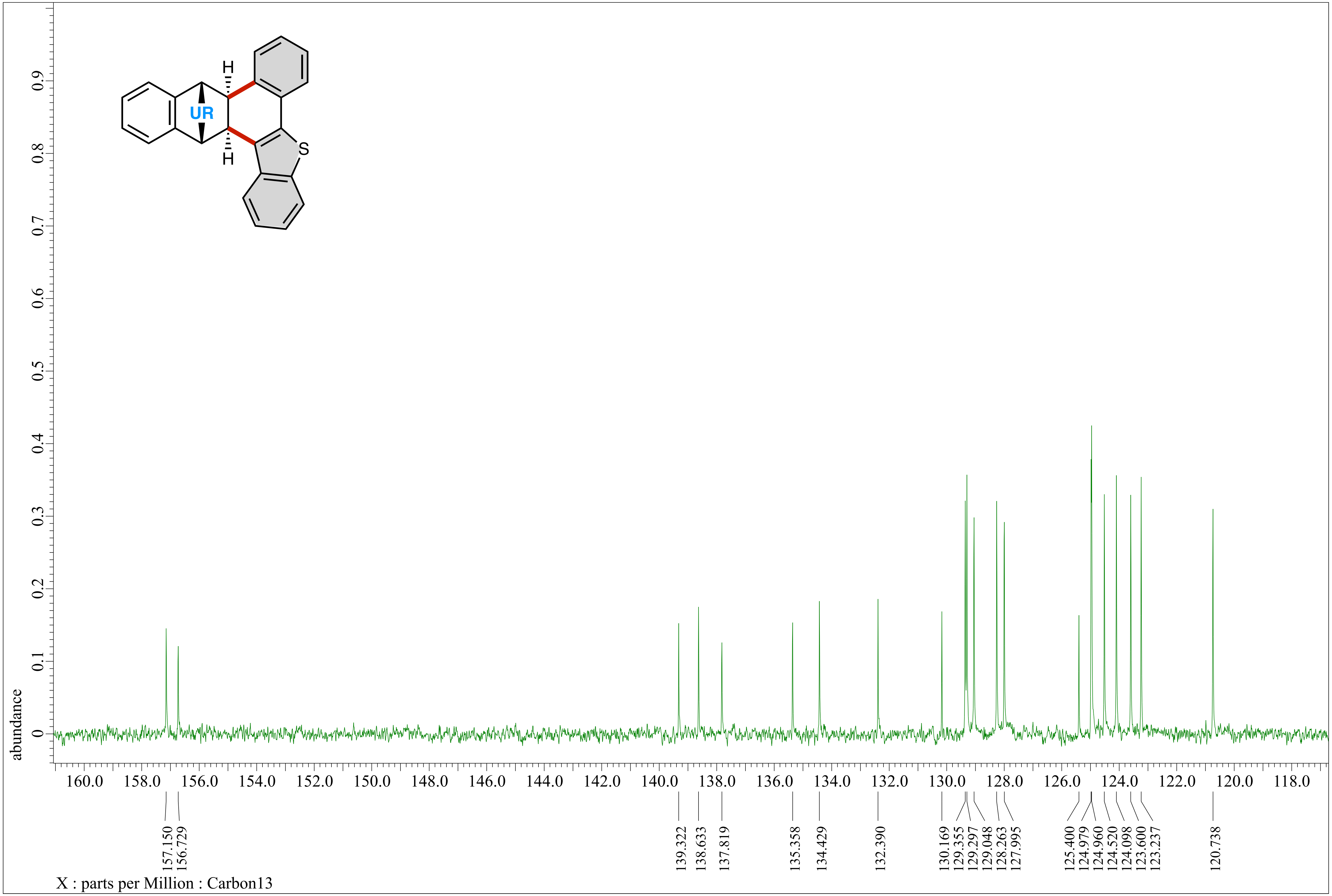

^{1}H NMR spectrum of **5aa** (solvent: $CDCl_3$)

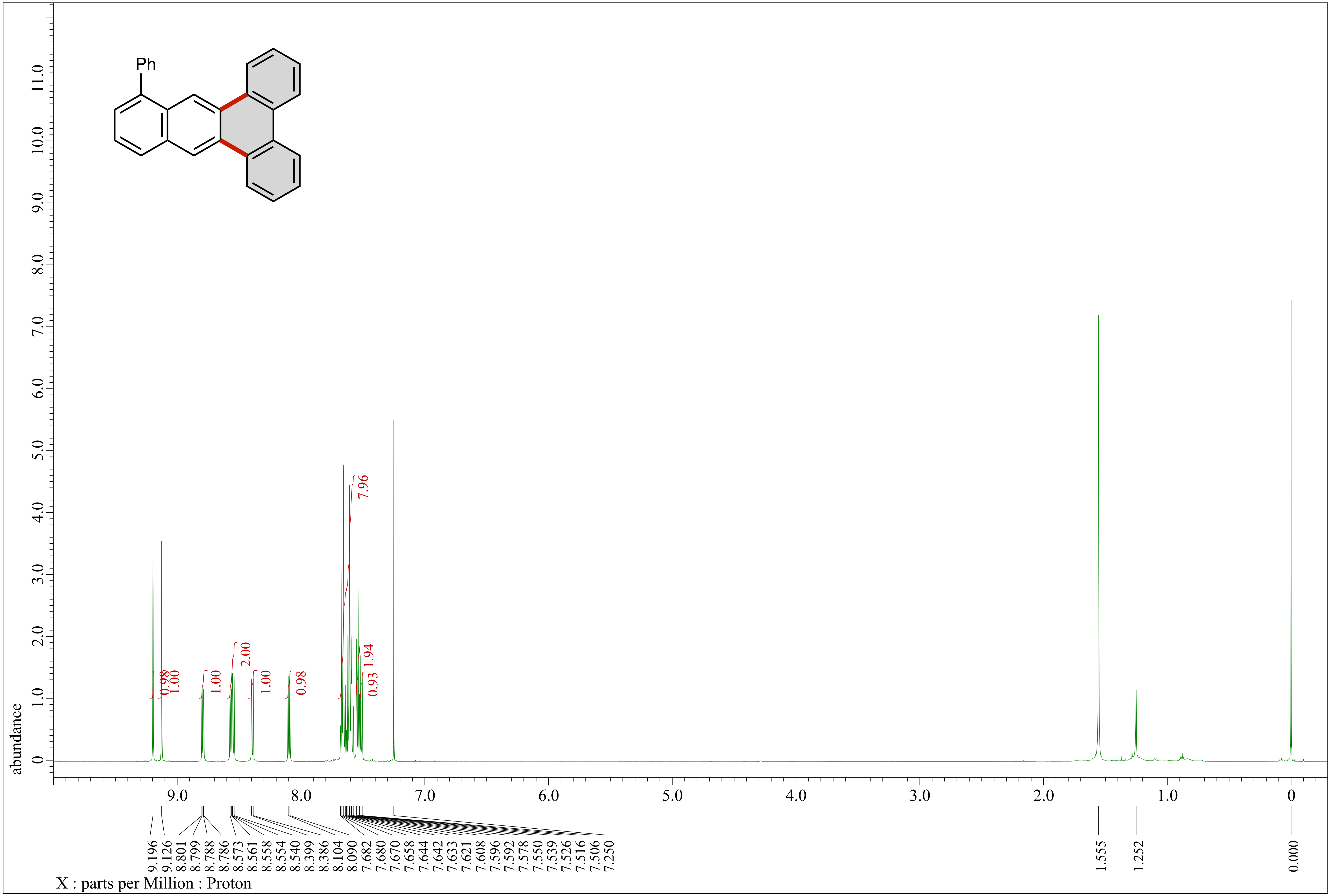

^{1}H NMR spectrum (inset) of **5aa** (solvent: $CDCl_3$)

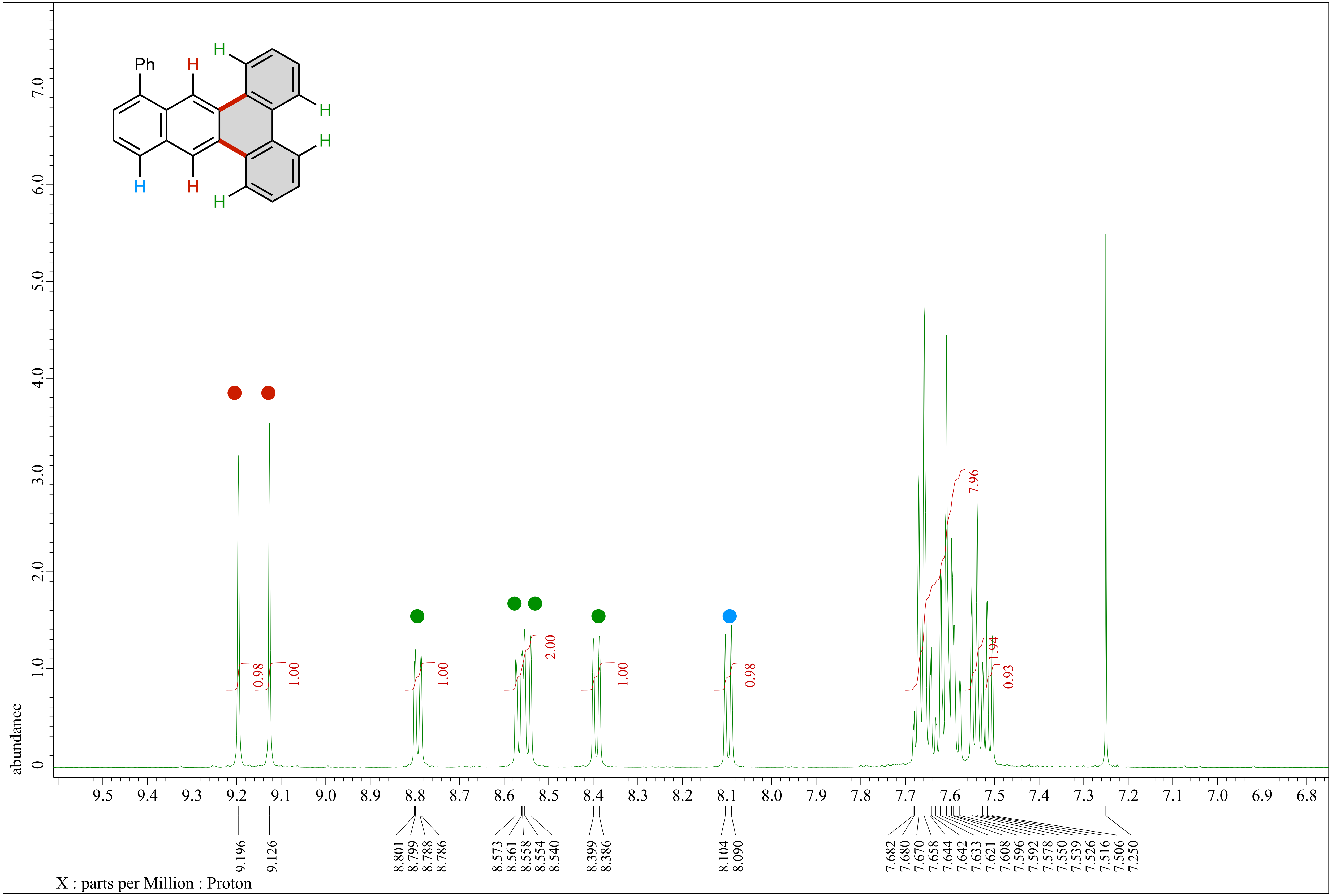

^{13}C NMR spectrum of **5aa** (solvent: $CDCl_3$)

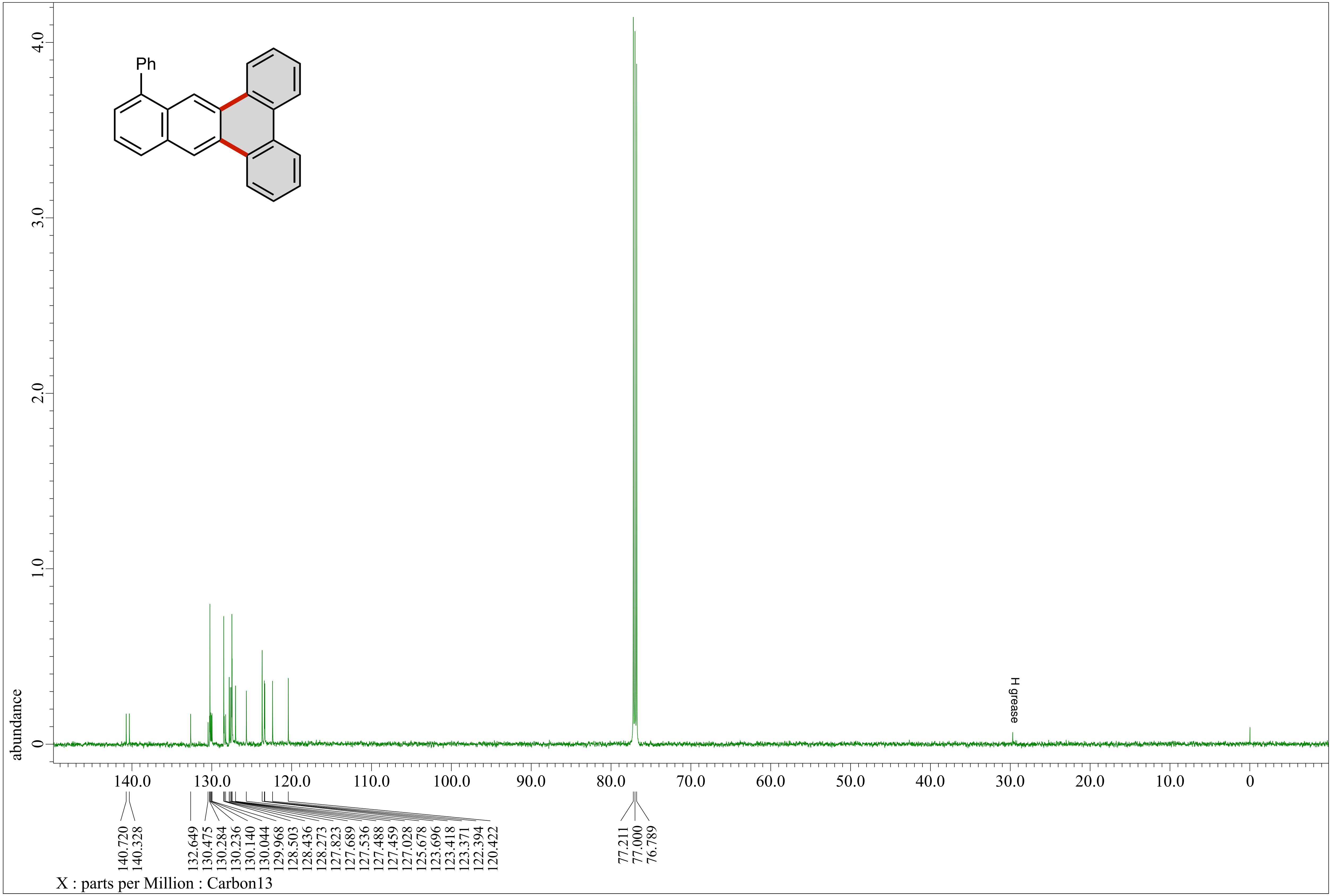

^{13}C NMR spectrum (inset) of **5aa** (solvent: $CDCl_3$)

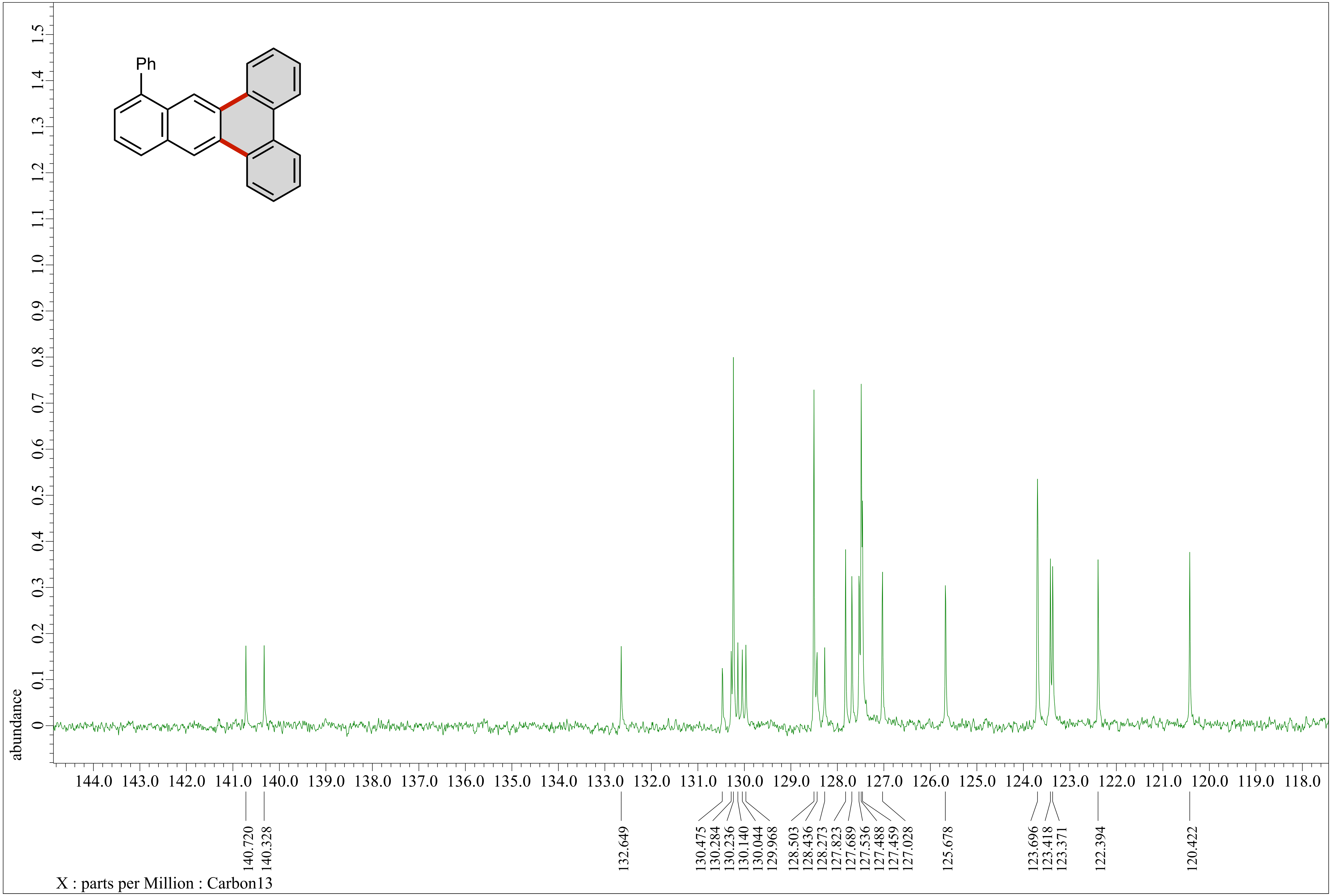

^{1}H NMR spectrum of **5ba** (solvent: $CDCl_3$)

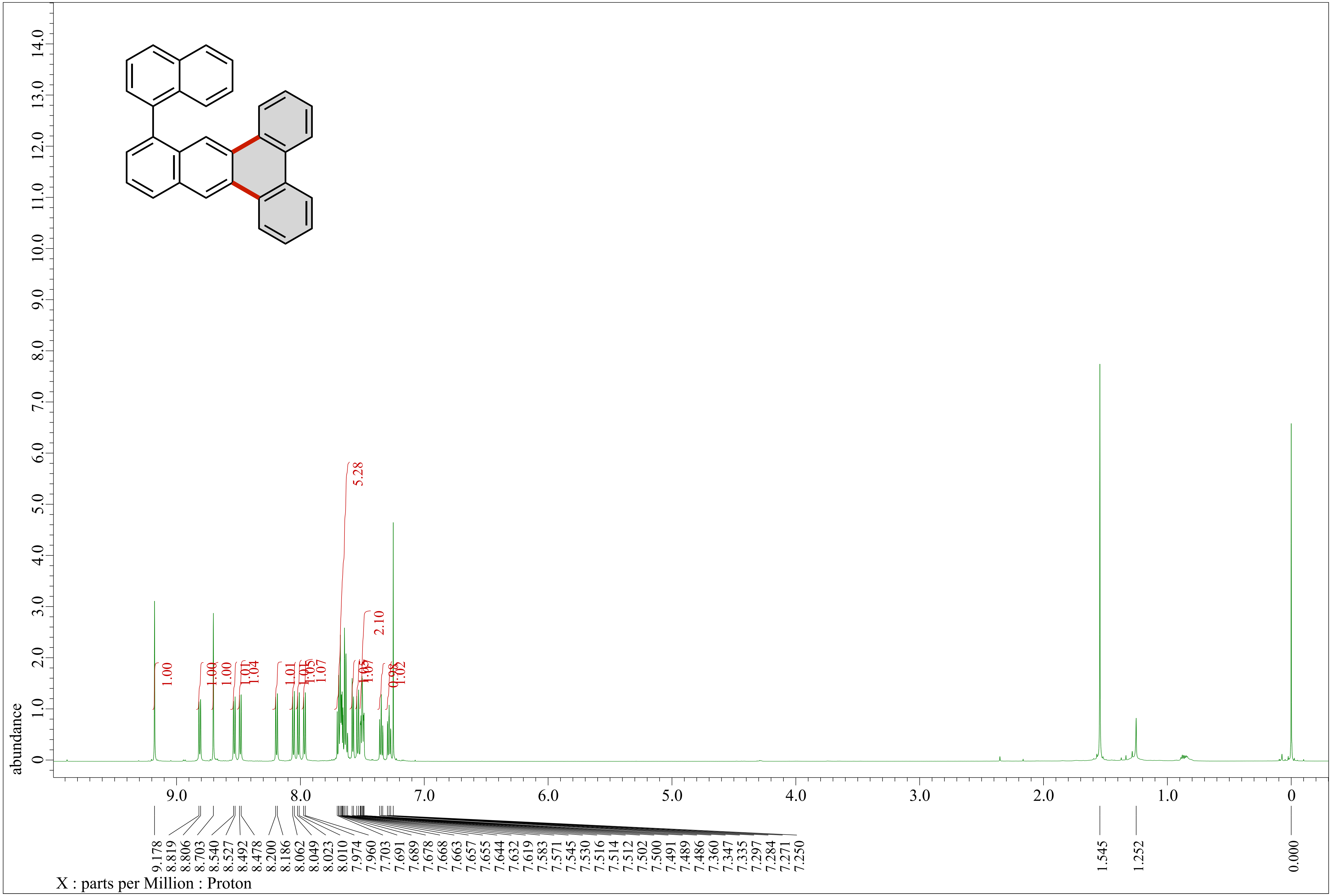

^{1}H NMR spectrum (inset) of **5ba** (solvent: $CDCl_3$)

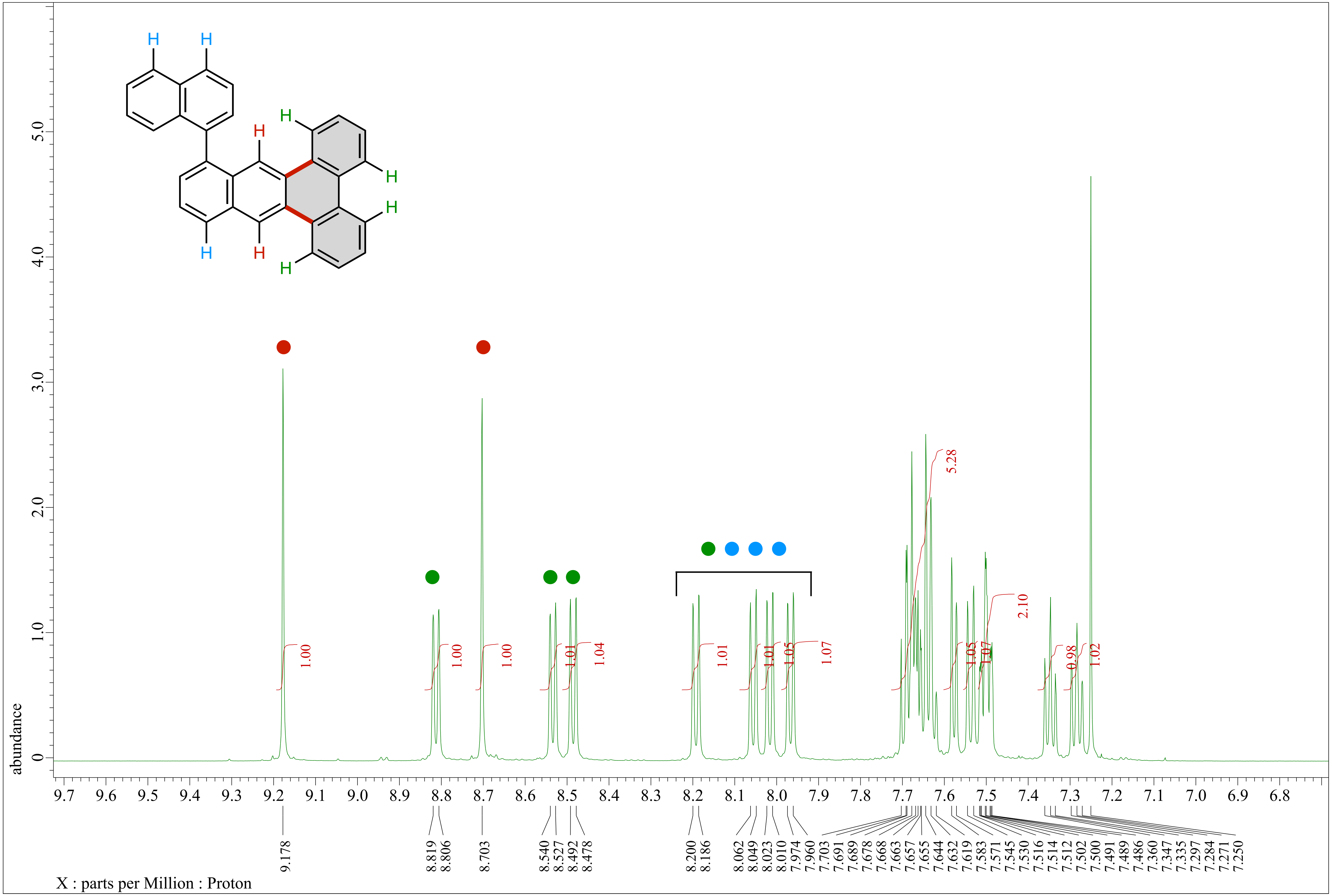

^{13}C NMR spectrum of **5ba** (solvent: $CDCl_3$)

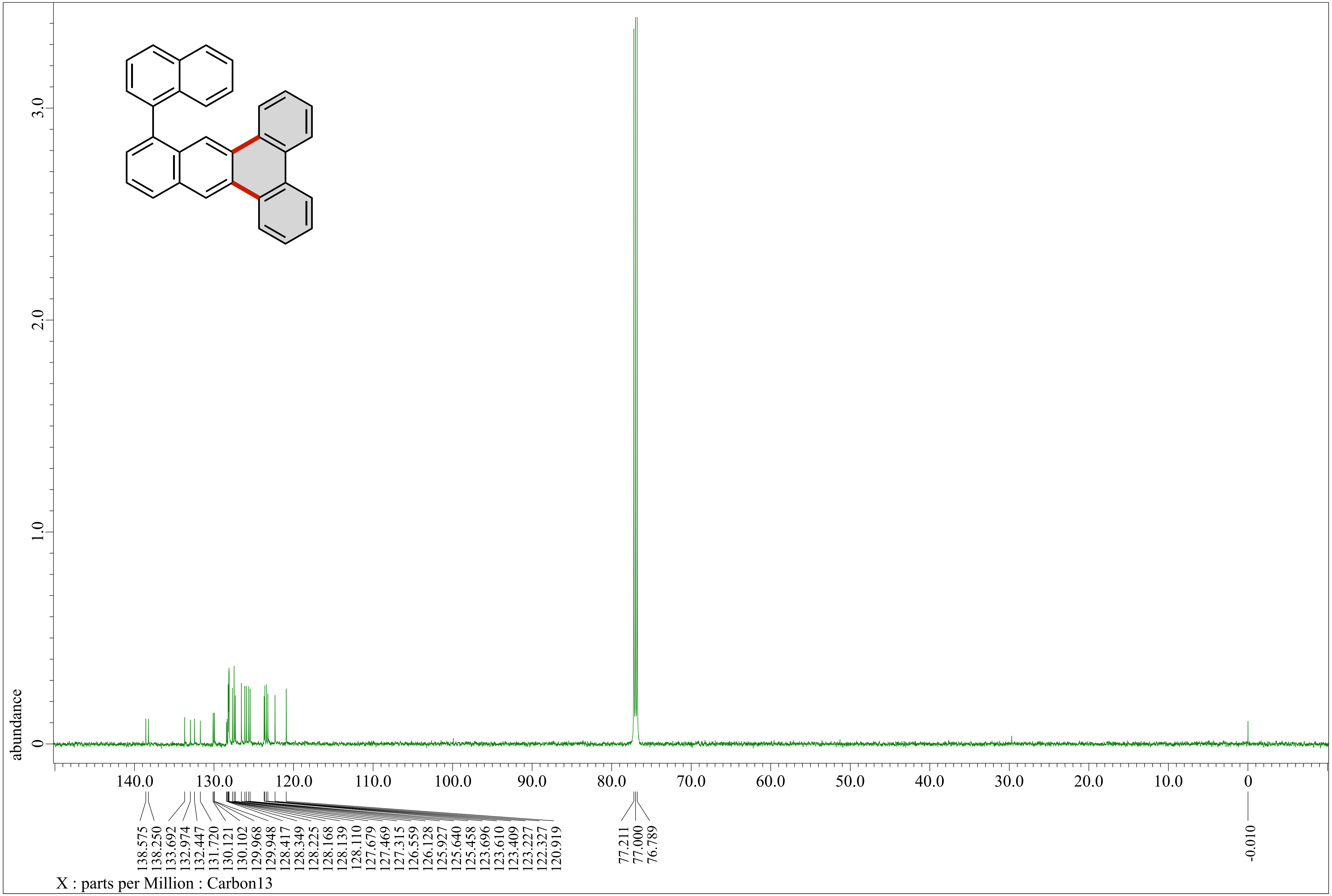

^{13}C NMR spectrum (inset) of **5ba** (solvent: $CDCl_3$)

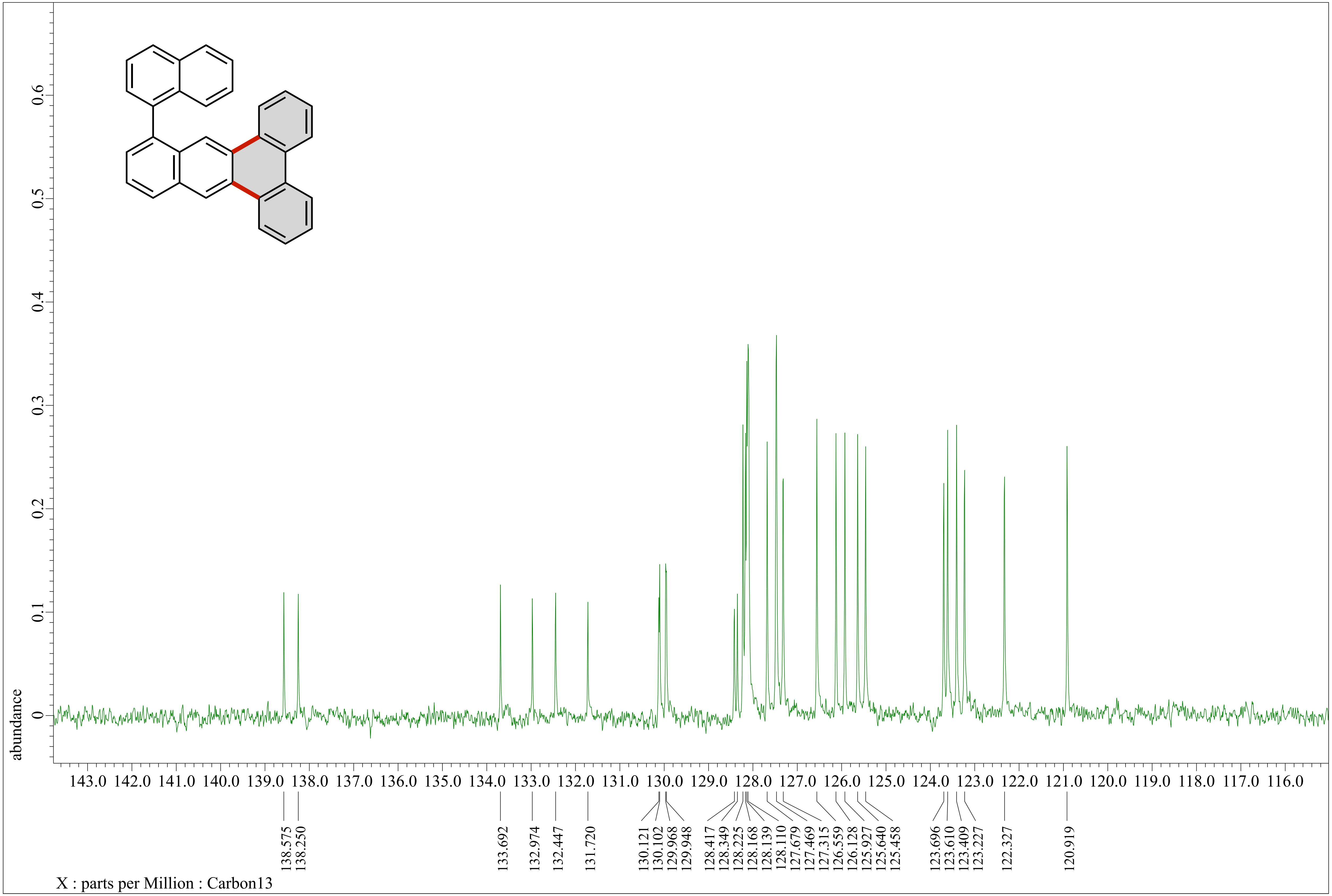

^{1}H NMR spectrum of **5ca** (solvent: $CDCl_3$)

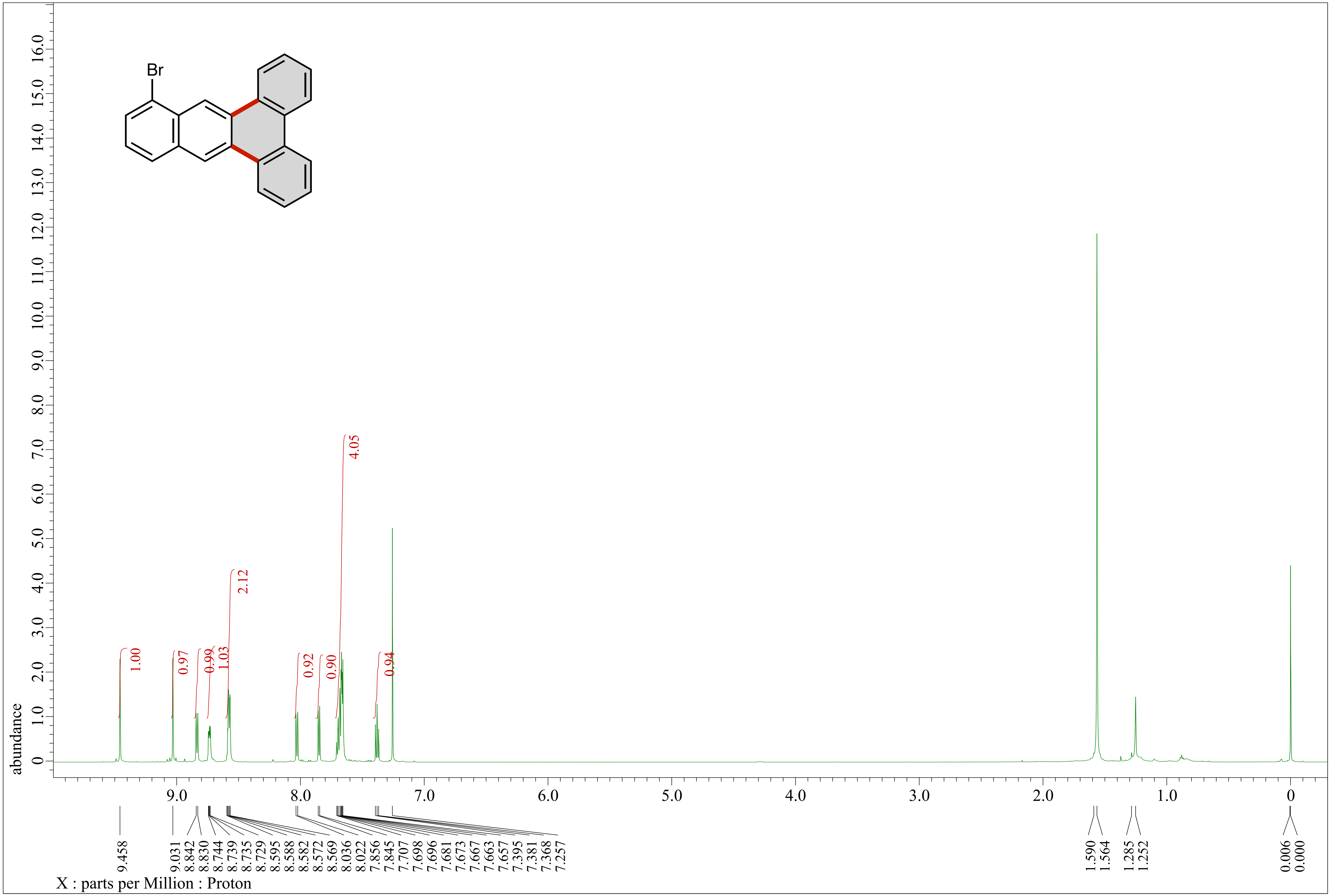

^{1}H NMR spectrum (inset) of **5ca** (solvent: $CDCl_3$)

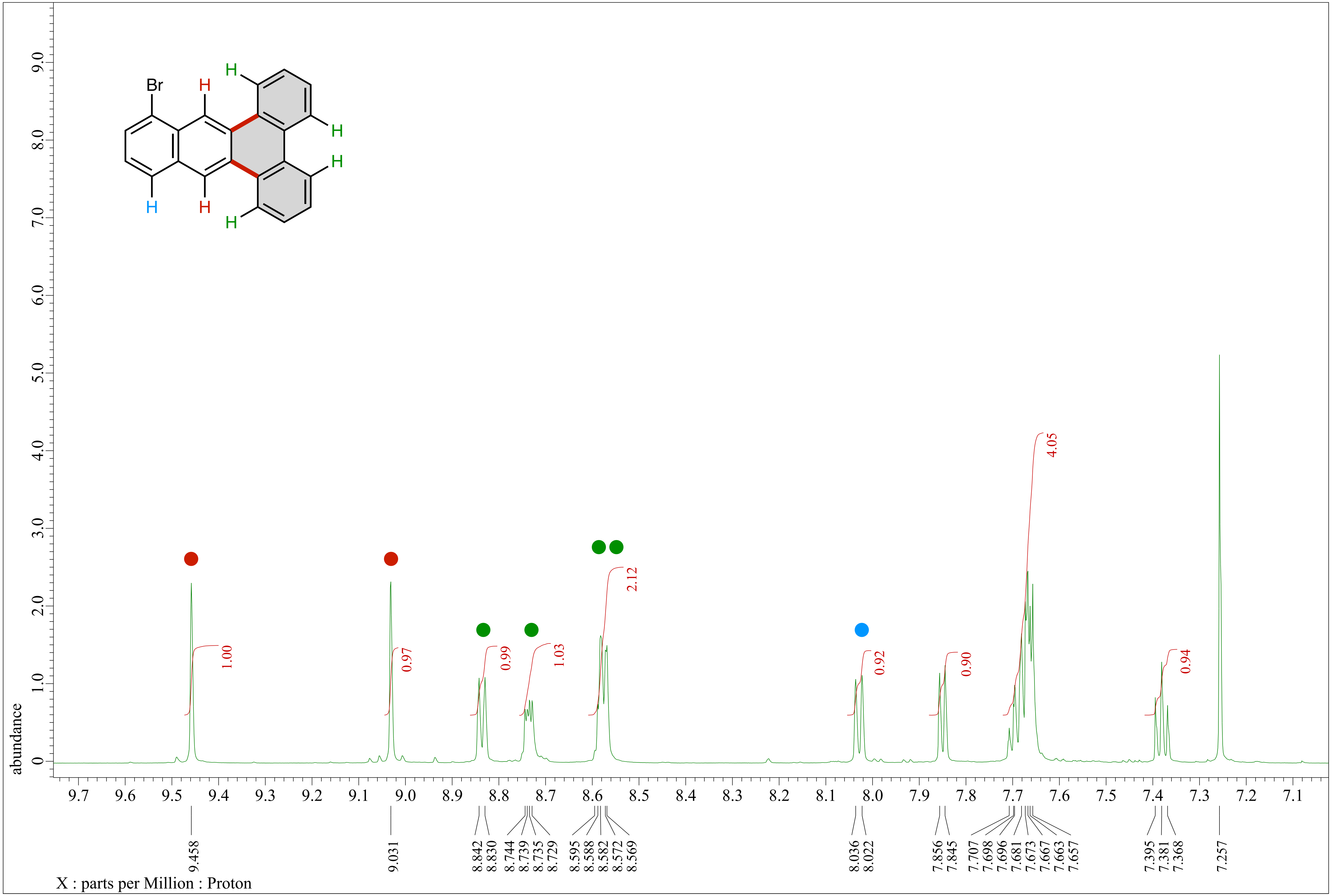

^{13}C NMR spectrum of **5ca** (solvent: $CDCl_3$)

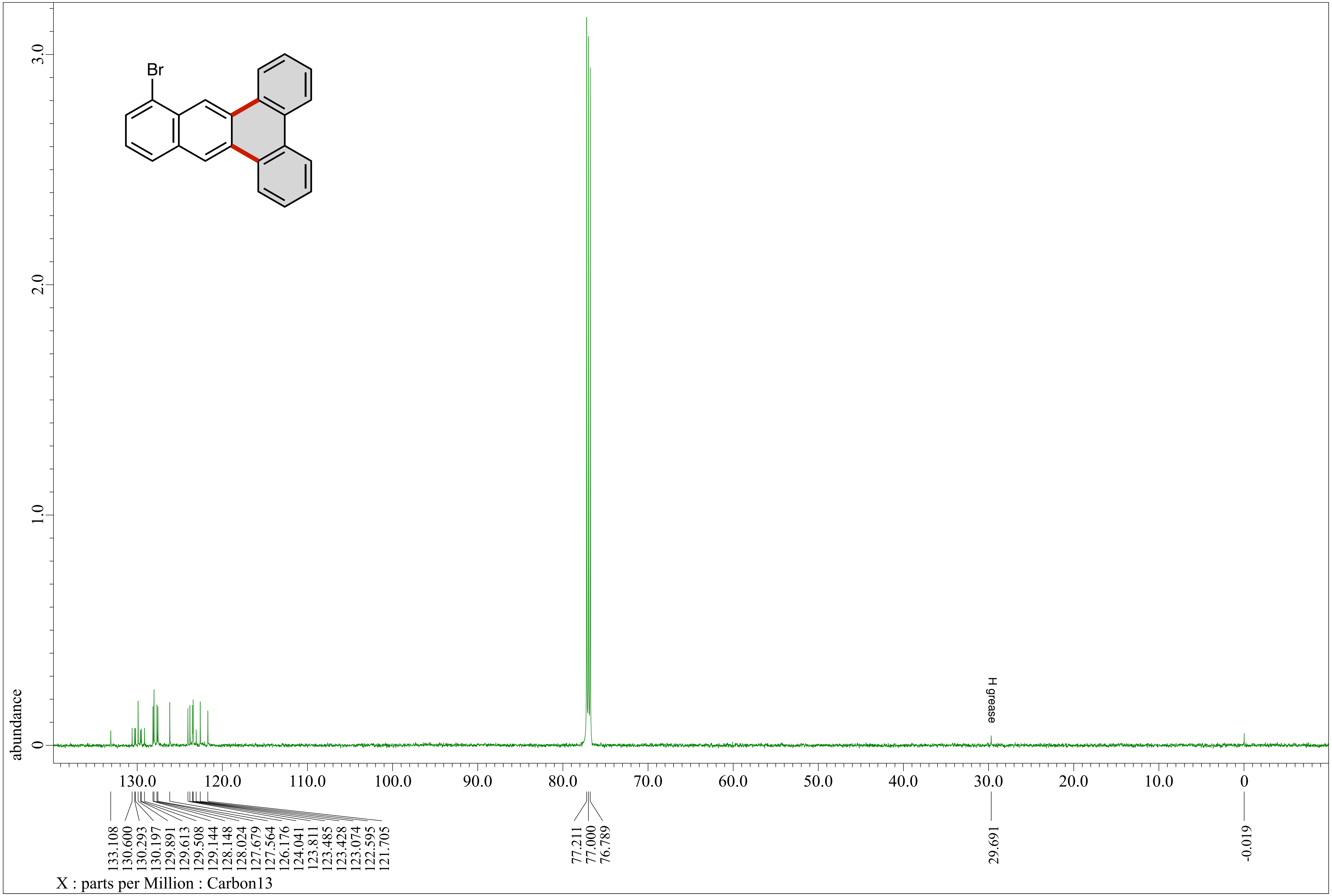

^{13}C NMR spectrum (inset) of **5ca** (solvent: $CDCl_3$)

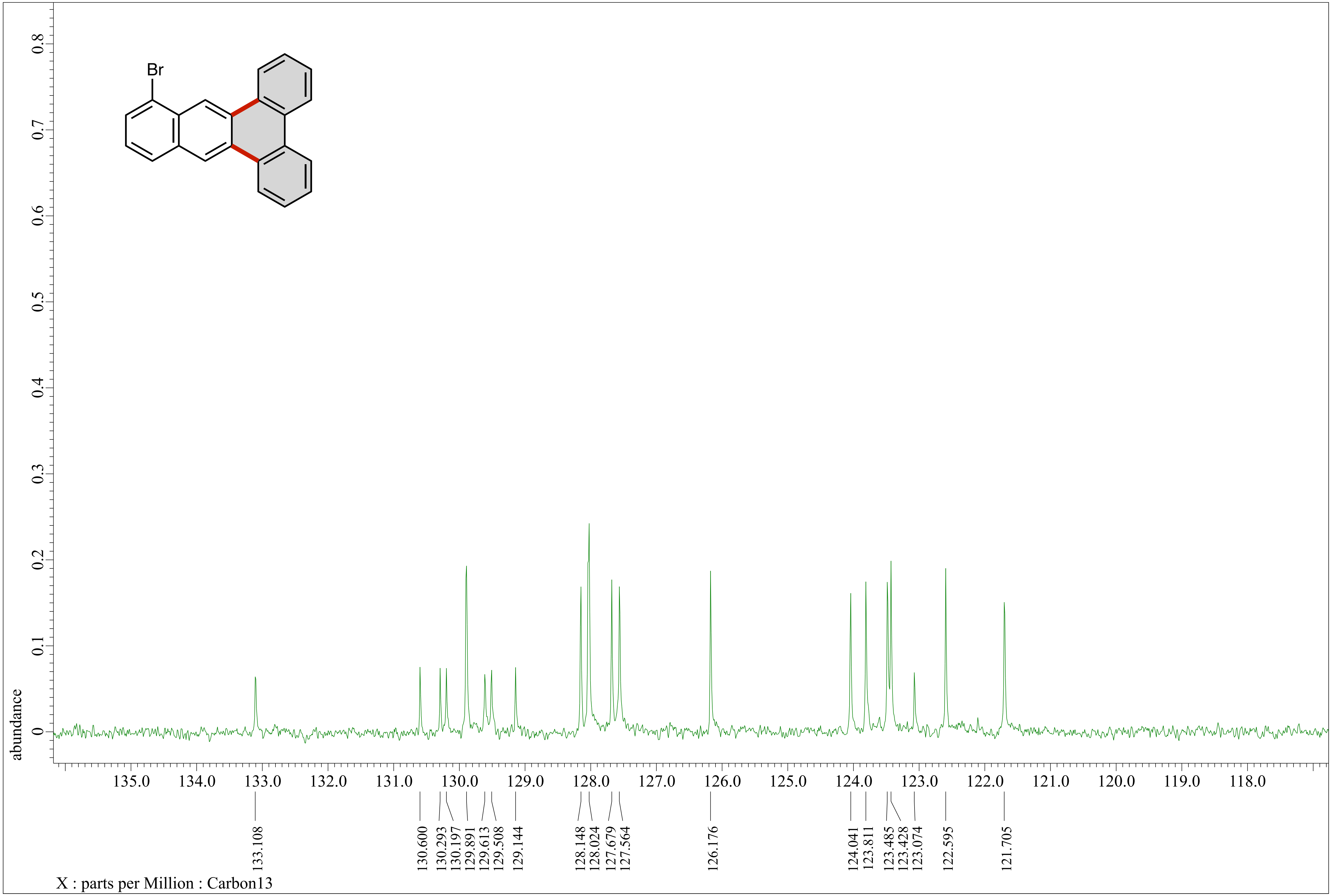

^{1}H NMR spectrum of **5da** (solvent: CD_2Cl_2)

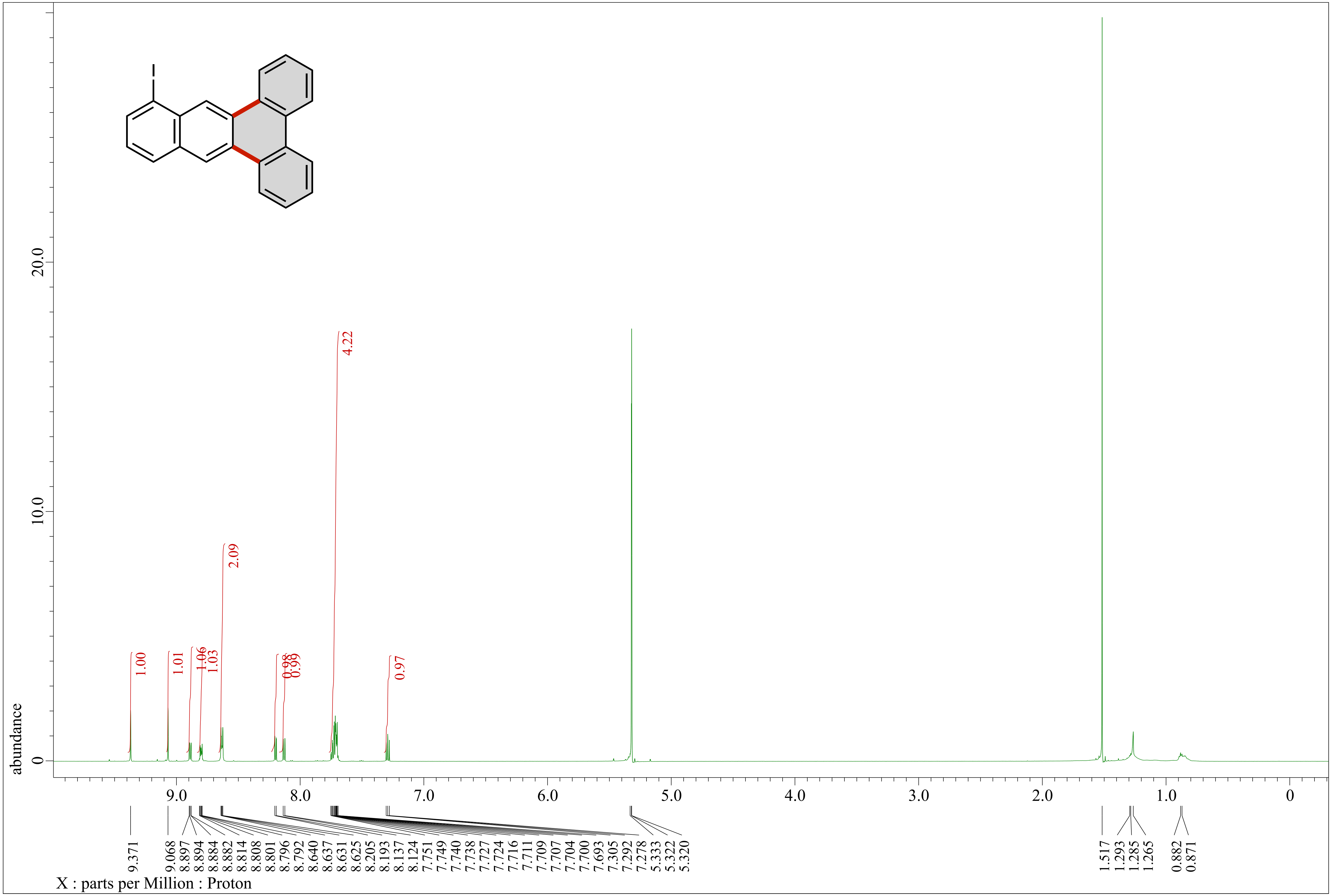

^{1}H NMR spectrum (inset) of **5da** (solvent: CD_2Cl_2)

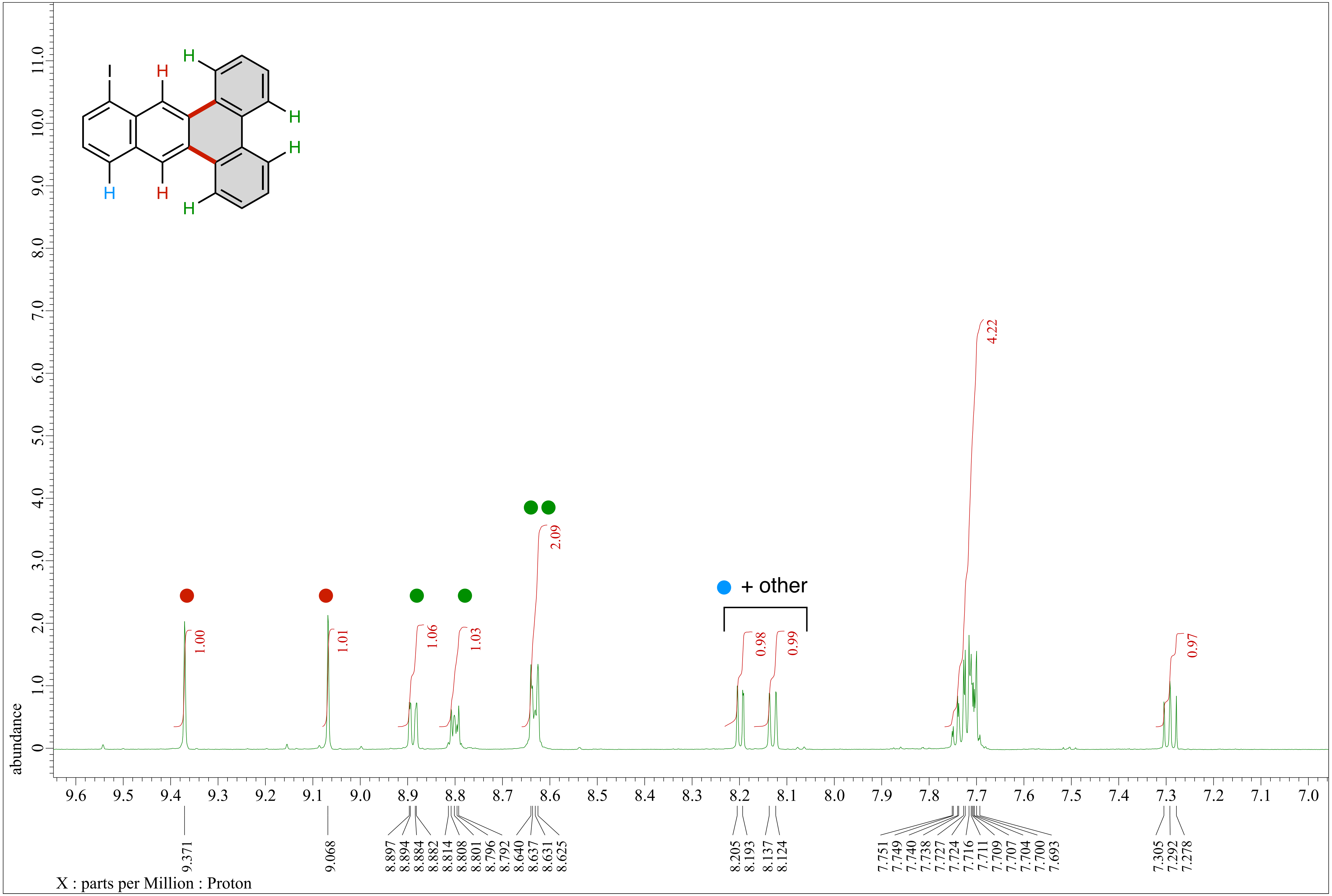

^{13}C NMR spectrum of **5da** (solvent: CD_2Cl_2)

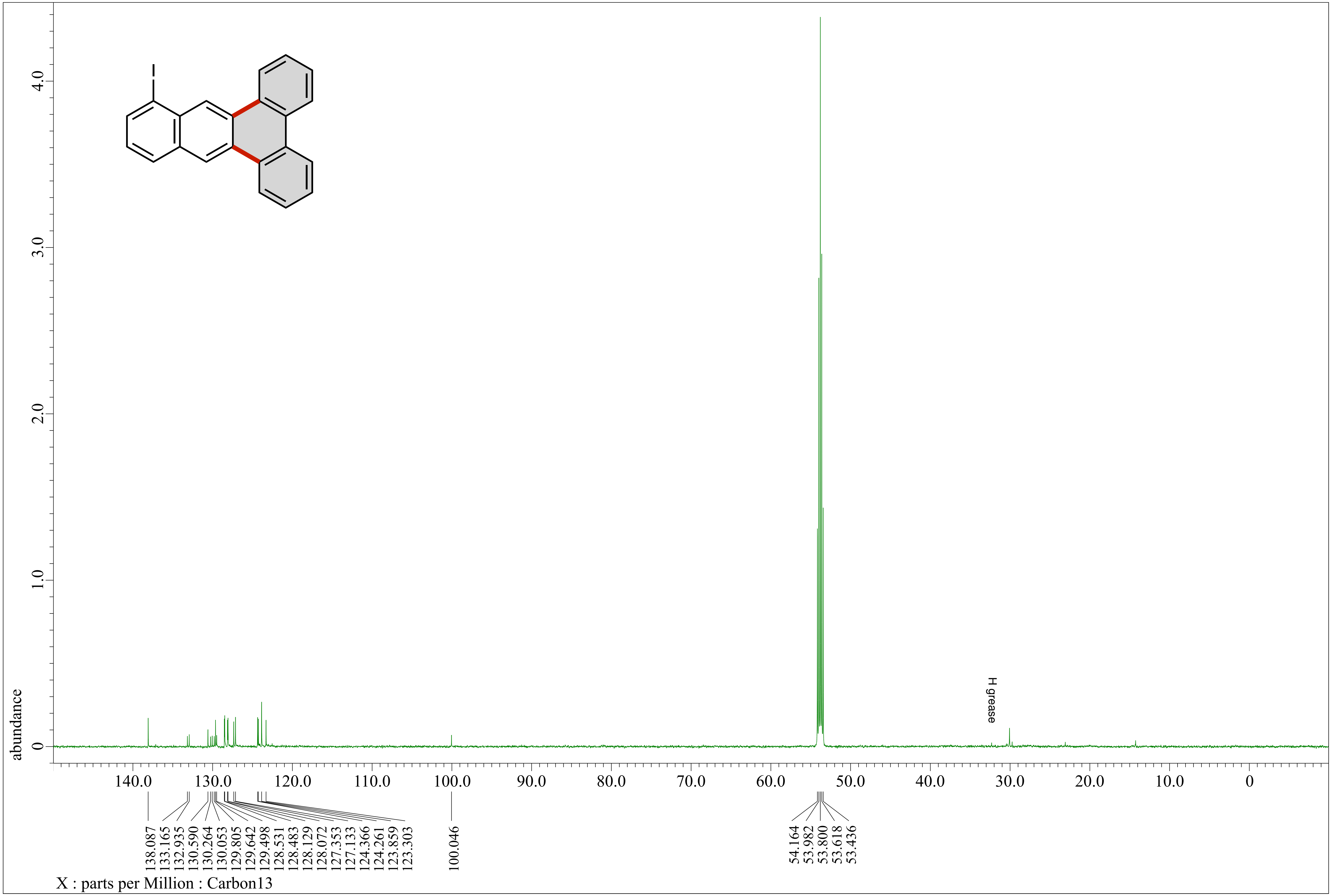

^{13}C NMR spectrum (inset) of **5da** (solvent: CD_2Cl_2)

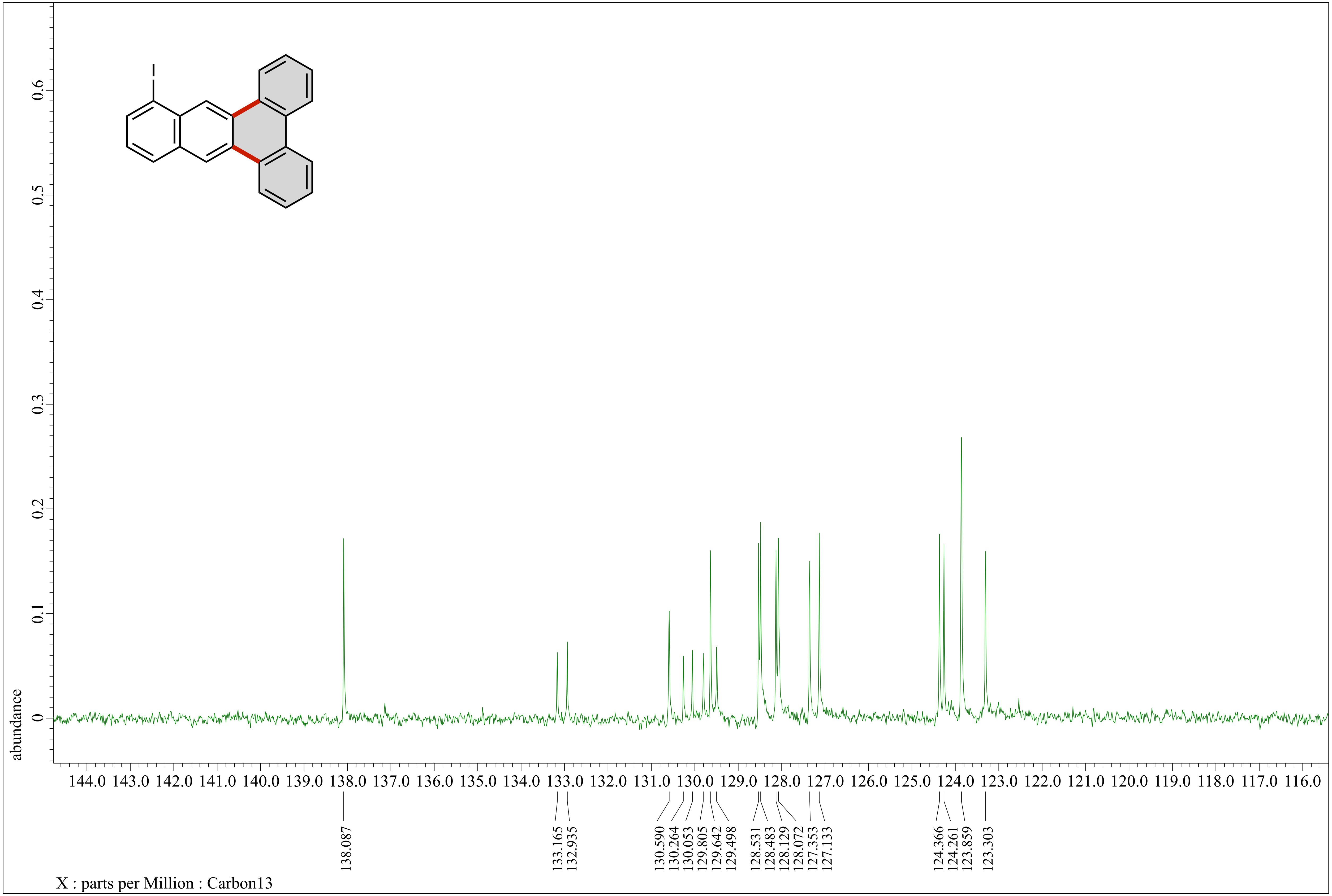

^{1}H NMR spectrum of **5ea** (solvent: $CDCl_3$)

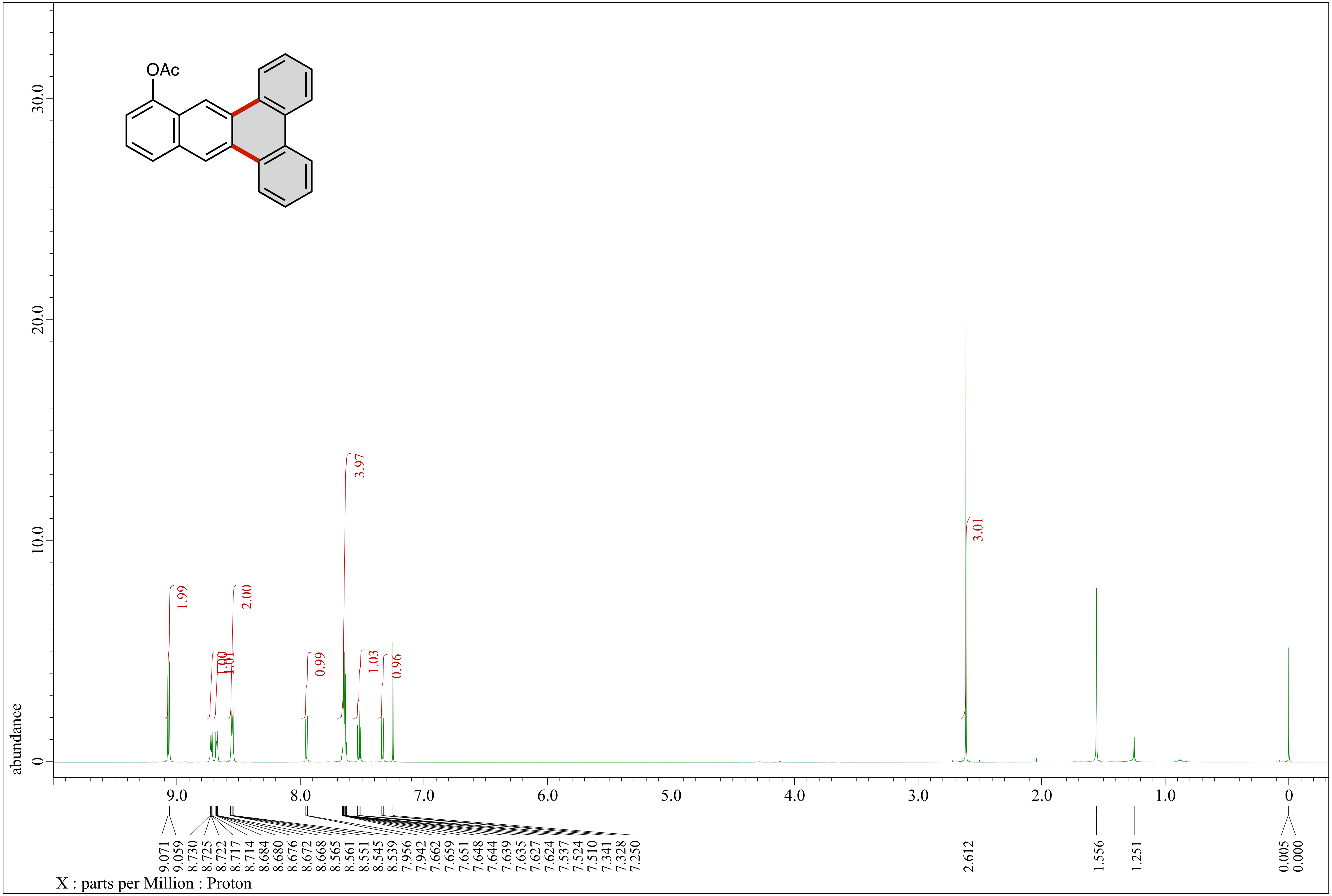

^{1}H NMR spectrum (inset) of **5ea** (solvent: $CDCl_3$)

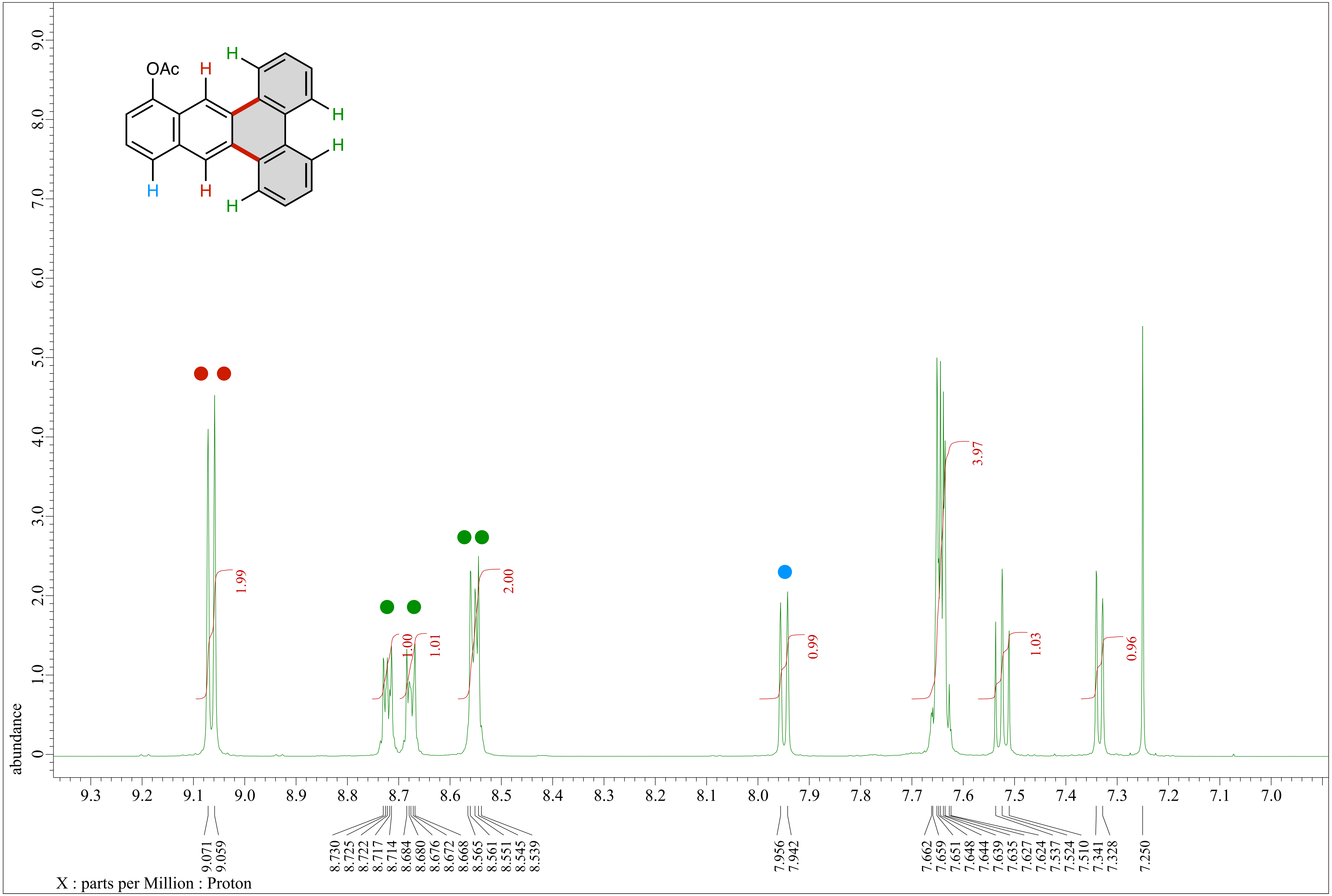

^{13}C NMR spectrum of **5ea** (solvent: $CDCl_3$)

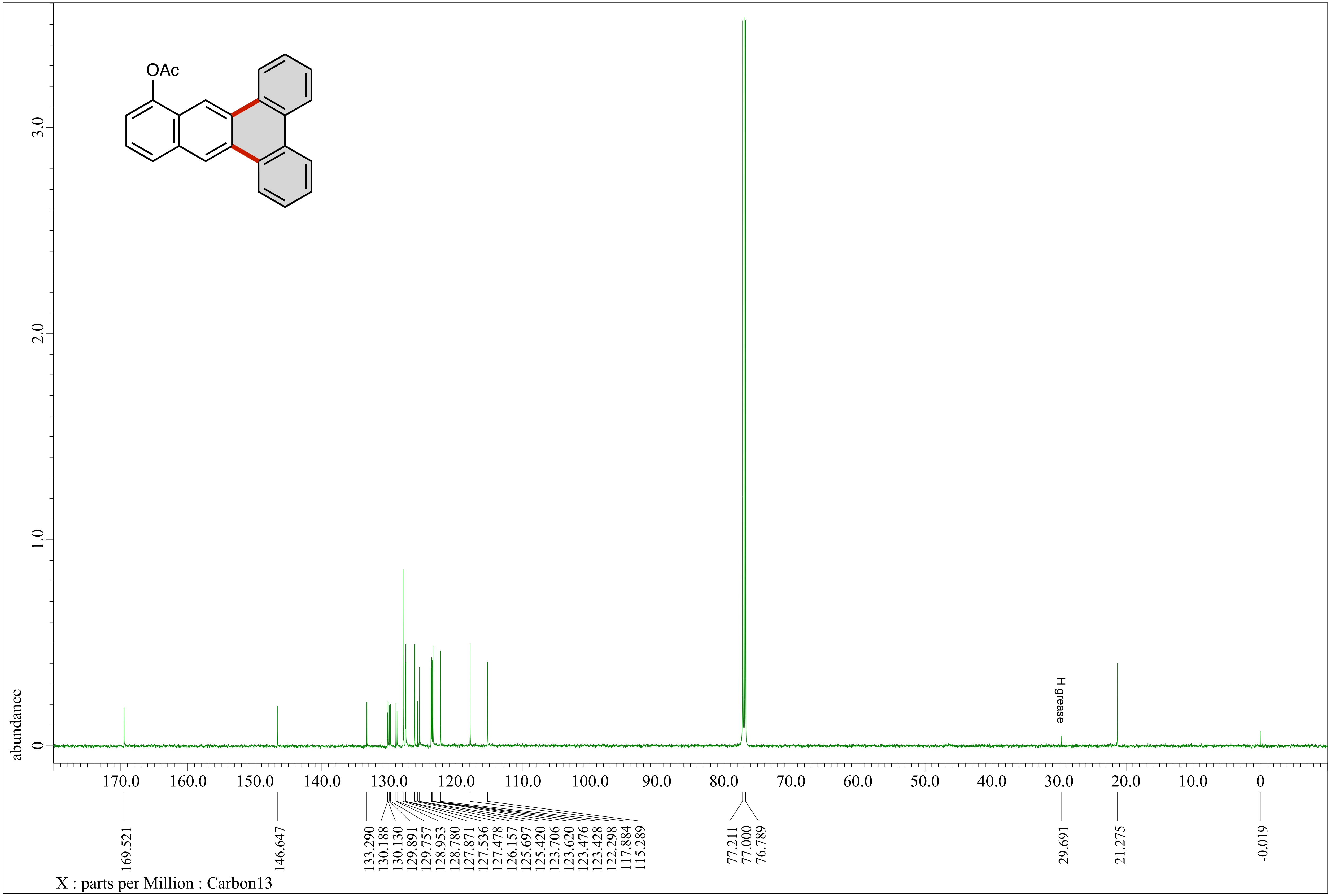

^{13}C NMR spectrum (inset) of **5ea** (solvent: $CDCl_3$)

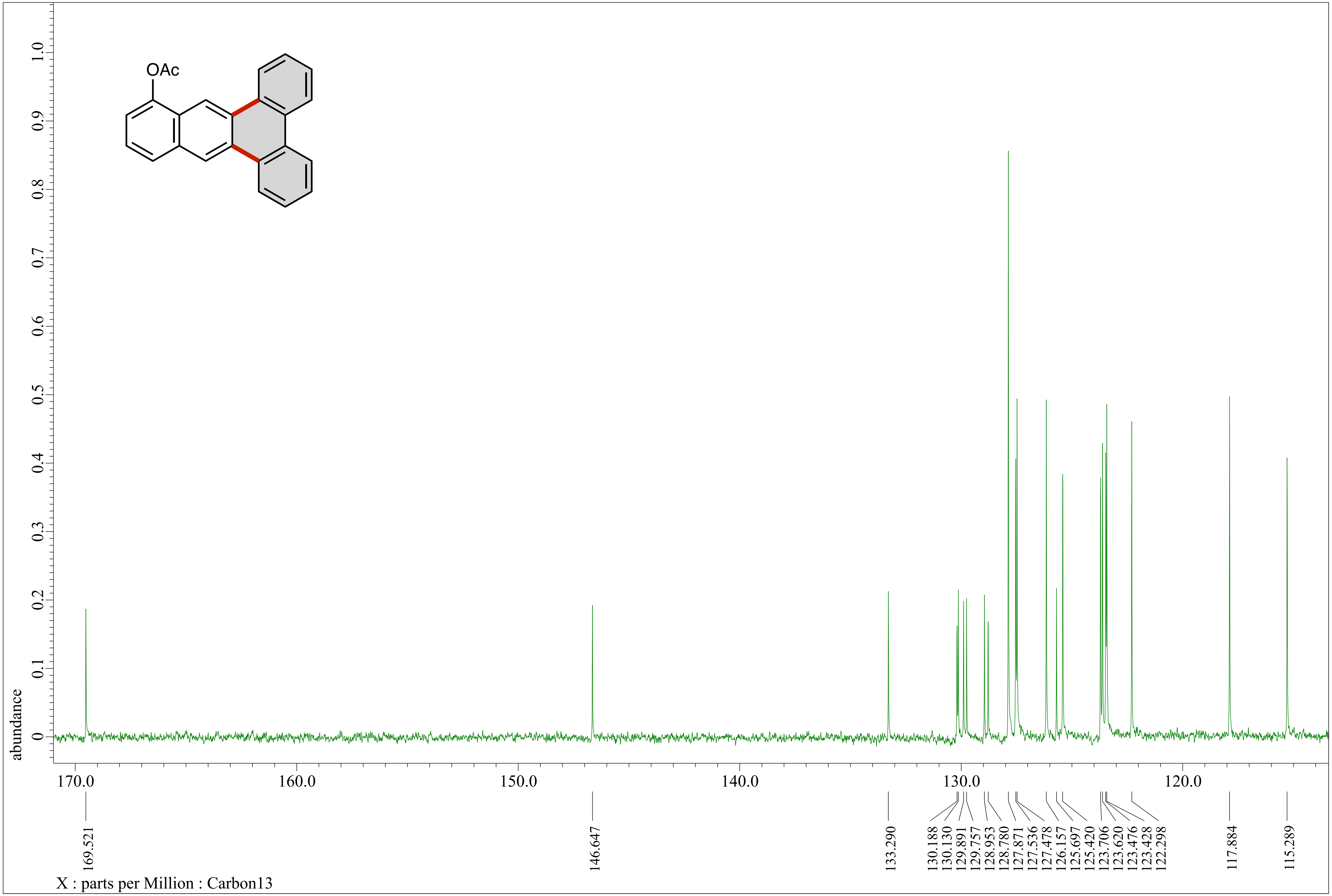

^{1}H NMR spectrum of **5fa** (solvent: $CDCl_3$)

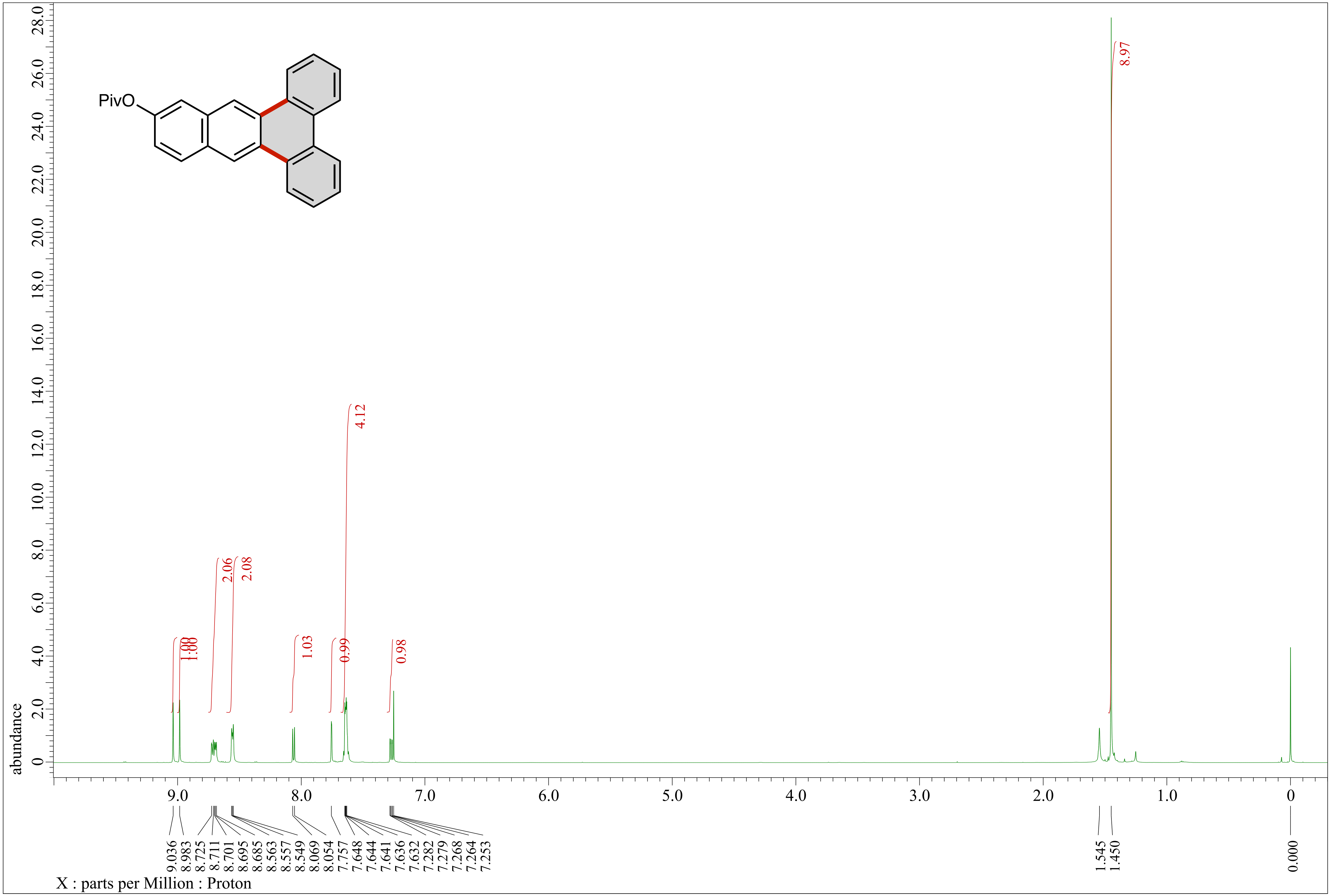

^{1}H NMR spectrum (inset) of **5fa** (solvent: $CDCl_3$)

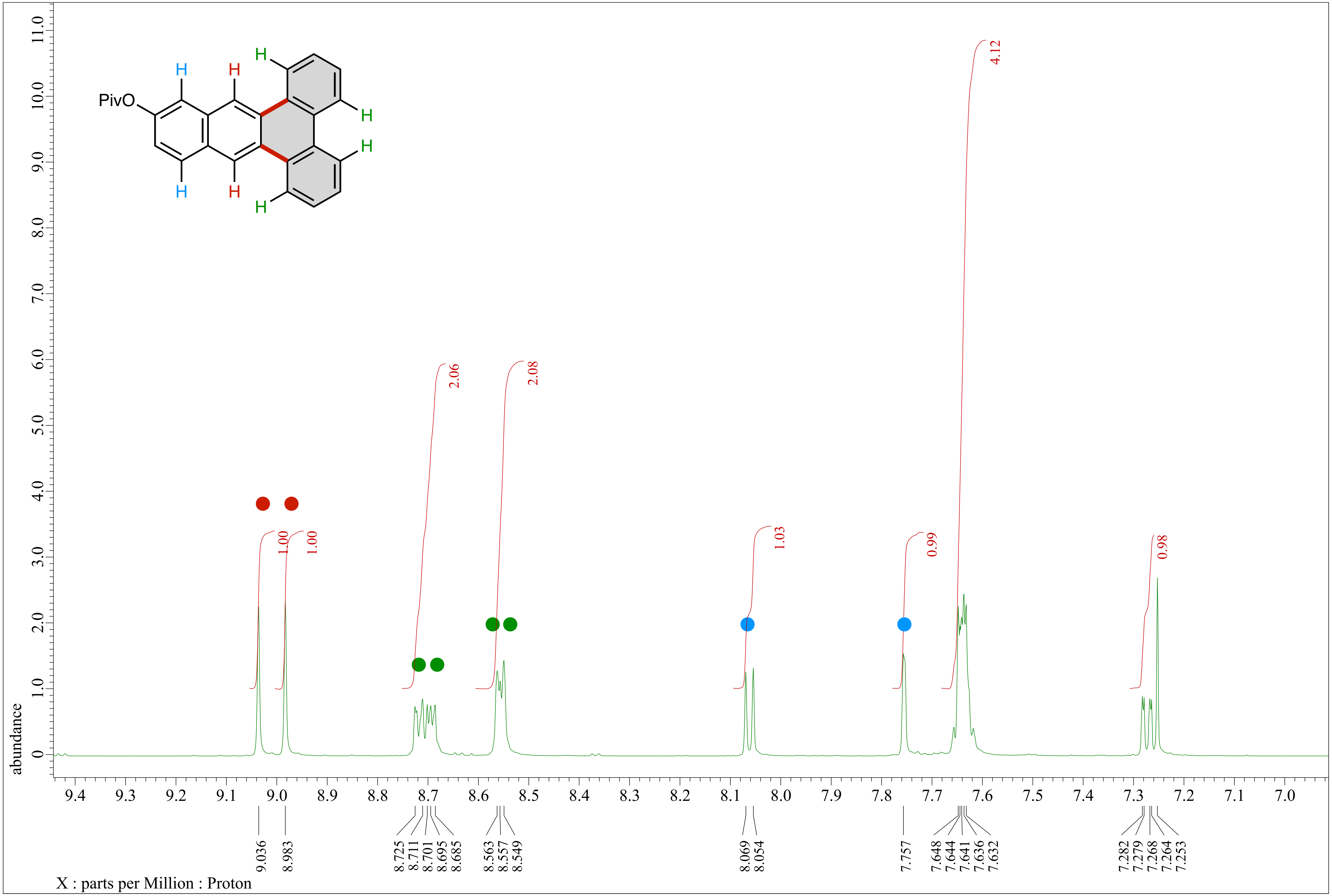

^{13}C NMR spectrum of **5fa** (solvent: $CDCl_3$)

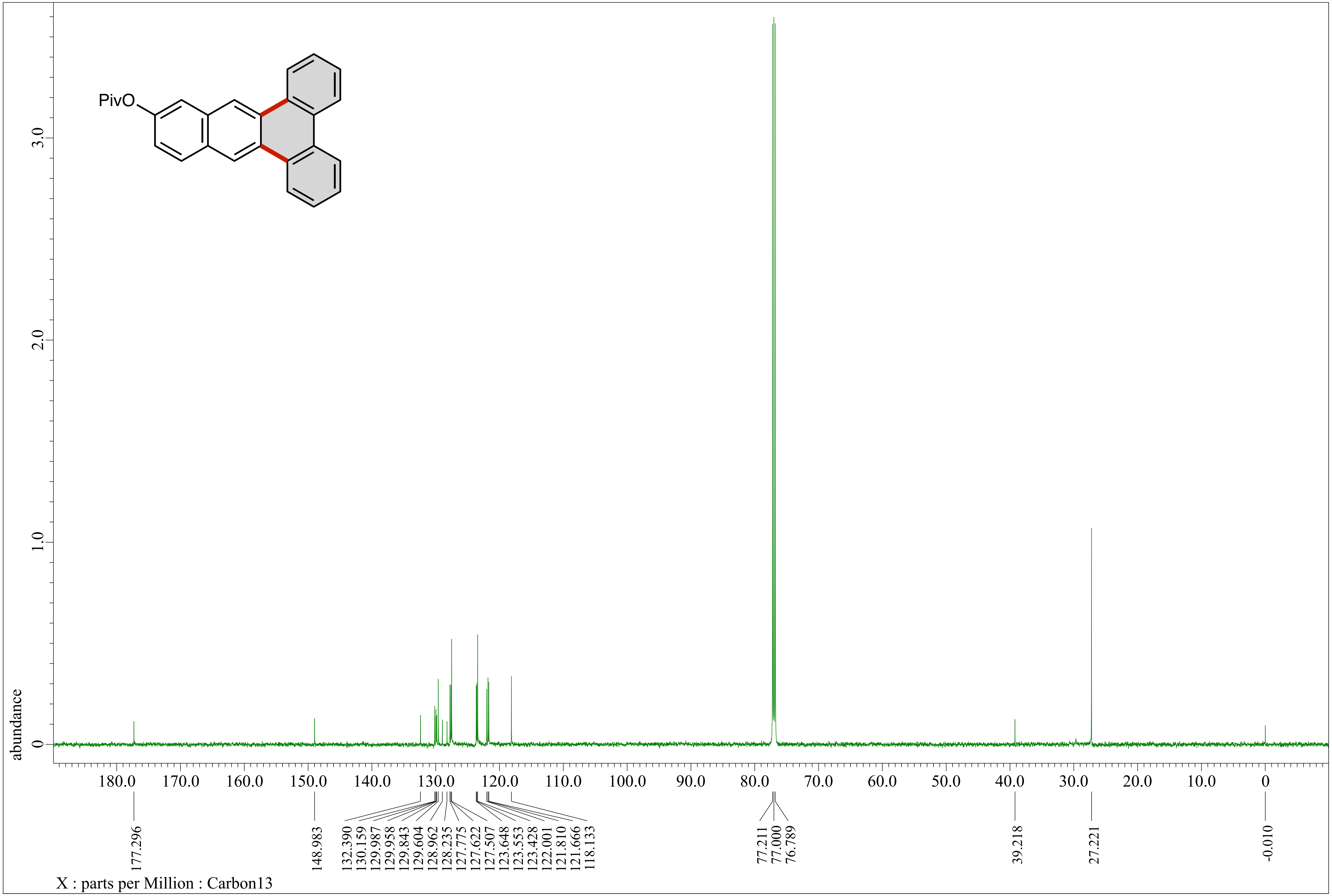

^{13}C NMR spectrum (inset) of **5fa** (solvent: $CDCl_3$)

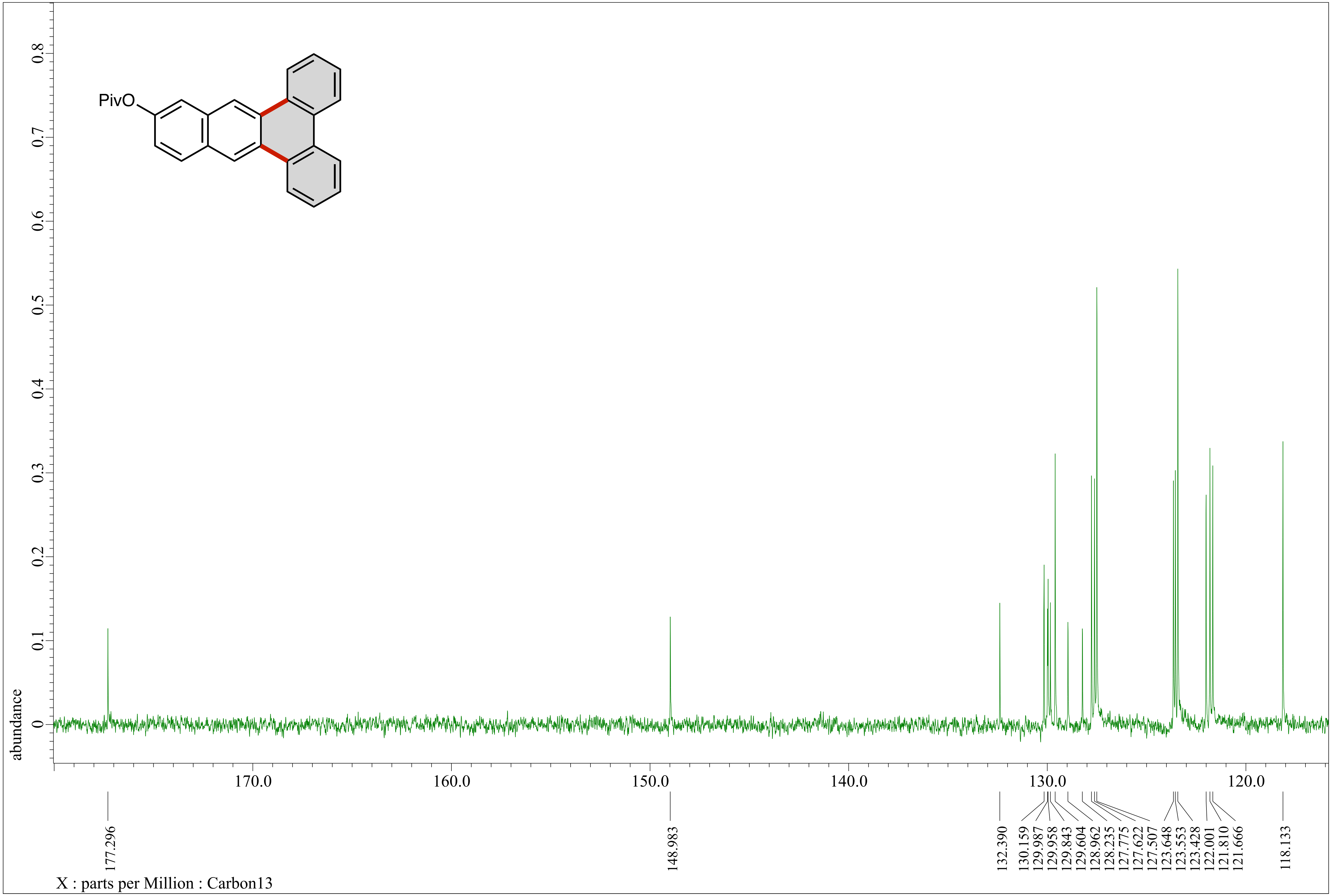

^{1}H NMR spectrum of **5ga** (solvent: $CDCl_3$)

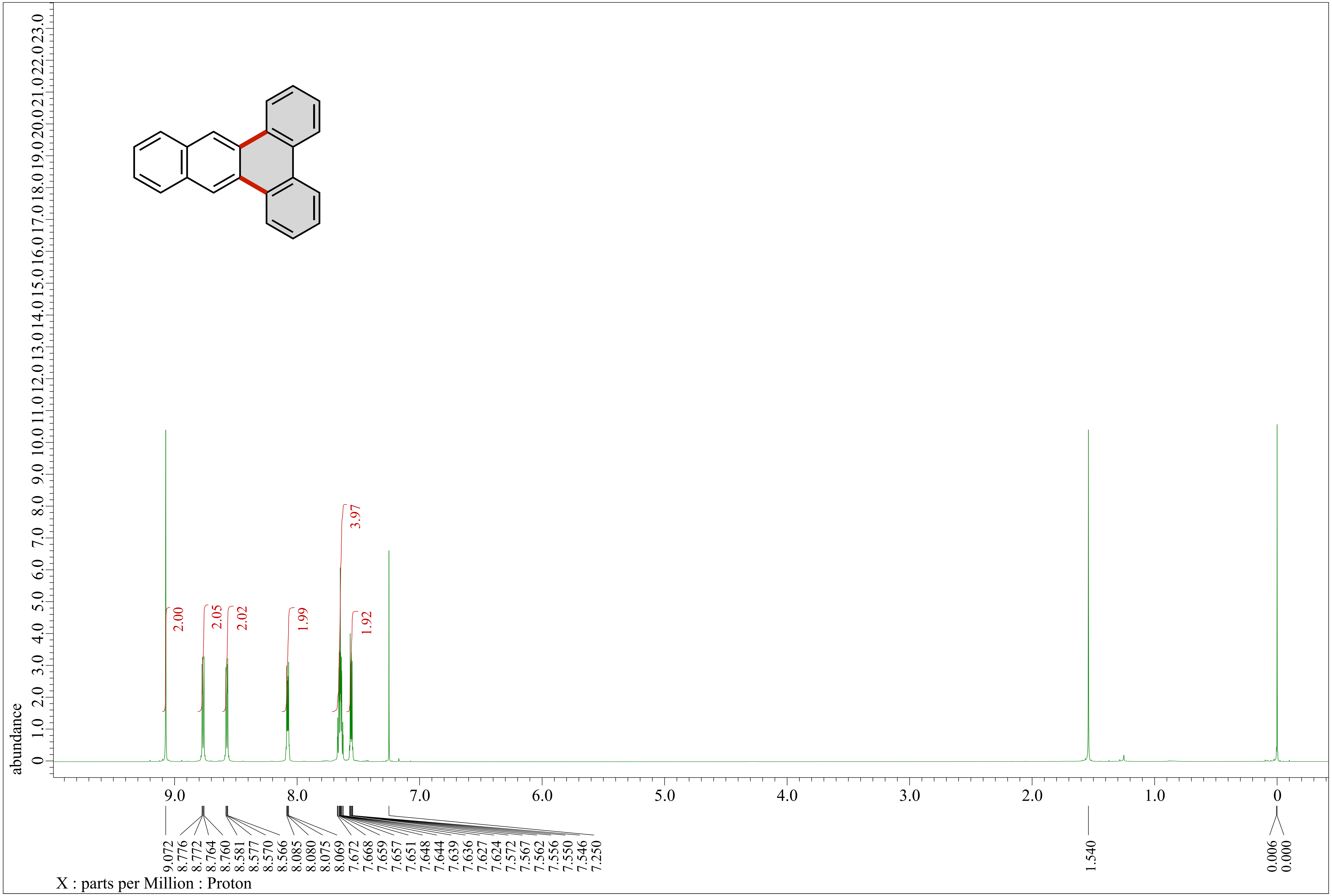

^{1}H NMR spectrum (inset) of **5ga** (solvent: $CDCl_3$)

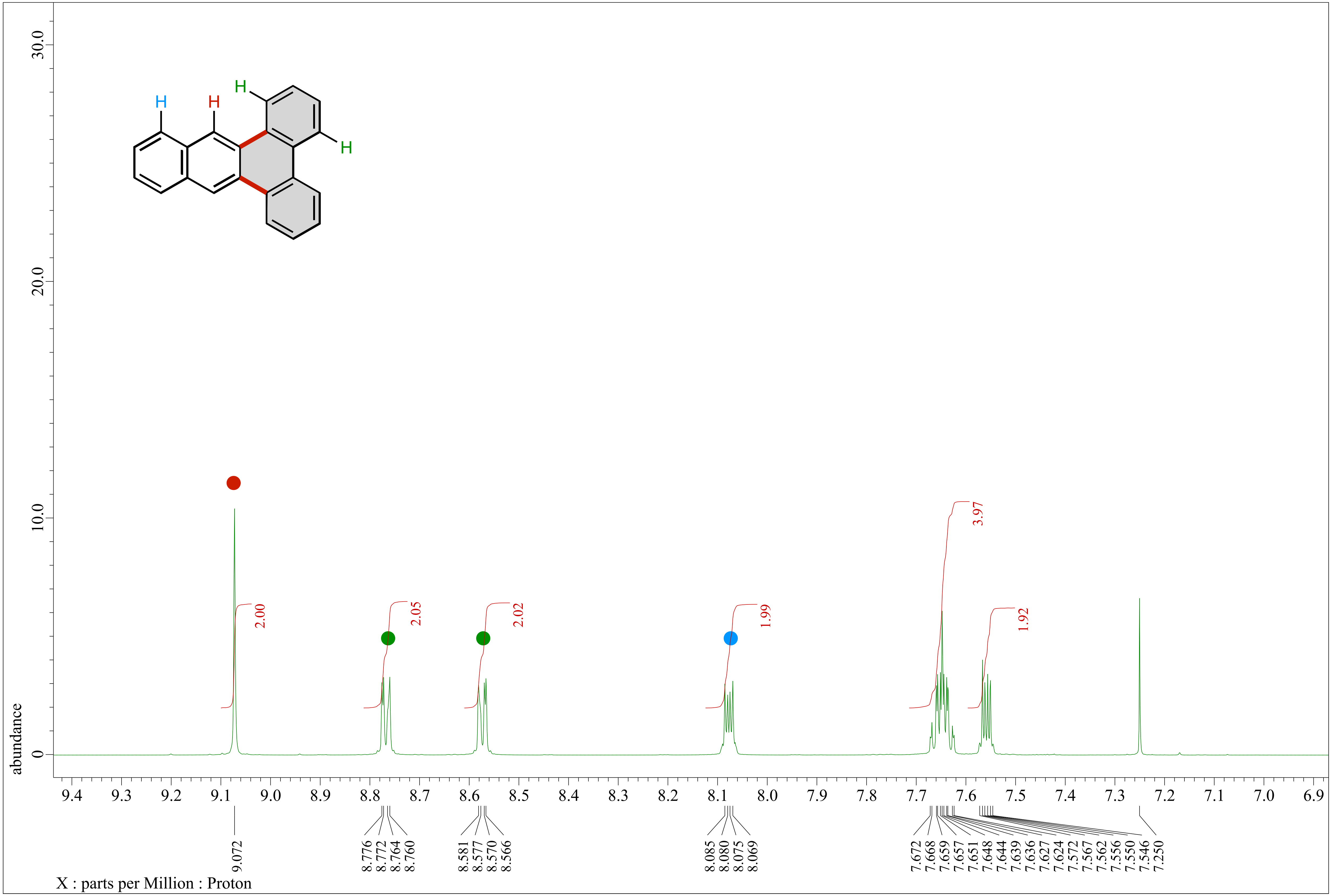

^{13}C NMR spectrum of **5ga** (solvent: $CDCl_3$)

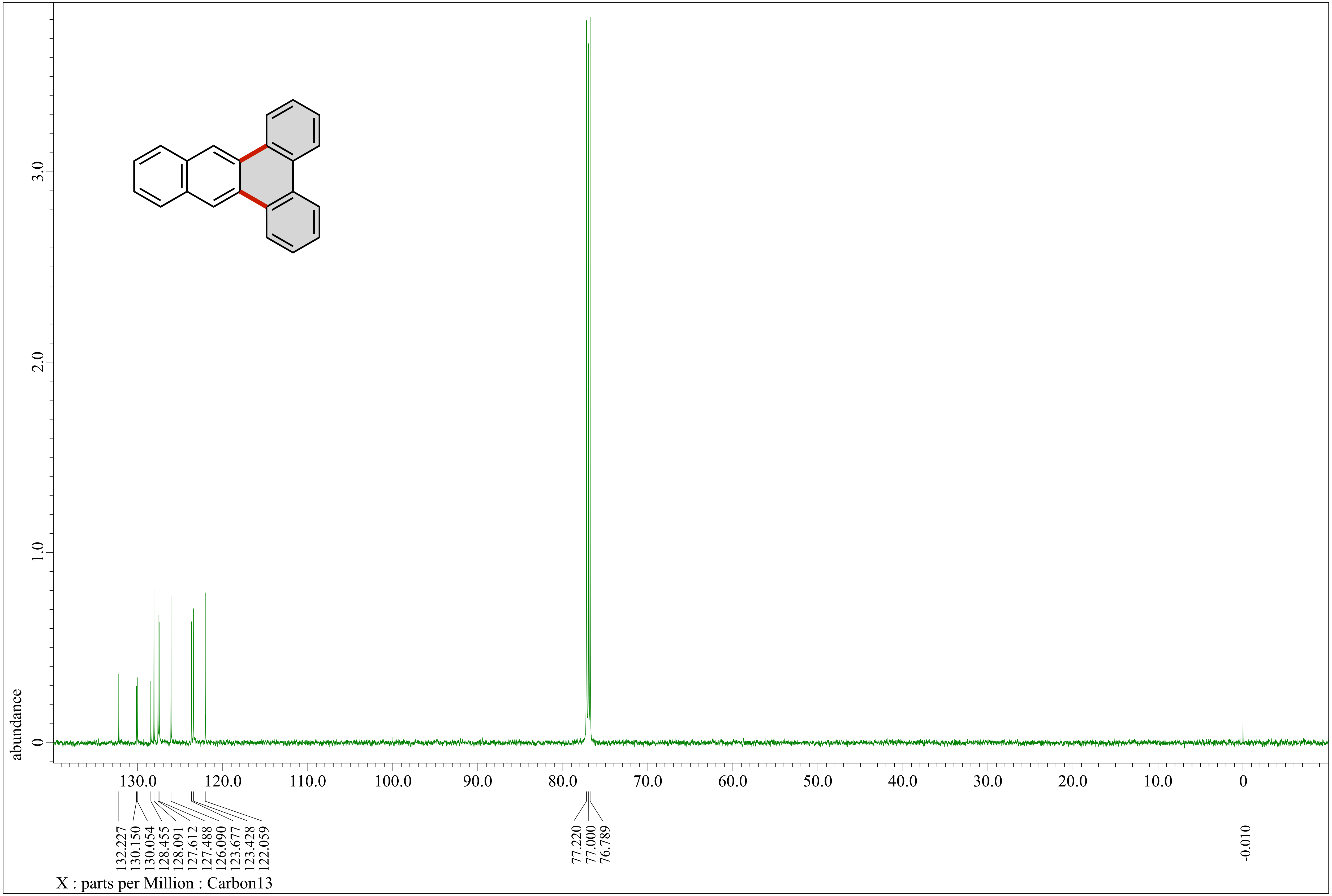

^{13}C NMR spectrum (inset) of **5ga** (solvent: $CDCl_3$)

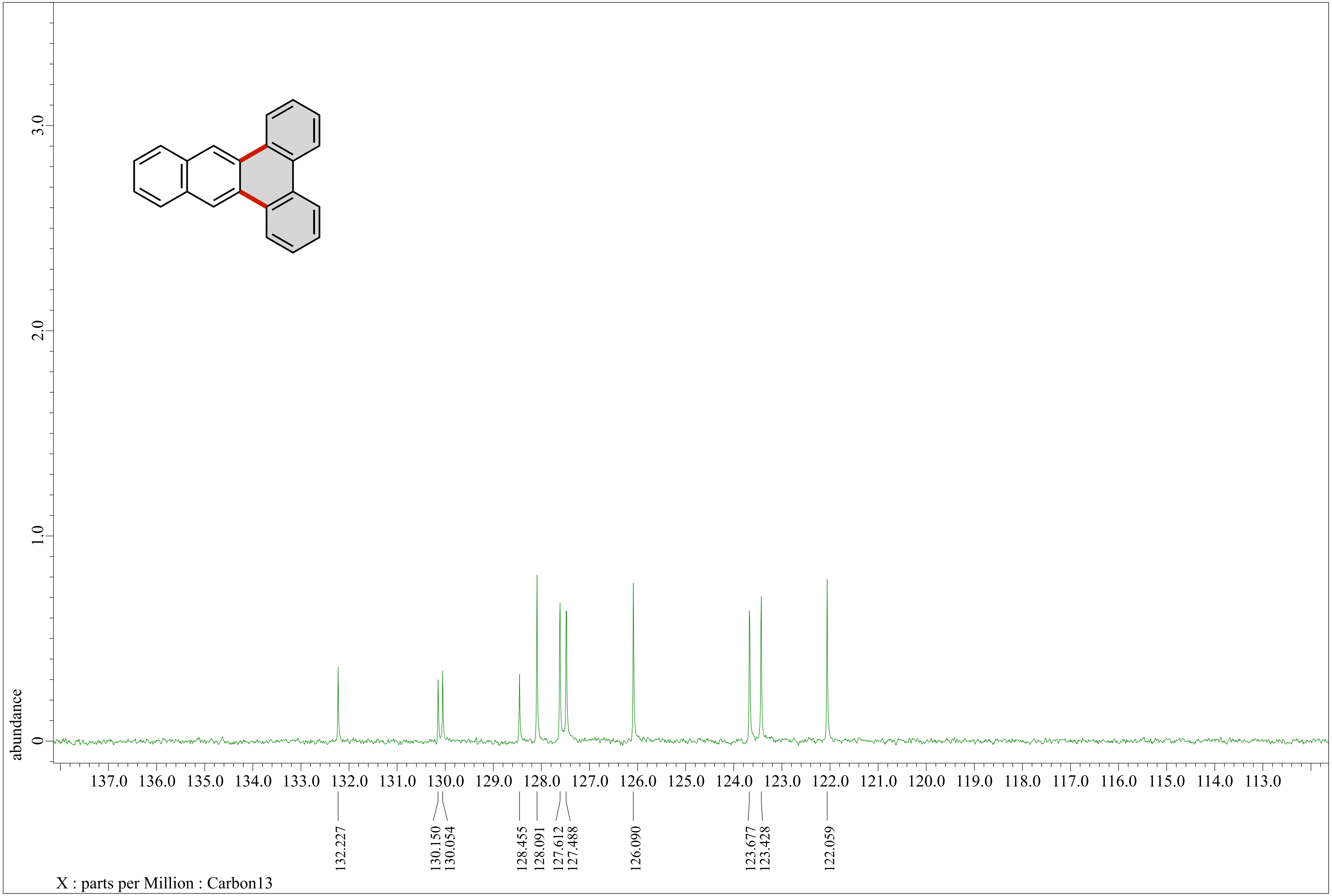

^{1}H NMR spectrum of **5ha** (solvent: $CDCl_3$)

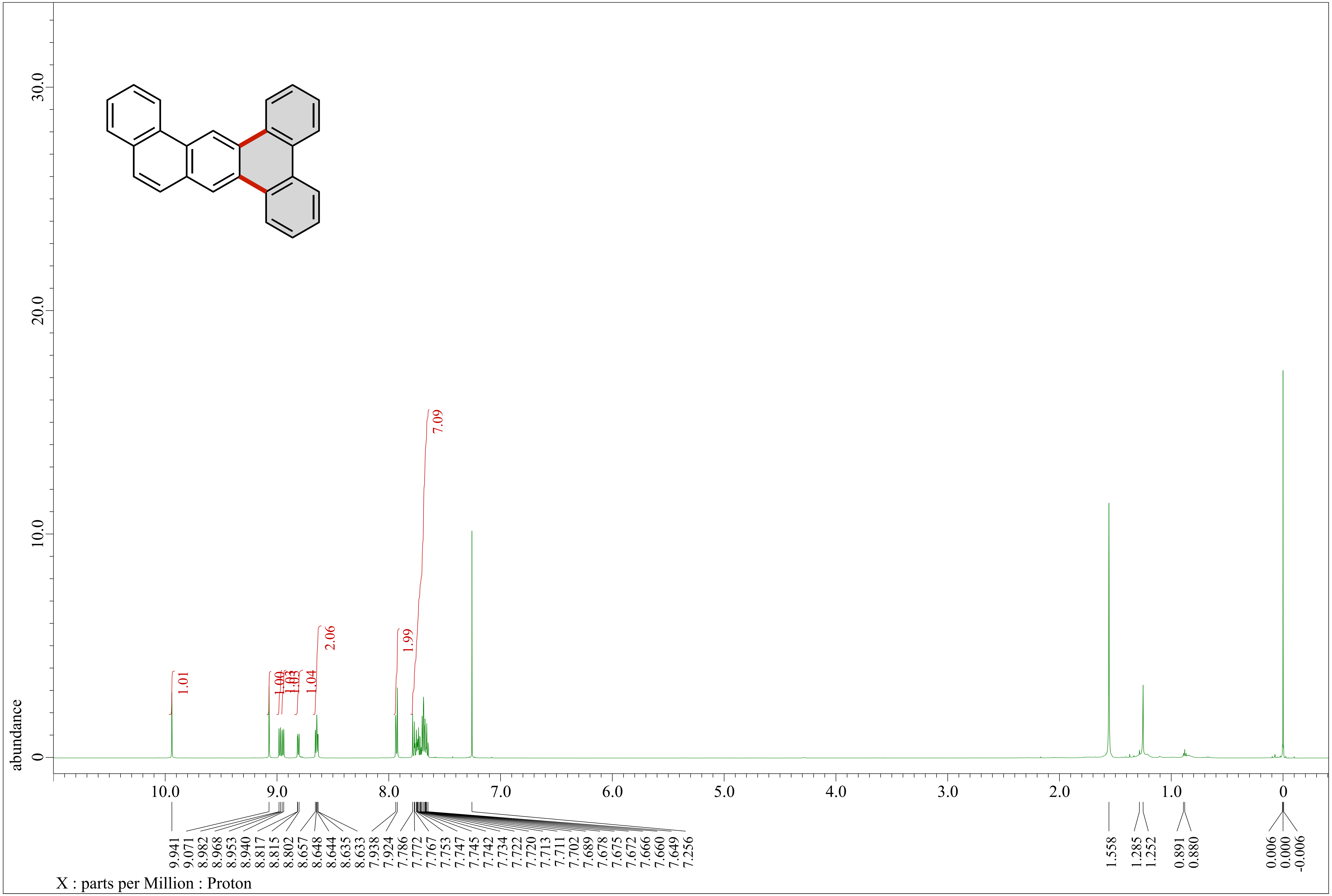

^{1}H NMR spectrum (inset) of **5ha** (solvent: $CDCl_3$)

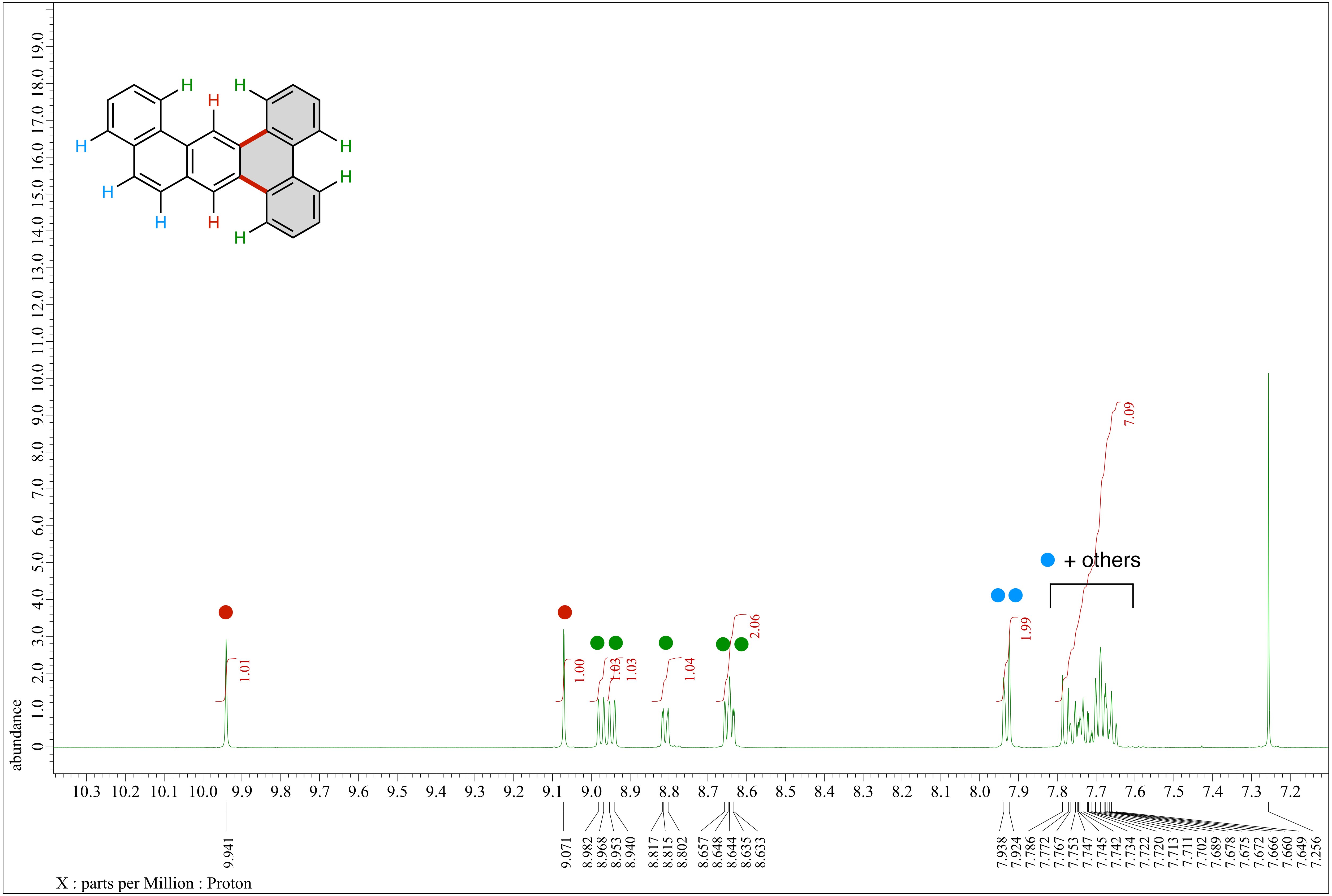

^{13}C NMR spectrum of **5ha** (solvent: $CDCl_3$)

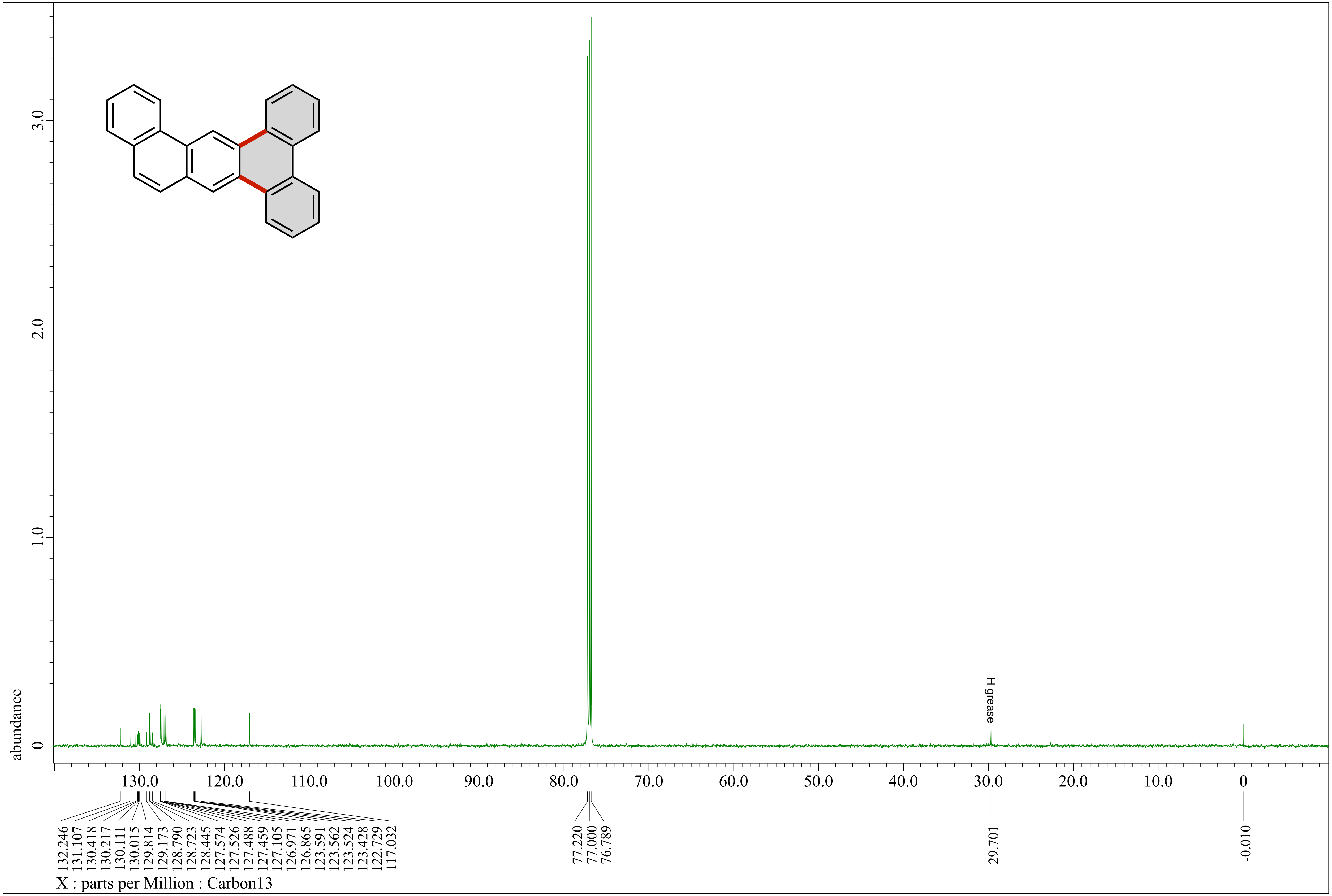

^{13}C NMR spectrum (inset) of **5ha** (solvent: $CDCl_3$)

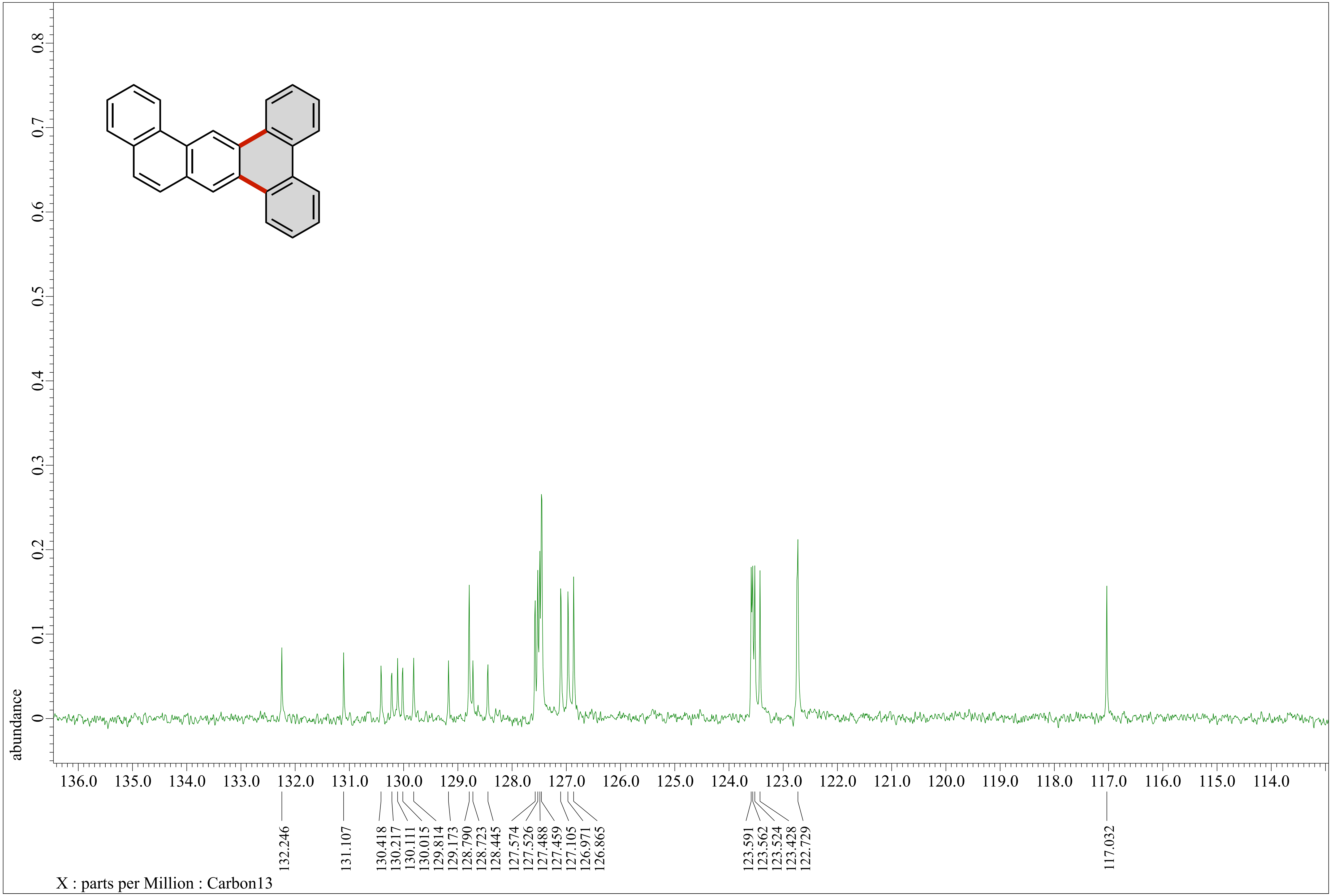

^{1}H NMR spectrum of **5ia** (solvent: $CDCl_3$)

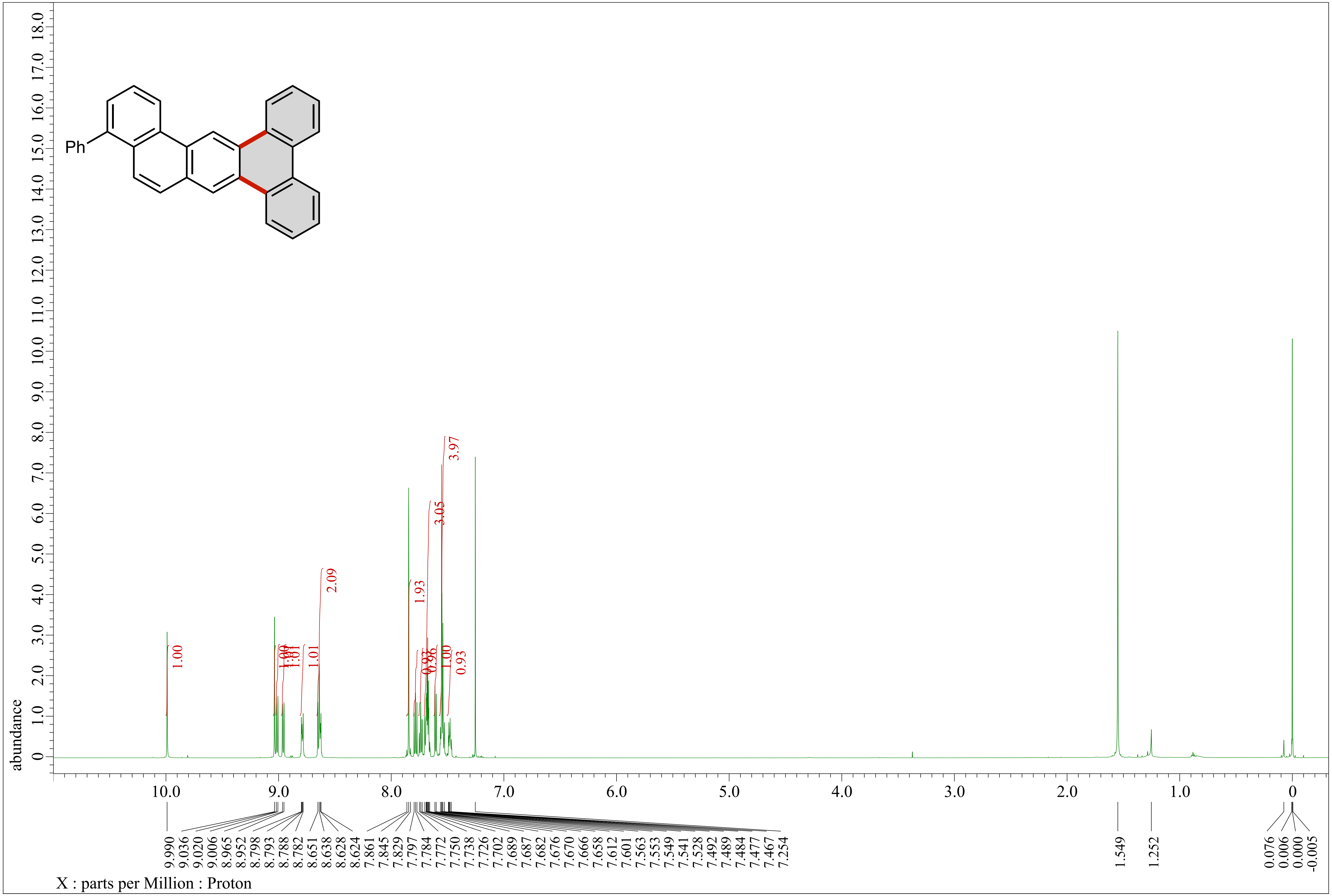

^{1}H NMR spectrum (inset) of **5ia** (solvent: $CDCl_3$)

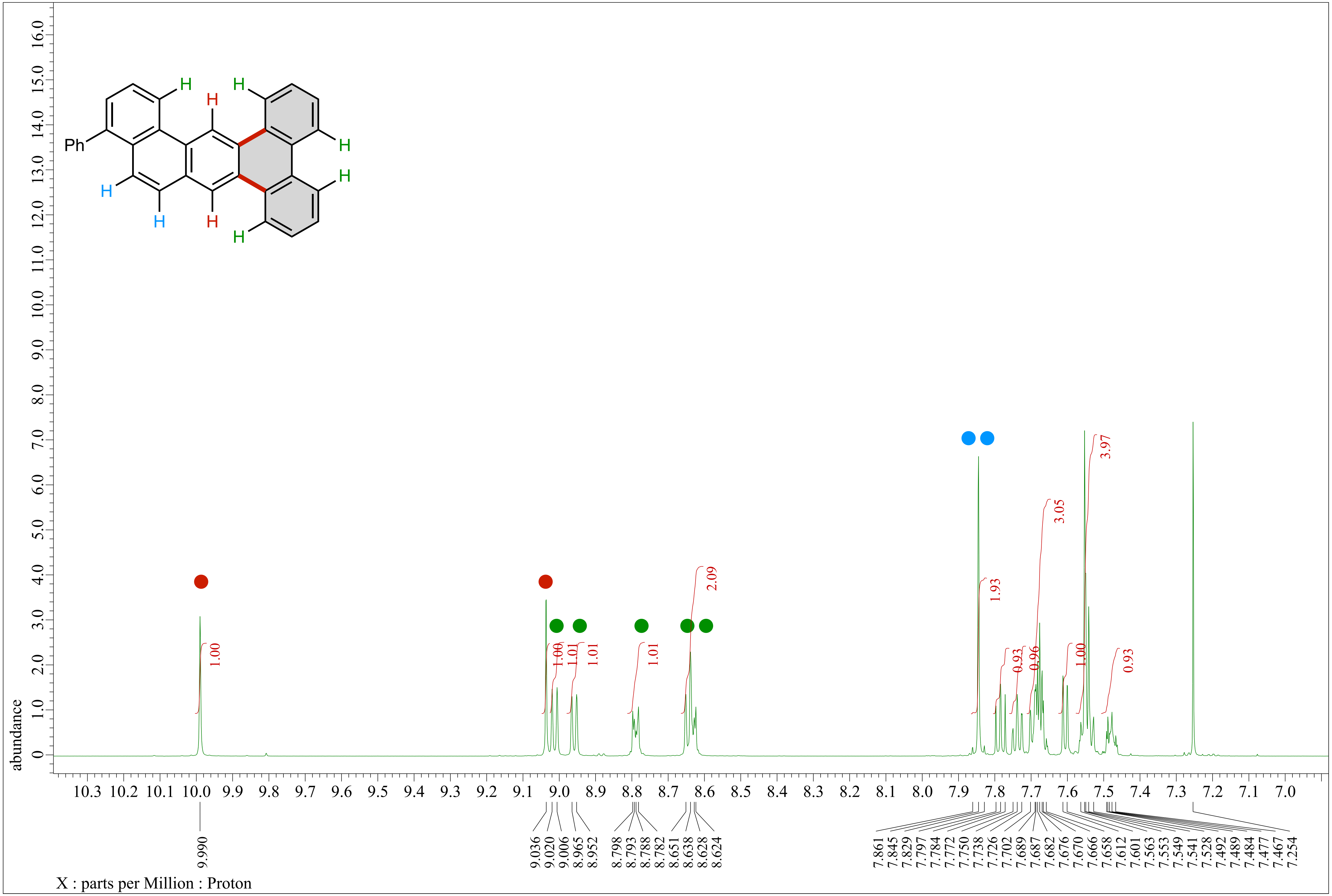

^{13}C NMR spectrum of **5ia** (solvent: $CDCl_3$)

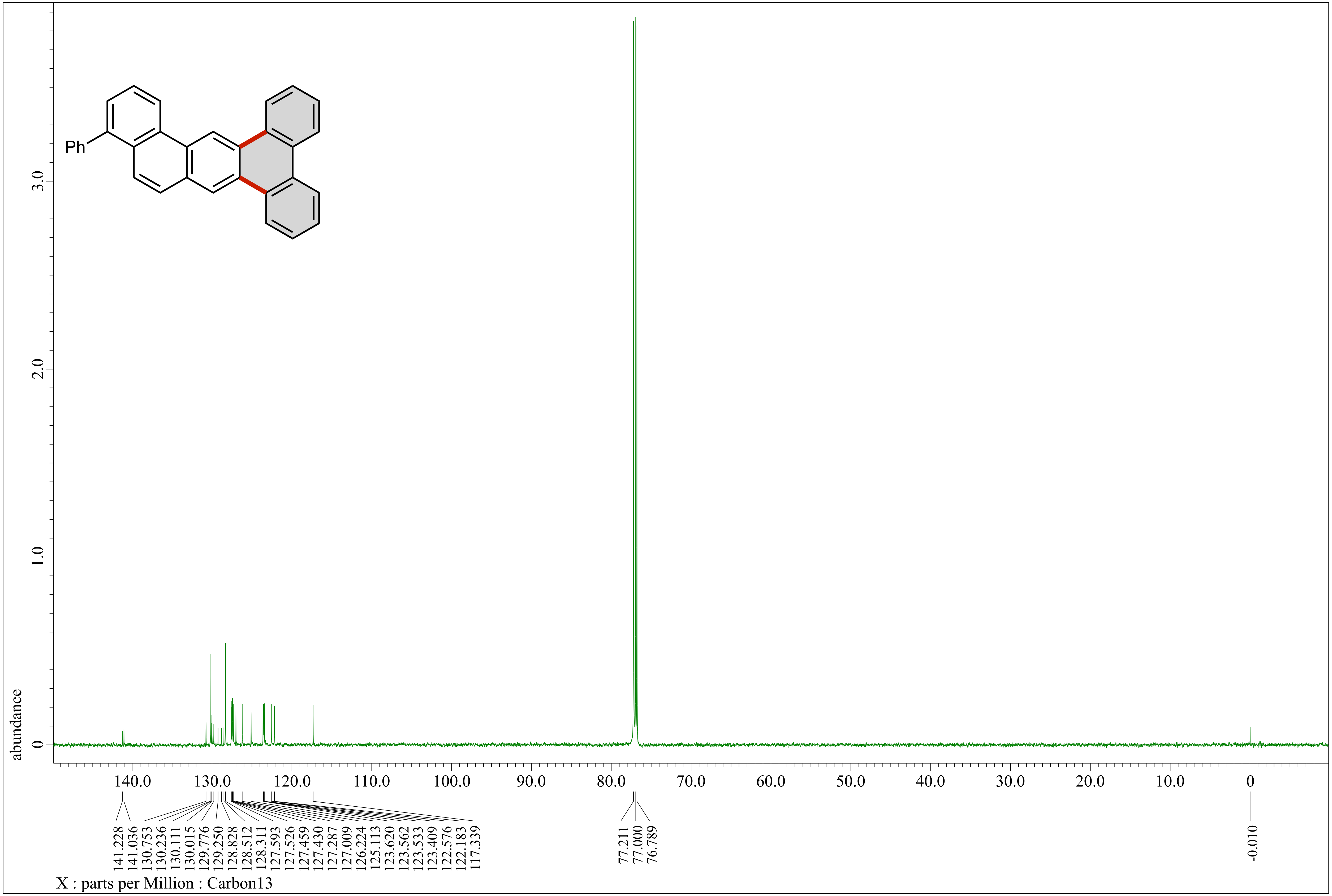

^{13}C NMR spectrum (inset) of **5ia** (solvent: $CDCl_3$)

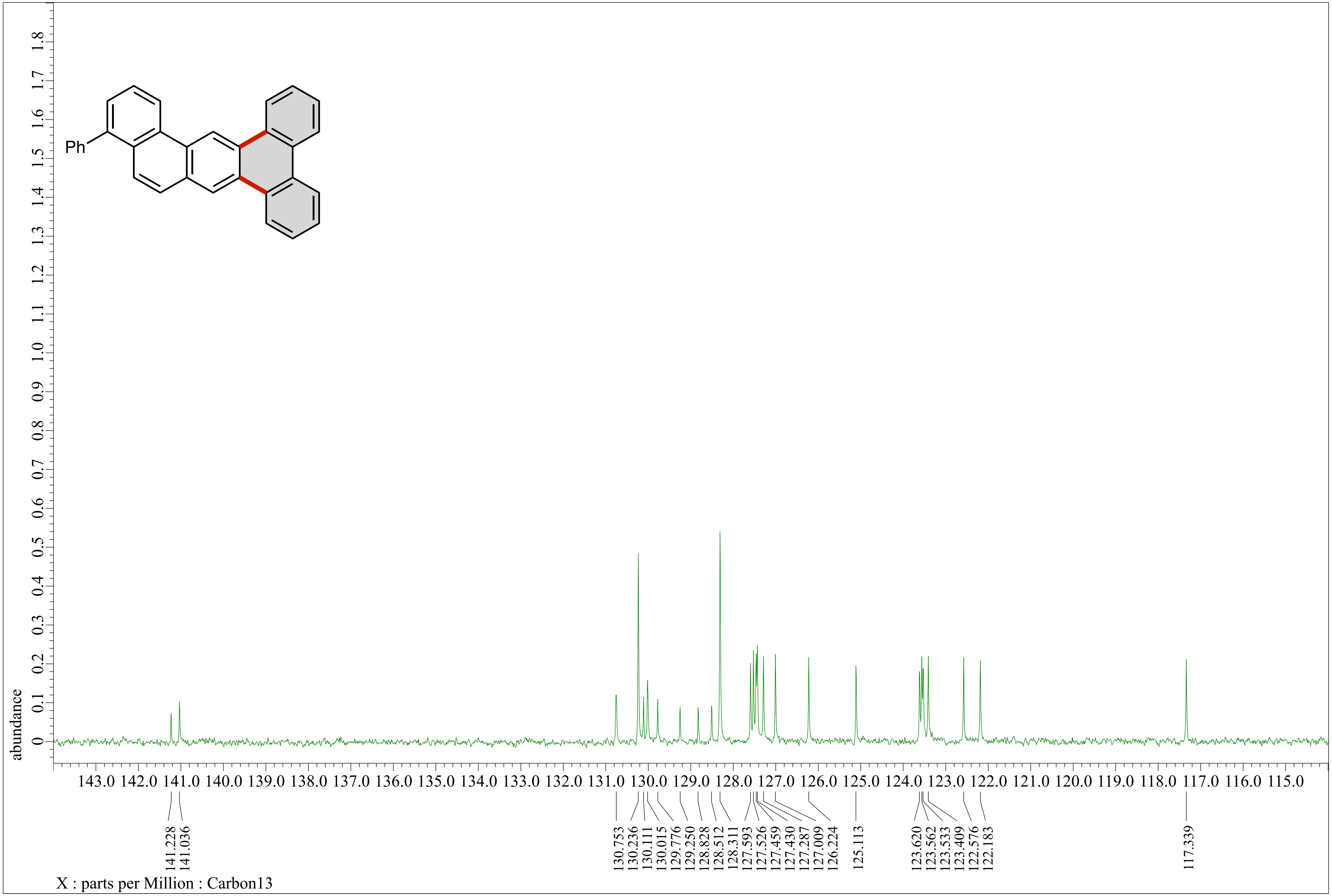

^{1}H NMR spectrum of **5ja** (solvent: $CDCl_3$)

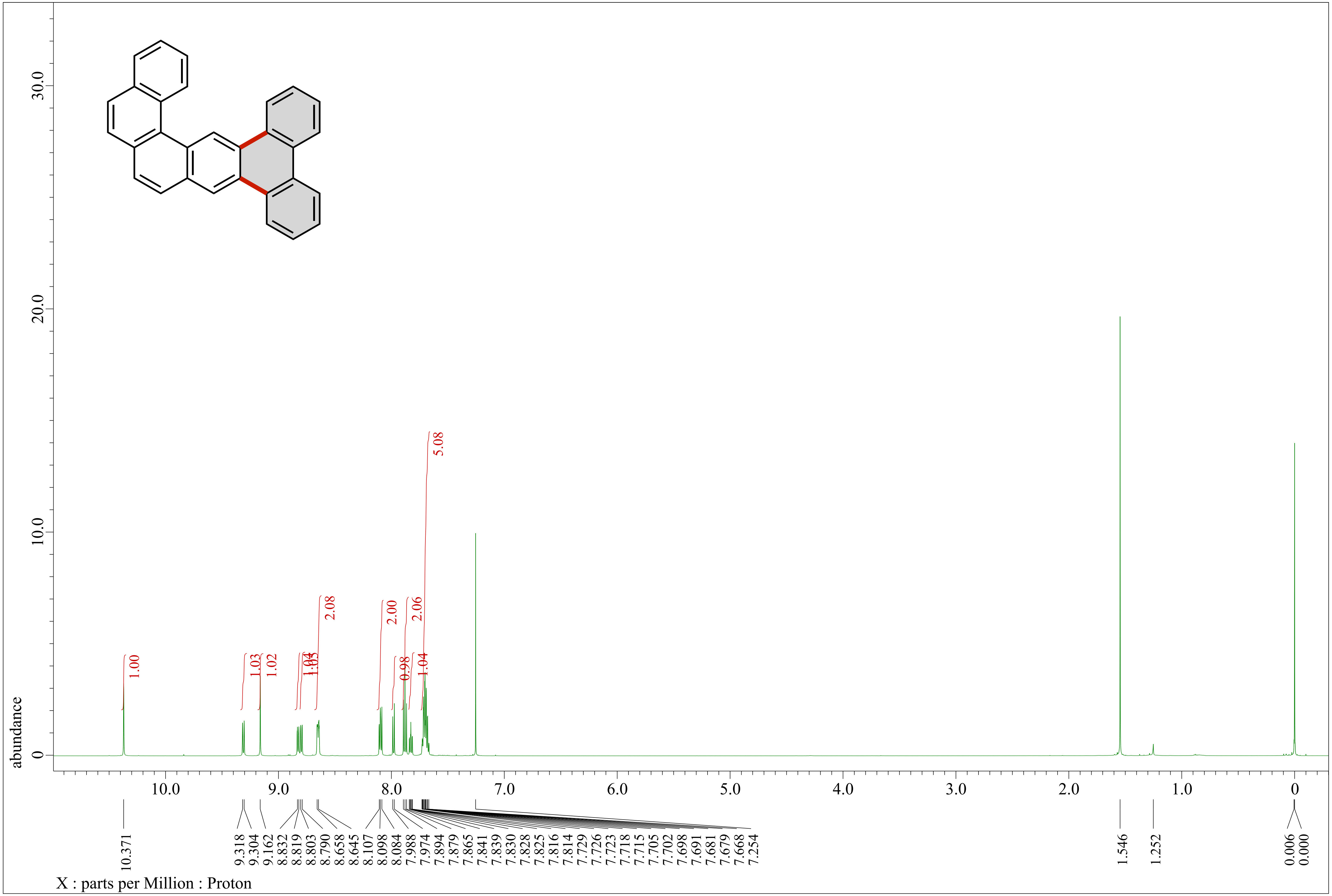

^{1}H NMR spectrum (inset) of **5ja** (solvent: $CDCl_3$)

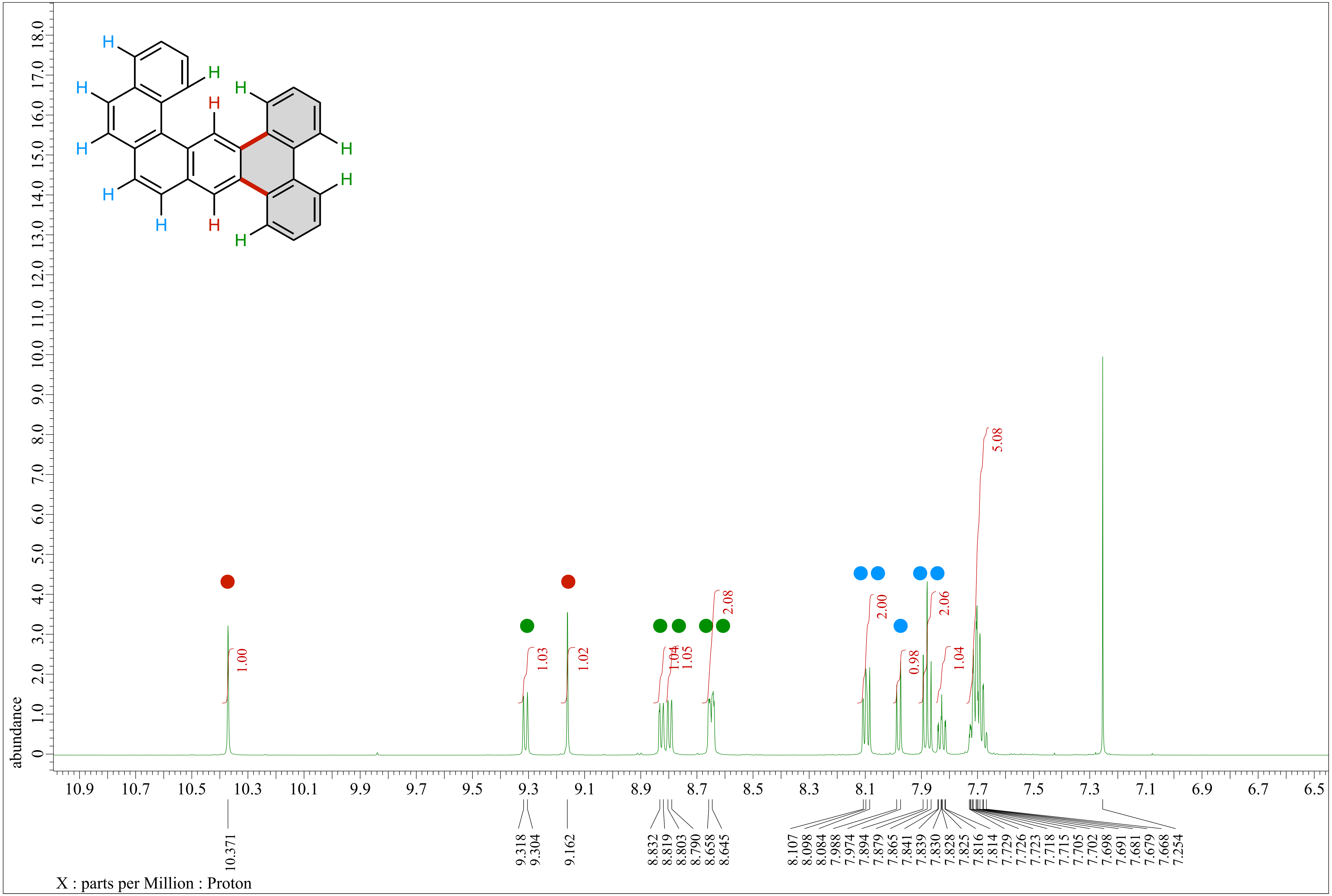

^{13}C NMR spectrum of **5ja** (solvent: $CDCl_3$)

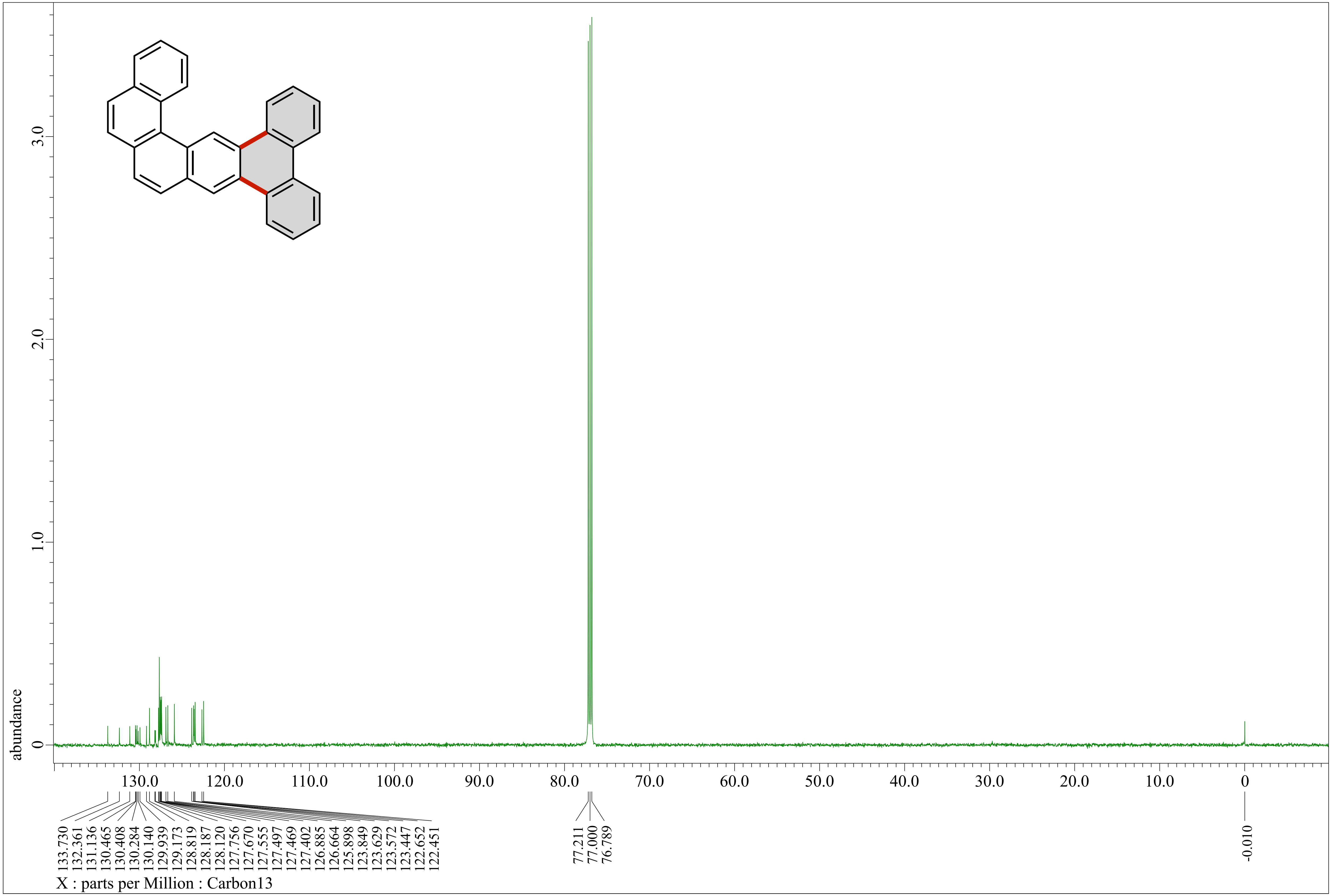

^{13}C NMR spectrum (inset) of **5ja** (solvent: $CDCl_3$)

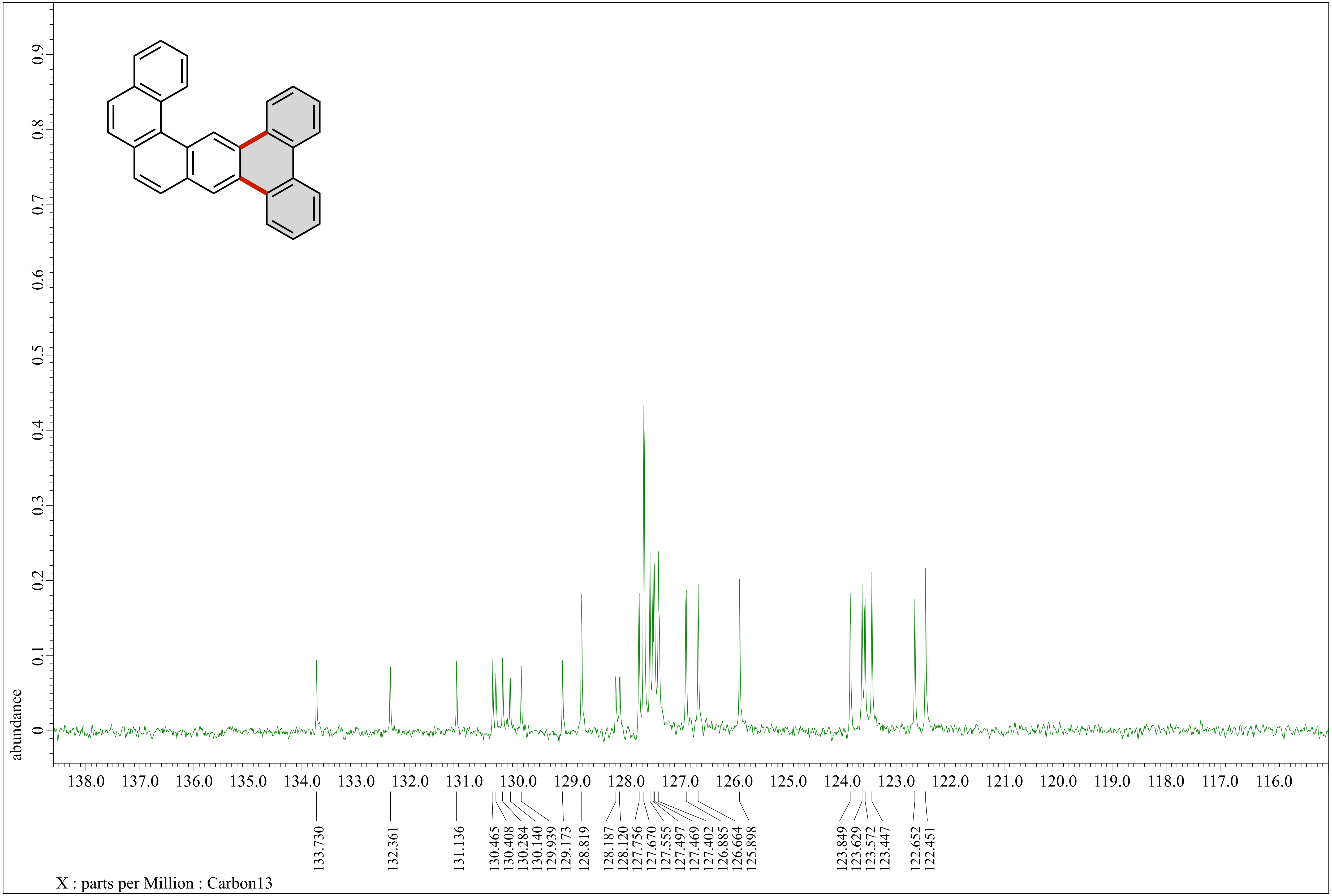

^{1}H NMR spectrum of **5ka** (solvent: $CDCl_3$)

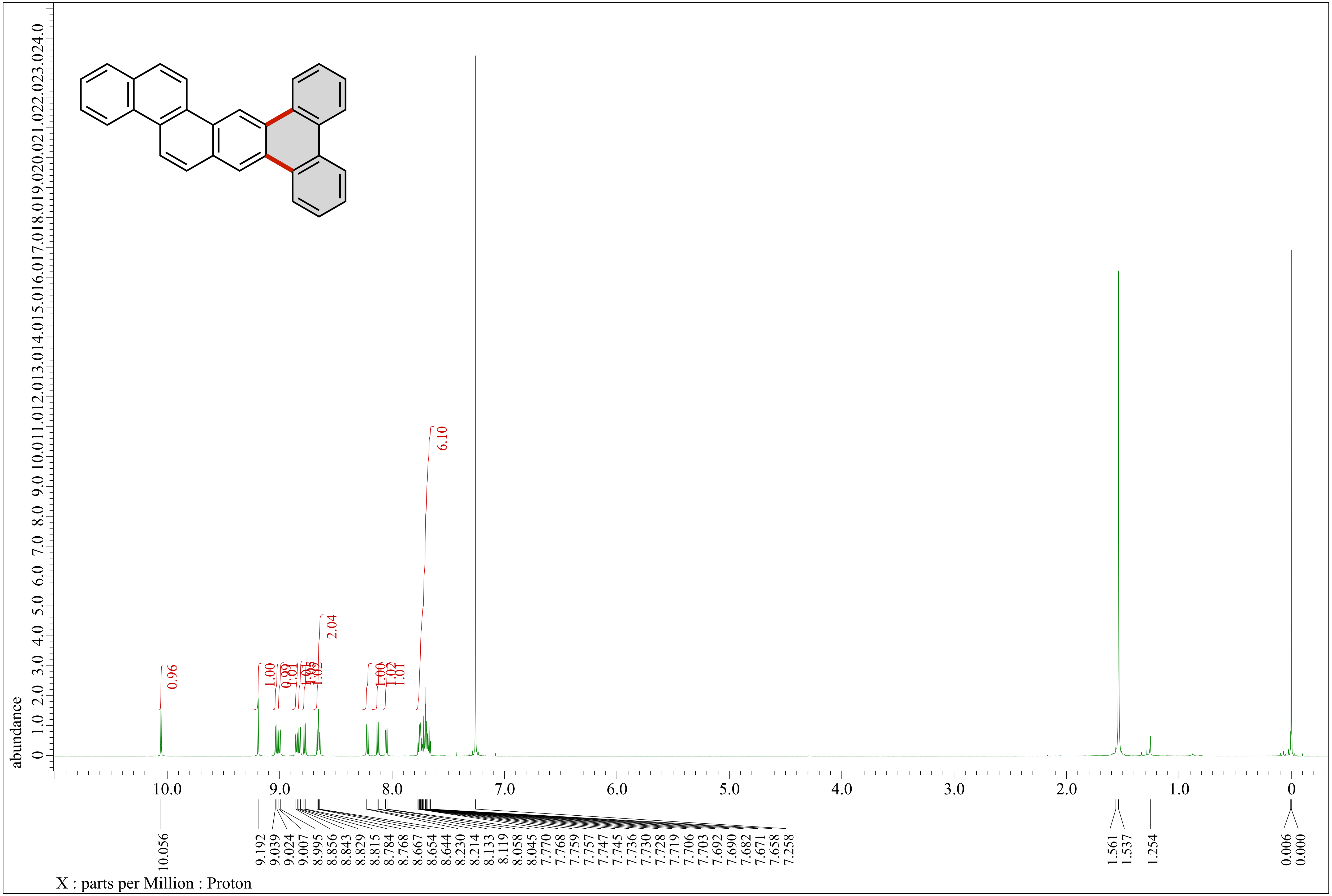

^{1}H NMR spectrum (inset) of **5ka** (solvent: $CDCl_3$)

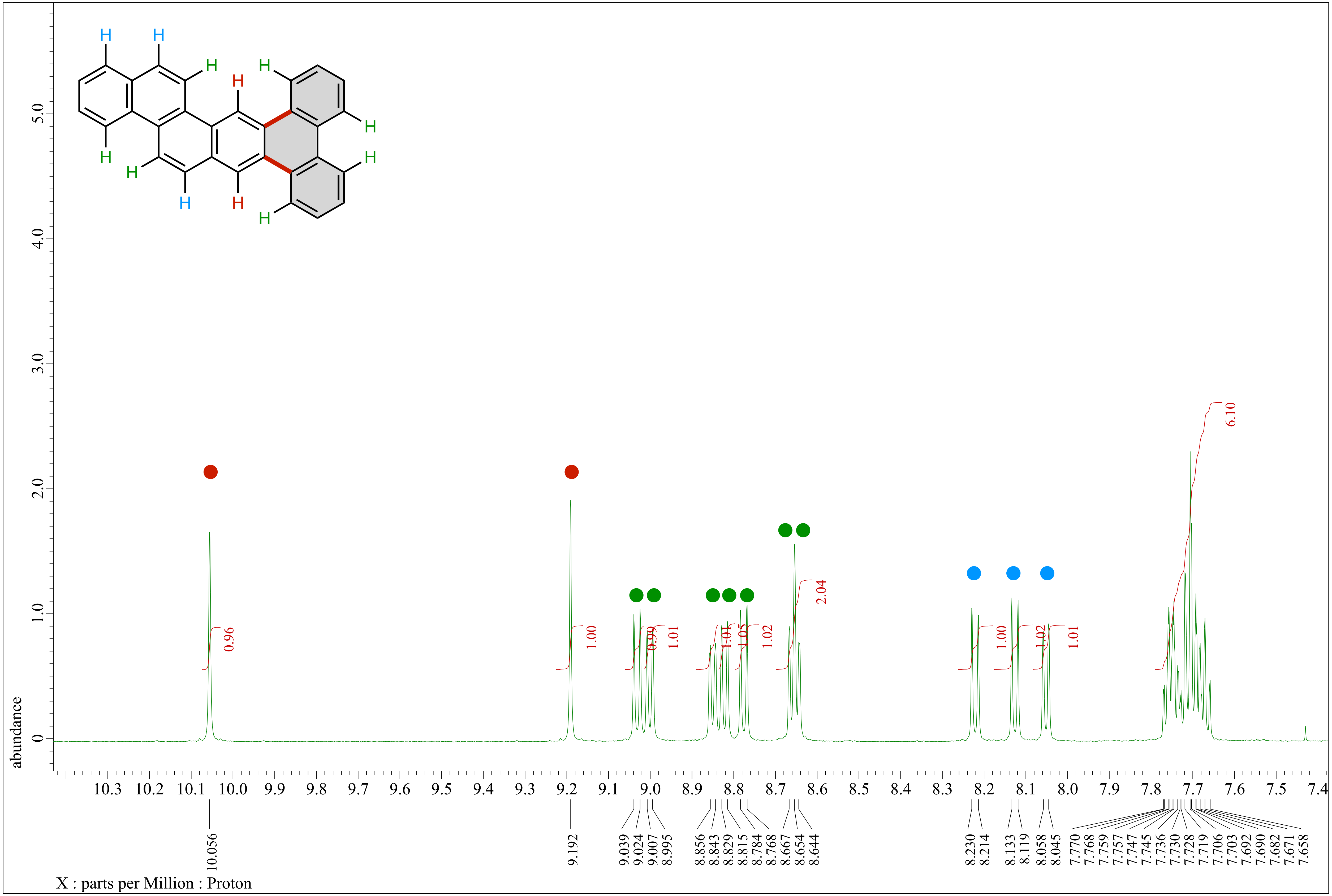

^{13}C NMR spectrum of **5ka** (solvent: $CDCl_3$)

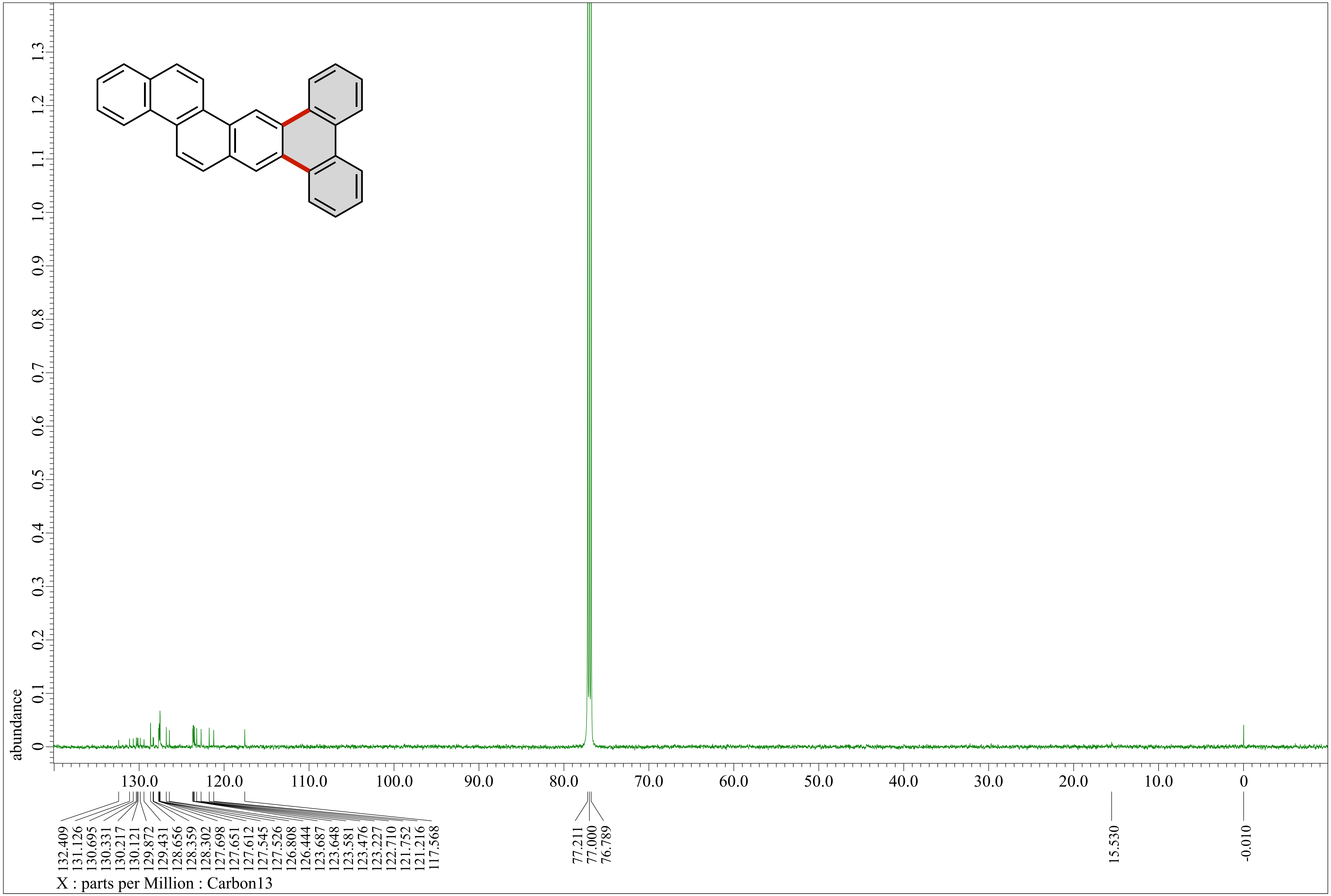

^{13}C NMR spectrum (inset) of **5ka** (solvent: $CDCl_3$)

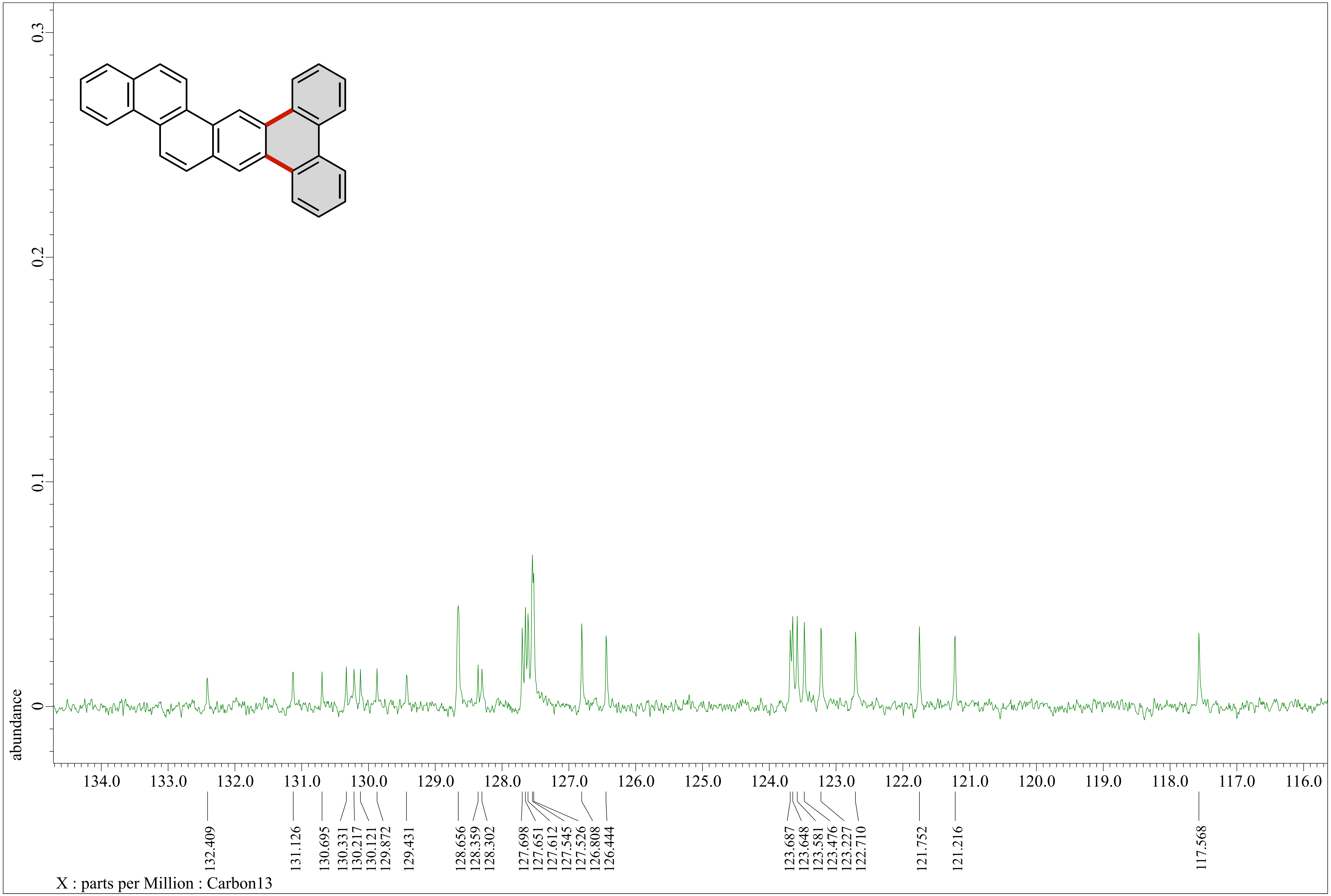

^{1}H NMR spectrum of **5la** (solvent: $CDCl_3$)

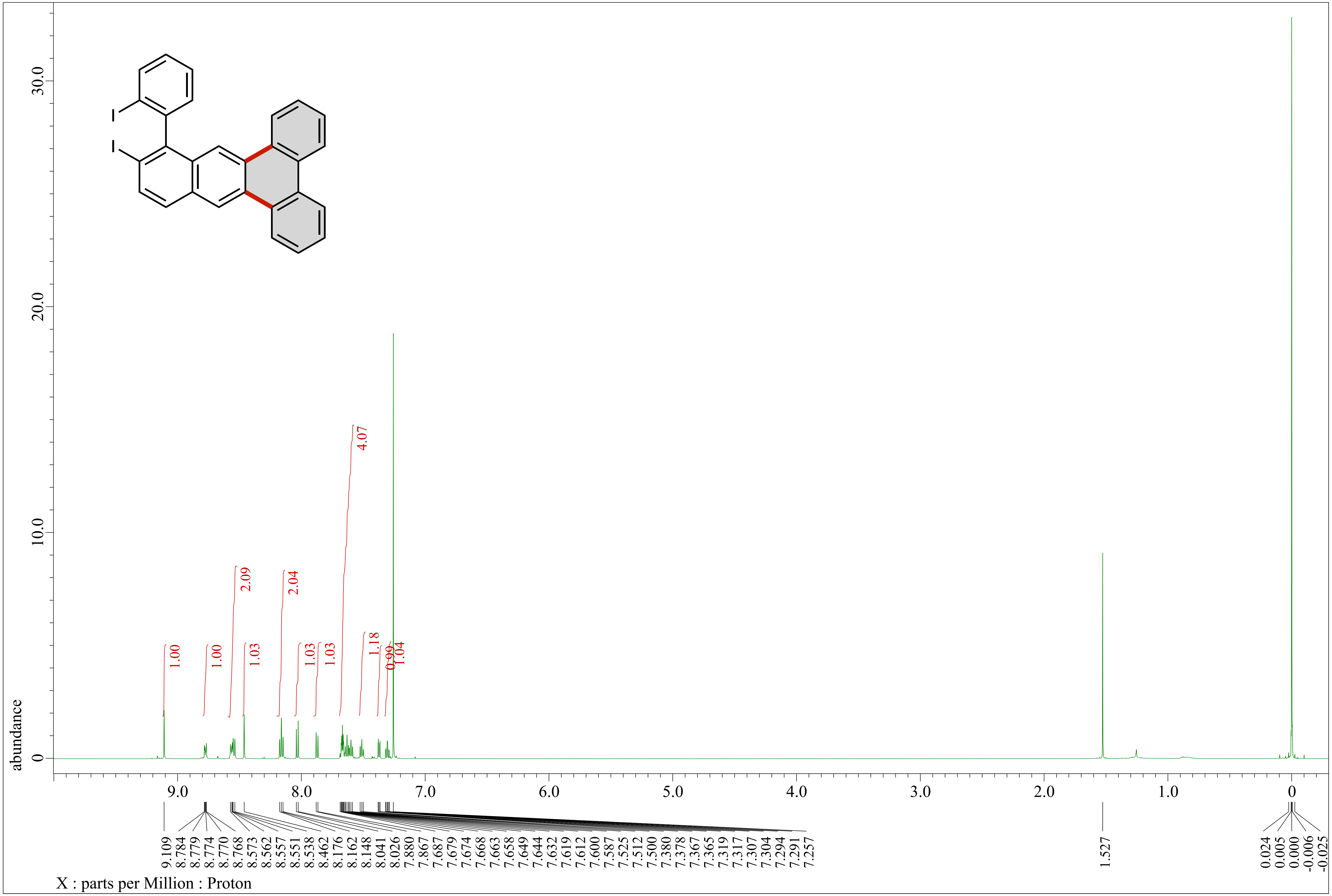

^{1}H NMR spectrum (inset) of **5la** (solvent: $CDCl_3$)

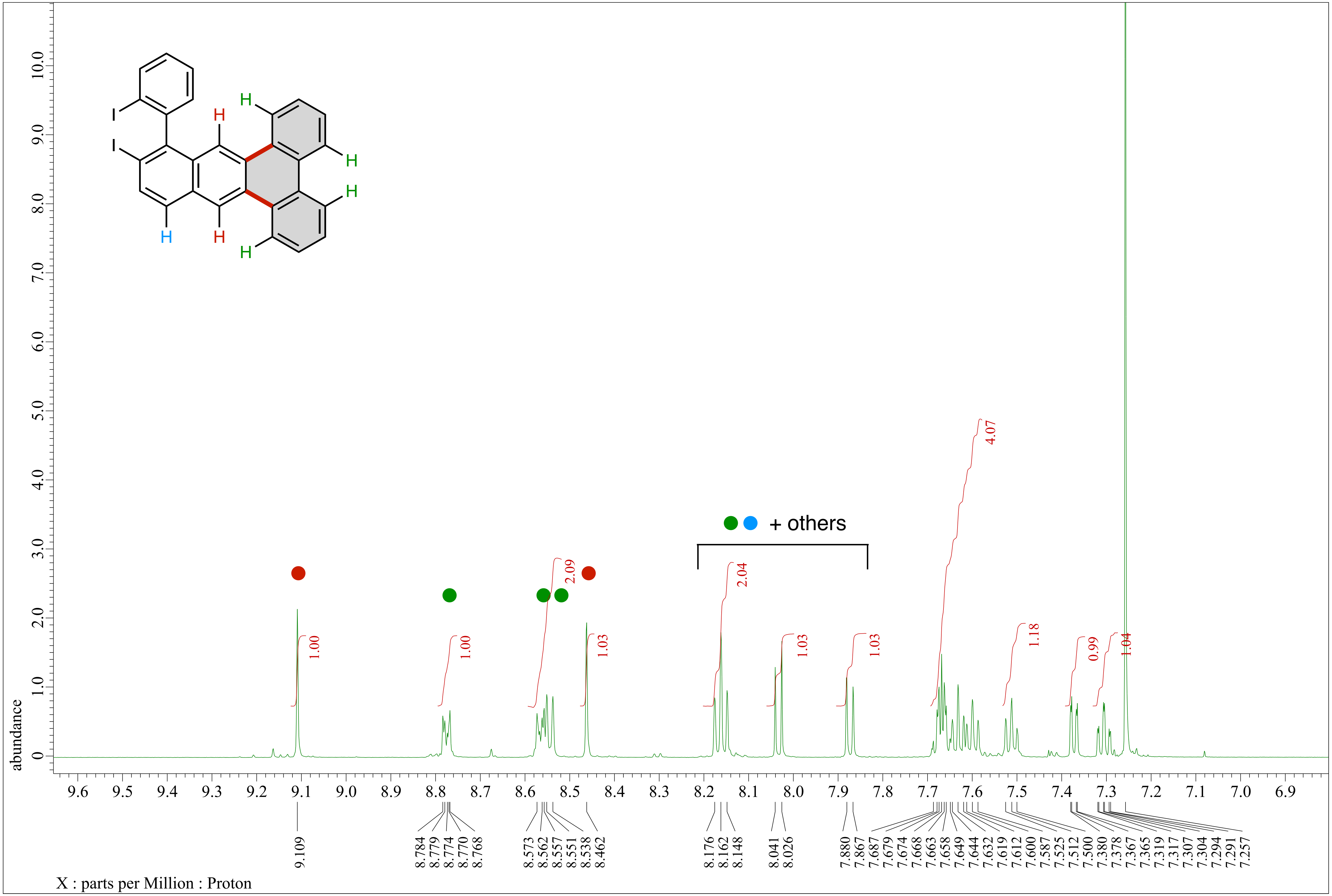

^{13}C NMR spectrum of **5la** (solvent: $CDCl_3$)

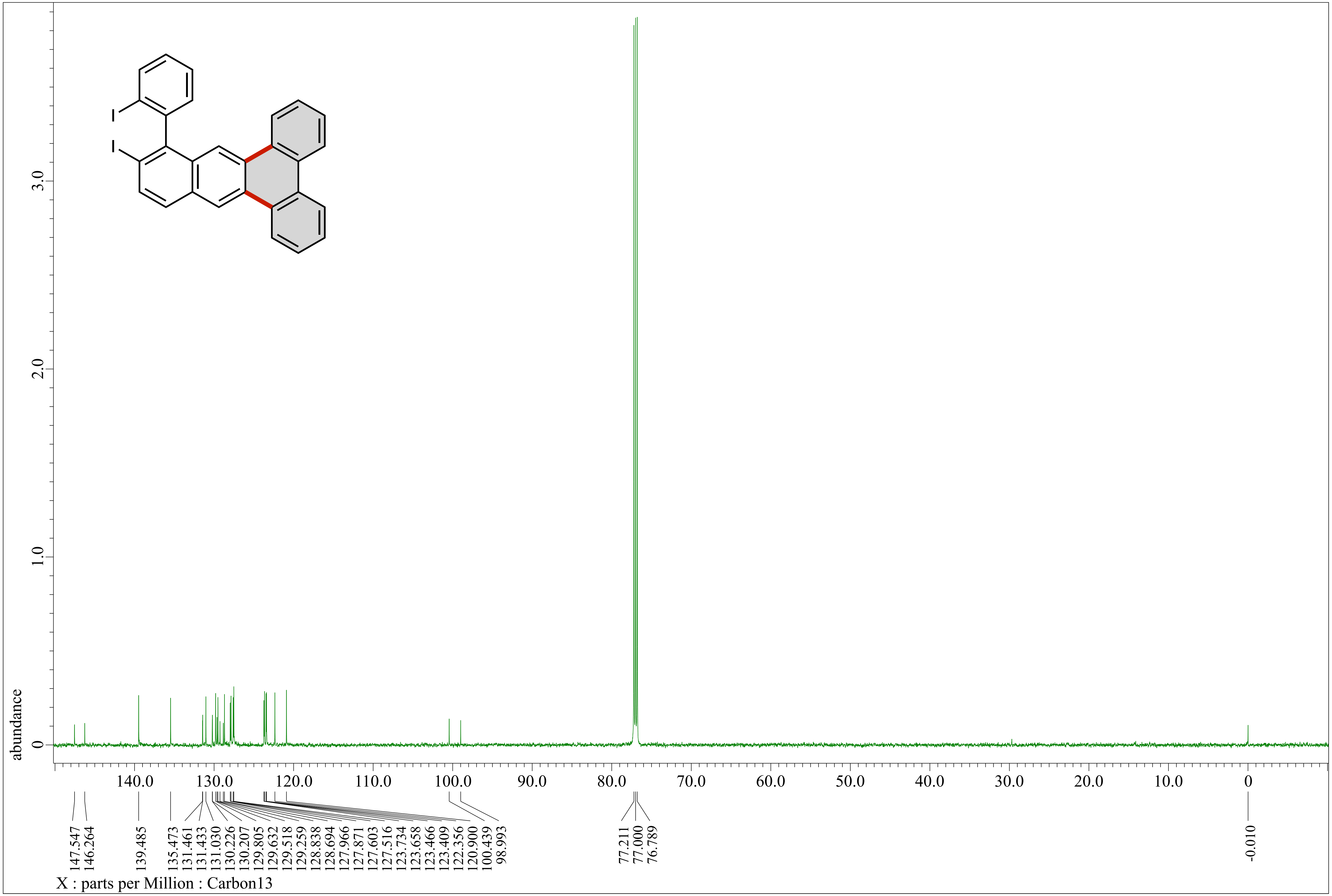

^{13}C NMR spectrum (inset) of **5la** (solvent: $CDCl_3$)

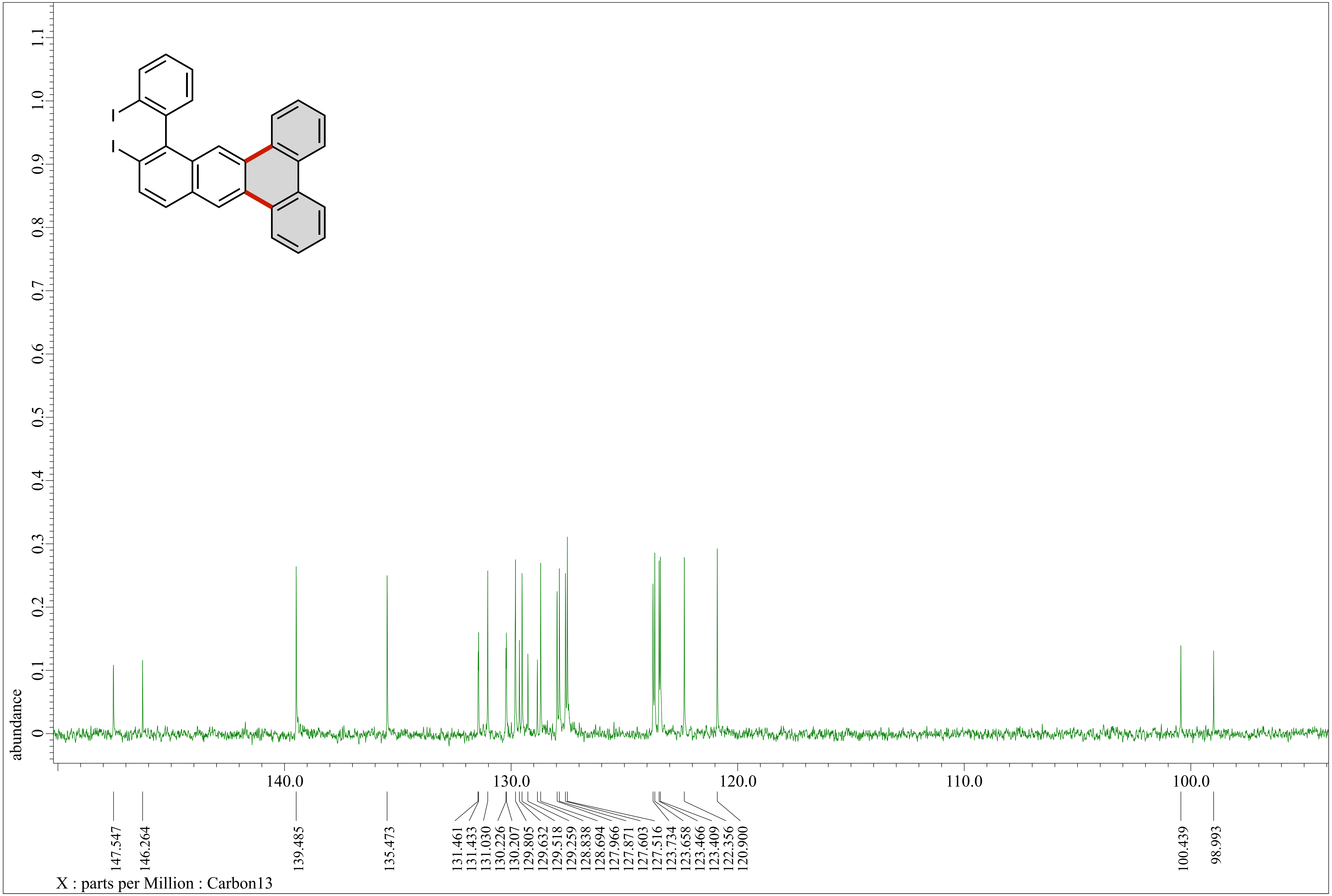

^1H NMR spectrum of **5ma** (solvent: $CDCl_3$)

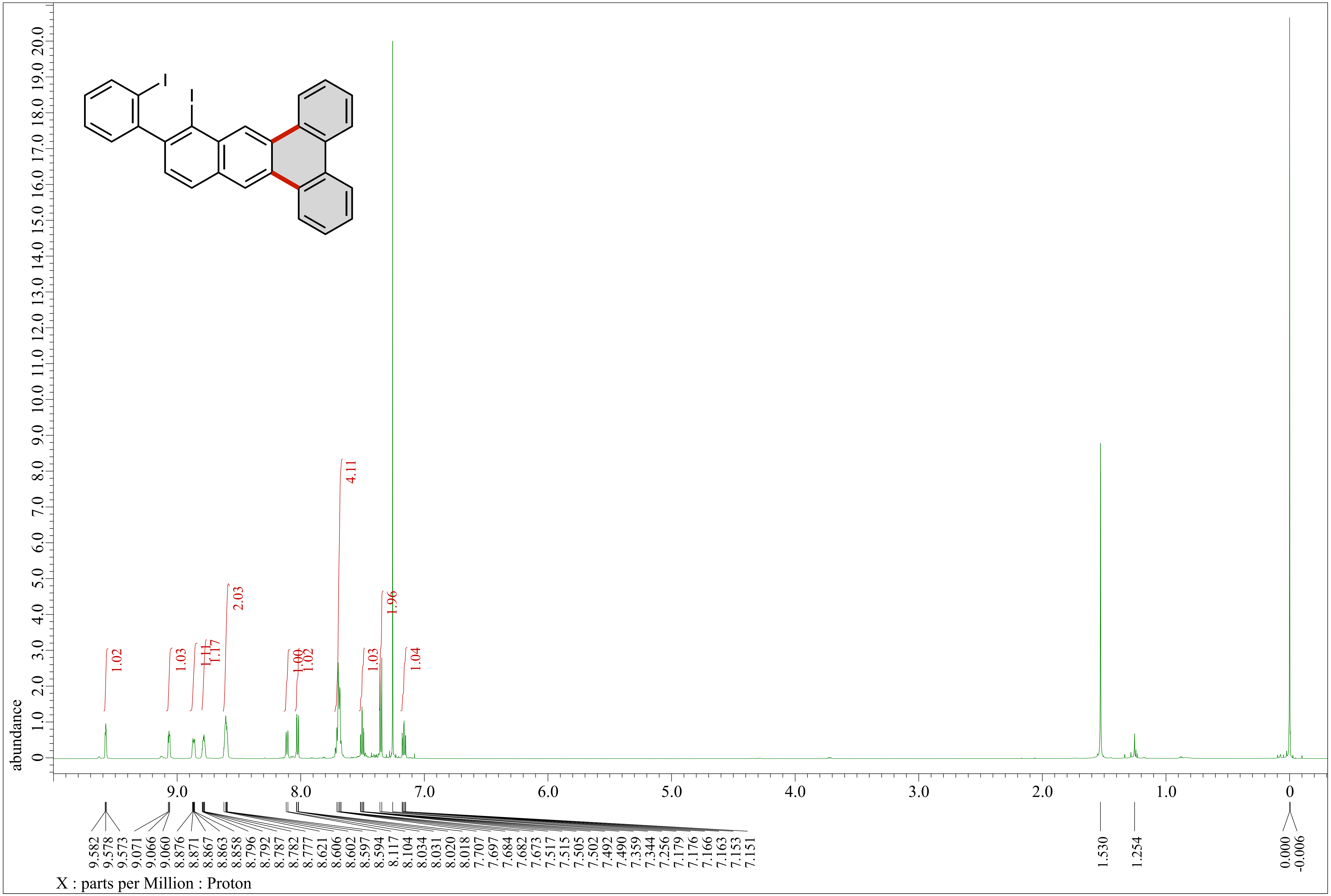

^{1}H NMR spectrum (inset) of **5ma** (solvent: $CDCl_3$)

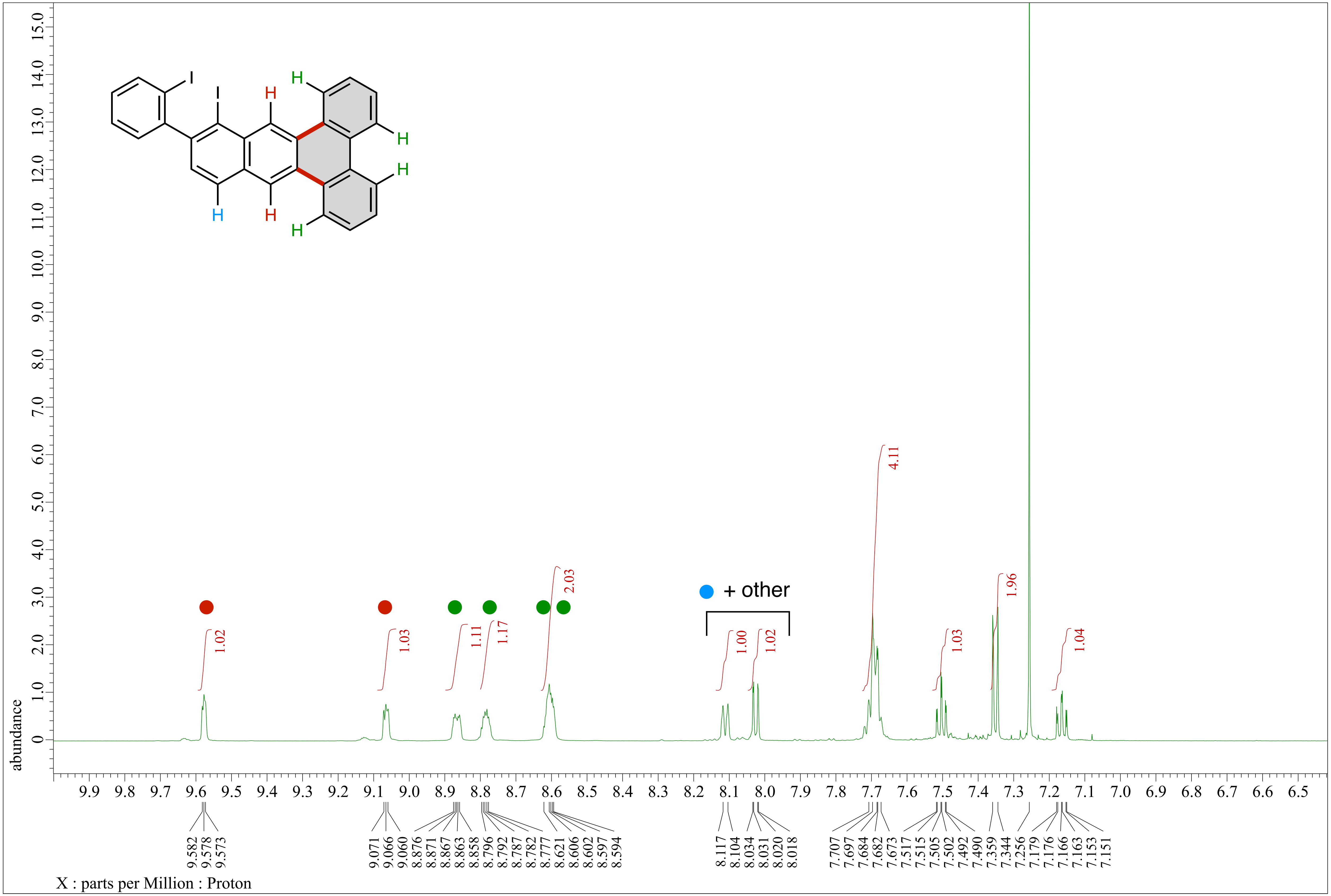

^{13}C NMR spectrum of **5ma** (solvent: $CDCl_3$)

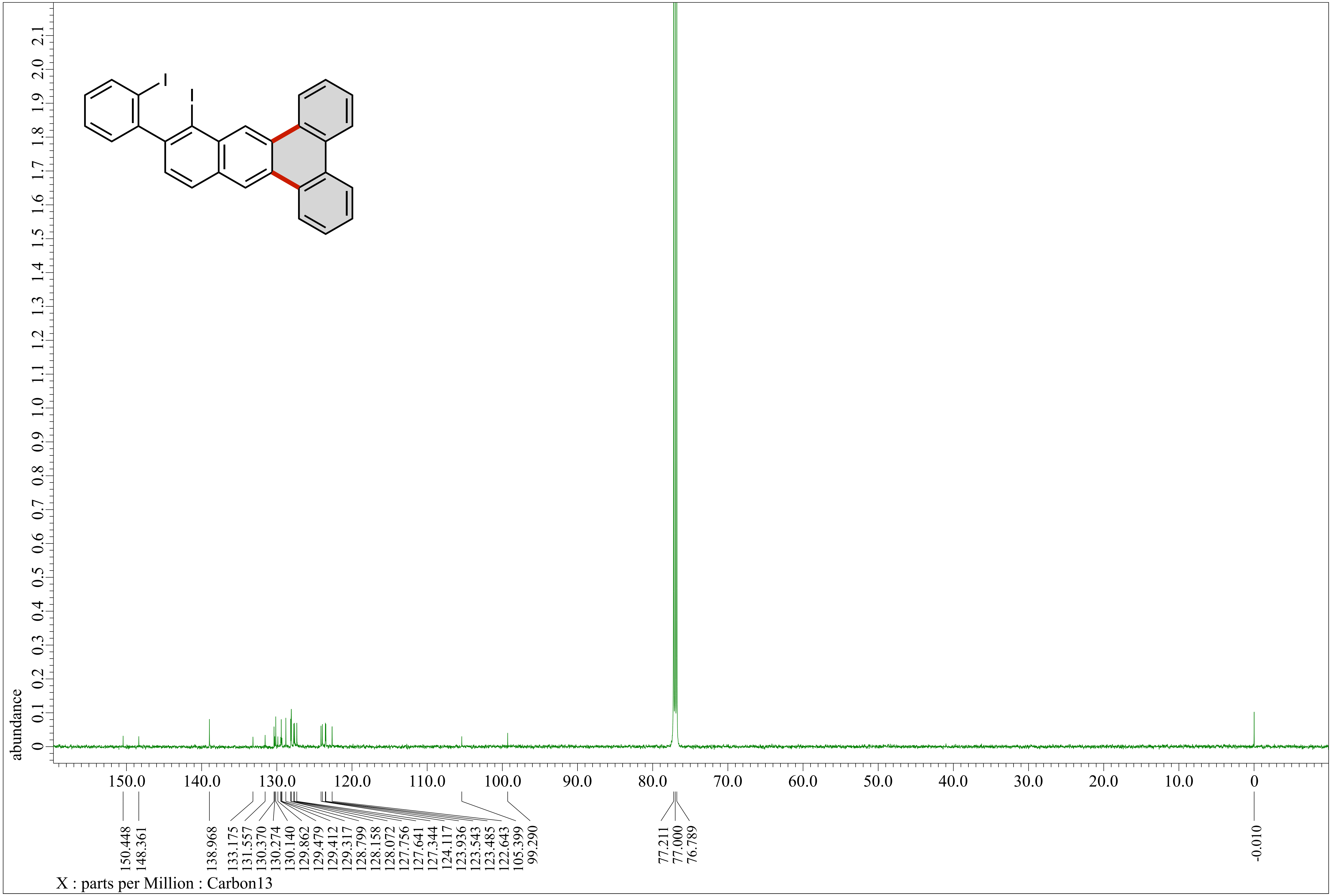

^{13}C NMR spectrum (inset) of **5ma** (solvent: $CDCl_3$)

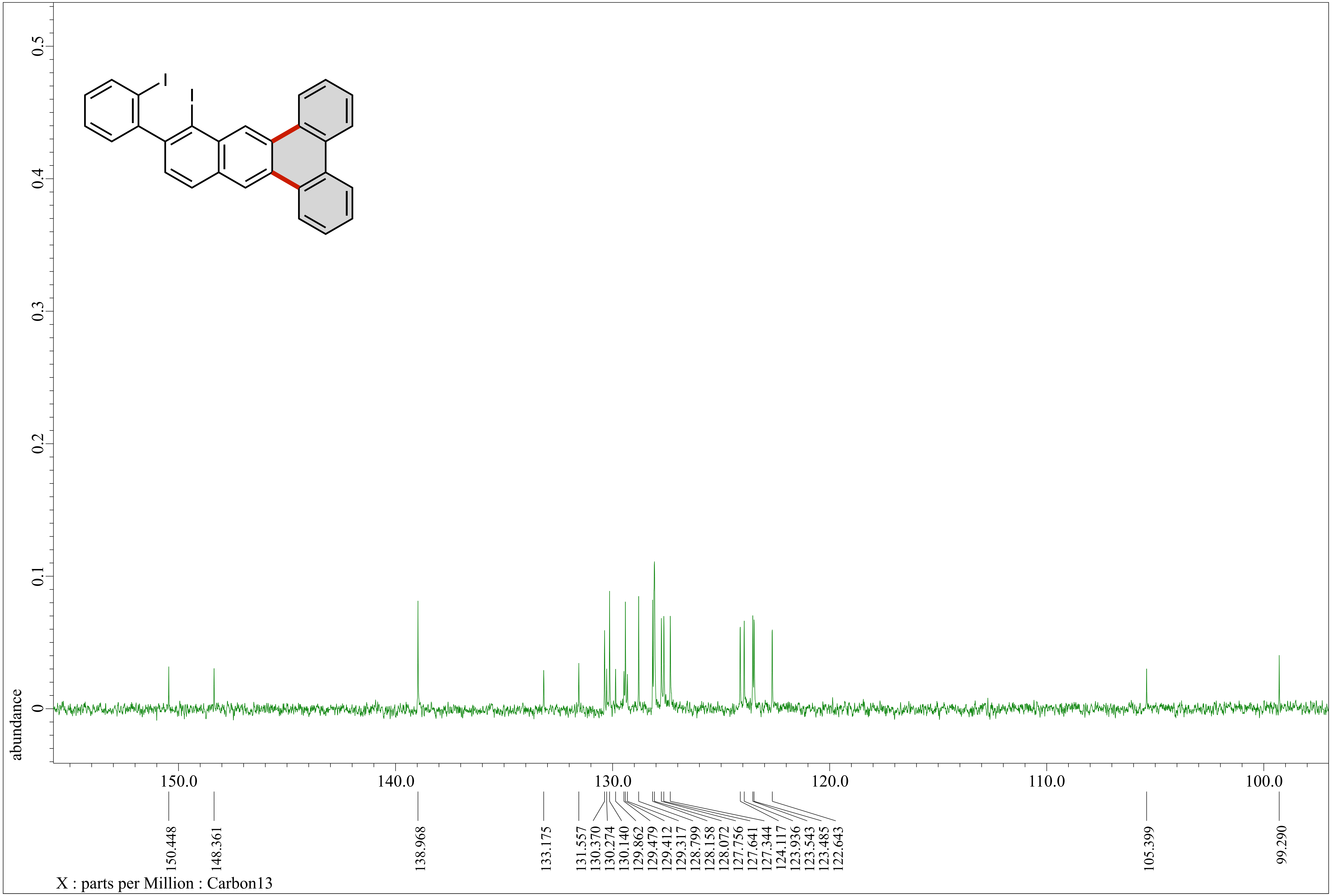

^{1}H NMR spectrum of **5na** (solvent: $CDCl_3$)

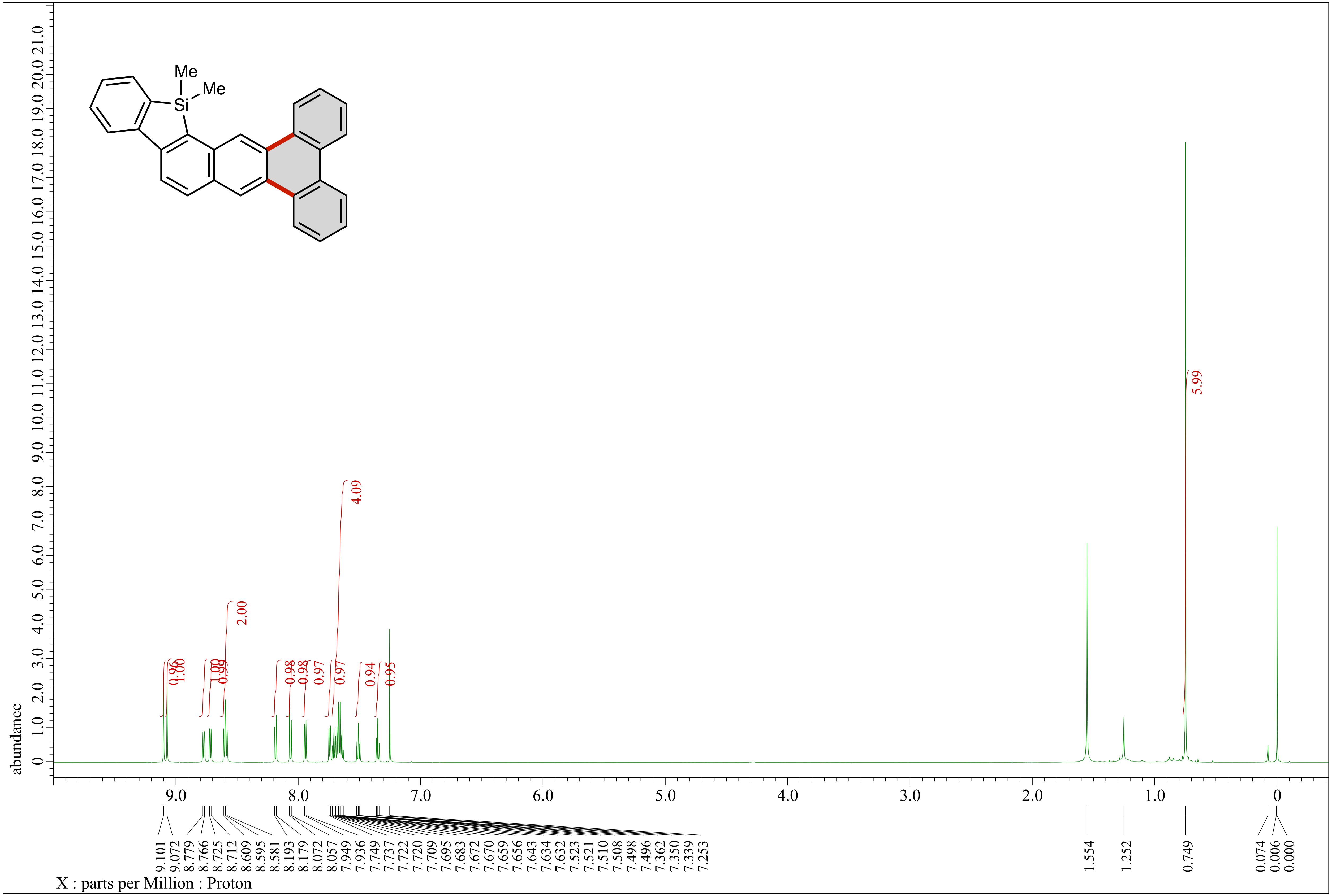

^1H NMR spectrum (inset) of **5na** (solvent: $CDCl_3$)

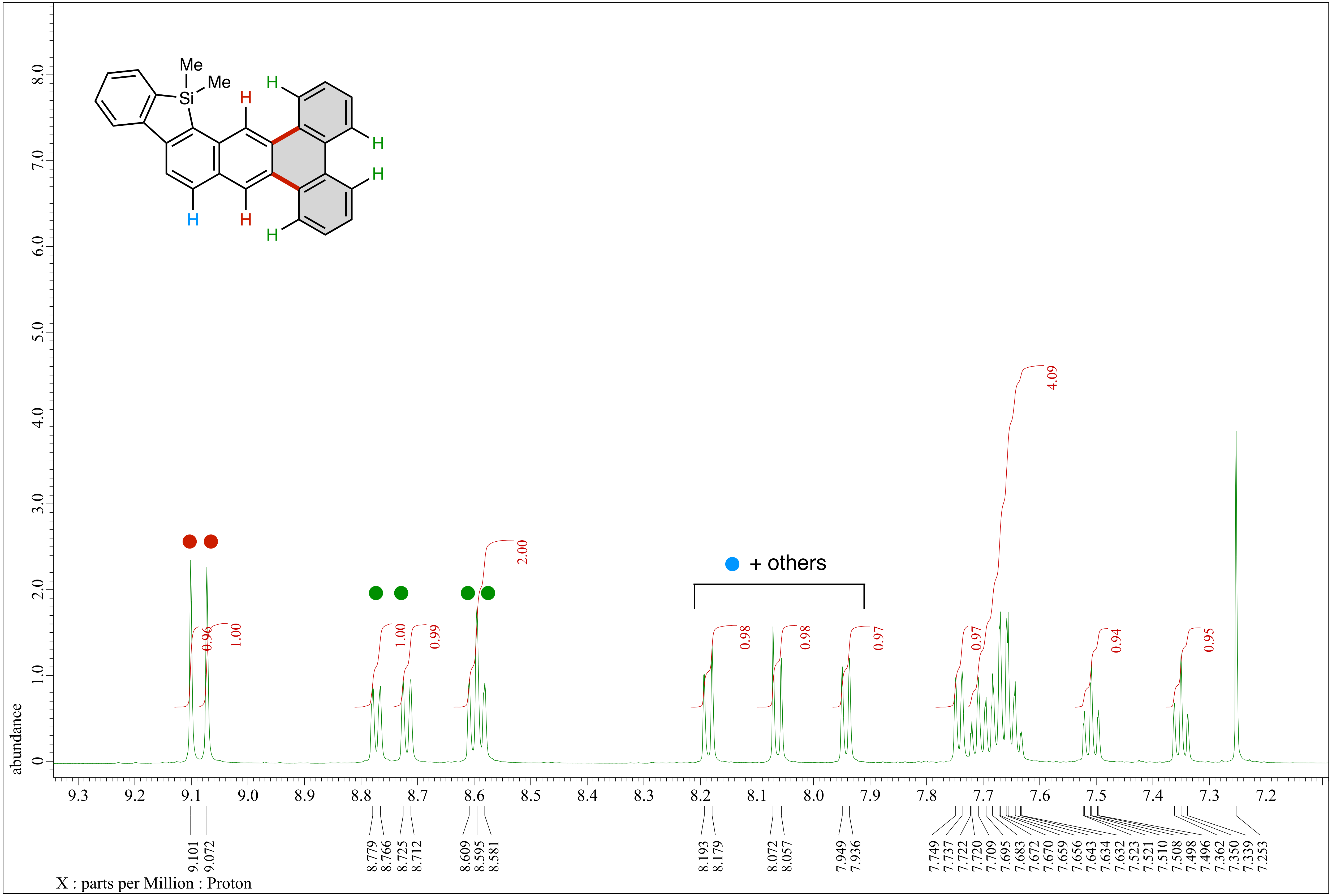

^{13}C NMR spectrum of **5na** (solvent: $CDCl_3$)

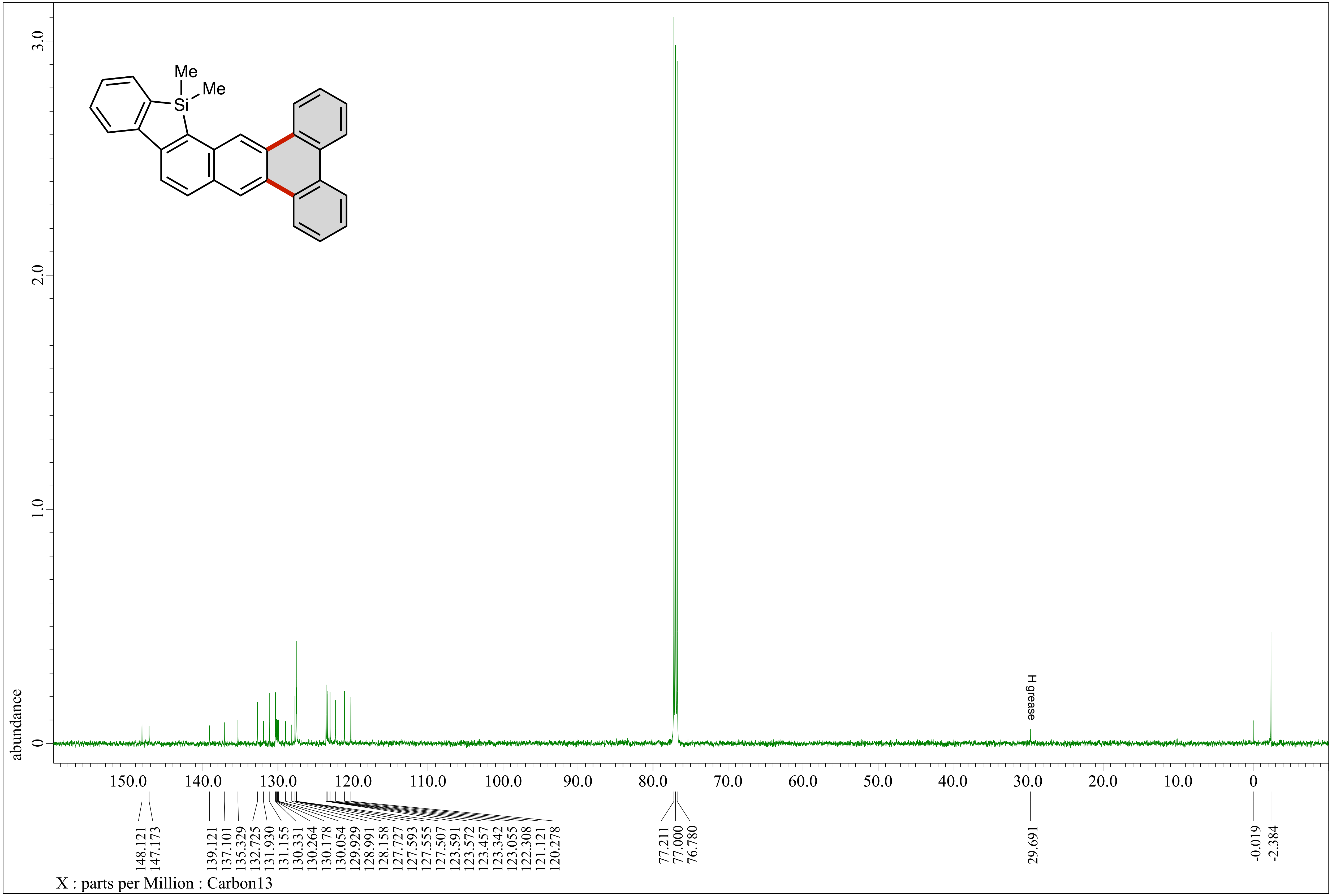

^{13}C NMR spectrum (inset) of **5na** (solvent: $CDCl_3$)

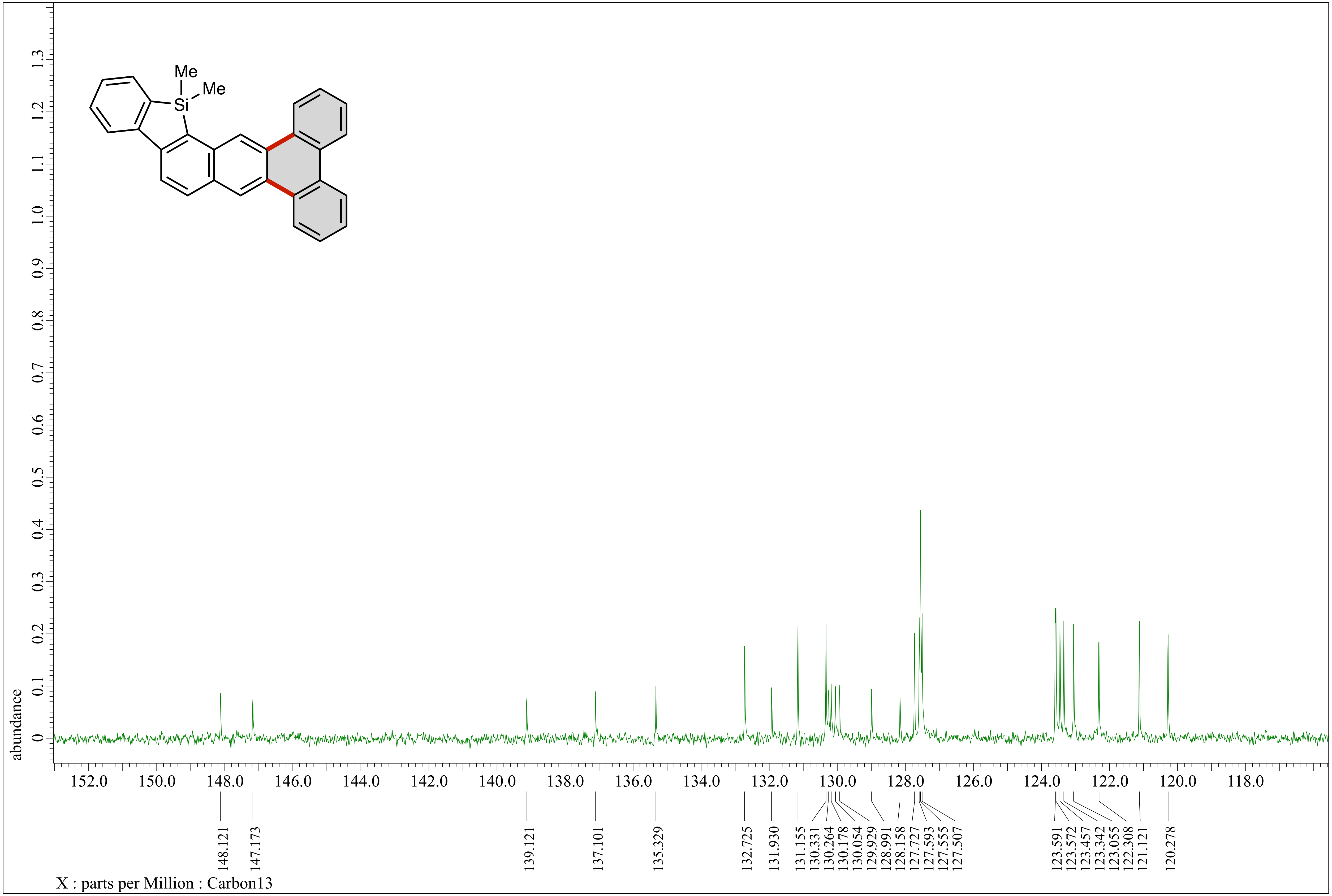

^{1}H NMR spectrum of **5oa** (solvent: $CDCl_3$)

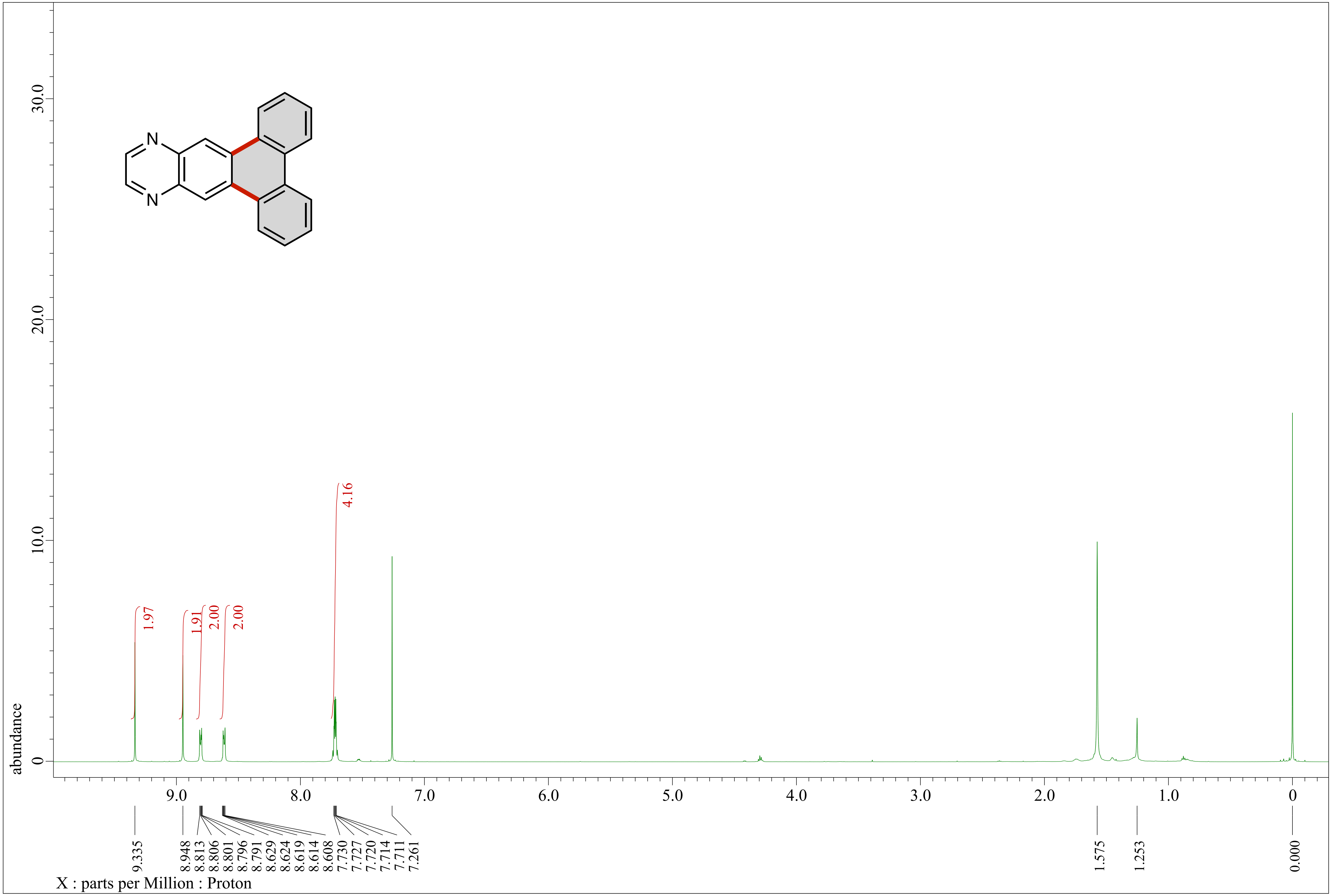

^{1}H NMR spectrum (inset) of **5oa** (solvent: $CDCl_3$)

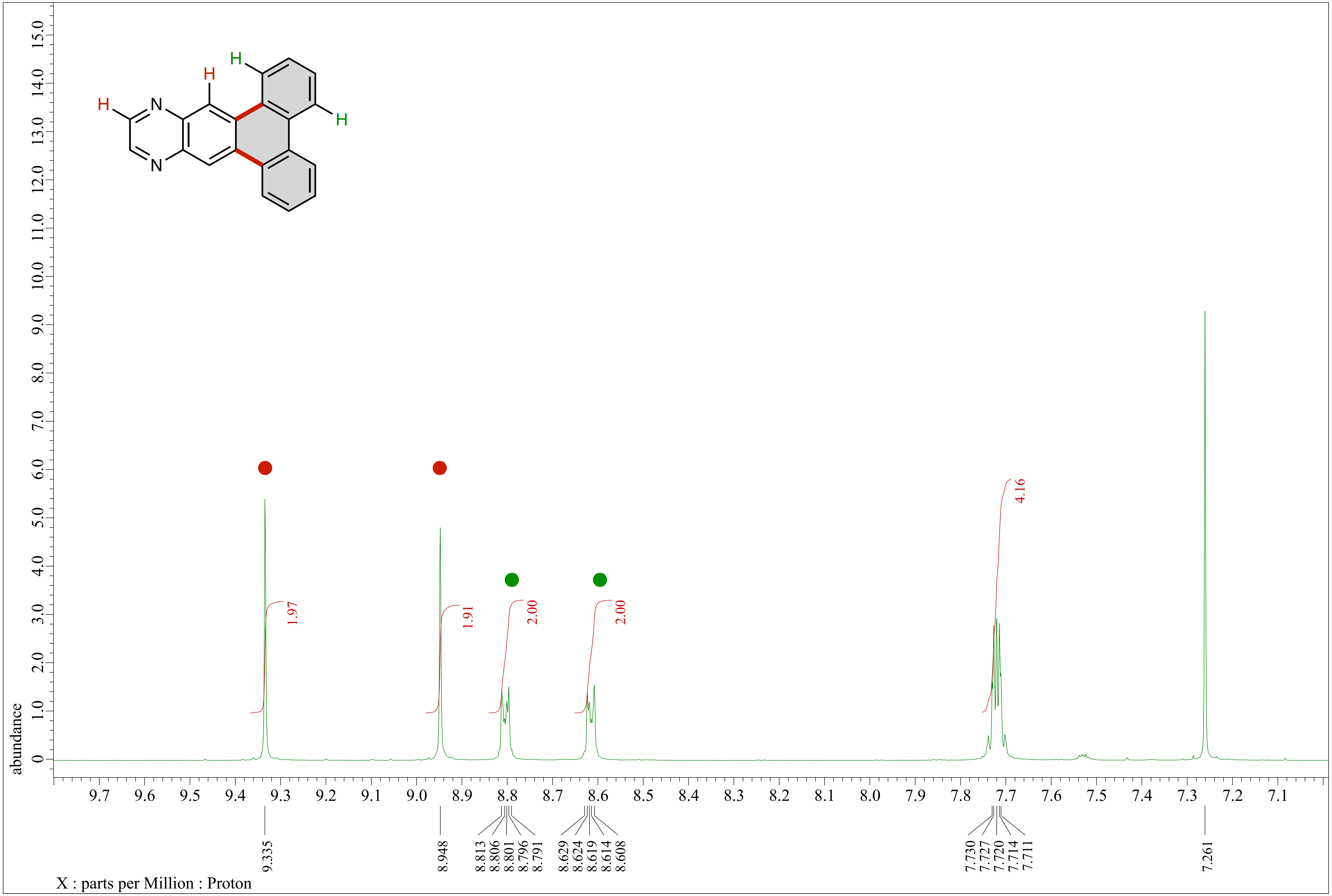

^{13}C NMR spectrum of **5oa** (solvent: $CDCl_3$)

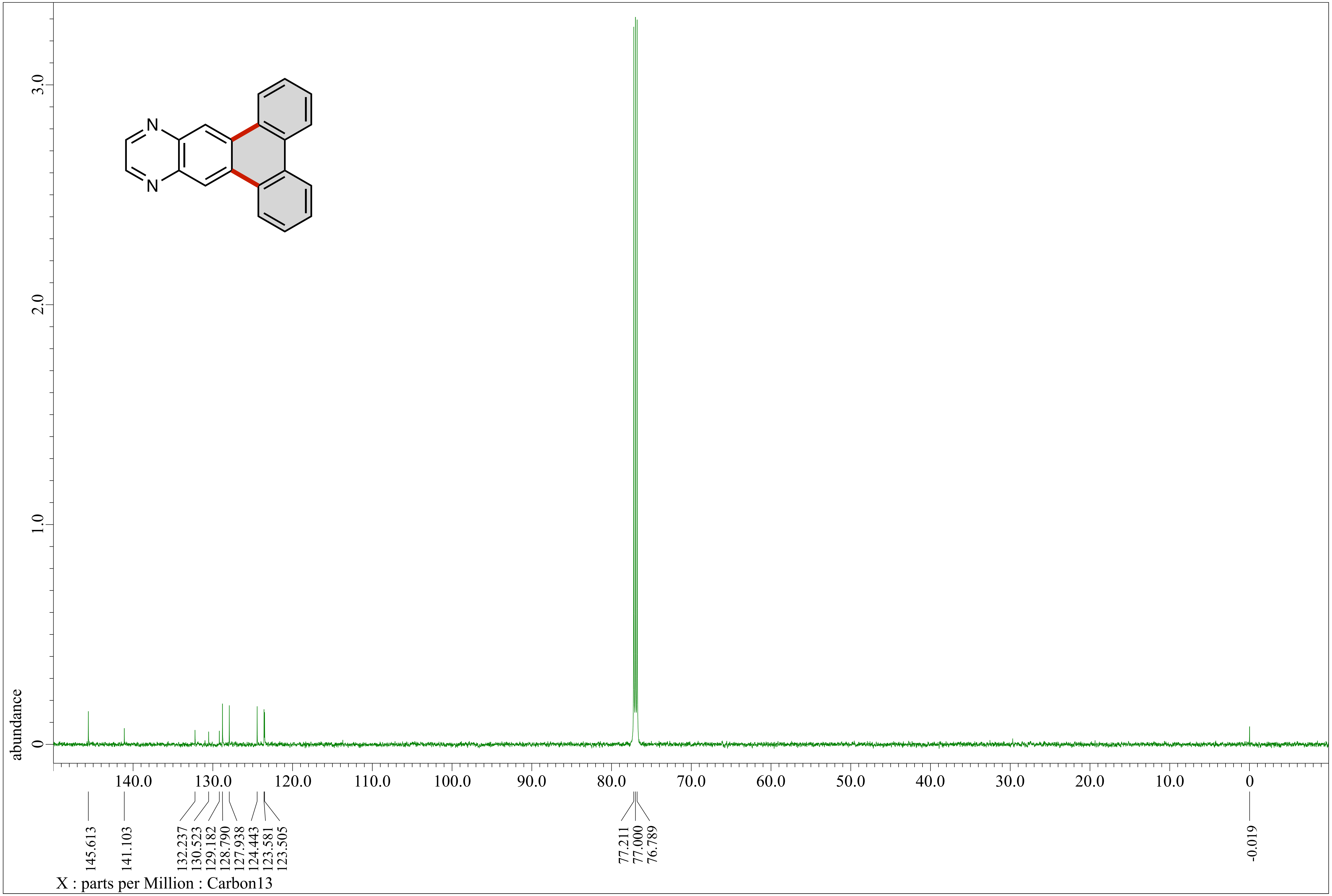

^{13}C NMR spectrum (inset) of **5oa** (solvent: $CDCl_3$)

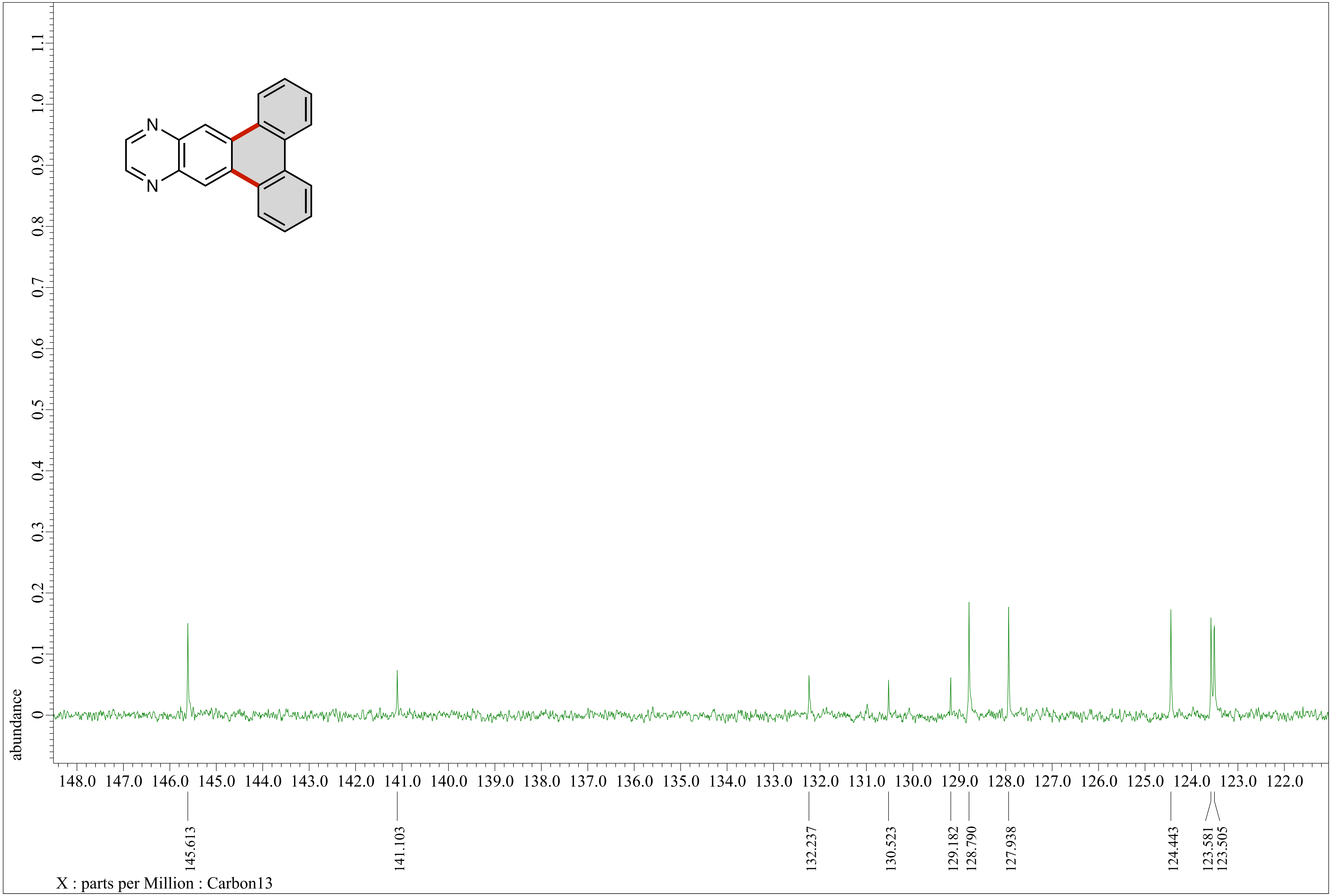

^{1}H NMR spectrum of **5gb** (solvent: $CDCl_3$)

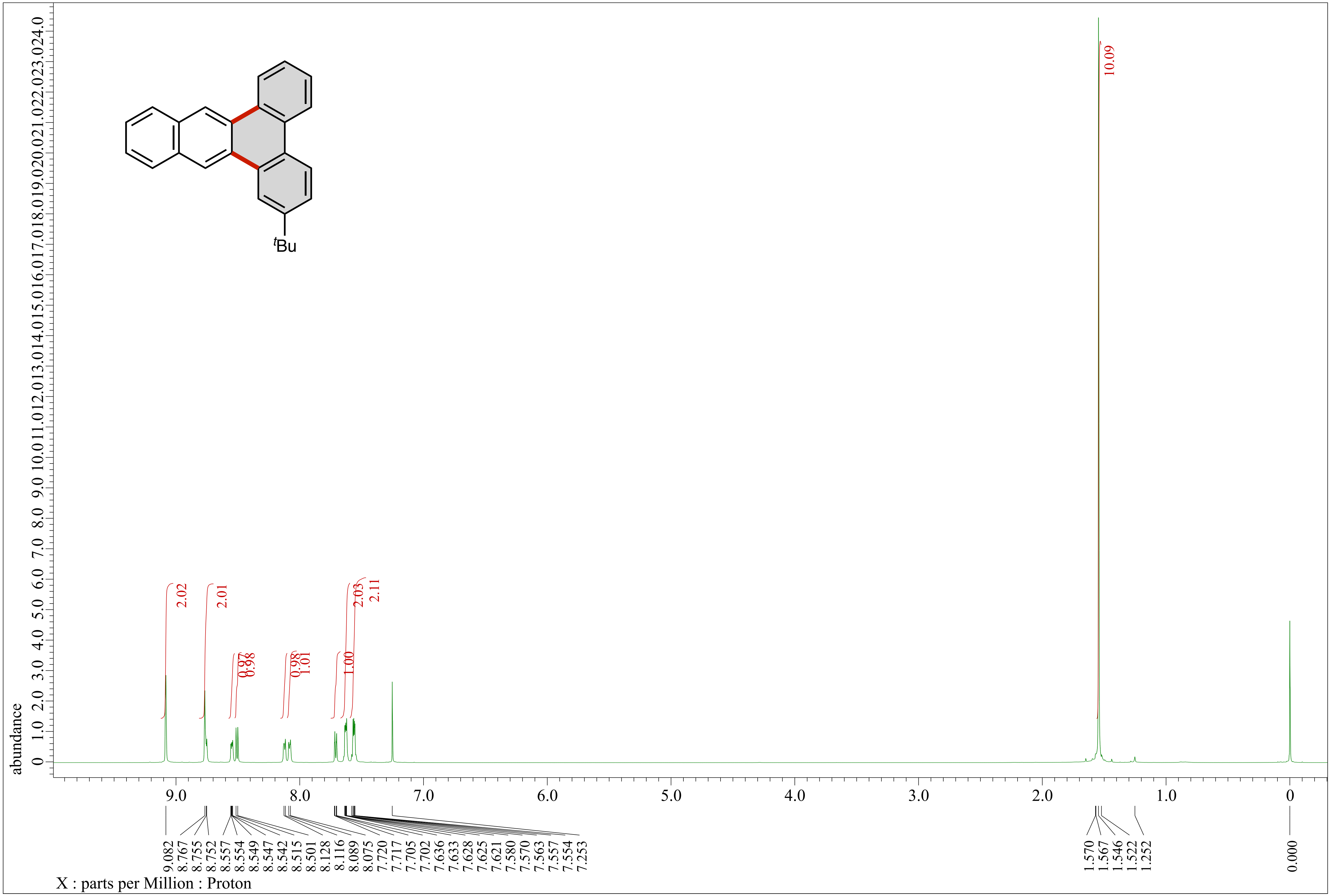

^{1}H NMR spectrum (inset) of **5gb** (solvent: $CDCl_3$)

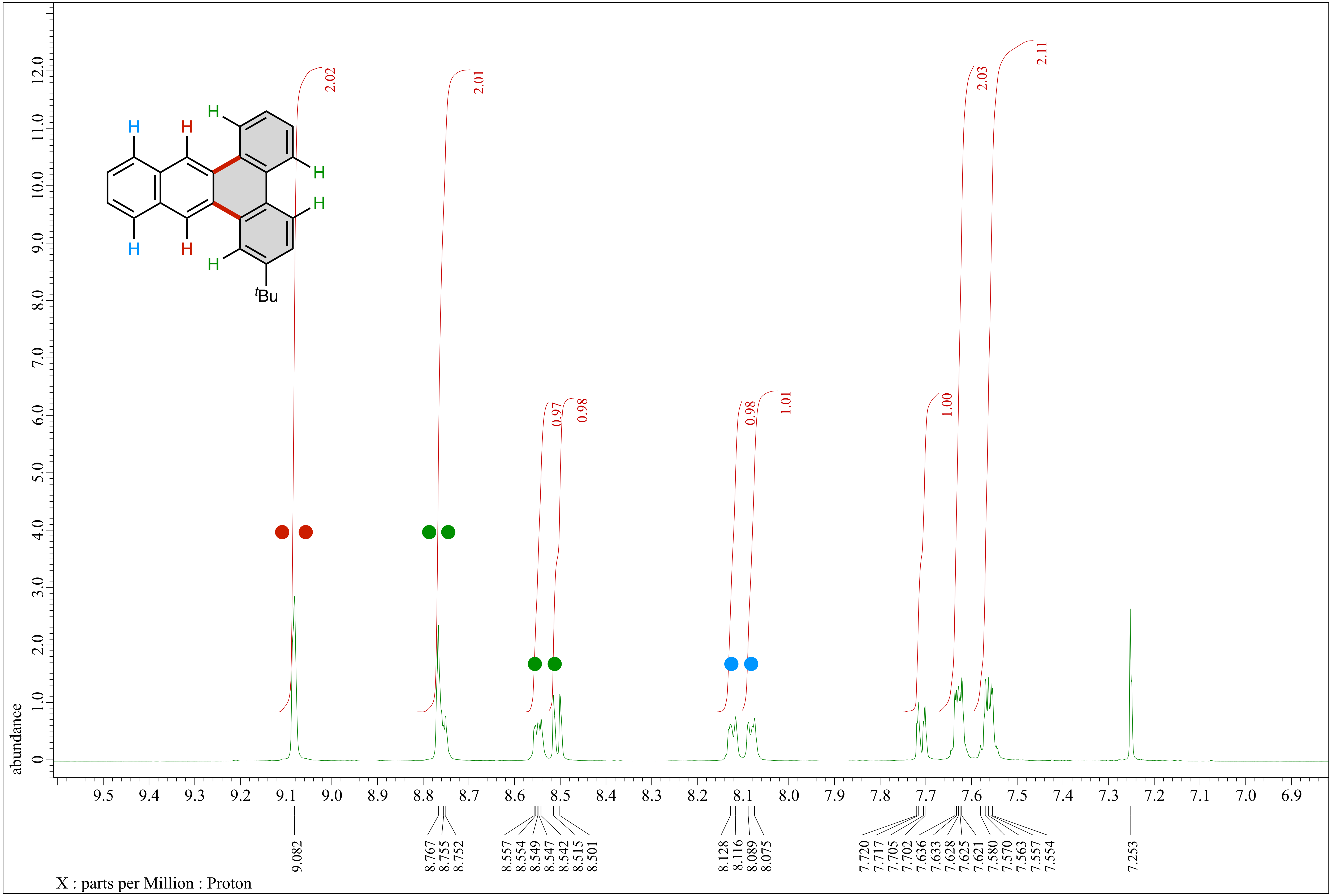

^{13}C NMR spectrum of **5gb** (solvent: $CDCl_3$)

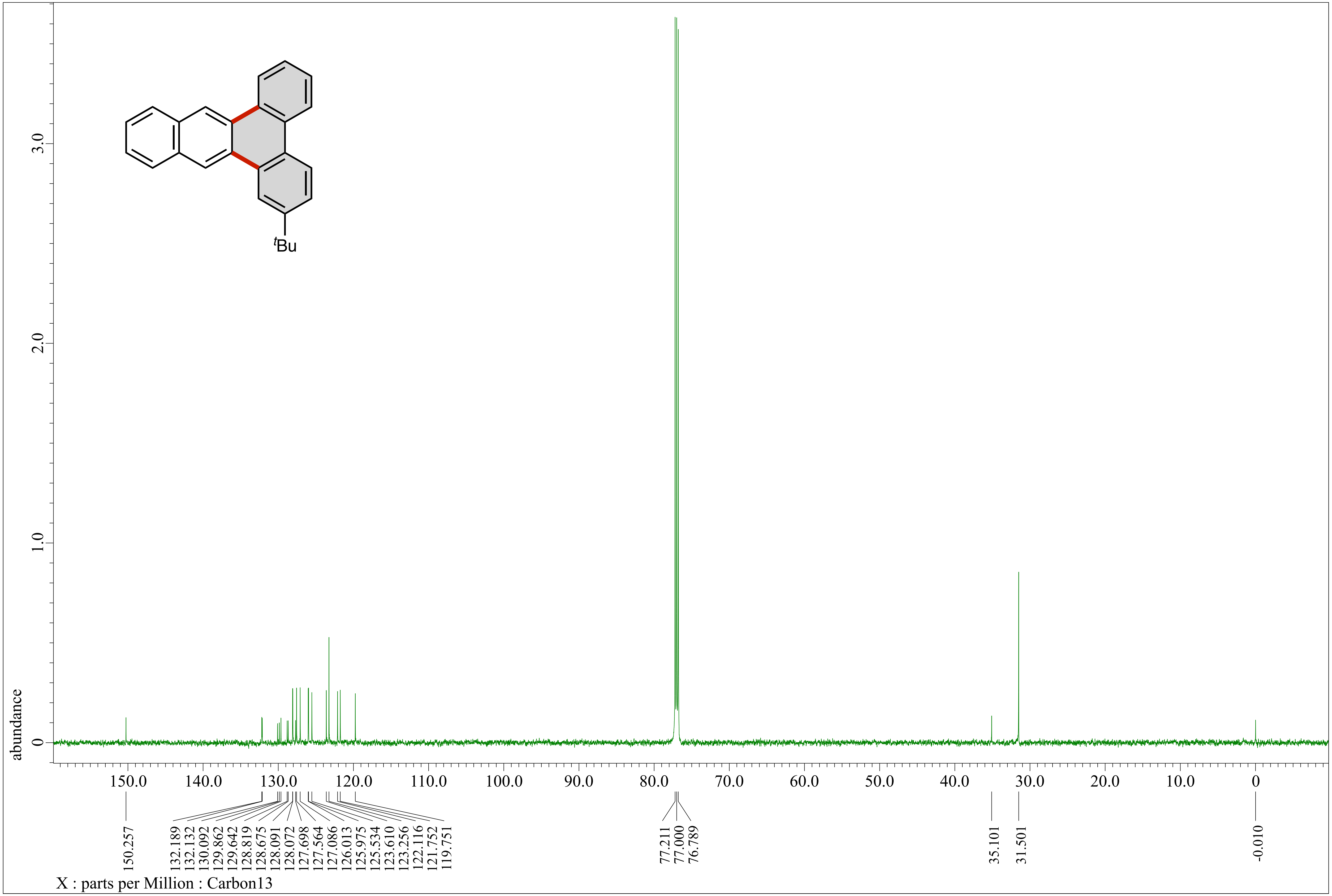

^{13}C NMR spectrum (inset) of **5gb** (solvent: $CDCl_3$)

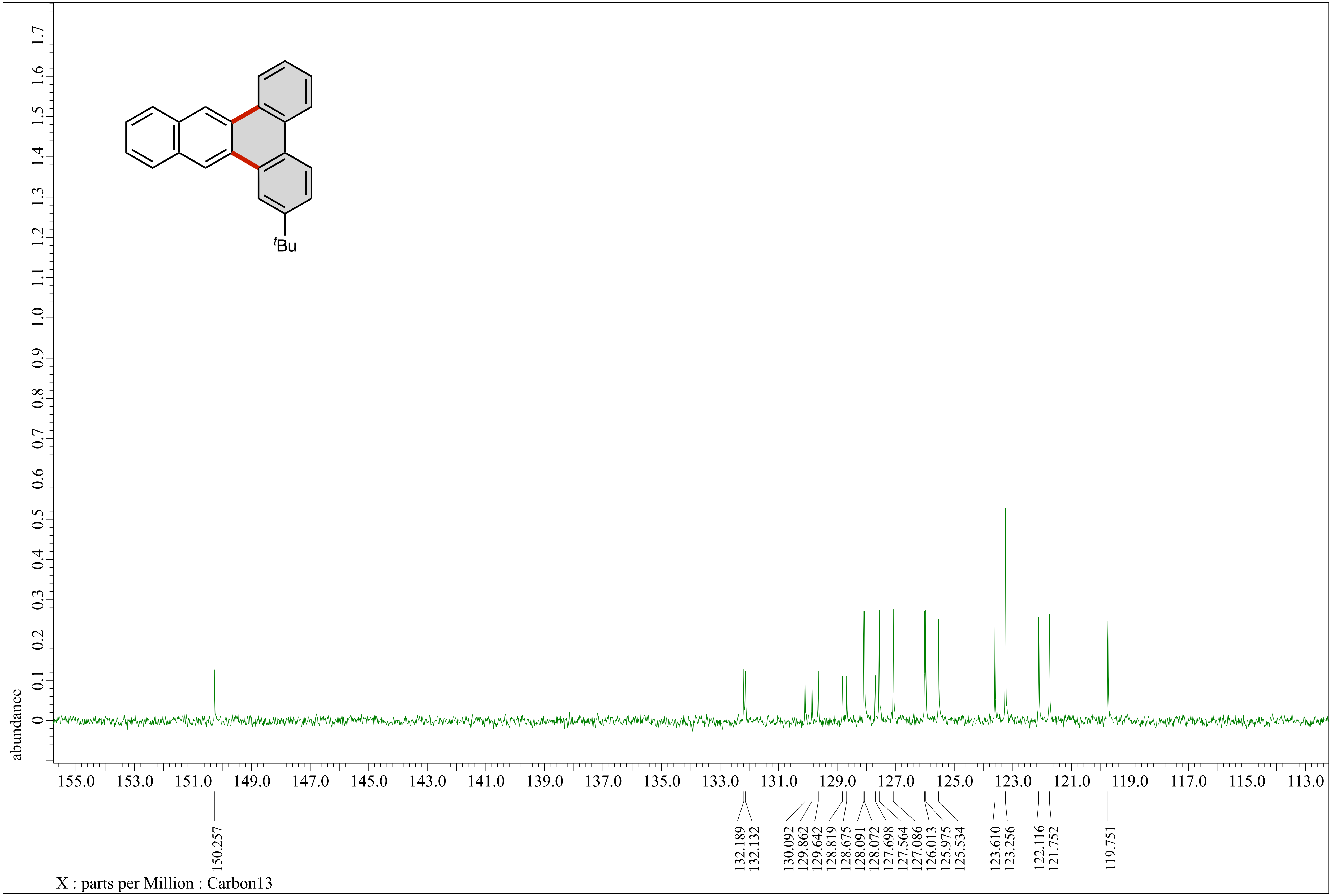

^{1}H NMR spectrum of **5gc** (solvent: $CDCl_3$)

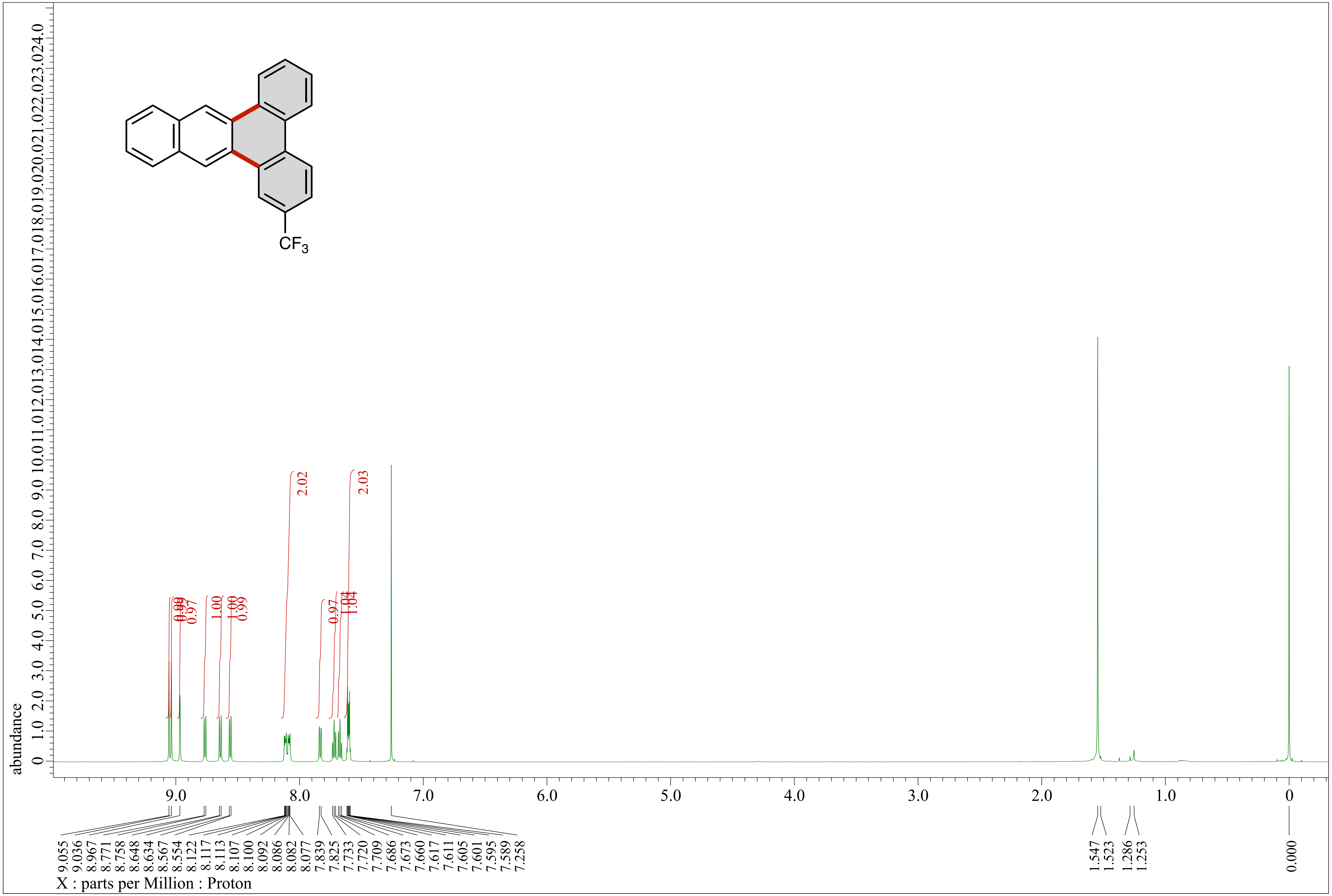

^{1}H NMR spectrum (inset) of **5gc** (solvent: $CDCl_3$)

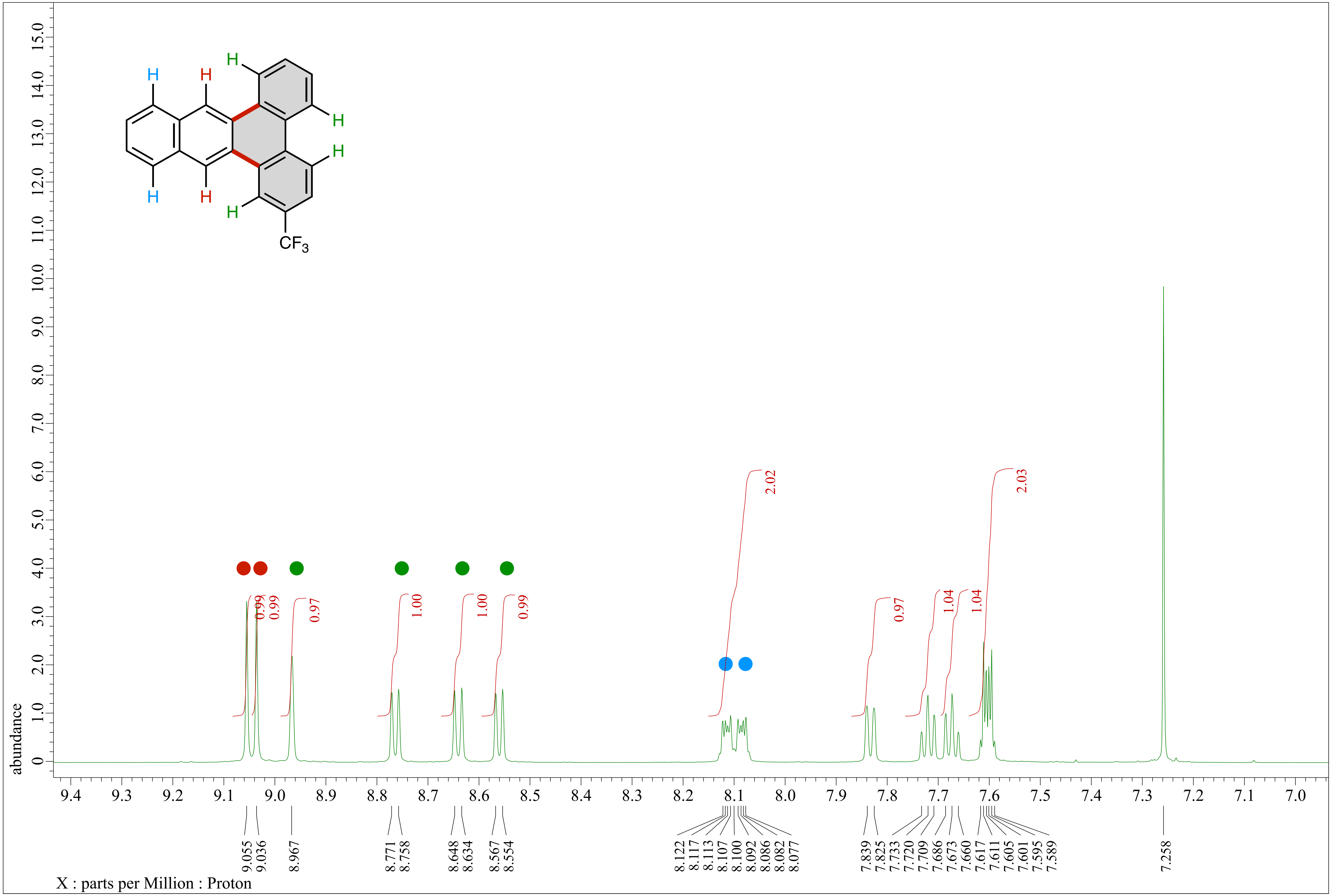

^{13}C NMR spectrum of **5gc** (solvent: $CDCl_3$)

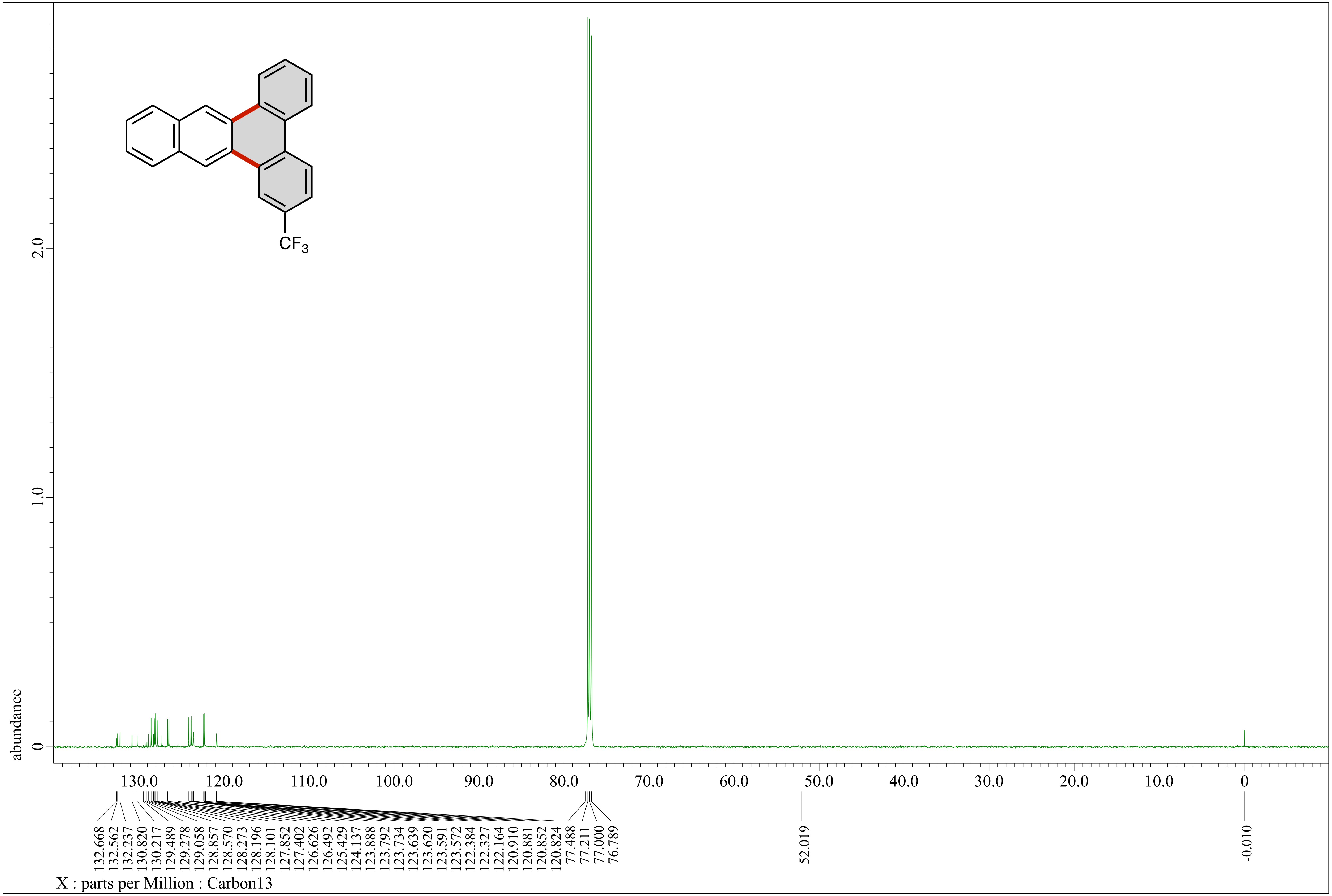

^{13}C NMR spectrum (inset) of **5gc** (solvent: $CDCl_3$)

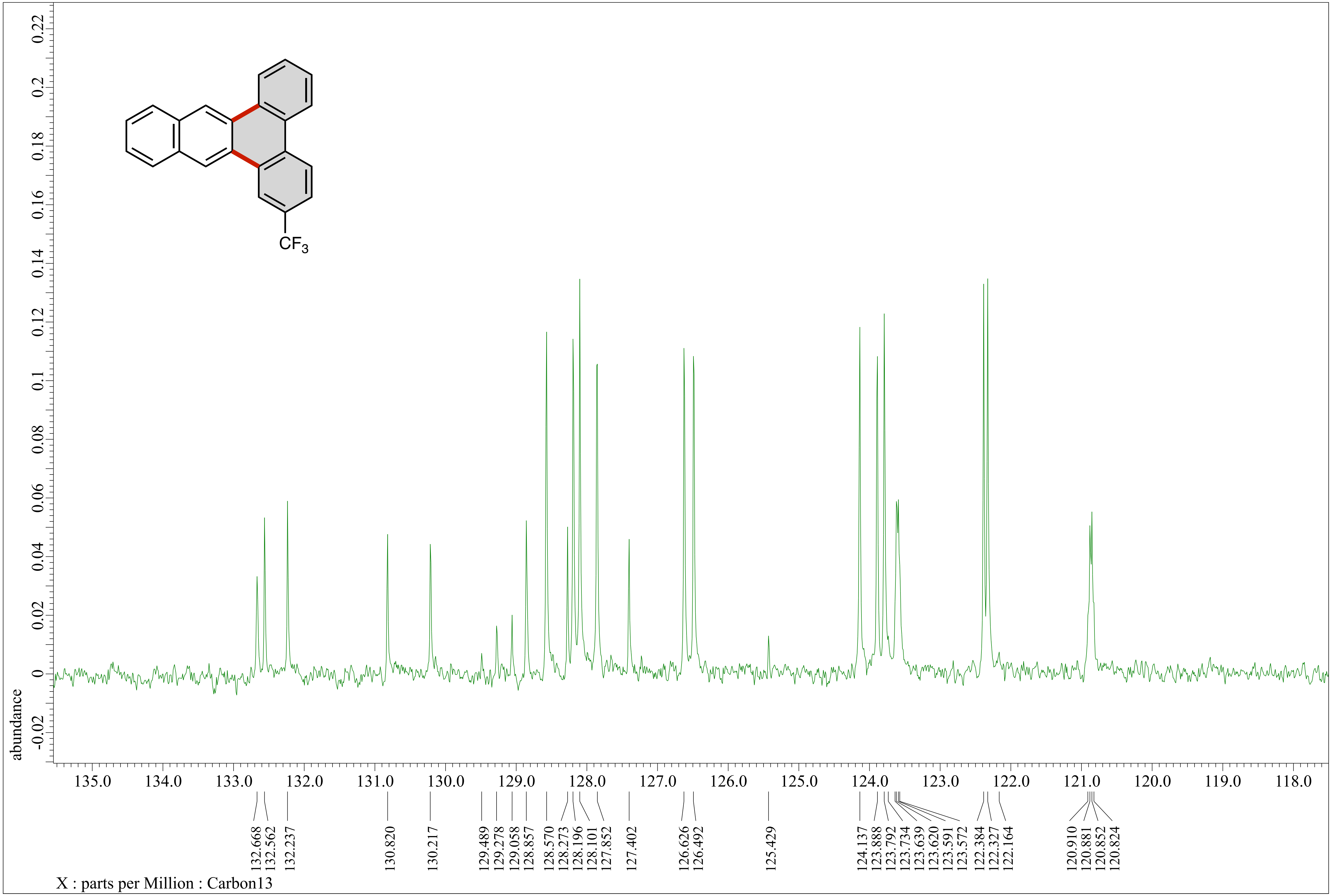

^{1}H NMR spectrum of **5gd** (solvent: $CDCl_3$)

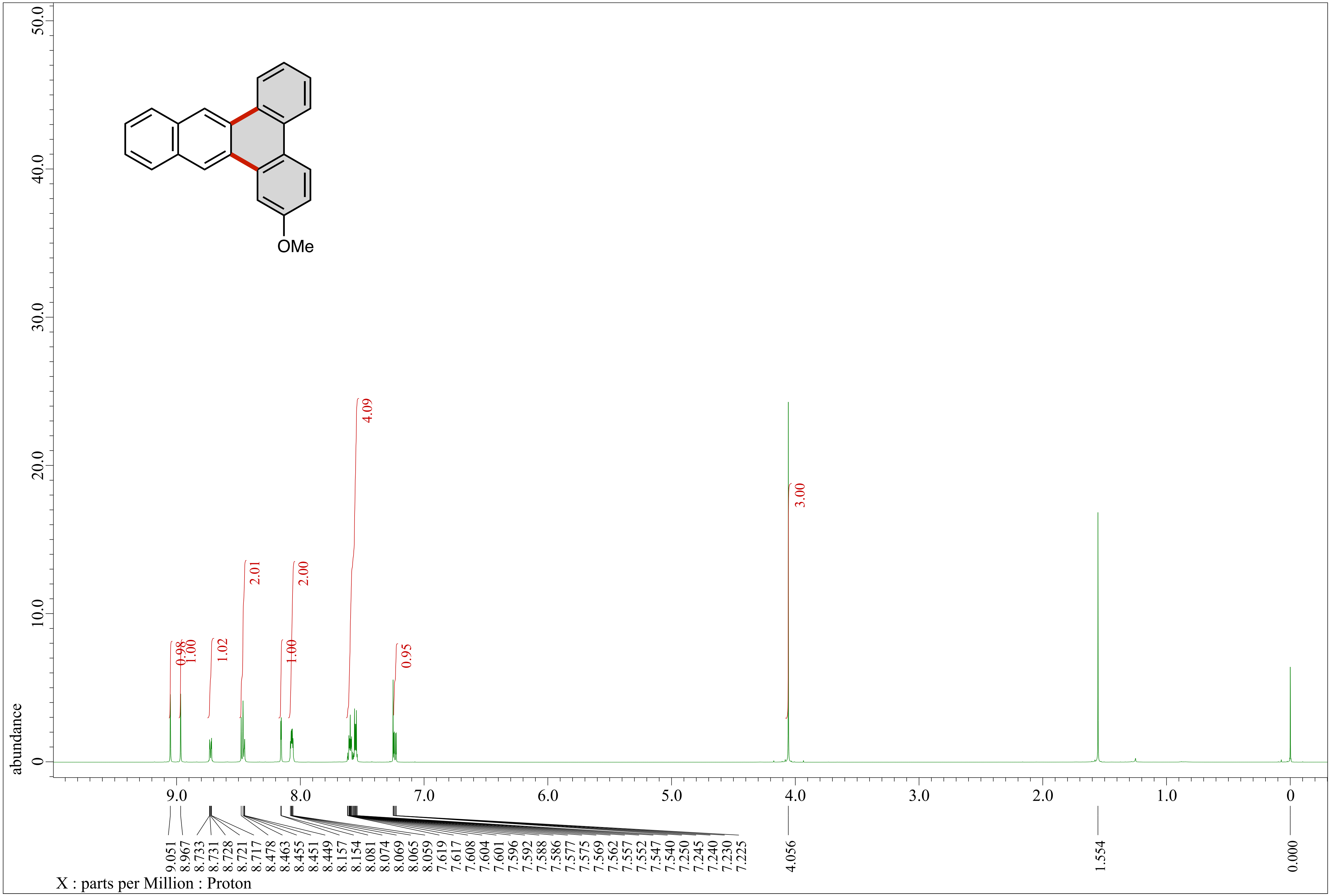

^{1}H NMR spectrum (inset) of **5gd** (solvent: $CDCl_3$)

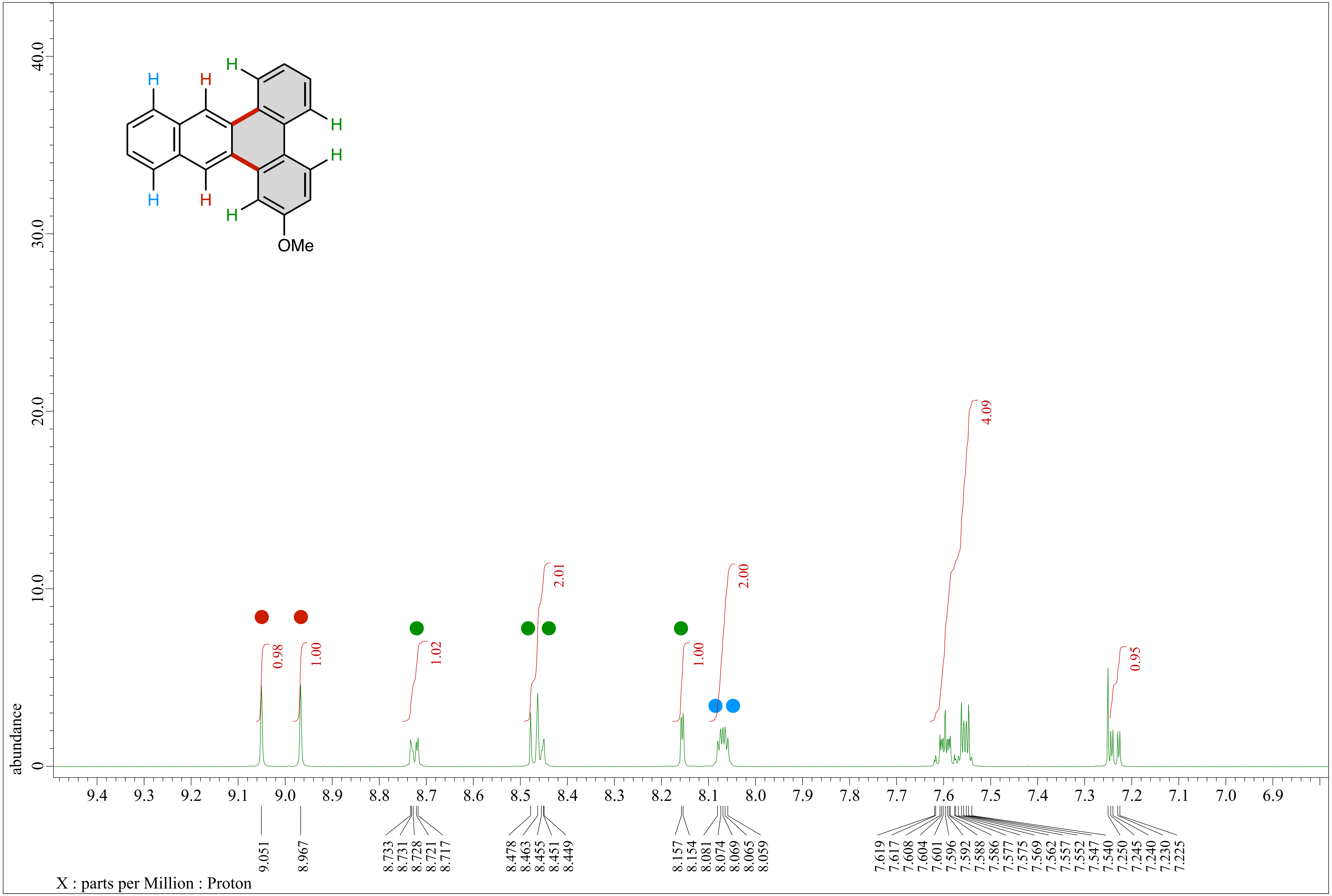

^{13}C NMR spectrum of **5gd** (solvent: $CDCl_3$)

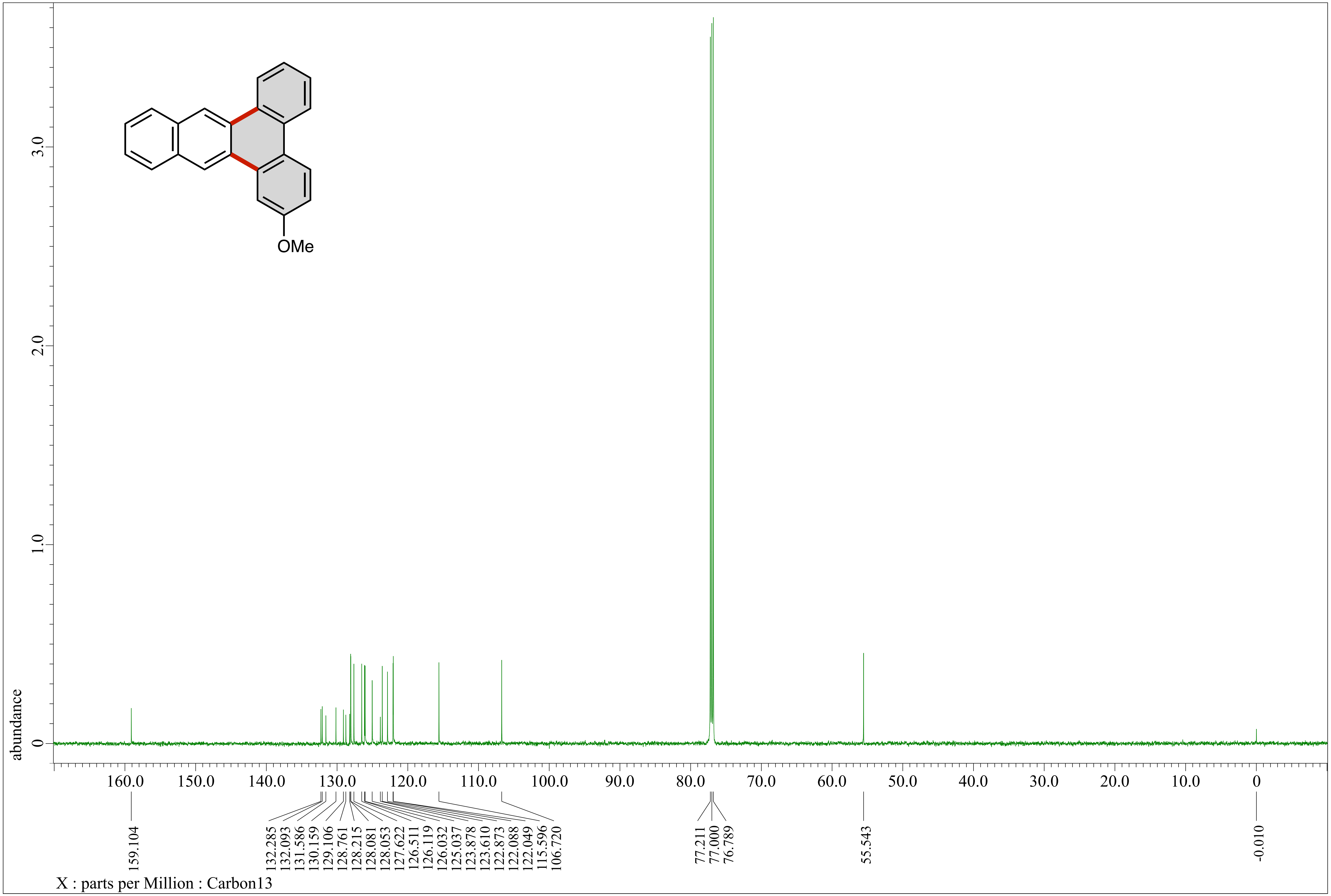

^{13}C NMR spectrum (inset) of **5gd** (solvent: $CDCl_3$)

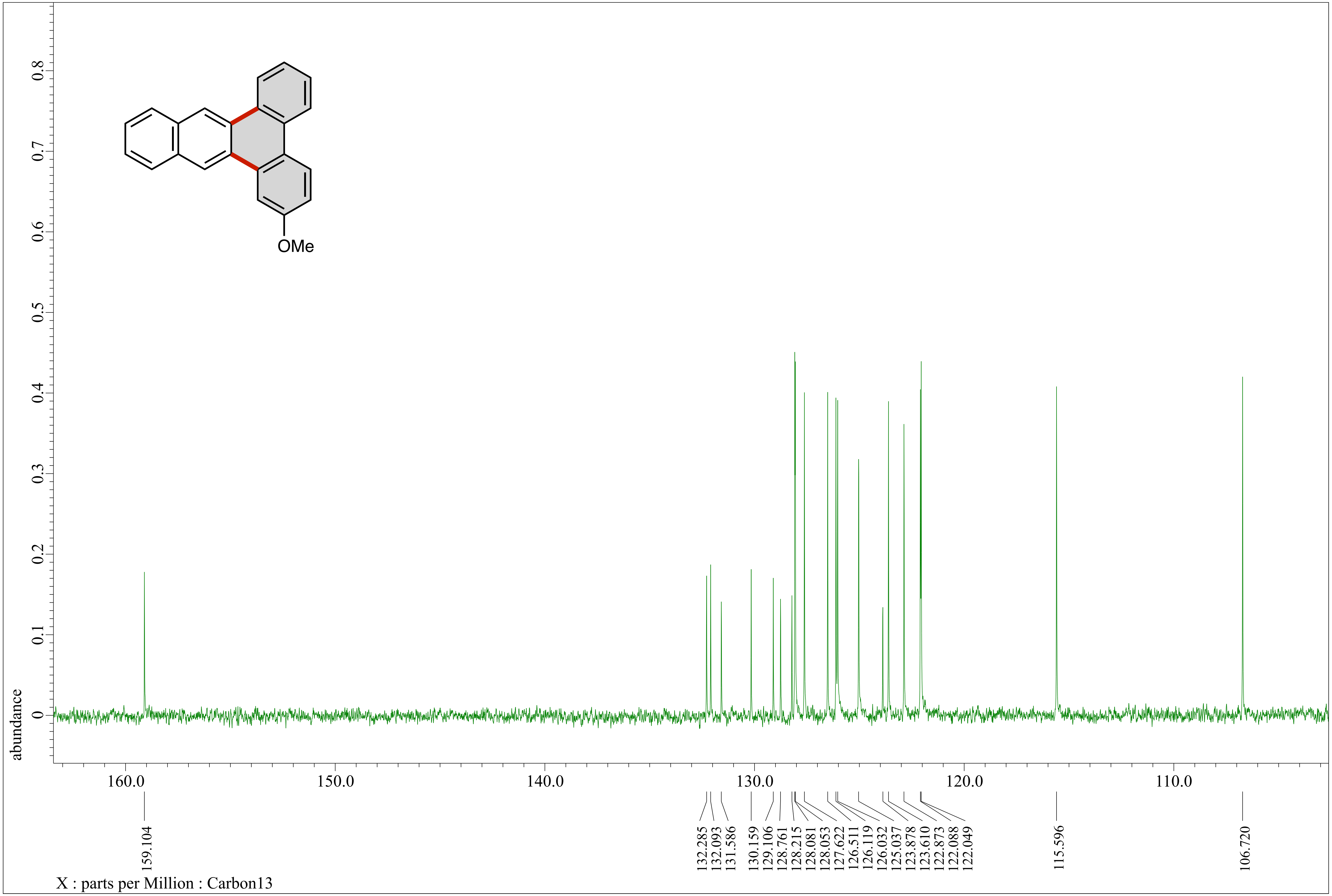

^{1}H NMR spectrum of **5ge** (solvent: $CDCl_3$)

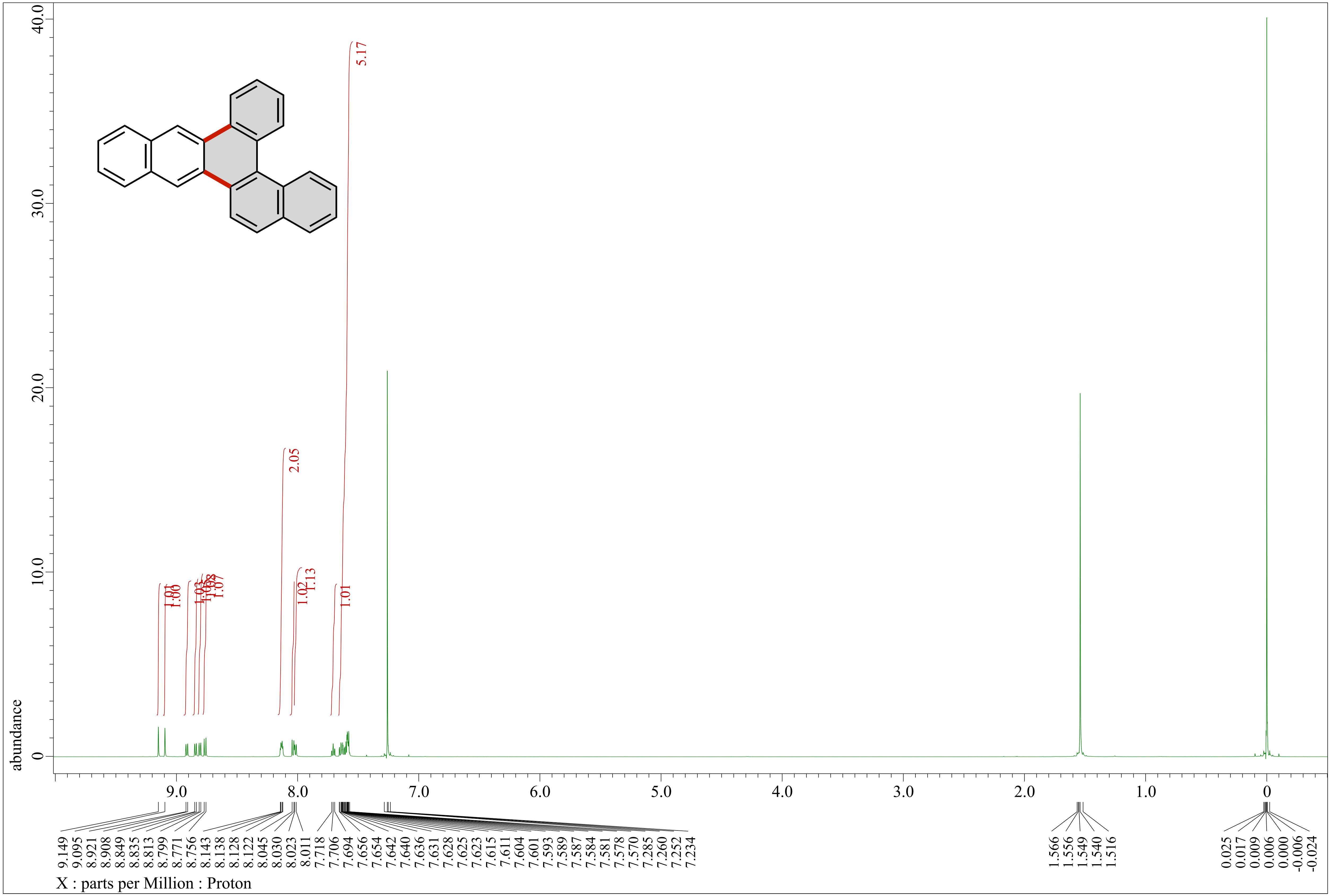

^{1}H NMR spectrum (inset) of **5ge** (solvent: $CDCl_3$)

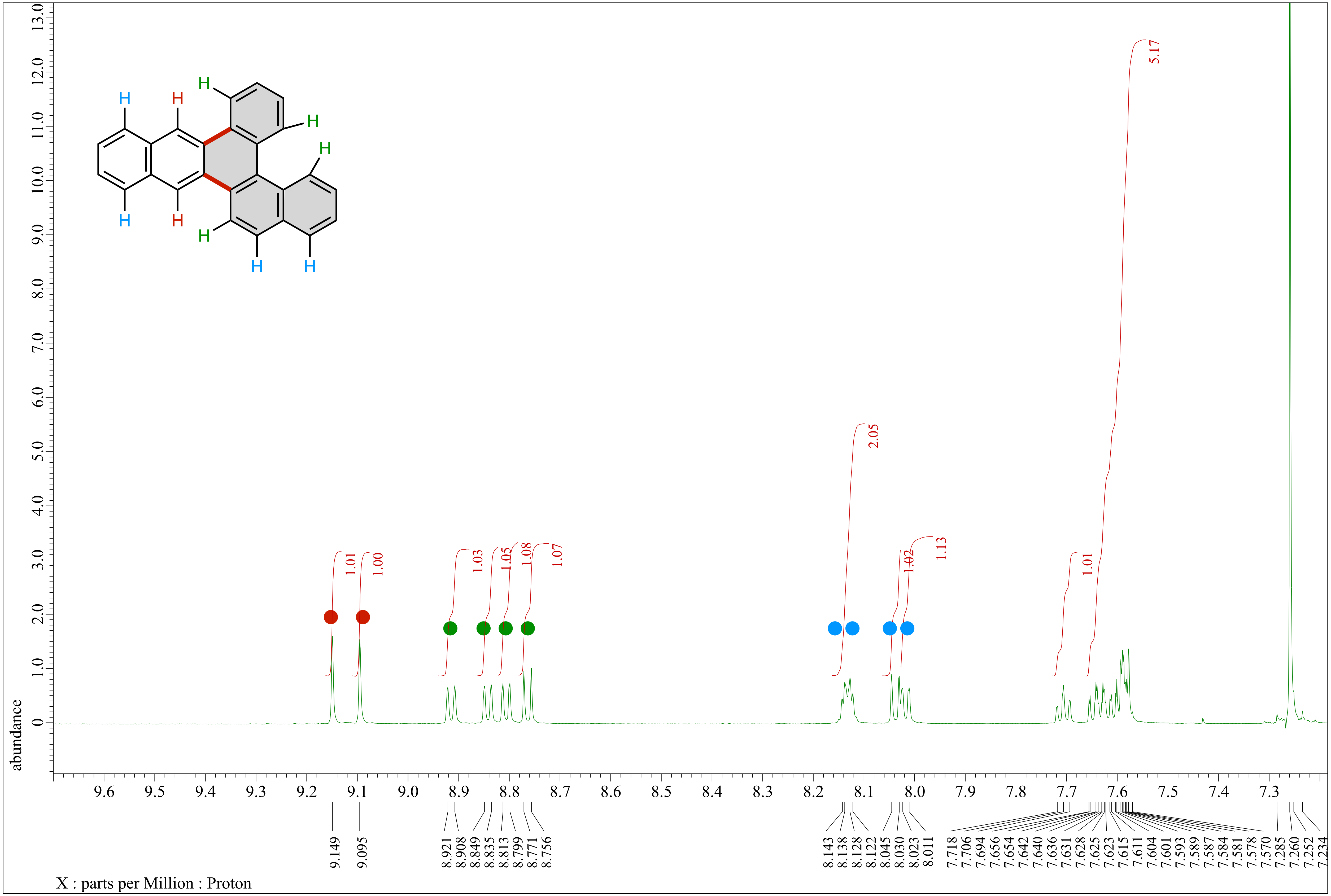

^{13}C NMR spectrum of **5ge** (solvent: $CDCl_3$)

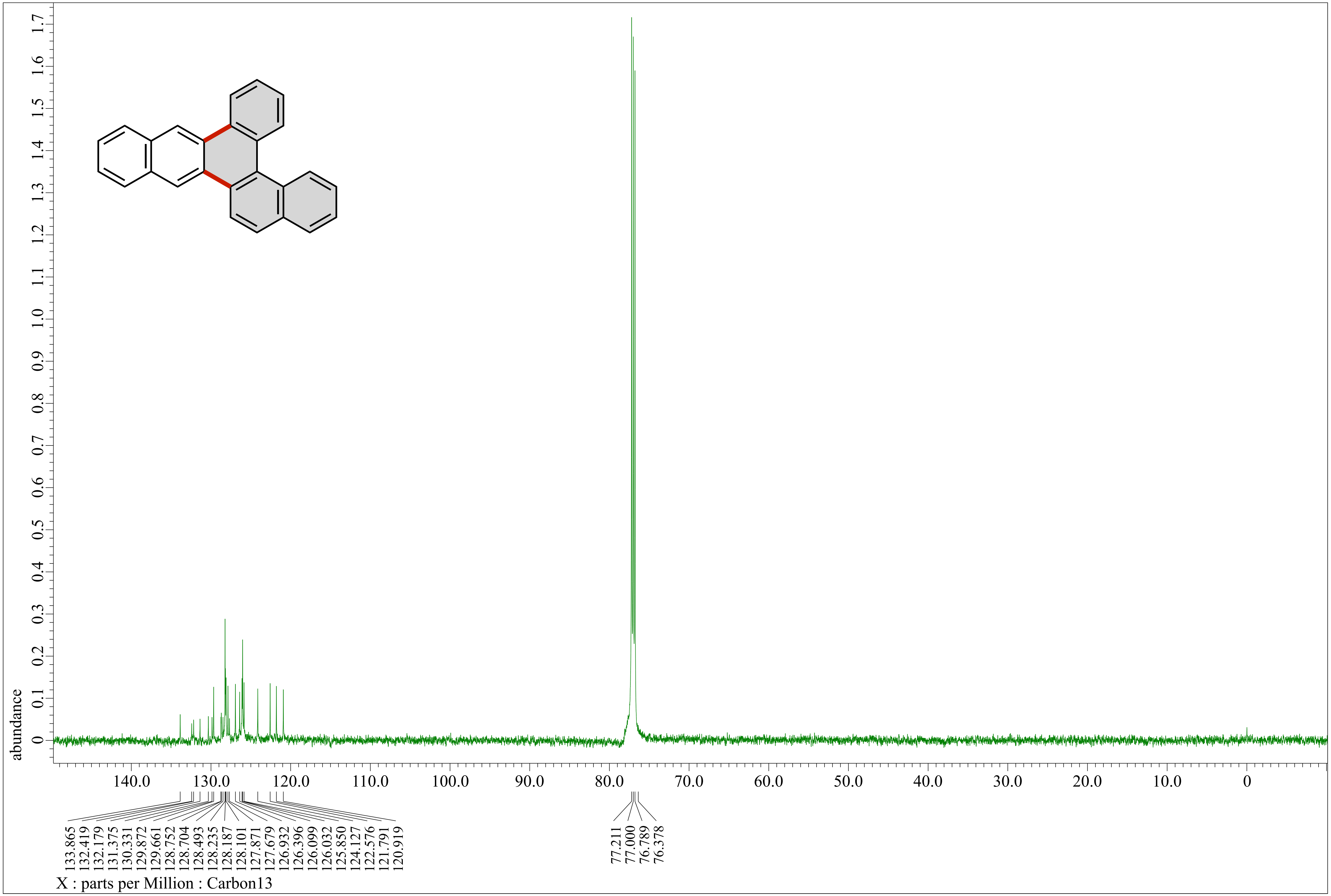

^{13}C NMR spectrum (inset) of **5ge** (solvent: $CDCl_3$)

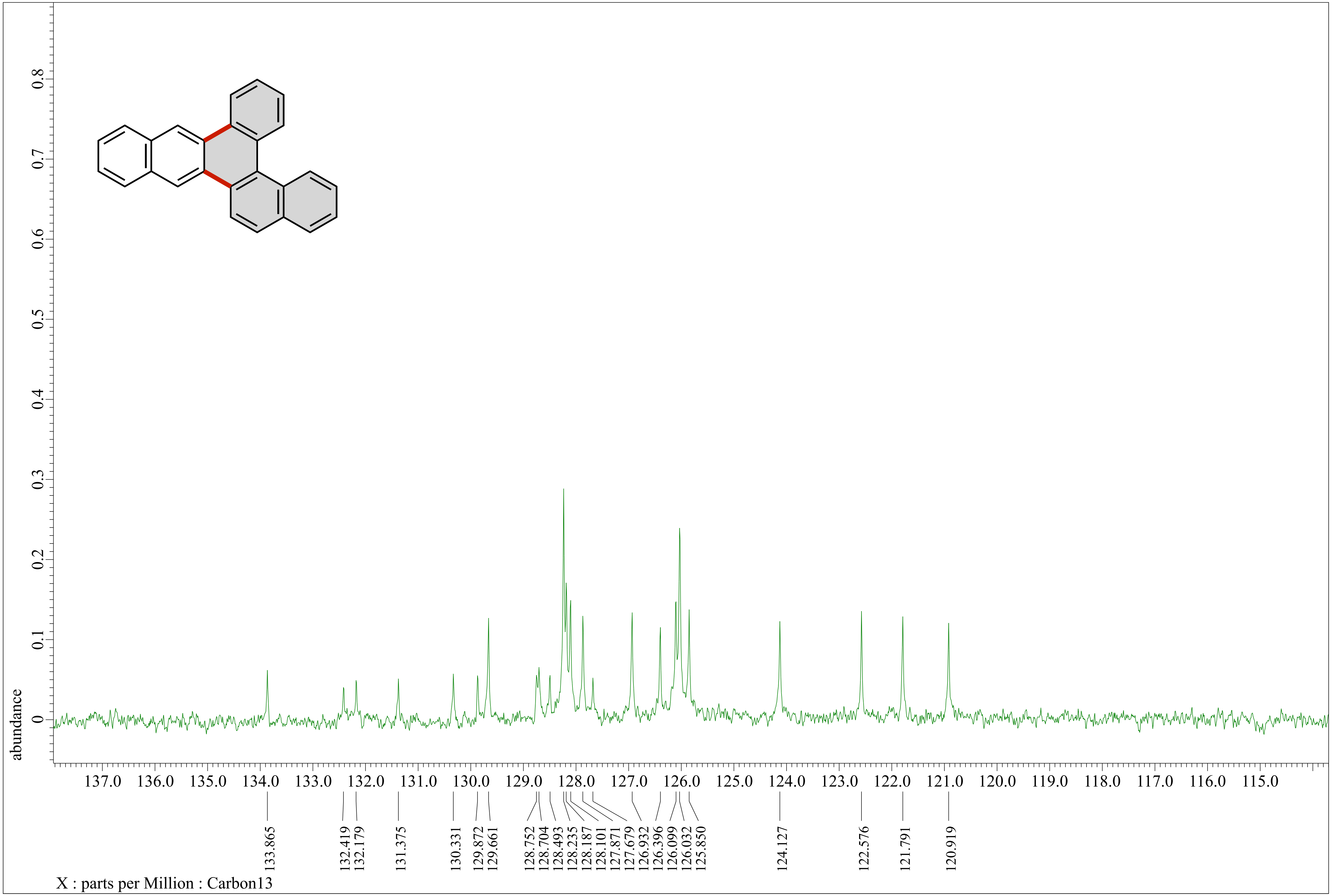

^{1}H NMR spectrum of **5gf** (solvent: $CDCl_3$)

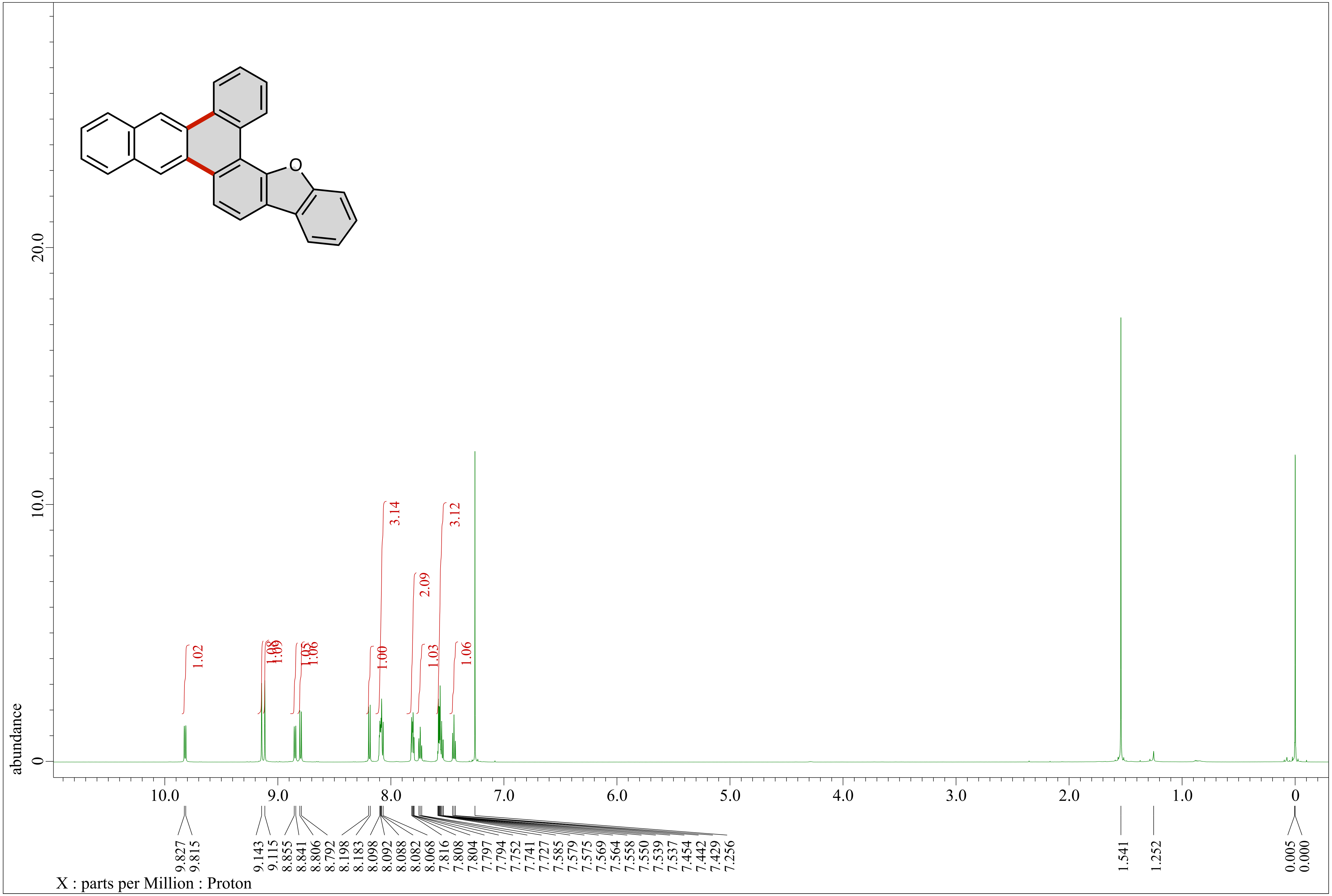

^{1}H NMR spectrum (inset) of **5gf** (solvent: $CDCl_3$)

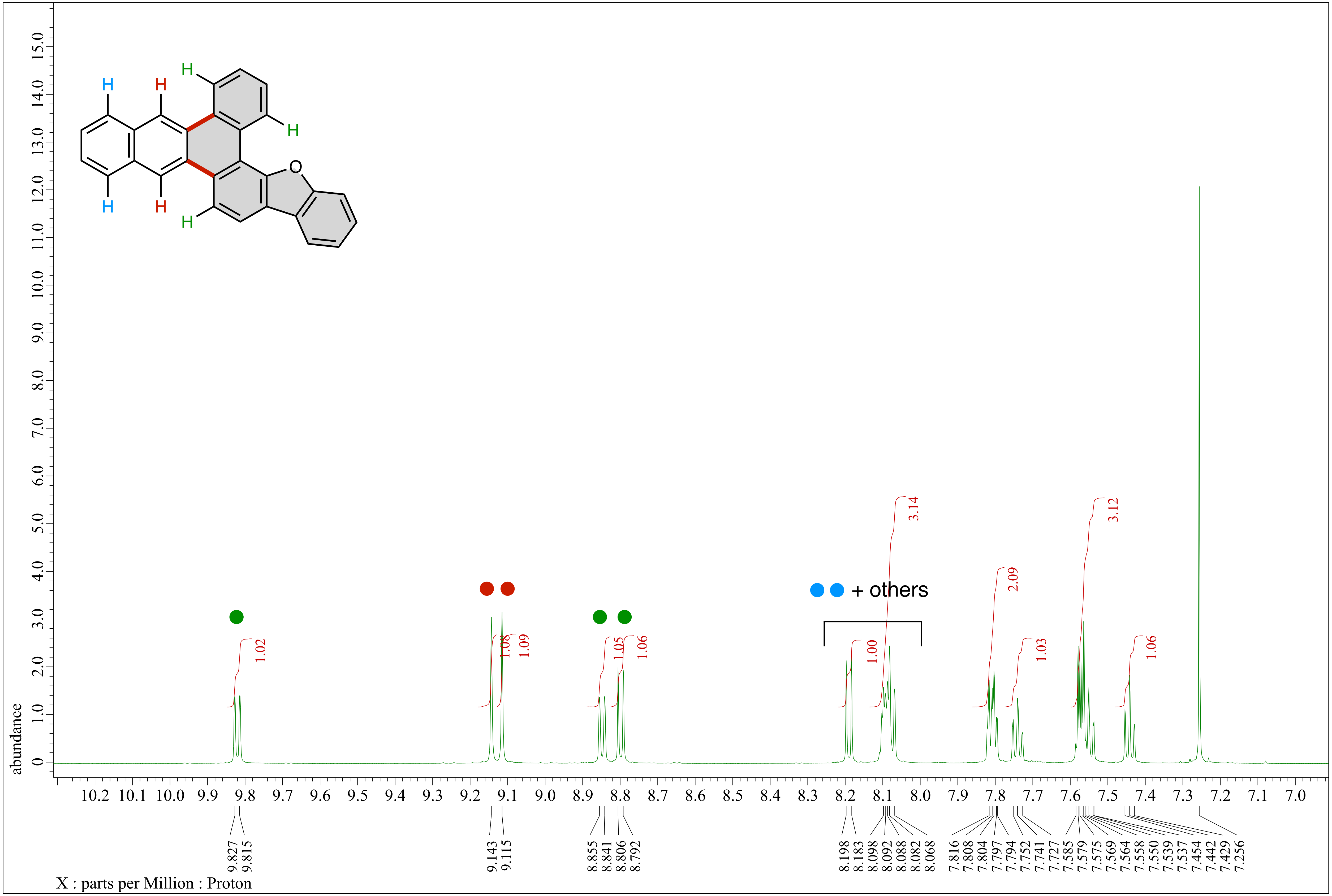

^{13}C NMR spectrum of **5gf** (solvent: $CDCl_3$)

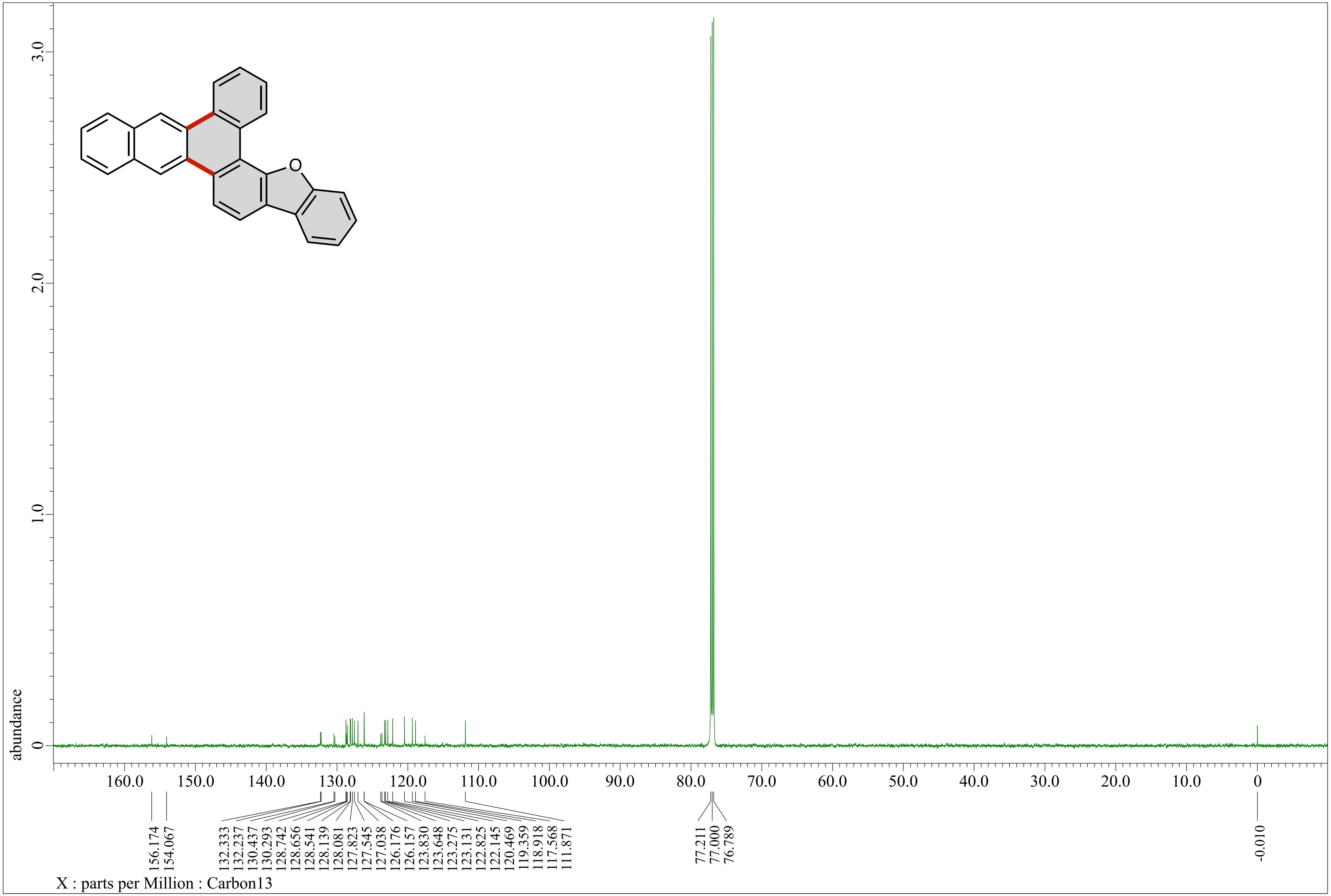

^{13}C NMR spectrum (inset) of **5gf** (solvent: $CDCl_3$)

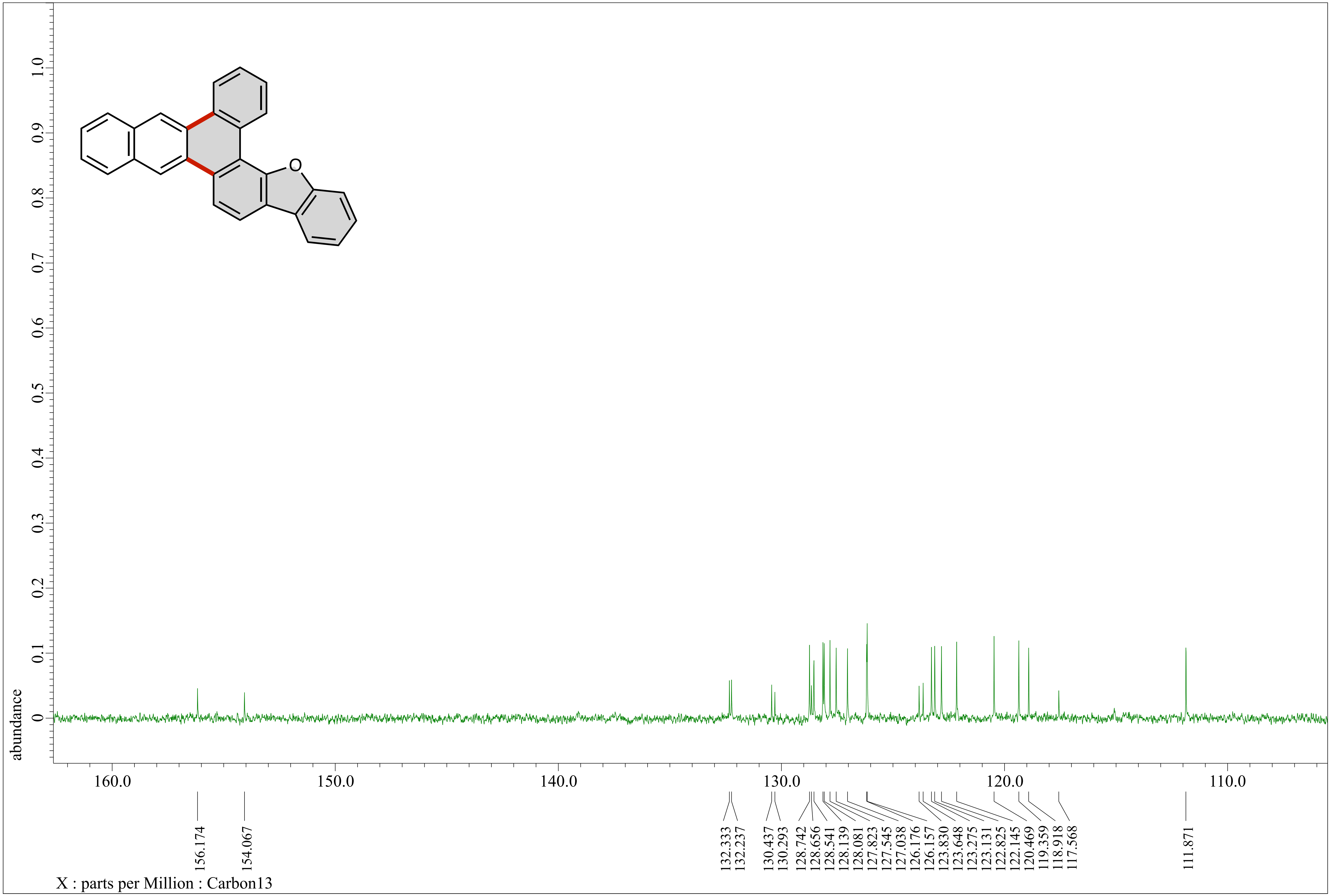

^{1}H NMR spectrum of **5gg** (solvent: $CDCl_3$)

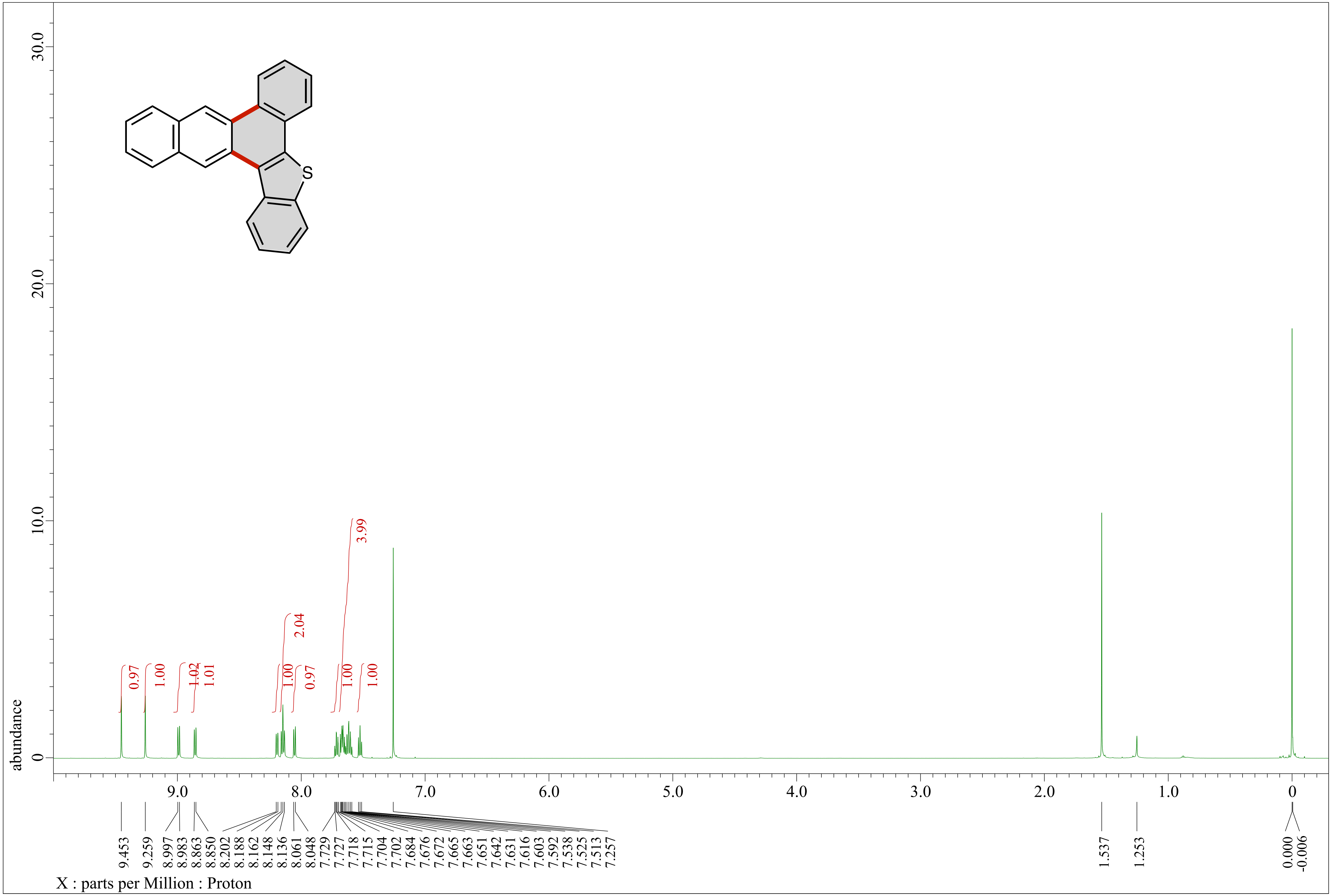

^{1}H NMR spectrum (inset) of **5gg** (solvent: $CDCl_3$)

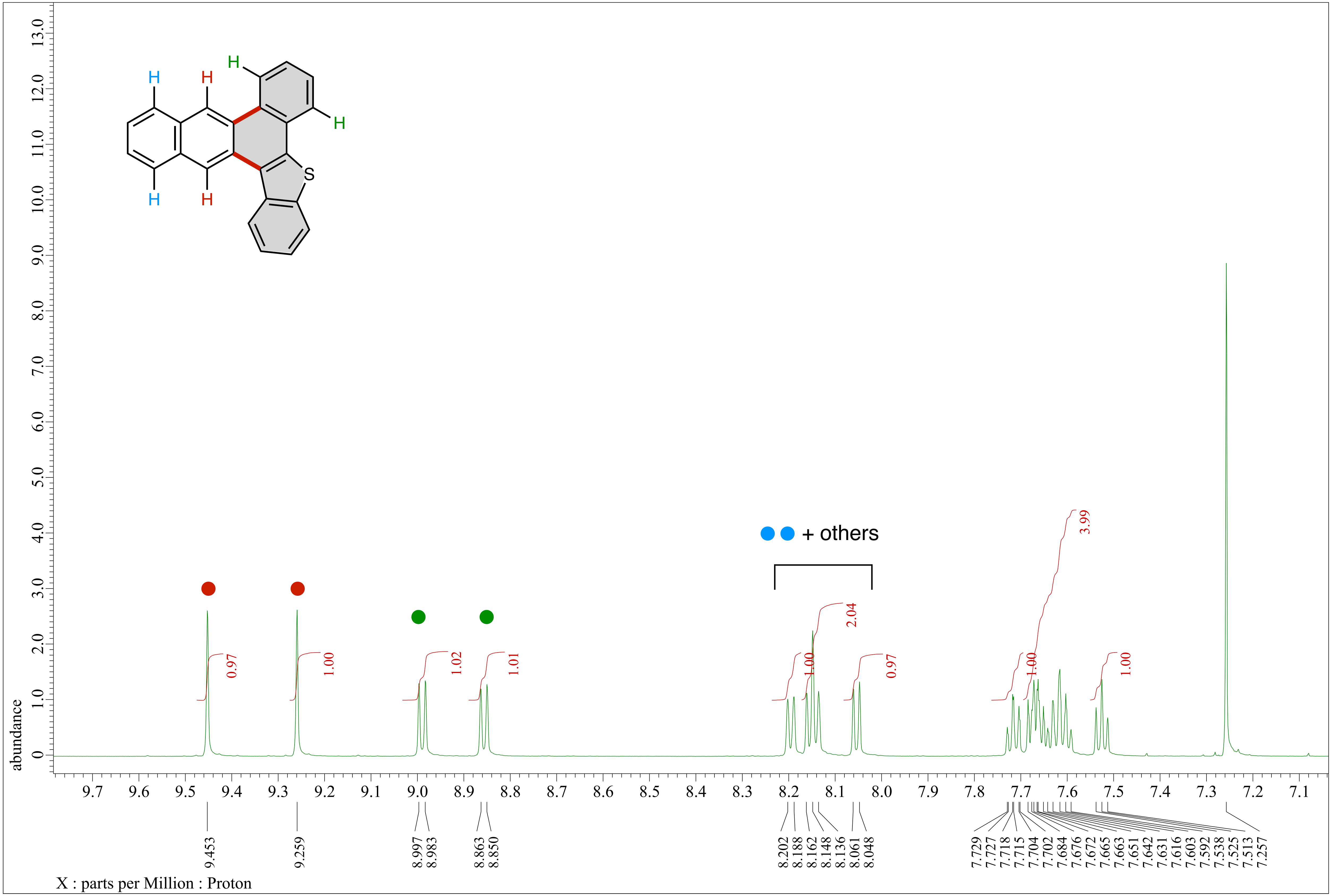

^{13}C NMR spectrum of **5gg** (solvent: $CDCl_3$)

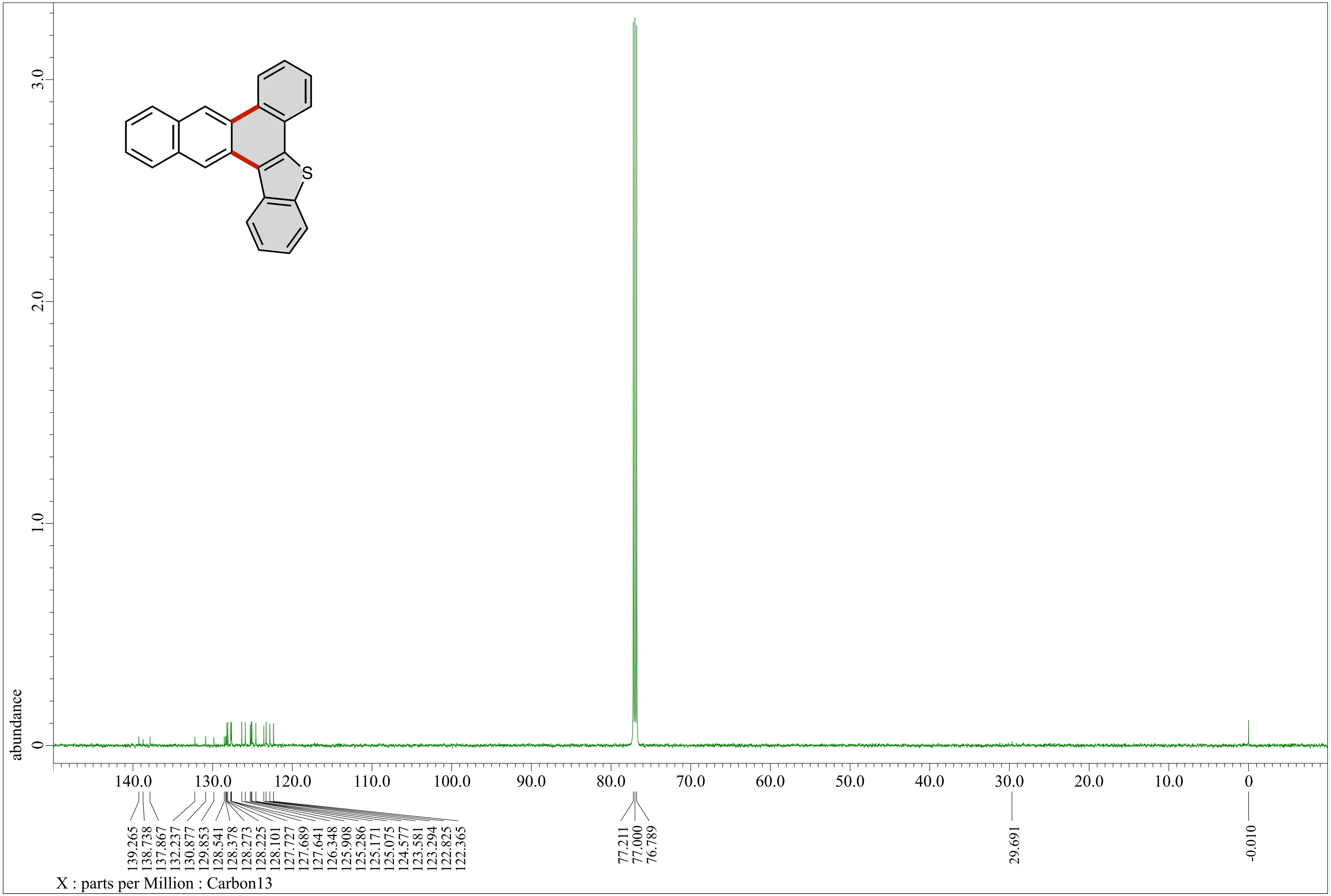

^{13}C NMR spectrum (inset) of **5gg** (solvent: $CDCl_3$)

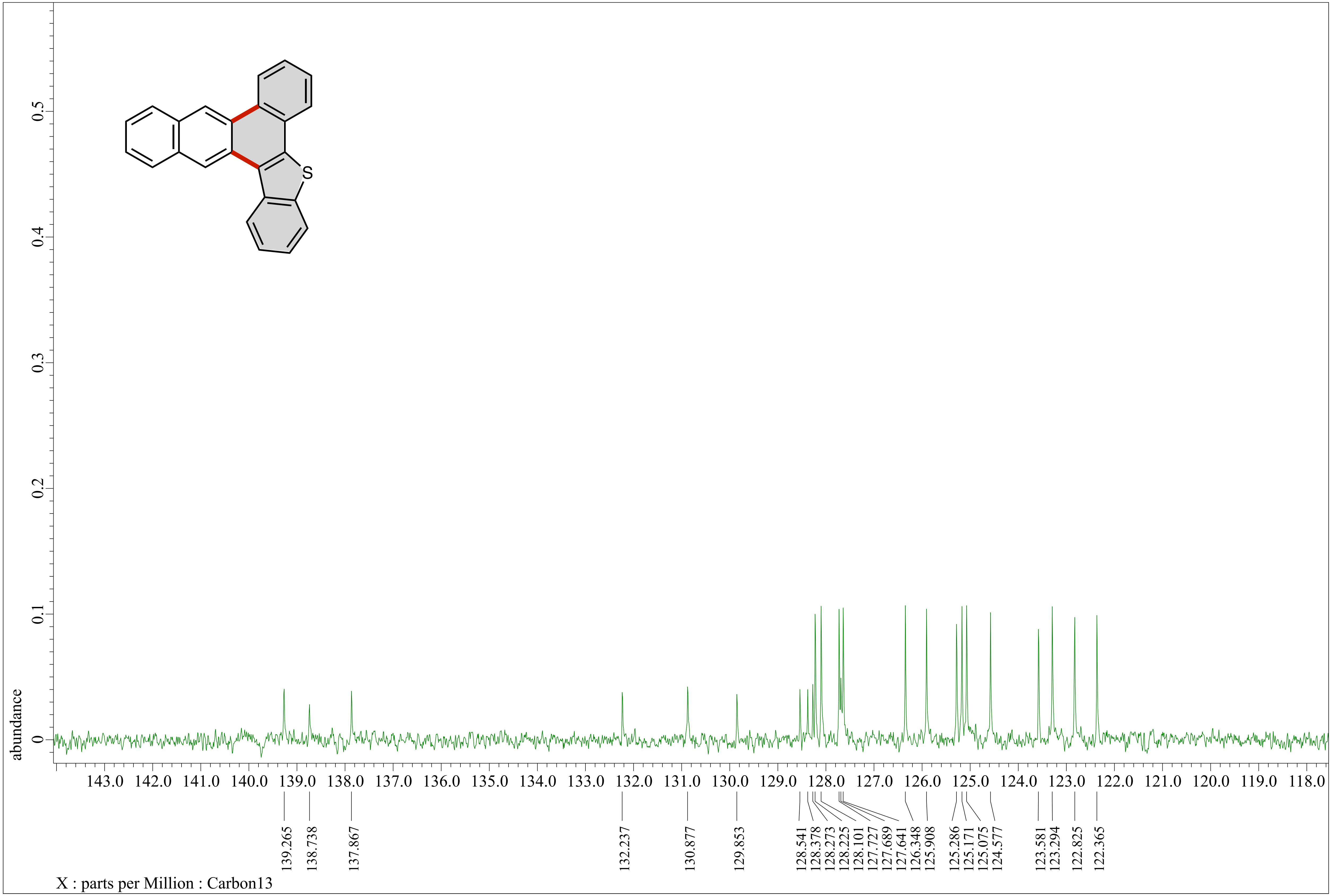

^{1}H NMR spectrum of **6ba-1** (solvent: $CDCl_3$)

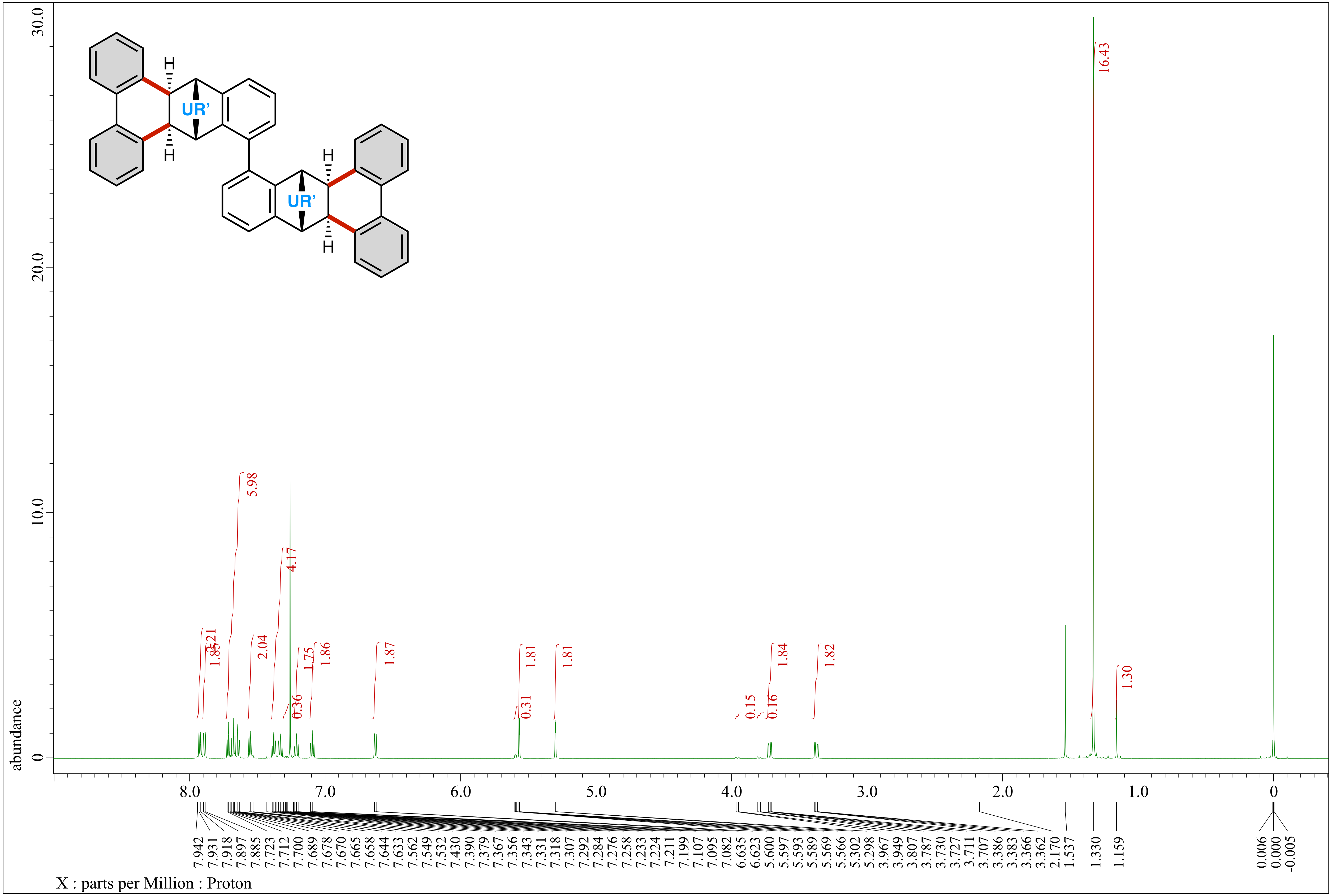

^{1}H NMR spectrum (inset) of **6ba-1** (solvent: $CDCl_3$)

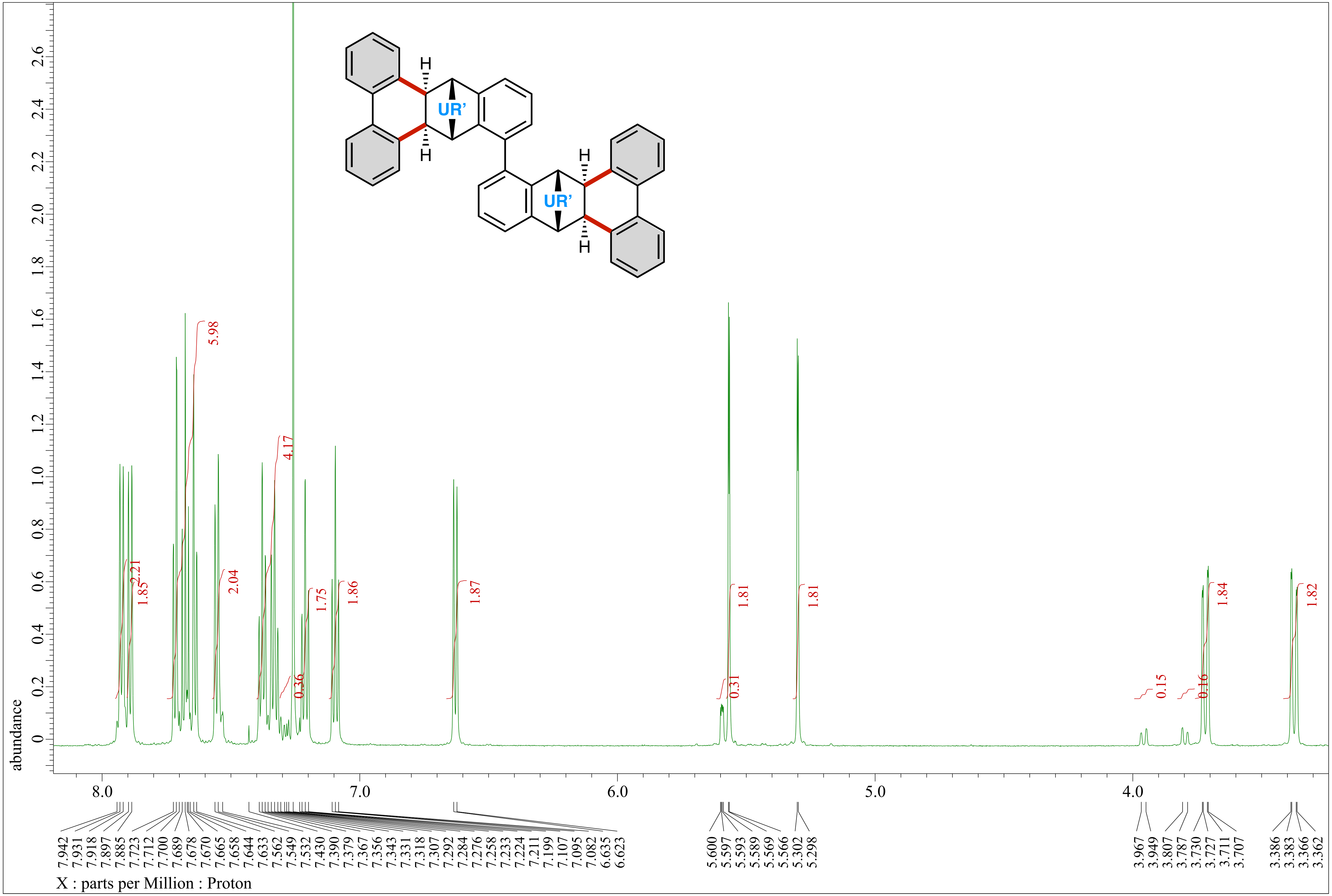

^{13}C NMR spectrum of **6ba-1** (solvent: $CDCl_3$)

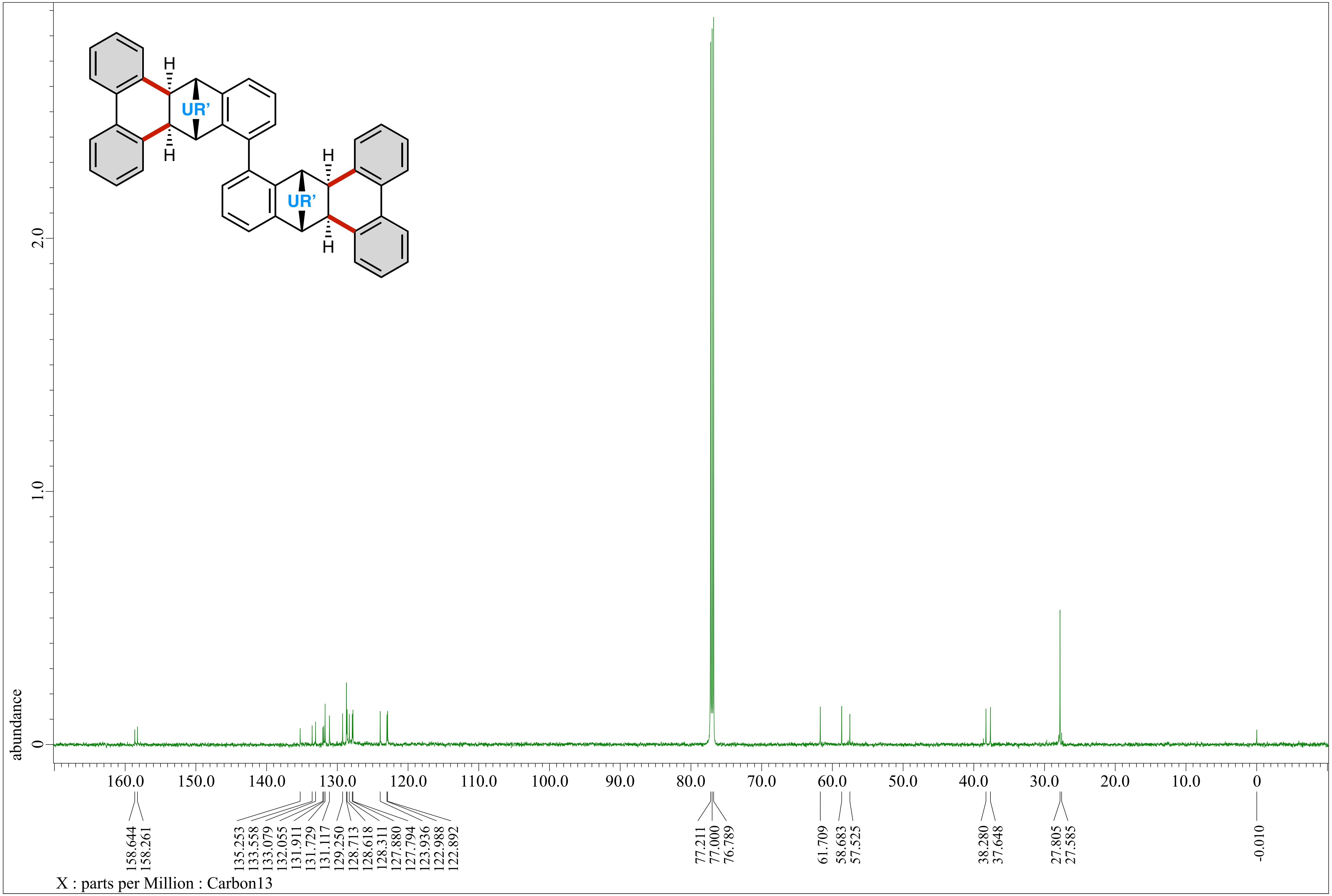

^{13}C NMR spectrum (inset) of **6ba-1** (solvent: $CDCl_3$)

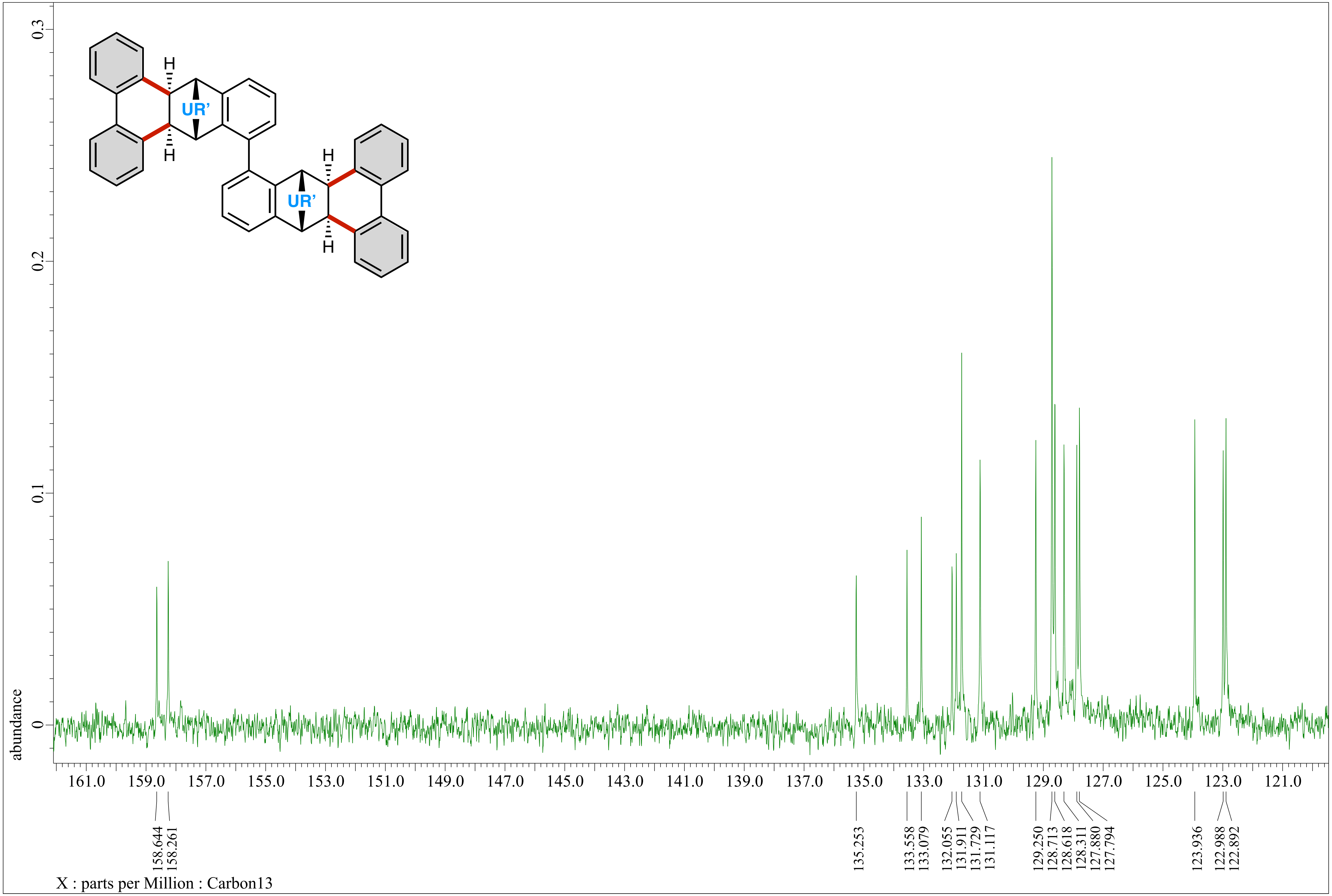

^{1}H NMR spectrum of **6ba-2** (solvent: $Cl_2CDCDCl_2$)

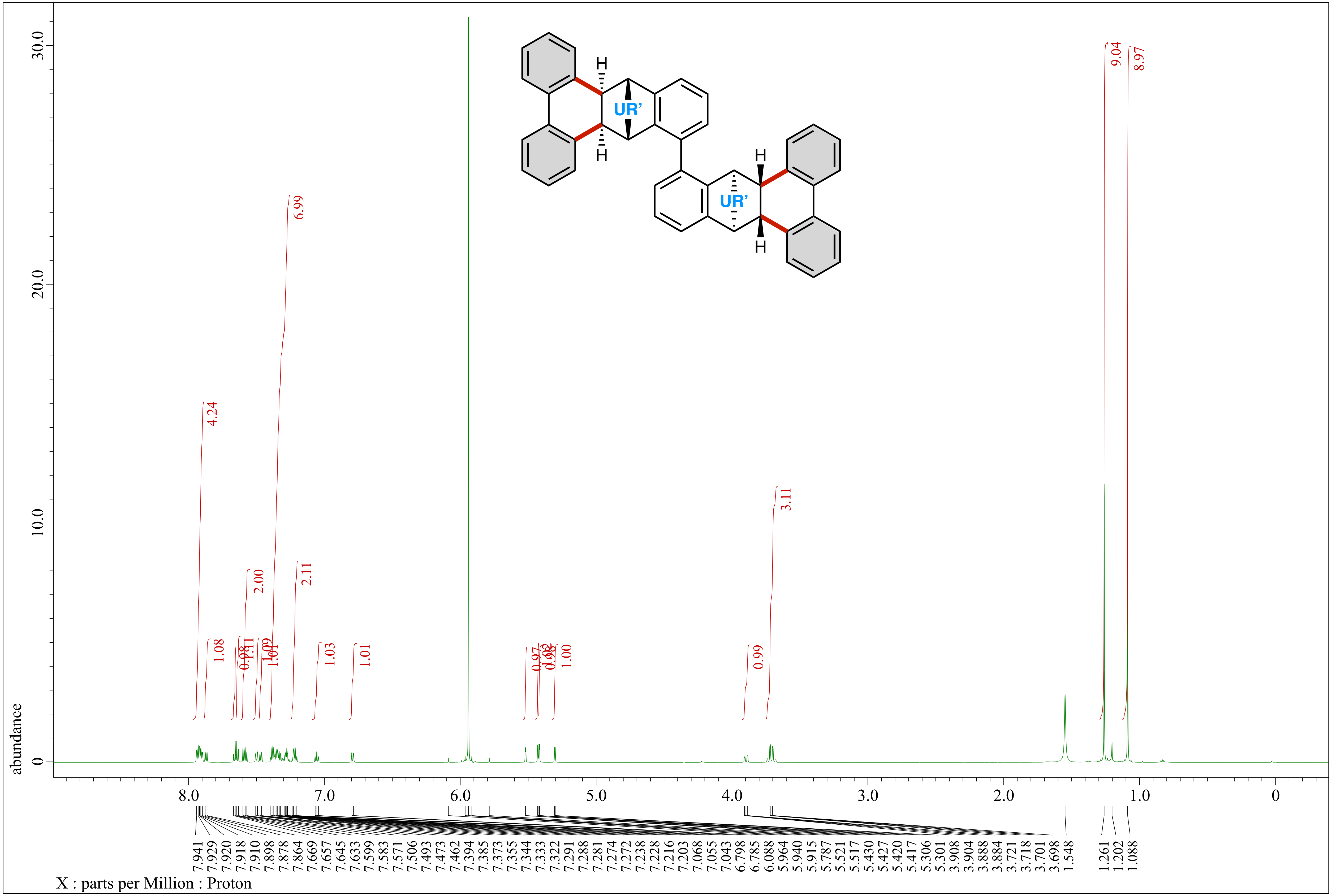

^{1}H NMR spectrum (inset) of **6ba-2** (solvent: $Cl_2CDCDCl_2$)

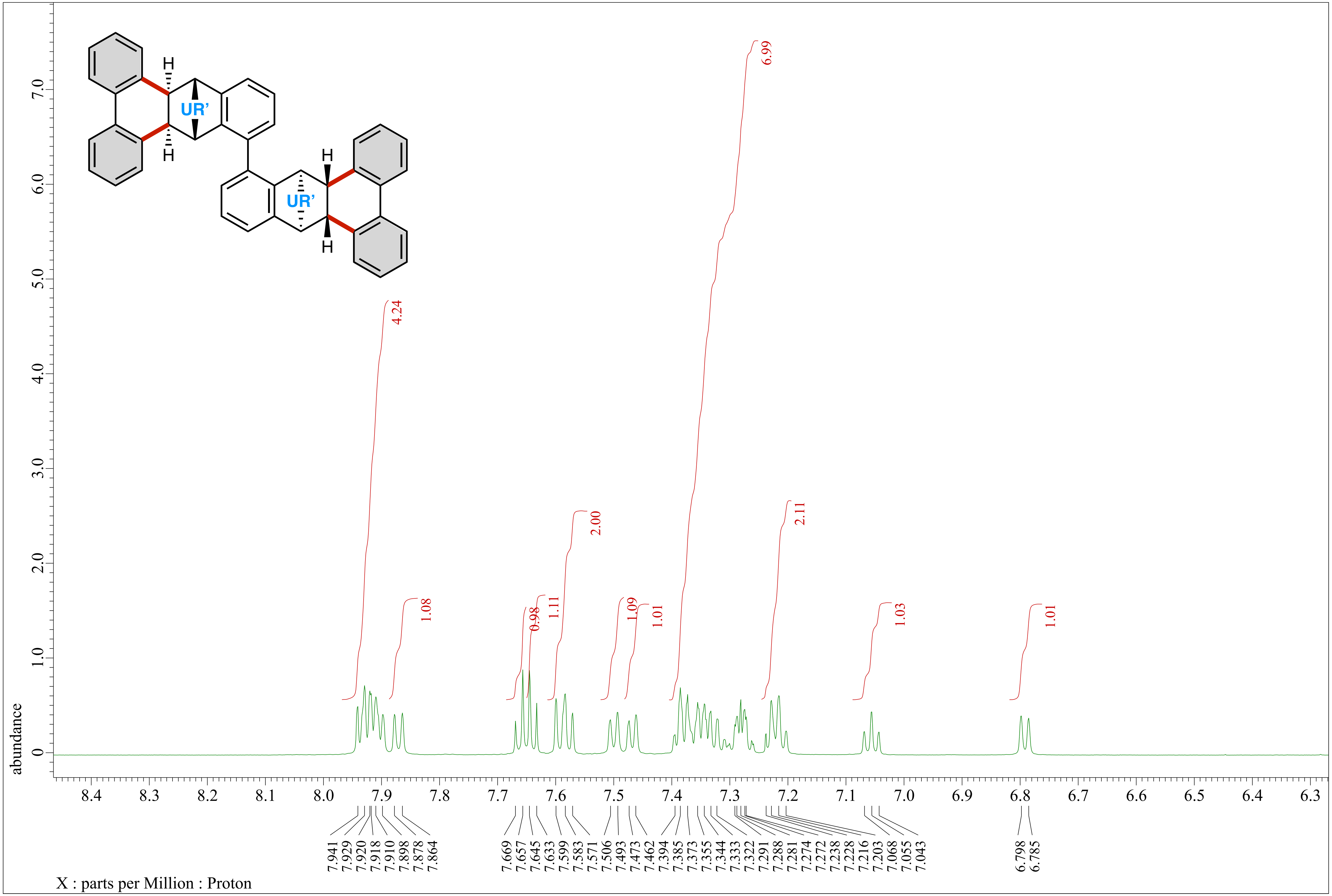

^{13}C NMR spectrum of **6ba-2** (solvent: $CDCl_3$)

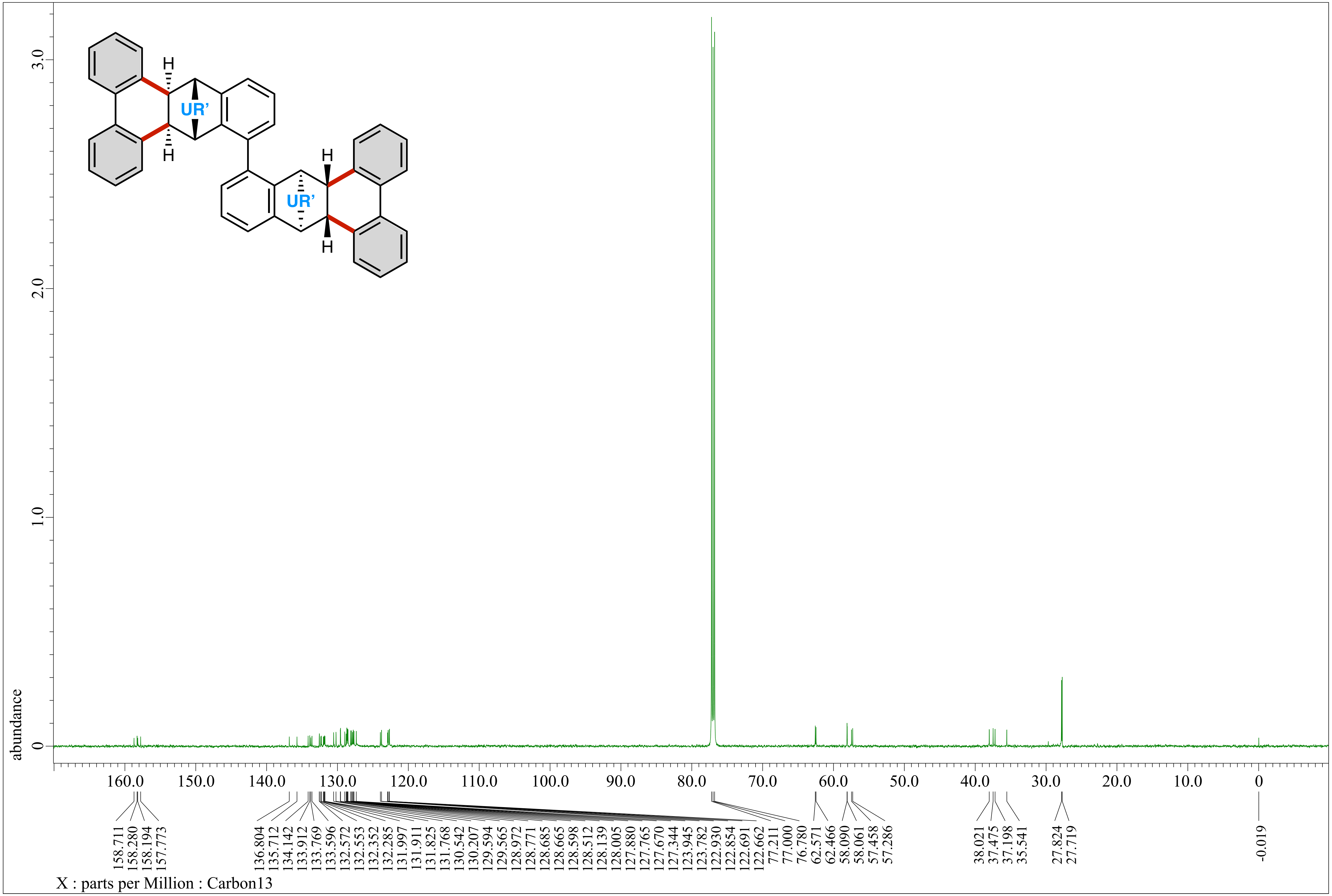

^{13}C NMR spectrum (inset) of **6ba-2** (solvent: $CDCl_3$)

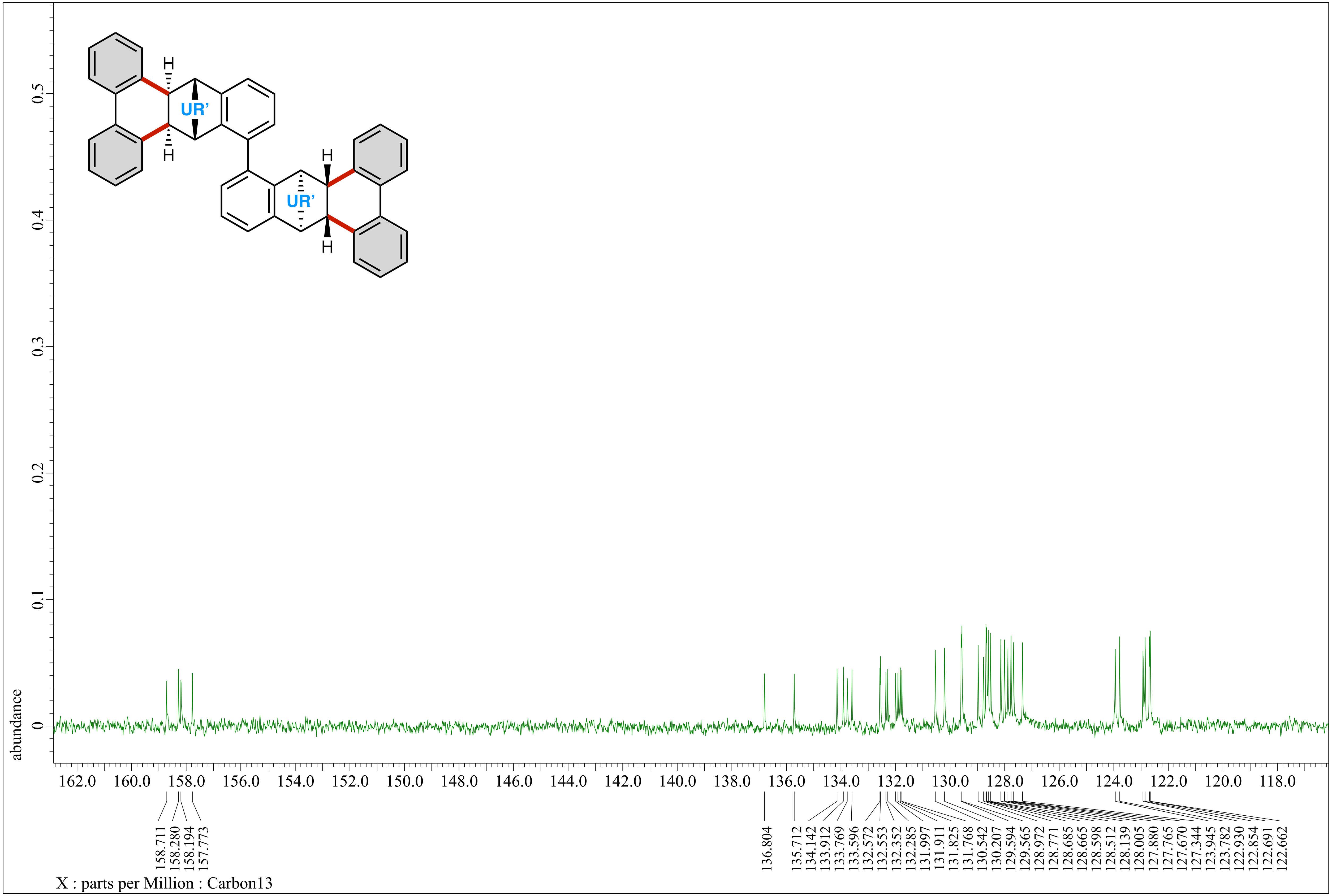

^{1}H NMR spectrum of **7ba** (solvent: $CDCl_3$)

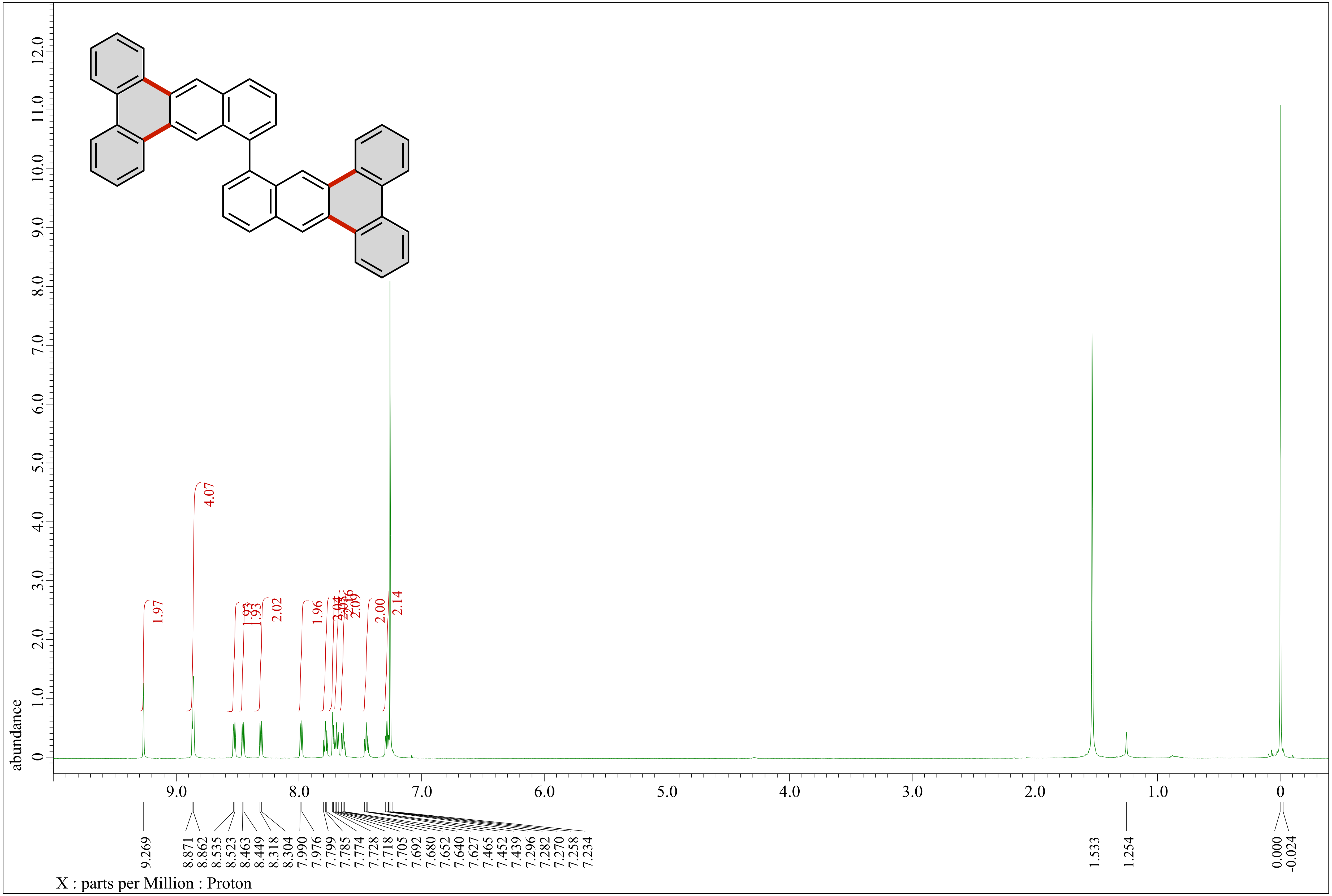

^{1}H NMR spectrum (inset) of **7ba** (solvent: $CDCl_3$)

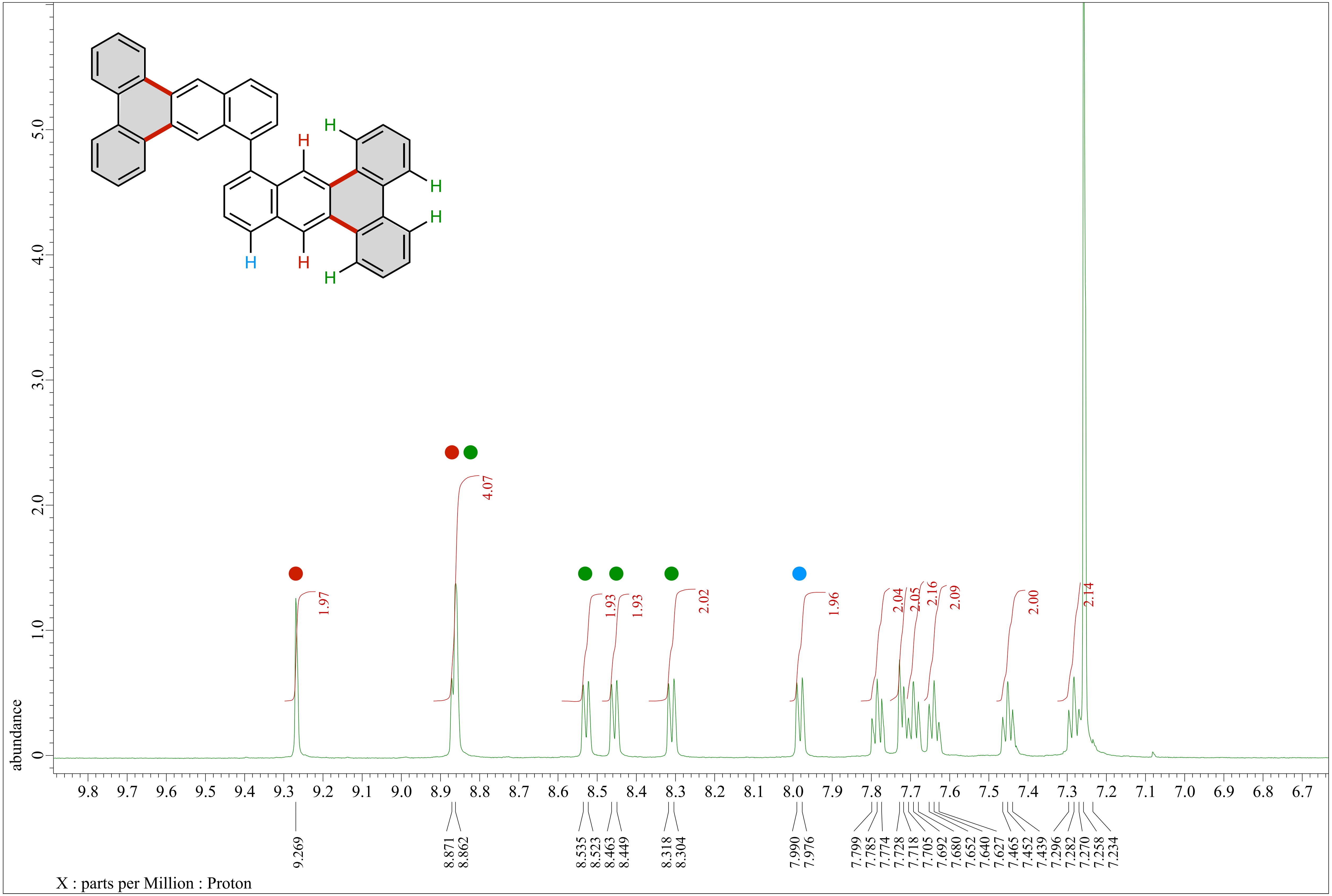

^{13}C NMR spectrum of **7ba** (solvent: $CDCl_3$)

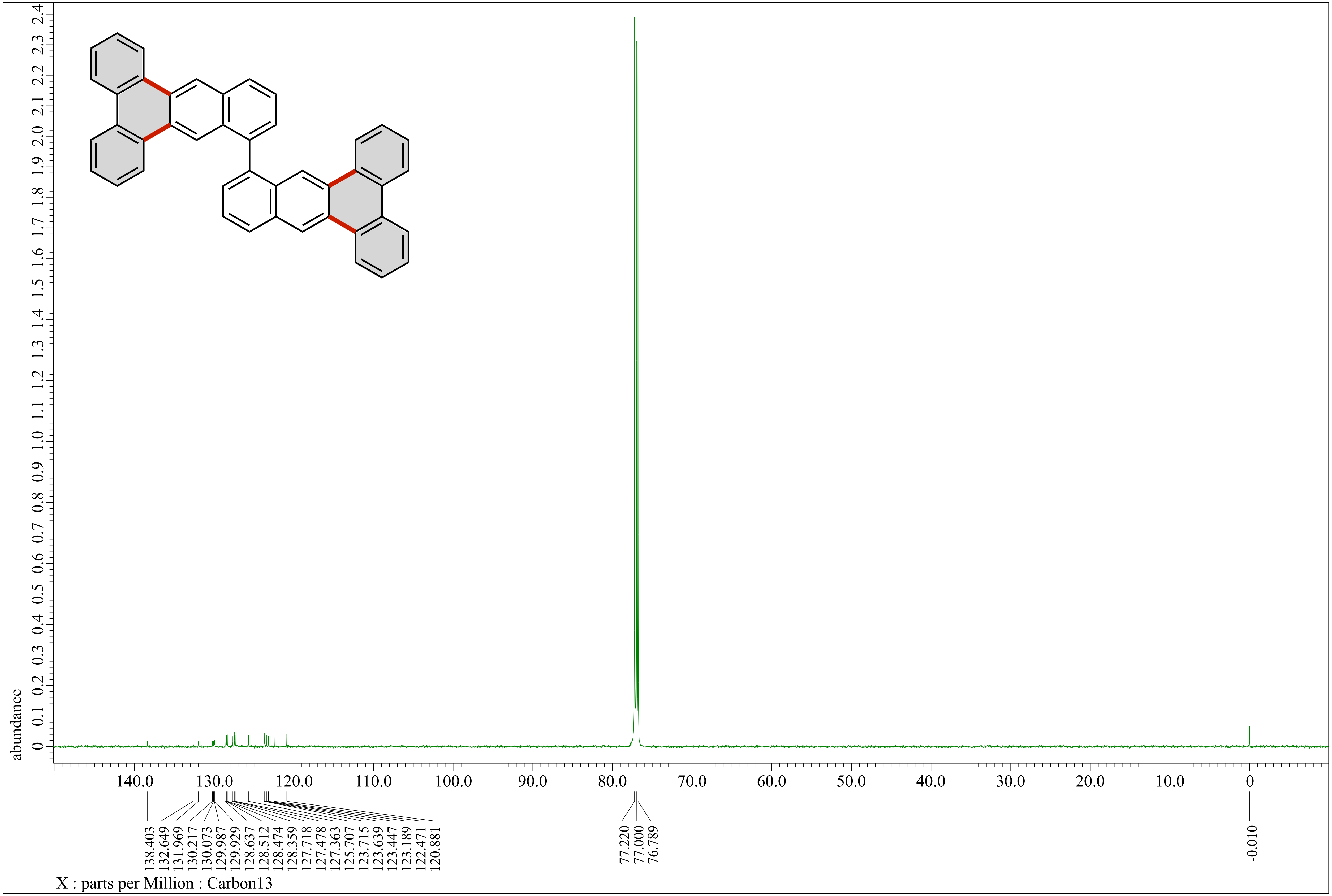

^{13}C NMR spectrum (inset) of **7ba** (solvent: $CDCl_3$)

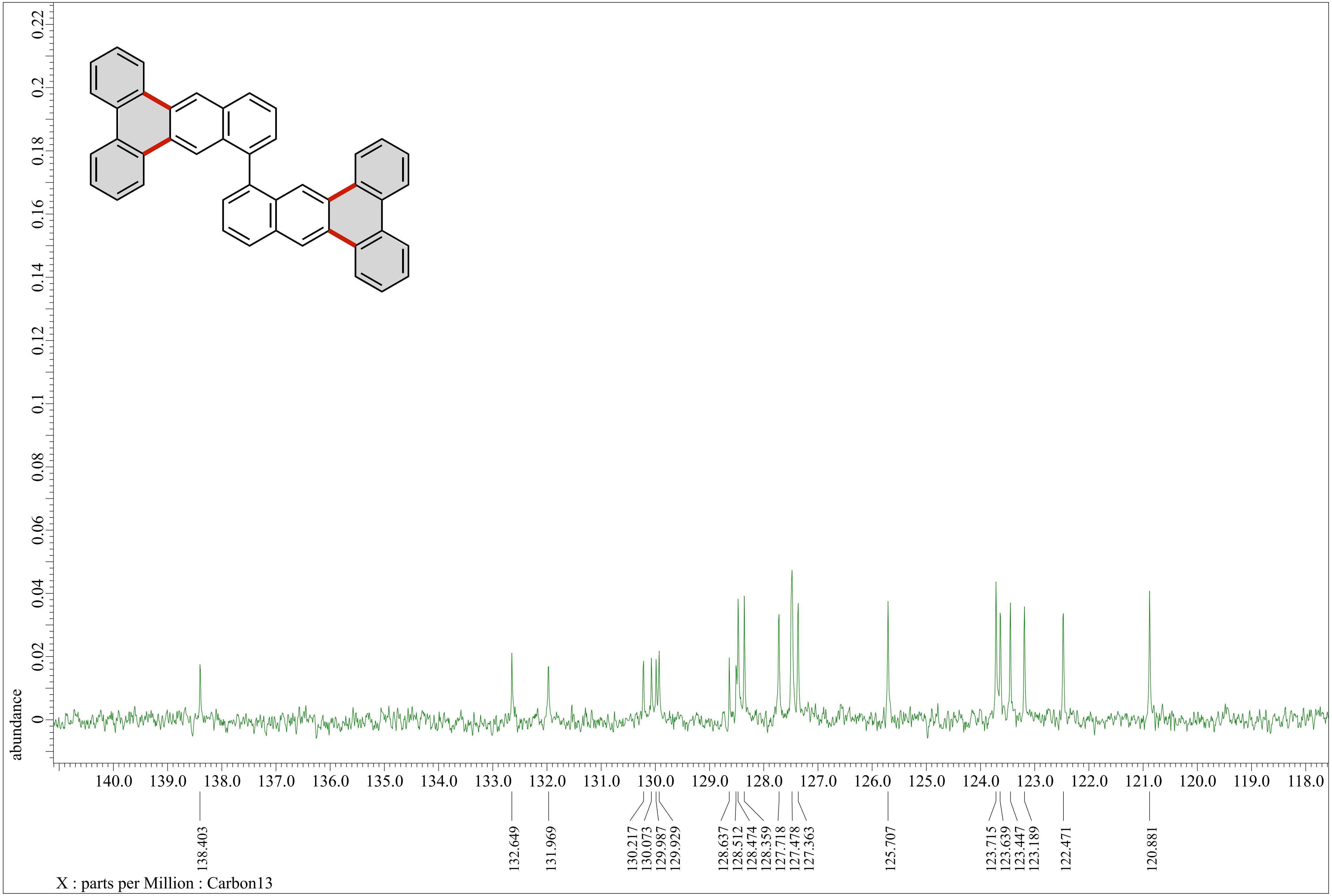

^{1}H NMR spectrum of **9** (solvent: $CDCl_3$)

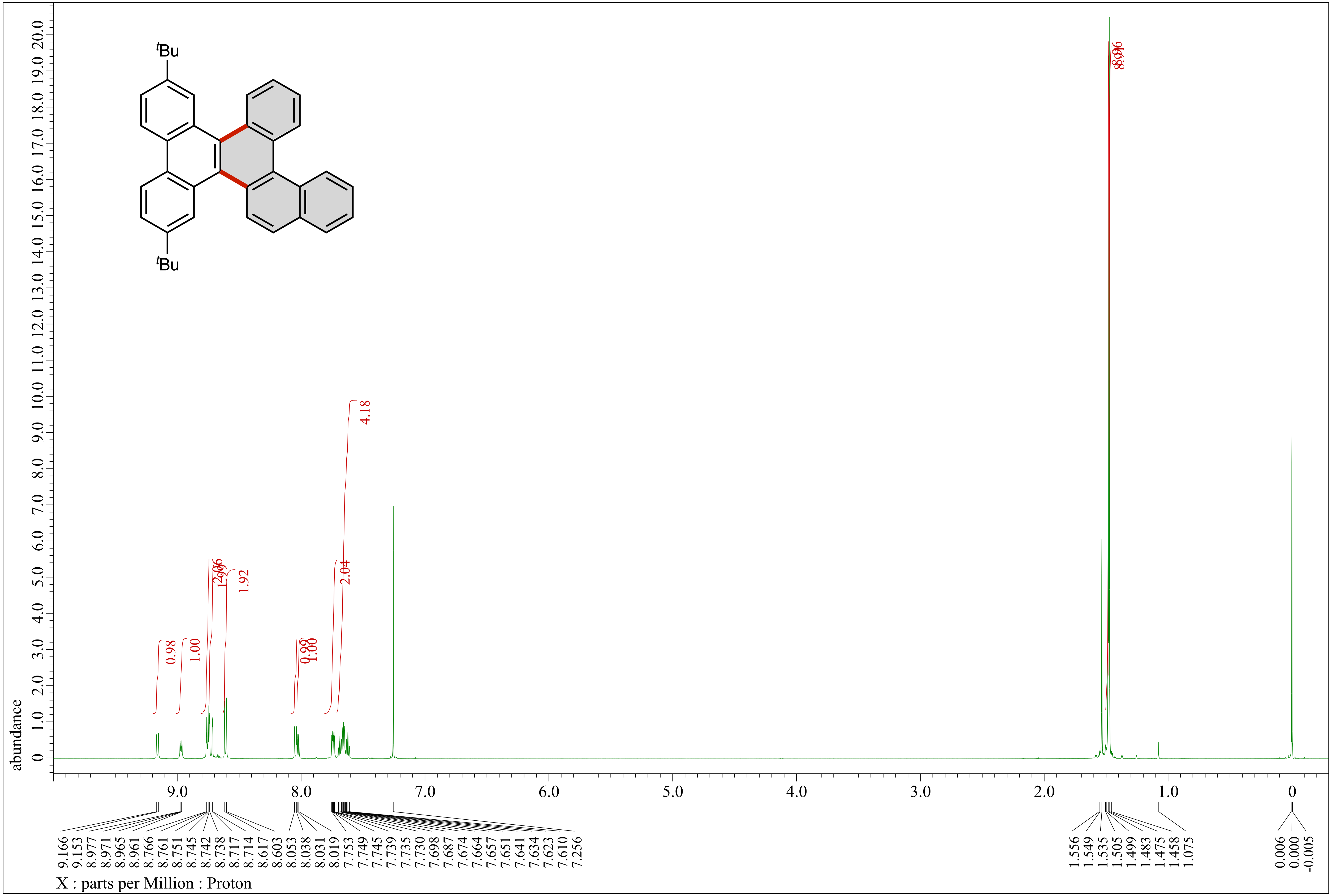

^{1}H NMR spectrum (inset) of **9** (solvent: $CDCl_3$)

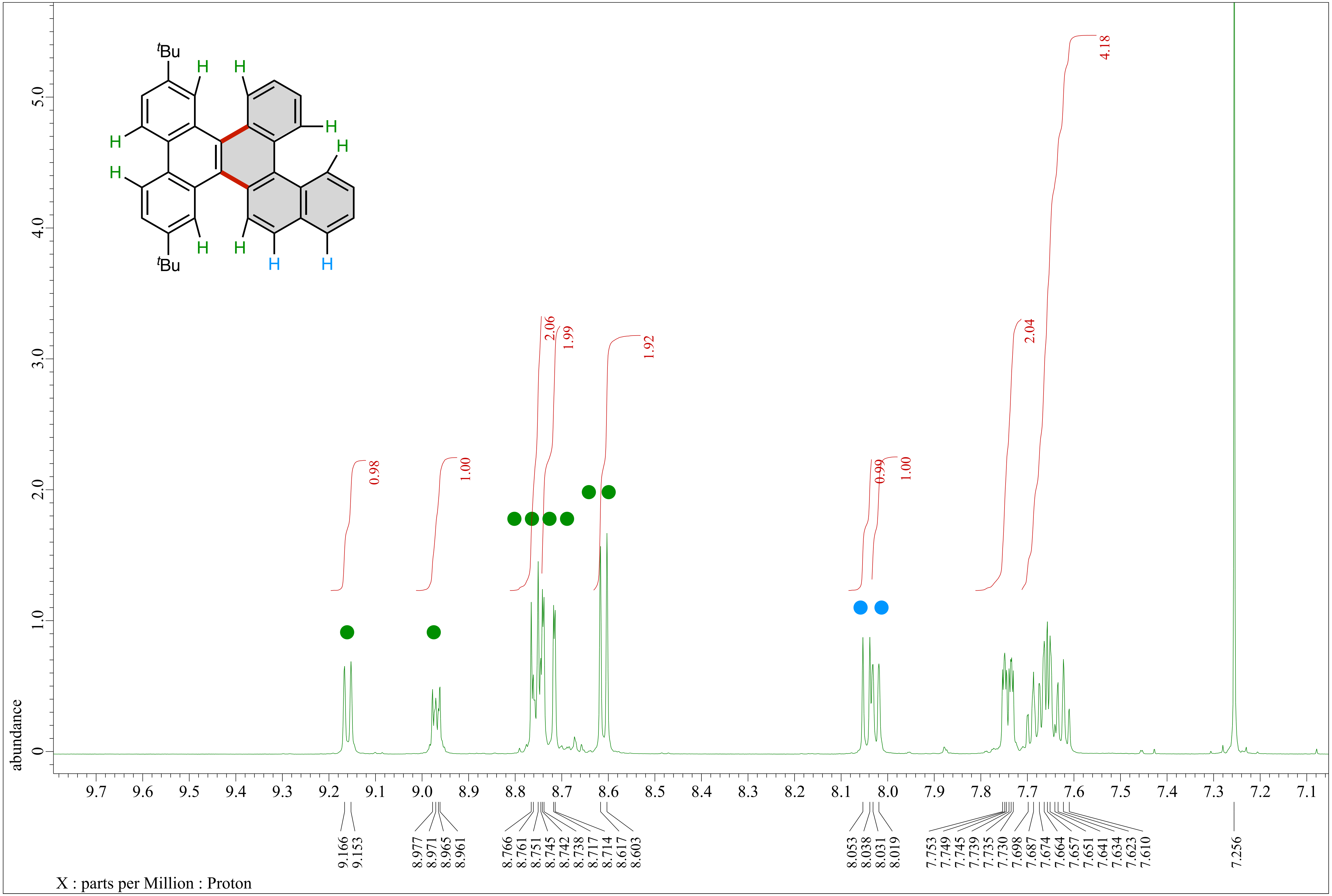

^{13}C NMR spectrum of **9** (solvent: $CDCl_3$)

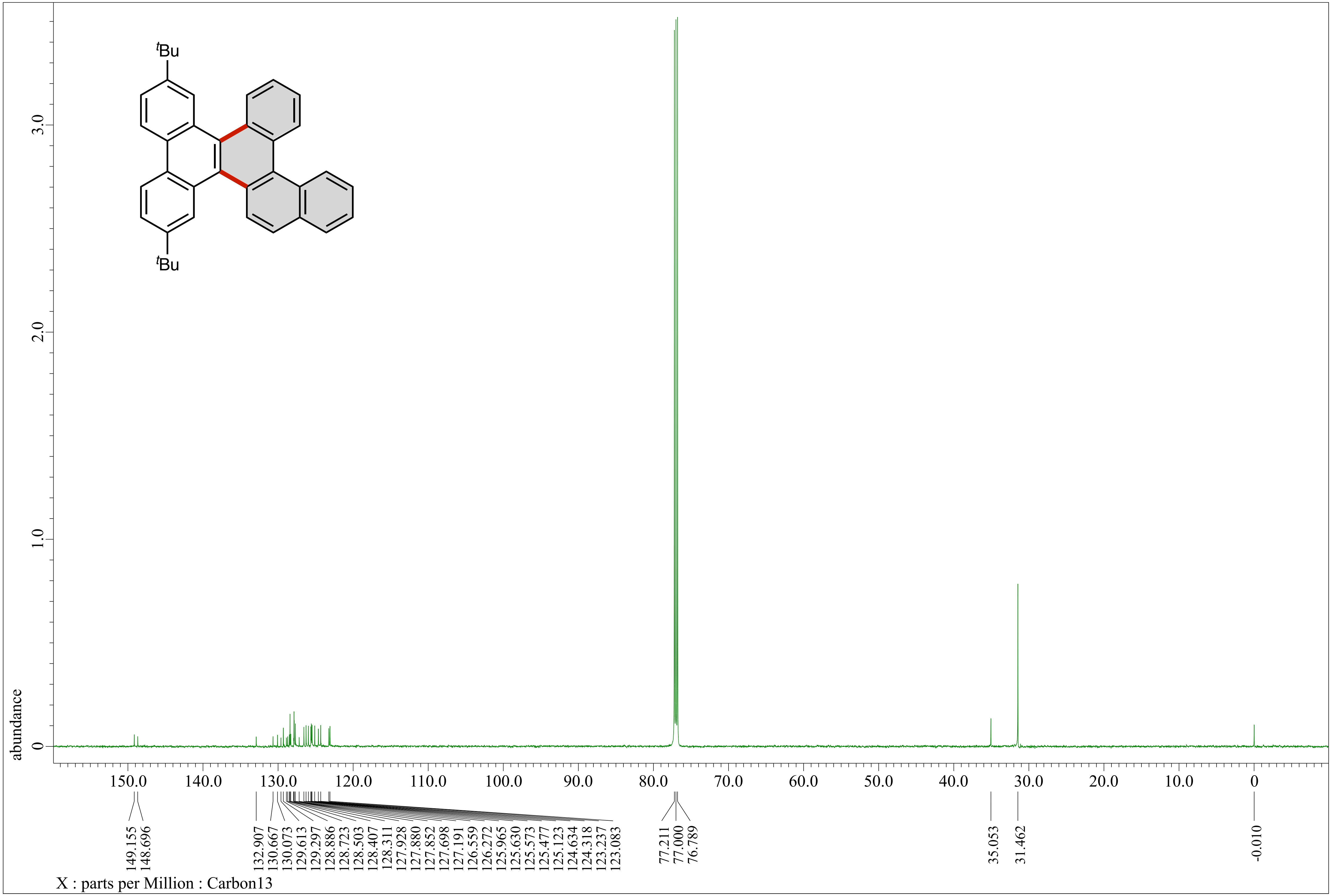

^{13}C NMR spectrum (inset) of **9** (solvent: $CDCl_3$)

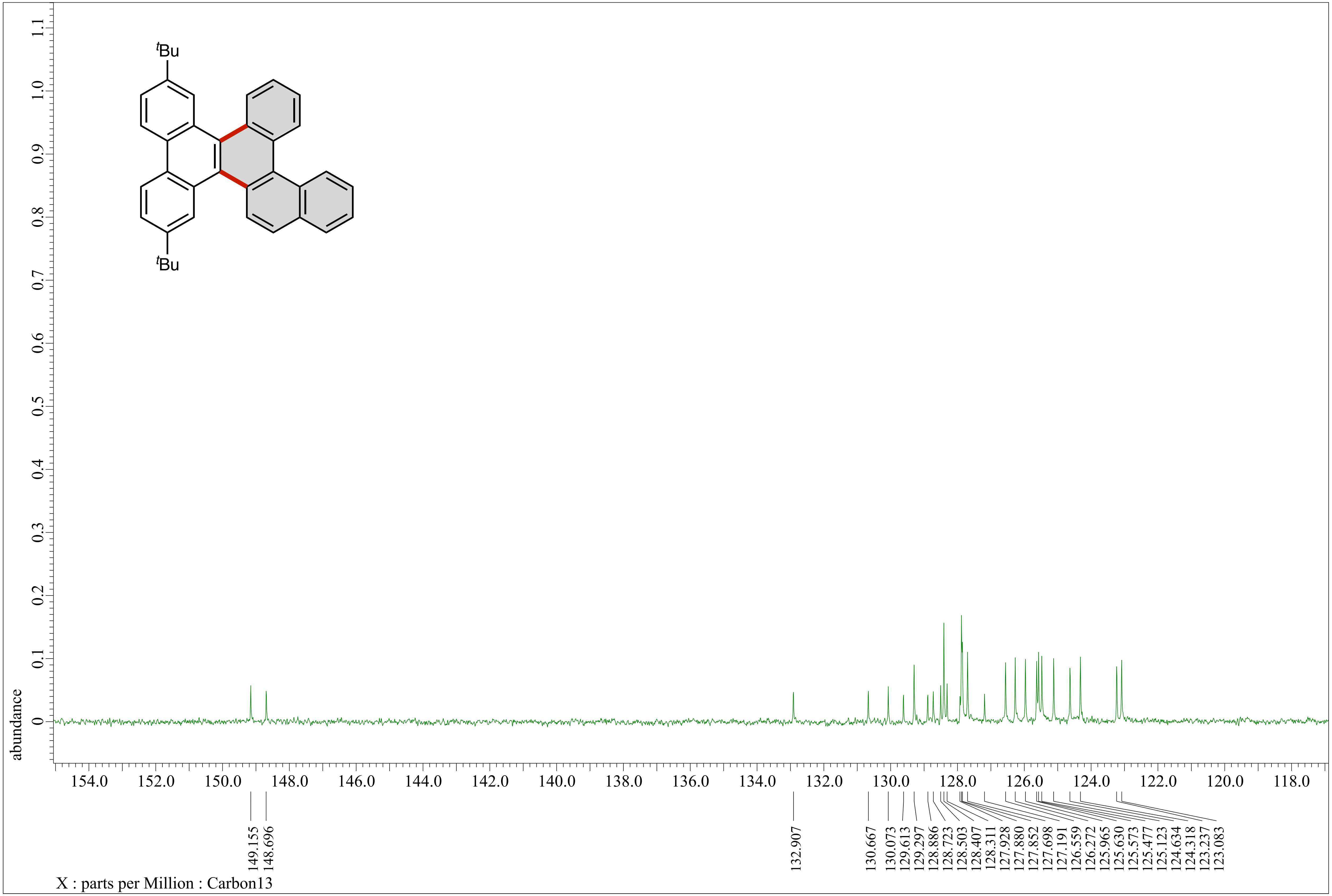

^{1}H NMR spectrum of **10** (solvent: $CDCl_3$)

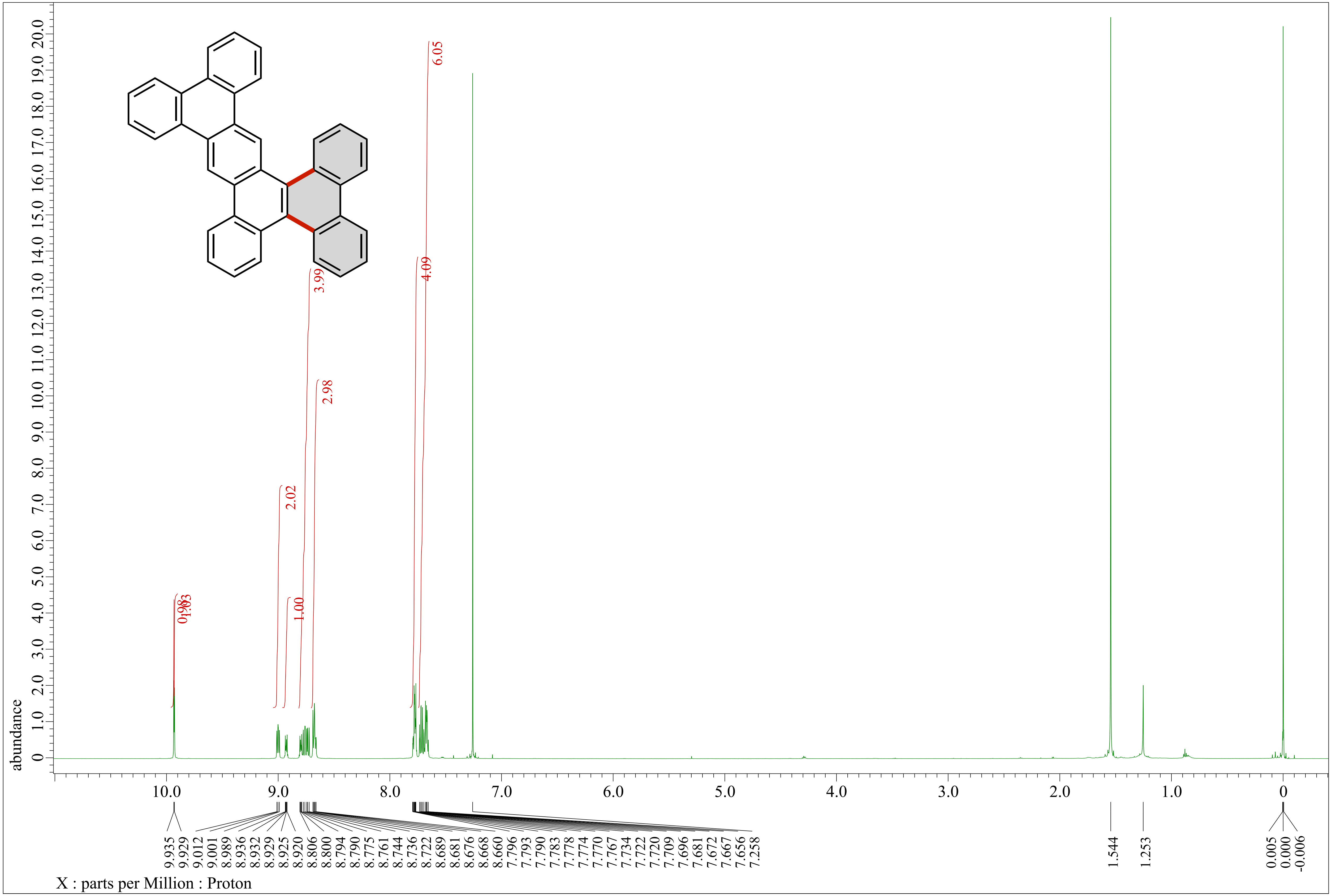

^{1}H NMR spectrum (inset) of **10** (solvent: $CDCl_3$)

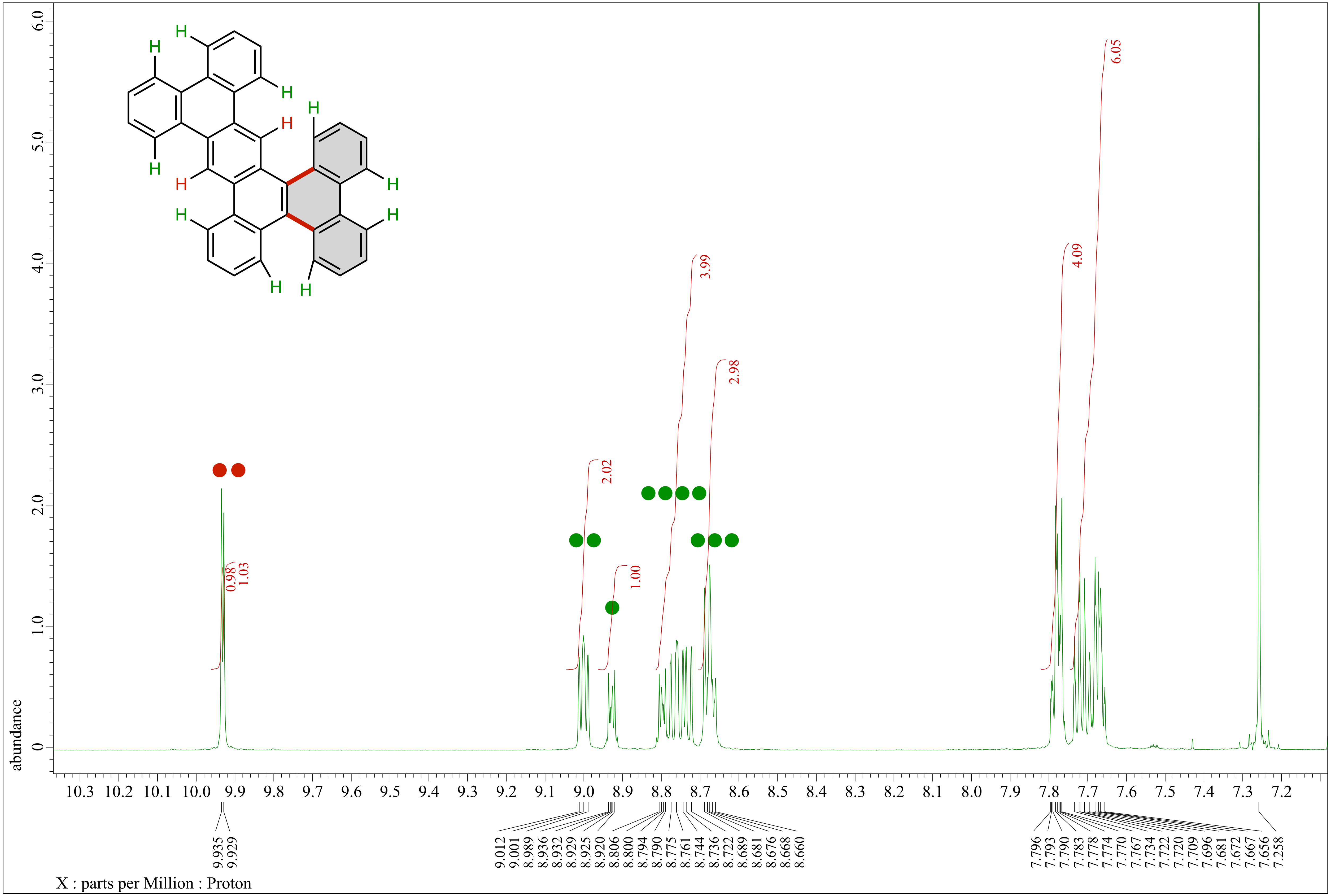

^{13}C NMR spectrum of **10** (solvent: $CDCl_3$)

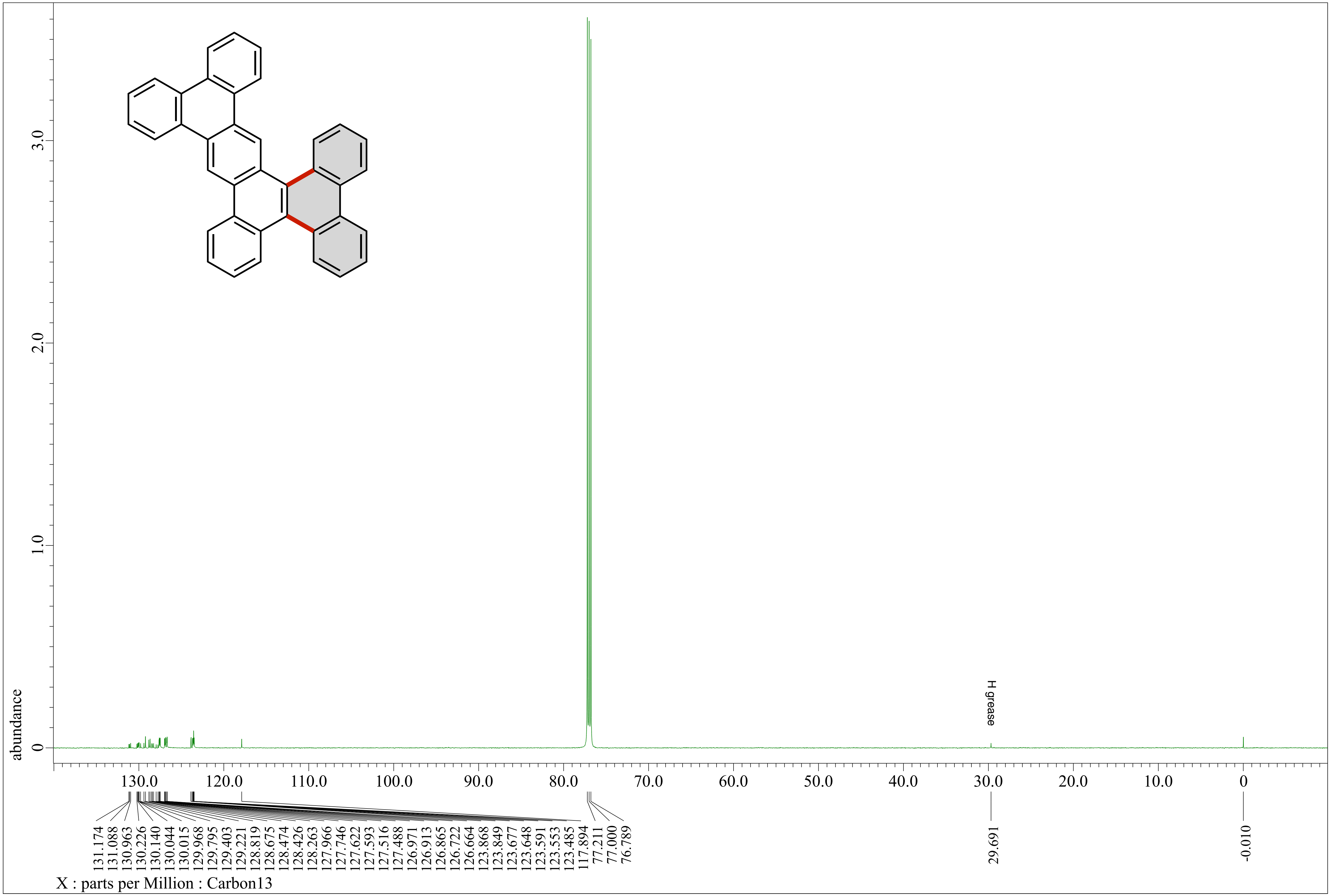

^{13}C NMR spectrum (inset) of **10** (solvent: $CDCl_3$)

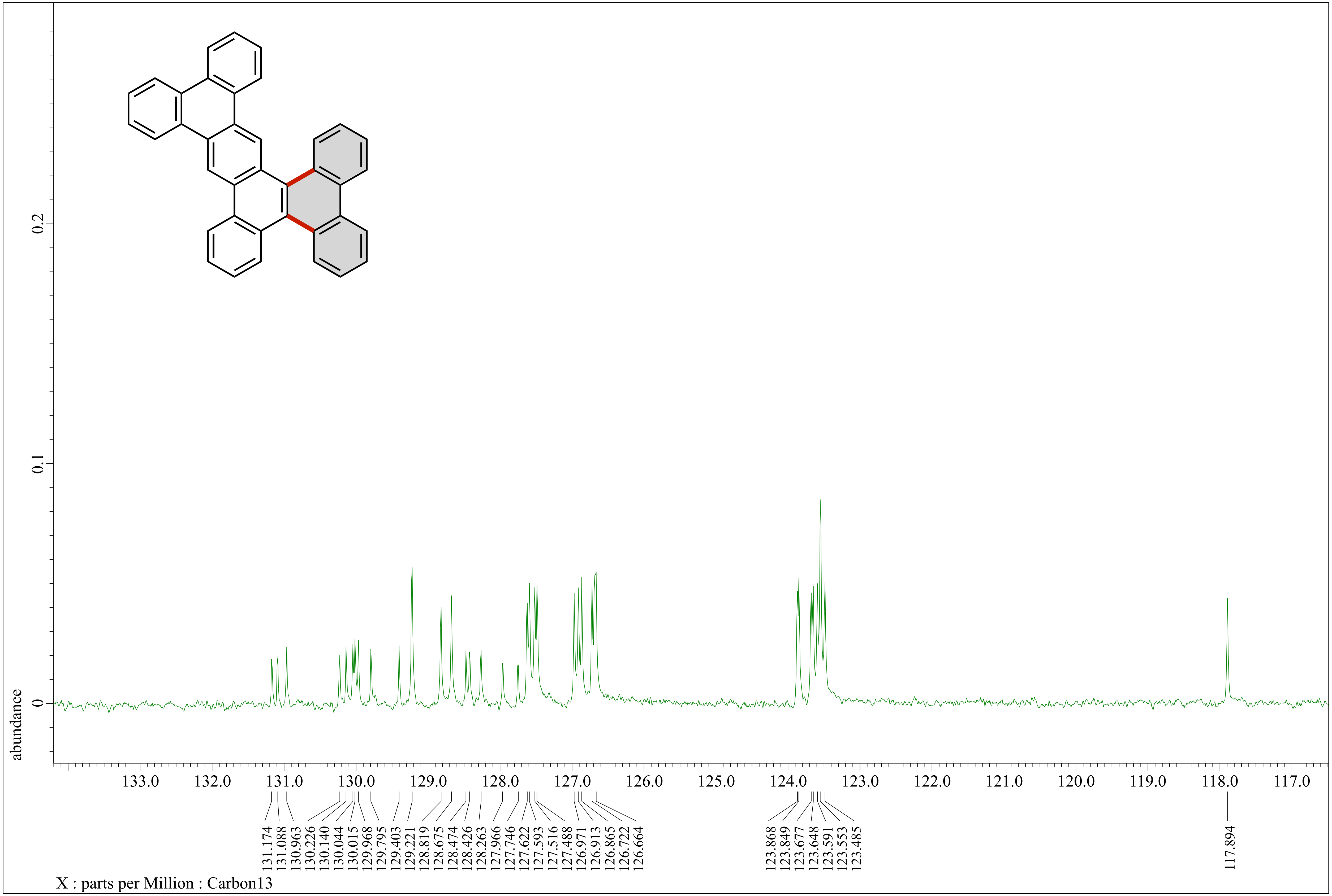

^{1}H NMR spectrum of **S1** (solvent: $CDCl_3$)

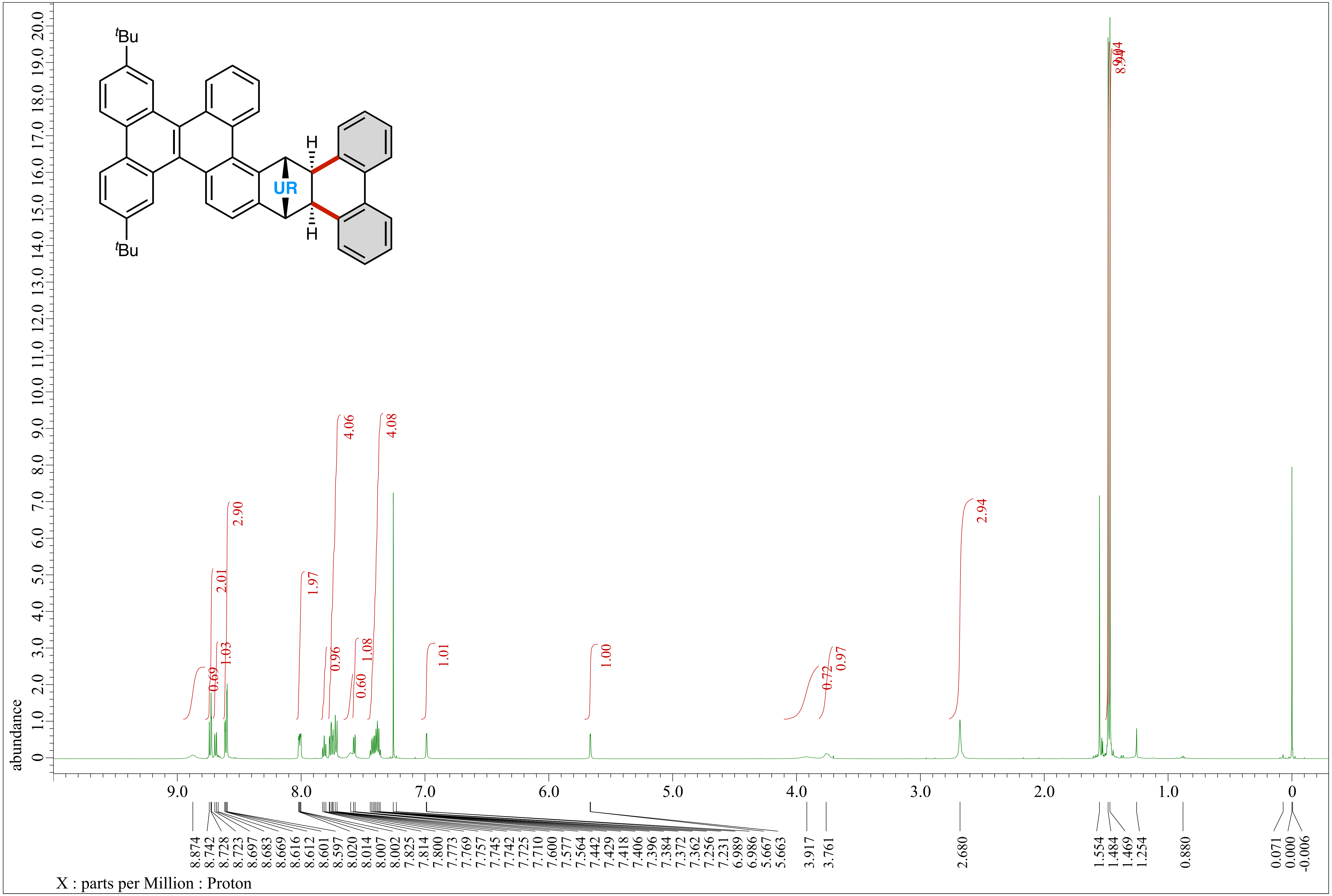

^{1}H NMR spectrum (inset) of **S1** (solvent: $CDCl_3$)

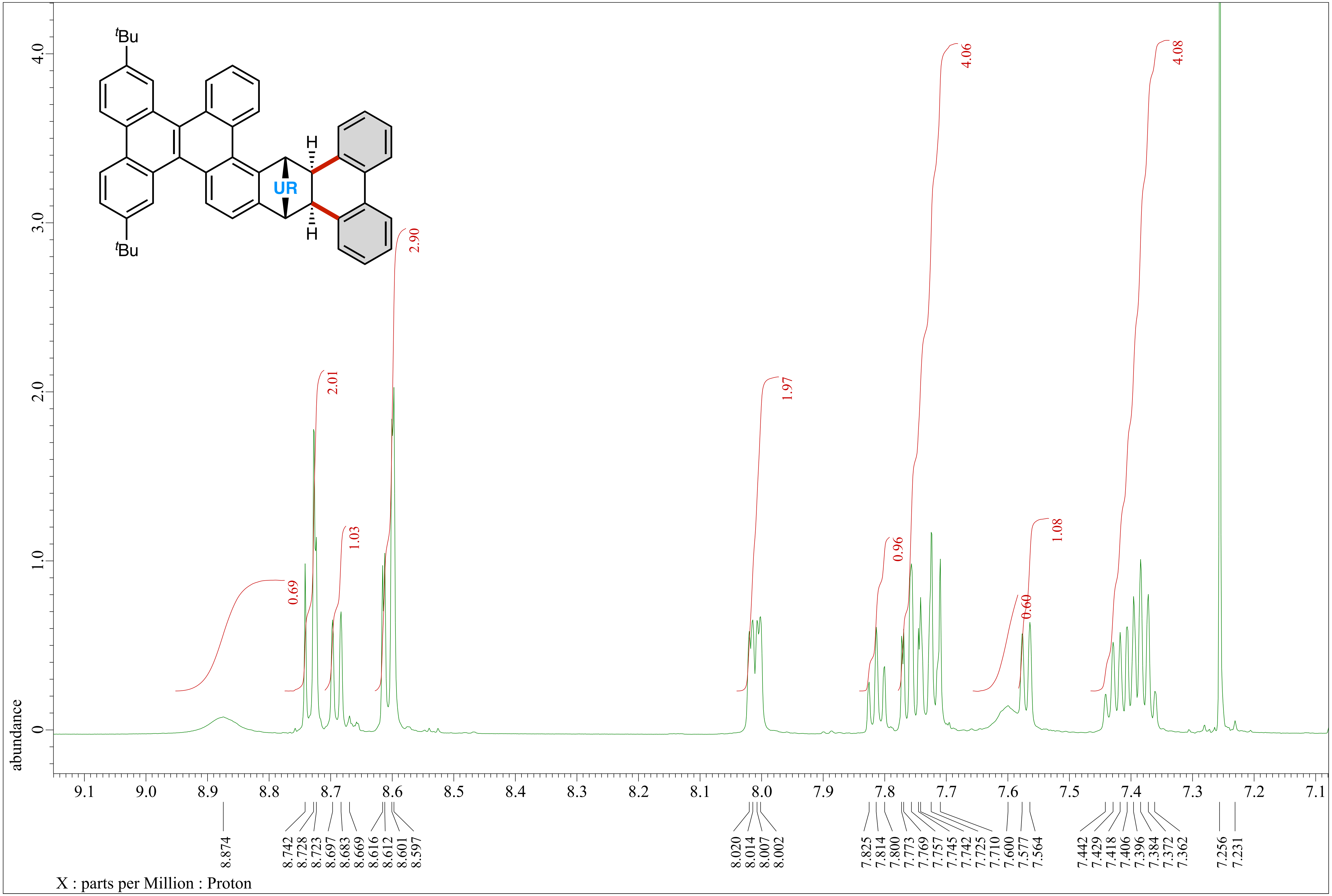

^{13}C NMR spectrum of **S1** (solvent: $CDCl_3$)

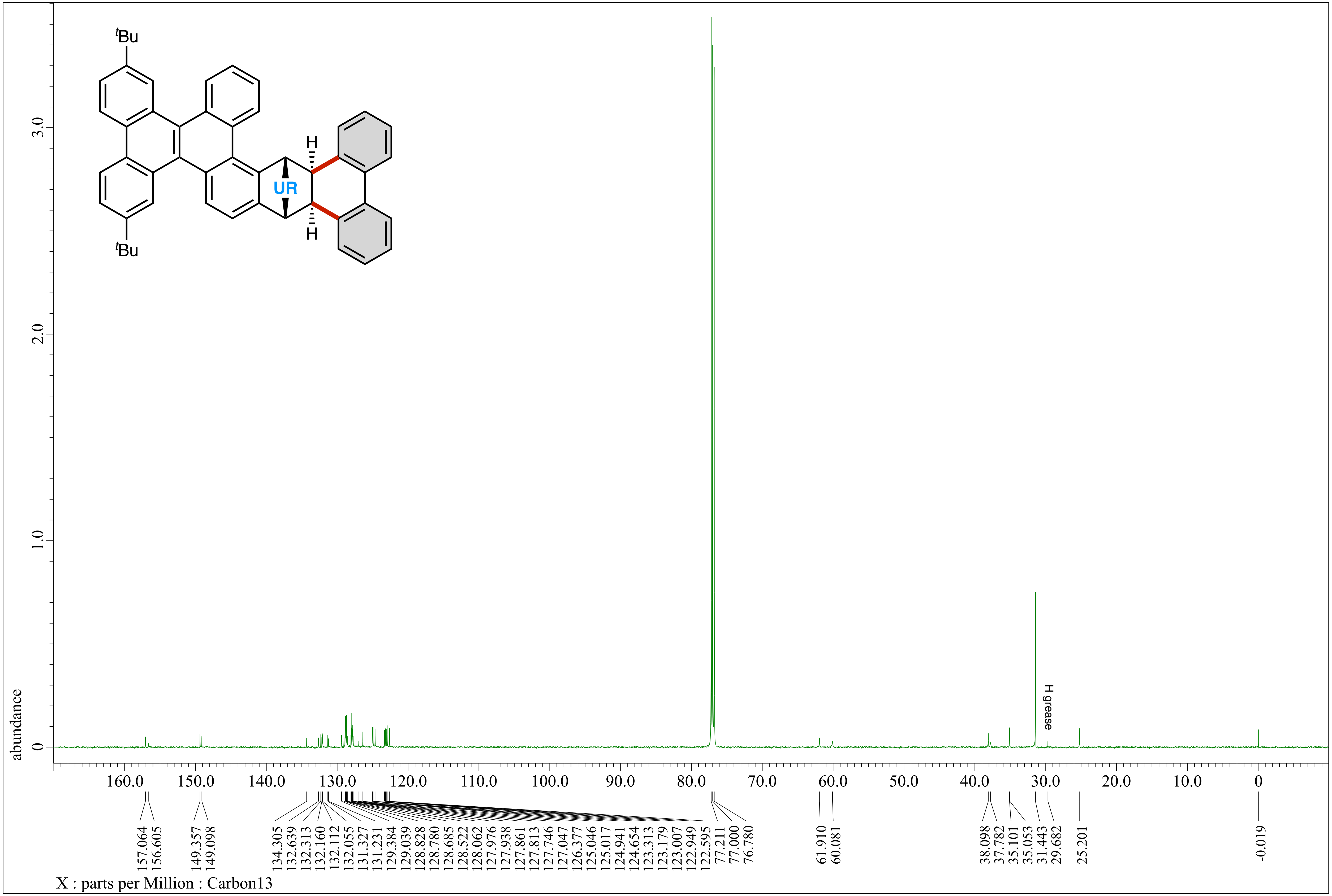

^{13}C NMR spectrum (inset) of **S1** (solvent: $CDCl_3$)

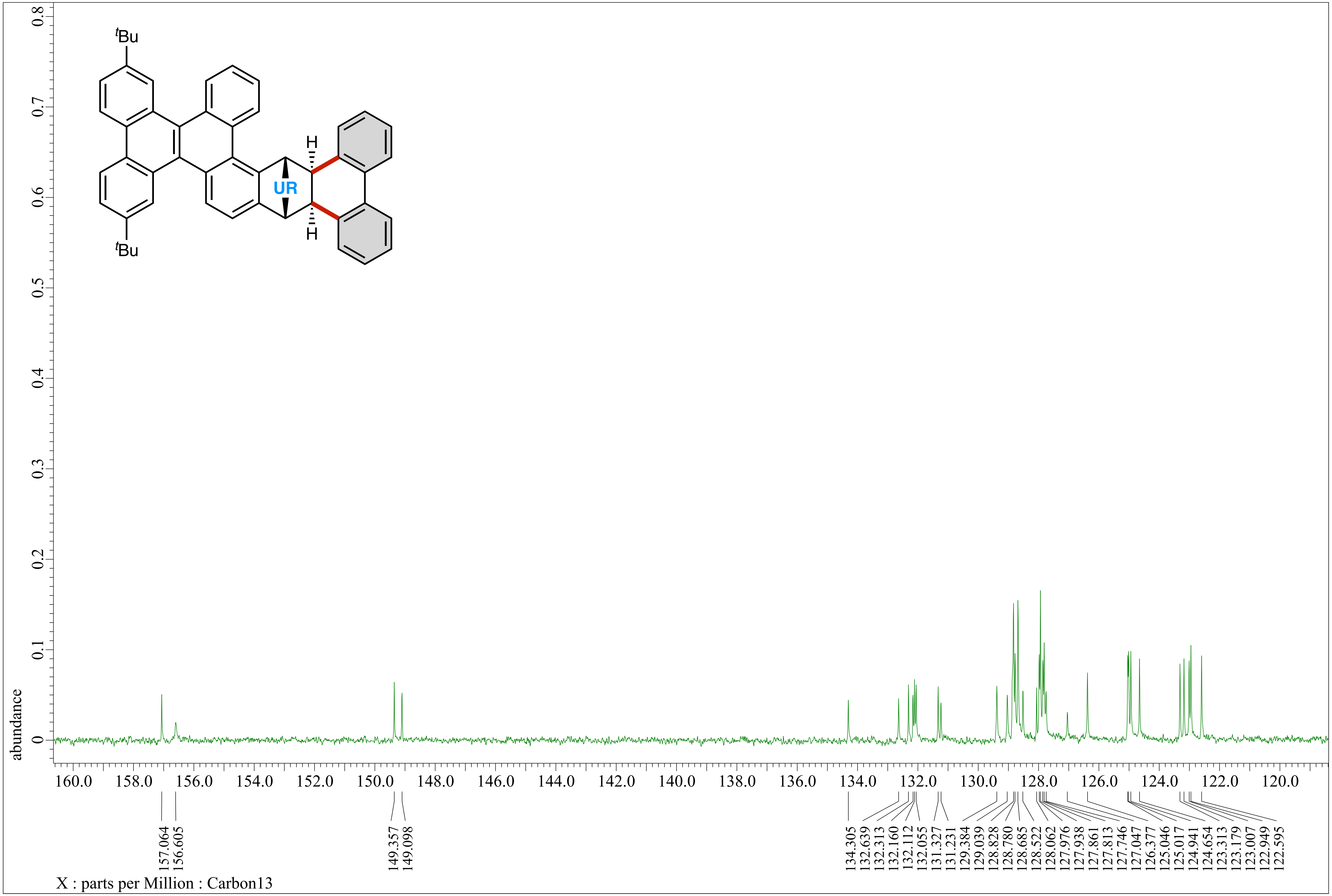

^{1}H NMR spectrum of **11** (solvent: $CDCl_3$)

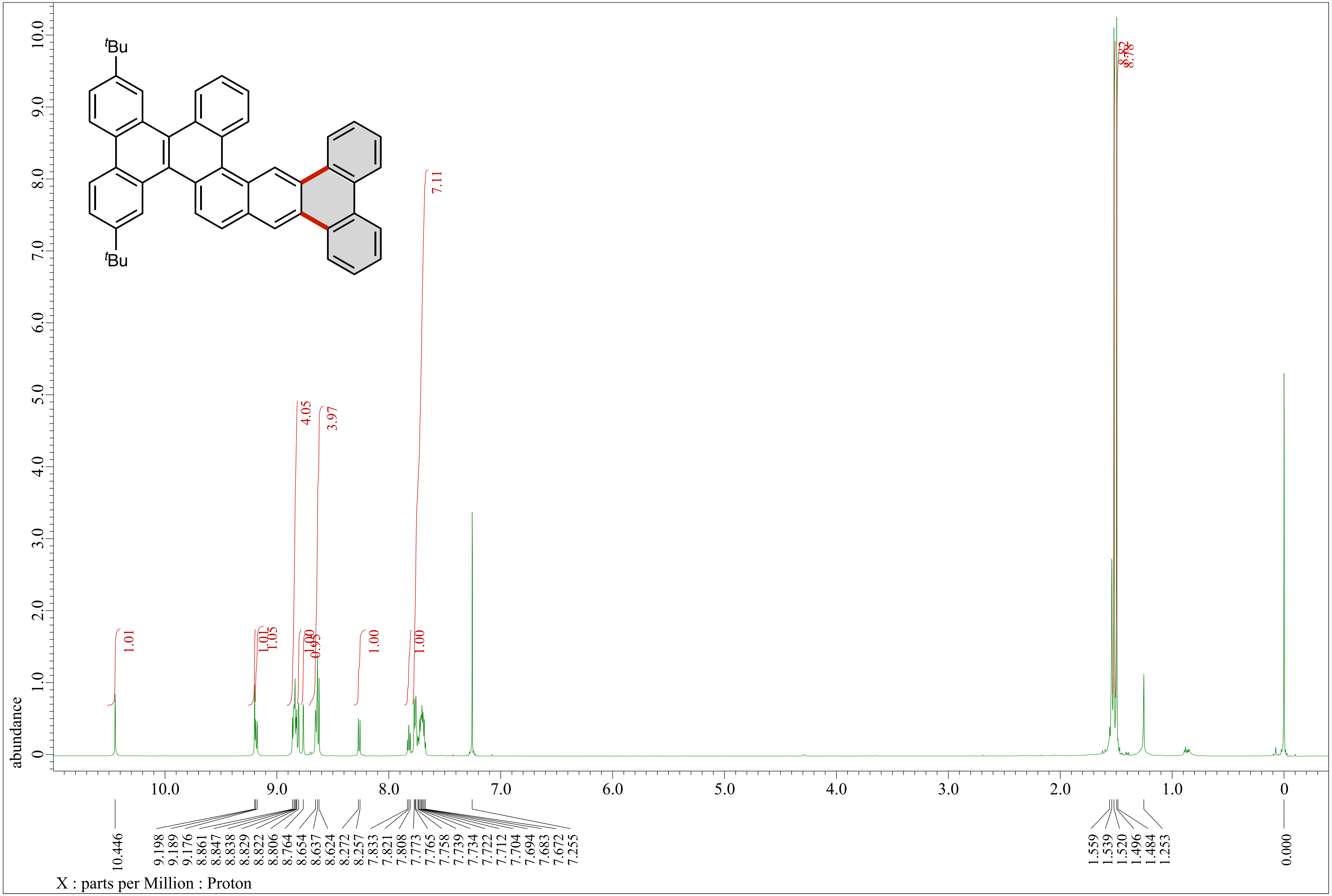

^{1}H NMR spectrum (inset) of **11** (solvent: $CDCl_3$)

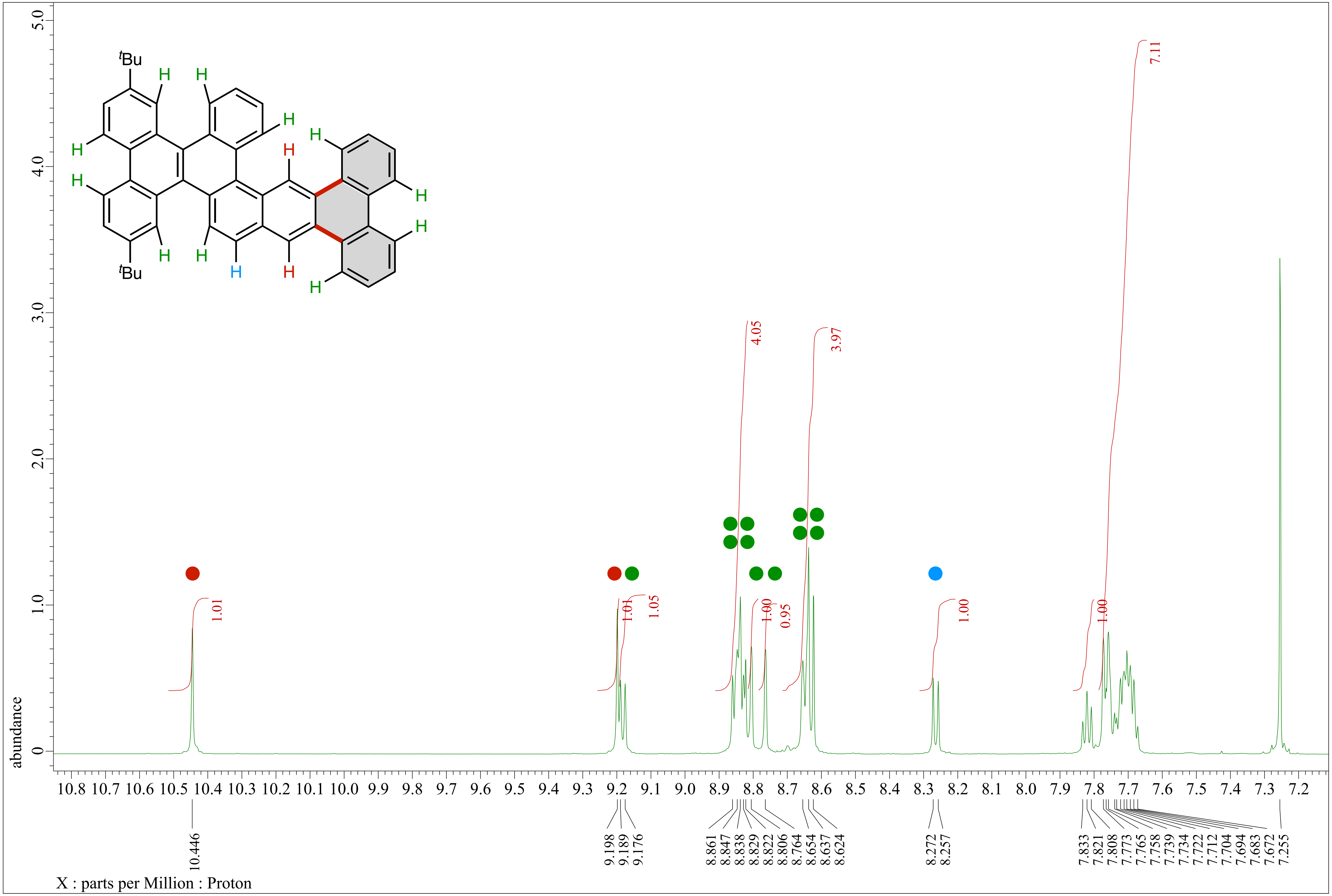

^{13}C NMR spectrum of **11** (solvent: $CDCl_3$)

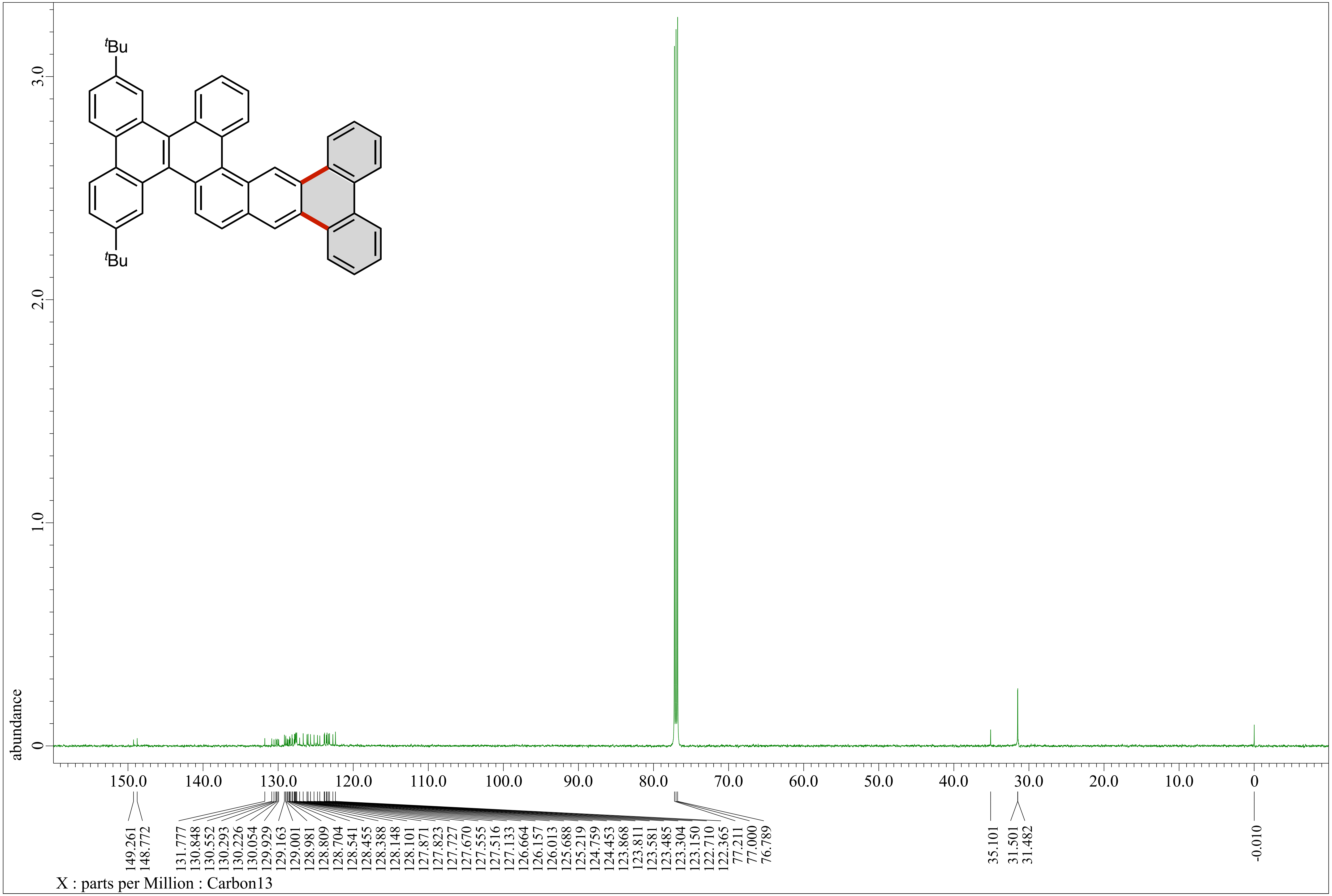

^{13}C NMR spectrum (inset) of **11** (solvent: $CDCl_3$)

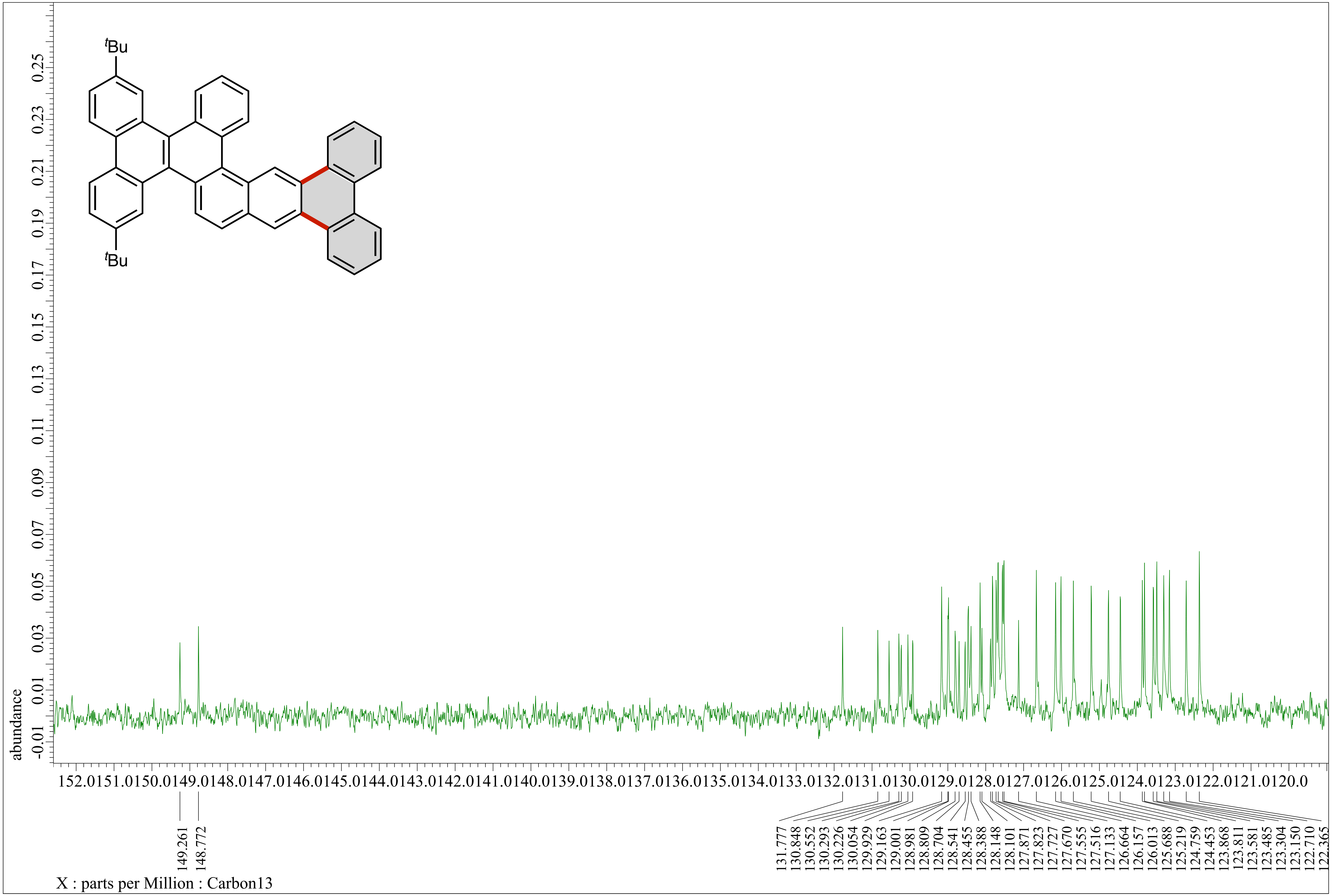

^{1}H NMR spectrum of **6ha** (solvent: $CDCl_3$)

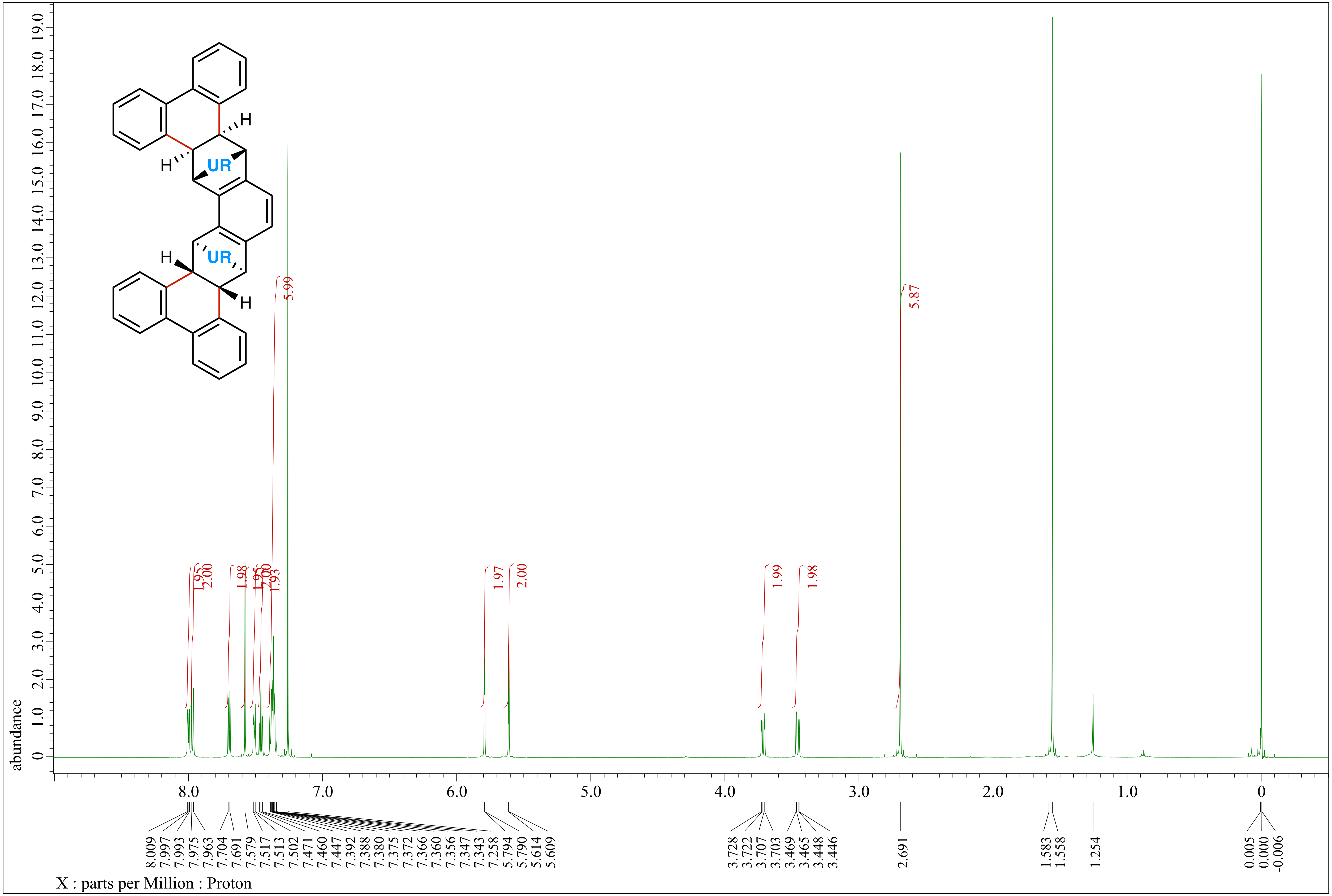

^{1}H NMR spectrum (inset) of **6ha** (solvent: $CDCl_3$)

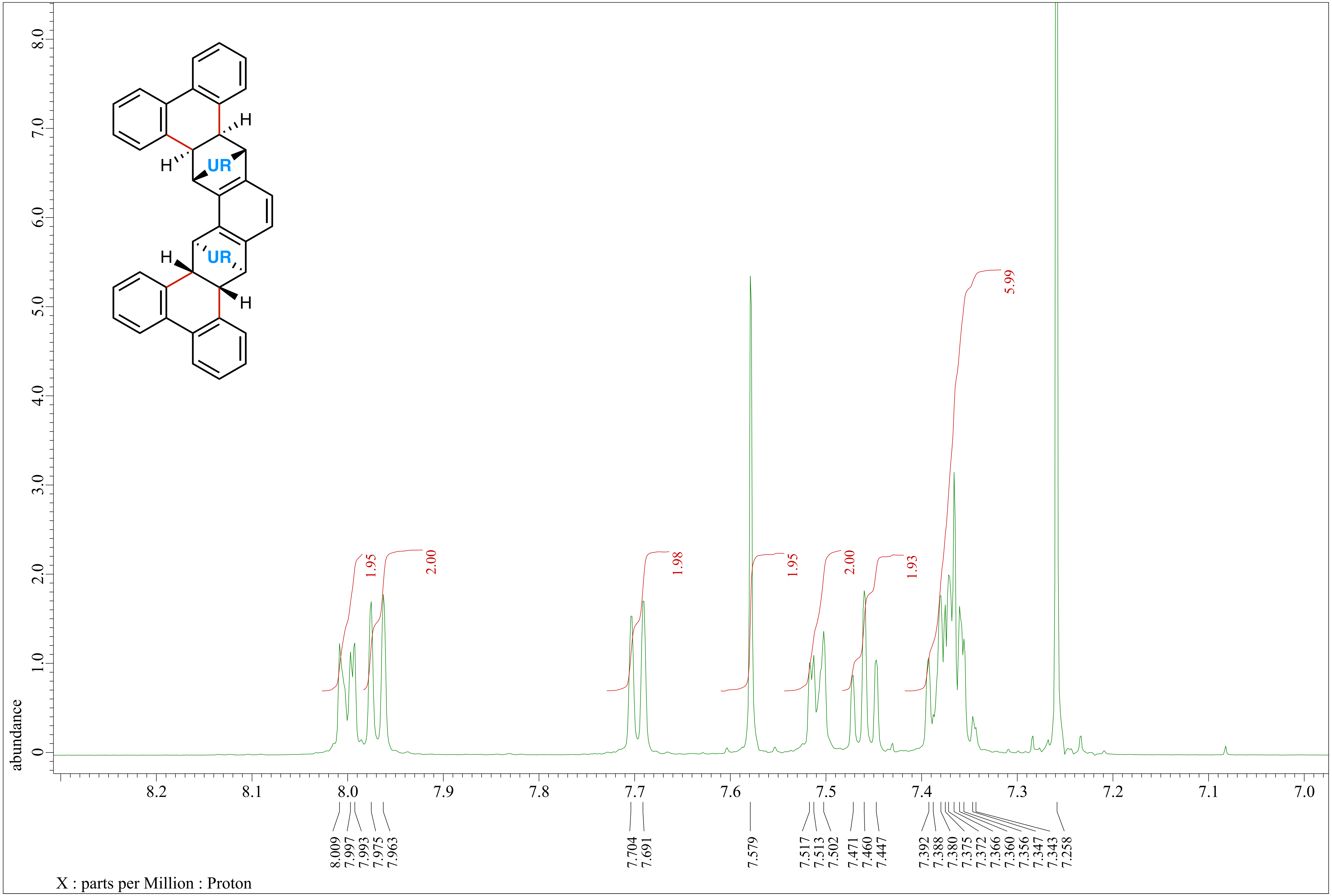

^{13}C NMR spectrum of **6ha** (solvent: $CDCl_3$)

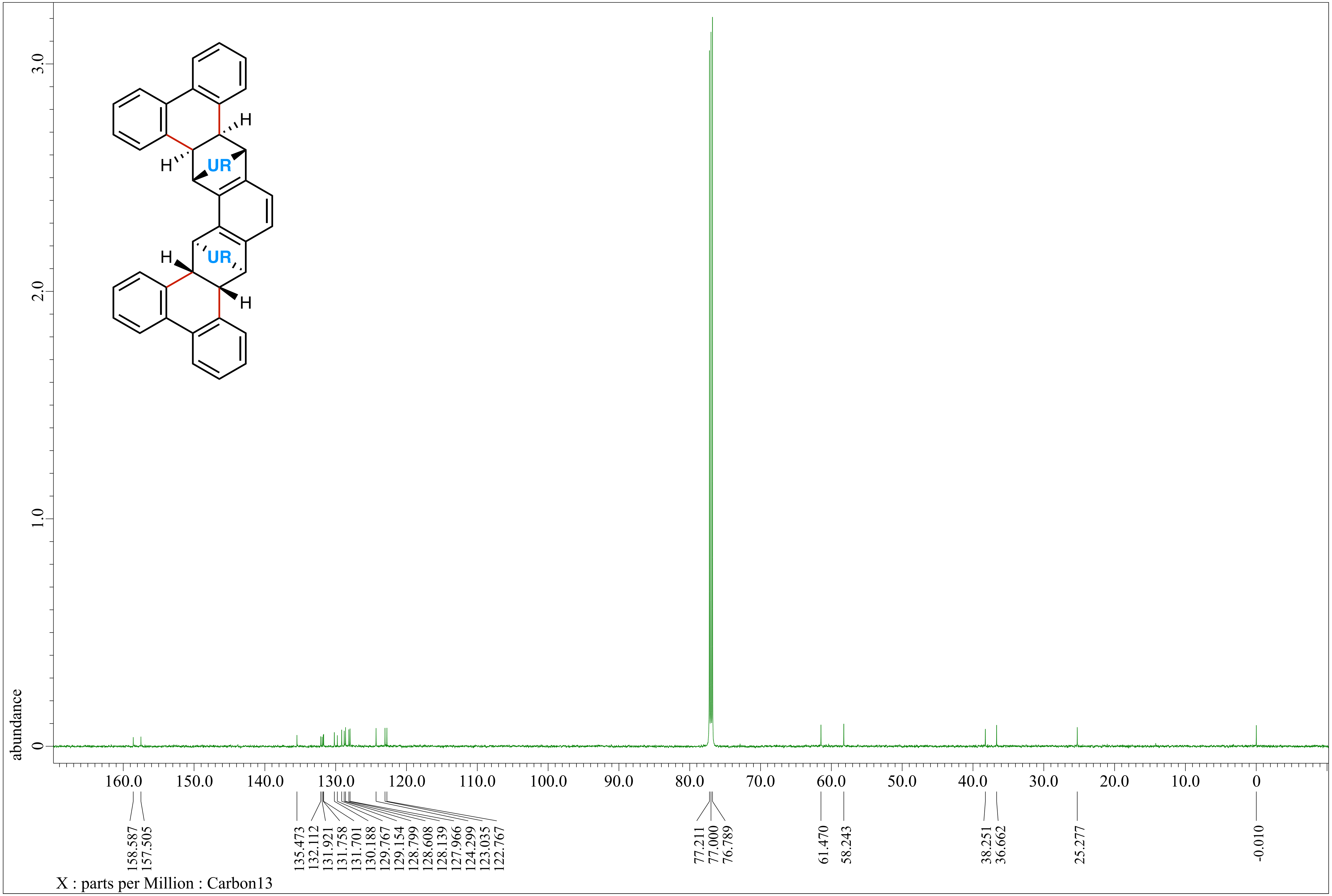

^{13}C NMR spectrum (inset) of **6ha** (solvent: $CDCl_3$)

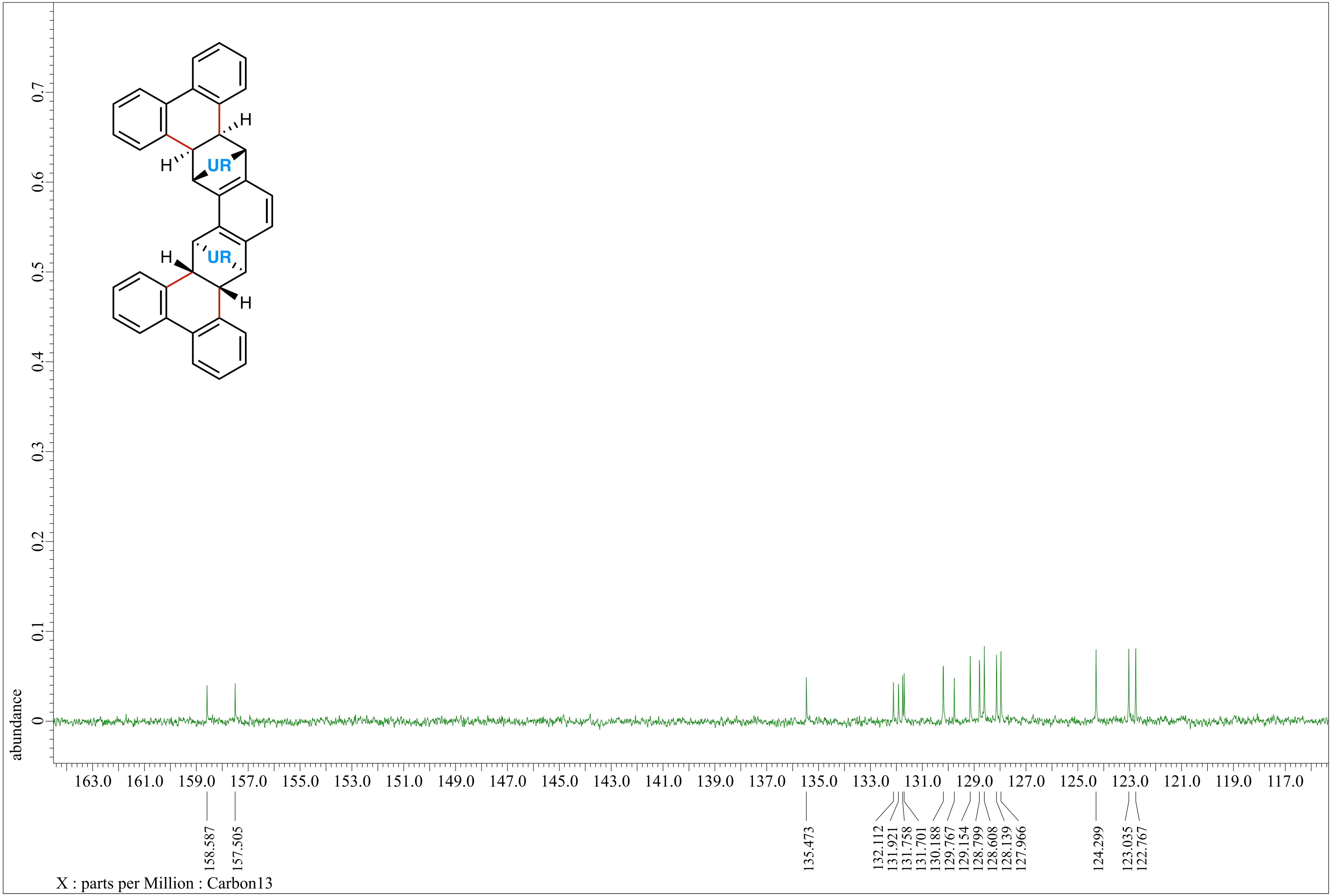

^{1}H NMR spectrum of **12** (solvent: $Cl_2CDCDCl_2$)

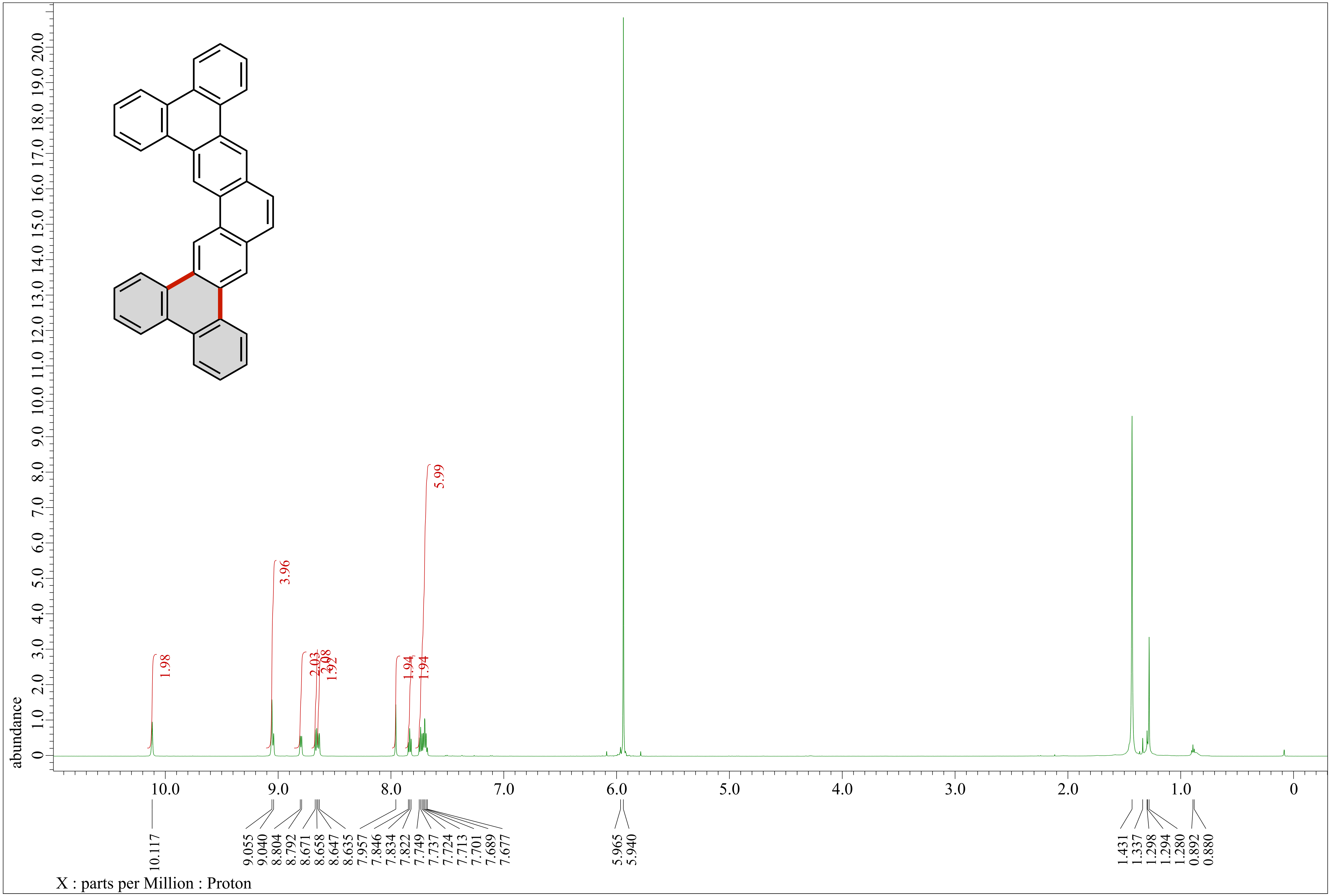

^{1}H NMR spectrum (inset) of **12** (solvent: $Cl_2CDCDCl_2$)

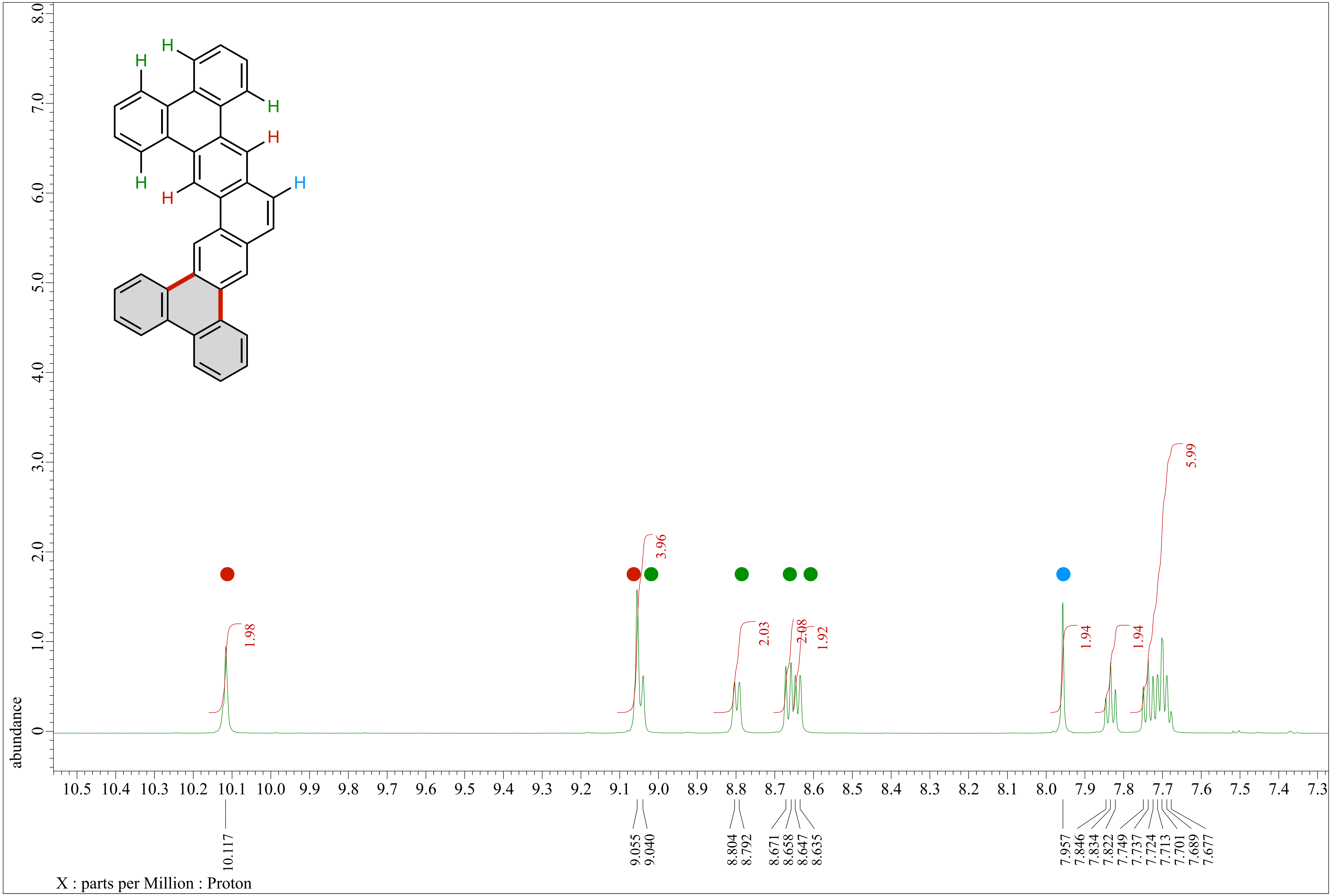

^{13}C NMR spectrum of **12** (solvent: $Cl_2CDCDCl_2$)

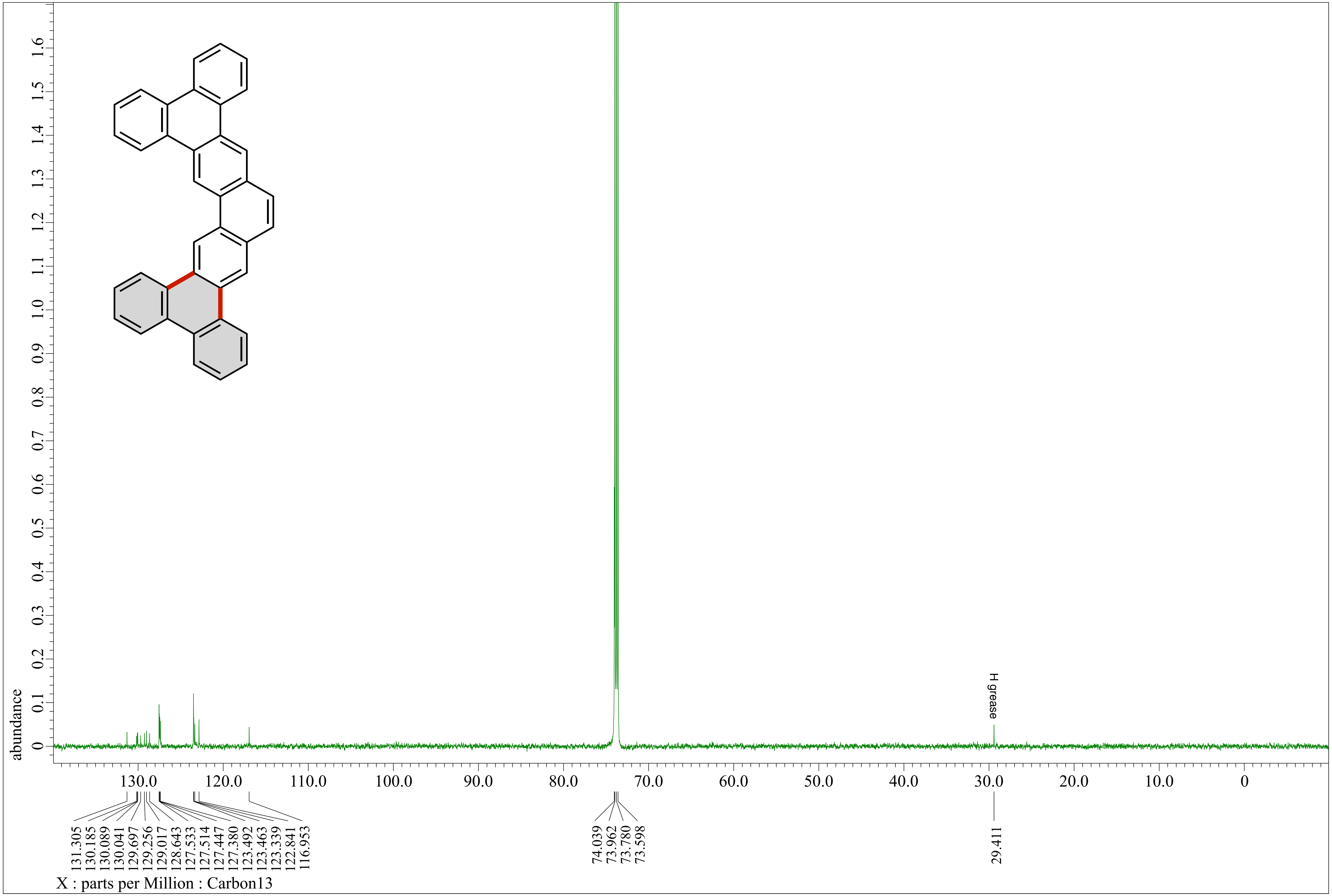

^{13}C NMR spectrum (inset) of **12** (solvent: $Cl_2CDCDCl_2$)

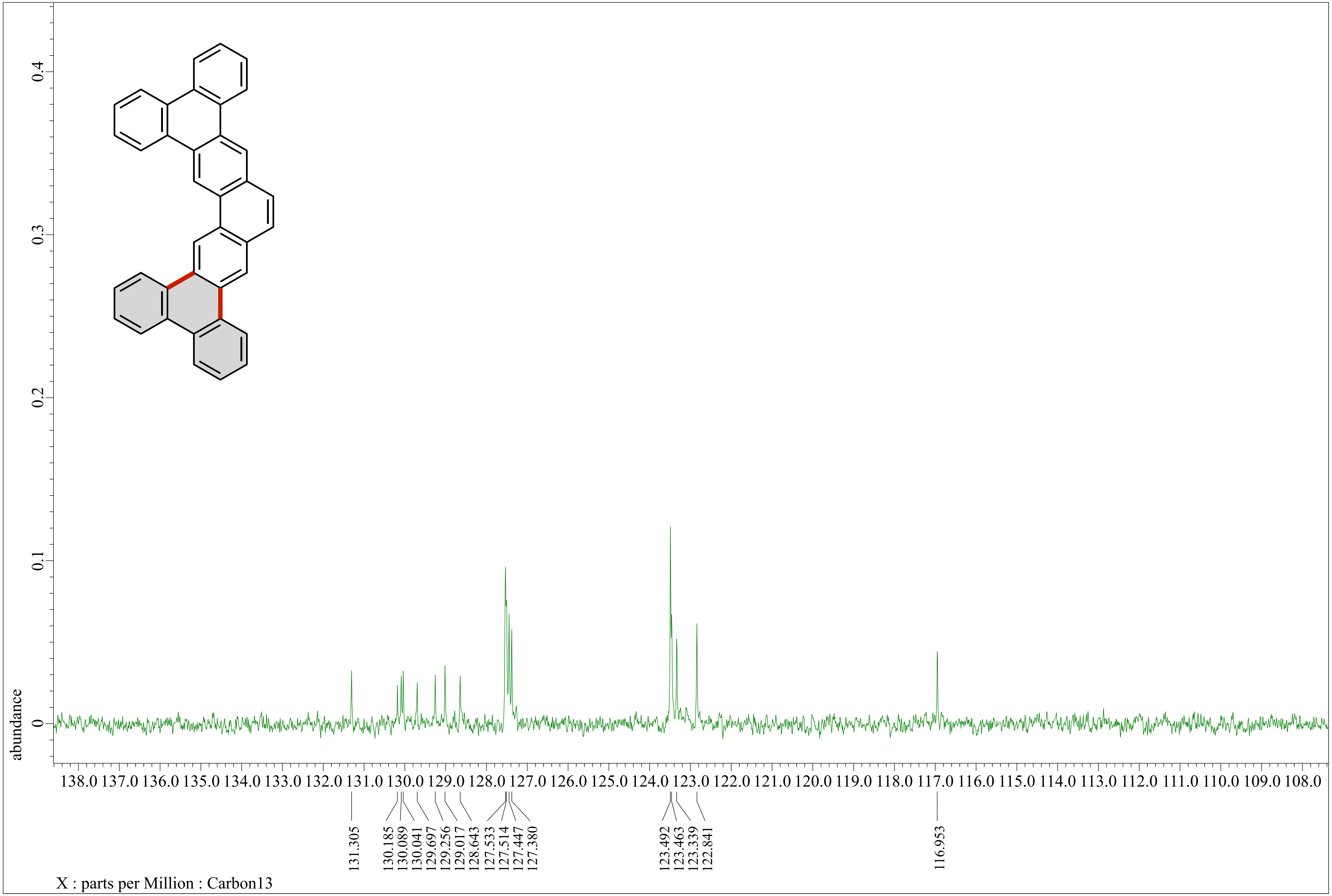

^{1}H NMR spectrum of **13** (solvent: $CDCl_3$)

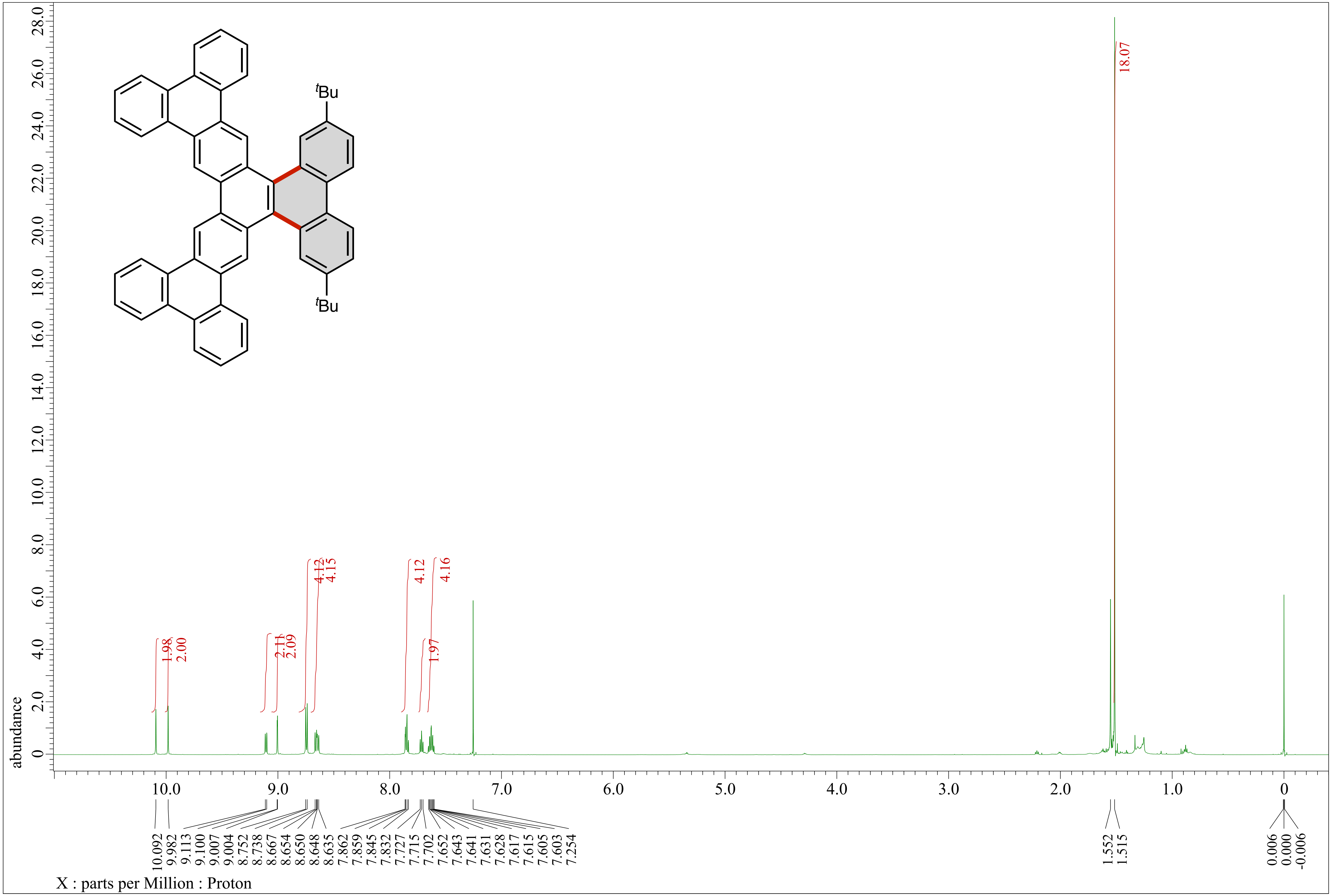

^{1}H NMR spectrum (inset) of **13** (solvent: $CDCl_3$)

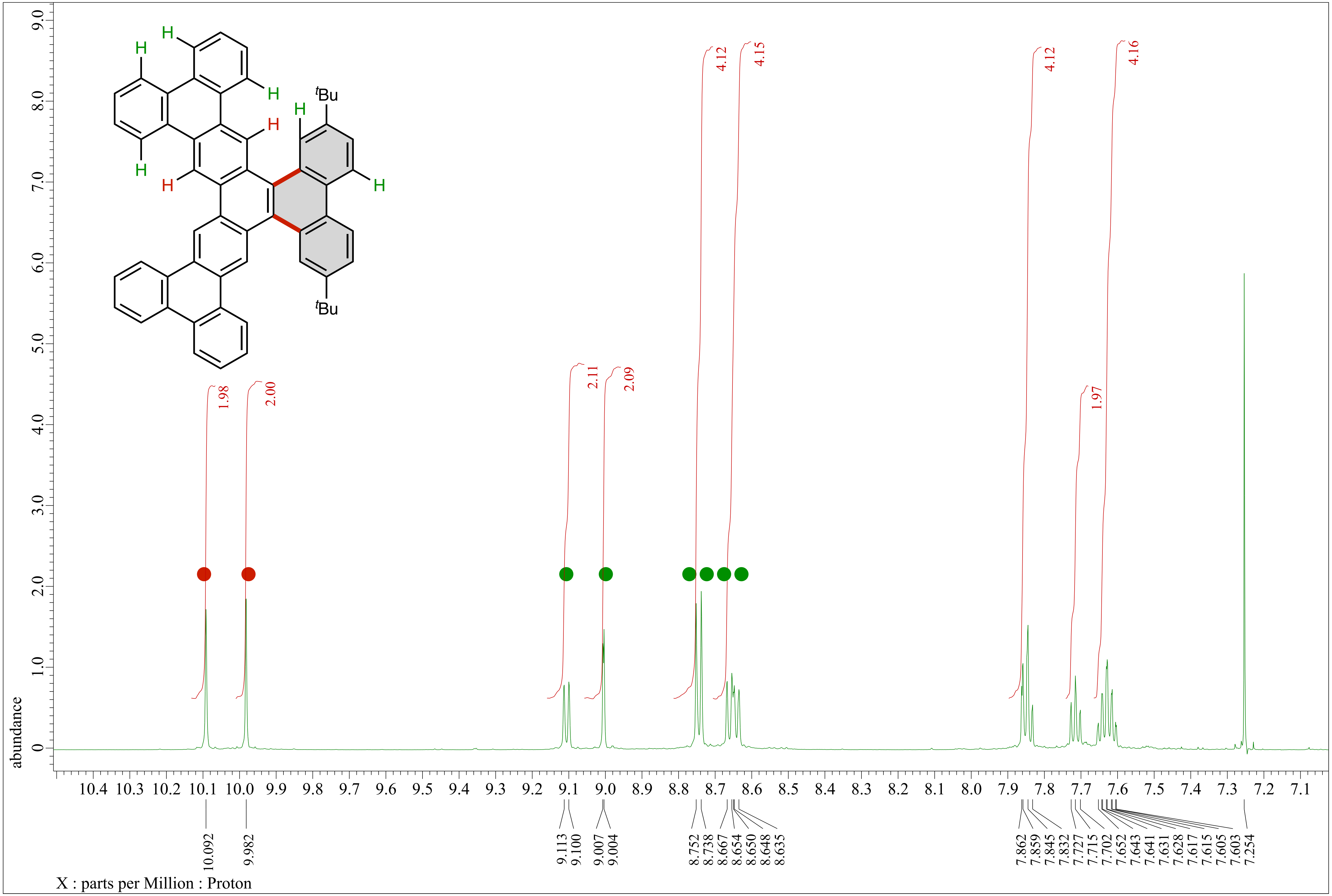

^{13}C NMR spectrum of **13** (solvent: $CDCl_3$)

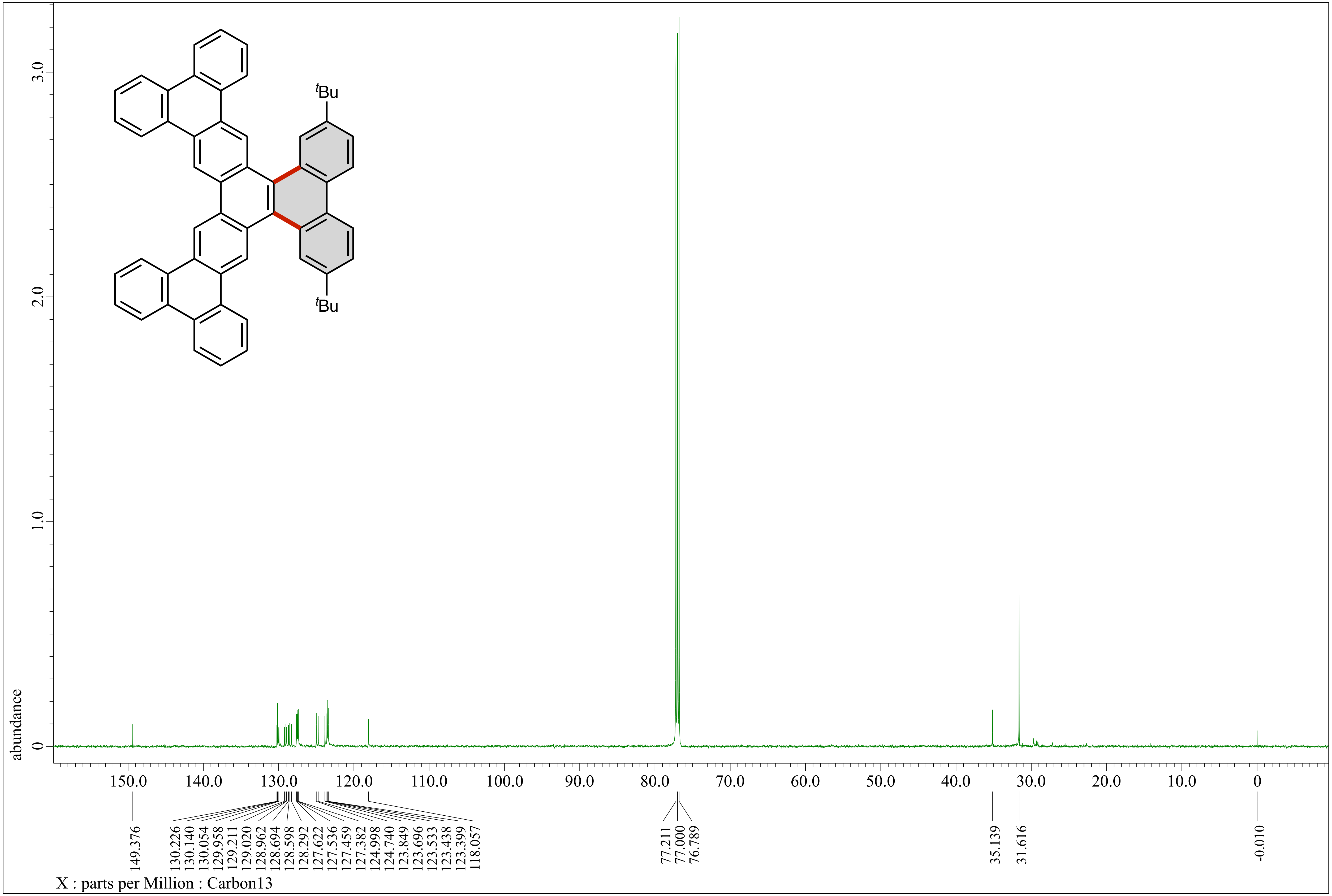

^{13}C NMR spectrum (inset) of **13** (solvent: $CDCl_3$)

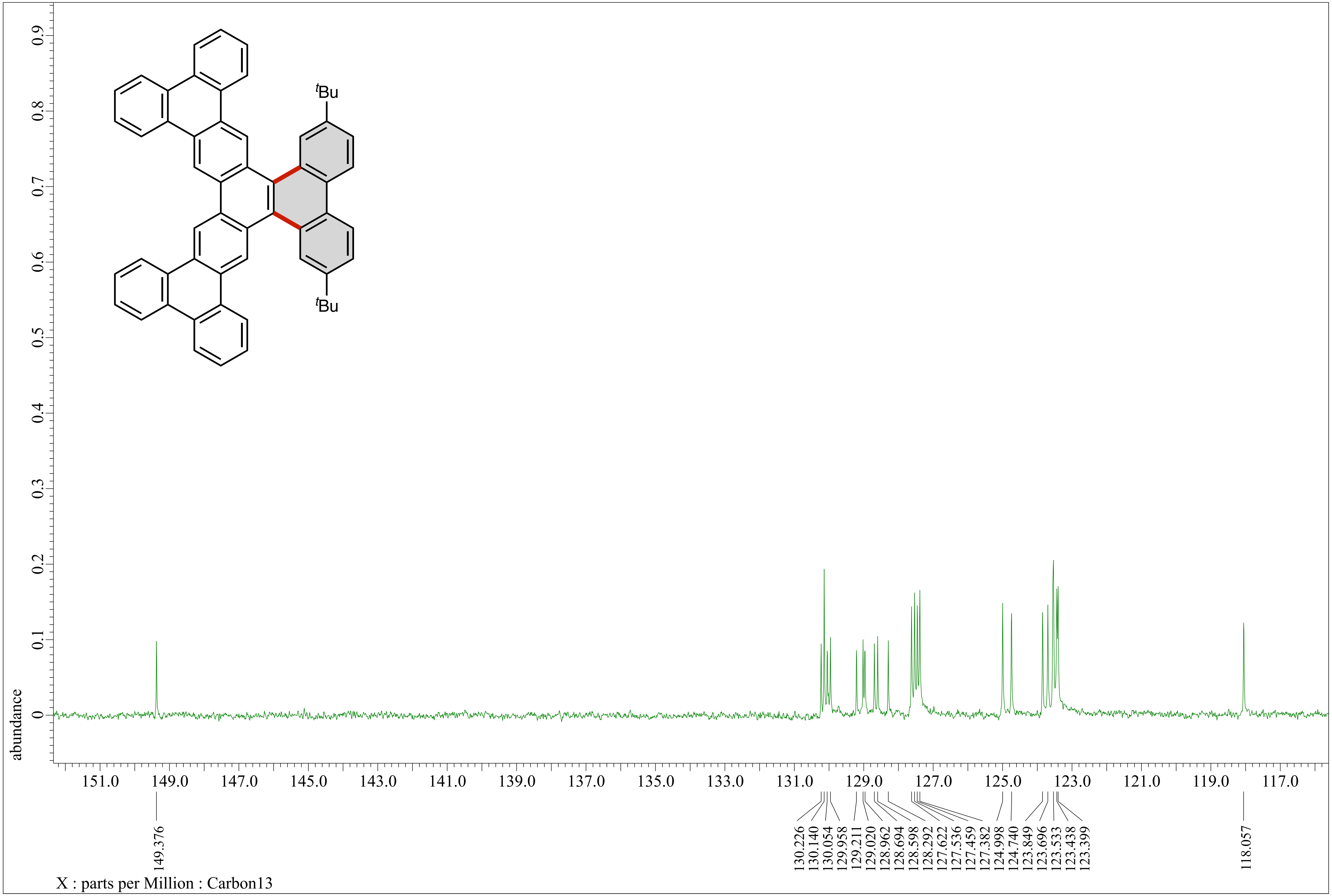

^{1}H NMR spectrum of **15** (solvent: $CDCl_3$)

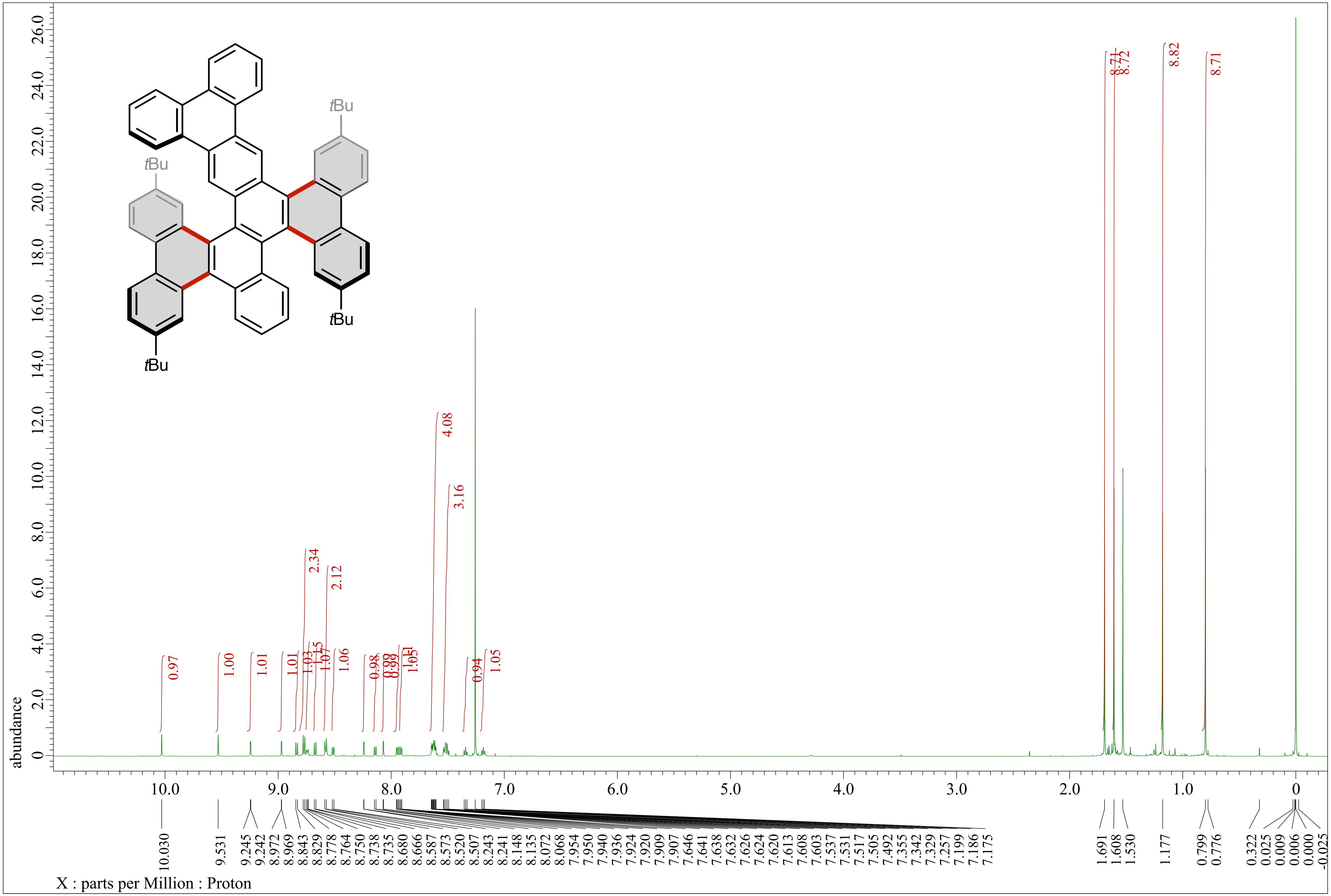

^{1}H NMR spectrum (inset) of **15** (solvent: $CDCl_3$)

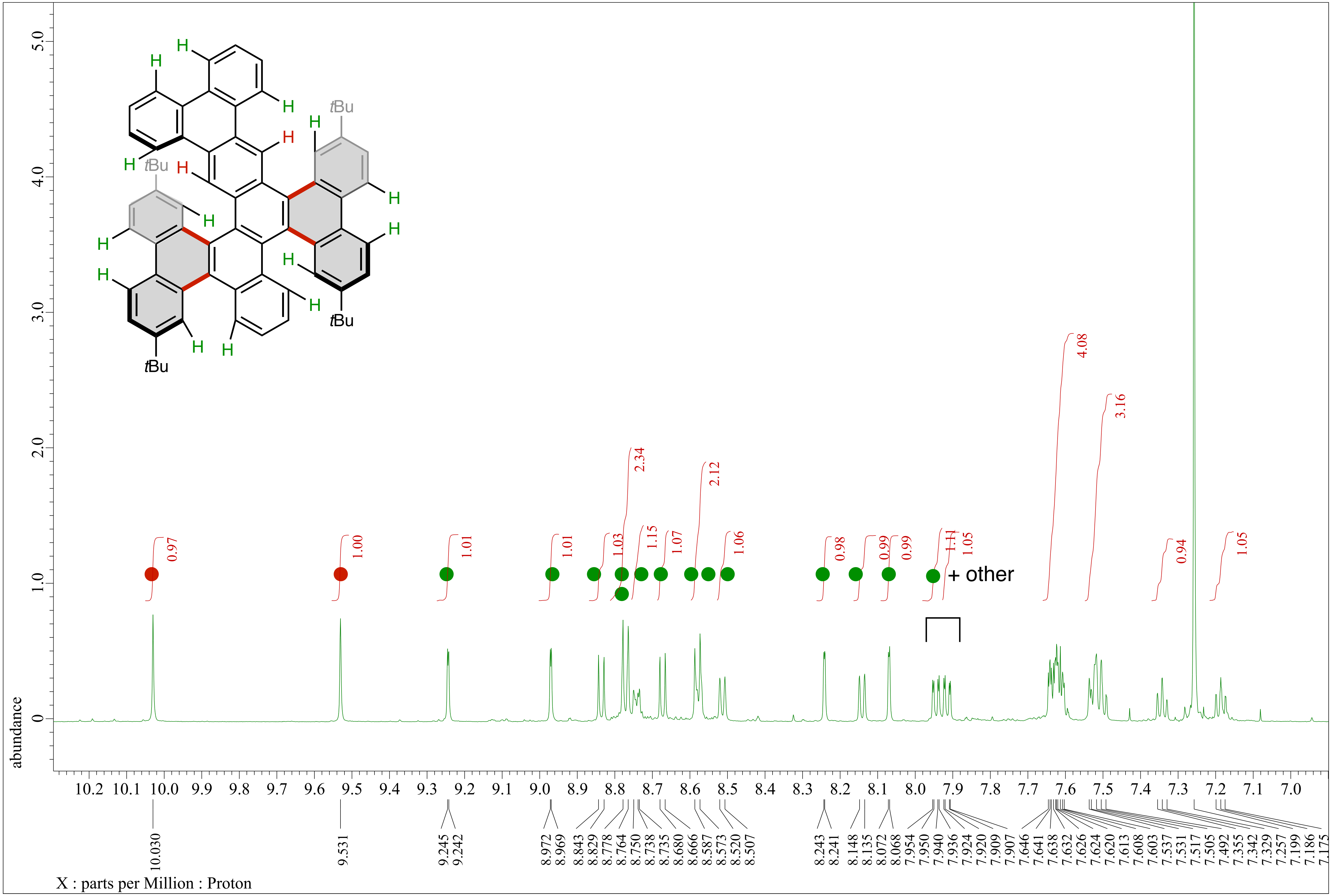

^{13}C NMR spectrum of **15** (solvent: $CDCl_3$)

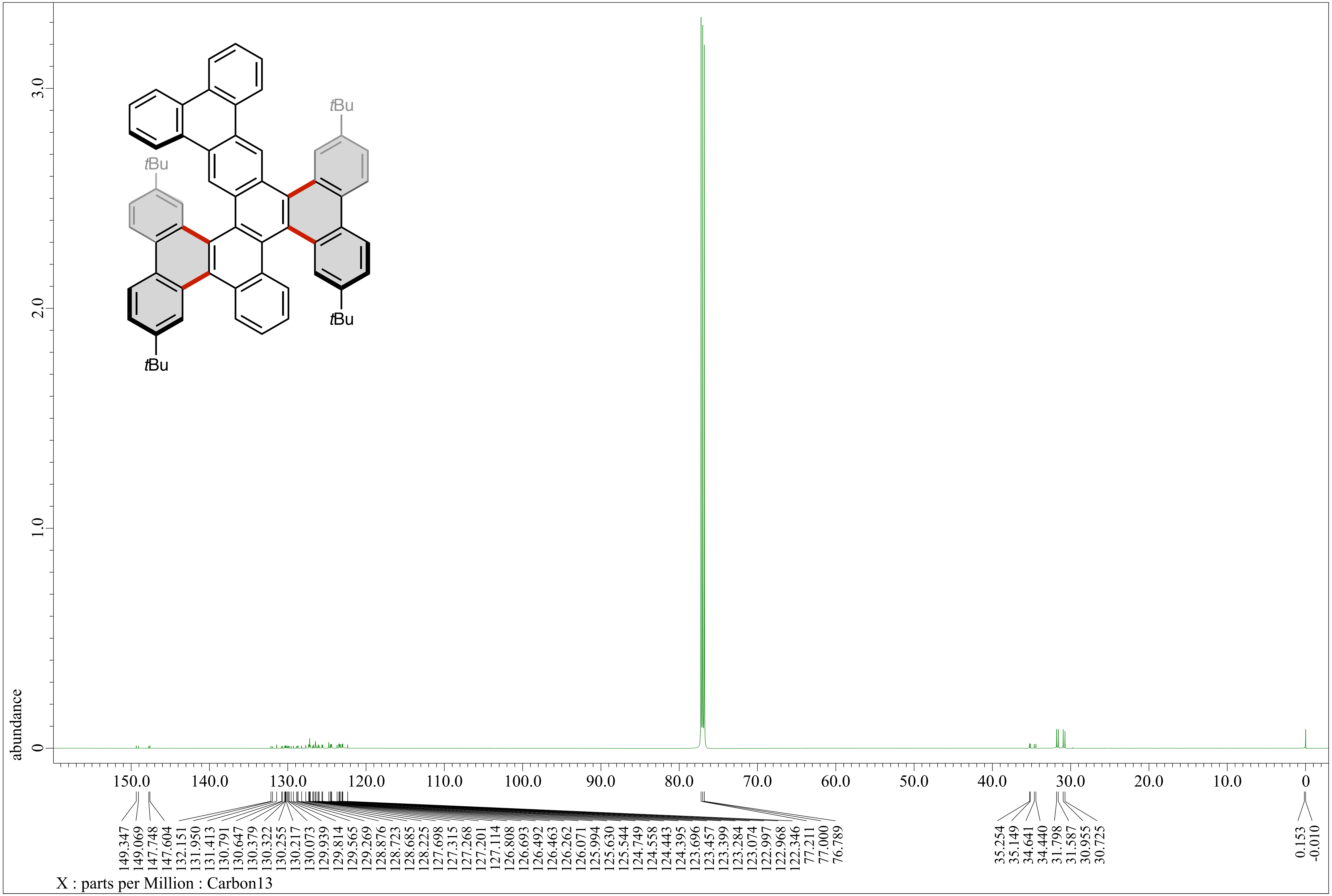

^{13}C NMR spectrum (inset) of **15** (solvent: $CDCl_3$)

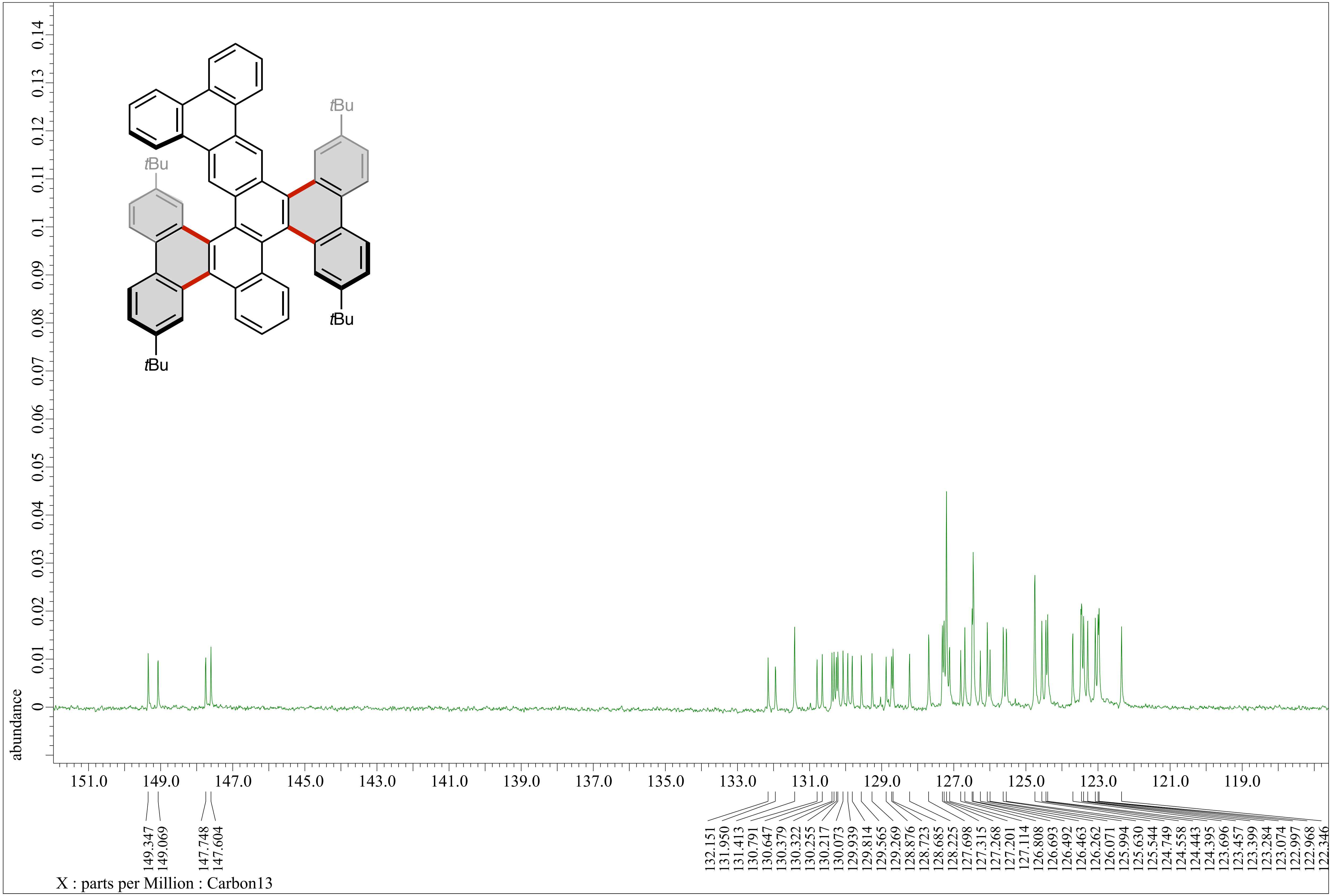

^{1}H NMR spectrum of **16** (solvent: $CDCl_3$)

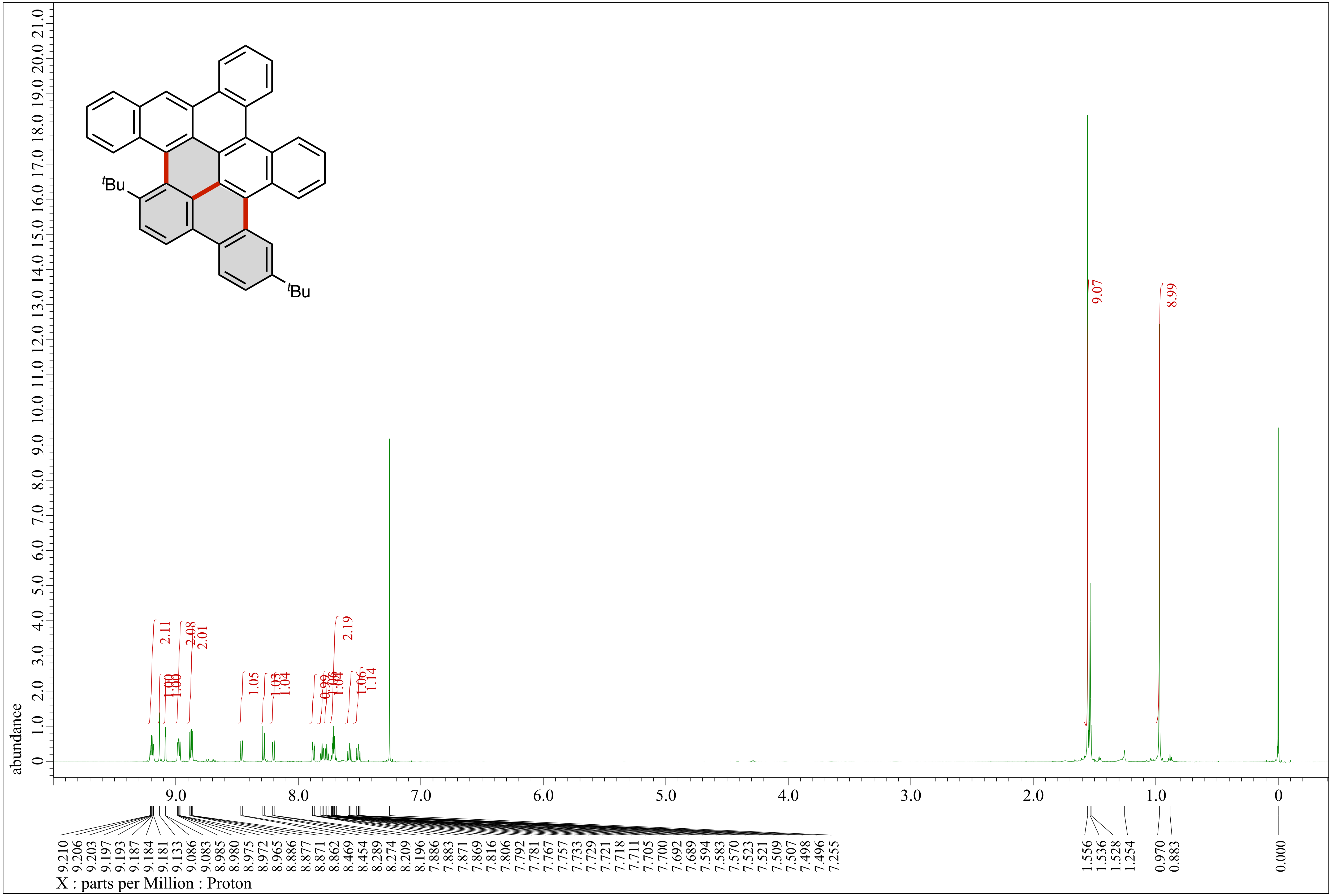

^{1}H NMR spectrum (inset) of **16** (solvent: $CDCl_3$)

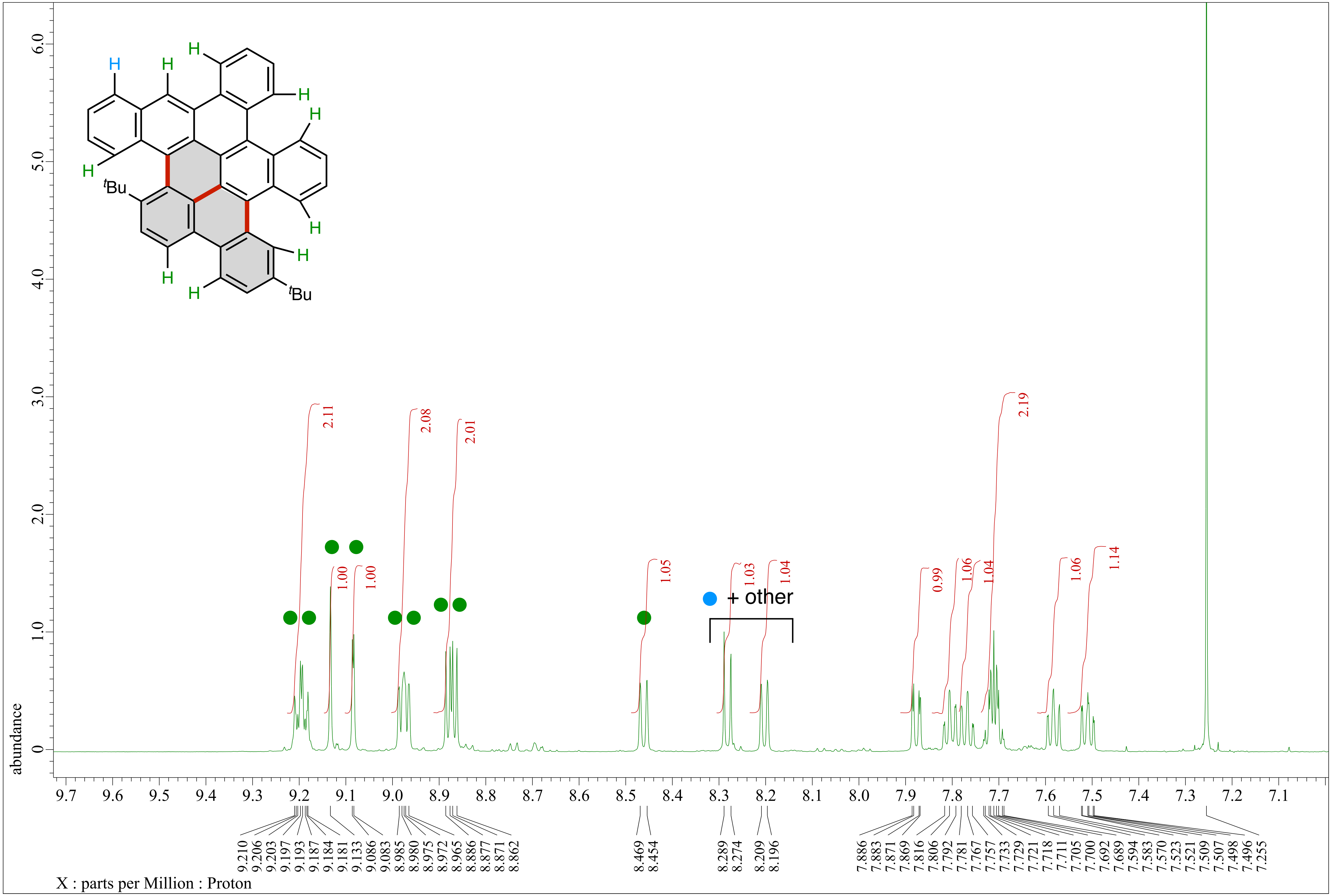

^{13}C NMR spectrum of **16** (solvent: $CDCl_3$)

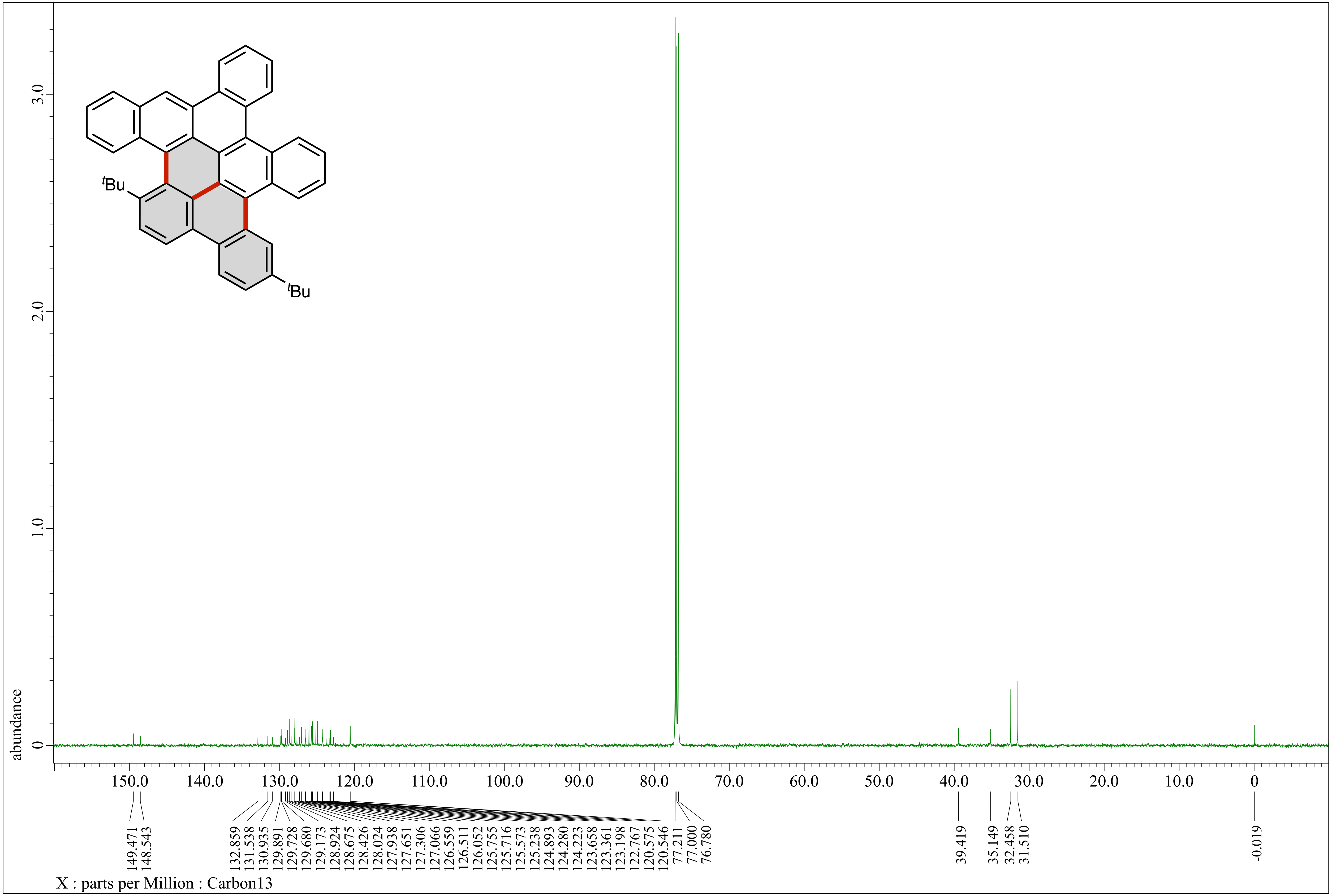

^{13}C NMR spectrum (inset) of **16** (solvent: $CDCl_3$)

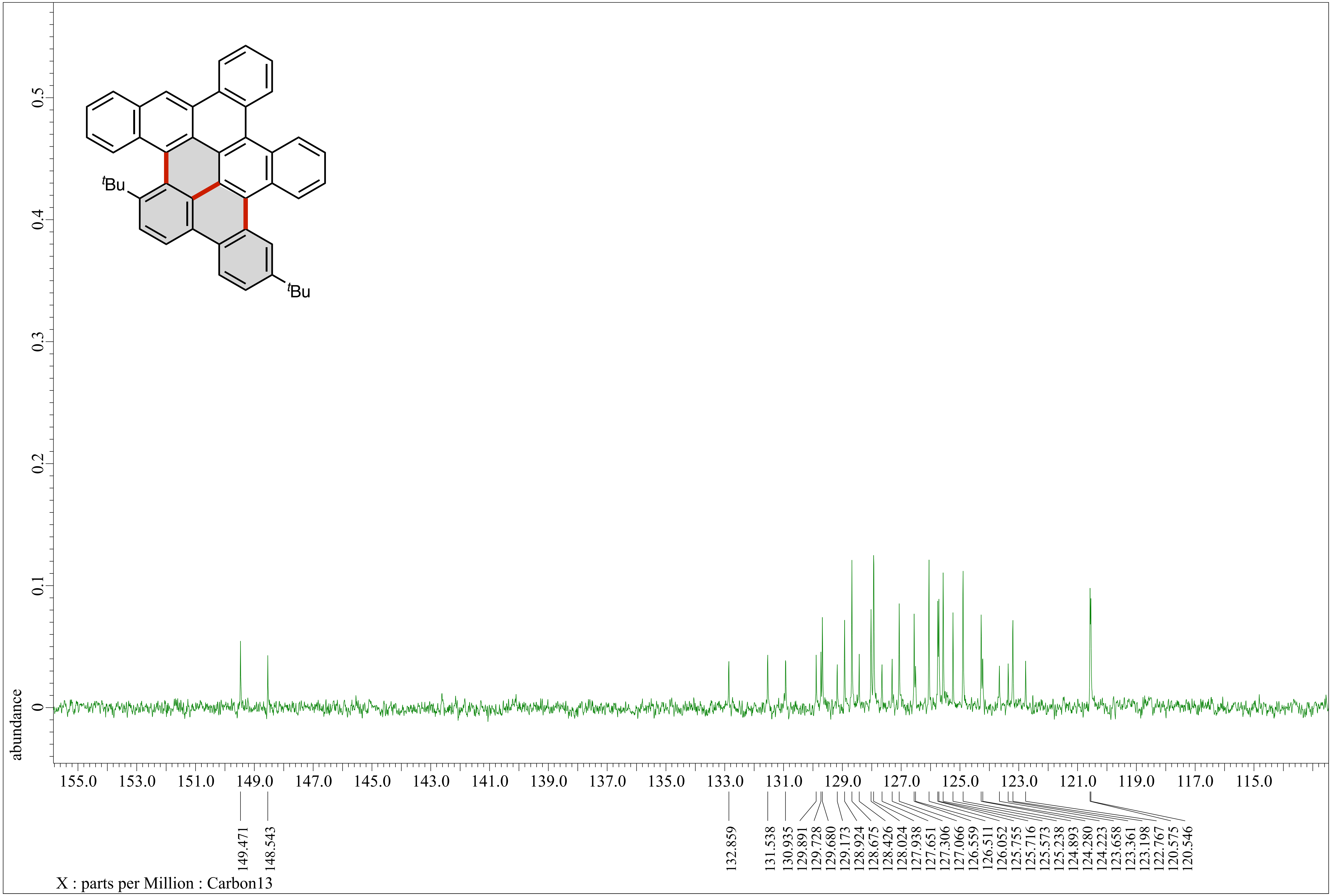

^{1}H NMR spectrum of **18** (solvent: $CDCl_3$)

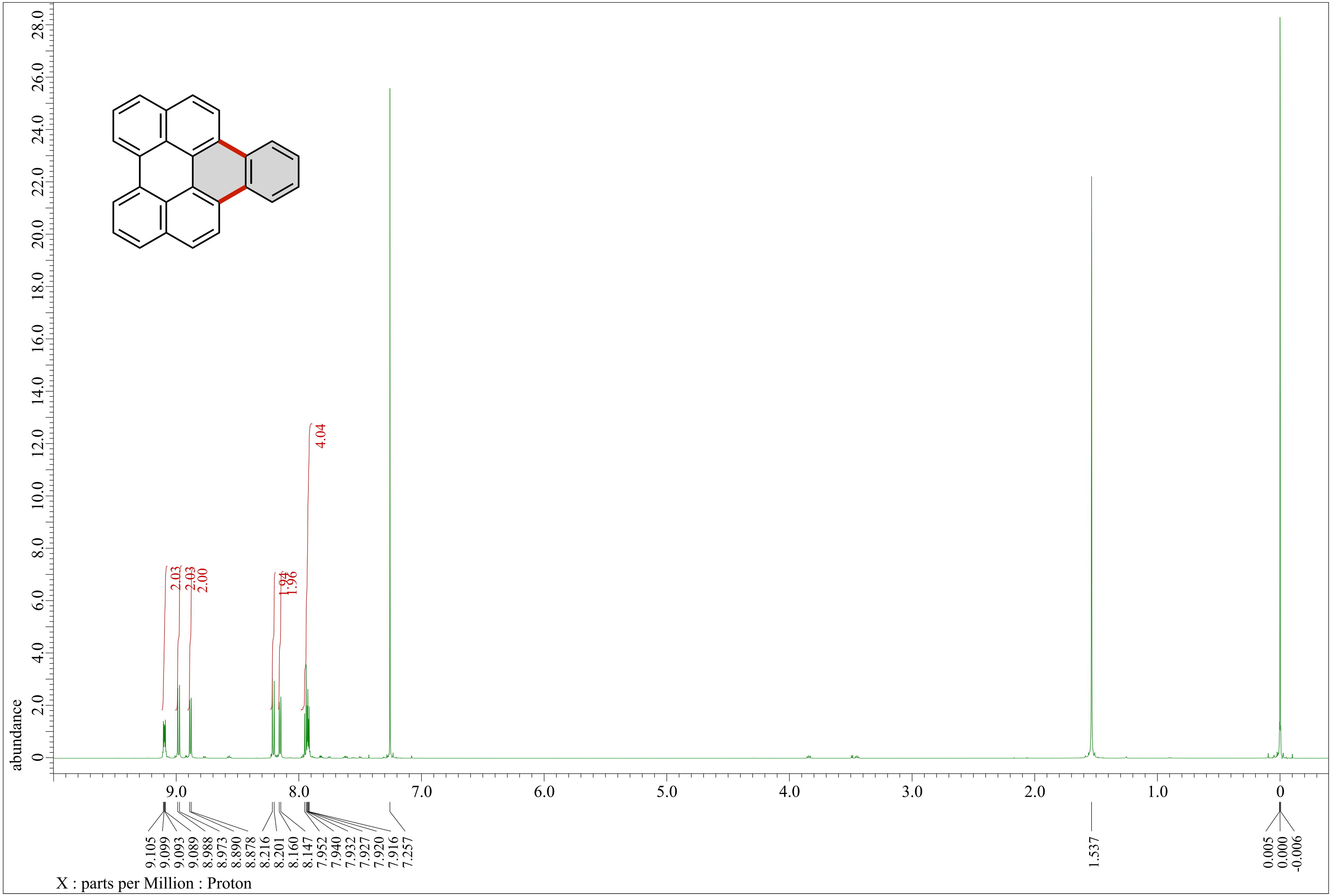

^{1}H NMR spectrum (inset) of **18** (solvent: $CDCl_3$)

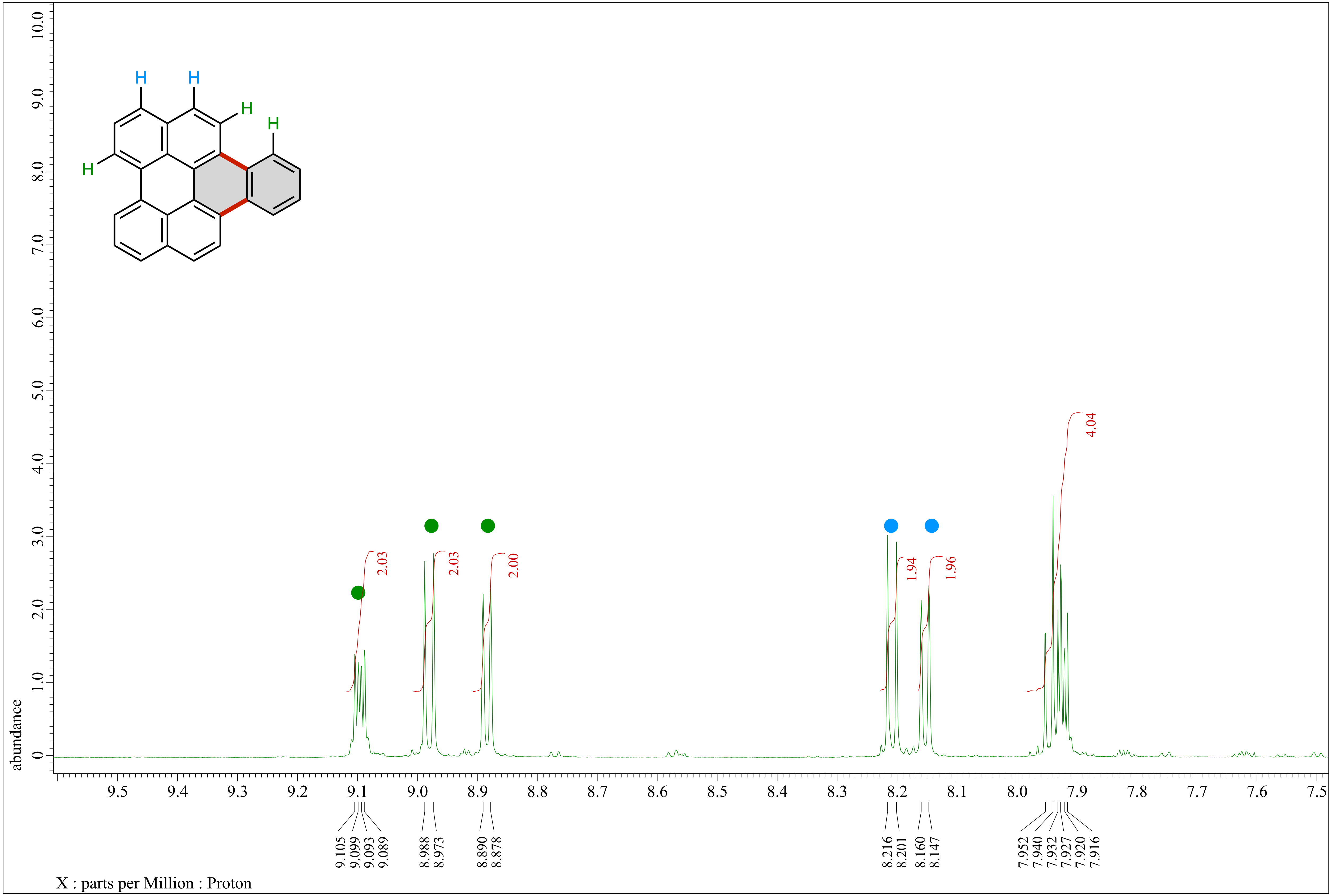

^{13}C NMR spectrum of **18** (solvent: $CDCl_3$)

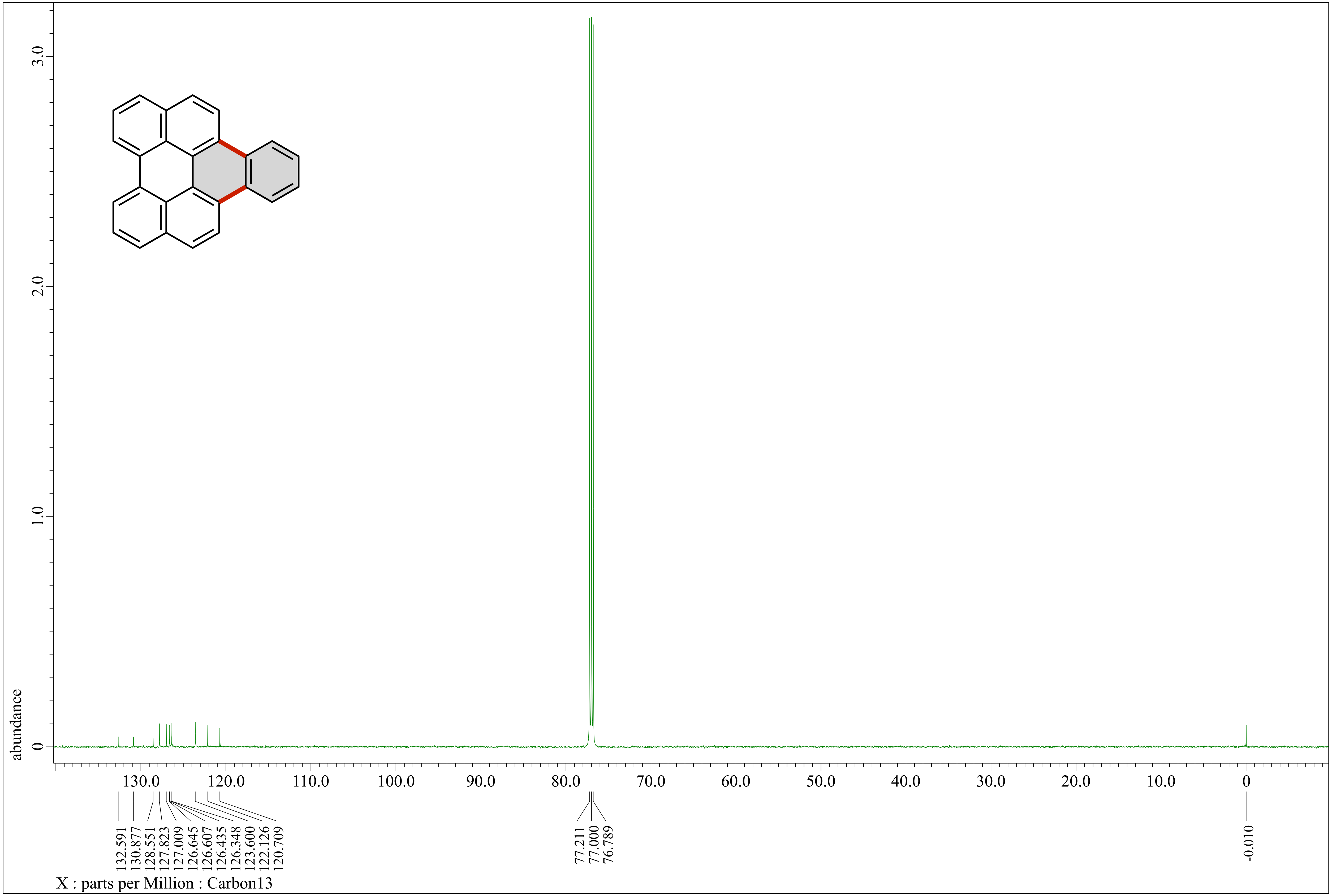

^{13}C NMR spectrum (inset) of **18** (solvent: $CDCl_3$)

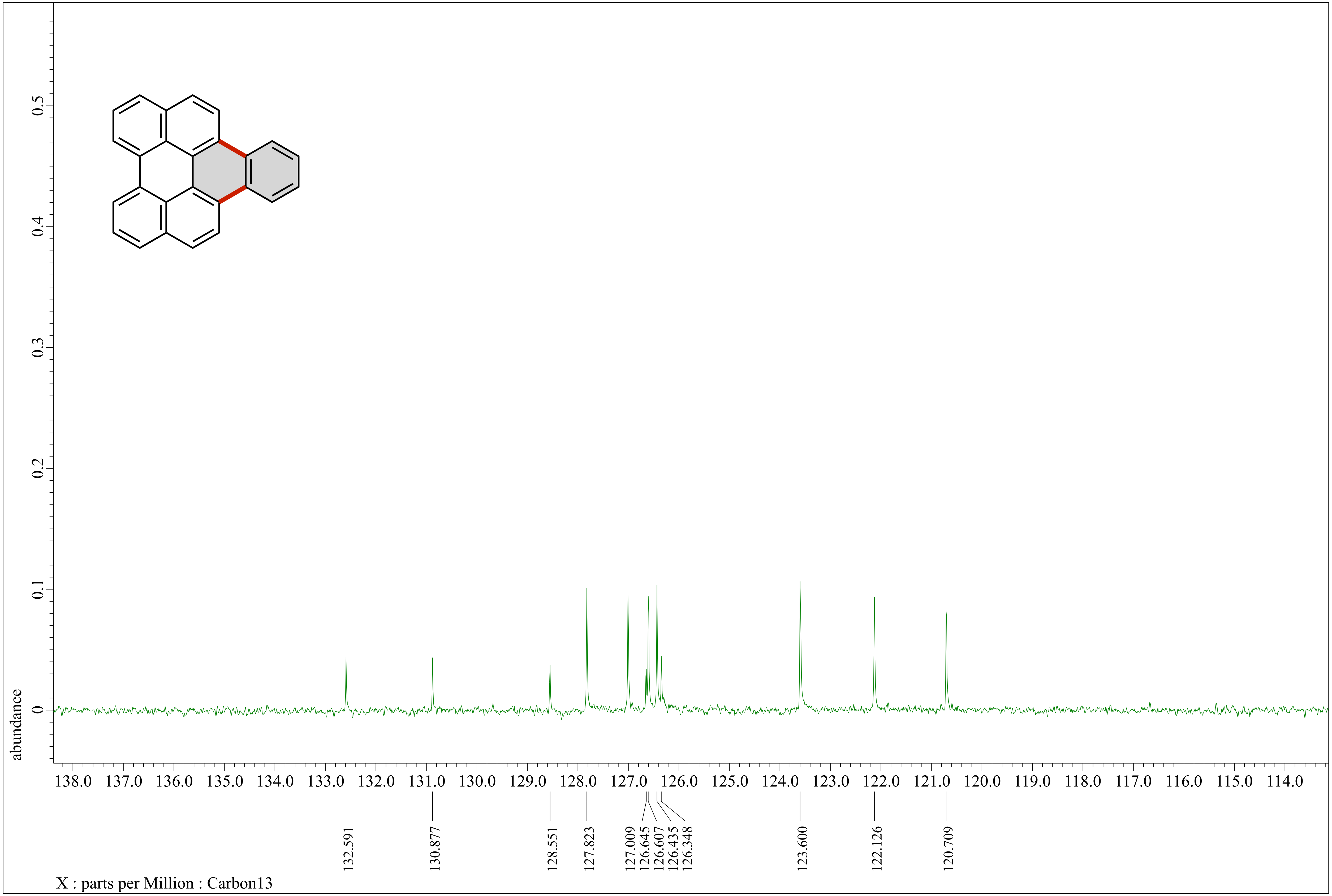

^{1}H NMR spectrum of **19** (solvent: $CDCl_3$)

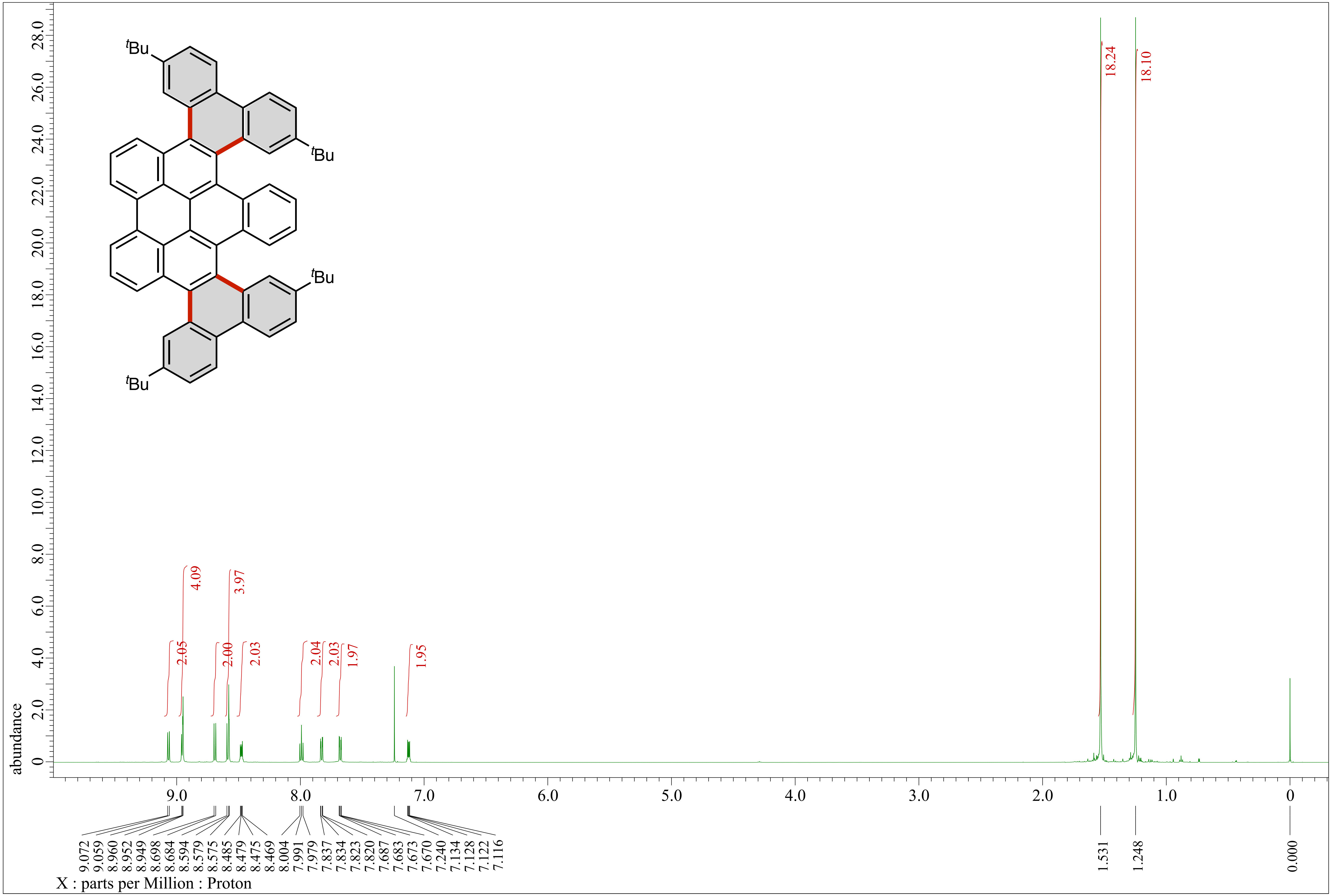

^{1}H NMR spectrum (inset) of **19** (solvent: $CDCl_3$)

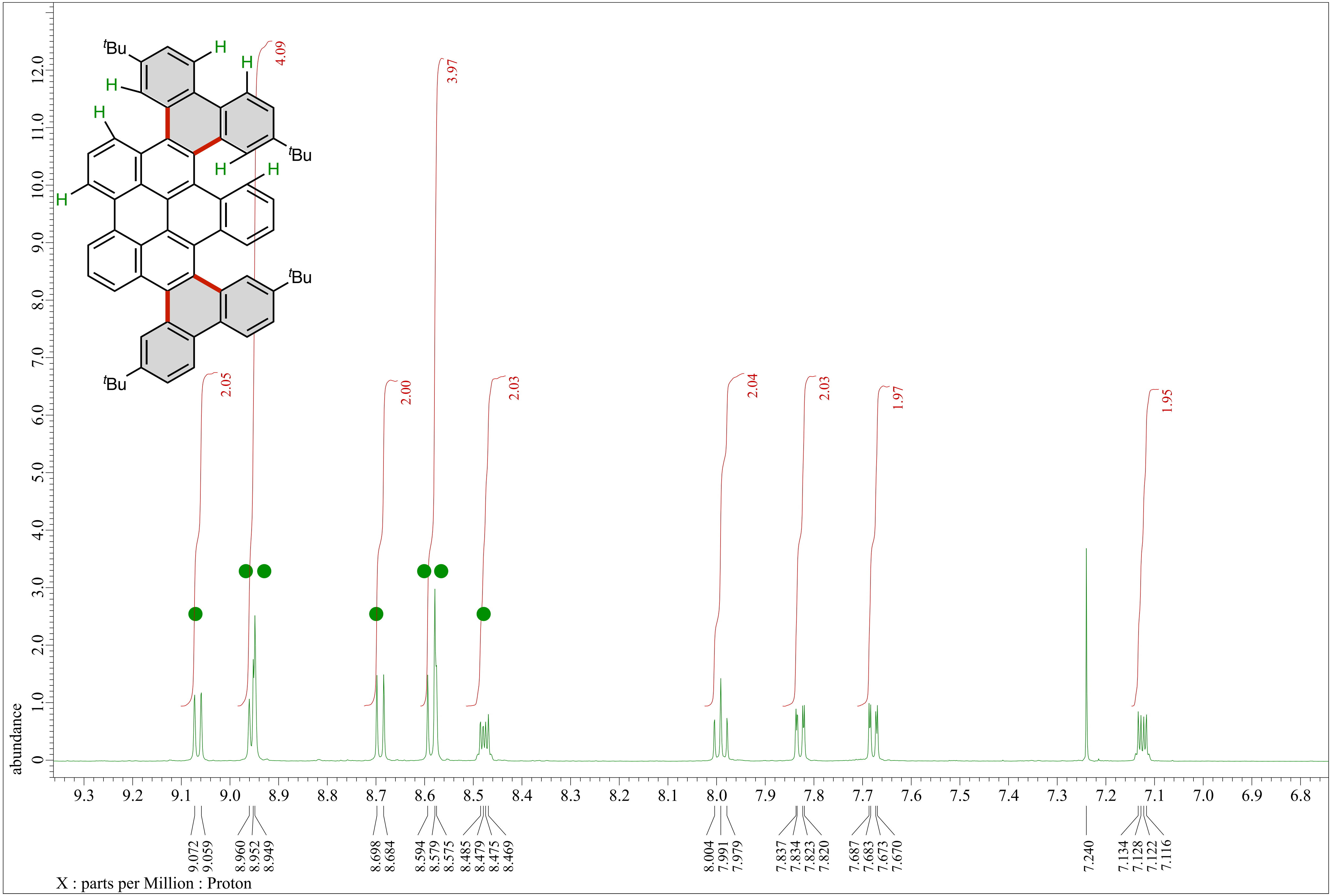

^{13}C NMR spectrum of **19** (solvent: $CDCl_3$)

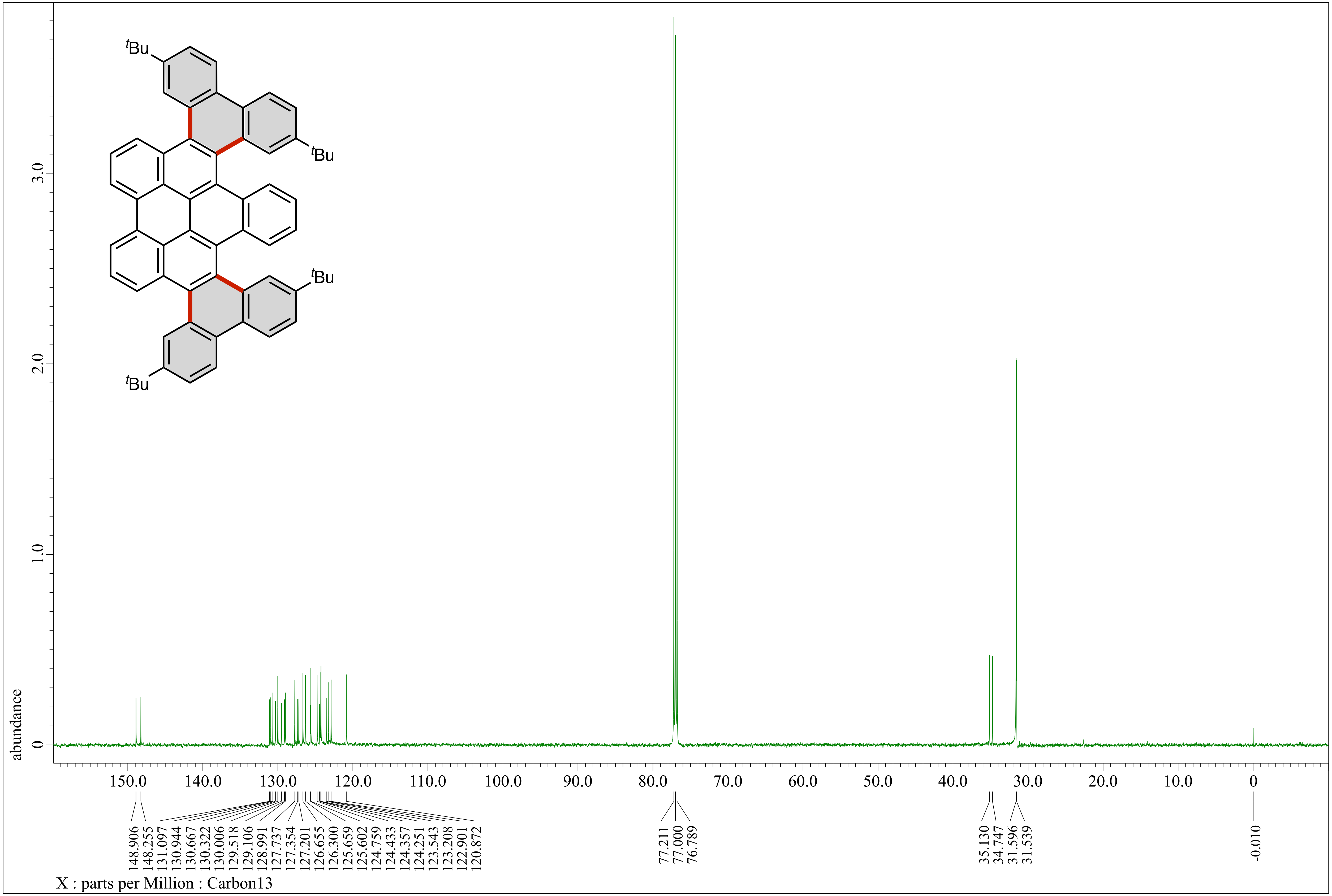

^{13}C NMR spectrum (inset) of **19** (solvent: $CDCl_3$)

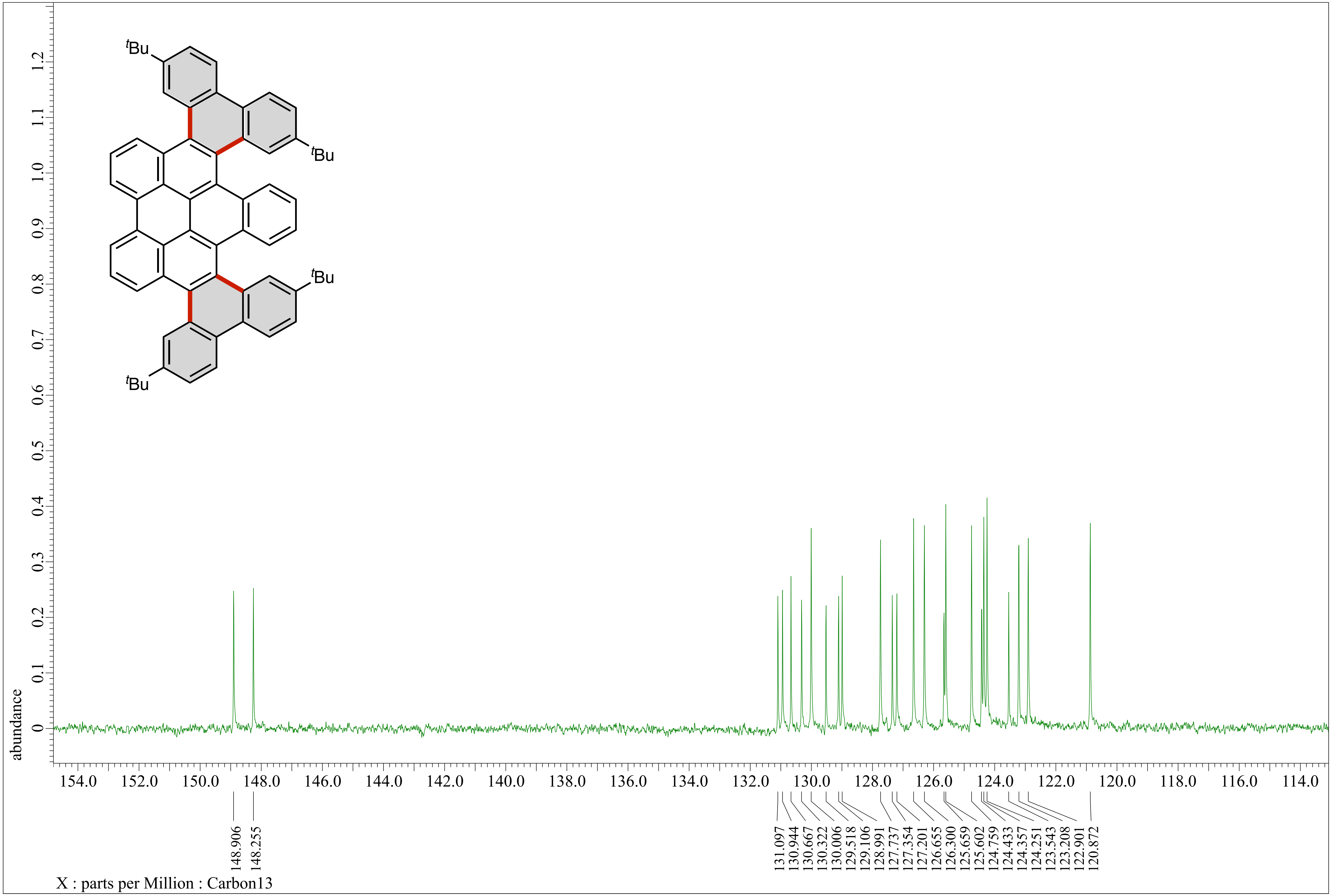

2. Supplementary References

1 Breton, G. W. & Turlington, M. Alternative synthetic routes to *N*-methyl-1,2,4-triazoline-3,5-dione (MeTAD) and other triazolinedione derivatives. *Tetrahedron Lett.* **55**, 4661-4663 (2014).

2 Cookson, R. C., Gupte, S. S., Stevens, I. D. R. & Watts, C. T. 4-Pheny-1,2,4-triazoline-3,5-dione. *Org. Synth.* **51**, 121 (1971).

3 Xi, X., Chen, T., Zhang, J.-S. & Han, L.-B. Efficient and selective hydrogenation of C–O bonds with a simple sodium formate catalyzed by nickel. *Chem. Commun.* **54**, 1521-1524 (2018).

4 Fu, W. C., Wang, Z., Chan, W. T. K., Lin, Z. & Kwong, F. Y. Regioselective synthesis of polycyclic and heptagon-embedded aromatic compounds through a versatile π-extension of aryl halides. *Angew. Chem. Int. Ed.* **56**, 7166-7170 (2017).

5 Fürstner, A. & Mamane, V. Flexible synthesis of phenanthrenes by a $PtCl_2$-catalyzed cycloisomerization reaction. *J. Org. Chem.* **67**, 6264-6267 (2002).

6 Wei, B., Zhang, D., Chen, Y.-H., Lei, A. & Knochel, P. Preparation of polyfunctional biaryl derivatives by cyclolanthanation of 2-bromobiaryls and heterocyclic analogues using $n\mathrm{Bu_2LaCl{\cdot}4LiCl}$. *Angew. Chem. Int. Ed.* **58**, 15631-15635 (2019).

7 Wu, B. & Yoshikai, N. Conversion of 2-iodobiaryls into 2,2′-diiodobiaryls via oxidation-iodination sequences: A versatile route to ladder-type heterofluorenes. *Angew. Chem. Int. Ed.* **54**, 8736-8739 (2015).

8 Xu, L., Zhang, S. & Li, P. Synthesis of silafluorenes and silaindenes via silyl radicals from arylhydrosilanes: Intramolecular cyclization and intermolecular annulation with alkynes. *Org. Chem. Front.* **2**, 459-463 (2015).

9 Tu, J., Li, G., Zhao, X. & Xu, F. Synthesis of triphenylene derivatives by Pd-catalyzed Suzuki coupling/ intramolecular C–H activation between arylboronic acids and dibromobiphenyls. *Tetrahedron Lett.* **60**, 44-47 (2019).

10 Leifert, D. & Studer, A. 9-Silafluorenes via base-promoted homolytic aromatic substitution (BHAS) – The electron as a catalyst. *Org. Lett.* **17**, 386-389 (2015).

11 Xu, S. *et al.* Palladium-catalyzed formal [4+1] annulation via metal carbene migratory insertion and C(sp^2)–H bond functionalization. *ACS Catal.* **7**, 1993-1997 (2017).

12 Nagamoto, Y., Yamaoka, Y., Fujimura, S., Takemoto, Y. & Takasu, K. Synthesis of functionalized polycyclic aromatic compounds via a formal [2+2]-cycloaddition. *Org. Lett.* **16**, 1008-1011 (2014).

13 Shi, G., Chen, D., Jiang, H., Zhang, Y. & Zhang, Y. Synthesis of fluorenes starting from 2-iodobiphenyls and CH_2Br_2 through palladium-catalyzed dual C–C bond formation. *Org. Lett.* **18**, 2958-2961 (2016).

14 Schlosser, M., Mangano, G. & Leroux, F. The superbase-mediated pairwise substitution of the 2,2′- and 6,6′-positions in a biphenyl derivative. *Eur. J. Org. Chem.* **2014**, 1014-1017 (2014).

15 Zhu, R.-Y., He, J., Wang, X.-C. & Yu, J.-Q. Ligand-promoted alkylation of C(sp^3)–H and C(sp^2)–H bonds *J. Am. Chem. Soc.* **136**, 13194-13197 (2014).

16 Sheldrick, G. M. SHELXT - integrated space-group and crystal-structure determination. *Acta Crystallogr.* **A71**, 3-8 (2015).

17 Sheldrick, G. M. Crystal structure refinement with SHELXL. *Acta Crystallogr.* **C71**, 3-8 (2015).

18 Dolomanov, O. V., Bourhis, L. J., Gildea, R. J. Howard, J. A. K. & Puschmann, H. Olex2: A complete structure solution, refinement and analysis program. *J. Appl. Crystallogr.* **42**, 339-341 (2009).

19 Frisch, M. J. *et al. Gaussian 16, Revision C.01*, Gaussian, Inc., Wallingford CT, 2016.

20 Zhao, Y. & Truhlar, G. D. The M06 suite of density functionals for main group thermochemistry, thermochemical kinetics, noncovalent interactions, excited states, and transition elements: two new functionals and systematic testing of four M06-class functionals and 12 other functionals. *Theor. Chem. Acc.* **120**, 215-241 (2008).

21 Hehre, W. J., Ditchfield, R. & Pople, J. A. Self-consistent molecular orbital methods. XII. Further extensions of gaussian-type basis sets for use in molecular orbital studies of organic molecules. *J. Chem. Phys.* **56**, 2257-2261 (1972).

22 Clark, T., Chandrasekhar, J., Spitznagel, G. W. & Schleyer, P. V. Efficient diffuse function-augmented basis sets for anion calculations. III. The 3-21+G basis set for first-row elements, Li-F. *J. Comput. Chem.* **4**, 294-301 (1983).

23 Frisch, M. J., Pople, J. A. & Binkley, J. S. Self-consistent molecular orbital methods 25. Supplementary functions for Gaussian basis sets. *J. Chem. Phys.* **80**, 3265-3269 (1984).

24 Fukui, K. The Path of Chemical Reactions – The IRC Approach. *Acc. Chem. Res.* **14**, 363-368 (1981).

25 Legault, C. Y. CYLview20. Université de Sherbrooke, http://www.cylview.org (2020).
