# Supplementary material for: Diversity-oriented synthesis of nanographenes enabled by dearomative annulative π-extension
Source: Nat Commun. 2021 Jun 24;12:3940. doi: 10.1038/s41467-021-24261-y (PMC8225822; doi:10.1038/s41467-021-24261-y)
Supplement: Supplementary file 2 — Description of Additional Supplementary Files [file 41467_2021_24261_MOESM2_ESM.docx]

Supplementary Data 1

Cartesian coordinates for all calculated geometries.
